# Single-Cell RNA-Seq Analysis of Cells from Degenerating and Non-Degenerating Intervertebral Discs from the Same Individual Reveals New Biomarkers for Intervertebral Disc Degeneration

**DOI:** 10.3390/ijms23073993

**Published:** 2022-04-03

**Authors:** Hosni Cherif, Matthew Mannarino, Alain Sarabia Pacis, Jiannis Ragoussis, Oded Rabau, Jean A. Ouellet, Lisbet Haglund

**Affiliations:** 1 Orthopaedic Research Lab, Department of Surgery, The Research Institute of the McGill University Health Centre, McGill University, Montreal, QC H3A 0G4, Canada; hosni.cherif@mail.mcgill.ca (H.C.); matthew.mannarino@mail.mcgill.ca (M.M.); 2 McGill Scoliosis and Spine Group, Department of Surgery, The Research Institute of the McGill University Health Centre, McGill University, Montreal, QC H3A 0G4, Canada; odedrabau@gmail.com (O.R.); jaouellet@hotmail.com (J.A.O.); 3 Canadian Centre for Computational Genomics, McGill University Genome Center, 740 Dr. Penfield Avenue, Montreal, QC H3A 0G4, Canada; alain.pacis@mcgill.ca; 4 Department of Human Genetics, McGill University Genome Center, 740 Dr. Penfield Avenue, Montreal, QC H3A 0G4, Canada; ioannis.ragoussis@mcgill.ca; 5 Department of Pediatric Orthopedics, McGill University, Shriner’s Hospital for Children, 1003 Decarie Blvd, Montreal, QC H3A 0G4, Canada

**Keywords:** single cell RNA sequencing, intervertebral disc, cartilage, disc degeneration, chondrocytes, senescence, cell biology, transcription factors

## Abstract

In this study, we used single-cell transcriptomic analysis to identify new specific biomarkers for nucleus pulposus (NP) and inner annulus fibrosis (iAF) cells, and to define cell populations within non-degenerating (nD) and degenerating (D) human intervertebral discs (IVD) of the same individual. Cluster analysis based on differential gene expression delineated 14 cell clusters. Gene expression profiles at single-cell resolution revealed the potential functional differences linked to degeneration, and among NP and iAF subpopulations. GO and KEGG analyses discovered molecular functions, biological processes, and transcription factors linked to cell type and degeneration state. We propose two lists of biomarkers, one as specific cell type, including *C2orf40*, *MGP*, *MSMP*, *CD44*, *EIF1*, *LGALS1*, *RGCC*, *EPYC*, *HILPDA*, *ACAN*, *MT1F*, *CHI3L1*, *ID1*, *ID3* and *TMED2*. The second list proposes predictive IVD degeneration genes, including *MT1G*, *SPP1*, *HMGA1*, *FN1*, *FBXO2*, *SPARC*, *VIM*, *CTGF*, *MGST1*, *TAF1D*, *CAPS*, *SPTSSB*, *S100A1*, *CHI3L2*, *PLA2G2A*, *TNRSF11B*, *FGFBP2*, *MGP*, *SLPI*, *DCN*, *MT-ND2*, *MTCYB*, *ADIRF*, *FRZB*, *CLEC3A*, *UPP1*, *S100A2*, *PRG4*, *COL2A1*, *SOD2* and *MT2A*. Protein and mRNA expression of *MGST1*, *vimentin*, *SOD2* and *SYF2 (p29)* genes validated our scRNA-seq findings. Our data provide new insights into disc cells phenotypes and biomarkers of IVD degeneration that could improve diagnostic and therapeutic options.

## 1. Introduction

Intervertebral disc (IVD) degeneration and associated low back pain is a chronic pathophysiological condition experienced by ~80% of individuals at some time in their lifespan. It is a global health problem with increasing socioeconomic cost [1,2,3,4,5,6] for which there are no disease-modifying therapies. The IVD is a fibro-cartilaginous, physiologically avascular tissue with poor self-renewing capacity [7,8,9,10,11,12]. Structurally, IVDs are divided into distinct zones of cartilaginous endplates, the inner and outer annulus fibrosus (iAF and oAF), and the central nucleus pulposus (NP), each with characteristic cell populations, biochemical and biomechanical properties [13]. Metabolic dysregulation of the cells plays a pivotal role in the pathobiology and results in alterations in extracellular matrix (ECM) composition throughout the IVD compartments [3,13,14].

Currently described in the literature are a number of IVD cell biomarkers, including *Col1a1*, *Col4*, *Lam1* and *Thy1* for AF cells [15,16,17,18,19,20,21,22], and *Krt8*, *Krt18*, *Krt19*, *Ca12*, *ACAN*, *Col1a2*, *Col2a1*, *Tie2+* and *Gd2+* for NP cells [17,23,24,25,26,27,28]. However, a lack of information on how the cells [29] and biochemical markers vary in health and disease prevents us from effectively targeting the progression of IVD degeneration. Informative, transcriptomic profiles of cells from degenerating (D) and non-degenerating (nD) IVDs have relied either on bulk RNA sequencing, microarray-based approaches [30,31,32,33,34,35,36], or animal studies [18,28,37,38,39,40,41,42]. These findings are not always translatable because of interspecies differences and large variability observed when cell populations are pooled, averaging out important changes [33,43,44,45,46,47,48,49]. Our goal was to define transcriptional profiles of the cell populations found in D and nD adult human IVD tissue that could provide insight into disease etiology and treatment options. We aimed also to identify novel cell-type specific biomarkers for iAF and NP cells. Our study provides a valuable resource for further investigation to better elucidate phenotypes of NP and iAF cells with defined markers and molecular signatures related to IVD degeneration. Deepening our knowledge of IVD cell heterogeneity in D and nD tissue may shed light on the pathogenesis and potentially aid in the development of diagnostic and therapeutic options in the future.

## 2. Results

### 2.1. Cellular Heterogeneity of Human Intervertebral Disc Cells

**Figure 1:**
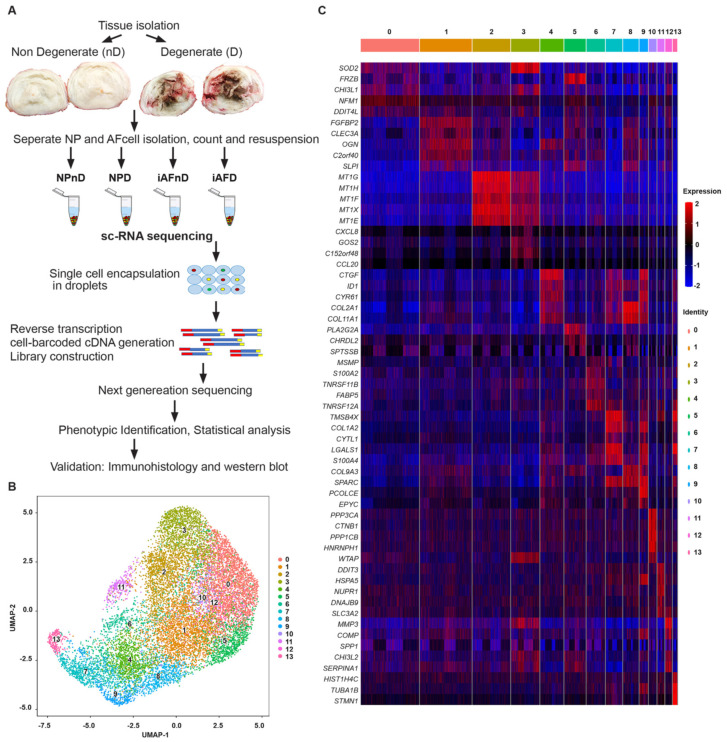
Single-cell RNA sequencing reveals heterogeneity of the human disc cells. (**A**) Schematic overview of the workflow of isolating and analyzing iAF and NP cells from human non-degenerating (nD) and degenerating (D) discs. (**B**) UMAP plot showing the unbiased classification of 3.131 iAFnD, 3.867 NPnD, 3.092 iAFD and 3.646 NPD. Cells are clustered according to transcriptome similarity in 2D space. Each dot represents one cell, colored by cluster. UMAP plot revealed that cells in the human disc are present in 14 unique clusters (0–13). (**C**) Heat map showing the top marker genes of each cluster (0–13) as determined by Seurat analysis. Expression of genes is represented using a z-score value in which red indicates higher expression and blue indicates lower expression.

Single-cell gene expression analysis can improve assessment of transcriptomic variation of individual cell populations hidden in bulk analysis. We used scRNA-seq of human NP and iAF cells from D and nD IVDs of the same individual to avoid confounding factors such as genetic background, age, sex, and lifestyle of separate individuals [50]. In total, we analyzed, 13,736 scRNA-seq profiles (Figure 1A, Table S1 and Supplemental Figure S1A–F), divided between 3131 (iAFnD), 3092 (iAFD), 3867 (NPnD) and 3646 (NPD) individual cells at a median sequencing depth of 43,000 reads/cell. The number of cells passing filtering allowed for reliable detection of gene classes (Figure 1A). The R package, Seurat [51,52] and Uniform manifold approximation and projection (UMAP) [53], was used to cluster and visualize all cells together (iAFnD, iAFD, NPnD and NPD) based on their transcriptional similarity. Unsupervised UMAP analysis revealed fourteen transcriptionally distinct subpopulations (Figure 1B), all expressing disc cell markers such as collagen and aggrecan. Next, we compared gene expression levels in the identified clusters to determine cluster marker genes. Known and novel DEGs were found, including *SOD2*, *FRZB*, *CHI3L1*, *SYF2* (cluster 0), *FGFBP2*, *CLEC3A*, *OGN*, *C2orf40* (cluster 1), *MT1G*, *MT1H*, *MT1F*, *MT1X* (cluster 2), *CXCL8*, *G0S2*, *CCL20* (cluster 3), *CTGF*, *ID1*, *COL2A1*, *COL11A1* (cluster 4), *FRZB*, *PLA2G2A*, *CHRDL2*, *SPTSSB* (cluster 5), *MSMP*, *S100A2*, *TNFRSF11B* (cluster 6), *TMSB4X*, *CYTL1*, *LGALS1* (cluster 7), *COL2A1*, *COL11A1*, *SLPI*, *SPARC* (cluster 8), *CTGF*, *CYR61*, *COL1A2* (cluster 9), *PPP3CA*, *CTNNB1*, *PPP1CB* (cluster 10), *DDIT3*, *HSPA5*, *NUPR1* (cluster 11), *MMP3*, *COMP*, *CHI3L2* (cluster 12), and *HIST1H4C*, *TUBA1B*, *TMSB4X* (cluster 13) (Figure 1C). Significantly DEGs were defined as a log fold change >0.25 and *p* < 5.4 × 10^−24^. The most significant DEGs in the clusters (0–13) were *SOD2* (LogFC = 0.42), *FGFBP2* (LogFC = 0.79), *MT1G* (LogFC = 1.69), *CXCL8* (LogFC = 1.46), *CTGF* (LogFC = 1.58), *FRZB* (LogFC = 1.56), *MSMP* (LogFC = 1.74), *TMSB4X* (LogFC = 1.72), *COL2A1* (LogFC = 1.48), *CTGF* (LogFC = 2.36), *PPP3CA* (LogFC = 1.16), *DDIT3* (LogFC = 1.01), MMP3 (LogFC = 1.52) and HIST1H4C (LogFC = 2.38) (Table S2 and Supplemental Figure S2). Taken together, these results reveal the overall pattern of transcriptomic profiles of human NP and iAF cells at the single-cell level.

### 2.2. Identification of Distinct Cell Populations in NP and iAF of Non-Degenerating and Degenerating Discs

**Figure 2:**
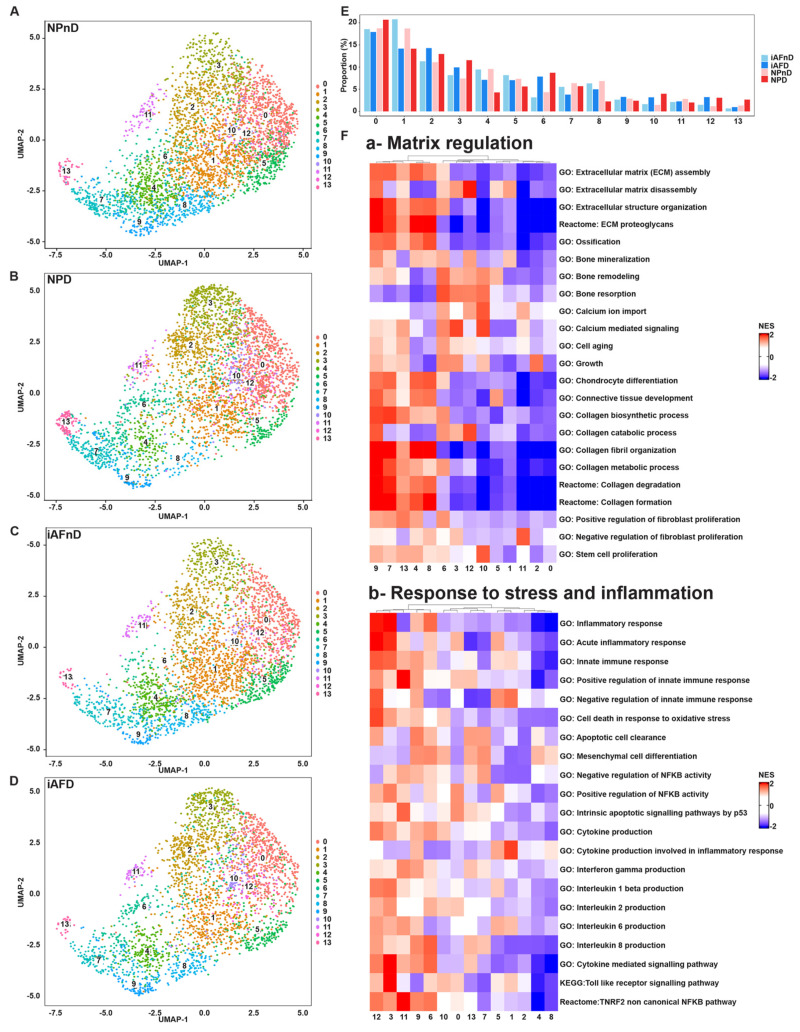

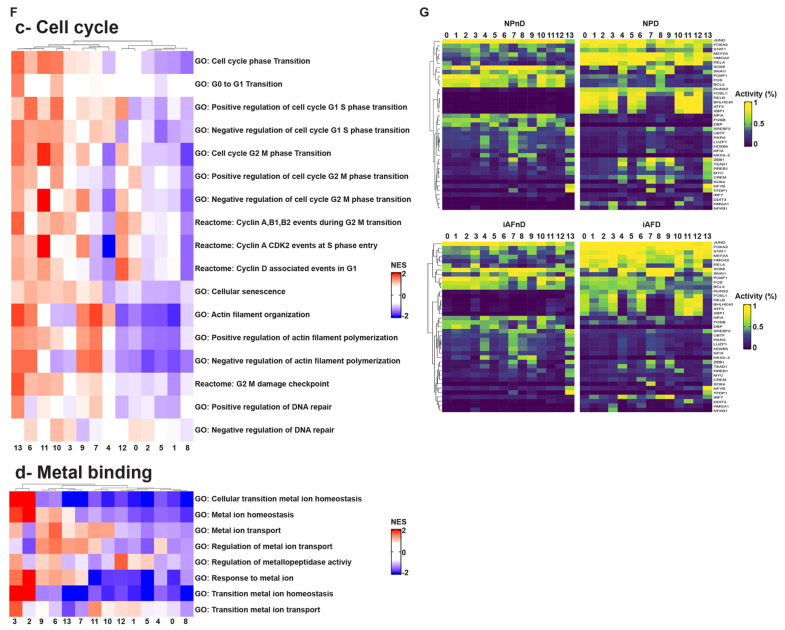
Single-cell RNA profiling and transcriptional changes correlated with cell type and IVD degeneration. Unsupervised UMAP clustering showing the change in cell distribution of the 14 different clusters for (**A**) NPnD, (**B**) NPD, (**C**) iAFnD and (**D**) iAFD. (**E**) Variations in cell proportion of the 14 different clusters between the samples. (**F**) QuSAGE analysis of cell population specific differential expression colored by statistically significant normalized enrichment scores. The main discovered IVD cell functions were presented in heatmaps in (**a**) matrix regulation, (**b**) response to stress and inflammation, (**c**) cell cycle and (**d**) metal binding. (**G**) Heatmap showing grade-related TFs in the identified 14 cell clusters.

We next analyzed how the number of cells assigned to each cluster varied with degeneration and IVD region. This analysis determines the number of cells present in each cluster and indicates if the variation is cell-type specific or linked to IVD degeneration. The analysis did not detect any pure region-specific clusters. However, clusters 6, 7, 11 and 13 contained 13.05–49.24% more cells in tissue from NPnD compared to tissue from iAFnD. We observed a similar increase in cell numbers when comparing iAFD to NPD in clusters 5, 8 and 9 (20.28–54.81%) and in NPD clusters 0, 3, 6–7, 10 and 13 (10.94–174.22%) when comparing to AFD. Clusters 0, 2, 3, 6, 10, 12 and 13 contained 9.33% to 63.56% more cells in tissue from NPD tissue compared to the number from NPnD tissue. For iAFD tissue, clusters 2–3, 6 and 9–13 contained 6.63–59.79% more cells than tissue from iAFnD. A 3.44% to 31.84% lower number of cells was found in clusters 0–1, 4–5 and 7–8 of iAFD tissue compared to iAFnD tissue. Finally, the number of cells in clusters 1, 4–5, 7–9 and 11 was 10.69–67.05% lower in NPD compared to NPnD (Figure 2A–E and Tables S3–S6). 

The function of the clusters and gene-set activity can be predicted based on published papers applying IVD cells to single-cell sequencing [48,49,54,55,56] and using Quantitative Set Analysis for Gene Expression (QuSAGE) [57]. Pathway enrichment analysis allowed us to classify the cell clusters into four major groups. Group 1 with clusters 1 and 4–9 is implicated in matrix regulation, showing higher activation of ECM regulation, connective tissue development, calcium regulation and collagen biogenesis pathways (Figure 2F(a)). Group 2 with clusters 3, 6, 9 and 11–12 is implicated in response to stress and inflammation, including the pathways, acute inflammatory response, regulation of innate immune response and toll-like receptor signaling (Figure 2F(b)). Group 3 with clusters 6, 10–11 and 13 showed an activation of pathways involved in cell-cycle regulation, senescence, cell-cycle G1S and G2M phase transition and cyclin-associated events (Figure 2F(c)). Group 4 with clusters 2 and 3 is implicated in metal ion binding and metal ion homeostasis and transition pathways (Figure 2F(d)).

It is important to highlight that IVD cells within a single cluster can to a minor extent contribute to functions in more than one of the four groups. Cell clusters contributing to only 1 of the 4 groups are 1, 2, 4, 5, 7, 8, 10 and 13. However, clusters 3, 6, 9, 11 and 12 contribute to pathways in more than one group. The main function of cluster 3 is in metal binding, but it is also implicated in response to stress and inflammation. Cluster 6 is mainly implicated in cell-cycle regulation and is also implicated in activation of matrix regulation, and in response to stress and inflammation. The main function of cluster 9 is in matrix regulation, but it is also implicated in response to stress and inflammation. Cluster 11 has a main function in cell-cycle regulation and is also implicated in response to stress and inflammation. Cluster 12’s main function is in response to stress and inflammation, although it is also implicated in matrix regulation (Figure 2F(a–d) and Table S7). We further linked transcription factor expression (TF) to the identified cell populations. Most cell clusters in NPnD and iAFnD showed high activation of JUND, SOX8, FOXF1 and no activation for DDIT3, HMGA1 and NFKB1. The top enriched TFs linked to degeneration were FOXA3, STAT1, MEF2A, HMGA2 and RELA. Of interest, we observed an opposite TF enrichment with degeneration in NP and iAF cells. Clusters 0–3, 5–6 and 10–12 showed higher expression of RUNX2, FOSL1, RELB, BHLHE40, ATF30 and XBP1 in iAFD and NPD compared with iAFnD and NPnD, respectively. The similar pattern of TF activation indicates common pathways implicated in IVD degeneration in both cell types (Figure 2G). 

### 2.3. Intervertebral Disc Cell Markers

**Figure 3:**
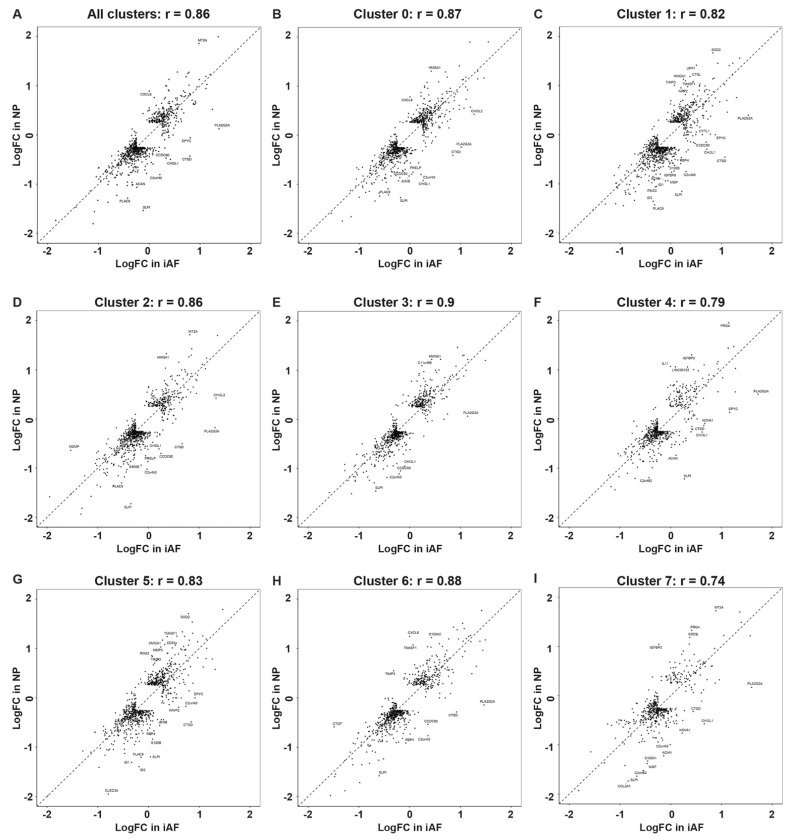

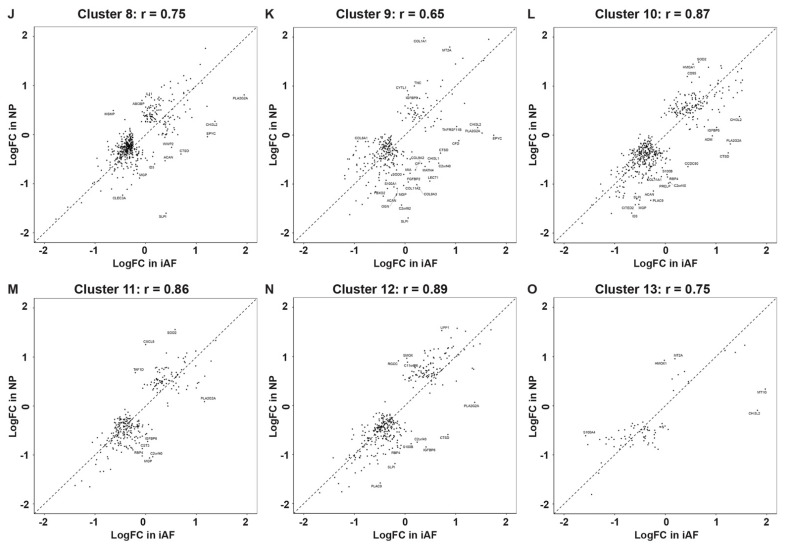
Correlation between changes in gene expression in iAF and NP cells from total RNA and per cluster. Scatter plots showing correlational analysis between DEGs in iAF and NP cells (**A**) all together and (**B**–**O**) in each of the 14 clusters (0–13). Pearson correlation coefficients (r) between iAF and NP were calculated using the log fold change (logFC) values of genes that are differentially expressed in cell type.

To study the differences between NP and iAF cells, we compared DEGs in each cluster. The majority of DEGs (695/719 genes in total) were shared between the two cell types with a Pearson correlation r = 0.86 (Figure 3A). However, some DEGs showed opposite expression in the two cell types. For example, genes related to senescence and oxidative stress (*C2orf40*, *MT1F* and *HIF-1α*) and extracellular matrix regulation (*PRELP*, *EPYC* and *CHI3L1*) were expressed at a significantly higher level in iAF cells. Opposite and distinct transcript expression with a higher expression in NP compared to iAF was observed for genes associated with initiation of mRNA translation (*EIF1*), cell-cycle progression (*RGCC*) and pathophysiology of arthritis (*LGALS1*). Moreover, comparison of NP and iAF cells showed that some genes present a similar trend but with a higher expression in one cell type compared to the other. As an example, a lower *MGP* gene expression was pronounced in NP (logFC.NP = −1.03 and logFC.iAF = −0.43), while a higher expression of *PLA2GA* was pronounced in AF (logFC.NP = 0.12 and logFC.iAF = 1.37) (Tables S8 and S9). Since the expression profile of all cells in a tissue sample is an aggregate of the profiles in the populations present, it might not be reflected in individual clusters. Correlational analysis between DEGs in NP and iAF cell in each of the 14 clusters (0–13) revealed positive correlations in all clusters (Pearson correlations ranging from r = 0.65–0.9) (Figure 3B–O), indicating an overall similar transcriptional profile in NP and iAF cells within the identified clusters. Significantly, DEGs between NP and iAF were, however, found in clusters 0–11 while none were found in clusters 12 and 13. The list of genes generated with the highest expression level in NP compared with iAF were specific to clusters 0–5 and include *MGP*, *MSMP*, *CHI3L1*, *C20rf40*, *ID1*, *ID3* and *TMED2*. These genes are associated with functions in matrix deposition, cytokine expression, cell growth, proliferation and senescence [58,59,60,61]. Interestingly, we found that mitochondrial and oxidative stress specific genes *MT-ND1-4*, *MT-CO1-3*, *MT-CYB*, *MT-ATP6*, *SOD2*, *BNIP3*, *HIF1a* and *PGK1* were lower in clusters 0–5, 7–9 and 11 of NP cells. Cell matrix genes *COL2A1*, *COL3A1*, *COL6A2*, *COL9A2*, *COL9A3*, *COL11A1*, *ACTG1*, *SPARC*, *OGN*, *SCRG1*, stimulators of chondrogenesis, *LECT1* and *CTGF* also showed a lower expression in NP cells in clusters 2, 4 and 5 as mentioned above, whereas cytokines and genes related to inflammation, *CP*, *MMP3*, *MT1G*, *MT1X*, *MT1E*, *MIF* and *LCN2* were lower in NP cells only in clusters 0 to 5. Only one DEG was found in cluster 6, *ENO1*, involved in processes such as growth control, hypoxia tolerance and allergic responses [62,63,64] and in cluster 10, *EIF4B*, required for mRNA binding to ribosomes [65] (Figure 3B–O and Table S7). All together, these findings suggest novel DEGs that could serve as potential NP and iAF cell-biomarker panels.

### 2.4. Cell Type Specific and Common Intervertebral Disc Degeneration Markers

**Figure 4:**
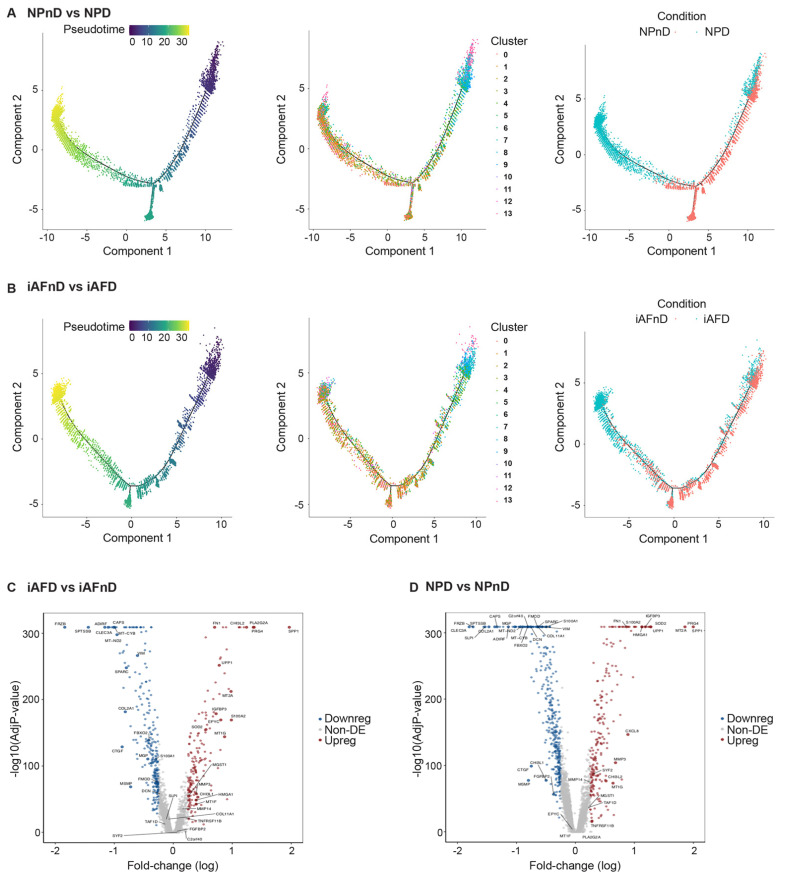
DEGs in iAF and NP cells from nD and D discs. Monocle method reconstruction of pseudotime trajectory axis and pseudospace trajectory for (**A**) NPnD vs. NPD and (**B**) iAFnD vs. iAFD defined cell clusters along the progression of IVD degeneration. Volcano plot depicting differentially expressed genes in D compared with nD (**C**) iAF and (**D**) NP cells. Red, grey and blue circles represent upregulated, non-differentially expressed and downregulated genes, respectively.

Monocle is a single-cell trajectory analysis that orders whole-transcriptomic profiles of single cells along an artificial temporal curve (pseudotime axis) [66]. Monocle trajectory analysis was used to characterize processes of degeneration by resolving the spatial organization of different subpopulations in iAFnD, NPnD, iAFD and NPD (Figure 4A,B). Pseudotime trajectory of both NP and iAF clusters showed a branched (bifurcated) trajectory. NP clusters (4–9 and 13) were dispersed at the start and the root of the trajectory, and clusters 0–3 and 10–13 were distributed in the middle and later periods. Pseudotime trajectory condition, showing the dispersion of NPnD and NPD cells, revealed a homology between clusters 4–9 and 13 and nD subpopulations. NPD clusters 0–3 and 10–13 were distributed at the same period of the trajectory (Figure 4A). In iAFnD, cells in clusters 0–2 and 10–13 were mainly distributed at the end of the trajectory. Additionally, in iAFD subpopulations linked to degeneration existed mainly at the start of the trajectory, mimicking the distribution of clusters 3–9 (Figure 4B). These findings highlight the heterogeneity and role of clusters in the progression and pathogenesis of IVD degeneration. The similar pattern of the identified clusters and their respective degenerative state in NPD and iAFD suggests a potential role of these clusters in the process of IVD degeneration. 

The UMAP learning technique for dimension reduction was used to identify expression patterns of genes strongly linked to IVD degeneration (Tables S12 and S13). Differences in gene expression in iAF and NP cells of nD (iAFnD, NPnD) and D (iAFD, NPD) discs is visualized in the volcano plots (Figure 4C,D). We identified a group of genes linked to degeneration common between the two cell types and a group of genes showing specific expression in the cell types. Genes including *MT1G*, *SPP1*, *HMGA1*, *FN1*, *UPP1*, *S100A2*, *PRG4*, *SOD2* and *MT2A*, which are mainly involved in cellular responses to stress, skeletal system development, extracellular matrix organisation, collagen catabolism and inflammation were highly expressed in both cell types from D compared with nD tissue. In addition, mitochondrial genes associated with responses to oxidative stress (*MT-CYB*, *MT-ND2*), genes involved in extracellular matrix organisation (*SPARC*, *VIM*, *CTGF*) and genes with roles in inflammatory responses, ions transport and in preventing tissue damage by limiting protease activity (*SPTSSB*, *S100A1*, *MGP* and *DCN*) [67,68] showed a similar expression decrease in iAFD and NPD when compared to iAFnD and NPnD, suggesting a common change that occurs during IVD degeneration. Several genes including *MGST1*, *PLA2G2A*, *MT1F*, *EPYC* and *CHI3L1* showed higher expression only in iAF cells with degeneration, while *C2orf40*, *SLPI* and *COL11A1* showed lower expression only in NPD cells. Finally, we observed a specific increase in the expression of *SYF2* (*p29*) for NPD and a decrease in TAF1D for iAFD when compared, respectively, with NPnD and AFnD (Figure 4C,D). Together, this provides a comprehensive picture of IVD cell heterogeneity, in D and nD tissue.

### 2.5. Gene Ontology (GO) and Kyoto Encyclopedia of Genes and Genomes (KEGG) Analysis Revealed Enriched Pathways in the Identified Cell Subpopulations

**Figure 5:**
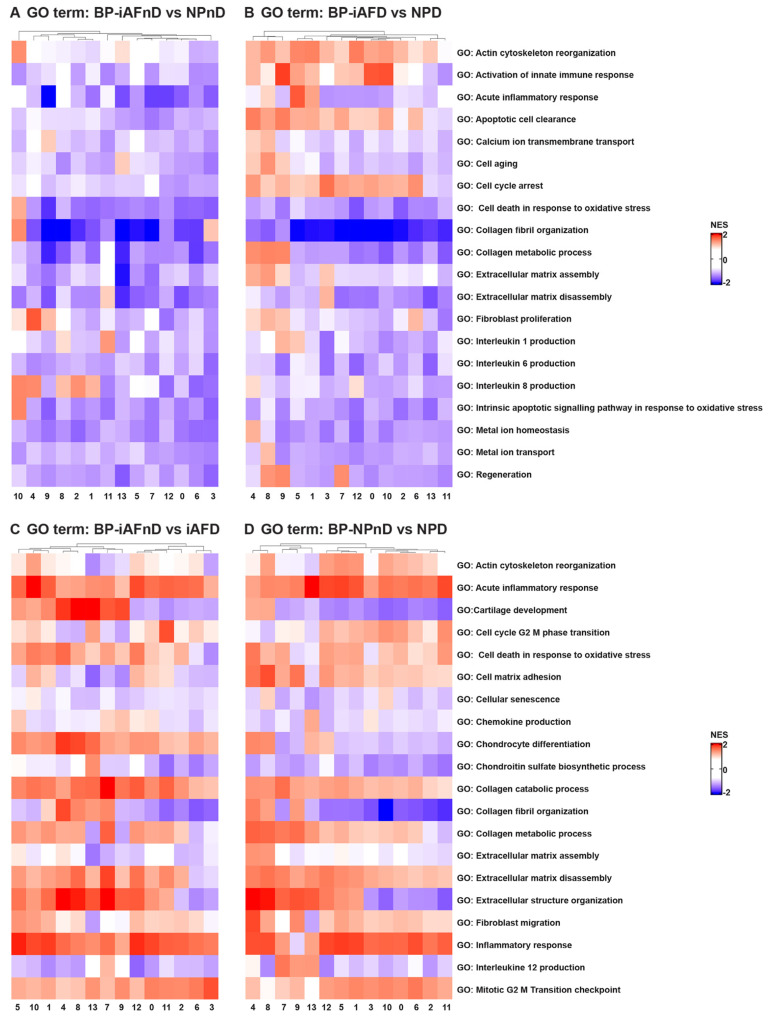

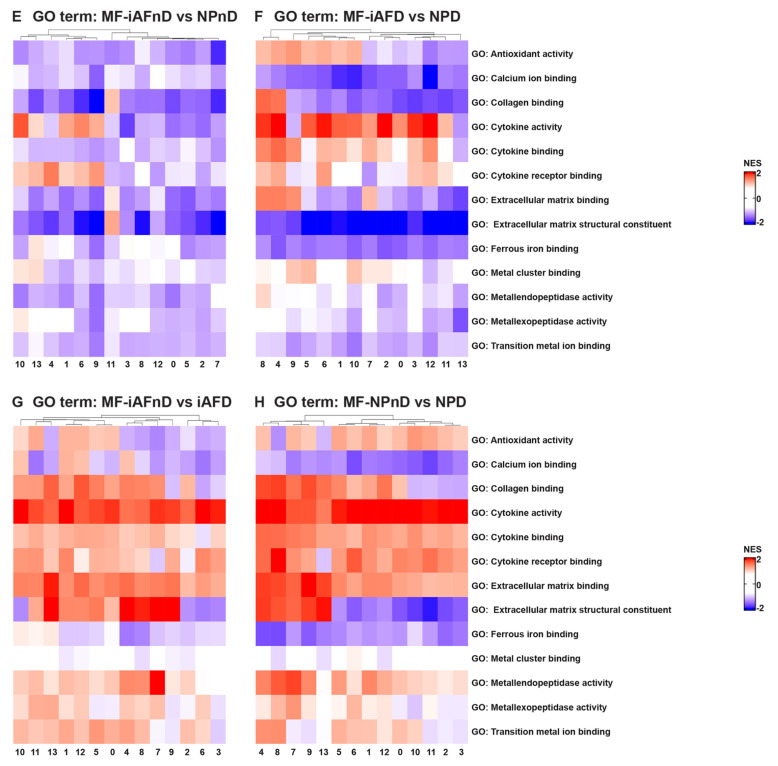

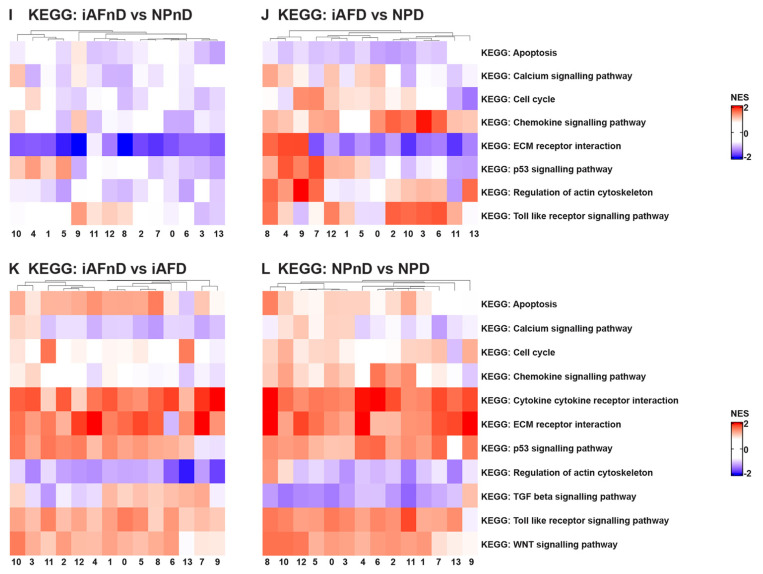
GO term and KEGG analysis of DEGs between NP and iAF cells from nD and D IVDs. Heatmap representations depicting the differences in affected biological processes (**A**–**D**), molecular functions (**E**–**H**) and KEGG (**I**–**L**) pathways per cluster (0–13) in iAFnD vs. NPnD (**A**,**E**,**I**), iAFD vs. NPD (**B**,**F**,**J**), iAFnD vs. iAFD (**C**,**G**,**K**) and NPnD vs. NPD (**D**,**H**,**L**) cell populations. Upregulated genes in each cluster were used for the analysis. Complete GO terms and KEGG lists are shown in the Supplemental Tables S14–S22.

Understanding intercellular networks of communication can help elucidate potential targets for therapy in a cell-type-specific manner. ScRNA-seq provides a unique starting point for deciphering molecular functions and biological process interactions according to IVD degeneration status and cell type. To investigate the molecular functions and the biological processes of the identified cell clusters, we performed GO [69] and KEGG [70] analysis. In our study, we identified DEGs that showed a significant abundance change (Fc >0.25 and *p* < 5.4 × 10^−24^) in NP and iAF cells from D compared to nD discs. We carried out an enrichment analysis for Gene Ontology (with an FDR of 0.02). The most interesting differences in biological process between NP and iAF cells from nD discs were found in clusters 10 and 4, showing higher activation in NPnD of actin cytoskeleton and collagen fibril organization, cell death and intrinsic signalling pathways in response to oxidative stress, interleukin 8 production and fibroblasts’ proliferation pathways (Figure 5A and Table S18). In degenerating tissue, higher activity in NPD was observed in clusters 0, 9 and 12 for innate immune response activation and in clusters 4 and 7–9 for collagen metabolic processes, apoptotic cell clearance and regeneration processes (Figure 5B and Table S18). Molecular functions of cytokine activity, cytokine receptor binding and metal cluster binding were enriched in clusters 1, 4, 6, 9–10 and 13 in iAFnD compared to NPnD (Figure 5E and Table S19), while antioxidant activity, collagen and matrix binding molecular functions were higher in NPD clusters 1, 4–6 and 8–10 (Figure 5F and Table S19). KEGG analysis between NPnD and iAFnD cells showed a significant enrichment of p53 (1, 4, 5 and 10 clusters) and toll-like receptor (8–9 and 11–12 clusters) signalling pathways in NPnD (Figure 5I and Table S22). When we compared iAFD to NPD we observed an upregulation of ECM receptor interactions, cell cycle and calcium regulation pathways in clusters 4, 7–9 and higher activation of chemokine and toll-like receptor signalling pathways in clusters 2–3, 6, 8 and 10–12 of NPD cells (Figure 5J and Table S22).

In addition, biological processes identified as enriched with degeneration for both cell types and in all clusters were acute inflammatory response, collagen catabolic processes and ECM disassembly, while processes such as collagen fibril organization, chondroitin sulfate biosynthetic processes and ECM assembly were downregulated in most cell clusters (Figure 5C,D). These fall mainly within an increase with degeneration in the molecular functions of antioxidant and cytokines activity, ECM and cytokine binding (Figure 5G,H). KEGG analysis was performed to show the function of identified DEGs between the nD and D groups. Interestingly, cytokine–cytokine and ECM receptor interaction, p53, WNT and toll-like receptor pathways were enriched mainly in iAFD (Figure 5K and Table S20) and NPD (Figure 5L and Table S21) compared to iAFnD and NPnD cell clusters. A detailed description of the 10 first GO terms with a positive correlation to IVD degeneration is expressed for each cluster and in each cell type, iAF in Tables S14 and S15, and NP cells in Tables S16 and S17. In this analysis, the identified biological processes and molecular functions are directly related to cell-cycle regulation, cytokine secretion and extracellular matrix synthesis, and are relevant to the progression of IVD degeneration. All together, these results revealed the major pathways involved in the pathogenesis of disc degeneration.

### 2.6. Quantitative Assessment Using RT-qPCR of Genes Identified by scRNA-Seq

**Figure 6:**
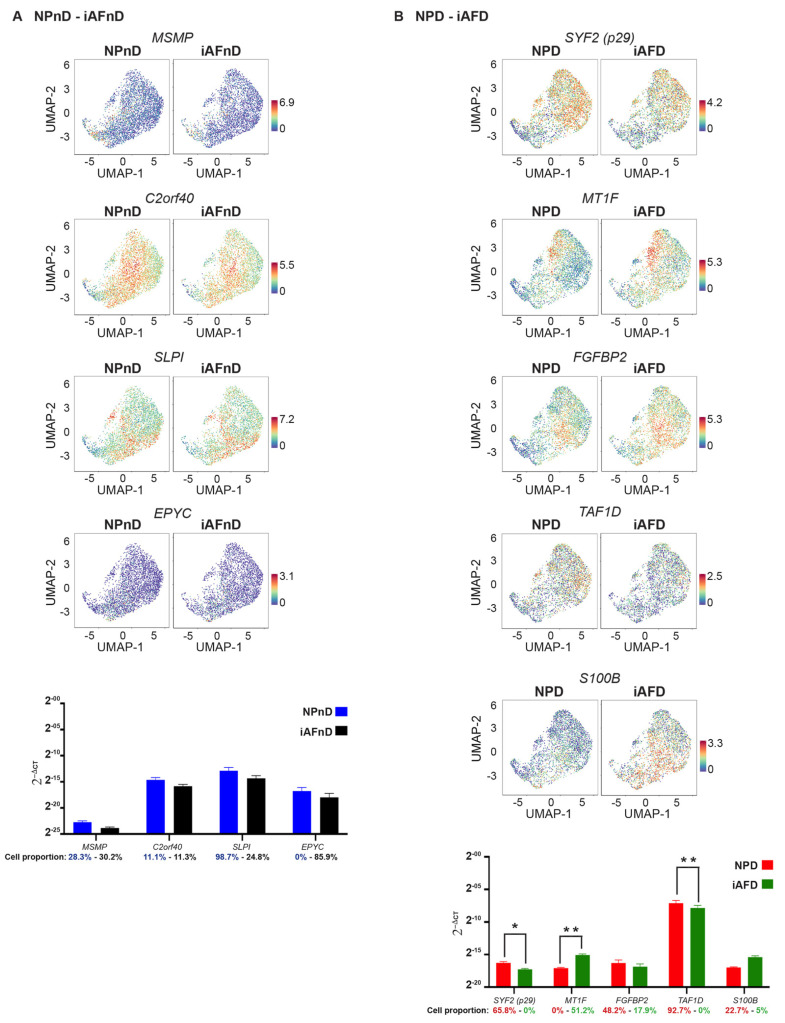

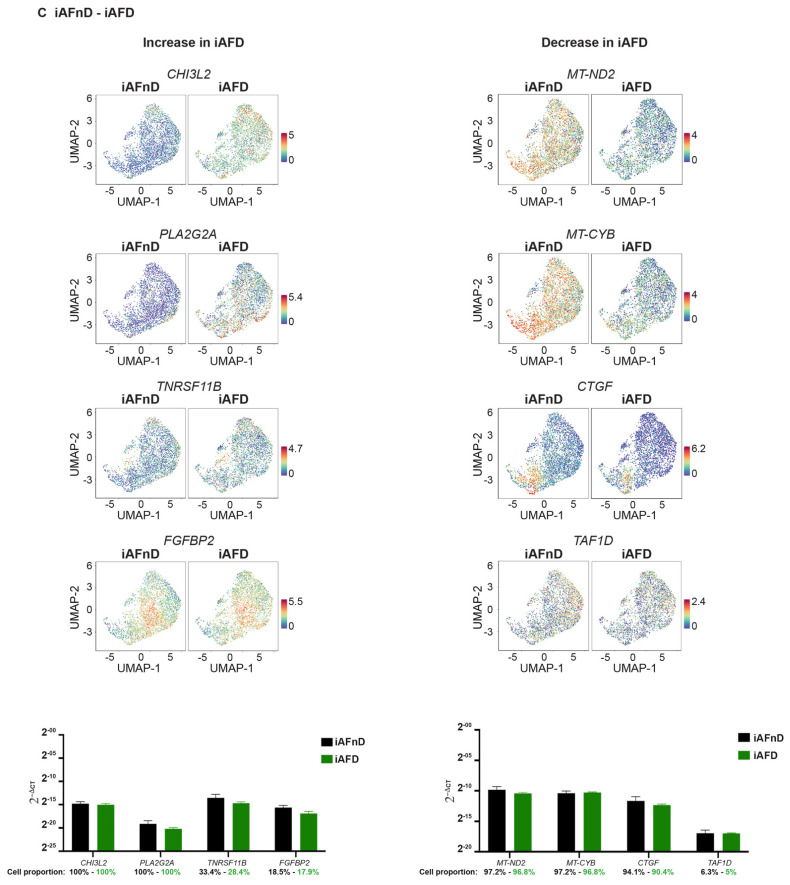

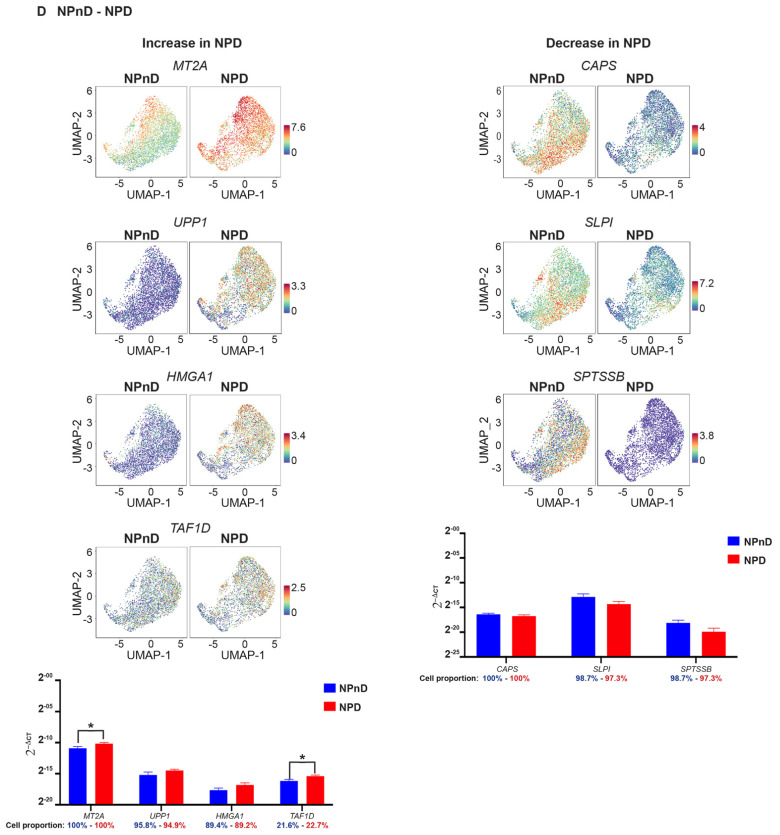

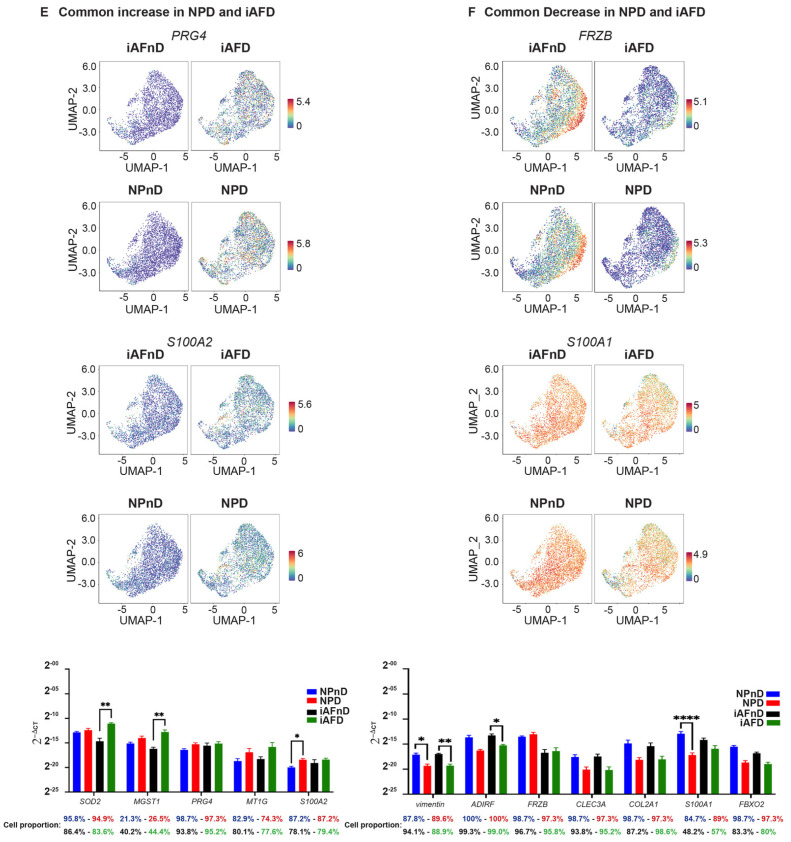
RT-PCR validation of DEGs identified from transcriptome analysis. Representative UMAPs and graphs highlighting the gene expression differences of *MSMP*, *C2orf40*, *SLPI* and *EPYC* between NPnD and iAFnD in (**A**); *SYF2*, *MT1F*, *FGFBP2*, *TAF1D* and *EPYC* between NPD and iAFD in (**B**). RT-PCR validation of IVD degeneration selected DEGs in the transcriptome data of *CHI3L2*, *PLA2G2A*, *TNRSF11B* and *FGFBP2* showing an increase or *MT-ND2*, *MT-CYB*, *CTGF* and *TAF1D* showing a decrease in iAFD (**C**) when compared to iAFnD cells from discs of the same individual. mRNA expression of *MT2A*, *UPP1*, *HMGA1* and *TAF1D* (increase) and *CAPS*, *SLPI* and *SPTSSB* (decrease) in NPD compared with NPnD (**D**). Validation of common degeneration markers by RT-qPCR showing an increase or a decrease with degeneration NPD (**E**) and AFD (**F**). The values were calculated using the 2^−∆∆Ct^ method: * *p* < 0.05, ** *p* < 0.01 and **** *p* < 0.0001.

Bulk RT-qPCR allowed us to evaluate the capacity of cluster-specific genes to translate in bulk RNA content of five separate individuals. We compared the expression of a selected list of genes according to their pattern of expression and their relevance to IVD degeneration, oxidative stress and cell senescence. Thus, we selected 33 genes as potential IVD cell biomarkers, and we classified them according to their expression at the single-cell level. To validate the observed differences between NPnD and AFnD, we verified mRNA expression of *MSMP*, *C2orf40*, *SLPI* and *EPYC* genes. *SYF2* *(p29)*, *MT1F FGFBP2*, *S100B* and *TAF1D* were used to confirm the changes between NPD and AFD. The RT-qPCR results for *CHI3L2*, *PLA2G2A*, *TNRSF11B*, *FGFBP2*, *MT-ND2*, *MT-CYB*, *CTGF*, *MT2A*, *UPP1*, *HMGA1*, *TAF1D*, *CAPS*, *SLPI*, *SPTSSB*, *SOD2*, *MGST1*, *PRG4*, *MT1G*, *S100A2*, *VIM*, *ADIRF*, *FRZB*, *CLEC3A*, *COL2A1*, *S100A1* and *FBXO2* were used to evaluate potential biomarkers of IVD degeneration. The genes selected to determine a difference between iAFnD and NPnD cells were not significantly different in RT-qPCR (Figure 6A). However, mRNA expression of *SYF2* *(p29)*, *MT1F and TAF1D* reproduced a significant difference (increase or decrease) as observed with scRNA when we compared NPD to iAFD cells (Figure 6B).

In iAF cells, mRNA expression of *CHI3L2*, *PLA2G2A*, *TNRSF11B*, *FGFBP2*, *MT-ND2*, *MT-CYB*, *CTGF* and *TAF1D* in relation to degeneration failed to reproduce scRNA-seq findings (Figure 6C). When we compared NPD to NPnD, a significant increase in NPD was confirmed for *MT2A* and *TAF1D* and validated scRNA results, while significance was not reached for *UPP1*, *HMGA1*, *CAPS*, *SLPI* and *SPTSSB* (Figure 6D). In addition, mRNA expression of *SOD2*, *MGST*, *PRG4*, *MT1G* and *S100A2* showed a common trend of increase (Figure 6E) or decrease (*VIM*, *ADIRF*, *FRZB*, *CLEC3A*, *COL2A1*, *S100A1* and *FBXO2*) in expression when we compared iAFD and NPD to iAFnD and NPnD, respectively. Significance was reached for the common biomarkers *SOD2*, *MGST1*, *S100A2*, *VIM*, *ADIRF*, *S100A1* (Figure 6F). Cell proportion represents the percentage of cells expressing the gene of interest (Figure 6A–F).

To evaluate the effect that the number of clusters expressing a specific gene as observed by scRNA will have on translatability to RT-qPCR analysis, we grouped genes showing similar pattern of expression in all NP and iAF clusters (20 genes; Group 1) and genes with opposite expression in clusters of the two cell types (13 genes; Group 2) (Tables S24 and S25). Correlating the RT-qPCR results of the selected genes with cell type and the number of cell subpopulations expressing the genes indicated that RT-qPCR could validate 15.78% (3/19 genes) in NP and 14.28% (2/14 genes) in iAF cells of the genes having similar pattern of expression in all the 14 identified clusters. The genes showing opposite expression in the 14 clusters in NP and iAF cells by scRNA-seq could be confirmed in 28.57% (4/14 genes) for NP and 26.321% (5/19 genes) for iAF cells (Table S25).

### 2.7. Expression of Selected Markers Identified by scRNA-seq and Validated by RT-qPCR at the Protein Level

**Figure 7:**
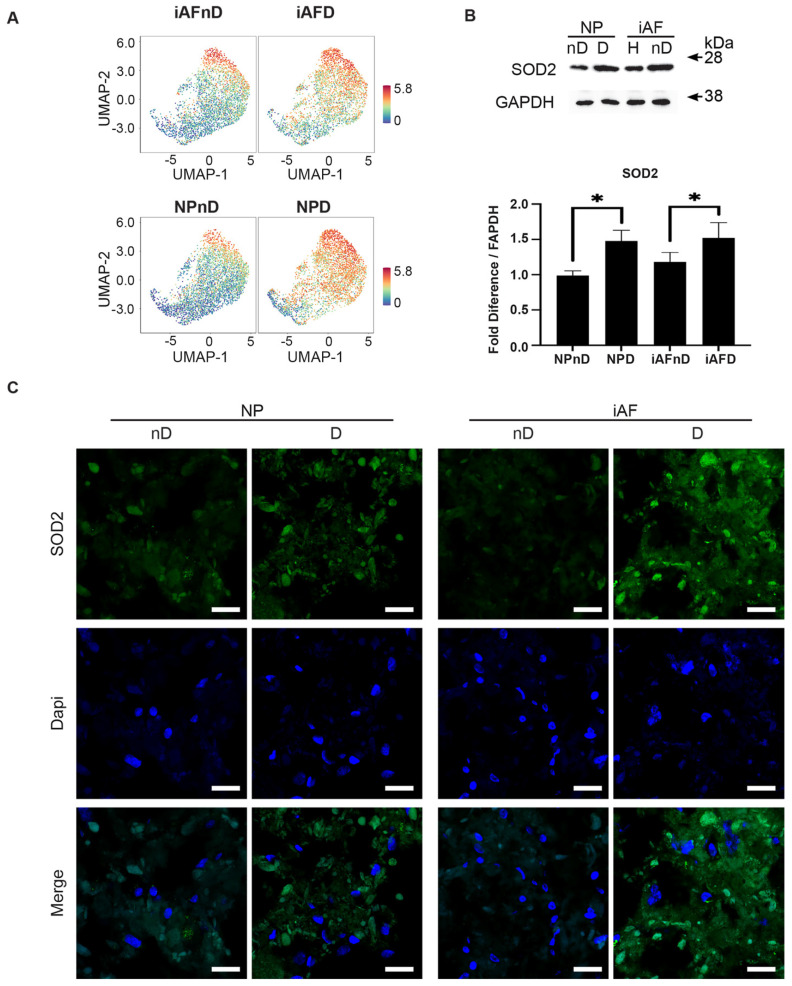
Validation of selected cell type and degeneration markers. (**A**) UMAP highlighting *SOD2* gene expression at the single-cell level in iAF and NP nD and D discs of the same individual. (**B**) Western Blot analysis of cell lysates from 5 additional individuals normalized to GAPDH (n = 5). (**C**) Immunohistochemistry staining of SOD2 (in green), Dapi (in blue) and merged images of cells from 5 additional individuals (scale = 100 µm, n = 5 biological replicates). Graphs are presented as mean ± SEM. * *p* < 0.05.

**Figure 8:**
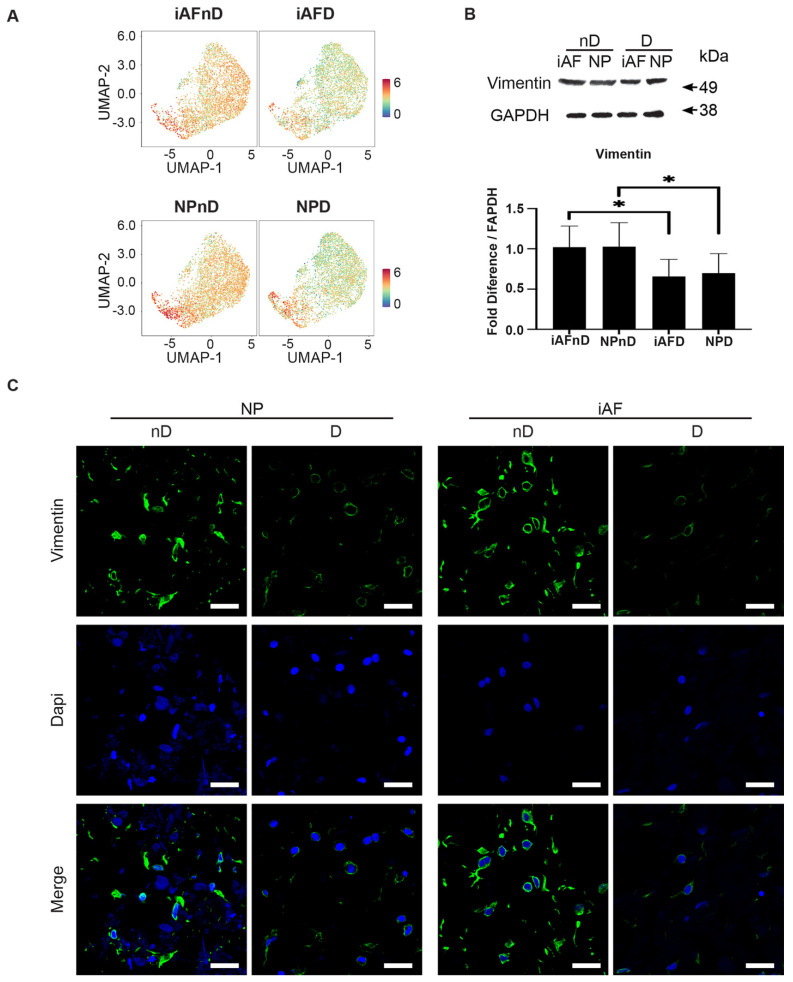
Validation of selected cell type and degeneration markers. (**A**) UMAP highlighting *vimentin* gene expression at the single-cell level in iAF and NP nD and D discs of the same individual. (**B**) Western Blot analysis of cell lysates from 5 additional individuals normalized to GAPDH (n = 5). (**C**) Immunohistochemistry staining of vimentin (in green), Dapi (in blue) and merged images of cells from 5 additional individuals (scale = 100 µm, n = 5 biological replicates). Graphs are presented as mean ± SEM. * *p* < 0.05.

**Figure 9:**
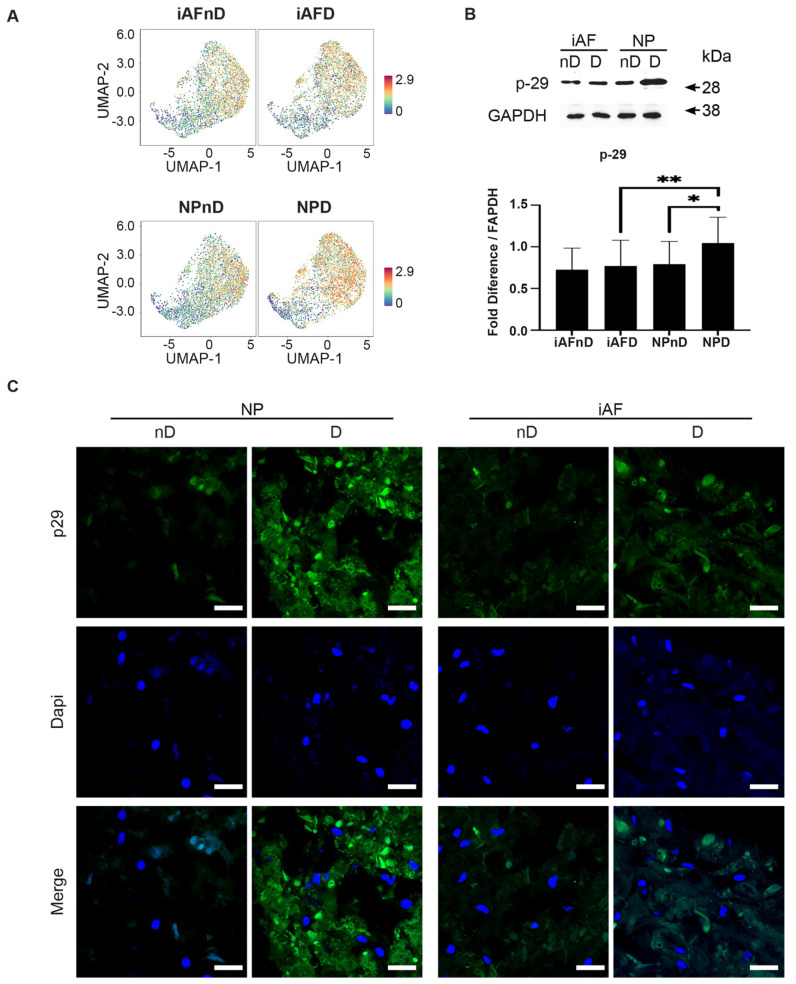
Validation of selected cell type and degeneration markers. (**A**) UMAP highlighting p29 gene expression at the single-cell level in iAF and NP nD and D discs of the same individual. (**B**) Western Blot analysis of cell lysates from 5 additional individuals normalized to GAPDH (n = 5). (**C**) Immunohistochemistry staining of p29 (in green), Dapi (in blue) and merged images of cells from 5 additional individuals (scale = 100 µm, n = 5 biological replicates). Graphs are presented as mean ± SEM. * *p* < 0.05, ** *p* < 0.01.

**Figure 10:**
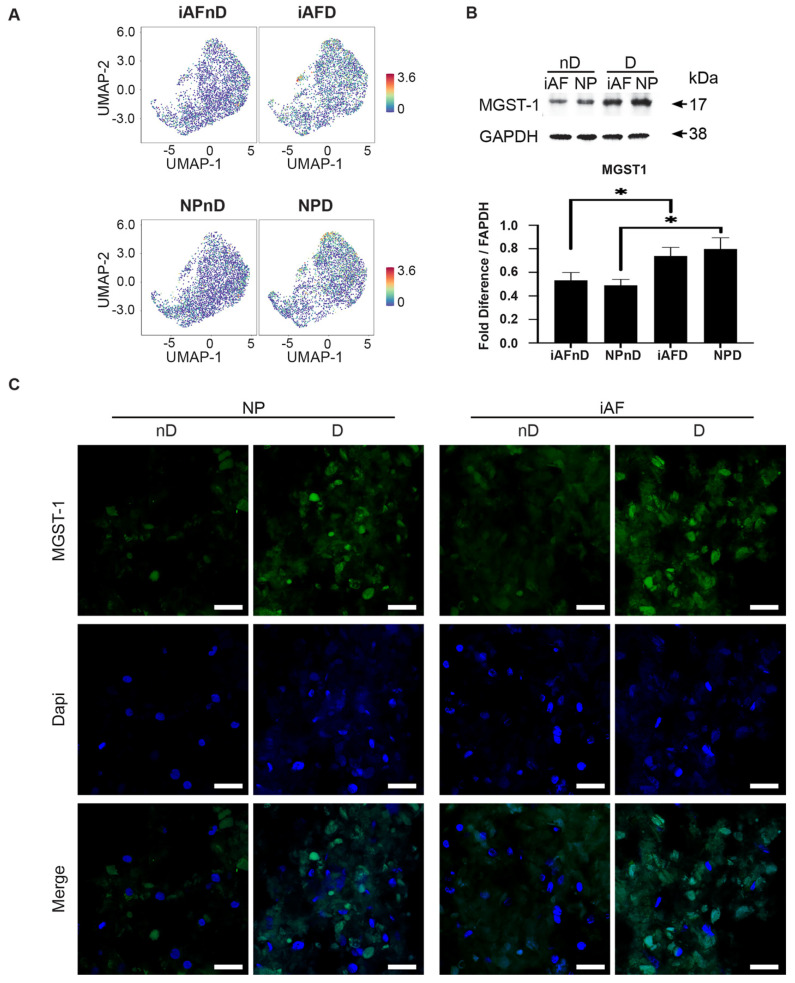
Validation of selected cell type and degeneration markers. (**A**) UMAP highlighting MGST-1 gene expression at the single cell level in iAF and NP nD and D discs of the same individual. (**B**) Western Blot analysis of cell lysates from 5 additional individuals normalized to GAPDH (n = 5). (**C**) Immunohistochemistry staining of MGST-1 (in green), Dapi (in blue) and merged images of cells from 5 additional individuals (scale = 100 µm, n = 5 biological replicates). Graphs are presented as mean ± SEM. * *p* < 0.05.

To further validate the transcriptional changes revealed by scRNA-seq analysis, we performed quantitative immunofluorescence and Western blotting for selected proteins including *SOD2*, *vimentin*, *p29 (SYF2)* and *MGST* (Figure 7–10). We surveyed their expression on histological sections of NP and iAF cell pellets following 21 days of culture to investigate stability and validity of the observed transcriptional changes. Representative gene(s) from RT-qPCR were selected: Group 1 (*vimentin*, *SYF2* (*p29)* and *MGST-1*) and Group 2 (*SOD2*).

Examination of UMAP visualisation confirmed that expression of the antioxidant enzyme located in the mitochondrial matrix *SOD2* is enriched in cells of degenerating NP and iAF discs. (Figure 7A. Western blotting and quantification of SOD2 showed a 0.23-fold (*p* < 0.05) and 0.34-fold (*p* < 0.05) higher expression in iAFD and NPD cells, respectively, compared to cells from nD IVDs (Figure 7B). Interestingly and consistent with our scRNA-sequencing analysis and Western Blot results, representative immunohistochemistry images of SOD2 showed a qualitative increase in both NPD and iAFD cells when compared to cells from nD tissue (Figure 7C). 

Based on previous *vimentin* expression in human IVDs, we postulated that the UMAP plot would be enriched for *vimentin* specific to NPnD and iAFnD cells compared with cells from degenerating discs [71,72], which we confirmed (Figure 8A). Furthermore, the lower expression related to degeneration was also reflected by the immunoblot result of vimentin. Quantification of the signal intensity demonstrated a significantly lower vimentin expression (–0.36- fold relative intensity, *p* < 0.05) in iAFnD cells relative to iAFD cells and in NPnD cells compared with NPD cells (−0.32-fold relative intensity, *p* < *0*.05) (Figure 8B). Finally, representative immunohistochemistry images of vimentin confirmed lower levels in relation to degeneration in NP and iAF cells (Figure 8C). 

UMAP visualisation revealed that expression of the *p29* gene is specifically enriched in NPD cells. (Figure 9A). Western blotting and quantification of p29 showed a 0.24-fold (*p* < 0.05) and 0.26-fold (*p* < 0.05) higher expression in NPD cells compared with NPnD and iAFD, respectively. No significant change was observed between NP and iAF cells from nD discs. (Figure 9B). Consistent with our scRNA-seq analysis and Western blot results, representative photomicrographs of p29 staining show qualitatively higher expression in NPD cells when compared either to NPnD or iAFD cells (Figure 9C). 

Although there is no clear enrichment in the UMAP plot, *MGST1* has previously been identified as a hub gene in the pathological process of osteoarthritis [73], and the mRNA has been shown to be highly expressed in human iAF cells from degenerating IVDs [74]. Here, immunoblot results showed a significant difference at the protein level between cells from nD and D discs (Figure 10A). The iAF cells from degenerating IVDs had a 0.28-fold higher relative intensity, (*p* < 0.05) than cells from non-degenerating IVDs, whereas a 0.38-fold higher relative intensity (*p* < *0*.05) was observed in NP cells of nD compared to D tissue, (Figure 10B). Finally, representative immunohistochemistry images of MGST1 showed a higher signal in concert with degeneration in NP and iAF cells with similar expression between the two cell types, which is consistent with our scRNA-seq findings (Figure 10C). Together, the validation of selected genes confirmed that scRNA-seq can generate results that are quantitatively stable and reproductible at the mRNA and protein-level but must be validated for each candidate. 

## 3. Discussion

Although there are very promising cell-based regenerative therapy and ongoing clinical trials to delay or regenerate degenerating IVDs [75,76,77,78], a more profound understanding of the pathophysiology and cell types involved is required [79]. In this study, scRNA-seq was applied to cells from D (Thompson Grade III–V) and non-degenerating (Thompson Grade I–II) discs of the same individual [80]. The deep transcriptomic data from >13,500 individual cells provided a comprehensive resource for the understanding and multidimensional characterization of the cells. The focus and strength of our analysis were the exploration of NP and iAF cells as a source of biomarkers with a potential to contribute to IVD degeneration. To avoid inter-donor variability observed in genome-wide association studies or other large-scale evaluations based on cell pooling and population averaging technologies [33,43,44,45,46,47,48,49,81], the present study provides the first scRNA-seq database comparison between cells of non-degenerating and degenerating human discs from the same individual. Validation of scRNA-seq expression profiling highlights potential markers that can be identified also by bulk RT-qPCR, immunohistochemistry and Western Blot. 

### 3.1. Cell Clusters in NP and iAF Tissue

ScRNA-seq identified 14 putative cell subsets shared by non-degenerating and degenerating discs with distinct patterns of DEGs linked to cell type and degeneration. Previous studies were not performed using tissue obtained from the same human, but rather using animal tissue [17,28,38,39,82], monolayer expanded cultures [81], a group of different individuals [27,83] or surgically resected tissue with limited ability to discriminate between NP and iAF tissue [84,85]. Shortfalls in their use, for transcriptome-based analysis, were evident from interspecies differences in gene expression [40,84], cell phenotype changes following monolayer expansion, processing with the difficulty to clearly demarcate NP and iAF tissue in every patient sample and the underlying disease leading to surgery such as disc herniation and spondylolisthesis [43,86,87].

We reported transcriptomic heterogeneity and potential functional differences at the single-cell level between NP and iAF cells and between cells from degenerating and non-degenerating tissue. Among the clusters characterized, six shared a similar increase with degeneration in the percentage of cell proportion between NP and iAF, while five clusters showed a similar decrease in the number of cells in degenerating discs. These subpopulations showed significant expression of genes involved in matrix regulation, response to stress and inflammation, cell cycle and metal binding. Similar cell subpopulations were identified by scRNA-seq analysis in human NP cells [48,49,54,81] and in OA [55,88].

To quantitatively assign cellular identity in each cluster, we performed QuSAGE by correlating cluster transcriptome to GO and Reactome pathway databases. Cell clusters 4 and 7–9 were highly correlated with matrix regulation related pathways, while cell clusters 0–2, 5 and 11 showed negative regulation of the same pathways. The observed upregulation of cell cycle pathways in clusters 6 and 10–13 suggest that these populations may play a pivotal role in IVD cell senescence and apoptosis. These findings were in accordance with previous studies reporting a profound change in metabolic processes in disc cells linked to IVD degeneration [89,90]. Although no scRNA-seq and cell cluster annotation had previously been performed in iAF cells from degenerating and non-degenerating IVD tissue, our results are generally in agreement with previous studies of NP cells [48,49,54,55,56]. 

### 3.2. Differences between NP and iAF

Significantly, DEGs were found in NP and iAF cells in most clusters (0–11). We discovered potential biomarkers showing higher expression in NP cells such as *C2orf40*, *MGP*, *MSMP*, *CHI3L1*, *LGALS1*, *ID1*, *ID3* and *TMED*. *C2orf40* (also called esophageal cancer-related gene 4 (ECRG4)) encodes a secreted protein with a suggested role as a marker of differentiated articular chondrocytes and in cartilage destruction [91]. *MGP* is linked to abnormal calcium deposition in cartilage, *HILPDA* stimulates the expression of cytokines, and *MSMP* is a chemoattractant protein that may influence inflammation [58,60,61,92]. Furthermore, expression of several mitochondrial, oxidative stress, cell matrix and inflammation related genes such as *MT-ND2*, *MT-ND3*, *MT-ND4*, *MT-CO1*, *MT-CO2*, *MT-CO3 MT-CYB*, *SOD2*, *BNIP3*, *HIF-1α*, *PGK1*, *COL6A2*, *COL2A1*, *COL9A2*, *COL9A3*, *ACTG1*, *SPARC*, *OGN*, *SCRG1*, *LECT1*, *CTGF*, *PPIB*, *CP*, *MMP3*, *MT1G*, *MT1X*, *MT1E*, *MIF* and *LCN2* showed significantly lower NP-specific expression in the same cell clusters (0–11). *HIF-1α* and *CTGF* expression has previously been suggested to play a determinant role in NP cell phenotype and in the regulation of proteoglycan production [93,94,95]. Whereas mitochondrial genes (*MT-ND2* and *MT-CYB* as an example) are known for their roles in suppressing reactive oxygen species and reducing cell susceptibility to oxidative stress [73,74,96]. We observed higher gene expression in iAF cells of genes associated with senescence, oxidative stress and extracellular matrix regulation such as *MT1F*, *PLA2GA*, *EPYC*, *PRELP*, *C10orf10*, *FGFBP2* and *CHI3L1*. Metallothionin (*MT1F*), a gene that encodes a cytosolic protein product involved in the management of oxidative stress in articular cartilage, was reported to be highly expressed in the osteoarthritic cartilage and in cartilage from patients with necrosis of the femoral head [97,98]. *PRELP* and *CHI3L1* regulate synthesis and degradation of the ECM, and they promote chondrocyte survival and proliferation [99,100,101]. The identified genes presented in the complete list (Tables S9–S11) may serve as potential specific markers for iAF and NP phenotypes. 

### 3.3. Differences with Degeneration

The spatial distribution of the identified cell subpopulations in the NP and iAF cells using pseudotime analysis was in line with their identified functions and respective degeneration states. In fact, clusters responsible for cartilage development and regeneration, connective tissue growth and regulation of chondrogenesis, cellular calcium and bone development were mainly populating the start and the root of the trajectory, which is in accordance with a non-degenerating tissue-cell phenotype. However, cell subpopulations expressing genes related to stress response and inflammation, organization of the cytoskeleton and cell cycle regulation were distributed at the end of the trajectory corroborating with the phenotype of cells in degenerating tissues. 

Changes in gene expression related to IVD degeneration were observed in both cell types in IVDs of the same individual. An interesting finding was the variable expression of the same gene within clusters of NP and iAF cells in comparison to degeneration. Many genes displayed a similar expression pattern shared between NP and iAF cells. As an example, *vimentin*, *UPP1*, *ADIRF*, *FRZB* and *MT2A* exhibit a similar expression in the cell types, while *SOD2*, *C2ORF40*, *SLPI*, *CTGF*, *SYF2* and *MSMP* expression showed an opposite distribution pattern in the cell types (Table S24). A higher expression of genes involved in extracellular matrix organisation and collagen catabolism such as uridine phosphorylase protein 1 (*UPP1*) and human articular chondrocytes differentiation (*S100A2*) was observed, while genes including *SPTSSB*, *SLPI* and *CAPS*, playing roles in inflammatory responses and preventing tissue damage, had a lower expression [67,68,102]. *UPP1* expression was reported to be elevated in synovial fibroblasts in rheumatoid arthritis when compared with cells from individuals not suffering from rheumatoid arthritis specifically in hypoxia. In addition, the protein UPP1 altered vimentin distribution in fibroblasts from Alzheimer’s patients [103,104]. *SPTSSB* (serine palmitoyl transferase small subunit-b), which increased serine palmitoyltransferase isoenzymes affinity, was also associated with neurodegeneration [105]. Among the candidate genes in disc cells, *CD44* and Fibronectin1 (*FN1*) were found to be highly expressed in NP and iAF cells of degenerating IVDs with a higher expression in NP compared with iAF. The membrane receptors for hyaluronan *CD44* play important roles in cartilage homeostasis [106,107]. In fact, increasing *CD44* expression has previously been correlated with matrix synthesis in NP cells of degenerating IVDs [108]. *FN1* is a glycoprotein present at the cell surface and in the extracellular matrix that increases with disc degeneration [109]. The expected high expression of *CD44* and *FN1* was observed in most of the clusters (9–14) in both cell types of degenerating IVDs. The present study uncovered several different pathophysiological aspects of iAF and NP degeneration at the molecular level, enhancing the current knowledge of the molecular mechanisms of disc degeneration. 

### 3.4. Molecular Functions and Biological Processes

GO term analysis discovered differences in biological processes and molecular functions between NP and iAF cells. We found a higher activation of biological processes such as actin cytoskeleton organization, cell death in response to oxidative stress, collagen fibril organization, fibroblasts proliferation and interleukin 8 production in NPnD compared to iAFnD. Differences in molecular functions between iAFnD and NPnD related to cytokine activity and cytokine receptor binding were observed in five clusters. One cluster showed a higher activation of collagen binding, ECM binding and ECM structural constituent molecular functions in NPnD.

We focused the analysis on biological process such as inflammatory response, cell response to oxidative stress, ECM organization and cell-cycle regulation to determine differences linked to degeneration. We observed differences in matrix and cell-cycle regulation mostly in clusters 10 and 4. Processes involved in innate immune response, actin cytoskeleton organization, apoptotic cell clearance and cell-cycle arrest were found to be higher in most NPD clusters as compared with iAFD. Our results were in accordance with the previous literature [110,111,112]. However, activation of cartilage development, chondrocyte differentiation and collagen fibril organization pathways were cell-type and clusters-specific. As an example, a cartilage development pathway was upregulated in 8/14 of iAFD clusters, while only cluster 2 showed higher activation of the same pathway in NPD when compared to the respective nD tissue. 

GO term results comparing the differences in molecular functions between iAFnD and NPnD to iAFD and NPD, respectively, identified antioxidant activity, collagen binding, cytokine activity, ECM binding, ECM structural constituent, metallopeptidases activity and metal ion binding as the common pathways upregulated with degeneration in both cell types. Collagen binding, ECM binding and structural ECM functions were previously reported following analysis of candidate marker genes of chondrocytes in human osteoarthritis reporting an enrichment of the molecular function of collagen binding. It has also previously been suggested that the integrin-binding sialoprotein (IBSP) interacts with collagen and appears to modulate cell-matrix interactions [113,114].

Finally, KEGG analyses confirmed the GO term results. In fact, p53 and toll-like receptor signalling pathways showed higher activation in NPnD compared with iAFnD. When NPD was compared to iAFD, more pathways (cell cycle, calcium and chemokine signalling, ECM receptor interaction and actin cytoskeleton regulation) were activated and a higher number of clusters highlighted these cell-type differences. KEGG analysis evaluating the effect of degeneration in each cell type revealed common enriched pathways between NP and iAF cells. We observed a higher activation of the cell-type specific signalling pathways (p53 and toll-like receptor) involving a majority of NPD and iAFD cell clusters. We also report, in both cell types, a higher activation of apoptosis, cytokine–cytokine receptor interaction, ECM receptor interaction and wnt signalling pathways that can be linked only to degeneration. Recently, enrichment of the cytokine activity pathways was identified by scRNA-seq in human NP cells [54]. Altogether, the results of GO and KEGG analysis suggest similar processes of disc degeneration in iAF and NP cells. However, some differences were observed between the two cell types, highlighting the heterogeneity and the function specificity of IVD cells.

### 3.5. RT-qPCR

Currently, RT-qPCR is considered the gold standard for validating and confirming the presence of transcripts in bulk RNA samples [115,116,117,118]. The high number of discovered DEGs and the high expression variability observed between cell types, cell clusters and degeneration state limit the number of genes that can be validated by RT-qPCR. We selected 33 genes from scRNA-seq to evaluate their potential as biomarkers in bulk RNA sequencing in biological replicates. The first selection criterion was biomarkers in human IVD that had not been discussed in this context before. In addition, genes were selected according to the number of clusters they were expressed in. This was done to determine if the difference or opposite expression within clusters would translate in bulk RT-qPCR. Within the selected genes, mRNA expression levels of genes between NP and iAF cells in non-degenerating and degenerating IVDs revealed potential biomarkers such as *SYF2(p29)*, *MT1F* and *TAF1D*. RT-qPCR could confirm the degenerative biomarkers detected by scRNA-seq in iAF (*SOD2*, *MGST1*, *ADIRF* and *VIM*) and NP cells (*S100A1*, *VIM*, *S100A2*, *MT2A* and *TAF1D*). However, RT-qPCR analysis of *UPP1*, *PRG4*, *MT1G*, *EPYC*, *CHI3L2*, *PLA2G2A*, *FRZB*, *CAPS*, *MT-ND2*, *MT-CYB*, *CTGF*, *FGFBP2*, *MT1F*, *HMGA1*, *MSMP*, *TNRSF11B*, *SPTSSB*, *SYF2 (p29)*, *CLEC3A*, *COL2A1*, *S100B*, *SLPI*, *IGFFBP3*, *FBXO2* and *C2orf40* failed to reproduce scRNA-seq findings. Surprisingly, no correlation was observed between the genes that could not be confirmed and the number of clusters they were expressed in. The degenerative marker *vimentin* was validated for both cell types. Observed alterations in the expression of extracellular matrix and inflammatory genes are consistent with their important role in the pathogenesis of IVD degeneration [119,120]. Of interest, 30% (10/33) of the selected genes discovered by scRNA-seq analysis showed a significant mRNA expression difference and were validated by bulk RT-qPCR. DEGs that failed to translate in mRNA expression were either expressed by few cells or may be specific to the individual included in our scRNA-seq analysis. RT-qPCR samples were also expanded whereas scRNA samples came straight from digestion, presenting a technical limitation that could explain some of the observed difference between the methods. Moreover, cell pooling-based transcription analysis such as RT-qPCR might mask an entire population as positive even if few cells actively transcribe a gene within a population of otherwise negative cells. Thus, identified biomarkers, validated by qPCR, need further confirmation at the protein level.

### 3.6. Immunohistology and Western Blot

Despite the limitations of GO term and RT-qPCR analysis, proteomic analysis still validates transcriptional results, and it may provide novel targets that aid in understanding the pathophysiology of IVD degeneration. Quantitative immunofluorescence and Western blotting were performed for selected proteins including SOD2, vimentin, p29 (SYF2) and MGST to validate if they are expressed with the same pattern at the protein level. ECM metabolism is associated with the redox state of the IVD [121]. SOD2 is a mitochondrial protein that binds to the superoxide by-products of oxidative phosphorylation, converting them to hydrogen peroxide and then to water and oxygen [122]. High expression of SOD2 has been suggested to reduce oxidative stress and attenuated inflammation [123] and could potentially have a therapeutic effect in IVD degeneration [124]. In accordance with scRNA-seq and RT-qPCR, immunohistochemistry and Western Blot results confirmed a higher SOD2 expression in degenerating NP and iAF cells. This finding is in accordance with previous studies [125,126,127] where increased GAG and COL2 expression and reduced inflammation and oxidative stress were found in mouse and rat models of IVD degeneration [124,128,129]. 

Vimentin, a type III intermediate filament protein, is one of the main compounds of the cytoskeleton. It is both a player and a target in tissue damage and repair [130]. Highly expressed in fibroblasts [131], vimentin plays a fundamental role in cell mechanics [72] and in a range of other physiological functions [132]. Here, a lower level of vimentin expression was found in both cell types of degenerating IVDs, and the results were in accordance with the transcriptomic and proteomic level [133]. Similar results were reported previously with few vimentin-positive NP and iAF cells in human degenerate and aged disc tissue and a stronger immunopositivity was observed in regenerative clones [71]. 

Human p29 protein, also known as SYF2, is associated with chromatin and is involved in DNA damage response, cell-cycle arrest, pre-mRNA splicing [134,135,136,137] and cell senescence [138]. In our study, immunohistochemistry and Western Blot results confirmed the specific increase in NP cells from degenerating IVDs. Although both cell types exhibit a higher expression linked to degeneration in scRNA-seq, RT-qPCR and Western Blot results for p29 could only be confirmed for NP cells. This could be explained by sc-RNA-seq results at the cluster level where p29 expression was lower in one cluster (cluster 8) for NP cells from degenerating tissue, while it was lower in three clusters (4, 5 and 8) for iAF cells. To our knowledge no previous studies linked p29 expression specifically to human NP cells in degenerating IVDs. 

MGST1 (Microsomal Glutathione S-Transferase 1) is an enzyme with a wide substrate specificity that protects the endoplasmic reticulum and outer mitochondrial membrane from oxidative stress. ScRNA-seq showed an opposite expression of MGST1 in the identified clusters with no significant UMAP enrichment in NP or iAF cells. However, we observed a significant higher protein expression in cells from degenerating discs by Western Blot and immunohistochemistry. This could be explained by the higher number of clusters (12/14) showing higher expression at the single cell level. MGST-1 has previously been identified as a hub gene in the pathological process of osteoarthritis [139], and the mRNA was shown to be highly expressed in human iAF cells from degenerating IVDs [140]. 

In summary, we have shown that scRNA-seq provides information of IVD degeneration, that is sometimes lost in bulk analysis. Interestingly, we could not identify any specific cell clusters found only in cells from the NP or iAF region of an adult human disc. Our findings support the heterogeneity of disc cells with specific changes linked to disc degeneration. A better understanding of molecular differences between the cell clusters could reveal novel pathways, relevant processes and downstream molecules critical for health and disease of the human IVD.

## 4. Materials and Methods

### 4.1. Cell Isolation and Culture

Briefly, human lumbar IVDs were retrieved from spines obtained with familial consent (IRB#s: A04-M53-08B and Tissue Biobank 2019-4896) through the Transplant Quebec Organ Donation Program. Discs dissected from the spinal column were graded according to Thompson grading system [80] and used for tissue and cell isolation. NP and iAF cells were isolated separately from degenerating and non-degenerating discs excluding outer AF as described previously [50,141]. Supplemental Table S26 provides an overview of donor demographics and a detailed description of IVDs included in each assay.

### 4.2. Single-Cell RNA-Sequencing

We collected 500,000 NP or iAF cells from degenerating or non-degenerating discs by centrifugation, and immediately applied sc-RNA-seq analysis (Supplemental Figure S1A). Unbiased transcriptome-wide scRNA-seq analysis and computational analysis were performed, and raw sequencing data for each sample was converted to matrices of expression counts using the Cell Ranger software 10X Chromium Single Cell 3 provided by 10X Genomics. Briefly, raw BCL files from the Illumina HiSeq4000 were demultiplexed into paired-end, gzip-compressed FASTQ files using Cell Ranger’s *mkfastq*. Using Cell Ranger’s count, reads were aligned to the GRCh38 human reference genome, and transcript counts were quantified for each annotated gene within every cell. The resulting UMI count matrices (genes × cells) were then provided as input to Seurat suite (version 3.2.3) [51,52]. To filter out low-quality cells, we defined a window of a minimum of 500 and a maximum of 5000 detected genes per cell. Cells with more than 5% of the transcript counts derived from mitochondrial-encoded genes were further removed. The iAF and NP datasets were integrated using Seurat’s alignment procedure. Briefly, canonical correlation analysis (CCA) was performed to identify shared sources of variation to produce anchors across the datasets, following *SCTransform* normalization. Clustering and visualization of the integrated dataset were performed using Uniform Manifold Approximation and Projection (UMAP), an unsupervised nonlinear dimensionality reduction technique, based on the first 20 principal components with a resolution of 0.3 (*FindClusters* and *RunUMAP* functions in Seurat).

### 4.3. Cell-Cycle Analysis

A cell-cycle gene set with G1/S and G2/M genes was used in the cell-cycle analysis [142,143]. We defined the approximate cell-cycle status according to the average expression levels of these two gene types. If the expression of both G1/S and G2/M genes is less than 1.5, the corresponding cell will be classified as the quiescent cell; otherwise, it is classified as the proliferative cell [144]. For proliferative cells, if the expression of G1/S genes is less than that of G2/M genes, the corresponding cell is in the G2/M state; otherwise, it is in the G1/S state. For cells in the G1/S state, if the expression of G2/M genes is more than 1.5, the corresponding cell is in the S state; otherwise, it is in the G1 state (Supplemental Figure S1B–D).

### 4.4. Identification of Differentially Expressed Genes among Clusters

Differentially expressed genes (DEGs) were identified with the Seurat R package [51]. Differential expression analysis between cells was performed using *FindAllMarkers*. Differentially expressed genes were identified using the cut-off of: |log fold-change| > 0.25 and Bonferroni adjusted *p*-value < 0.05. Cluster-specific marker genes were identified using similar cut-off (*FindAllMarkers* function; up-regulated genes only). Pearson correlation coefficients between iAF and NP were calculated using the log fold-change of genes that are differentially expressed in either cell type. 

### 4.5. Gene Ontology (GO) Functional Enrichment and Kyoto Encyclopedia of Genes and Genomes (KEGG) Analysis of DEGs

In order to identify disturbed biological functions in IDD and to understand the importance of genes, GO classification was performed, which included the following categories: BP (biological process) and MF (molecular functions). Gene ontology (GO) provides a comprehensive set of functional annotation tools for the investigation of the biological context of large lists of genes. GO functional enrichment analysis was conducted for identified DEGs using Enrichr [145] with default parameters. KEGG pathway enrichment analysis identified significantly enriched metabolic pathways or signal transduction pathways in differentially expressed genes. For GO and KEGG clusters analysis, the distribution of expression of each cluster was demonstrated by using a heatmap.

### 4.6. RT-qPCR

Total RNA, from NP and iAF cells from non-degenerating and degenerating IVDs of five additional individuals was isolated, and reverse transcribed as previously described [50,141]. Target genes were selected and analyzed (Tables S24 and S25). Custom TaqMan Array 96-Well Fast Plates (Applied Biosystems, Foster City, CA, USA) were designed to analyze the selected genes using the primers described in Table S23. The expression of mRNAs was determined using TaqMan Master Mix (2×), and qPCR data analysed from cDNA arrays by the 2^−DDCT^ method [146] was used to validate candidate genes identified through scRNA-Seq and the validation by protein analysis. 

### 4.7. Immunohistochemistry

Pellet cultures were cryopreserved in OTC and 5 µm sections were cut for immunostaining. Sections were fixed in 4% buffered paraformaldehyde and blocked at room temperature for 1 h with 1% goat or donkey serum. After washing with PBS, sections were incubated with rabbit-anti SOD2 (ab68155), rabbit-anti vimentin (ab92547), rabbit-anti MGST1 (ab131059) and mouse-anti SYF2 (ab236417) antibodies purchased from ©Abcam plc. (Biomedical Campus, Discovery Drive, Trumpington, Cambridge, CB2 0AX, UK). Sections were then washed and incubated with the appropriate secondary antibodies. Using confocal microscopy, IHC staining was detected, and the stained sections were analyzed for fluorescence intensity blindly. 

### 4.8. Western Blot

Protein lysate of pellet cultures was mixed with lammelli buffer, then heated for 5 min at 95 °C, cooled down and vortexed before loading to gel (4–20% Criterion TGX Precast Midi Protein Gel, Bio-Rad Laboratories, Hercules, CA, USA). We loaded 15μg of protein per well. Gels were run in 1x Tris/Glycine/SDS running buffer for 1h at 80V and protein transfer for 2h at 0.45A using a transfer system (Bio-Rad Laboratories, USA). Following the transfer, the nitro cellulose membrane was rinsed with Tris Buffered Saline with Tween 20 (TBST) (0.05% Tween) and blocked with TBST (0.5% Tween 20) + 5% dry milk (or BSA) in for 60 min. The membranes were incubated with primary antibodies towards described above (SOD2, vimentin, MGST1 and SYF2). HRP-conjugated secondary antibodies were used to detect the proteins with Clarity Western ECL Substrate 1:1 (Bio-Rad Laboratories, USA), and chemiluminescence signal was measured using Image Quant LAS 4000 CCD imager and software (GE Healthcare, Life Sciences, Chicago, IL, USA). 

## Data Availability

All data generated or analyzed during this study are included in the manuscript and Supporting Files. Source data files have been provided for all figures and Figure Supplements. Single-cell RNA-sequencing is deposited in the Gene Expression Omnibus with the accession code GSE199866.

## Supplementary Materials

The following supporting information can be downloaded at: https://www.mdpi.com/article/10.3390/ijms23073993/s1.
