# Supplementary material for: Single-Cell RNA-Seq Analysis of Cells from Degenerating and Non-Degenerating Intervertebral Discs from the Same Individual Reveals New Biomarkers for Intervertebral Disc Degeneration
Source: Int J Mol Sci. 2022 Apr 3;23(7):3993. doi: 10.3390/ijms23073993 (PMC8999935; doi:10.3390/ijms23073993)
Supplement: Supplementary file 1 [file ijms-23-03993-s001.zip › ijms-1655681-SI/Supplemental Figures.pdf]

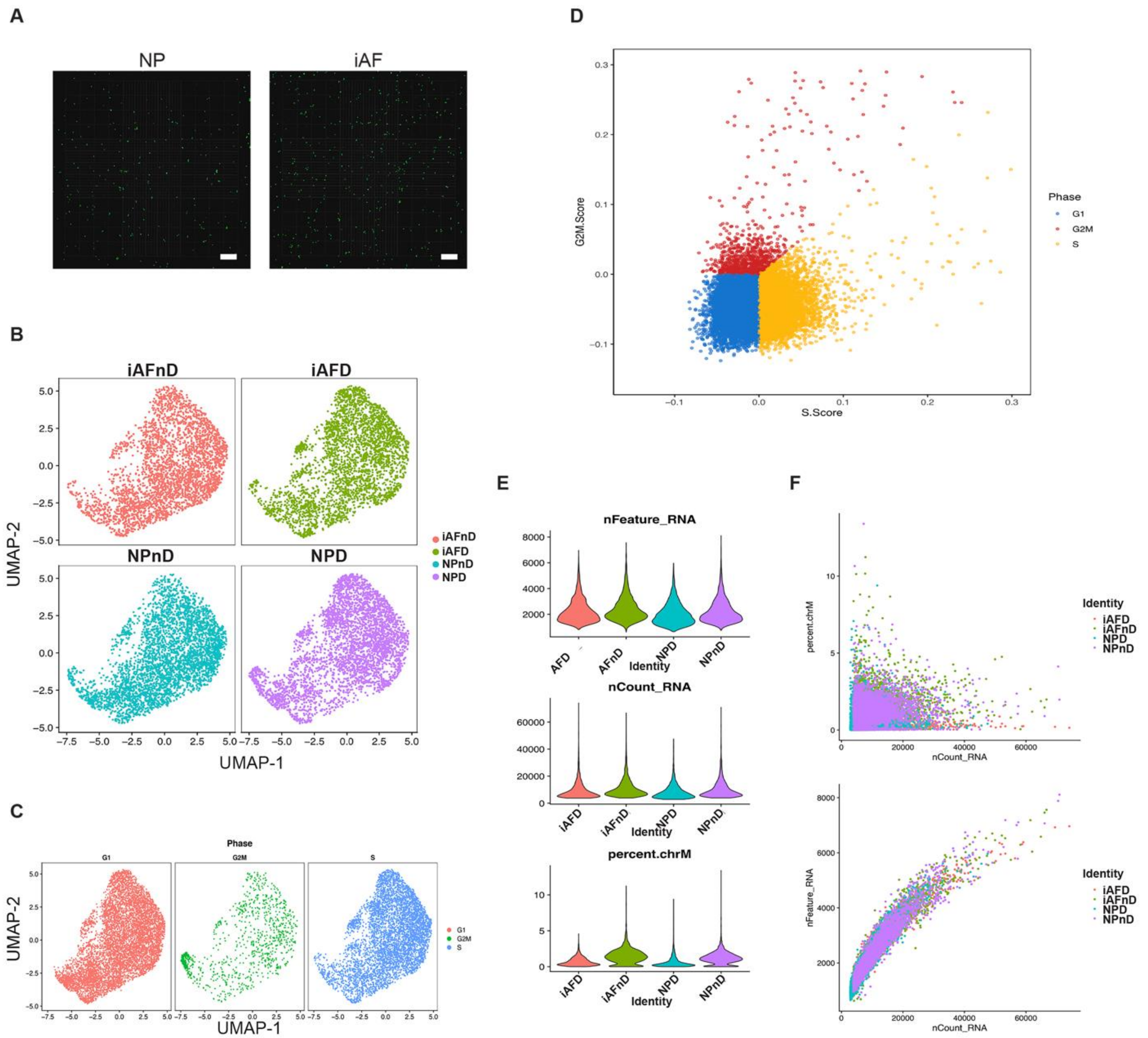

Figure S1: Cell viability and quality control. Cell viability and quality control **(A)** Representative photomicrographs showing cell viability of NP and iAF samples prior performing the sc-RNA sequencing, Scale = 100 μm. **(B)** Plot distributions of unique molecular identifiers (UMIs) and no batch effect between the four different samples was detected. **(C)** UMAP plots showing the cell cycle status of each cell. **(D)** Classification of cells categorised based on the relative expression of genes associated with the G1/S stage and G2/M stage. **(E)** Violin plots illustrating the number of genes, unique molecular identifiers (UMIs) and the percentage of mitochondrial genes in single-cell data. **(F)** The relationship between the percentage of mitochondrial genes and the mRNA reads, together with the relationship between the amount of mRNA and the reads of mRNA.

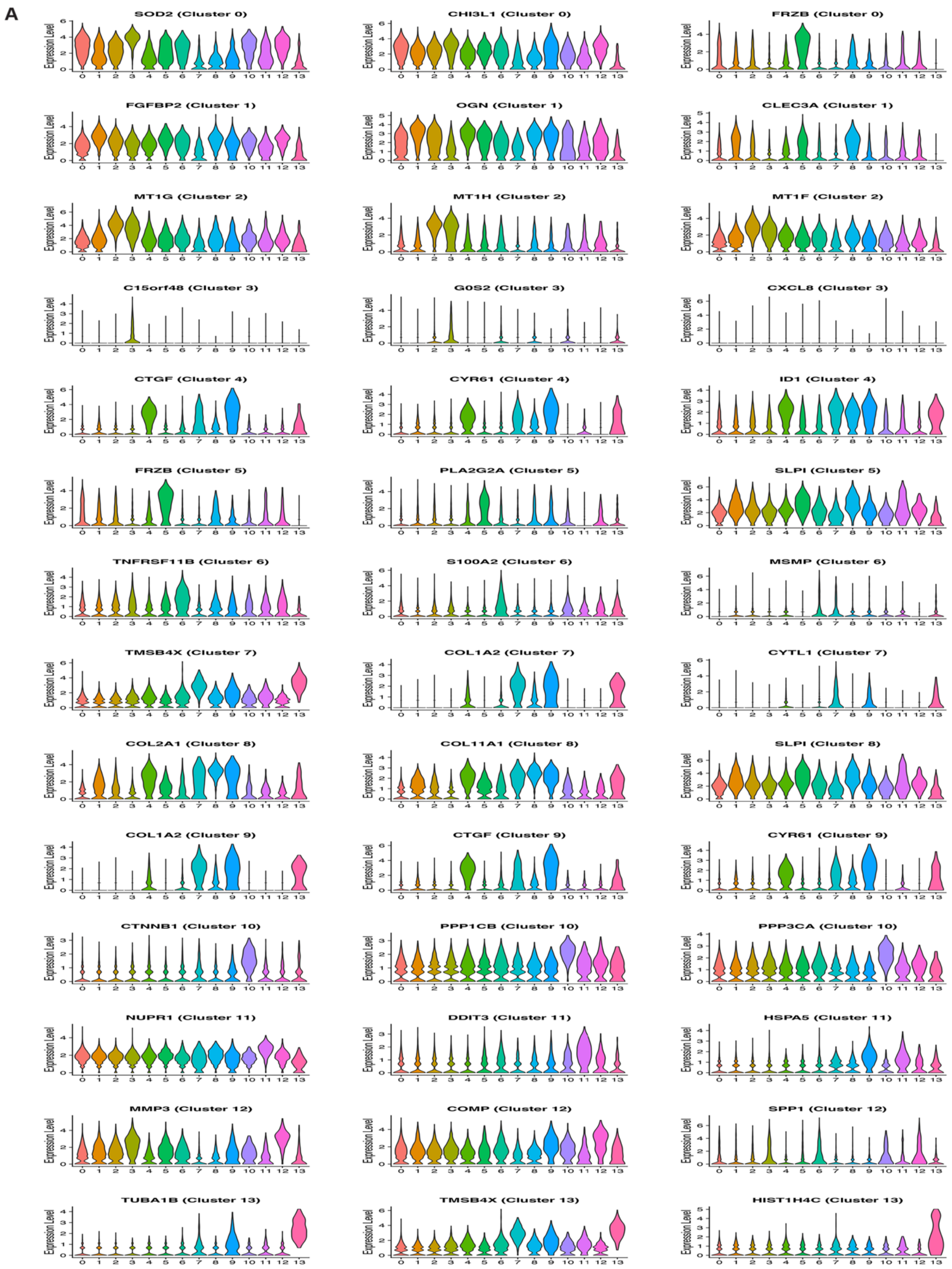

Figure S2: Violin plots of the top three upregulated genes in each cluster (0-13). Violin plots illustrating the expression of the top three upregulated genes in each of the 14 clusters (0-13) following the UMAP clustering of the human disc cells.
