# Supplementary material for: Single-Cell RNA-Seq Analysis of Cells from Degenerating and Non-Degenerating Intervertebral Discs from the Same Individual Reveals New Biomarkers for Intervertebral Disc Degeneration
Source: Int J Mol Sci. 2022 Apr 3;23(7):3993. doi: 10.3390/ijms23073993 (PMC8999935; doi:10.3390/ijms23073993)
Supplement: Supplementary file 1 [file ijms-23-03993-s001.zip › ijms-1655681-SI/Table S1.pdf]

Table S1.10X samples preparation.

Sample Name	cDNA PCR [300-6000bp] (ng/ul)	cDNA Yield (ng)	cDNA PCR used to Seq Ready (ng)	Seq Ready [300-6000] (ng/uL)	Seq Ready Yield (ng)	Index	cDNA PCR	Index PCR	Cell /uL	Viability (%)	Target	Species
**NPH**	2.48	104	79	20	628	SI-GA-A9	12	12	500	92%	>5000	Human
**AFH**	2.40	101	77	25	790	SI-GA-B9	12	12	800	93%	>5000	Human
**NPD**	1.96	82	63	16	515	SI-GA-C9	12	12	335	82%	>5000	Human
**AFD**	2.35	99	75	18	586	SI-GA-D9	12	12	215	83%	>5000	Human
