# Supplementary material for: Single-Cell RNA-Seq Analysis of Cells from Degenerating and Non-Degenerating Intervertebral Discs from the Same Individual Reveals New Biomarkers for Intervertebral Disc Degeneration
Source: Int J Mol Sci. 2022 Apr 3;23(7):3993. doi: 10.3390/ijms23073993 (PMC8999935; doi:10.3390/ijms23073993)
Supplement: Supplementary file 1 [file ijms-23-03993-s001.zip › ijms-1655681-SI/Table S10-11.pdf]

Table S10: Differentially expressed genes between NPnD and AFnD per cluster.

gene	cluster	avg_logFC	p_val	p_val_adj	pct.1	pct.2
MGP	0	0.25179672	2.17E-11	3.96E-07	1	1
MT1G	0	-0.2522361	1.81E-07	3.31E-03	0.846	0.898
MT-ND4	0	-0.2527034	4.45E-07	8.12E-03	0.892	0.905
HLA-C	0	-0.254313	3.66E-14	6.69E-10	0.932	0.966
ALDOA	0	-0.2575857	1.30E-20	2.37E-16	0.997	0.997
MT-CO1	0	-0.2599458	4.34E-07	7.91E-03	0.913	0.909
PLK2	0	-0.2631184	6.80E-10	1.24E-05	0.095	0.213
COL6A2	0	-0.2748108	6.74E-11	1.23E-06	0.521	0.654
ACTG1	0	-0.2749259	9.35E-14	1.71E-09	0.996	0.998
CHRDL2	0	-0.2771136	6.49E-07	1.19E-02	0.288	0.403
HOXC10	0	-0.2791711	7.67E-18	1.40E-13	0.659	0.847
BNIP3	0	-0.2818963	3.22E-15	5.88E-11	0.985	0.997
MT-CO2	0	-0.2820203	8.29E-09	1.51E-04	0.919	0.917
HLA-B	0	-0.2998163	3.28E-17	5.98E-13	0.826	0.919
MT1E	0	-0.3061218	4.38E-11	7.99E-07	0.935	0.964
ENO1	0	-0.3173622	5.51E-18	1.01E-13	0.972	0.983
SQSTM1	0	-0.3204348	1.34E-13	2.45E-09	0.909	0.957
PGK1	0	-0.3306687	7.39E-18	1.35E-13	0.778	0.878
HLA-A	0	-0.354205	1.10E-20	2.01E-16	0.949	0.981
MT-ND3	0	-0.4309323	2.71E-16	4.95E-12	0.752	0.809
SOD2	0	-0.5273723	1.23E-10	2.24E-06	0.932	0.966
MMP3	0	-0.5289701	7.08E-20	1.29E-15	0.554	0.785
gene	cluster	avg_logFC	p_val	p_val_adj	pct.1	pct.2
MSMP	1	0.53596609	3.42E-11	6.25E-07	0.4	0.246
MGP	1	0.39342773	1.81E-21	3.31E-17	1	1
CHI3L1	1	0.31733529	4.36E-09	7.96E-05	0.963	0.949
OGN	1	-0.2516232	6.83E-15	1.25E-10	0.994	1
MT-CO3	1	-0.2530107	6.26E-11	1.14E-06	0.938	0.948
MT-ND2	1	-0.2565422	9.68E-08	1.77E-03	0.855	0.857
MT-CO2	1	-0.2645567	6.49E-12	1.18E-07	0.963	0.972
MT1F	1	-0.2684973	6.78E-07	1.24E-02	0.889	0.937
SCRG1	1	-0.2685539	5.19E-13	9.46E-09	0.997	1
SOD2	1	-0.271484	2.31E-06	4.21E-02	0.811	0.894
MT-CO1	1	-0.277389	1.62E-13	2.95E-09	0.963	0.969
HOXC10	1	-0.2801914	9.93E-18	1.81E-13	0.712	0.84
PGK1	1	-0.3106968	1.20E-19	2.19E-15	0.842	0.934

MT1G	1	-0.3193095	2.17E-08	3.96E-04	0.855	0.932
LECT1	1	-0.3367349	1.54E-18	2.82E-14	0.978	0.988
MT1E	1	-0.345454	2.10E-15	3.83E-11	0.959	0.982
COL2A1	1	-0.3747145	3.82E-07	6.97E-03	0.952	0.969
MMP3	1	-0.3875493	4.11E-14	7.50E-10	0.643	0.826
MT-ND3	1	-0.432511	1.18E-25	2.15E-21	0.873	0.897
gene	cluster	avg_logFC	p_val	p_val_adj	pct.1	pct.2
MGP	2	0.33751931	3.14E-08	5.72E-04	1	1
C2orf40	2	0.26541059	2.37E-06	4.32E-02	1	0.997
MT1X	2	-0.2695427	6.58E-10	1.20E-05	1	1
RPS11	2	-0.2734398	1.52E-30	2.78E-26	1	1
MT-ND3	2	-0.3111312	1.28E-08	2.34E-04	0.925	0.91
MT1E	2	-0.3132891	2.90E-16	5.29E-12	1	1
CP	2	-0.3142365	6.97E-13	1.27E-08	0.837	0.963
CTGF	2	-0.4243629	2.92E-10	5.32E-06	0.429	0.641
gene	cluster	avg_logFC	p_val	p_val_adj	pct.1	pct.2
ID3	3	0.38643431	1.16E-07	2.12E-03	0.774	0.625
ID1	3	0.36510218	9.12E-08	1.66E-03	0.77	0.566
TMED2	3	0.31422054	7.62E-10	1.39E-05	0.871	0.73
ACTG1	3	-0.2572653	5.65E-07	1.03E-02	0.997	0.992
TPI1	3	-0.2580728	5.32E-10	9.71E-06	0.979	0.984
AIMP1	3	-0.2684555	8.01E-07	1.46E-02	0.491	0.633
GAPDH	3	-0.2711251	1.28E-15	2.33E-11	1	1
ANKRD12	3	-0.2726962	1.54E-07	2.81E-03	0.397	0.586
WSB1	3	-0.2731992	1.58E-06	2.88E-02	0.69	0.824
RNF114	3	-0.2778101	7.46E-08	0.00136106	0.446	0.621
ACTB	3	-0.2802378	4.71E-07	0.00859642	0.937	0.988
PMP22	3	-0.2815847	9.97E-07	0.01818905	0.666	0.777
ZNF385D	3	-0.2922713	4.94E-08	0.00090227	0.627	0.754
LDHA	3	-0.2989846	2.77E-10	5.05E-06	0.997	1
CRTAC1	3	-0.3028959	9.87E-07	0.0180035	0.216	0.395
EIF4A2	3	-0.3068387	5.63E-09	1.03E-04	0.962	0.996
WBP2	3	-0.3102784	4.16E-08	7.58E-04	0.481	0.633
MT-ND4	3	-0.3107791	6.75E-08	1.23E-03	0.972	0.977
EIF1	3	-0.3148073	7.74E-19	1.41E-14	1	1
SH3BP5	3	-0.3272633	1.23E-10	2.25E-06	0.244	0.488
PTX3	3	-0.3340527	9.23E-07	1.68E-02	0.105	0.266
CCDC85B	3	-0.3365896	6.49E-08	1.18E-03	0.666	0.797
BNIP3	3	-0.3406309	1.98E-09	3.61E-05	0.958	0.988

PSAP	3	-0.3415155	2.51E-12	4.58E-08	0.937	0.977
ALDOA	3	-0.3478536	3.74E-16	6.82E-12	0.99	0.992
MT-ND3	3	-0.3503084	7.92E-09	1.45E-04	0.889	0.93
HLA-C	3	-0.3523517	1.08E-09	1.96E-05	0.909	0.961
HNRNPC	3	-0.3572758	7.99E-13	1.46E-08	0.805	0.91
SLC3A2	3	-0.3689898	7.61E-11	1.39E-06	0.202	0.438
MMP3	3	-0.3764307	5.74E-09	1.05E-04	0.822	0.949
HLA-A	3	-0.3792713	9.05E-10	1.65E-05	0.909	0.977
S100A4	3	-0.3931186	1.00E-06	1.83E-02	0.721	0.867
HIF-1a	3	-0.4019868	4.57E-12	8.33E-08	0.749	0.879
HSD17B14	3	-0.4057964	6.98E-11	1.27E-06	0.303	0.52
PRELP	3	-0.4114668	2.08E-07	3.79E-03	0.951	0.977
NR1D1	3	-0.4117812	5.28E-10	9.63E-06	0.247	0.48
CTSL	3	-0.4181372	1.11E-09	2.03E-05	0.422	0.613
HLA-B	3	-0.4224948	7.47E-12	1.36E-07	0.742	0.898
PKM	3	-0.4257168	3.71E-12	6.77E-08	0.913	0.98
MIF	3	-0.4274285	2.82E-23	5.14E-19	0.997	1
MRPS6	3	-0.4521581	4.39E-08	0.00080182	0.878	0.93
MT1E	3	-0.4524023	1.79E-07	0.00327009	1	1
FBXO32	3	-0.4645763	2.51E-10	4.59E-06	0.111	0.324
ENO1	3	-0.5330728	8.35E-18	1.52E-13	0.93	0.988
PGK1	3	-0.5593427	6.94E-20	1.27E-15	0.76	0.938
SQSTM1	3	-0.5995085	2.50E-17	4.56E-13	0.93	0.973
SOD2	3	-0.6077706	1.23E-16	2.24E-12	0.993	0.996
WTAP	3	-0.612426	5.62E-09	1.03E-04	0.774	0.84
ADM	3	-0.6196359	1.85E-06	3.38E-02	0.564	0.68
ZFP36L1	3	-0.7304534	2.76E-07	5.04E-03	0.436	0.59
LCN2	3	-0.8512432	9.01E-10	1.64E-05	0.627	0.781
**gene**	**cluster**	**avg_logFC**	**p_val**	**p_val_adj**	**pct.1**	**pct.2**
**MSMP**	**4**	**0.59206369**	**5.62E-08**	**1.03E-03**	**0.495**	**0.297**
SH3BGRL3	4	0.30376821	1.79E-09	3.28E-05	0.976	0.932
CP	4	-0.2504612	2.31E-07	4.21E-03	0.841	0.919
NNMT	4	-0.2530077	7.05E-08	1.29E-03	0.949	0.98
RPS11	4	-0.2596472	4.17E-24	7.61E-20	1	1
PRELP	4	-0.2723925	7.04E-07	1.29E-02	0.962	0.98
MT1E	4	-0.3202198	1.96E-06	3.57E-02	0.957	0.99
HLA-A	4	-0.329627	6.54E-13	1.19E-08	0.911	0.966
HLA-B	4	-0.3485507	1.61E-14	2.94E-10	0.789	0.939
OGN	4	-0.3694091	1.01E-10	1.84E-06	0.997	1

MT-ND3	4	-0.4252319	4.77E-20	8.70E-16	0.949	0.932
LECT1	4	-0.4377333	5.45E-14	9.95E-10	0.914	0.97
**gene**	**cluster**	**avg_logFC**	**p_val**	**p_val_adj**	**pct.1**	**pct.2**
**MGP**	**5**	**0.43998162**	**1.63E-10**	**2.97E-06**	**1**	**1**
RPS11	5	-0.2553599	3.74E-20	6.83E-16	1	1
CP	5	-0.2624913	3.08E-07	5.63E-03	0.852	0.93
MIF	5	-0.2627767	2.64E-10	4.81E-06	0.996	1
FMOD	5	-0.2660912	1.02E-09	1.86E-05	0.965	0.996
PGK1	5	-0.2927694	6.56E-07	1.20E-02	0.831	0.934
BNIP3	5	-0.2986971	2.50E-08	4.56E-04	1	0.996
WSB1	5	-0.3182608	2.18E-09	3.98E-05	0.623	0.797
CCDC80	5	-0.3331481	2.03E-08	3.71E-04	0.965	0.973
CTGF	5	-0.4279264	6.09E-07	1.11E-02	0.419	0.617
MT-ND1	5	-0.4530368	1.87E-10	3.42E-06	0.574	0.781
MT-ATP6	5	-0.4587192	1.10E-11	2.01E-07	0.718	0.898
MT-ND2	5	-0.4635159	4.68E-09	8.54E-05	0.627	0.785
MT-CO3	5	-0.4667023	5.76E-12	1.05E-07	0.778	0.934
MT-ND4	5	-0.5260146	2.26E-14	4.12E-10	0.831	0.953
MT-CYB	5	-0.5748444	2.47E-15	4.51E-11	0.683	0.875
MT-CO2	5	-0.6003844	2.23E-16	4.06E-12	0.838	0.965
MT-CO1	5	-0.6137221	2.25E-16	4.11E-12	0.842	0.969
MT-ND3	5	-0.6397894	5.57E-17	1.02E-12	0.637	0.871
**gene**	**cluster**	**avg_logFC**	**p_val**	**p_val_adj**	**pct.1**	**pct.2**
ENO1	6	-0.4568745	2.06E-06	3.75E-02	0.982	1
**gene**	**cluster**	**avg_logFC**	**p_val**	**p_val_adj**	**pct.1**	**pct.2**
MT-CO2	7	-0.2576745	2.85E-07	5.20E-03	0.984	0.988
VKORC1	7	-0.2578301	1.41E-06	2.57E-02	0.996	1
PDIA3	7	-0.2788365	2.39E-06	4.36E-02	0.589	0.734
ITM2B	7	-0.2840279	2.09E-06	3.81E-02	0.837	0.925
TMEM59	7	-0.3041115	5.68E-07	1.04E-02	0.825	0.896
PPIC	7	-0.316072	1.84E-08	3.36E-04	0.894	0.965
CD63	7	-0.3311561	8.18E-09	1.49E-04	0.996	1
HLA-C	7	-0.338294	7.55E-07	1.38E-02	0.671	0.827
PRELP	7	-0.3403027	6.72E-07	1.23E-02	0.78	0.884
MT-ND2	7	-0.3420792	5.42E-11	9.88E-07	0.967	0.988
HLA-B	7	-0.3565343	3.56E-07	6.50E-03	0.492	0.682
SPARC	7	-0.3743277	2.60E-10	4.74E-06	0.992	0.994
COL3A1	7	-0.4226575	2.31E-06	4.22E-02	0.614	0.775
IGFBP7	7	-0.4227234	1.52E-08	2.78E-04	0.915	0.983

SCRG1	7	-0.449223	1.09E-07	1.99E-03	0.967	0.994
HLA-A	7	-0.4515007	8.62E-11	1.57E-06	0.671	0.855
MT-ND3	7	-0.4578881	1.99E-23	3.63E-19	0.972	0.988
COL9A3	7	-0.5284739	4.95E-07	9.04E-03	0.512	0.711
**gene**	**cluster**	**avg_logFC**	**p_val**	**p_val_adj**	**pct.1**	**pct.2**
RPS11	8	-0.2716817	3.31E-18	6.03E-14	1	1
SPARC	8	-0.2899288	1.69E-06	3.08E-02	0.962	1
CCDC80	8	-0.3016224	1.34E-07	2.44E-03	0.837	0.939
COL11A1	8	-0.3104508	7.70E-09	1.40E-04	0.989	1
MT-CYB	8	-0.3112126	2.09E-06	3.81E-02	0.97	0.949
COL9A2	8	-0.3174083	1.10E-06	2.01E-02	0.697	0.838
MT-ND3	8	-0.3306738	4.22E-08	7.70E-04	0.962	0.949
COL2A1	8	-0.4068242	2.85E-09	5.20E-05	1	1
**gene**	**cluster**	**avg_logFC**	**p_val**	**p_val_adj**	**pct.1**	**pct.2**
MT-CO3	9	-0.4564641	4.47E-07	8.16E-03	0.955	0.976
MT-ND3	9	-0.5405621	6.49E-08	1.18E-03	0.91	0.964
**gene**	**cluster**	**avg_logFC**	**p_val**	**p_val_adj**	**pct.1**	**pct.2**
EIF4B	10	-0.5040037	7.42E-07	1.35E-02	0.929	0.98
**gene**	**cluster**	**avg_logFC**	**p_val**	**p_val_adj**	**pct.1**	**pct.2**
MT-ND3	11	-0.9650522	4.12E-07	7.51E-03	0.193	0.545
MT-ND2	11	-1.2057694	5.41E-08	9.88E-04	0.239	0.621

Table S11: Differentially expressed genes between NPD and AFD per cluster.

gene	cluster	avg_logFC	p_val	p_val_adj	pct.1	pct.2
HMGA1	0	0.87400514	5.82E-78	1.06E-73	0.858	0.404
CD55	0	0.71161478	1.28E-61	2.34E-57	0.922	0.662
CXCL8	0	0.6888357	8.20E-13	1.50E-08	0.182	0.051
TIMP3	0	0.57563956	5.21E-25	9.50E-21	0.749	0.52
C11orf96	0	0.54331093	4.16E-24	7.59E-20	0.528	0.285
MT2A	0	0.52415206	3.11E-25	5.68E-21	1	1
TM4SF1	0	0.49871341	1.08E-26	1.97E-22	0.777	0.484
NRP2	0	0.49745642	5.95E-19	1.09E-14	0.606	0.403
SMOX	0	0.46728082	7.60E-18	1.39E-13	0.581	0.404
RGCC	0	0.45226257	6.48E-20	1.18E-15	0.911	0.751
UPP1	0	0.41786366	1.14E-27	2.09E-23	0.875	0.621
GSTO1	0	0.36269994	4.82E-27	8.80E-23	0.906	0.738
TMSB4X	0	0.35804218	3.97E-07	0.00725004	0.841	0.726
SPP1	0	0.35356897	2.68E-06	0.04895527	0.897	0.753
SNHG12	0	0.34435728	3.36E-17	6.13E-13	0.743	0.605
S100A10	0	0.33748103	1.07E-16	1.95E-12	0.987	0.939
TMSB10	0	0.32759749	2.65E-20	4.84E-16	0.996	0.993
NBEAL1	0	0.31576574	2.72E-28	4.96E-24	0.996	0.986
TGM2	0	0.30043324	1.69E-15	3.09E-11	0.504	0.312
EPB41L4A-AS1	0	0.29836863	2.50E-24	4.56E-20	0.964	0.897
ANXA1	0	0.28346429	1.32E-08	0.00024075	0.716	0.63
ZFAS1	0	0.28289146	2.49E-35	4.55E-31	1	0.996
CTSL	0	0.28283214	9.45E-08	0.00172482	0.809	0.659
PDLIM4	0	0.27474884	4.58E-15	8.35E-11	0.87	0.76
AC090498.1	0	0.26079516	1.25E-48	2.27E-44	1	1
TAF1D	0	0.26062799	1.43E-12	2.61E-08	0.706	0.599
CCND1	0	0.25723181	1.57E-09	2.87E-05	0.424	0.283
RELB	0	0.25441688	3.17E-10	5.78E-06	0.566	0.417
GPX4	0	0.25281092	3.16E-18	5.77E-14	0.997	0.978
SH3BGRL3	0	0.25151785	5.75E-17	1.05E-12	0.991	0.977
PKM	0	-0.2511766	5.55E-13	1.01E-08	0.861	0.908
CALM2	0	-0.2545043	4.56E-16	8.32E-12	0.938	0.975
HAPLN1	0	-0.2550567	1.61E-09	2.94E-05	0.44	0.59
MDFI	0	-0.2553418	4.31E-11	7.87E-07	0.15	0.287
CDK5RAP2	0	-0.2562409	1.71E-09	3.11E-05	0.166	0.305
FCGRT	0	-0.2565341	1.28E-17	2.33E-13	0.286	0.507

SNRPN	0	-0.2583813	1.58E-15	2.89E-11	0.565	0.731
DYNLL1	0	-0.2624758	6.71E-15	1.22E-10	0.743	0.884
ID4	0	-0.2651441	8.84E-07	0.01612431	0.288	0.401
DDR2	0	-0.2656216	1.80E-18	3.28E-14	0.184	0.397
MT-ND2	0	-0.2688783	1.57E-13	2.87E-09	0.31	0.502
MT-ND3	0	-0.269583	1.95E-11	3.56E-07	0.314	0.489
CCNG2	0	-0.2714739	1.34E-12	2.45E-08	0.39	0.558
CDO1	0	-0.2732633	1.19E-17	2.17E-13	0.093	0.269
TPM2	0	-0.2734079	1.09E-19	1.99E-15	0.269	0.509
CALR	0	-0.273456	7.10E-17	1.30E-12	0.391	0.594
B2M	0	-0.2737571	1.27E-21	2.31E-17	0.958	0.995
NT5C3A	0	-0.2737897	5.54E-10	1.01E-05	0.316	0.46
CTHRC1	0	-0.2779223	3.59E-11	6.56E-07	0.314	0.484
LSP1	0	-0.2781954	3.38E-22	6.16E-18	0.092	0.296
MT-CYB	0	-0.2784171	5.03E-12	9.17E-08	0.361	0.527
NNMT	0	-0.2812719	1.23E-10	2.25E-06	0.782	0.854
FTL	0	-0.28467	3.00E-34	5.48E-30	1	1
PTMS	0	-0.2851974	1.67E-12	3.05E-08	0.332	0.502
EPYC	0	-0.2861632	2.09E-25	3.82E-21	0.029	0.206
PABPC1	0	-0.2900105	5.77E-17	1.05E-12	0.755	0.877
TPI1	0	-0.2904195	4.72E-20	8.61E-16	0.983	0.998
CRIP1	0	-0.2906448	1.12E-24	2.05E-20	0.146	0.392
CALD1	0	-0.2919546	2.00E-16	3.64E-12	0.369	0.592
COL11A2	0	-0.2924296	9.39E-19	1.71E-14	0.198	0.408
MT1E	0	-0.2928024	1.24E-08	0.0002264	0.934	0.94
P4HA1	0	-0.2934236	2.86E-17	5.22E-13	0.462	0.666
FGF7	0	-0.2934463	1.59E-07	0.00289272	0.09	0.188
FOSL1	0	-0.2949119	3.25E-08	0.00059319	0.284	0.417
PPP1R3C	0	-0.2967752	8.44E-13	1.54E-08	0.221	0.381
HLA-A	0	-0.2973082	3.09E-18	5.63E-14	0.764	0.87
MMP3	0	-0.2982117	6.33E-10	1.15E-05	0.79	0.883
SAT1	0	-0.300481	6.88E-20	1.25E-15	0.943	0.98
EEF2	0	-0.3021791	7.21E-25	1.32E-20	0.979	0.998
TMEM59	0	-0.3023766	2.59E-21	4.72E-17	0.955	0.982
MAOB	0	-0.3071898	1.07E-19	1.96E-15	0.2	0.422
CD320	0	-0.3093058	6.13E-27	1.12E-22	0.186	0.451
IGFBP6	0	-0.3107711	3.25E-09	5.93E-05	0.302	0.442
PNRC1	0	-0.3114555	2.20E-14	4.02E-10	0.923	0.955
GSN	0	-0.3141919	1.14E-22	2.08E-18	0.235	0.482

EEF1A1	0	-0.3143059	2.46E-57	4.48E-53	1	1
COL6A2	0	-0.3151065	1.53E-19	2.78E-15	0.344	0.561
MT-ND1	0	-0.3177474	3.55E-10	6.47E-06	0.272	0.417
SLC16A3	0	-0.3178981	7.42E-16	1.35E-11	0.306	0.505
PLD3	0	-0.3180703	7.25E-24	1.32E-19	0.237	0.495
FAM162A	0	-0.3190679	1.35E-18	2.47E-14	0.927	0.957
BTG1	0	-0.3206918	9.91E-12	1.81E-07	0.968	0.989
PPP3CA	0	-0.3237946	1.49E-15	2.73E-11	0.858	0.93
ID2	0	-0.3244189	3.56E-13	6.49E-09	0.306	0.484
C4orf3	0	-0.3259735	2.18E-29	3.97E-25	0.968	0.991
PGK1	0	-0.3308867	1.88E-19	3.43E-15	0.958	0.953
NAMPT	0	-0.3345797	4.76E-15	8.68E-11	0.537	0.697
CTSF	0	-0.3347353	8.36E-39	1.53E-34	0.073	0.357
S100A4	0	-0.3354836	3.03E-09	5.52E-05	0.533	0.65
BNIP3	0	-0.3377216	1.10E-18	2.00E-14	0.995	0.998
SERPINA1	0	-0.3380581	1.06E-16	1.93E-12	0.924	0.975
AEBP1	0	-0.3428151	9.89E-19	1.80E-14	0.455	0.668
RHOBTB3	0	-0.3463275	7.85E-17	1.43E-12	0.249	0.451
DDIT4	0	-0.3467532	1.26E-20	2.29E-16	0.308	0.558
SDC2	0	-0.3513917	1.87E-22	3.41E-18	0.577	0.778
IFITM1	0	-0.3514496	7.84E-20	1.43E-15	0.282	0.514
TMEM45A	0	-0.3531612	7.17E-16	1.31E-11	0.847	0.917
MT-ATP6	0	-0.3535826	1.03E-12	1.88E-08	0.562	0.699
NUCB1	0	-0.3578246	1.55E-27	2.83E-23	0.345	0.612
SOX9	0	-0.3591661	6.12E-17	1.12E-12	0.488	0.659
PRDX4	0	-0.3628861	2.42E-23	4.41E-19	0.443	0.671
EID1	0	-0.3648638	2.30E-32	4.19E-28	0.744	0.919
ARRDC3	0	-0.364906	1.15E-07	0.00210413	0.422	0.532
VKORC1	0	-0.3675923	4.05E-31	7.39E-27	0.847	0.942
PSAP	0	-0.3685248	8.67E-31	1.58E-26	0.743	0.899
FXYD6	0	-0.3715788	1.32E-20	2.41E-16	0.497	0.702
FBXO2	0	-0.3726228	1.34E-26	2.45E-22	0.418	0.659
MT1X	0	-0.3749812	6.18E-09	0.00011268	0.996	1
SPON2	0	-0.3796866	1.62E-43	2.96E-39	0.036	0.312
IER2	0	-0.3819612	1.16E-12	2.11E-08	0.184	0.343
CLEC3A	0	-0.3828544	5.32E-24	9.72E-20	0.066	0.264
COL2A1	0	-0.3835107	1.60E-21	2.91E-17	0.194	0.415
CD99	0	-0.3870086	2.53E-30	4.61E-26	0.711	0.883
TSC22D1	0	-0.3879285	2.10E-14	3.83E-10	0.398	0.579

ITM2B	0	-0.3892783	1.09E-31	1.99E-27	0.952	0.982
SLC39A8	0	-0.3930242	2.19E-29	4.00E-25	0.249	0.525
MGP	0	-0.3947381	9.92E-13	1.81E-08	0.997	0.998
TGFBI	0	-0.3948773	4.88E-16	8.90E-12	0.513	0.677
NDUFA4L2	0	-0.396054	3.18E-66	5.81E-62	1	1
EGLN3	0	-0.4057408	1.55E-21	2.83E-17	0.284	0.525
ATRAID	0	-0.4084843	2.91E-34	5.31E-30	0.601	0.843
LUM	0	-0.4121172	2.62E-28	4.78E-24	1	1
MFGE8	0	-0.4177442	1.21E-23	2.21E-19	0.699	0.829
EGR1	0	-0.4189965	1.58E-28	2.88E-24	0.139	0.403
GPX3	0	-0.4193587	8.33E-26	1.52E-21	0.769	0.912
IFITM3	0	-0.4334142	5.75E-33	1.05E-28	0.808	0.931
FAM46A	0	-0.442953	5.87E-27	1.07E-22	0.326	0.585
HIF1A	0	-0.4442862	2.55E-33	4.65E-29	0.639	0.868
NGFRAP1	0	-0.4466207	3.25E-34	5.94E-30	0.492	0.764
GLRX	0	-0.4478171	7.88E-18	1.44E-13	0.219	0.422
PAM	0	-0.4481937	1.51E-37	2.75E-33	0.279	0.603
BAMBI	0	-0.4523121	1.11E-17	2.02E-13	0.375	0.576
IFITM2	0	-0.453165	1.43E-28	2.61E-24	0.775	0.89
DUSP1	0	-0.4536711	7.98E-20	1.46E-15	0.267	0.484
CFD	0	-0.4547764	1.74E-32	3.18E-28	0.062	0.307
C10orf10	0	-0.4590114	7.50E-20	1.37E-15	0.444	0.65
SERPING1	0	-0.4643116	1.72E-37	3.13E-33	0.418	0.711
IGFBP7	0	-0.4682824	3.22E-32	5.87E-28	0.599	0.841
COL9A3	0	-0.4692735	3.03E-29	5.52E-25	0.22	0.504
NOVA1	0	-0.4738627	3.83E-11	6.98E-07	0.794	0.847
ADM	0	-0.4880969	1.64E-19	3.00E-15	0.757	0.854
CA9	0	-0.489323	5.61E-33	1.02E-28	0.268	0.563
MALAT1	0	-0.4920693	2.26E-53	4.13E-49	1	1
ID1	0	-0.4952113	7.85E-13	1.43E-08	0.231	0.395
C1S	0	-0.4984667	2.99E-39	5.46E-35	0.408	0.72
CCDC85B	0	-0.4985777	2.56E-25	4.67E-21	0.899	0.949
COL11A1	0	-0.5000618	1.00E-36	1.83E-32	0.279	0.596
HTRA1	0	-0.5129422	5.29E-38	9.65E-34	0.418	0.729
SOD3	0	-0.5222278	1.58E-26	2.88E-22	0.867	0.933
PCOLCE2	0	-0.5241619	3.51E-51	6.41E-47	0.72	0.94
ID3	0	-0.5276835	5.86E-17	1.07E-12	0.143	0.323
MT-CO2	0	-0.5334273	4.02E-22	7.33E-18	0.85	0.917
WWP2	0	-0.5365451	8.07E-36	1.47E-31	0.427	0.713

PHLDA1	0	-0.5478882	9.81E-13	1.79E-08	0.301	0.469
LCN2	0	-0.5543773	3.24E-21	5.92E-17	0.261	0.48
GAPDH	0	-0.5583981	2.24E-48	4.09E-44	1	1
FOS	0	-0.5640224	2.26E-30	4.12E-26	0.297	0.569
S100B	0	-0.564042	4.43E-30	8.08E-26	0.436	0.673
FMOD	0	-0.5710339	7.11E-56	1.30E-51	0.627	0.912
FGFBP2	0	-0.5769326	2.55E-30	4.66E-26	0.891	0.966
CP	0	-0.6038779	1.01E-28	1.84E-24	0.357	0.599
RBP4	0	-0.6149579	6.31E-38	1.15E-33	0.312	0.617
OGN	0	-0.6286272	2.06E-44	3.75E-40	0.537	0.857
IGFBP5	0	-0.6368556	1.54E-30	2.81E-26	0.154	0.428
CST3	0	-0.6520033	2.54E-73	4.64E-69	0.954	0.995
PLAC9	0	-0.6651449	4.03E-38	7.36E-34	0.34	0.653
G0S2	0	-0.6737991	3.27E-07	0.00597518	0.265	0.395
MT1F	0	-0.6931354	1.68E-33	3.06E-29	0.773	0.91
MT1G	0	-0.6971816	3.11E-21	5.68E-17	0.936	0.958
ACAN	0	-0.7135483	7.63E-52	1.39E-47	0.71	0.924
LECT1	0	-0.7309481	7.58E-66	1.38E-61	0.334	0.783
CLU	0	-0.7597861	7.48E-61	1.36E-56	0.996	1
JUNB	0	-0.7682042	3.31E-32	6.05E-28	0.334	0.616
DCN	0	-0.80842	3.53E-109	6.44E-105	0.999	1
C2orf40	0	-0.8092448	1.49E-62	2.71E-58	0.93	0.993
PRELP	0	-0.9267468	2.63E-85	4.80E-81	0.771	0.98
CCDC80	0	-0.9669301	1.16E-85	2.11E-81	0.706	0.964
CHI3L2	0	-0.9767206	7.96E-68	1.45E-63	0.554	0.912
CHI3L1	0	-1.1404299	3.64E-70	6.65E-66	0.886	0.975
PLA2G2A	0	-1.1549015	2.08E-82	3.80E-78	0.198	0.7
SLPI	0	-1.2244489	2.89E-75	5.27E-71	0.735	0.957
CTSD	0	-1.3158617	4.78E-97	8.72E-93	0.721	0.969
**gene**	**cluster**	**avg_logFC**	**p_val**	**p_val_adj**	**pct.1**	**pct.2**
PRG4	1	1.01243342	3.16E-11	5.76E-07	0.651	0.479
HMGA1	1	0.90349513	4.67E-49	8.53E-45	0.725	0.299
MT2A	1	0.90163191	2.72E-37	4.96E-33	1	1
UPP1	1	0.86651142	1.45E-49	2.65E-45	0.804	0.42
SPP1	1	0.85278565	1.38E-15	2.52E-11	0.891	0.717
TM4SF1	1	0.84445325	7.19E-41	1.31E-36	0.748	0.347
TIMP3	1	0.78009287	2.43E-26	4.43E-22	0.766	0.498
CD55	1	0.75206725	7.74E-48	1.41E-43	0.926	0.648
NRP2	1	0.74867343	6.90E-35	1.26E-30	0.597	0.247

CTSL	1	0.73419897	6.04E-27	1.10E-22	0.795	0.507
RGCC	1	0.67608743	9.59E-33	1.75E-28	0.95	0.781
SMOX	1	0.642074	1.45E-26	2.65E-22	0.601	0.299
C11orf96	1	0.63014832	5.55E-24	1.01E-19	0.533	0.235
SOD2	1	0.56237724	2.68E-27	4.90E-23	1	0.982
IGFBP3	1	0.54409884	2.21E-12	4.04E-08	0.605	0.379
TMSB10	1	0.4877596	6.16E-26	1.12E-21	0.992	0.989
EPB41L4A-AS1	1	0.47396886	7.09E-35	1.29E-30	0.932	0.742
GSTO1	1	0.47370446	6.68E-31	1.22E-26	0.857	0.607
EIF1	1	0.46951081	1.01E-50	1.83E-46	1	1
MSMP	1	0.44287471	1.14E-06	0.02083911	0.244	0.123
TGM2	1	0.42430131	5.38E-25	9.81E-21	0.494	0.196
ZFAS1	1	0.41799223	3.06E-37	5.58E-33	1	0.982
SNHG12	1	0.41465075	2.66E-20	4.86E-16	0.626	0.388
ANXA1	1	0.41318715	1.82E-12	3.33E-08	0.822	0.717
GYPC	1	0.41118004	1.10E-17	2.01E-13	0.535	0.306
STK17A	1	0.40353612	4.40E-18	8.02E-14	0.647	0.402
CCND1	1	0.39572793	1.01E-15	1.85E-11	0.477	0.249
BZW1	1	0.38240136	3.51E-16	6.40E-12	0.857	0.655
ABI3BP	1	0.38119569	3.57E-08	0.00065199	0.659	0.553
FAP	1	0.36775408	1.90E-15	3.47E-11	0.459	0.24
VMP1	1	0.33850275	2.08E-09	3.79E-05	0.651	0.523
RGS16	1	0.33795183	1.42E-11	2.59E-07	0.382	0.187
SYF2	1	0.33547334	3.64E-20	6.64E-16	0.913	0.795
LGALS1	1	0.3271778	2.61E-09	4.77E-05	0.986	0.957
AIMP1	1	0.32577682	1.20E-14	2.19E-10	0.698	0.5
TPM1	1	0.32272775	6.47E-10	1.18E-05	0.539	0.381
MIF	1	0.31466901	4.28E-27	7.81E-23	1	1
SERPINE1	1	0.31080905	5.41E-11	9.88E-07	0.341	0.162
BHLHE40	1	0.30581355	5.09E-11	9.29E-07	0.754	0.582
NT5E	1	0.30135489	2.83E-11	5.17E-07	0.424	0.24
PMP22	1	0.29839011	2.98E-18	5.43E-14	0.934	0.772
RHOC	1	0.29782971	7.94E-11	1.45E-06	0.872	0.753
RNF114	1	0.29733707	1.24E-13	2.25E-09	0.734	0.541
SGIP1	1	0.29387675	1.57E-08	0.00028624	0.359	0.205
DNER	1	0.29294177	2.08E-11	3.79E-07	0.694	0.527
AC090498.1	1	0.29283715	1.26E-37	2.31E-33	1	1
NBEAL1	1	0.2886867	1.99E-21	3.63E-17	0.998	0.977
NBPF14	1	0.28735427	6.22E-12	1.13E-07	0.471	0.272

ISG20	1	0.28587751	2.30E-10	4.20E-06	0.657	0.507
PRDX6	1	0.28284715	4.22E-15	7.70E-11	0.911	0.756
KLF3	1	0.27407642	2.50E-08	0.00045549	0.444	0.292
S100A10	1	0.27313106	1.88E-09	3.43E-05	0.996	0.984
RPL36	1	0.27282491	6.12E-39	1.12E-34	1	1
S100A16	1	0.26484041	1.00E-07	0.00183108	0.405	0.263
RELB	1	0.26239992	6.08E-10	1.11E-05	0.448	0.276
HMGA2	1	0.26152002	2.10E-13	3.83E-09	0.331	0.135
CSGALNACT2	1	0.26018986	2.98E-08	0.00054417	0.417	0.269
AKAP12	1	0.25926431	1.53E-13	2.79E-09	0.225	0.057
DAB2	1	0.25654013	2.36E-08	0.00043022	0.422	0.274
RAN	1	0.25642979	2.26E-11	4.13E-07	0.824	0.678
FXYD5	1	0.25535158	4.48E-13	8.17E-09	0.942	0.852
LINC00493	1	0.2544324	5.54E-14	1.01E-09	0.93	0.815
KRT18	1	0.25384735	7.05E-12	1.29E-07	0.345	0.158
PIM3	1	-0.2502261	5.08E-11	9.27E-07	0.304	0.498
RPN2	1	-0.2503091	9.95E-15	1.82E-10	0.083	0.267
IFITM2	1	-0.2503167	1.17E-07	0.00213307	0.816	0.858
HNRNPA1	1	-0.2511896	3.47E-13	6.34E-09	0.973	0.986
MYL6B	1	-0.2512263	2.10E-10	3.83E-06	0.589	0.717
MYEOV2	1	-0.2513269	5.73E-10	1.05E-05	0.517	0.683
SUMO2	1	-0.2517413	4.14E-17	7.56E-13	0.965	0.986
ROMO1	1	-0.2521707	4.96E-13	9.05E-09	0.773	0.893
COL6A2	1	-0.252237	6.54E-08	0.00119329	0.498	0.632
CUTA	1	-0.2533044	9.34E-13	1.70E-08	0.721	0.829
COL5A2	1	-0.2534561	1.89E-11	3.45E-07	0.18	0.372
TMEM219	1	-0.2536525	3.68E-10	6.72E-06	0.508	0.662
HMGN3	1	-0.2554957	6.83E-15	1.25E-10	0.136	0.349
IFI6	1	-0.2555641	6.66E-13	1.21E-08	0.242	0.452
NOL3	1	-0.2557831	2.43E-09	4.44E-05	0.36	0.53
VAMP2	1	-0.2596295	1.45E-11	2.64E-07	0.711	0.813
C1orf21	1	-0.2604036	5.65E-11	1.03E-06	0.39	0.582
ZFR	1	-0.2609336	1.11E-11	2.03E-07	0.184	0.365
STK26	1	-0.2627801	2.45E-10	4.47E-06	0.324	0.505
PDIA4	1	-0.2633674	3.06E-15	5.58E-11	0.184	0.406
H3F3B	1	-0.2634249	5.92E-15	1.08E-10	1	1
FKBP9	1	-0.263649	1.61E-17	2.93E-13	0.112	0.34
IFITM3	1	-0.2658528	3.10E-10	5.66E-06	0.829	0.87
EEF2	1	-0.26639	2.22E-13	4.06E-09	0.973	0.975

RP5-1097P24.1	1	-0.2671737	6.91E-12	1.26E-07	0.109	0.276
CMPK1	1	-0.2676716	2.65E-11	4.84E-07	0.607	0.76
GPX3	1	-0.2680835	3.61E-13	6.58E-09	0.857	0.959
CALM2	1	-0.2686565	2.34E-15	4.27E-11	0.932	0.986
MT-ND5	1	-0.2694756	1.06E-09	1.93E-05	0.397	0.587
PSAP	1	-0.2695778	7.91E-11	1.44E-06	0.853	0.936
DUT	1	-0.2696992	1.06E-11	1.94E-07	0.471	0.648
H1F0	1	-0.2698288	5.36E-12	9.78E-08	0.209	0.416
SARAF	1	-0.2699735	1.52E-12	2.77E-08	0.51	0.692
WFDC2	1	-0.2701825	4.90E-22	8.94E-18	0.033	0.242
BSG	1	-0.2710012	4.67E-10	8.52E-06	0.547	0.701
N4BP2L2	1	-0.2716541	1.61E-12	2.94E-08	0.616	0.795
FKBP10	1	-0.2718472	1.32E-11	2.41E-07	0.37	0.562
NUCB1	1	-0.2749493	4.90E-12	8.94E-08	0.496	0.68
ATPIF1	1	-0.2758406	3.15E-11	5.75E-07	0.564	0.724
PCOLCE	1	-0.2759802	5.73E-12	1.05E-07	0.14	0.317
CLU	1	-0.2759968	6.72E-07	0.01226316	1	1
PPIC	1	-0.2767204	9.37E-14	1.71E-09	0.223	0.45
BRK1	1	-0.2770195	3.35E-14	6.11E-10	0.678	0.852
PLXDC2	1	-0.2782285	5.00E-14	9.12E-10	0.331	0.555
MAGED1	1	-0.2808712	9.66E-17	1.76E-12	0.056	0.242
CTSA	1	-0.2812697	3.52E-21	6.43E-17	0.066	0.295
SRP9	1	-0.2813842	1.92E-15	3.50E-11	0.632	0.811
MIA	1	-0.2829209	1.24E-16	2.27E-12	0.988	0.998
TOB1	1	-0.2836891	5.45E-16	9.94E-12	0.186	0.422
BTG1	1	-0.284334	1.68E-10	3.07E-06	0.965	0.986
NME4	1	-0.2846164	1.36E-13	2.48E-09	0.242	0.452
OS9	1	-0.2851735	2.24E-11	4.08E-07	0.347	0.525
PRKAR1A	1	-0.2854726	1.23E-09	2.25E-05	0.568	0.71
HSPB6	1	-0.2855484	6.30E-20	1.15E-15	0.085	0.317
CDK5RAP2	1	-0.2858289	1.76E-08	0.0003208	0.124	0.263
SGCB	1	-0.2862348	3.64E-15	6.64E-11	0.316	0.562
SUMO1	1	-0.2866472	8.93E-15	1.63E-10	0.531	0.735
IFITM1	1	-0.2876736	2.24E-09	4.09E-05	0.324	0.5
TMED10	1	-0.2878057	1.46E-14	2.67E-10	0.82	0.9
MORF4L2	1	-0.2888536	9.35E-13	1.71E-08	0.444	0.637
MT1X	1	-0.2894924	7.45E-09	0.00013599	0.996	1
IGFBP7	1	-0.2897636	5.85E-10	1.07E-05	0.82	0.911
PNKD	1	-0.291118	1.38E-20	2.51E-16	0.14	0.395

HMGN2	1	-0.2932952	3.35E-16	6.11E-12	0.828	0.893
SLC16A3	1	-0.2952618	3.23E-10	5.89E-06	0.329	0.509
H3F3A	1	-0.2964453	1.26E-14	2.29E-10	0.882	0.934
NEAT1	1	-0.2969495	4.23E-13	7.71E-09	0.986	0.986
ITM2A	1	-0.2977798	2.90E-08	0.00052869	0.465	0.621
AEBP1	1	-0.2998435	1.59E-10	2.90E-06	0.628	0.767
SNRPN	1	-0.3009782	3.04E-15	5.55E-11	0.614	0.788
RNASET2	1	-0.301089	6.21E-21	1.13E-16	0.207	0.486
B2M	1	-0.3023766	2.61E-19	4.77E-15	0.977	0.993
CEBPD	1	-0.3037404	4.04E-07	0.00737928	0.638	0.712
FGFRL1	1	-0.3037887	1.41E-16	2.57E-12	0.163	0.393
SERPING1	1	-0.3045689	1.47E-08	0.00026798	0.519	0.628
PABPC1	1	-0.3059602	6.55E-16	1.20E-11	0.694	0.854
TCF4	1	-0.3061024	1.07E-19	1.96E-15	0.12	0.368
HSP90B1	1	-0.3089463	4.74E-16	8.65E-12	0.725	0.884
AP3S1	1	-0.3089617	6.95E-12	1.27E-07	0.661	0.806
PCSK1N	1	-0.3106219	6.29E-08	0.00114715	0.221	0.365
EEF1A1	1	-0.3133508	1.59E-34	2.89E-30	1	1
CYR61	1	-0.3160974	4.16E-11	7.59E-07	0.141	0.311
OMD	1	-0.3163039	8.66E-15	1.58E-10	0.087	0.274
CANX	1	-0.3180385	4.51E-18	8.22E-14	0.583	0.799
NAA38	1	-0.3209497	1.99E-16	3.64E-12	0.531	0.749
TMEM230	1	-0.3232514	3.78E-16	6.90E-12	0.527	0.715
LAGE3	1	-0.3233549	4.28E-18	7.81E-14	0.366	0.626
UGP2	1	-0.3242305	1.76E-14	3.20E-10	0.448	0.648
DNM3OS	1	-0.3244886	4.19E-15	7.64E-11	0.095	0.285
RCN2	1	-0.3245784	8.29E-13	1.51E-08	0.486	0.669
SDC2	1	-0.3253113	3.21E-15	5.86E-11	0.703	0.863
P4HA1	1	-0.3261272	2.50E-16	4.57E-12	0.562	0.758
SLC39A8	1	-0.3267634	8.30E-18	1.51E-13	0.25	0.514
SUMF2	1	-0.3273409	3.33E-17	6.07E-13	0.324	0.562
PPP3CA	1	-0.3311043	6.66E-11	1.21E-06	0.864	0.929
CDO1	1	-0.3324818	2.03E-15	3.70E-11	0.12	0.324
PEBP1	1	-0.334822	4.47E-17	8.16E-13	0.638	0.808
NDUFA4L2	1	-0.3372088	2.36E-40	4.31E-36	1	1
CHID1	1	-0.3401035	1.56E-20	2.85E-16	0.374	0.653
AK1	1	-0.3408888	3.87E-19	7.06E-15	0.736	0.906
TTC3	1	-0.3414086	1.03E-17	1.88E-13	0.415	0.671
RNF130	1	-0.3415982	2.18E-17	3.97E-13	0.382	0.619

PKM	1	-0.343078	1.97E-12	3.60E-08	0.874	0.925
MALAT1	1	-0.3431912	5.51E-24	1.01E-19	1	1
BHLHE41	1	-0.3441655	1.34E-18	2.45E-14	0.178	0.429
TRPS1	1	-0.3469879	1.27E-20	2.32E-16	0.172	0.438
CTSF	1	-0.348894	1.09E-21	2.00E-17	0.172	0.441
PPP1R3C	1	-0.3490105	2.45E-14	4.47E-10	0.269	0.489
TM2D1	1	-0.3499901	6.18E-18	1.13E-13	0.432	0.664
SLC26A2	1	-0.3516295	1.47E-19	2.69E-15	0.062	0.272
LUM	1	-0.3525187	8.78E-10	1.60E-05	1	1
PRDX1	1	-0.353205	2.12E-17	3.86E-13	0.733	0.847
MT1E	1	-0.3535783	5.71E-13	1.04E-08	0.934	0.966
FKBP2	1	-0.3536685	5.34E-21	9.75E-17	0.483	0.765
FTL	1	-0.3542422	1.56E-37	2.84E-33	1	1
PRDX4	1	-0.3545486	3.67E-18	6.70E-14	0.709	0.87
SNAI2	1	-0.3555209	4.76E-13	8.68E-09	0.207	0.409
ITM2B	1	-0.3568961	3.48E-17	6.34E-13	0.971	0.991
PCOLCE2	1	-0.3587698	3.80E-23	6.93E-19	0.907	0.977
DYNLL1	1	-0.3606226	2.46E-17	4.49E-13	0.752	0.877
INHBA	1	-0.3614335	1.25E-15	2.28E-11	0.126	0.336
LSP1	1	-0.3615881	2.46E-20	4.49E-16	0.141	0.39
GAS6	1	-0.3629015	4.96E-27	9.05E-23	0.134	0.441
IER2	1	-0.3651374	4.68E-15	8.54E-11	0.14	0.349
SMOC2	1	-0.3658495	2.90E-24	5.28E-20	0.066	0.32
ID2	1	-0.3695119	2.98E-11	5.44E-07	0.24	0.42
ISLR	1	-0.3719431	8.23E-25	1.50E-20	0.13	0.416
CAPS	1	-0.3743494	7.43E-11	1.36E-06	0.618	0.733
SAT1	1	-0.3778584	2.23E-17	4.06E-13	0.965	0.991
HIF1A	1	-0.3792654	3.80E-21	6.94E-17	0.808	0.945
SDF4	1	-0.3817543	2.25E-21	4.11E-17	0.463	0.719
GSN	1	-0.3835165	1.33E-16	2.43E-12	0.364	0.596
LCN2	1	-0.3835969	1.20E-07	0.00219419	0.49	0.626
SERF2	1	-0.3858703	6.74E-36	1.23E-31	1	1
GRN	1	-0.3873744	7.67E-23	1.40E-18	0.105	0.363
SOX9	1	-0.3897396	1.91E-14	3.49E-10	0.566	0.756
MT-CO3	1	-0.3908667	2.04E-13	3.73E-09	0.781	0.84
EGLN3	1	-0.3935631	1.39E-12	2.54E-08	0.281	0.482
MT-ND4	1	-0.3958562	2.17E-19	3.97E-15	0.915	0.963
CD59	1	-0.3962369	4.94E-25	9.01E-21	0.905	0.968
PLD3	1	-0.397293	3.86E-21	7.05E-17	0.36	0.632

FGFBP2	1	-0.3978831	3.12E-13	5.70E-09	0.988	1
TSPAN3	1	-0.3986957	6.62E-20	1.21E-15	0.318	0.566
CALR	1	-0.4006963	1.58E-16	2.88E-12	0.502	0.721
SPON2	1	-0.4018261	2.23E-24	4.07E-20	0.083	0.345
ID4	1	-0.4042367	8.20E-17	1.50E-12	0.221	0.459
FCGRT	1	-0.405476	3.70E-22	6.75E-18	0.434	0.678
ANGPTL2	1	-0.4064283	6.37E-21	1.16E-16	0.277	0.55
CRIP1	1	-0.4083239	6.34E-26	1.16E-21	0.221	0.546
P4HB	1	-0.4090103	5.65E-21	1.03E-16	0.62	0.813
RHOBTB3	1	-0.4115224	1.16E-22	2.12E-18	0.264	0.553
CA9	1	-0.4117492	1.80E-10	3.28E-06	0.428	0.58
TMED2	1	-0.4143042	3.64E-23	6.65E-19	0.576	0.813
GAPDH	1	-0.4158157	3.77E-10	6.87E-06	1	1
FXYD1	1	-0.4208413	4.81E-22	8.78E-18	0.293	0.564
MT-ND3	1	-0.4227111	1.66E-18	3.02E-14	0.331	0.587
POLR2L	1	-0.4291621	2.80E-25	5.10E-21	0.758	0.884
EID1	1	-0.4292744	4.52E-33	8.25E-29	0.767	0.92
DDR2	1	-0.4321518	2.32E-25	4.24E-21	0.262	0.559
CD320	1	-0.4372985	9.59E-30	1.75E-25	0.314	0.653
EGR1	1	-0.437438	1.60E-19	2.92E-15	0.182	0.438
SRGN	1	-0.4440567	1.98E-22	3.61E-18	0.207	0.484
TPD52	1	-0.4468798	1.92E-24	3.51E-20	0.601	0.817
TGFBI	1	-0.4513802	5.91E-09	0.00010784	0.556	0.699
C1S	1	-0.4538892	4.28E-23	7.81E-19	0.49	0.733
SCRG1	1	-0.4557968	8.27E-20	1.51E-15	0.994	1
COL9A2	1	-0.4567341	2.26E-22	4.12E-18	0.229	0.505
PDPN	1	-0.4572842	2.17E-15	3.95E-11	0.471	0.664
LY6E	1	-0.4575282	3.93E-29	7.17E-25	0.32	0.637
DCN	1	-0.4595617	5.51E-35	1.01E-30	1	1
CNPY2	1	-0.4604392	1.26E-27	2.30E-23	0.444	0.731
MDFI	1	-0.4652836	1.82E-23	3.32E-19	0.2	0.491
DDIT4	1	-0.4688271	1.34E-14	2.45E-10	0.32	0.534
MT-CYB	1	-0.4708893	3.27E-18	5.97E-14	0.372	0.619
MT-CO1	1	-0.478909	1.01E-19	1.84E-15	0.895	0.947
MGP	1	-0.4823187	6.17E-13	1.13E-08	0.994	0.998
VCAN	1	-0.4896295	1.37E-20	2.50E-16	0.231	0.489
CST3	1	-0.4937845	8.53E-42	1.56E-37	0.99	1
LRPAP1	1	-0.4943231	1.45E-27	2.65E-23	0.366	0.664
HTRA1	1	-0.5006409	7.34E-32	1.34E-27	0.665	0.897

PAX1	1	-0.5036897	2.05E-23	3.74E-19	0.32	0.594
IGF2	1	-0.5145889	9.80E-16	1.79E-11	0.136	0.347
COL11A2	1	-0.5153307	2.93E-33	5.35E-29	0.36	0.712
C7orf73	1	-0.5191111	1.99E-27	3.63E-23	0.504	0.76
PDIA3	1	-0.519566	3.51E-31	6.40E-27	0.362	0.687
PNRC1	1	-0.5282422	5.90E-26	1.08E-21	0.868	0.934
VKORC1	1	-0.5327779	2.14E-46	3.90E-42	0.934	0.993
FBXO2	1	-0.534584	2.58E-40	4.71E-36	0.541	0.858
FMOD	1	-0.5349739	5.28E-40	9.64E-36	0.88	0.979
PDIA6	1	-0.5353691	3.31E-36	6.04E-32	0.38	0.726
CHI3L2	1	-0.5372534	1.19E-20	2.17E-16	0.628	0.865
ATRAID	1	-0.5377865	6.26E-44	1.14E-39	0.707	0.936
MT-ATP6	1	-0.5417496	3.36E-20	6.13E-16	0.558	0.756
NGFRAP1	1	-0.54749	2.74E-40	5.00E-36	0.694	0.909
DUSP1	1	-0.5537798	7.40E-21	1.35E-16	0.269	0.53
MT-ND1	1	-0.5549901	6.18E-29	1.13E-24	0.242	0.584
OGN	1	-0.5651329	1.36E-18	2.49E-14	0.841	0.938
MT-ND2	1	-0.5756701	7.00E-29	1.28E-24	0.266	0.603
FOS	1	-0.5879382	9.45E-27	1.72E-22	0.372	0.653
S100A4	1	-0.5974765	8.38E-20	1.53E-15	0.671	0.813
MT1H	1	-0.5984093	2.40E-10	4.37E-06	0.634	0.76
CFD	1	-0.6015319	2.97E-18	5.41E-14	0.095	0.308
COL9A3	1	-0.6040655	1.27E-22	2.31E-18	0.455	0.724
WWP2	1	-0.6050547	2.95E-35	5.38E-31	0.545	0.82
FAM46A	1	-0.6150434	3.44E-36	6.28E-32	0.391	0.735
FXYD6	1	-0.6152286	1.43E-43	2.62E-39	0.554	0.868
ID1	1	-0.615388	2.23E-21	4.07E-17	0.211	0.484
PAM	1	-0.6242629	9.22E-50	1.68E-45	0.403	0.806
GOLIM4	1	-0.6304335	1.17E-23	2.14E-19	0.215	0.495
NOVA1	1	-0.6305609	1.37E-21	2.51E-17	0.816	0.916
CD99	1	-0.6743575	1.49E-67	2.71E-63	0.839	0.989
C2orf82	1	-0.6786551	8.35E-14	1.52E-09	0.973	0.984
CHI3L1	1	-0.6869478	1.29E-16	2.36E-12	0.944	0.977
JUNB	1	-0.6883931	3.59E-27	6.54E-23	0.331	0.651
MT-CO2	1	-0.7257773	4.49E-35	8.19E-31	0.824	0.945
BAMBI	1	-0.727267	7.23E-37	1.32E-32	0.428	0.779
IBSP	1	-0.7322221	1.44E-14	2.63E-10	0.012	0.139
ITM2C	1	-0.7394345	1.15E-55	2.10E-51	0.234	0.694
LECT1	1	-0.7457296	9.93E-42	1.81E-37	0.736	0.95

PRELP	1	-0.7731635	2.25E-52	4.11E-48	0.948	0.995
COL11A1	1	-0.782567	5.67E-50	1.03E-45	0.496	0.877
S100B	1	-0.7921066	2.08E-41	3.79E-37	0.634	0.847
ID3	1	-0.7948066	4.20E-24	7.66E-20	0.147	0.422
RBP4	1	-0.8130182	5.42E-43	9.90E-39	0.655	0.906
IGFBP6	1	-0.8284032	6.27E-36	1.14E-31	0.541	0.815
CP	1	-0.8294788	1.41E-40	2.58E-36	0.359	0.721
CLEC3A	1	-0.842016	8.94E-33	1.63E-28	0.233	0.587
C2orf40	1	-0.8611738	5.02E-44	9.16E-40	0.986	1
MT1F	1	-0.8722134	2.69E-38	4.91E-34	0.839	0.957
COL2A1	1	-0.8800457	3.38E-43	6.16E-39	0.326	0.717
TSC22D1	1	-0.8880453	3.84E-39	7.00E-35	0.45	0.779
CYTL1	1	-0.8949876	1.46E-23	2.67E-19	0.159	0.443
PLA2G2A	1	-0.9150473	4.49E-25	8.19E-21	0.388	0.708
MT1G	1	-0.9739522	2.17E-33	3.96E-29	0.93	0.977
ACAN	1	-0.9765802	6.43E-74	1.17E-69	0.847	0.991
EPYC	1	-0.9798985	2.00E-54	3.65E-50	0.093	0.539
PLAC9	1	-0.9943381	9.76E-60	1.78E-55	0.517	0.886
CCDC80	1	-1.0314464	5.73E-65	1.05E-60	0.876	0.995
SLPI	1	-1.4488676	1.38E-37	2.51E-33	0.891	0.993
CTSD	1	-1.5330578	3.31E-85	6.04E-81	0.767	0.989

gene	cluster	avg_logFC	p_val	p_val_adj	pct.1	pct.2
HMGA1	2	0.99486752	9.36E-63	1.71E-58	0.831	0.353
SPP1	2	0.94264852	4.56E-17	8.33E-13	0.862	0.638
CD55	2	0.73743895	3.03E-46	5.53E-42	0.892	0.611
MT2A	2	0.71945726	1.00E-47	1.83E-43	1	1
RGCC	2	0.62089395	6.88E-32	1.26E-27	0.939	0.724
TM4SF1	2	0.58690281	1.01E-33	1.84E-29	0.818	0.443
CTSL	2	0.54333226	1.57E-22	2.87E-18	0.877	0.622
UPP1	2	0.44800959	2.52E-26	4.60E-22	0.809	0.468
TIMP3	2	0.44688912	6.22E-14	1.14E-09	0.689	0.486
C11orf96	2	0.43376593	2.07E-17	3.78E-13	0.492	0.231
C15orf48	2	0.43003336	1.56E-09	2.86E-05	0.256	0.106
GSTO1	2	0.42849938	5.17E-22	9.44E-18	0.845	0.615
TMSB10	2	0.42022589	7.26E-20	1.32E-15	0.998	0.993
NT5E	2	0.4061562	4.48E-20	8.17E-16	0.532	0.26
SNHG12	2	0.37193817	1.53E-15	2.80E-11	0.678	0.466
S100A16	2	0.36347184	1.63E-14	2.98E-10	0.502	0.283
NRP2	2	0.35636856	1.48E-08	0.00027047	0.519	0.369

S100A10	2	0.34467236	8.72E-16	1.59E-11	0.981	0.955
TNFRSF12A	2	0.34373302	8.15E-19	1.49E-14	0.71	0.4
TGM2	2	0.3354576	1.03E-15	1.87E-11	0.511	0.274
EIF1	2	0.33143017	1.06E-25	1.94E-21	1	1
ZFAS1	2	0.3142574	3.48E-21	6.35E-17	0.996	0.998
STK17A	2	0.30854346	4.16E-13	7.58E-09	0.638	0.396
FNIP2	2	0.3082838	4.28E-14	7.80E-10	0.468	0.253
SH3BGRL3	2	0.29792724	6.63E-19	1.21E-14	0.996	0.989
IER3	2	0.2973654	6.73E-09	0.0001228	0.445	0.274
RGS10	2	0.29390401	1.60E-11	2.93E-07	0.612	0.446
SMOX	2	0.29196692	2.96E-11	5.40E-07	0.504	0.321
VIMP	2	0.28868879	9.20E-12	1.68E-07	0.79	0.643
LRRFIP1	2	0.28453333	3.50E-12	6.38E-08	0.494	0.292
AKAP12	2	0.28251322	2.07E-14	3.78E-10	0.246	0.066
AC090498.1	2	0.28096012	1.88E-35	3.43E-31	1	1
S100A6	2	0.28076771	7.01E-09	0.00012783	1	1
EPB41L4A-AS1	2	0.28026944	4.78E-13	8.73E-09	0.915	0.819
GYPC	2	0.27248679	5.22E-08	0.00095302	0.576	0.437
PSMA7	2	0.27104751	1.54E-11	2.82E-07	0.858	0.747
PLP2	2	0.26091108	2.99E-09	5.45E-05	0.799	0.681
FXYD5	2	0.25078022	1.33E-12	2.42E-08	0.93	0.792
LINC00152	2	0.25038942	2.99E-07	0.00545743	0.388	0.247
FKBP10	2	-0.2507879	4.19E-14	7.65E-10	0.191	0.421
HLA-C	2	-0.2508644	1.28E-08	0.00023285	0.725	0.835
TTC3	2	-0.2530032	4.09E-10	7.46E-06	0.403	0.595
CNPY2	2	-0.2535177	1.86E-10	3.40E-06	0.483	0.661
N4BP2L2	2	-0.2563859	8.98E-10	1.64E-05	0.585	0.742
WFDC2	2	-0.2564581	1.51E-21	2.76E-17	0.028	0.24
NME4	2	-0.2575863	1.02E-11	1.85E-07	0.222	0.423
WSB1	2	-0.2600481	4.58E-12	8.35E-08	0.722	0.837
DUT	2	-0.2601222	6.67E-12	1.22E-07	0.441	0.629
SH3BGRL	2	-0.2618515	3.03E-14	5.54E-10	0.216	0.448
WBP5	2	-0.2618798	9.24E-10	1.69E-05	0.644	0.765
CHID1	2	-0.2619806	1.12E-14	2.04E-10	0.244	0.486
HSP90B1	2	-0.2620135	3.50E-09	6.39E-05	0.608	0.74
ID4	2	-0.2623794	7.65E-07	0.0139506	0.278	0.419
SUMF2	2	-0.2635364	4.67E-14	8.52E-10	0.22	0.45
NAMPT	2	-0.2642356	5.35E-10	9.76E-06	0.481	0.633
NT5C3A	2	-0.2657474	2.98E-10	5.45E-06	0.214	0.398

ISLR	2	-0.2666949	5.59E-20	1.02E-15	0.07	0.303
TPD52L1	2	-0.2676104	4.73E-13	8.63E-09	0.169	0.373
STK26	2	-0.2679824	3.04E-13	5.55E-09	0.138	0.335
TPI1	2	-0.269277	2.20E-12	4.02E-08	0.987	0.993
PABPC1	2	-0.2701021	8.43E-13	1.54E-08	0.735	0.846
IFI6	2	-0.2711621	1.73E-09	3.16E-05	0.148	0.312
PAPSS2	2	-0.2732237	1.71E-10	3.11E-06	0.364	0.561
RAB1A	2	-0.2735368	3.66E-10	6.67E-06	0.646	0.769
FAM162A	2	-0.2736069	1.02E-10	1.85E-06	0.915	0.959
NOL3	2	-0.2741953	1.61E-10	2.95E-06	0.254	0.437
BTG1	2	-0.2747505	9.40E-09	0.00017154	0.977	0.982
ATPIF1	2	-0.2757784	6.30E-11	1.15E-06	0.445	0.622
FXYD1	2	-0.2769782	1.35E-14	2.47E-10	0.146	0.357
AVPI1	2	-0.2773879	1.45E-14	2.64E-10	0.161	0.385
IFRD1	2	-0.2778935	3.10E-10	5.65E-06	0.462	0.629
NDUFA5	2	-0.2787252	4.72E-13	8.62E-09	0.447	0.654
SRGN	2	-0.2792432	9.61E-12	1.75E-07	0.148	0.33
C7orf73	2	-0.2798754	2.80E-07	0.00510102	0.578	0.686
COL9A2	2	-0.2803051	1.71E-13	3.13E-09	0.11	0.303
TPD52	2	-0.2805714	2.06E-10	3.76E-06	0.534	0.676
POLR2L	2	-0.2807805	6.08E-10	1.11E-05	0.701	0.842
HMGN2	2	-0.2809938	3.30E-15	6.02E-11	0.758	0.891
IGF2	2	-0.2836915	7.15E-10	1.31E-05	0.057	0.188
CD320	2	-0.2839227	1.05E-13	1.91E-09	0.246	0.459
H3F3B	2	-0.2847878	6.53E-17	1.19E-12	0.996	1
PLXDC2	2	-0.2863095	1.05E-13	1.92E-09	0.256	0.475
SEPP1	2	-0.2870285	4.07E-16	7.43E-12	0.144	0.373
TCEAL8	2	-0.287204	2.75E-14	5.02E-10	0.47	0.695
GPX3	2	-0.2876661	3.99E-10	7.29E-06	0.841	0.93
PRDX2	2	-0.2898549	6.73E-12	1.23E-07	0.559	0.71
UBC	2	-0.2900971	4.91E-08	0.00089518	0.841	0.923
HNRNPA1	2	-0.2916168	8.47E-16	1.55E-11	0.975	0.989
CALD1	2	-0.2948391	4.95E-13	9.03E-09	0.379	0.595
PPP3CA	2	-0.2956348	4.49E-09	8.19E-05	0.881	0.934
SERPINA5	2	-0.2959422	7.21E-18	1.32E-13	0.189	0.448
MT-ND1	2	-0.2973116	3.40E-07	0.00620037	0.419	0.563
CCNG2	2	-0.2984295	3.81E-11	6.95E-07	0.297	0.484
CRTAC1	2	-0.2998267	5.07E-13	9.25E-09	0.214	0.425
FTL	2	-0.3012558	1.67E-28	3.04E-24	1	1

PNRC1	2	-0.3013109	3.19E-10	5.82E-06	0.913	0.971
MT-ND2	2	-0.3013582	5.76E-11	1.05E-06	0.411	0.6
BHLHE41	2	-0.3044693	1.55E-16	2.83E-12	0.131	0.355
SPON2	2	-0.3054073	3.42E-17	6.23E-13	0.055	0.251
CDO1	2	-0.3080549	4.03E-17	7.35E-13	0.091	0.308
TMEM59	2	-0.3091202	5.52E-14	1.01E-09	0.949	0.977
MT-CO2	2	-0.3099461	3.59E-07	0.00654898	0.919	0.946
CTSF	2	-0.3101224	2.12E-22	3.87E-18	0.085	0.344
PRDX4	2	-0.3136859	2.43E-12	4.43E-08	0.553	0.706
PTP4A1	2	-0.3136923	4.94E-07	0.00901103	0.451	0.586
MT-ATP6	2	-0.3137998	1.46E-07	0.00266622	0.71	0.794
ID2	2	-0.3147163	2.32E-09	4.23E-05	0.324	0.502
LRPAP1	2	-0.3164585	3.49E-14	6.38E-10	0.265	0.482
BNIP3	2	-0.3201605	4.04E-11	7.37E-07	0.998	0.998
PGK1	2	-0.3205727	5.06E-13	9.24E-09	0.909	0.966
GSN	2	-0.3211779	2.52E-16	4.60E-12	0.227	0.466
PEBP1	2	-0.3215362	1.91E-15	3.48E-11	0.523	0.744
HLA-A	2	-0.3220492	2.66E-11	4.85E-07	0.754	0.851
SNRPN	2	-0.3257204	2.74E-17	5.00E-13	0.504	0.731
NEAT1	2	-0.3280959	3.06E-12	5.58E-08	0.972	0.977
IFITM2	2	-0.3312123	1.30E-13	2.38E-09	0.769	0.891
ITM2B	2	-0.3354813	1.04E-17	1.90E-13	0.917	0.984
COL6A2	2	-0.3382809	4.47E-16	8.16E-12	0.309	0.545
PPP1R3C	2	-0.340993	7.05E-13	1.29E-08	0.25	0.45
SERPING1	2	-0.3442315	8.30E-17	1.51E-12	0.32	0.575
SERPINA1	2	-0.3448295	6.00E-20	1.10E-15	0.919	0.989
EEF1A1	2	-0.3490571	6.30E-42	1.15E-37	1	1
PSAP	2	-0.3502027	2.30E-19	4.19E-15	0.761	0.923
EID1	2	-0.3504568	1.44E-22	2.62E-18	0.797	0.932
TGFBI	2	-0.3517776	8.62E-12	1.57E-07	0.443	0.64
EEF2	2	-0.3528421	2.43E-19	4.44E-15	0.962	0.98
IFITM1	2	-0.3603491	2.70E-13	4.93E-09	0.311	0.525
P4HB	2	-0.3630106	9.24E-17	1.69E-12	0.508	0.729
ATRAID	2	-0.3640149	1.65E-18	3.01E-14	0.636	0.819
CRIP1	2	-0.365585	2.71E-22	4.95E-18	0.157	0.448
DUSP1	2	-0.3704346	3.71E-12	6.76E-08	0.328	0.529
SERF2	2	-0.3713748	9.12E-28	1.66E-23	0.998	1
CEBPD	2	-0.3740063	8.68E-10	1.58E-05	0.619	0.731
FAM46A	2	-0.3774249	2.28E-15	4.16E-11	0.364	0.584

MMP3	2	-0.3776968	5.20E-13	9.48E-09	0.814	0.921
LSP1	2	-0.3785707	3.26E-25	5.94E-21	0.059	0.326
MFGE8	2	-0.3835727	2.76E-16	5.04E-12	0.608	0.79
NAA38	2	-0.3928239	6.38E-22	1.16E-17	0.434	0.695
NUCB1	2	-0.3935287	1.60E-23	2.91E-19	0.318	0.606
B2M	2	-0.3946481	1.06E-24	1.94E-20	0.96	0.977
LY6E	2	-0.3950908	1.84E-25	3.36E-21	0.208	0.525
NNMT	2	-0.3962421	1.68E-13	3.06E-09	0.807	0.916
PRKAR1A	2	-0.3989658	3.50E-21	6.38E-17	0.407	0.686
PKM	2	-0.3990706	2.27E-18	4.15E-14	0.818	0.914
CA9	2	-0.4011556	2.27E-16	4.13E-12	0.316	0.543
S100A4	2	-0.4016938	4.36E-15	7.95E-11	0.547	0.749
DDIT4	2	-0.4038703	1.70E-12	3.10E-08	0.32	0.52
MT1G	2	-0.4053098	1.74E-23	3.18E-19	1	1
IFITM3	2	-0.4062538	1.32E-18	2.41E-14	0.803	0.928
VKORC1	2	-0.4065957	1.73E-23	3.16E-19	0.862	0.971
RHOBTB3	2	-0.4065957	3.48E-15	6.35E-11	0.307	0.527
CAPS	2	-0.4084772	5.53E-09	0.00010092	0.468	0.624
DDR2	2	-0.4123629	3.99E-20	7.28E-16	0.195	0.455
PLD3	2	-0.416326	1.23E-25	2.25E-21	0.197	0.509
SDC2	2	-0.4165404	1.17E-21	2.13E-17	0.54	0.787
PAX1	2	-0.4231002	2.81E-18	5.12E-14	0.28	0.529
FBXO2	2	-0.4237079	2.00E-22	3.65E-18	0.426	0.692
C1S	2	-0.4237697	2.49E-22	4.55E-18	0.386	0.665
MDFI	2	-0.424837	9.90E-16	1.81E-11	0.248	0.466
ITM2C	2	-0.426364	2.49E-23	4.54E-19	0.155	0.439
SLC39A8	2	-0.4278473	7.06E-24	1.29E-19	0.201	0.493
VCAN	2	-0.429697	3.00E-26	5.47E-22	0.106	0.405
NDUFA4L2	2	-0.4326996	4.99E-58	9.11E-54	1	1
EGLN3	2	-0.4331445	1.26E-17	2.31E-13	0.235	0.475
ID1	2	-0.4391688	1.78E-07	0.00324002	0.347	0.491
EGR1	2	-0.4402676	5.25E-11	9.57E-07	0.233	0.416
IGFBP7	2	-0.4409393	1.87E-24	3.42E-20	0.585	0.855
TSC22D1	2	-0.4420342	1.12E-08	0.00020403	0.5	0.647
SAT1	2	-0.4424844	5.77E-23	1.05E-18	0.839	0.977
CD99	2	-0.4501471	9.23E-29	1.68E-24	0.727	0.912
COL11A2	2	-0.4515257	1.80E-22	3.29E-18	0.218	0.505
HTRA1	2	-0.4623665	2.05E-23	3.75E-19	0.487	0.756
PAM	2	-0.4818295	8.47E-28	1.55E-23	0.324	0.64

NGFRAP1	2	-0.4907737	4.73E-29	8.63E-25	0.572	0.837
IGFBP6	2	-0.4923011	8.56E-10	1.56E-05	0.39	0.554
FOS	2	-0.4987636	1.11E-18	2.03E-14	0.407	0.636
CFD	2	-0.5063629	3.18E-25	5.81E-21	0.044	0.299
MALAT1	2	-0.5106556	3.26E-40	5.95E-36	1	1
HIF1A	2	-0.5132044	3.94E-34	7.19E-30	0.659	0.928
GAPDH	2	-0.5132542	3.57E-24	6.51E-20	1	1
PCOLCE2	2	-0.5140652	1.79E-33	3.27E-29	0.744	0.939
CLEC3A	2	-0.5198424	2.32E-18	4.24E-14	0.112	0.355
ADM	2	-0.5201476	1.82E-12	3.32E-08	0.699	0.803
CP	2	-0.53686	8.25E-15	1.51E-10	0.485	0.658
SCRG1	2	-0.5448685	9.32E-19	1.70E-14	0.913	0.975
FXYD6	2	-0.5468005	5.93E-28	1.08E-23	0.498	0.758
NOVA1	2	-0.5485681	1.63E-15	2.98E-11	0.748	0.878
JUNB	2	-0.5545899	3.44E-14	6.28E-10	0.481	0.656
COL9A3	2	-0.57057	4.42E-30	8.07E-26	0.227	0.59
WWP2	2	-0.5806964	4.43E-29	8.08E-25	0.383	0.683
CST3	2	-0.5939968	4.70E-48	8.58E-44	0.947	0.998
IGFBP5	2	-0.5994842	1.29E-27	2.35E-23	0.061	0.344
CLU	2	-0.6029546	2.84E-32	5.18E-28	0.996	1
BAMBI	2	-0.6098398	1.85E-25	3.38E-21	0.324	0.618
MT1F	2	-0.6178799	1.62E-26	2.95E-22	0.975	0.995
COL2A1	2	-0.6247497	9.38E-23	1.71E-18	0.254	0.541
ID3	2	-0.635848	2.41E-13	4.39E-09	0.214	0.414
EPYC	2	-0.6359494	6.53E-34	1.19E-29	0.04	0.351
COL11A1	2	-0.6385128	5.50E-39	1.00E-34	0.297	0.706
LUM	2	-0.6407837	7.97E-35	1.45E-30	1	1
FGFBP2	2	-0.6472095	1.61E-28	2.93E-24	0.883	0.968
FMOD	2	-0.6543969	5.41E-48	9.88E-44	0.627	0.923
LCN2	2	-0.6783307	5.69E-18	1.04E-13	0.337	0.593
RBP4	2	-0.6827018	5.07E-26	9.25E-22	0.413	0.722
CHI3L1	2	-0.7039159	6.28E-26	1.15E-21	0.886	0.986
PLAC9	2	-0.7042281	2.87E-30	5.23E-26	0.511	0.796
C2orf40	2	-0.7267669	3.79E-40	6.92E-36	0.928	0.998
S100B	2	-0.7471072	1.10E-35	2.01E-31	0.436	0.749
DCN	2	-0.7597277	3.73E-69	6.81E-65	1	1
ACAN	2	-0.7691965	1.18E-40	2.15E-36	0.752	0.946
OGN	2	-0.7928418	6.10E-31	1.11E-26	0.606	0.894
LECT1	2	-0.9203668	7.32E-58	1.34E-53	0.394	0.86

PRELP	2	-0.9640905	2.48E-64	4.53E-60	0.771	0.975
CHI3L2	2	-0.9926551	3.73E-54	6.81E-50	0.544	0.928
CCDC80	2	-1.1889475	2.41E-71	4.39E-67	0.72	0.982
PLA2G2A	2	-1.2164005	3.92E-45	7.16E-41	0.288	0.749
CTSD	2	-1.2603706	8.52E-77	1.56E-72	0.682	0.991
SLPI	2	-1.5795046	2.06E-47	3.76E-43	0.824	0.98
**gene**	**cluster**	**avg_logFC**	**p_val**	**p_val_adj**	**pct.1**	**pct.2**
CXCL8	3	1.27483819	2.15E-12	3.93E-08	0.409	0.173
TFPI2	3	0.7575551	2.12E-08	0.00038654	0.276	0.114
CD55	3	0.70548193	1.54E-31	2.81E-27	0.919	0.697
HMGA1	3	0.66518018	1.03E-23	1.89E-19	0.838	0.596
C11orf96	3	0.60821777	1.57E-17	2.86E-13	0.684	0.42
PRG4	3	0.59553837	2.81E-08	0.0005122	0.722	0.537
RGCC	3	0.54921235	3.84E-16	7.01E-12	0.862	0.651
IER3	3	0.53878941	6.90E-09	0.00012582	0.691	0.511
TIMP3	3	0.46167471	1.26E-10	2.30E-06	0.67	0.479
NRP2	3	0.42074561	9.69E-07	0.01769043	0.679	0.573
TMSB4X	3	0.39671274	8.84E-08	0.00161316	0.85	0.687
SLC7A2	3	0.31508244	9.64E-08	0.00175829	0.698	0.518
FNIP2	3	0.31504333	2.02E-12	3.69E-08	0.587	0.339
MT2A	3	0.31159267	1.31E-10	2.39E-06	1	1
TIMP2	3	0.29198604	1.33E-09	2.42E-05	0.715	0.544
FAP	3	0.28548114	2.20E-11	4.02E-07	0.435	0.202
GPX4	3	0.27320178	2.80E-12	5.11E-08	0.998	0.99
RHOC	3	0.26673729	6.29E-08	0.00114859	0.836	0.678
AKAP12	3	0.25389723	1.77E-06	0.0323761	0.259	0.124
RABGGTB	3	0.25281605	7.01E-09	0.00012797	0.874	0.818
CTSF	3	-0.2516606	6.86E-11	1.25E-06	0.133	0.322
CALR	3	-0.2565085	5.29E-07	0.00965683	0.411	0.567
GLTSCR2	3	-0.2567217	2.48E-12	4.53E-08	0.85	0.935
SNRPN	3	-0.259933	2.95E-09	5.39E-05	0.575	0.71
TPI1	3	-0.2608663	4.83E-10	8.81E-06	0.974	0.987
FBXO2	3	-0.2613198	6.31E-07	0.01151963	0.435	0.573
EEF1A1	3	-0.262415	4.64E-21	8.47E-17	1	1
ITM2B	3	-0.2638664	2.66E-09	4.85E-05	0.952	0.964
B2M	3	-0.2650457	1.44E-12	2.63E-08	0.988	0.993
IGFBP7	3	-0.2656178	5.06E-09	9.23E-05	0.641	0.831
PRDX2	3	-0.2668813	1.66E-07	0.00302263	0.575	0.697
EID1	3	-0.2712945	2.92E-10	5.32E-06	0.781	0.876

COL6A2	3	-0.2718399	1.26E-09	2.30E-05	0.359	0.557
CALD1	3	-0.2724766	2.66E-09	4.85E-05	0.461	0.648
PGK1	3	-0.2812905	2.12E-08	0.00038618	0.905	0.958
ATRAID	3	-0.2815205	5.91E-11	1.08E-06	0.656	0.837
LUM	3	-0.28213	1.17E-09	2.14E-05	1	1
IFITM1	3	-0.2845582	7.26E-09	0.00013253	0.278	0.466
SDF4	3	-0.2847622	2.21E-11	4.04E-07	0.368	0.58
SNAI2	3	-0.2882351	2.43E-07	0.00444181	0.228	0.388
VKORC1	3	-0.2892882	1.08E-10	1.96E-06	0.812	0.893
EEF2	3	-0.2905992	3.40E-12	6.20E-08	0.964	0.99
TGFBI	3	-0.2916552	5.81E-07	0.01060176	0.42	0.58
GSN	3	-0.2932701	3.45E-11	6.29E-07	0.219	0.436
WSB1	3	-0.294884	2.55E-10	4.65E-06	0.746	0.906
FAM46A	3	-0.2987172	3.24E-08	0.0005909	0.366	0.528
WFDC2	3	-0.2998212	6.21E-17	1.13E-12	0.045	0.257
FXYD6	3	-0.3004395	6.82E-10	1.24E-05	0.494	0.684
PPP1R3C	3	-0.3064654	1.42E-12	2.59E-08	0.204	0.436
HTRA1	3	-0.3072328	4.31E-08	0.00078666	0.466	0.655
SOD3	3	-0.3092115	7.69E-09	0.0001403	0.907	0.967
BNIP3	3	-0.3107619	2.55E-11	4.65E-07	0.995	1
SERPINA1	3	-0.3120775	2.08E-09	3.79E-05	0.924	0.967
FGFBP2	3	-0.313862	1.21E-06	0.02202533	0.872	0.958
NAMPT	3	-0.3225256	4.51E-12	8.23E-08	0.651	0.808
CDO1	3	-0.3238496	1.30E-09	2.37E-05	0.133	0.306
HMGN2	3	-0.3261389	2.32E-13	4.23E-09	0.748	0.886
SERPING1	3	-0.3272138	6.40E-11	1.17E-06	0.423	0.632
MFGE8	3	-0.3331768	1.69E-10	3.09E-06	0.615	0.795
COL11A1	3	-0.3364399	1.58E-10	2.88E-06	0.235	0.45
NDUFA4L2	3	-0.3369415	5.58E-27	1.02E-22	1	1
PKM	3	-0.3373531	6.40E-13	1.17E-08	0.822	0.899
ID2	3	-0.3395392	3.21E-08	0.00058658	0.489	0.632
DDIT4L	3	-0.3445284	7.32E-07	0.01335268	0.817	0.899
NGFRAP1	3	-0.3445986	6.21E-11	1.13E-06	0.513	0.691
HLA-A	3	-0.34777	1.79E-11	3.27E-07	0.781	0.883
MAOB	3	-0.3491772	3.31E-13	6.04E-09	0.285	0.528
MT1G	3	-0.3534027	1.83E-06	0.03346782	0.981	0.99
CA9	3	-0.3541945	3.40E-11	6.20E-07	0.318	0.534
COL2A1	3	-0.3587255	1.20E-11	2.19E-07	0.2	0.42
COL9A3	3	-0.3637091	5.73E-12	1.04E-07	0.185	0.404

C1S	3	-0.3669534	1.21E-14	2.21E-10	0.527	0.73
NNMT	3	-0.3738927	1.77E-10	3.23E-06	0.781	0.863
IFITM3	3	-0.382432	7.70E-15	1.40E-10	0.8	0.925
CFD	3	-0.3943754	2.20E-12	4.01E-08	0.095	0.29
PLAC9	3	-0.4136301	2.02E-11	3.69E-07	0.42	0.645
ADM	3	-0.4209936	3.00E-12	5.48E-08	0.72	0.87
HIF1A	3	-0.4220157	7.05E-18	1.29E-13	0.542	0.795
EGR1	3	-0.4501523	2.73E-09	4.99E-05	0.247	0.433
MALAT1	3	-0.4550185	1.48E-26	2.71E-22	1	1
GAPDH	3	-0.4614092	1.18E-23	2.15E-19	1	1
SLC39A8	3	-0.4628927	1.17E-19	2.13E-15	0.361	0.648
FOS	3	-0.4711255	5.97E-11	1.09E-06	0.371	0.557
ACAN	3	-0.4724019	1.81E-16	3.30E-12	0.646	0.857
S100B	3	-0.473642	6.19E-16	1.13E-11	0.392	0.655
CCDC85B	3	-0.4773248	3.09E-12	5.64E-08	0.895	0.948
PCOLCE2	3	-0.4819292	2.01E-24	3.67E-20	0.732	0.928
IFITM2	3	-0.4891425	3.06E-19	5.58E-15	0.743	0.896
JUNB	3	-0.5017376	3.33E-07	0.00607245	0.534	0.658
OGN	3	-0.5033247	9.62E-14	1.76E-09	0.42	0.681
FMOD	3	-0.5082797	2.60E-26	4.75E-22	0.501	0.824
CST3	3	-0.5439275	2.05E-26	3.75E-22	0.931	0.984
MT1F	3	-0.5529759	1.16E-12	2.11E-08	0.929	0.974
RBP4	3	-0.5672658	7.38E-22	1.35E-17	0.34	0.648
LECT1	3	-0.6187715	8.37E-28	1.53E-23	0.259	0.638
CLU	3	-0.6330888	2.35E-26	4.28E-22	0.995	1
G0S2	3	-0.6502804	6.36E-11	1.16E-06	0.496	0.707
C2orf40	3	-0.6619168	2.67E-26	4.87E-22	0.886	0.98
IGFBP5	3	-0.667489	3.45E-22	6.29E-18	0.074	0.358
DCN	3	-0.6931783	1.94E-40	3.54E-36	0.995	1
LCN2	3	-0.7061518	1.88E-10	3.42E-06	0.475	0.642
PLA2G2A	3	-0.7454265	1.59E-26	2.90E-22	0.278	0.678
CHI3L1	3	-0.7460432	2.76E-24	5.03E-20	0.857	0.984
PRELP	3	-0.8379369	8.35E-35	1.52E-30	0.67	0.928
CCDC80	3	-0.8629784	2.23E-28	4.07E-24	0.618	0.866
CHI3L2	3	-0.959927	2.71E-35	4.95E-31	0.705	0.964
CTSD	3	-0.9976897	1.55E-41	2.83E-37	0.72	0.951
SLPI	3	-1.0157929	6.78E-30	1.24E-25	0.765	0.951
**gene**	**cluster**	**avg_logFC**	**p_val**	**p_val_adj**	**pct.1**	**pct.2**
MT2A	4	1.20009219	6.22E-17	1.13E-12	1	1

LINC00152	4	0.89844905	7.46E-16	1.36E-11	0.846	0.559
IGFBP3	4	0.84354733	4.49E-10	8.19E-06	0.615	0.332
MSMP	4	0.76446186	1.65E-06	0.03008542	0.25	0.077
TM4SF1	4	0.71879562	6.61E-15	1.21E-10	0.763	0.409
SERPINE2	4	0.65290243	7.29E-15	1.33E-10	1	1
MIR4435-2HG	4	0.62012205	3.73E-12	6.81E-08	0.673	0.377
TPM1	4	0.61555872	4.23E-11	7.72E-07	0.821	0.65
LGALS1	4	0.61236611	3.34E-15	6.10E-11	0.994	0.991
RND3	4	0.5915209	1.06E-07	0.00193966	0.436	0.214
NT5E	4	0.55160086	7.52E-15	1.37E-10	0.679	0.323
IER3	4	0.54184145	2.30E-07	0.00419949	0.449	0.214
HMGA1	4	0.53466633	2.81E-10	5.13E-06	0.423	0.145
CTSL	4	0.50251993	3.22E-07	0.0058698	0.622	0.395
GSTO1	4	0.49337676	1.18E-08	0.00021607	0.686	0.445
TNFRSF12A	4	0.48164133	7.33E-10	1.34E-05	0.673	0.373
FHL2	4	0.48067562	1.38E-10	2.52E-06	0.429	0.159
UPP1	4	0.47238892	1.18E-07	0.00214836	0.513	0.273
VMP1	4	0.46804471	4.52E-09	8.24E-05	0.821	0.659
TMSB10	4	0.44698209	1.12E-08	0.0002044	1	0.995
FN1	4	0.42687964	7.25E-12	1.32E-07	1	1
TGM2	4	0.39742425	1.27E-08	0.00023127	0.353	0.123
TNC	4	0.39469163	1.11E-10	2.03E-06	0.455	0.164
STK17A	4	0.38875664	2.83E-07	0.00516482	0.474	0.236
GYPC	4	0.38690491	7.85E-08	0.00143296	0.628	0.382
COL6A3	4	0.37094712	1.87E-07	0.00340647	0.622	0.405
PLAUR	4	0.37043979	1.14E-07	0.00208372	0.494	0.241
EIF1	4	0.35601404	5.48E-10	1.00E-05	1	1
FAP	4	0.35035051	1.30E-09	2.36E-05	0.558	0.273
PMEPA1	4	0.32983403	2.12E-06	0.03868377	0.455	0.245
BRI3	4	0.32129868	5.65E-09	0.0001031	0.955	0.864
SPP1	4	0.30977393	1.40E-06	0.02556322	0.769	0.582
SLC25A6	4	0.2760128	1.09E-08	0.0001985	0.994	0.964
KIFC3	4	0.27062066	1.92E-09	3.50E-05	0.333	0.091
SGIP1	4	0.26602279	1.32E-07	0.00240319	0.346	0.123
STK26	4	-0.2507605	1.36E-06	0.02479049	0.186	0.427
ANAPC13	4	-0.2652925	2.35E-06	0.04285267	0.301	0.523
IFI16	4	-0.271989	1.09E-06	0.0198245	0.179	0.409
IFITM10	4	-0.2761252	1.01E-06	0.01851559	0.385	0.645
LBH	4	-0.2793318	1.10E-08	0.00020016	0.064	0.309

N4BP2L2	4	-0.2819344	2.64E-06	0.04819604	0.763	0.868
HMGN2	4	-0.2849671	3.50E-07	0.00638967	0.795	0.877
LY6E	4	-0.2859439	5.83E-07	0.01064228	0.506	0.723
PFN2	4	-0.2943159	7.77E-07	0.01418399	0.41	0.645
PCOLCE2	4	-0.2952552	1.25E-06	0.02272063	0.872	0.914
SMIM5	4	-0.2962658	2.18E-08	0.00039839	0.077	0.323
TMEM230	4	-0.301216	2.09E-07	0.00381963	0.776	0.886
POLR2L	4	-0.3022436	4.85E-07	0.00884869	0.942	0.982
SERPINA1	4	-0.3038486	1.17E-09	2.14E-05	0.84	0.973
CNPY2	4	-0.3045007	3.61E-07	0.00658415	0.647	0.832
SRP9	4	-0.3081738	7.61E-09	0.00013885	0.705	0.873
TSPAN3	4	-0.308738	8.42E-07	0.01536648	0.327	0.568
TCEA1	4	-0.3114486	2.82E-07	0.00515471	0.583	0.768
H3F3B	4	-0.3132271	2.34E-10	4.27E-06	1	1
NDUFA4L2	4	-0.3170412	5.52E-16	1.01E-11	1	1
SNRPN	4	-0.3257658	7.09E-08	0.00129333	0.564	0.782
TMEM59	4	-0.3269549	1.82E-07	0.00331552	0.878	0.973
MGP	4	-0.3379242	2.51E-07	0.00457532	0.994	1
NNMT	4	-0.3384704	4.01E-09	7.32E-05	0.91	0.982
TMED10	4	-0.3446363	8.68E-10	1.58E-05	0.763	0.9
CST3	4	-0.3470812	3.98E-11	7.26E-07	0.994	0.991
SPON2	4	-0.3493756	7.02E-09	0.00012811	0.071	0.318
C7orf73	4	-0.3493756	1.95E-08	0.00035519	0.84	0.959
FMOD	4	-0.3587324	1.79E-10	3.26E-06	0.904	0.982
RHOBTB3	4	-0.3762831	8.23E-08	0.00150207	0.494	0.677
ISLR	4	-0.3858213	9.17E-09	0.0001673	0.301	0.582
PAX1	4	-0.3869869	3.23E-07	0.00589096	0.474	0.673
AK1	4	-0.3950018	1.68E-09	3.07E-05	0.699	0.845
FRZB	4	-0.4040341	7.52E-08	0.00137191	0.103	0.336
PRELP	4	-0.4067487	8.79E-11	1.60E-06	0.891	0.986
UGP2	4	-0.4171483	1.25E-09	2.29E-05	0.59	0.8
DNM3OS	4	-0.4208941	4.94E-08	0.00090136	0.462	0.686
RBP4	4	-0.4274822	2.32E-06	0.04232588	0.654	0.841
B2M	4	-0.4284967	3.69E-12	6.74E-08	0.987	0.995
ID4	4	-0.4376682	2.01E-07	0.00367505	0.494	0.705
SOX9	4	-0.4389878	1.19E-08	0.00021717	0.654	0.809
FXYD6	4	-0.4393885	9.80E-12	1.79E-07	0.782	0.941
S100A1	4	-0.4432281	5.25E-14	9.58E-10	0.981	1
PNRC1	4	-0.4463679	2.43E-13	4.43E-09	0.923	0.977

MIA	4	-0.4526779	2.80E-12	5.11E-08	0.962	0.995
FTL	4	-0.4576785	2.67E-21	4.87E-17	1	1
ITM2C	4	-0.4650333	2.16E-09	3.93E-05	0.513	0.764
PAM	4	-0.4681133	6.62E-14	1.21E-09	0.647	0.864
WWP2	4	-0.4745388	3.99E-10	7.29E-06	0.494	0.723
FBXO2	4	-0.4774662	1.47E-12	2.68E-08	0.526	0.805
SNAI2	4	-0.4855078	2.08E-06	0.03796247	0.564	0.718
CAPS	4	-0.4860942	1.56E-07	0.00284717	0.583	0.732
BAMBI	4	-0.4871889	8.00E-10	1.46E-05	0.506	0.768
NGFRAP1	4	-0.4900126	4.10E-15	7.49E-11	0.692	0.932
DCN	4	-0.4933819	5.81E-15	1.06E-10	1	1
PPP1R3C	4	-0.5133106	3.32E-11	6.07E-07	0.365	0.659
ID1	4	-0.5218385	7.66E-10	1.40E-05	0.647	0.886
CRYAB	4	-0.5278266	2.58E-10	4.72E-06	0.744	0.895
CLEC3A	4	-0.5350928	4.13E-08	0.00075302	0.167	0.409
FOS	4	-0.5435317	6.71E-08	0.00122453	0.372	0.595
COL11A1	4	-0.5624213	6.25E-09	0.00011405	0.821	0.945
CFD	4	-0.5679109	5.94E-08	0.00108339	0.083	0.314
IGFBP6	4	-0.5745789	2.32E-08	0.00042346	0.481	0.673
COL2A1	4	-0.5908466	1.78E-10	3.24E-06	0.647	0.895
IGF2	4	-0.5918955	8.95E-08	0.00163357	0.128	0.359
PLAC9	4	-0.5938373	6.40E-15	1.17E-10	0.744	0.95
IFI6	4	-0.6058849	5.41E-07	0.00987955	0.333	0.545
COL9A2	4	-0.6260807	3.34E-16	6.09E-12	0.314	0.691
TSC22D1	4	-0.6672191	3.90E-09	7.11E-05	0.455	0.673
C2orf40	4	-0.6728229	1.23E-15	2.24E-11	0.974	1
OGN	4	-0.6990433	2.41E-17	4.40E-13	0.917	1
CHI3L1	4	-0.7141984	1.18E-12	2.15E-08	0.872	0.977
CHI3L2	4	-0.7165805	3.65E-19	6.66E-15	0.577	0.914
FGFBP2	4	-0.7345936	2.58E-13	4.71E-09	0.814	0.977
S100B	4	-0.7348	7.40E-12	1.35E-07	0.635	0.818
NOVA1	4	-0.7826099	4.42E-17	8.07E-13	0.667	0.918
CP	4	-0.7848672	7.82E-16	1.43E-11	0.487	0.795
CCDC80	4	-0.8014993	3.03E-23	5.53E-19	0.878	0.986
CTSD	4	-0.8184881	1.97E-16	3.59E-12	0.897	0.995
COL11A2	4	-0.8227311	5.74E-23	1.05E-18	0.34	0.777
SCRG1	4	-0.8291632	3.04E-19	5.55E-15	0.987	1
COL9A3	4	-0.8818169	7.19E-19	1.31E-14	0.5	0.864
C2orf82	4	-0.9023762	8.51E-09	0.00015531	0.885	0.955

LECT1	4	-0.9889699	5.30E-27	9.67E-23	0.641	0.968
EPYC	4	-1.0028102	2.69E-23	4.91E-19	0.224	0.718
ACAN	4	-1.0353047	4.47E-31	8.15E-27	0.885	0.991
PLA2G2A	4	-1.0970194	4.80E-16	8.76E-12	0.462	0.841
SLPI	4	-1.7701439	6.37E-29	1.16E-24	0.821	0.991
**gene**	**cluster**	**avg_logFC**	**p_val**	**p_val_adj**	**pct.1**	**pct.2**
SPP1	5	1.331687	1.29E-12	2.35E-08	0.907	0.743
HMGA1	5	0.97106643	2.04E-30	3.72E-26	0.79	0.289
MT2A	5	0.87360808	2.62E-14	4.77E-10	1	1
CD55	5	0.80606444	5.54E-25	1.01E-20	0.917	0.587
TM4SF1	5	0.77987905	1.41E-17	2.57E-13	0.746	0.39
TIMP3	5	0.75302584	2.02E-13	3.68E-09	0.82	0.532
UPP1	5	0.64810147	3.40E-18	6.20E-14	0.834	0.472
CTSL	5	0.63919024	5.57E-15	1.02E-10	0.868	0.628
SMOX	5	0.55229184	1.94E-11	3.54E-07	0.571	0.307
IGFBP3	5	0.52324508	1.15E-08	0.00020965	0.61	0.344
RGCC	5	0.52224366	1.77E-10	3.22E-06	0.917	0.729
C11orf96	5	0.51227646	4.13E-09	7.53E-05	0.551	0.294
EIF1	5	0.50948054	1.89E-30	3.45E-26	1	1
SOD2	5	0.50328541	5.30E-11	9.68E-07	1	0.991
SNHG12	5	0.48398638	3.66E-14	6.68E-10	0.737	0.427
BZW1	5	0.45889954	7.48E-12	1.36E-07	0.878	0.628
ZFAS1	5	0.4473573	1.77E-16	3.23E-12	1	0.991
FAP	5	0.44061782	5.36E-14	9.78E-10	0.595	0.257
TMSB10	5	0.43712357	2.98E-08	0.0005432	1	0.968
GYPC	5	0.41120821	9.29E-12	1.70E-07	0.639	0.344
FNIP2	5	0.39248603	9.40E-09	0.00017152	0.483	0.243
EPB41L4A-AS1	5	0.39163486	4.81E-13	8.77E-09	0.966	0.766
GSTO1	5	0.38934895	2.12E-09	3.87E-05	0.829	0.628
PDLIM4	5	0.38149604	3.76E-09	6.86E-05	0.907	0.761
TGM2	5	0.37922274	7.59E-10	1.39E-05	0.395	0.147
TAGLN2	5	0.37778596	4.50E-08	0.00082126	0.761	0.569
S100A10	5	0.37222736	9.58E-09	0.00017487	1	0.986
PRDX6	5	0.35859539	3.91E-11	7.13E-07	0.907	0.752
ADSS	5	0.3503889	2.13E-08	0.00038886	0.717	0.491
TREM1	5	0.34771261	7.82E-07	0.01426417	0.546	0.33
VIMP	5	0.34424435	7.12E-10	1.30E-05	0.893	0.72
PSMA7	5	0.34106995	2.45E-09	4.46E-05	0.917	0.729
PMP22	5	0.30893922	3.29E-07	0.00600872	0.932	0.826

SH3GLB1	5	0.30152544	2.46E-08	0.00044924	0.795	0.601
RHOD	5	0.29933478	8.18E-08	0.00149277	0.566	0.321
SYF2	5	0.29390875	1.75E-07	0.00319398	0.912	0.766
AC090498.1	5	0.29369019	7.54E-17	1.38E-12	1	1
SH3BGRL3	5	0.28902425	7.15E-09	0.0001304	1	0.982
GPX4	5	0.28613883	4.60E-08	0.00083866	0.99	0.982
LRRFIP1	5	0.27761106	9.85E-07	0.01797795	0.42	0.216
HDAC2	5	0.27338554	6.47E-07	0.01180505	0.541	0.33
HINT1	5	0.26712089	9.59E-09	0.00017494	0.961	0.959
MIF	5	0.26375438	6.72E-13	1.23E-08	1	1
TATDN1	5	0.25938185	2.42E-06	0.04411865	0.62	0.44
RAC1	5	0.25838609	3.07E-07	0.00559397	0.971	0.876
NBEAL1	5	0.253388	1.23E-08	0.00022383	1	0.977
SGCB	5	-0.2559976	1.69E-06	0.03086427	0.268	0.482
TCEA3	5	-0.262302	1.06E-09	1.93E-05	0.146	0.408
TSPAN4	5	-0.2645159	4.79E-07	0.00873185	0.288	0.518
PSAP	5	-0.2652475	1.71E-07	0.00311227	0.844	0.94
FKBP10	5	-0.2673047	2.40E-06	0.04382472	0.293	0.486
FTL	5	-0.2709647	8.66E-15	1.58E-10	1	1
FCGRT	5	-0.2724851	2.74E-06	0.04992408	0.454	0.642
ENAH	5	-0.2743463	1.31E-07	0.00238647	0.088	0.28
DUT	5	-0.2757718	3.85E-07	0.00702395	0.459	0.683
NDUFA1	5	-0.2773306	1.67E-07	0.00305238	0.771	0.872
CALD1	5	-0.2790924	5.36E-08	0.00097757	0.439	0.679
SLC26A2	5	-0.2794415	1.14E-07	0.00208642	0.073	0.266
WFDC2	5	-0.2829659	6.30E-11	1.15E-06	0.029	0.257
FGFRL1	5	-0.2838761	1.61E-08	0.00029384	0.141	0.381
C4orf3	5	-0.2850996	1.28E-09	2.34E-05	0.985	1
RNASET2	5	-0.2860951	7.12E-08	0.00129828	0.156	0.376
H3F3A	5	-0.287205	2.70E-07	0.00491828	0.893	0.954
TCF4	5	-0.2881164	2.80E-10	5.11E-06	0.098	0.353
P4HA1	5	-0.2886026	1.59E-06	0.02903372	0.537	0.702
TMED2	5	-0.2892241	3.16E-07	0.00576901	0.712	0.807
GAS6	5	-0.2917943	5.11E-08	0.00093282	0.127	0.349
PABPC1	5	-0.2921166	3.55E-07	0.00647808	0.722	0.862
CCDC151	5	-0.2932525	1.91E-06	0.03479213	0.346	0.555
RP11-983P16.4	5	-0.2944052	3.68E-09	6.71E-05	0.195	0.463
SUMO1	5	-0.2981705	1.34E-08	0.00024401	0.59	0.752
EEF2	5	-0.2990847	1.21E-09	2.21E-05	0.985	1

MRPL33	5	-0.3035251	1.25E-06	0.02289863	0.595	0.78
TPI1	5	-0.3036688	8.36E-08	0.00152491	0.98	0.995
CD59	5	-0.3047873	2.56E-07	0.00467896	0.917	0.977
TCTN1	5	-0.3062397	2.66E-10	4.86E-06	0.088	0.335
PAPSS2	5	-0.3062397	1.20E-06	0.02190333	0.473	0.679
TRPS1	5	-0.3079864	1.49E-07	0.00272792	0.205	0.44
RCN2	5	-0.3091461	1.28E-06	0.02336653	0.459	0.642
BNIP3	5	-0.3092428	2.27E-07	0.0041358	1	0.995
STK26	5	-0.309513	1.30E-06	0.02366652	0.302	0.509
MIA	5	-0.3114683	1.36E-08	0.00024815	0.961	0.991
ATPIF1	5	-0.3121719	3.24E-09	5.91E-05	0.444	0.688
CRIP1	5	-0.315591	5.08E-08	0.00092607	0.2	0.436
PNISR	5	-0.3159572	5.36E-07	0.00977823	0.502	0.683
SNRPN	5	-0.3165415	3.79E-08	0.00069225	0.644	0.826
CHID1	5	-0.316641	2.79E-08	0.00050891	0.346	0.587
COL5A2	5	-0.3198825	1.92E-07	0.00350284	0.151	0.367
PEBP1	5	-0.3201602	2.03E-07	0.00371219	0.6	0.748
SRP9	5	-0.323523	3.36E-09	6.13E-05	0.62	0.803
LRPAP1	5	-0.3275862	1.91E-06	0.03493722	0.41	0.583
DNM3OS	5	-0.3284276	1.09E-10	1.99E-06	0.044	0.275
LAGE3	5	-0.3287126	8.86E-08	0.00161732	0.351	0.583
PCOLCE2	5	-0.3291503	1.90E-11	3.47E-07	0.932	0.982
SRSF5	5	-0.3295498	9.75E-08	0.00177977	0.449	0.638
TSPAN3	5	-0.3310766	5.26E-07	0.00959048	0.366	0.569
IFITM10	5	-0.3321104	2.17E-11	3.96E-07	0.068	0.33
HNRNPA1	5	-0.3353815	7.11E-11	1.30E-06	0.976	0.986
HIF1A	5	-0.3354451	2.87E-07	0.00523539	0.81	0.899
EEF1A1	5	-0.3357451	3.45E-20	6.30E-16	1	1
CD320	5	-0.3360927	4.34E-09	7.91E-05	0.288	0.573
FAM162A	5	-0.33692	1.03E-08	0.00018772	0.941	0.982
UGP2	5	-0.3387581	6.90E-07	0.01259797	0.561	0.693
ISLR	5	-0.3396037	1.10E-12	2.01E-08	0.083	0.376
INSIG2	5	-0.3417404	1.70E-06	0.03098038	0.541	0.711
IGFBP7	5	-0.3424463	1.98E-07	0.00361804	0.78	0.917
SAT1	5	-0.3445626	5.65E-08	0.00103161	0.941	0.991
HMGN2	5	-0.3477183	2.60E-10	4.75E-06	0.795	0.945
SOX9	5	-0.3523134	8.18E-07	0.01492016	0.576	0.739
AEBP1	5	-0.3534272	8.34E-07	0.0152251	0.727	0.812
POLR2L	5	-0.3545162	7.53E-08	0.00137371	0.722	0.872

EID1	5	-0.3555075	1.71E-11	3.13E-07	0.834	0.959
N4BP2L2	5	-0.355644	3.59E-08	0.00065503	0.668	0.812
DDR2	5	-0.3596147	5.27E-08	0.00096245	0.244	0.482
PNRC1	5	-0.360347	4.78E-08	0.00087247	0.927	0.977
ITM2B	5	-0.3619062	1.03E-08	0.00018856	0.971	0.991
SEPP1	5	-0.3639807	8.88E-09	0.00016201	0.205	0.468
SPON2	5	-0.3644187	6.42E-11	1.17E-06	0.078	0.33
IFITM3	5	-0.3649375	1.48E-09	2.70E-05	0.829	0.95
DYNLL1	5	-0.3683327	4.99E-10	9.10E-06	0.78	0.899
MT-ND4	5	-0.3688776	1.10E-07	0.00201516	0.917	0.972
PDIA6	5	-0.3690054	2.91E-09	5.32E-05	0.42	0.651
CTSF	5	-0.371379	3.82E-12	6.97E-08	0.127	0.427
BHLHE41	5	-0.3723105	7.56E-11	1.38E-06	0.102	0.367
IFITM1	5	-0.3742064	5.16E-09	9.41E-05	0.283	0.555
SDC2	5	-0.3746961	5.36E-09	9.78E-05	0.683	0.862
S100A4	5	-0.3768679	1.30E-06	0.02363326	0.585	0.743
GRN	5	-0.3791207	1.32E-12	2.41E-08	0.098	0.394
NME4	5	-0.3817695	5.38E-13	9.82E-09	0.176	0.5
CDO1	5	-0.3832007	1.34E-12	2.45E-08	0.078	0.362
TTC3	5	-0.387647	1.01E-10	1.84E-06	0.42	0.697
ANGPTL2	5	-0.3881512	2.93E-09	5.34E-05	0.341	0.615
MALAT1	5	-0.3881966	5.27E-14	9.61E-10	1	1
IFI6	5	-0.3888926	5.20E-07	0.00949468	0.215	0.436
SERF2	5	-0.3914069	3.70E-16	6.75E-12	1	1
B2M	5	-0.3957998	6.02E-13	1.10E-08	0.976	1
PDIA3	5	-0.3965424	2.88E-08	0.00052625	0.376	0.592
P4HB	5	-0.3980472	1.88E-10	3.43E-06	0.605	0.789
CNPY2	5	-0.3994098	4.75E-11	8.66E-07	0.571	0.775
SRGN	5	-0.4012809	3.83E-10	6.98E-06	0.19	0.463
IER2	5	-0.4021507	1.61E-07	0.00293444	0.166	0.381
NDUFA4L2	5	-0.404242	9.46E-27	1.73E-22	1	1
SMOC2	5	-0.4046046	3.87E-10	7.07E-06	0.068	0.303
TPD52	5	-0.4077772	2.94E-11	5.37E-07	0.698	0.853
CA9	5	-0.4096577	6.92E-08	0.00126224	0.346	0.578
PKM	5	-0.41467	8.77E-11	1.60E-06	0.815	0.927
SCRG1	5	-0.414917	2.33E-08	0.00042498	0.985	1
ID2	5	-0.4185487	5.72E-09	0.00010432	0.293	0.546
MT-ND3	5	-0.4205846	8.36E-10	1.52E-05	0.376	0.647
SDF4	5	-0.4226723	1.26E-11	2.30E-07	0.454	0.697

ID4	5	-0.4265304	2.81E-08	0.00051329	0.293	0.541
LY6E	5	-0.4283867	3.37E-14	6.14E-10	0.307	0.638
CHRDL2	5	-0.4305415	5.73E-09	0.00010453	0.151	0.394
C7orf73	5	-0.4321727	5.20E-08	0.00094842	0.6	0.748
NAA38	5	-0.4376053	1.60E-13	2.92E-09	0.459	0.757
ATRAID	5	-0.4397199	2.25E-15	4.11E-11	0.737	0.945
PAM	5	-0.4400669	2.31E-10	4.22E-06	0.546	0.72
PLD3	5	-0.4480975	6.11E-12	1.11E-07	0.395	0.67
DCN	5	-0.4504172	7.77E-18	1.42E-13	1	1
C1S	5	-0.4509652	1.32E-12	2.40E-08	0.454	0.748
MT-CO1	5	-0.4626992	1.36E-08	0.00024862	0.902	0.963
PPP1R3C	5	-0.4665823	3.15E-10	5.74E-06	0.263	0.528
PRDX4	5	-0.4670861	8.16E-12	1.49E-07	0.595	0.803
FAM46A	5	-0.4719248	4.23E-13	7.71E-09	0.376	0.711
LCN2	5	-0.4735452	6.04E-08	0.00110253	0.332	0.564
MT-ND2	5	-0.4752222	3.93E-11	7.18E-07	0.307	0.619
MT-CYB	5	-0.4857082	5.26E-08	0.00096	0.483	0.656
EGR1	5	-0.4858743	4.00E-09	7.30E-05	0.156	0.408
CHI3L1	5	-0.4983437	6.49E-09	0.0001184	0.98	1
HTRA1	5	-0.5116444	1.75E-14	3.20E-10	0.551	0.844
MT-CO3	5	-0.5165072	1.68E-10	3.06E-06	0.8	0.922
CFD	5	-0.517373	8.15E-09	0.00014871	0.112	0.349
GOLIM4	5	-0.5220635	1.08E-06	0.01972372	0.244	0.436
C10orf10	5	-0.5297859	9.81E-09	0.00017891	0.39	0.633
COL11A2	5	-0.5317961	1.90E-14	3.46E-10	0.415	0.734
COL9A2	5	-0.5363519	5.96E-17	1.09E-12	0.195	0.578
CYTL1	5	-0.5398545	7.93E-10	1.45E-05	0.078	0.312
MT-ND1	5	-0.5475174	3.63E-13	6.62E-09	0.185	0.518
CST3	5	-0.5520099	2.57E-22	4.70E-18	0.985	1
FMOD	5	-0.5596282	4.20E-22	7.66E-18	0.873	0.977
LSP1	5	-0.5629852	6.90E-18	1.26E-13	0.093	0.459
VKORC1	5	-0.5664216	3.12E-20	5.70E-16	0.912	0.986
MT-ATP6	5	-0.5682925	2.68E-10	4.89E-06	0.644	0.807
DUSP1	5	-0.5721713	3.44E-11	6.28E-07	0.283	0.546
SNAI2	5	-0.5738425	2.69E-11	4.91E-07	0.18	0.468
BTG1	5	-0.5787749	2.23E-15	4.06E-11	0.985	0.995
CD99	5	-0.5796519	1.29E-20	2.36E-16	0.81	0.972
NGFRAP1	5	-0.5875452	3.16E-18	5.77E-14	0.659	0.853
FBXO2	5	-0.5997371	2.32E-22	4.23E-18	0.478	0.849

MDFI	5	-0.6044125	4.96E-15	9.05E-11	0.239	0.569
RHOBTB3	5	-0.6096832	7.00E-19	1.28E-14	0.278	0.683
OGN	5	-0.6401547	6.05E-13	1.10E-08	0.722	0.908
NOVA1	5	-0.6532986	4.91E-14	8.96E-10	0.878	0.945
FOS	5	-0.6608187	3.96E-15	7.23E-11	0.317	0.661
ITM2C	5	-0.6651848	1.96E-19	3.57E-15	0.229	0.619
BAMBI	5	-0.6785672	2.16E-19	3.93E-15	0.312	0.725
PRELP	5	-0.7021288	6.05E-22	1.10E-17	0.917	1
FXYD6	5	-0.7174621	6.60E-26	1.20E-21	0.576	0.885
MT1H	5	-0.7198619	1.54E-07	0.0028116	0.654	0.789
CHI3L2	5	-0.7262611	7.21E-19	1.32E-14	0.756	0.968
RBP4	5	-0.7301543	1.01E-14	1.84E-10	0.585	0.853
CP	5	-0.7505233	7.82E-10	1.43E-05	0.556	0.725
MT-CO2	5	-0.7600291	3.04E-13	5.55E-09	0.859	0.936
MT1G	5	-0.7615072	6.28E-15	1.15E-10	0.932	0.977
LECT1	5	-0.7646652	3.17E-22	5.78E-18	0.722	0.954
JUNB	5	-0.7905392	4.57E-14	8.35E-10	0.405	0.683
TSC22D1	5	-0.7973383	1.62E-17	2.95E-13	0.532	0.812
WWP2	5	-0.799858	8.61E-25	1.57E-20	0.527	0.858
COL9A3	5	-0.8076997	7.58E-20	1.38E-15	0.488	0.826
C2orf40	5	-0.8214116	9.67E-25	1.77E-20	0.98	1
MT1F	5	-0.8464629	4.19E-20	7.64E-16	0.824	0.959
ID1	5	-0.8602701	6.12E-13	1.12E-08	0.22	0.514
CCDC80	5	-0.8866482	1.37E-28	2.51E-24	0.946	0.995
ACAN	5	-0.9202126	4.16E-36	7.58E-32	0.917	0.995
ID3	5	-0.9260421	2.33E-14	4.25E-10	0.146	0.468
S100B	5	-0.9300581	9.53E-29	1.74E-24	0.663	0.917
COL2A1	5	-0.9330296	2.93E-22	5.34E-18	0.317	0.725
COL11A1	5	-0.9412792	1.19E-26	2.17E-22	0.556	0.881
EPYC	5	-0.944896	3.19E-28	5.81E-24	0.078	0.573
PLA2G2A	5	-0.9659868	5.58E-18	1.02E-13	0.766	0.927
PLAC9	5	-1.0423924	1.32E-28	2.40E-24	0.488	0.867
CLEC3A	5	-1.1240495	1.90E-26	3.47E-22	0.215	0.693
CTSD	5	-1.2496984	1.70E-32	3.10E-28	0.776	0.986
SLPI	5	-1.3748608	7.35E-21	1.34E-16	0.976	0.995
TMSB4X	6	0.87683199	1.12E-15	2.04E-11	0.903	0.712
**gene**	**cluster**	**avg_logFC**	**p_val**	**p_val_adj**	**pct.1**	**pct.2**
MT2A	6	0.76158514	2.78E-31	5.07E-27	1	1
CD55	6	0.66638106	1.05E-23	1.91E-19	0.925	0.712

TM4SF1	6	0.64118796	3.65E-19	6.66E-15	0.915	0.737
LGALS1	6	0.54421922	6.15E-16	1.12E-11	0.994	0.979
TIMP3	6	0.51060876	7.10E-07	0.01294673	0.682	0.523
TMSB10	6	0.50062258	4.88E-19	8.90E-15	1	0.996
HMGA1	6	0.43291793	9.51E-11	1.74E-06	0.89	0.7
GSTO1	6	0.41404436	1.66E-13	3.03E-09	0.868	0.708
AKAP12	6	0.40620344	2.87E-13	5.24E-09	0.406	0.136
NRP2	6	0.40325056	1.46E-06	0.02656543	0.588	0.444
C11orf96	6	0.35366264	8.16E-08	0.00148958	0.676	0.461
S100A6	6	0.33090679	2.20E-09	4.01E-05	1	1
FHL2	6	0.32429125	1.99E-08	0.00036228	0.553	0.354
EIF1	6	0.30178743	8.36E-15	1.53E-10	1	1
NBEAL1	6	0.27675418	1.71E-11	3.12E-07	0.997	0.979
LRRFIP1	6	0.26861671	8.54E-07	0.01557483	0.654	0.506
EGLN1	6	-0.2534168	1.22E-07	0.00223192	0.33	0.523
ARMCX3	6	-0.2541749	3.32E-07	0.00605385	0.384	0.564
NAA38	6	-0.2542295	1.93E-07	0.00351394	0.456	0.642
H3F3B	6	-0.2552777	1.24E-08	0.00022628	0.994	0.996
LAMP1	6	-0.2556564	1.04E-06	0.01903838	0.314	0.498
EEF2	6	-0.2556988	1.00E-06	0.01832497	0.969	0.979
SLC39A8	6	-0.2575873	2.07E-09	3.77E-05	0.286	0.519
CD320	6	-0.2579887	1.71E-07	0.00312134	0.362	0.543
IFI6	6	-0.2596441	1.30E-06	0.02363617	0.211	0.383
P4HB	6	-0.2638748	5.30E-08	0.00096767	0.645	0.852
FCGRT	6	-0.2650529	1.93E-08	0.00035249	0.412	0.617
EEF1A1	6	-0.2669596	7.10E-20	1.30E-15	1	1
HNRNPA1	6	-0.2684229	7.45E-10	1.36E-05	0.959	0.988
TPD52L1	6	-0.2764994	3.80E-08	0.00069349	0.204	0.403
CRIP1	6	-0.2770383	4.73E-07	0.00862677	0.286	0.481
CHID1	6	-0.2774288	5.31E-09	9.69E-05	0.374	0.593
PNRC1	6	-0.2803465	1.35E-07	0.0024716	0.909	0.963
SOX9	6	-0.2826141	5.52E-07	0.01007777	0.604	0.774
SAT1	6	-0.2830535	3.60E-07	0.00657105	0.912	0.979
ME1	6	-0.2856769	9.14E-07	0.01667304	0.239	0.412
DDR2	6	-0.286425	7.71E-10	1.41E-05	0.208	0.432
NOL3	6	-0.2900433	3.83E-08	0.00069802	0.358	0.56
SDC2	6	-0.2901219	6.20E-11	1.13E-06	0.635	0.852
TMEM45A	6	-0.2914628	8.56E-08	0.00156189	0.887	0.947
FOXA3	6	-0.2930231	3.44E-11	6.29E-07	0.116	0.342

PDIA6	6	-0.2960919	1.77E-09	3.22E-05	0.472	0.663
SERPING1	6	-0.296229	3.53E-07	0.00644876	0.418	0.58
NT5C3A	6	-0.2995111	6.78E-07	0.01237722	0.343	0.523
MEF2A	6	-0.3007386	4.13E-08	0.0007539	0.346	0.543
LY6E	6	-0.300806	1.45E-10	2.65E-06	0.308	0.56
TPD52	6	-0.3016338	6.75E-10	1.23E-05	0.575	0.786
HSD17B14	6	-0.3071625	2.26E-08	0.00041167	0.425	0.601
FXYD6	6	-0.3074034	2.37E-07	0.00432837	0.651	0.778
IGF2	6	-0.3076688	3.39E-08	0.00061883	0.088	0.259
PLD3	6	-0.3134417	3.40E-07	0.0062094	0.403	0.588
PDIA3	6	-0.3136142	2.11E-09	3.84E-05	0.409	0.63
CYTL1	6	-0.3144141	4.89E-08	0.00089268	0.179	0.383
PKM	6	-0.3186941	2.30E-08	0.00041888	0.871	0.955
PPP1R3C	6	-0.3195568	8.26E-09	0.00015078	0.327	0.551
MMP3	6	-0.3206106	2.13E-08	0.00038933	0.774	0.881
CTSF	6	-0.3233371	5.76E-14	1.05E-09	0.16	0.436
FOS	6	-0.3251518	3.01E-07	0.00549698	0.336	0.519
MDFI	6	-0.3255022	9.44E-07	0.01723245	0.286	0.453
HMGN2	6	-0.3277288	2.84E-11	5.18E-07	0.723	0.872
SPON2	6	-0.3306341	6.90E-14	1.26E-09	0.063	0.296
IFRD1	6	-0.3307379	7.93E-07	0.01447146	0.572	0.695
LUM	6	-0.3312785	4.15E-09	7.58E-05	0.997	1
P4HA1	6	-0.3341596	1.57E-10	2.86E-06	0.572	0.745
LSP1	6	-0.3451513	2.24E-09	4.10E-05	0.214	0.428
EGLN3	6	-0.3454484	7.59E-08	0.00138502	0.343	0.547
CNPY2	6	-0.3493123	2.08E-12	3.80E-08	0.456	0.7
B2M	6	-0.3502479	6.66E-15	1.21E-10	0.972	0.988
SDF4	6	-0.3529161	6.79E-12	1.24E-07	0.465	0.675
PRDX4	6	-0.3542342	2.24E-10	4.09E-06	0.597	0.794
HTRA1	6	-0.354636	4.02E-13	7.33E-09	0.588	0.802
PAM	6	-0.3578212	1.07E-14	1.95E-10	0.481	0.741
SLC16A3	6	-0.3578775	1.83E-07	0.00333669	0.579	0.737
ADM	6	-0.3581776	2.51E-08	0.00045784	0.673	0.848
MALAT1	6	-0.3603078	1.75E-15	3.19E-11	1	1
PSAP	6	-0.3622693	1.01E-11	1.84E-07	0.777	0.918
FAM162A	6	-0.3661304	8.92E-14	1.63E-09	0.934	0.984
HSP90B1	6	-0.3664738	1.23E-11	2.24E-07	0.594	0.827
VKORC1	6	-0.3668758	2.93E-13	5.35E-09	0.947	0.963
INSIG2	6	-0.3687572	9.46E-09	0.00017264	0.613	0.774

PGK1	6	-0.3710174	2.58E-11	4.71E-07	0.918	0.984
ANGPTL2	6	-0.3713013	4.43E-11	8.08E-07	0.252	0.498
CP	6	-0.3721454	5.24E-08	0.00095549	0.522	0.695
NDUFA4L2	6	-0.3806962	1.37E-30	2.50E-26	1	1
BNIP3	6	-0.382419	1.73E-15	3.16E-11	0.997	1
MIA	6	-0.3858578	4.38E-15	8.00E-11	0.95	0.988
TMEM59	6	-0.3873455	6.04E-15	1.10E-10	0.956	0.992
GSN	6	-0.3942862	7.93E-12	1.45E-07	0.283	0.535
VCAN	6	-0.3950307	3.23E-13	5.90E-09	0.176	0.453
C1S	6	-0.3963094	6.96E-10	1.27E-05	0.415	0.634
AK2	6	-0.4003259	1.64E-09	3.00E-05	0.761	0.852
IGFBP6	6	-0.4043313	1.18E-09	2.15E-05	0.484	0.704
CAPS	6	-0.4160434	7.06E-11	1.29E-06	0.447	0.683
CD99	6	-0.4321093	3.87E-15	7.06E-11	0.789	0.93
SERPINA1	6	-0.4400844	4.53E-14	8.27E-10	0.865	0.975
ITM2C	6	-0.4406162	5.38E-20	9.82E-16	0.286	0.663
SNRPN	6	-0.4422219	1.13E-19	2.06E-15	0.503	0.794
DDIT4L	6	-0.4425545	1.21E-10	2.20E-06	0.824	0.914
ATRAID	6	-0.4528805	4.61E-20	8.41E-16	0.66	0.893
IGFBP7	6	-0.4538654	2.21E-14	4.03E-10	0.701	0.914
FBXO2	6	-0.4594482	6.09E-16	1.11E-11	0.418	0.691
LRPAP1	6	-0.4620903	2.60E-17	4.74E-13	0.314	0.626
CA9	6	-0.464252	2.53E-08	0.00046105	0.525	0.691
COL11A2	6	-0.4719308	2.38E-13	4.33E-09	0.299	0.551
TSC22D1	6	-0.4778264	2.79E-07	0.0050965	0.66	0.765
WWP2	6	-0.4782978	5.31E-15	9.70E-11	0.318	0.626
COL11A1	6	-0.4826706	1.04E-13	1.90E-09	0.437	0.757
COL9A2	6	-0.4872909	5.03E-11	9.18E-07	0.189	0.432
FAM46A	6	-0.4905322	1.41E-14	2.57E-10	0.374	0.646
NGFRAP1	6	-0.4910029	5.32E-17	9.70E-13	0.648	0.84
ITM2B	6	-0.498778	6.75E-20	1.23E-15	0.94	0.992
CLU	6	-0.5113047	4.06E-15	7.40E-11	1	1
CCDC85B	6	-0.5217112	2.98E-13	5.43E-09	0.918	0.975
LCN2	6	-0.5308935	7.17E-08	0.00130782	0.283	0.477
ARRDC3	6	-0.5346439	1.78E-12	3.24E-08	0.531	0.741
PCOLCE2	6	-0.5401793	2.96E-22	5.40E-18	0.792	0.951
CLEC3A	6	-0.542158	9.16E-20	1.67E-15	0.075	0.383
CFD	6	-0.5483732	3.64E-13	6.64E-09	0.091	0.329
SCRG1	6	-0.5571383	1.37E-14	2.50E-10	0.953	0.992

IGFBP5	6	-0.5930754	7.87E-14	1.44E-09	0.182	0.469
GAPDH	6	-0.60482	1.65E-22	3.00E-18	1	1
EGR1	6	-0.6054622	6.26E-14	1.14E-09	0.226	0.486
MT1F	6	-0.6076368	4.51E-08	0.00082254	0.849	0.893
HIF1A	6	-0.6193953	5.49E-22	1.00E-17	0.692	0.905
FMOD	6	-0.6199497	1.19E-23	2.16E-19	0.726	0.926
OGN	6	-0.655709	7.71E-15	1.41E-10	0.777	0.901
CST3	6	-0.6618683	4.77E-33	8.71E-29	0.969	1
COL9A3	6	-0.6860025	3.09E-13	5.64E-09	0.403	0.634
DCN	6	-0.7154734	1.01E-38	1.85E-34	1	1
PLAC9	6	-0.727388	1.07E-15	1.95E-11	0.456	0.733
S100B	6	-0.7355213	2.51E-20	4.58E-16	0.506	0.741
CHI3L1	6	-0.7480705	4.66E-12	8.50E-08	0.89	0.984
EPYC	6	-0.7492392	4.84E-26	8.82E-22	0.079	0.453
CHI3L2	6	-0.7852064	1.14E-24	2.08E-20	0.597	0.901
C2orf82	6	-0.7998335	7.37E-14	1.35E-09	0.921	0.963
FGFBP2	6	-0.801238	4.63E-26	8.45E-22	0.855	0.984
C2orf40	6	-0.8017192	2.47E-20	4.51E-16	0.965	1
COL2A1	6	-0.8186313	5.08E-17	9.27E-13	0.248	0.568
ACAN	6	-0.8674719	4.80E-37	8.75E-33	0.77	0.963
RBP4	6	-0.9764932	9.05E-24	1.65E-19	0.497	0.798
LECT1	6	-1.0206904	4.22E-31	7.70E-27	0.519	0.926
PRELP	6	-1.066544	2.49E-46	4.54E-42	0.777	0.988
CCDC80	6	-1.1139139	1.09E-43	1.99E-39	0.698	0.971
PLA2G2A	6	-1.2838713	6.41E-26	1.17E-21	0.311	0.712
SLPI	6	-1.3055529	4.70E-31	8.58E-27	0.783	0.984
CTSD	6	-1.4583018	3.06E-36	5.59E-32	0.783	0.979
**gene**	**cluster**	**avg_logFC**	**p_val**	**p_val_adj**	**pct.1**	**pct.2**
MT2A	7	0.93481962	9.32E-20	1.70E-15	1	0.991
TMSB4X	7	0.80481004	3.54E-14	6.46E-10	0.995	1
IGFBP3	7	0.77611107	4.37E-10	7.98E-06	0.821	0.573
LGALS1	7	0.73070547	3.41E-21	6.23E-17	1	1
KIAA0101	7	0.70016475	4.62E-09	8.43E-05	0.415	0.111
PMEPA1	7	0.69853449	1.59E-16	2.90E-12	0.744	0.308
C12orf75	7	0.671355	2.45E-11	4.48E-07	0.773	0.513
PGF	7	0.65386976	1.29E-06	0.02355048	0.502	0.239
PPP1R14B	7	0.61851733	4.49E-09	8.18E-05	0.778	0.59
LINC00152	7	0.59560364	9.60E-12	1.75E-07	0.778	0.453
CCND1	7	0.55860415	4.37E-08	0.00079672	0.671	0.427

SCX	7	0.5568464	1.10E-08	0.00020004	0.415	0.12
MIR4435-2HG	7	0.5433349	5.09E-12	9.28E-08	0.758	0.402
TM4SF1	7	0.53310522	2.52E-07	0.00459159	0.754	0.496
TPM1	7	0.52277641	2.87E-09	5.24E-05	0.865	0.718
NRP2	7	0.51707831	3.34E-08	0.00060975	0.676	0.41
PFN1	7	0.45538488	5.65E-07	0.01030182	0.995	0.966
AXL	7	0.44327089	2.35E-06	0.04287582	0.787	0.581
UBE2S	7	0.42109531	1.21E-06	0.02206715	0.411	0.171
GNG11	7	0.41261812	4.25E-07	0.007747	0.71	0.479
SPOCK1	7	0.39619966	1.41E-09	2.57E-05	0.502	0.171
ACTB	7	0.39458901	1.99E-07	0.00363891	0.99	0.957
MT-CO1	7	0.37736073	1.00E-06	0.01833154	1	0.949
STMN1	7	0.3713883	1.65E-07	0.00300428	0.44	0.154
JAG1	7	0.34303951	1.06E-07	0.00193919	0.478	0.179
HINT1	7	0.33197905	9.30E-07	0.01696866	0.986	0.94
MT-ND4	7	0.33158942	8.69E-07	0.01585103	0.99	0.949
FAM89B	7	0.32327302	2.76E-07	0.00503635	0.473	0.188
PLP2	7	0.31716254	1.06E-06	0.01940111	0.87	0.701
ARL4C	7	0.31414053	2.48E-06	0.0452978	0.338	0.103
BIRC5	7	0.29662508	7.48E-08	0.00136578	0.251	0.017
TMSB10	7	0.29594142	6.02E-07	0.01097746	1	1
TK1	7	0.27786845	1.96E-06	0.03579048	0.29	0.068
SMAD9	7	-0.2548167	8.96E-07	0.01634848	0.097	0.308
EBF1	7	-0.259267	7.56E-07	0.0138019	0.145	0.385
COMMD6	7	-0.2635776	1.92E-08	0.00034974	0.971	1
CTSA	7	-0.2664999	1.45E-07	0.00265179	0.222	0.504
KAZALD1	7	-0.2735973	2.35E-07	0.00428877	0.193	0.453
FAM134B	7	-0.2965429	1.00E-08	0.00018302	0.145	0.419
DYNLT3	7	-0.2999991	3.84E-07	0.00700179	0.575	0.812
ATP6V1G1	7	-0.3023737	1.64E-06	0.02992762	0.937	0.974
NENF	7	-0.3068552	8.90E-08	0.00162331	0.899	0.974
MALAT1	7	-0.3087621	5.90E-08	0.00107683	1	1
IGF2	7	-0.3192304	1.76E-07	0.0032096	0.039	0.222
SSR4	7	-0.3253121	1.64E-07	0.00299048	0.937	0.991
FXYD1	7	-0.330872	2.31E-08	0.00042188	0.155	0.419
FTL	7	-0.3460857	3.06E-12	5.59E-08	1	1
NDUFA4L2	7	-0.3461759	1.63E-10	2.97E-06	0.986	1
FCGRT	7	-0.3474013	6.72E-07	0.01226299	0.377	0.632
SPON2	7	-0.3483407	3.31E-07	0.00604092	0.155	0.41

SMIM5	7	-0.3517586	6.38E-10	1.16E-05	0.092	0.368
N4BP2L2	7	-0.3528808	2.56E-07	0.0046761	0.643	0.829
CST3	7	-0.357904	9.27E-09	0.00016919	0.961	1
SELM	7	-0.3677984	3.25E-11	5.93E-07	0.957	1
C6orf48	7	-0.3719684	6.54E-08	0.00119328	0.758	0.915
SRGN	7	-0.3794896	2.57E-08	0.00046857	0.309	0.607
PCOLCE2	7	-0.3839975	1.16E-06	0.02112466	0.802	0.897
PPIC	7	-0.3862069	2.64E-07	0.00482613	0.778	0.897
PPP1R3C	7	-0.3882233	2.57E-06	0.04693993	0.353	0.59
SOD3	7	-0.3970173	5.34E-08	0.00097378	0.541	0.846
SERPINA1	7	-0.404681	1.23E-06	0.0225041	0.473	0.726
PAM	7	-0.4140239	2.07E-09	3.78E-05	0.473	0.735
SEPP1	7	-0.4246556	6.79E-13	1.24E-08	0.053	0.359
UGP2	7	-0.4350863	5.44E-09	9.93E-05	0.502	0.769
C1S	7	-0.4436142	7.91E-09	0.00014426	0.261	0.564
VKORC1	7	-0.4480874	4.44E-10	8.11E-06	0.995	1
TIMP1	7	-0.4508196	2.67E-09	4.88E-05	0.995	1
ATRAID	7	-0.4523262	2.81E-11	5.13E-07	0.787	0.94
CD99	7	-0.4524322	1.44E-14	2.62E-10	0.961	1
IFI6	7	-0.4589309	1.75E-07	0.00319973	0.357	0.632
ID4	7	-0.4608125	1.00E-06	0.01823814	0.512	0.718
MFGE8	7	-0.4740233	7.33E-08	0.00133723	0.517	0.752
CEBPD	7	-0.4752347	3.88E-10	7.08E-06	0.43	0.752
PPP3CA	7	-0.4781715	1.22E-06	0.02218957	0.749	0.897
CLEC3A	7	-0.4831329	2.28E-12	4.16E-08	0.068	0.376
LUM	7	-0.5026133	2.39E-15	4.36E-11	0.99	1
ITM2C	7	-0.5054056	2.54E-08	0.00046334	0.599	0.752
WWP2	7	-0.5078463	2.07E-09	3.78E-05	0.43	0.709
TMEM59	7	-0.5086695	1.79E-12	3.27E-08	0.816	0.957
NUPR1	7	-0.5281493	7.50E-09	0.00013692	0.749	0.915
FMOD	7	-0.5348799	1.18E-10	2.16E-06	0.734	0.923
BAMBI	7	-0.5511268	4.74E-08	0.00086576	0.29	0.556
CP	7	-0.5742972	1.96E-13	3.58E-09	0.145	0.513
CAPS	7	-0.5904602	1.45E-06	0.02645484	0.353	0.573
IGFBP6	7	-0.5913789	2.88E-07	0.00526032	0.647	0.769
HIF1A	7	-0.592561	1.85E-11	3.37E-07	0.763	0.897
DCN	7	-0.5956937	1.67E-16	3.05E-12	0.942	1
FBXO2	7	-0.598038	8.08E-13	1.47E-08	0.309	0.641
MGP	7	-0.6009583	5.11E-11	9.32E-07	0.923	0.991

PNRC1	7	-0.6109915	8.66E-14	1.58E-09	0.662	0.923
SOX9	7	-0.6133067	8.56E-12	1.56E-07	0.498	0.803
ID3	7	-0.6160072	1.13E-08	0.00020687	0.739	0.906
COL9A2	7	-0.6298215	3.39E-18	6.19E-14	0.203	0.658
SAT1	7	-0.6405208	2.22E-08	0.00040516	0.947	0.974
CHI3L2	7	-0.6441249	7.67E-10	1.40E-05	0.382	0.65
B2M	7	-0.6530138	1.64E-25	3.00E-21	0.99	1
TNFRSF11B	7	-0.6642508	9.66E-09	0.00017618	0.401	0.684
ITM2B	7	-0.6647567	2.63E-17	4.81E-13	0.899	0.983
FGFBP2	7	-0.7208834	4.90E-14	8.94E-10	0.43	0.803
HAPLN1	7	-0.7336965	1.64E-12	3.00E-08	0.522	0.795
EPYC	7	-0.8015867	3.79E-22	6.91E-18	0.058	0.521
COL11A1	7	-0.8091503	3.08E-13	5.62E-09	0.836	0.932
S100B	7	-0.8115179	1.31E-12	2.39E-08	0.386	0.718
OGN	7	-0.8517318	1.92E-13	3.50E-09	0.473	0.803
COL11A2	7	-0.8695731	2.13E-25	3.89E-21	0.126	0.667
MIA	7	-0.878656	4.31E-20	7.86E-16	0.739	0.94
CTSD	7	-0.8972075	7.81E-17	1.42E-12	0.758	0.983
PLAC9	7	-0.9160307	1.67E-19	3.05E-15	0.734	0.966
NOVA1	7	-0.9219427	5.71E-16	1.04E-11	0.377	0.718
S100A1	7	-0.9604368	6.39E-17	1.17E-12	0.787	0.949
SCRG1	7	-1.0193068	2.35E-19	4.29E-15	0.942	0.966
C2orf40	7	-1.1129423	5.99E-19	1.09E-14	0.618	0.932
COL9A3	7	-1.1266944	1.92E-24	3.51E-20	0.275	0.778
ACAN	7	-1.2100741	2.53E-25	4.62E-21	0.705	0.932
LECT1	7	-1.3291797	1.00E-27	1.82E-23	0.348	0.855
CHI3L1	7	-1.3578476	9.43E-21	1.72E-16	0.454	0.897
COL2A1	7	-1.4072325	7.16E-23	1.31E-18	0.425	0.897
SLPI	7	-1.4965332	9.01E-25	1.64E-20	0.444	0.889
PLA2G2A	7	-1.7524335	9.40E-21	1.72E-16	0.266	0.718
**gene**	**cluster**	**avg_logFC**	**p_val**	**p_val_adj**	**pct.1**	**pct.2**
MT2A	8	1.31271711	5.66E-16	1.03E-11	1	1
SERPINE2	8	0.79079465	5.76E-12	1.05E-07	1	1
LGALS1	8	0.73069656	6.59E-12	1.20E-07	1	0.994
ABI3BP	8	0.6223593	1.91E-06	0.03482323	0.707	0.448
RGCC	8	0.57330742	8.72E-09	0.00015918	0.878	0.61
FN1	8	0.56409956	4.45E-12	8.12E-08	1	1
COL6A3	8	0.51706927	2.16E-08	0.00039358	0.683	0.364
NRP2	8	0.51111253	2.24E-10	4.10E-06	0.537	0.156

TNC	8	0.50348165	1.57E-12	2.86E-08	0.476	0.084
TGM2	8	0.50267213	1.64E-10	2.99E-06	0.415	0.078
SLC20A1	8	0.49973687	2.45E-06	0.04471562	0.402	0.149
ARPC1B	8	0.49722511	2.38E-07	0.00434807	0.768	0.461
NT5E	8	0.4805291	3.41E-09	6.22E-05	0.524	0.175
TM4SF1	8	0.47755652	2.06E-06	0.03752351	0.549	0.24
SH3BGRL3	8	0.46849843	8.85E-09	0.00016146	1	0.987
BMP2	8	0.46051497	2.94E-07	0.00536361	0.451	0.156
FXYD5	8	0.4502293	2.75E-07	0.00502523	0.939	0.753
S100A16	8	0.43052006	2.48E-06	0.04518416	0.598	0.305
GYPC	8	0.42843399	8.26E-07	0.01506506	0.512	0.234
LTBP1	8	0.42632117	2.58E-06	0.04711921	0.512	0.24
LINC00152	8	0.41982883	4.64E-08	0.00084682	0.512	0.175
WWTR1	8	0.41790072	9.37E-08	0.00171057	0.671	0.344
SLC25A5	8	0.4138542	3.26E-09	5.94E-05	0.732	0.351
TMSB10	8	0.41357054	2.25E-06	0.04097836	1	1
PLP2	8	0.4102197	3.22E-08	0.00058721	0.866	0.506
CCDC109B	8	0.39890422	7.17E-10	1.31E-05	0.549	0.169
VDAC2	8	0.39166533	1.38E-07	0.00252385	0.72	0.403
IER3	8	0.38955733	2.46E-07	0.00449609	0.439	0.136
PPP1R14B	8	0.36786909	2.27E-06	0.04144134	0.427	0.169
NBEAL1	8	0.34825743	7.69E-09	0.00014037	0.988	1
FAP	8	0.32736112	6.70E-08	0.00122208	0.561	0.214
F13A1	8	0.30745996	6.68E-08	0.0012191	0.378	0.091
MIF	8	0.30223851	1.85E-06	0.03379039	1	1
AC090498.1	8	0.25097494	5.24E-07	0.00956791	1	1
FTL	8	-0.2715407	2.42E-07	0.00442234	1	1
NDUFA4L2	8	-0.3309727	6.26E-11	1.14E-06	1	1
H3F3B	8	-0.3455344	3.89E-07	0.00708938	1	1
CST3	8	-0.3683667	2.41E-06	0.04405357	0.988	1
B2M	8	-0.3705566	1.49E-07	0.00271111	0.963	1
PNRC1	8	-0.3902565	1.20E-06	0.02192492	0.951	0.994
PAM	8	-0.4014643	5.14E-07	0.00938037	0.817	0.87
TMEM59	8	-0.4059515	2.33E-07	0.00424281	0.915	0.994
EID1	8	-0.4324038	2.19E-08	0.00040021	0.854	0.968
FXYD1	8	-0.4518105	1.65E-06	0.03003618	0.488	0.708
CD59	8	-0.4559233	7.66E-08	0.00139844	0.939	0.974
SOX9	8	-0.4603468	8.81E-08	0.00160725	0.768	0.883
SMOC2	8	-0.4747687	8.51E-07	0.01552111	0.146	0.455

PRELP	8	-0.4814939	1.48E-07	0.00270601	0.927	0.994
LUM	8	-0.4882483	1.74E-06	0.03180573	1	1
SPON2	8	-0.4919094	5.85E-07	0.01068281	0.122	0.435
OGN	8	-0.5173398	3.56E-07	0.00649598	0.805	0.961
COL11A1	8	-0.520912	1.88E-08	0.0003427	0.951	0.994
FGFBP2	8	-0.5665503	2.51E-07	0.00457567	0.927	0.994
SNAI2	8	-0.6157036	5.61E-07	0.01023765	0.488	0.747
ID1	8	-0.6736771	8.10E-08	0.00147723	0.732	0.864
DCN	8	-0.6750162	7.18E-14	1.31E-09	1	1
SCRG1	8	-0.6947551	8.22E-12	1.50E-07	1	1
WWP2	8	-0.7096718	3.34E-11	6.10E-07	0.585	0.857
ID3	8	-0.7764782	2.84E-09	5.17E-05	0.622	0.831
COL9A2	8	-0.7926951	1.27E-13	2.31E-09	0.488	0.812
COL11A2	8	-0.796883	5.26E-17	9.59E-13	0.634	0.929
CP	8	-0.8038154	1.32E-07	0.00240781	0.378	0.649
CCDC80	8	-0.9337075	5.65E-17	1.03E-12	0.902	0.994
CTSD	8	-0.9935417	1.06E-12	1.93E-08	0.78	0.968
LECT1	8	-1.0381677	8.56E-19	1.56E-14	0.841	0.994
COL9A3	8	-1.0770878	1.07E-15	1.95E-11	0.634	0.916
CHI3L2	8	-1.0772329	4.24E-13	7.73E-09	0.537	0.87
ACAN	8	-1.1256622	1.68E-29	3.07E-25	0.976	1
PLA2G2A	8	-1.1393716	6.48E-09	0.0001183	0.537	0.838
EPYC	8	-1.232519	3.61E-19	6.58E-15	0.195	0.792
SLPI	8	-2.1891482	1.33E-21	2.42E-17	0.854	1
**gene**	**cluster**	**avg_logFC**	**p_val**	**p_val_adj**	**pct.1**	**pct.2**
COL1A1	9	1.39798857	8.24E-12	1.50E-07	0.682	0.218
LGALS1	9	1.09960113	1.46E-13	2.67E-09	1	1
TMSB4X	9	0.85065271	3.30E-08	0.00060272	0.977	0.941
IGFBP3	9	0.74293088	5.65E-07	0.01031221	0.773	0.465
MT2A	9	0.69799691	2.55E-06	0.04653782	1	1
TPM1	9	0.69214646	3.12E-08	0.0005688	0.943	0.752
PFN1	9	0.65176639	8.07E-08	0.00147264	0.977	0.941
TNC	9	0.64156704	4.60E-09	8.40E-05	0.739	0.366
TMSB10	9	0.63346019	4.80E-10	8.76E-06	1	1
CFL1	9	0.60663193	8.06E-07	0.01469958	0.943	0.911
KIAA0101	9	0.50488794	1.28E-06	0.02342925	0.364	0.069
SERPINE2	9	0.48623384	1.83E-07	0.00333949	1	1
COL8A1	9	0.44734849	4.31E-07	0.00786033	0.398	0.089
YBX1	9	0.43054451	1.29E-07	0.00235703	0.989	0.96

SLC25A5	9	0.41781463	1.94E-06	0.03540033	0.716	0.436
UBA52	9	0.26718949	4.72E-08	0.00086171	1	1
SMIM5	9	-0.2750615	1.80E-06	0.03279382	0.023	0.277
B2M	9	-0.3083427	1.04E-06	0.01903614	1	1
FTL	9	-0.3237178	2.67E-07	0.00487642	1	1
ATRAID	9	-0.3813654	1.41E-06	0.0257879	0.943	0.99
FCGRT	9	-0.3986188	2.10E-06	0.03832051	0.659	0.842
HLA-C	9	-0.4003638	3.35E-08	0.000611	0.966	0.98
ECM2	9	-0.4063279	1.49E-06	0.02711172	0.352	0.663
CTSF	9	-0.4137999	6.63E-07	0.01209523	0.318	0.653
TPD52	9	-0.4282083	2.63E-06	0.04796181	0.352	0.653
HLA-A	9	-0.4346465	2.26E-08	0.00041227	0.989	1
COL11A1	9	-0.4674691	1.46E-06	0.02659627	0.932	0.98
GRN	9	-0.4676883	2.74E-07	0.00500705	0.795	0.941
TMEM59	9	-0.4707853	7.91E-13	1.44E-08	0.989	1
HLA-B	9	-0.4846041	1.47E-06	0.02682092	0.841	0.931
PAM	9	-0.5085874	3.88E-10	7.08E-06	0.557	0.921
HAPLN1	9	-0.5193235	6.68E-07	0.01219247	0.784	0.98
CST3	9	-0.5319118	2.36E-11	4.32E-07	0.977	1
BAMBI	9	-0.5326065	1.79E-06	0.03263368	0.409	0.743
ITM2B	9	-0.5333631	3.24E-13	5.92E-09	1	1
FRZB	9	-0.546231	1.59E-06	0.02907769	0.159	0.485
PSAP	9	-0.5467054	6.86E-11	1.25E-06	0.818	1
ITM2A	9	-0.5504735	5.23E-09	9.55E-05	0.545	0.881
PCOLCE2	9	-0.5624954	1.34E-11	2.45E-07	0.955	0.99
C1R	9	-0.5639472	9.95E-09	0.00018149	0.636	0.931
LUM	9	-0.5699103	2.79E-10	5.09E-06	1	1
MFGE8	9	-0.5729386	1.39E-07	0.00252778	0.807	0.931
PLD3	9	-0.5897016	2.08E-10	3.79E-06	0.784	0.97
HIF1A	9	-0.5974347	1.11E-08	0.00020327	0.886	0.97
PNRC1	9	-0.5998152	1.50E-08	0.00027288	0.648	0.931
IFITM1	9	-0.6181644	2.20E-06	0.04015896	0.42	0.723
SOD3	9	-0.6317179	5.07E-10	9.25E-06	0.739	0.98
FMOD	9	-0.6331945	1.73E-12	3.16E-08	0.864	0.99
SERPING1	9	-0.6387451	2.75E-10	5.02E-06	0.523	0.871
OMD	9	-0.6447086	3.34E-07	0.006095	0.17	0.495
SERPINA5	9	-0.6669775	6.61E-08	0.00120589	0.693	0.891
CRYAB	9	-0.7099523	1.67E-06	0.03039921	0.852	0.931
IFI6	9	-0.7145317	1.85E-06	0.03381844	0.455	0.693

SERPINA1	9	-0.7234854	4.14E-09	7.56E-05	0.727	0.911
PRELP	9	-0.7317149	5.94E-09	0.00010848	0.943	0.97
CLU	9	-0.7790291	1.49E-09	2.71E-05	0.955	1
S100A1	9	-0.7841069	1.95E-11	3.56E-07	0.83	0.96
MIA	9	-0.7908137	2.88E-14	5.25E-10	0.83	0.99
C1S	9	-0.798925	3.92E-16	7.16E-12	0.636	0.97
PLAC9	9	-0.8049827	4.48E-11	8.18E-07	0.75	0.96
FBXO2	9	-0.8172528	1.07E-13	1.95E-09	0.432	0.881
DCN	9	-0.8240009	1.69E-16	3.09E-12	1	1
COL2A1	9	-0.8757948	7.44E-10	1.36E-05	0.511	0.871
SCRG1	9	-0.9289418	2.94E-16	5.37E-12	0.966	1
CP	9	-0.9403715	7.17E-12	1.31E-07	0.398	0.842
COL11A2	9	-0.9971962	9.86E-15	1.80E-10	0.261	0.772
COL9A2	9	-1.0424615	1.54E-15	2.81E-11	0.352	0.842
ACAN	9	-1.0614819	6.73E-16	1.23E-11	0.83	0.98
C2orf40	9	-1.0629174	1.15E-12	2.09E-08	0.83	0.99
CHI3L1	9	-1.0723743	1.56E-11	2.84E-07	0.511	0.921
CTSD	9	-1.0928467	9.07E-15	1.65E-10	0.955	1
FGFBP2	9	-1.1347167	9.00E-12	1.64E-07	0.58	0.97
OGN	9	-1.1534404	6.40E-14	1.17E-09	0.636	0.96
CFD	9	-1.1572019	3.42E-11	6.23E-07	0.125	0.574
C2orf82	9	-1.2214605	3.63E-07	0.00661673	0.739	0.881
PLA2G2A	9	-1.355084	4.33E-08	0.00078961	0.443	0.752
CHI3L2	9	-1.3609886	5.48E-13	1.00E-08	0.625	0.901
COL9A3	9	-1.3928477	1.02E-17	1.86E-13	0.42	0.911
MATN4	9	-1.398729	3.05E-11	5.56E-07	0.023	0.446
LECT1	9	-1.6831382	8.88E-20	1.62E-15	0.625	0.95
EPYC	9	-1.8643891	3.76E-20	6.86E-16	0.307	0.901
SLPI	9	-1.9370569	1.02E-13	1.86E-09	0.648	0.931
**gene**	**cluster**	**avg_logFC**	**p_val**	**p_val_adj**	**pct.1**	**pct.2**
CD55	10	0.89802673	9.35E-19	1.71E-14	0.966	0.663
HMGA1	10	0.77945856	1.00E-12	1.83E-08	0.862	0.49
GSTO1	10	0.52538989	2.34E-11	4.28E-07	0.924	0.633
GYPC	10	0.48157707	1.44E-07	0.00262608	0.683	0.418
SNHG12	10	0.40842271	3.47E-07	0.00633494	0.8	0.561
EIF1	10	0.33623053	1.48E-09	2.71E-05	1	1
ZFAS1	10	0.33161637	2.14E-08	0.00039013	1	0.99
CSNK2B	10	0.30763745	1.52E-06	0.02768885	0.876	0.694
AC090498.1	10	0.30283124	1.50E-12	2.74E-08	1	1

EEF1A1	10	-0.2923275	2.70E-09	4.92E-05	1	1
FTL	10	-0.3217065	2.18E-08	0.00039756	1	1
RTN4	10	-0.3331979	1.31E-07	0.00239461	0.683	0.918
RNF152	10	-0.347595	7.19E-08	0.00131227	0.103	0.388
SMOC2	10	-0.350381	2.63E-07	0.0047957	0.021	0.224
CTSF	10	-0.3530518	2.97E-10	5.42E-06	0.083	0.418
FXYD1	10	-0.3540259	1.23E-06	0.02244548	0.11	0.357
PGK1	10	-0.3553986	2.23E-06	0.04067402	0.91	0.98
FKBP9	10	-0.3558343	1.53E-06	0.02791304	0.138	0.398
ISLR	10	-0.3578647	3.71E-10	6.77E-06	0.034	0.327
SPON2	10	-0.3652746	2.62E-08	0.00047834	0.048	0.306
ANGPTL2	10	-0.369419	3.37E-07	0.0061503	0.214	0.5
LY6E	10	-0.3709322	9.57E-07	0.01745575	0.276	0.551
NUCB1	10	-0.378222	9.42E-08	0.00171957	0.379	0.673
NDUFA4L2	10	-0.3843359	4.97E-13	9.06E-09	1	1
DDR2	10	-0.3862261	9.80E-08	0.00178824	0.214	0.52
SAT1	10	-0.3942477	7.10E-07	0.0129486	0.931	0.969
COL11A2	10	-0.4075977	3.53E-07	0.00643405	0.193	0.5
MALAT1	10	-0.4142152	6.56E-09	0.00011979	1	1
IFITM2	10	-0.4178094	1.01E-06	0.01849511	0.683	0.878
LSP1	10	-0.4285802	1.46E-08	0.00026604	0.09	0.388
CFD	10	-0.4288075	5.08E-08	0.00092709	0.062	0.327
B2M	10	-0.4319323	1.94E-09	3.54E-05	0.959	1
HSP90B1	10	-0.4322072	4.98E-08	0.00090961	0.593	0.847
HTRA1	10	-0.4374885	1.88E-06	0.03437545	0.524	0.776
AHNAK	10	-0.4413632	6.71E-09	0.00012239	0.186	0.52
PPP3CA	10	-0.4423493	1.11E-10	2.02E-06	0.993	1
CDK5RAP2	10	-0.4548244	2.30E-06	0.0419928	0.11	0.357
CA9	10	-0.4619705	1.59E-06	0.02906921	0.338	0.592
VCAN	10	-0.4662993	3.48E-08	0.00063474	0.11	0.408
CLU	10	-0.4724588	1.65E-07	0.00300457	0.993	1
CD99	10	-0.4737398	3.18E-08	0.0005794	0.786	0.929
AEBP1	10	-0.476232	6.15E-07	0.01122729	0.476	0.724
FGFBP2	10	-0.4776013	9.14E-07	0.01668133	0.945	1
COL9A3	10	-0.4841396	3.09E-08	0.00056451	0.241	0.582
PCOLCE2	10	-0.4866455	1.39E-08	0.00025417	0.71	0.918
HIF1A	10	-0.5103886	3.27E-09	5.97E-05	0.724	0.939
FXYD6	10	-0.5129334	3.27E-08	0.0005972	0.572	0.806
CCDC85B	10	-0.5132589	5.28E-08	0.00096282	0.931	0.99

EPYC	10	-0.5227945	5.16E-11	9.42E-07	0.034	0.347
SUMF2	10	-0.535955	3.24E-09	5.91E-05	0.441	0.735
WWP2	10	-0.5394701	1.80E-09	3.28E-05	0.49	0.806
PAM	10	-0.5419408	4.04E-09	7.37E-05	0.352	0.673
PHKG1	10	-0.5428466	1.33E-08	0.00024358	0.414	0.714
GAPDH	10	-0.5449471	1.42E-08	0.00025899	1	1
LCN2	10	-0.5716217	1.32E-06	0.02402527	0.248	0.52
C10orf10	10	-0.5836004	1.23E-06	0.02243663	0.414	0.684
NGFRAP1	10	-0.5837114	1.51E-10	2.75E-06	0.545	0.796
VKORC1	10	-0.60939	8.04E-13	1.47E-08	0.855	0.969
SCRG1	10	-0.6168635	5.45E-07	0.00993646	0.959	1
DCN	10	-0.6185831	5.54E-17	1.01E-12	1	1
CLEC3A	10	-0.6188237	9.61E-09	0.00017533	0.041	0.306
FMOD	10	-0.6530762	3.90E-11	7.13E-07	0.621	0.827
CST3	10	-0.6587792	2.66E-15	4.86E-11	0.945	1
MT1F	10	-0.8127184	2.31E-10	4.21E-06	0.814	0.929
IGFBP5	10	-0.8128161	1.03E-09	1.89E-05	0.2	0.551
S100B	10	-0.8156481	5.76E-14	1.05E-09	0.462	0.827
ID3	10	-0.8197042	1.75E-08	0.00031885	0.11	0.408
COL11A1	10	-0.8530227	2.47E-12	4.51E-08	0.372	0.765
CHI3L1	10	-0.8661591	1.78E-11	3.25E-07	0.814	0.969
C2orf40	10	-0.8688759	3.88E-15	7.08E-11	0.945	0.99
OGN	10	-0.881907	4.83E-12	8.82E-08	0.559	0.878
RBP4	10	-0.912633	2.93E-16	5.34E-12	0.338	0.786
COL2A1	10	-0.9316731	6.34E-11	1.16E-06	0.159	0.531
PLAC9	10	-0.9405189	1.02E-13	1.86E-09	0.345	0.755
MT1G	10	-0.9410638	4.88E-10	8.90E-06	0.931	0.969
LECT1	10	-0.9411687	1.77E-16	3.23E-12	0.31	0.816
CHI3L2	10	-0.9994682	4.82E-16	8.80E-12	0.497	0.888
ACAN	10	-1.0890372	1.49E-20	2.72E-16	0.752	0.99
SLPI	10	-1.1112803	7.89E-17	1.44E-12	0.71	0.98
PRELP	10	-1.2103476	3.05E-26	5.57E-22	0.676	1
CCDC80	10	-1.3027906	6.12E-20	1.12E-15	0.676	0.98
PLA2G2A	10	-1.3047457	2.47E-15	4.50E-11	0.255	0.724
CTSD	10	-1.5622504	2.56E-21	4.66E-17	0.738	0.959
**gene**	**cluster**	**avg_logFC**	**p_val**	**p_val_adj**	**pct.1**	**pct.2**
HMGA1	11	0.79798069	1.92E-06	0.03506226	0.658	0.329
NBEAL1	11	0.50571889	1.62E-09	2.96E-05	0.973	0.943
AC090498.1	11	0.45632901	1.12E-11	2.04E-07	1	1

ZFAS1	11	0.45100381	9.85E-09	0.00017981	1	0.986
RPS27	11	0.38525666	2.28E-10	4.17E-06	1	1
RPL10	11	0.31408661	4.93E-07	0.00900054	1	1
RPL27A	11	0.3020781	5.01E-07	0.00913497	1	1
RPL37	11	0.29405514	1.64E-06	0.0298854	1	1
RPL41	11	0.28949612	1.04E-07	0.00190414	1	1
BSG	11	-0.4453307	8.63E-07	0.01575029	0.575	0.857
CD99	11	-0.4841788	2.07E-06	0.03774976	0.753	0.943
SAT1	11	-0.530885	1.56E-06	0.02843739	0.945	1
PKM	11	-0.6415108	1.76E-08	0.00032196	0.849	0.971
CST3	11	-0.7107374	6.98E-08	0.00127399	0.918	0.957
RBP4	11	-0.9261666	1.04E-07	0.00189056	0.288	0.686
CTSD	11	-1.0821183	7.13E-08	0.0013005	0.836	0.957
**gene**	**cluster**	**avg_logFC**	**p_val**	**p_val_adj**	**pct.1**	**pct.2**
SPP1	12	1.5785977	1.10E-07	0.00200131	0.964	0.75
RGCC	12	0.94321239	3.90E-13	7.12E-09	0.973	0.68
UPP1	12	0.8020883	7.68E-15	1.40E-10	0.893	0.48
SMOX	12	0.7037554	2.64E-07	0.00480914	0.545	0.25
C11orf96	12	0.69299959	1.22E-07	0.00223203	0.464	0.16
TIMP3	12	0.66800106	2.49E-07	0.0045489	0.83	0.55
TM4SF1	12	0.65108152	7.69E-07	0.01403156	0.83	0.53
HMGA1	12	0.62890811	3.04E-09	5.55E-05	0.696	0.34
NRP2	12	0.59458981	1.30E-07	0.00237112	0.652	0.35
CD55	12	0.57294562	8.14E-10	1.48E-05	0.982	0.82
RGS16	12	0.5285252	7.57E-07	0.01381685	0.402	0.1
HMGA2	12	0.40630757	3.54E-07	0.00646541	0.384	0.08
EPB41L4A-AS1	12	0.38901955	1.05E-06	0.01916744	0.946	0.75
EIF1	12	0.25342743	1.38E-06	0.02523355	1	1
CLEC3A	12	-0.3205568	5.75E-07	0.01048898	0.107	0.41
NDUFA4L2	12	-0.3412137	3.26E-10	5.94E-06	1	1
SMOC2	12	-0.3557542	4.05E-07	0.00739017	0.009	0.23
EID1	12	-0.3646431	9.94E-08	0.00181424	0.661	0.93
ITM2B	12	-0.3841854	1.03E-07	0.00187683	0.982	1
BSG	12	-0.3939436	1.11E-06	0.02029951	0.473	0.76
SPON2	12	-0.3965762	9.39E-08	0.00171304	0.009	0.25
TPM2	12	-0.4072027	2.07E-06	0.03770118	0.321	0.62
FKBP9	12	-0.4072413	6.25E-09	0.00011406	0.062	0.39
BHLHE41	12	-0.4131321	1.08E-08	0.0001974	0.071	0.4
HSP90B1	12	-0.4209162	4.03E-07	0.00735289	0.679	0.89

PRSS23	12	-0.4234836	2.61E-06	0.04767754	0.071	0.32
NGFRAP1	12	-0.4389715	9.45E-07	0.01724834	0.589	0.84
CTSF	12	-0.4584872	2.84E-10	5.18E-06	0.143	0.54
GPX3	12	-0.4640689	1.47E-06	0.02674746	0.821	0.92
IFITM3	12	-0.4643997	2.26E-08	0.00041168	0.795	0.94
GSN	12	-0.4647266	3.23E-07	0.00589657	0.339	0.62
PRDX2	12	-0.4651727	2.64E-07	0.00481944	0.625	0.79
HLA-A	12	-0.5005931	4.42E-09	8.06E-05	0.902	0.99
PRDX4	12	-0.5032896	3.94E-08	0.00071932	0.679	0.87
CST3	12	-0.5164763	2.37E-10	4.32E-06	0.982	1
VKORC1	12	-0.5170917	7.26E-12	1.32E-07	0.875	0.99
GAPDH	12	-0.5184145	1.21E-11	2.22E-07	1	1
PSAP	12	-0.5211663	1.38E-09	2.52E-05	0.902	0.95
CLU	12	-0.5275094	1.43E-06	0.02618343	1	1
CD99	12	-0.5397005	8.86E-12	1.62E-07	0.768	0.92
LRPAP1	12	-0.5418798	1.26E-09	2.30E-05	0.286	0.65
ATRAID	12	-0.5428334	3.08E-10	5.61E-06	0.714	0.87
TGFBI	12	-0.5446234	1.84E-06	0.0336277	0.562	0.82
PKM	12	-0.5543996	6.95E-13	1.27E-08	0.964	0.99
SERPING1	12	-0.577861	9.94E-10	1.81E-05	0.58	0.88
DCN	12	-0.6014961	7.59E-18	1.39E-13	1	1
FMOD	12	-0.6080249	9.20E-12	1.68E-07	0.848	0.98
WWP2	12	-0.6241543	1.72E-08	0.00031437	0.5	0.74
BAMBI	12	-0.6251588	1.53E-07	0.0027901	0.339	0.69
HIF1A	12	-0.6493446	2.70E-08	0.000493	0.821	0.98
C1S	12	-0.6644386	3.59E-11	6.55E-07	0.589	0.85
ACAN	12	-0.6785155	3.51E-10	6.40E-06	0.75	0.96
S100B	12	-0.6835325	1.07E-07	0.00195981	0.598	0.75
CA9	12	-0.7000715	3.04E-10	5.55E-06	0.5	0.77
COL11A1	12	-0.7084512	9.73E-13	1.78E-08	0.268	0.73
CCDC80	12	-0.7298994	1.03E-08	0.00018852	0.875	0.98
PLAC9	12	-0.7453679	1.35E-08	0.00024646	0.357	0.72
RBP4	12	-0.7471364	2.67E-11	4.88E-07	0.527	0.86
CHI3L2	12	-0.7474423	1.37E-07	0.00249313	0.786	0.96
C2orf40	12	-0.755014	2.38E-14	4.35E-10	1	1
LECT1	12	-0.8037324	3.20E-11	5.84E-07	0.732	0.95
PRELP	12	-0.830346	1.12E-16	2.05E-12	0.982	0.99
LCN2	12	-0.8498634	4.57E-08	0.00083344	0.554	0.84
PLA2G2A	12	-0.9210331	1.58E-09	2.88E-05	0.348	0.77

IGFBP6	12	-0.9612557	5.07E-10	9.25E-06	0.482	0.79
MT1F	12	-0.9905928	8.81E-08	0.00160729	0.759	0.92
MT1G	12	-1.0912614	4.17E-08	0.00076155	0.911	1
CTSD	12	-1.6742918	4.57E-23	8.34E-19	0.732	0.98
**gene**	**cluster**	**avg_logFC**	**p_val**	**p_val_adj**	**pct.1**	**pct.2**
MT2A	13	0.82780234	7.55E-07	0.01377231	1	1
PAPSS1	13	-0.4560737	1.50E-06	0.02728784	0.227	0.7
NDUFA4L2	13	-0.4627149	2.19E-06	0.03988547	0.979	1
EBF1	13	-0.4900283	1.71E-09	3.12E-05	0.062	0.533
B2M	13	-0.6233797	1.34E-07	0.00244962	1	1
COL9A2	13	-0.6446695	4.85E-08	0.00088559	0.134	0.6
COL11A2	13	-0.7365852	1.64E-06	0.02999717	0.134	0.5
HAPLN1	13	-0.7864822	9.93E-07	0.01811648	0.495	0.9
C1S	13	-0.8121438	7.95E-09	0.00014512	0.216	0.7
CFD	13	-0.9111493	6.69E-07	0.01221458	0.052	0.4
BAMBI	13	-0.9927656	3.80E-08	0.00069353	0.196	0.667
DCN	13	-1.0638704	6.82E-08	0.00124502	0.918	1
COL9A3	13	-1.0971406	7.00E-10	1.28E-05	0.216	0.767
SCRG1	13	-1.2424167	7.68E-08	0.0014013	0.918	0.967
CHI3L1	13	-1.2895858	3.26E-08	0.00059478	0.299	0.8
COL2A1	13	-1.2999444	6.58E-07	0.01201391	0.433	0.833
C2orf40	13	-1.3046669	1.26E-09	2.30E-05	0.577	0.967
ACAN	13	-1.3304183	4.23E-11	7.72E-07	0.68	0.933
EPYC	13	-1.4634658	1.01E-09	1.83E-05	0.062	0.533
CTSD	13	-1.542369	1.39E-12	2.53E-08	0.608	1
LECT1	13	-1.5692981	1.29E-09	2.35E-05	0.402	0.833
PLA2G2A	13	-1.6608087	3.69E-10	6.74E-06	0.361	0.867
CHI3L2	13	-1.6996067	9.85E-13	1.80E-08	0.33	0.933
SLPI	13	-1.8486423	7.38E-10	1.35E-05	0.423	0.9
