# Supplementary material for: Single-Cell RNA-Seq Analysis of Cells from Degenerating and Non-Degenerating Intervertebral Discs from the Same Individual Reveals New Biomarkers for Intervertebral Disc Degeneration
Source: Int J Mol Sci. 2022 Apr 3;23(7):3993. doi: 10.3390/ijms23073993 (PMC8999935; doi:10.3390/ijms23073993)
Supplement: Supplementary file 1 [file ijms-23-03993-s001.zip › ijms-1655681-SI/Table S12.pdf]

Table S12: Differentially expressed genes between AFD and AFnD

gene	cluster	avg_logFC	p_val	p_val_adj	pct.1	pct.2
SPP1	0	2.57023305	2.83E-124	5.17E-120	0.753	0.064
PRG4	0	1.54276463	2.78E-75	5.07E-71	0.498	0.024
COMP	0	1.4564712	6.75E-99	1.23E-94	0.966	0.613
PTGS2	0	1.33641275	4.56E-105	8.32E-101	0.686	0.076
VEGFA	0	1.33312822	9.00E-117	1.64E-112	0.874	0.324
CHI3L2	0	1.26218405	1.06E-86	1.93E-82	0.912	0.497
G0S2	0	1.19193541	8.62E-20	1.57E-15	0.395	0.174
MT2A	0	1.18135357	5.16E-72	9.42E-68	1	0.997
MT1X	0	1.13400835	2.23E-51	4.07E-47	1	0.995
CCDC85B	0	1.10699512	2.68E-80	4.89E-76	0.949	0.702
WTAP	0	1.10181237	2.30E-72	4.19E-68	0.908	0.578
SERPINE2	0	1.09700184	4.66E-82	8.50E-78	0.998	0.986
PHLDA1	0	1.06406901	3.31E-63	6.04E-59	0.469	0.043
IGFBP3	0	1.05209957	1.41E-58	2.58E-54	0.433	0.036
S100A2	0	1.02945893	7.50E-46	1.37E-41	0.637	0.291
PLA2G2A	0	1.00790305	5.41E-55	9.88E-51	0.7	0.251
UPP1	0	0.97409631	6.48E-76	1.18E-71	0.621	0.133
MT1G	0	0.97053609	2.77E-35	5.05E-31	0.958	0.898
TGFBI	0	0.97043607	3.93E-83	7.17E-79	0.677	0.141
ERO1A	0	0.93333075	1.67E-72	3.05E-68	0.742	0.312
FN1	0	0.89669996	2.87E-98	5.23E-94	1	0.998
BMP2	0	0.87468122	9.39E-67	1.71E-62	0.464	0.028
MRPS6	0	0.87106892	4.75E-40	8.68E-36	0.948	0.905
PPP3CA	0	0.854816	1.82E-71	3.31E-67	0.93	0.585
CTSD	0	0.84592868	2.09E-29	3.81E-25	0.969	0.938
DDIT4L	0	0.81556649	6.31E-68	1.15E-63	0.926	0.552
ADM	0	0.7981543	1.06E-43	1.93E-39	0.854	0.583
BHLHE40	0	0.79308981	1.64E-45	3.00E-41	0.677	0.329
TMEM45A	0	0.78046516	9.74E-57	1.78E-52	0.917	0.716
STK17A	0	0.77699538	5.52E-56	1.01E-51	0.543	0.129
IGFBP5	0	0.76784334	3.36E-47	6.14E-43	0.428	0.067
ARF6	0	0.76517891	4.36E-61	7.95E-57	0.742	0.401
MT1H	0	0.74073325	6.32E-12	1.15E-07	0.675	0.546
CTSL	0	0.73292609	1.75E-23	3.19E-19	0.659	0.468
TNFRSF12A	0	0.73101355	1.02E-40	1.86E-36	0.451	0.115
BZW1	0	0.72436973	6.62E-59	1.21E-54	0.778	0.408

HILPDA	0	0.72170638	1.33E-45	2.42E-41	0.626	0.25
TREM1	0	0.7061875	6.19E-74	1.13E-69	0.475	0.012
SOD2	0	0.70064745	1.15E-41	2.10E-37	1	0.966
CTHRC1	0	0.69561282	7.47E-65	1.36E-60	0.484	0.046
FOSL1	0	0.68089746	1.18E-59	2.15E-55	0.417	0.021
TIMP1	0	0.65820307	1.99E-17	3.64E-13	0.996	0.998
SLC3A2	0	0.64765613	6.32E-32	1.15E-27	0.616	0.344
ENO2	0	0.64649715	1.74E-50	3.17E-46	0.7	0.315
CA9	0	0.63736859	2.37E-48	4.32E-44	0.563	0.174
NDRG1	0	0.6345852	2.38E-42	4.35E-38	0.724	0.411
FAM162A	0	0.62946033	1.30E-52	2.38E-48	0.957	0.8
RNF145	0	0.62622107	1.46E-44	2.66E-40	0.585	0.219
SLC2A1	0	0.61486164	8.61E-41	1.57E-36	0.52	0.177
PGK1	0	0.60509615	1.11E-47	2.02E-43	0.953	0.878
PTP4A1	0	0.59990636	3.77E-32	6.88E-28	0.652	0.404
PDE4B	0	0.59557382	1.54E-53	2.81E-49	0.558	0.134
EGLN3	0	0.59324109	3.89E-41	7.10E-37	0.525	0.17
ARRDC3	0	0.57326062	1.26E-19	2.30E-15	0.532	0.324
PPP1CB	0	0.57314315	2.59E-27	4.73E-23	0.832	0.644
CRTAC1	0	0.57207933	1.63E-48	2.98E-44	0.493	0.112
GAPDH	0	0.56821964	3.54E-44	6.46E-40	1	1
NRP2	0	0.565826	3.51E-36	6.41E-32	0.403	0.095
PPP1R15A	0	0.5616194	2.21E-39	4.02E-35	0.56	0.222
ORM1	0	0.56081399	1.03E-08	0.00018875	0.383	0.246
PPFIBP1	0	0.56073728	4.90E-30	8.94E-26	0.601	0.348
HLA-DRB1	0	0.56036782	4.28E-39	7.81E-35	0.534	0.201
SLC25A37	0	0.55991999	1.55E-41	2.82E-37	0.897	0.668
AK2	0	0.55580456	1.12E-41	2.04E-37	0.819	0.587
PMP22	0	0.54908564	1.42E-33	2.59E-29	0.848	0.625
SGIP1	0	0.54598083	1.15E-53	2.10E-49	0.401	0.029
VAPA	0	0.52554077	2.27E-48	4.15E-44	0.964	0.842
NAMPT	0	0.51059213	4.08E-31	7.45E-27	0.697	0.425
ATP6V1G1	0	0.50606359	3.53E-48	6.44E-44	0.993	0.955
MT1F	0	0.50538905	8.41E-14	1.53E-09	0.91	0.869
WDR45B	0	0.50319133	1.03E-38	1.88E-34	0.579	0.241
EMP3	0	0.49957119	3.52E-38	6.43E-34	0.919	0.78
GBE1	0	0.49508247	4.28E-25	7.81E-21	0.722	0.539
ARPP19	0	0.491237	1.02E-34	1.86E-30	0.704	0.425
ARF4	0	0.48739634	5.29E-32	9.64E-28	0.785	0.575

GOLT1B	0	0.48657387	1.06E-40	1.93E-36	0.542	0.189
DNAJB9	0	0.48249053	6.07E-32	1.11E-27	0.758	0.522
SPRY2	0	0.46859692	1.41E-36	2.57E-32	0.354	0.059
CLK1	0	0.46551444	1.86E-34	3.39E-30	0.74	0.446
BTG1	0	0.45360596	1.13E-23	2.06E-19	0.989	0.974
WWP2	0	0.44420283	1.44E-21	2.63E-17	0.713	0.513
NT5C3A	0	0.4431571	1.53E-25	2.79E-21	0.46	0.2
SAR1A	0	0.44234708	9.91E-34	1.81E-29	0.621	0.301
INSIG2	0	0.43928706	2.78E-18	5.08E-14	0.599	0.43
RNMT	0	0.43736782	5.30E-24	9.67E-20	0.551	0.308
SLC5A3	0	0.43450182	4.41E-18	8.05E-14	0.578	0.372
SH3BP5	0	0.433618	6.68E-22	1.22E-17	0.579	0.346
HSPA5	0	0.4274981	4.44E-14	8.10E-10	0.509	0.332
ERRFI1	0	0.42678325	8.64E-18	1.58E-13	0.399	0.186
WDR33	0	0.42677	3.00E-25	5.47E-21	0.601	0.361
FGFBP2	0	0.42671048	1.94E-19	3.53E-15	0.966	0.914
ISG20	0	0.42506703	9.79E-21	1.79E-16	0.605	0.392
SLC39A8	0	0.42104845	1.29E-26	2.36E-22	0.525	0.256
HMGA1	0	0.42000124	6.55E-13	1.19E-08	0.404	0.238
RAB13	0	0.4184382	4.75E-30	8.67E-26	0.933	0.831
GPX1	0	0.41649906	6.93E-27	1.26E-22	0.921	0.83
XBP1	0	0.41592116	5.95E-21	1.09E-16	0.693	0.516
ENO1	0	0.41555348	1.70E-22	3.09E-18	0.998	0.983
DDIT3	0	0.41551487	9.33E-22	1.70E-17	0.648	0.399
IFRD1	0	0.41477745	3.64E-26	6.63E-22	0.742	0.527
GRB10	0	0.41373976	1.02E-33	1.86E-29	0.253	0.012
CSGALNACT2	0	0.41100294	4.77E-25	8.70E-21	0.413	0.157
C4orf48	0	0.40953343	1.66E-31	3.03E-27	0.428	0.127
NPR3	0	0.40818493	3.41E-41	6.22E-37	0.287	0.007
COX17	0	0.40036541	4.37E-27	7.97E-23	0.807	0.623
TMEM38B	0	0.39916839	1.28E-23	2.34E-19	0.671	0.449
RABGGTB	0	0.39754429	1.12E-25	2.04E-21	0.791	0.616
ZNF385D	0	0.39505137	1.22E-22	2.22E-18	0.749	0.532
SLC25A36	0	0.38965099	6.39E-28	1.17E-23	0.652	0.361
BAALC	0	0.38837797	5.73E-18	1.04E-13	0.505	0.286
TFPI	0	0.38197232	8.09E-29	1.48E-24	0.301	0.053
CD44	0	0.38111769	6.47E-25	1.18E-20	0.951	0.785
CAPZA2	0	0.37981467	3.40E-24	6.21E-20	0.753	0.575
RELB	0	0.37760797	9.80E-23	1.79E-18	0.417	0.169

MED21	0	0.37724821	7.06E-20	1.29E-15	0.581	0.367
ATP6V0E1	0	0.37688982	2.52E-27	4.59E-23	0.919	0.824
CLEC2B	0	0.37476552	1.19E-08	0.00021683	0.285	0.155
EPS8	0	0.37410798	2.23E-24	4.08E-20	0.442	0.179
RAB1A	0	0.37267944	2.16E-22	3.95E-18	0.785	0.675
MIF	0	0.37082763	1.16E-40	2.12E-36	1	1
CDK5RAP2	0	0.36670705	2.97E-13	5.42E-09	0.305	0.134
FAM210A	0	0.36317036	1.36E-26	2.49E-22	0.458	0.186
KLF3	0	0.36113752	6.63E-17	1.21E-12	0.417	0.208
BNIP3	0	0.36065951	1.28E-20	2.33E-16	0.998	0.997
GLRX	0	0.35531239	5.34E-08	0.00097381	0.422	0.293
C11orf96	0	0.35141613	1.28E-20	2.33E-16	0.285	0.077
EIF4EBP1	0	0.35078785	1.21E-11	2.20E-07	0.565	0.423
EVI2A	0	0.34780392	1.41E-30	2.58E-26	0.383	0.096
PLAUR	0	0.34737224	3.24E-23	5.91E-19	0.428	0.174
HMGA2	0	0.3438079	5.68E-38	1.04E-33	0.26	0.003
SMIM15	0	0.3429076	6.33E-18	1.16E-13	0.536	0.336
DNER	0	0.34260159	1.82E-13	3.31E-09	0.57	0.391
CLDND1	0	0.3414832	6.95E-23	1.27E-18	0.565	0.317
CTNNB1	0	0.33982693	5.93E-12	1.08E-07	0.401	0.231
GUK1	0	0.33973742	5.42E-28	9.90E-24	0.94	0.821
SMIM3	0	0.33879964	4.02E-31	7.33E-27	0.354	0.076
SELK	0	0.33871696	1.31E-25	2.39E-21	0.895	0.797
TM4SF1	0	0.33722445	1.53E-06	0.02786366	0.484	0.372
SDCBP	0	0.33502181	3.05E-15	5.56E-11	0.652	0.463
RGS16	0	0.33348338	4.16E-14	7.58E-10	0.291	0.114
RSRC2	0	0.33305575	3.14E-18	5.73E-14	0.605	0.392
STK26	0	0.33220066	2.48E-28	4.53E-24	0.384	0.108
PTGES3	0	0.33091337	1.17E-27	2.14E-23	0.984	0.943
SERPINE1	0	0.33064337	3.91E-28	7.14E-24	0.244	0.024
SNRNP27	0	0.32981854	5.38E-22	9.82E-18	0.529	0.281
ADAMTS6	0	0.32511989	1.11E-16	2.02E-12	0.218	0.053
POLR2H	0	0.32284921	1.44E-21	2.63E-17	0.616	0.367
MMP3	0	0.32024126	1.69E-08	0.00030805	0.883	0.785
SEC61G	0	0.31754639	2.72E-20	4.97E-16	0.993	0.991
C15orf48	0	0.3161471	7.20E-21	1.31E-16	0.179	0.015
VDAC1	0	0.31612047	1.69E-14	3.08E-10	0.856	0.823
DDIT4	0	0.31555938	1.32E-11	2.41E-07	0.558	0.384
CCNG2	0	0.31386815	1.55E-12	2.82E-08	0.558	0.401

EGR1	0	0.31286721	8.20E-11	1.50E-06	0.403	0.241
MSANTD3	0	0.31117059	1.47E-23	2.69E-19	0.43	0.17
A4GALT	0	0.31086102	1.78E-33	3.25E-29	0.26	0.015
AK4	0	0.30716611	1.48E-18	2.69E-14	0.547	0.308
NTMT1	0	0.30709727	1.01E-19	1.84E-15	0.545	0.303
RNF126	0	0.30455342	3.56E-22	6.50E-18	0.401	0.153
METAP2	0	0.30412284	9.24E-20	1.69E-15	0.836	0.685
UBL3	0	0.30366947	3.40E-16	6.20E-12	0.567	0.355
CFD	0	0.30217626	8.62E-15	1.57E-10	0.307	0.115
NBPF14	0	0.30141876	7.78E-25	1.42E-20	0.383	0.126
PLA2G4A	0	0.30066771	6.72E-22	1.23E-17	0.408	0.16
HMGN1	0	0.30065704	3.29E-14	6.00E-10	0.841	0.762
IER3	0	0.30034454	3.74E-14	6.82E-10	0.296	0.117
PID1	0	0.29986622	2.53E-12	4.62E-08	0.606	0.448
SYPL1	0	0.2997512	1.89E-16	3.45E-12	0.745	0.582
TMSB4X	0	0.29945314	6.28E-08	0.00114616	0.726	0.614
C11orf31	0	0.2944233	5.19E-16	9.47E-12	0.778	0.671
CLDN12	0	0.29440262	4.15E-21	7.57E-17	0.448	0.198
PHPT1	0	0.29094434	3.70E-16	6.76E-12	0.8	0.656
SEP7	0	0.28997641	3.54E-12	6.45E-08	0.619	0.472
PLCG2	0	0.28780161	1.85E-17	3.38E-13	0.197	0.038
CD55	0	0.28721678	8.20E-13	1.50E-08	0.662	0.491
PCSK1N	0	0.28710851	1.45E-16	2.64E-12	0.287	0.098
TRMT112	0	0.28637881	8.80E-19	1.61E-14	0.942	0.893
CDC37L1	0	0.28484276	3.09E-17	5.64E-13	0.511	0.287
NDUFC1	0	0.28227855	9.64E-17	1.76E-12	0.89	0.799
ZNF593	0	0.27934841	6.69E-17	1.22E-12	0.59	0.365
SNHG12	0	0.27894724	2.86E-11	5.22E-07	0.605	0.454
IL11	0	0.27826132	9.04E-17	1.65E-12	0.117	0.002
WSB1	0	0.27781814	4.66E-18	8.50E-14	0.868	0.73
ATP6V1C1	0	0.27777545	2.84E-16	5.18E-12	0.507	0.299
RGCC	0	0.27693045	7.04E-11	1.28E-06	0.751	0.645
EPYC	0	0.27665264	5.48E-19	1.00E-14	0.206	0.036
NDFIP1	0	0.27652776	4.01E-15	7.31E-11	0.717	0.565
SLC7A2	0	0.27604878	3.52E-26	6.42E-22	0.236	0.026
NOL7	0	0.2753535	6.06E-13	1.11E-08	0.697	0.549
VIMP	0	0.27521007	2.63E-13	4.79E-09	0.708	0.537
RAB7A	0	0.2751193	2.12E-12	3.87E-08	0.704	0.577
CNIH1	0	0.27509962	1.11E-16	2.03E-12	0.809	0.67

PRR34-AS1	0	0.27415588	8.50E-24	1.55E-19	0.329	0.09
CLIC4	0	0.27403385	5.29E-13	9.65E-09	0.529	0.36
TNFSF11	0	0.27311992	9.10E-30	1.66E-25	0.209	0.003
SOX9	0	0.27287785	2.40E-08	0.00043773	0.659	0.578
SLC20A1	0	0.27134106	5.75E-15	1.05E-10	0.292	0.114
KAT5	0	0.26991296	1.75E-14	3.19E-10	0.43	0.239
PAPOLA	0	0.26964404	1.05E-13	1.91E-09	0.688	0.54
EIF1B	0	0.26939441	3.68E-15	6.71E-11	0.769	0.661
DDX41	0	0.26922473	1.79E-15	3.26E-11	0.319	0.133
RP11-125O18.1	0	0.26857677	7.48E-15	1.37E-10	0.271	0.096
SAT1	0	0.26776057	6.00E-13	1.09E-08	0.98	0.928
RORA	0	0.26698664	7.24E-15	1.32E-10	0.509	0.312
RNF114	0	0.26591498	3.52E-11	6.42E-07	0.625	0.509
MOB4	0	0.2653403	6.44E-17	1.17E-12	0.413	0.203
ANKRD37	0	0.26377352	2.57E-10	4.70E-06	0.43	0.274
MRPL14	0	0.26299951	2.84E-13	5.18E-09	0.616	0.454
RAB14	0	0.26249707	4.46E-11	8.14E-07	0.478	0.334
SFT2D1	0	0.26112793	1.09E-11	1.98E-07	0.727	0.616
RTN4	0	0.26009026	6.03E-11	1.10E-06	0.653	0.525
POMP	0	0.25971375	4.10E-16	7.49E-12	0.87	0.783
TIMM10B	0	0.25965402	7.39E-18	1.35E-13	0.383	0.165
WBP5	0	0.25963073	1.80E-09	3.29E-05	0.74	0.657
CCND1	0	0.25957413	7.36E-22	1.34E-17	0.283	0.069
OAZ1	0	0.25932842	8.97E-23	1.64E-18	0.998	0.99
CTTN	0	0.2593066	2.65E-09	4.83E-05	0.451	0.312
TMSB10	0	0.25902604	1.29E-08	0.00023614	0.993	0.983
TNS1	0	0.25631973	8.51E-18	1.55E-13	0.352	0.136
TVP23B	0	0.25624256	2.90E-16	5.29E-12	0.442	0.227
GPRC5A	0	0.2559583	4.73E-14	8.63E-10	0.314	0.136
AVPI1	0	0.25557356	3.35E-11	6.12E-07	0.365	0.201
DNAJB1	0	0.25470229	1.38E-06	0.02522532	0.307	0.194
MGST1	0	0.2542484	1.41E-11	2.57E-07	0.442	0.256
MTHFD2	0	0.25357654	7.79E-07	0.01420537	0.626	0.556
SRSF1	0	0.25308436	4.04E-10	7.37E-06	0.44	0.293
SDHA	0	0.25264387	2.89E-13	5.28E-09	0.357	0.177
CHMP4B	0	0.25244758	1.90E-12	3.46E-08	0.69	0.547
EIF4A2	0	0.251918	8.23E-20	1.50E-15	0.995	1
BTG3	0	0.25152322	5.75E-11	1.05E-06	0.435	0.274
TNFAIP6	0	0.25105006	3.37E-21	6.15E-17	0.161	0.007

PTX3	0	0.2506984	3.14E-10	5.74E-06	0.116	0.022
ENAH	0	-0.2500025	3.79E-14	6.92E-10	0.182	0.373
IFI27L2	0	-0.2501032	2.11E-13	3.86E-09	0.767	0.892
MRPS21	0	-0.251191	1.72E-16	3.14E-12	0.83	0.935
MZT2B	0	-0.2527523	4.45E-19	8.12E-15	0.915	0.983
ATP5L	0	-0.2528027	3.35E-18	6.12E-14	0.935	0.986
DNAJC1	0	-0.25304	5.51E-13	1.01E-08	0.245	0.423
DIO2	0	-0.2542139	2.78E-12	5.07E-08	0.116	0.277
PTPRD-AS1	0	-0.2546948	1.90E-13	3.46E-09	0.323	0.516
ATPIF1	0	-0.2547331	1.93E-13	3.53E-09	0.556	0.744
GOLIM4	0	-0.2555817	1.91E-08	0.00034921	0.287	0.437
DAAM1	0	-0.2558896	9.83E-14	1.79E-09	0.193	0.382
EIF3M	0	-0.2564523	2.24E-14	4.08E-10	0.587	0.756
C12orf57	0	-0.2566581	1.75E-20	3.18E-16	0.951	0.988
SLC44A1	0	-0.2579536	1.94E-14	3.54E-10	0.171	0.363
NFIC	0	-0.2587521	2.64E-12	4.82E-08	0.296	0.468
RPL5	0	-0.2591308	1.33E-48	2.43E-44	1	1
MTDH	0	-0.2593068	9.91E-14	1.81E-09	0.538	0.702
PABPC4	0	-0.259677	4.23E-11	7.72E-07	0.542	0.685
KRT18	0	-0.2598986	3.50E-13	6.39E-09	0.193	0.38
EFNA1	0	-0.2603807	1.03E-16	1.88E-12	0.218	0.444
NACA	0	-0.2629044	1.45E-40	2.65E-36	1	1
AC005152.3	0	-0.2629172	1.16E-27	2.12E-23	0.032	0.263
SERPINI1	0	-0.2629803	7.84E-09	0.00014312	0.13	0.258
ANG	0	-0.2634136	4.02E-17	7.33E-13	0.224	0.448
FCGRT	0	-0.263836	1.03E-12	1.88E-08	0.507	0.652
COX7C	0	-0.2655107	1.49E-39	2.71E-35	1	1
PPIC	0	-0.2655539	1.50E-14	2.73E-10	0.166	0.356
CCNG1	0	-0.2663217	1.10E-15	2.00E-11	0.424	0.635
H1FX	0	-0.2667846	6.10E-19	1.11E-14	0.128	0.349
PLPP1	0	-0.267141	7.90E-17	1.44E-12	0.099	0.287
MAGED1	0	-0.267365	2.65E-11	4.83E-07	0.09	0.234
ATP5H	0	-0.267495	9.21E-16	1.68E-11	0.612	0.762
RPS15A	0	-0.2680127	2.24E-51	4.09E-47	1	1
ENPP1	0	-0.2683314	4.75E-20	8.67E-16	0.119	0.348
RARRES3	0	-0.2707128	6.86E-26	1.25E-21	0.054	0.289
MIA	0	-0.27207	2.53E-11	4.62E-07	0.939	0.966
BSG	0	-0.2722308	2.60E-16	4.74E-12	0.561	0.759
PRKCSH	0	-0.2723875	9.29E-14	1.70E-09	0.327	0.504

MPST	0	-0.2736187	5.16E-20	9.41E-16	0.327	0.578
RPS21	0	-0.2740218	2.44E-41	4.46E-37	0.998	1
RPA3	0	-0.2749046	9.02E-16	1.65E-11	0.384	0.589
C1orf21	0	-0.275225	5.13E-18	9.35E-14	0.408	0.642
RPS4X	0	-0.2757485	4.63E-66	8.46E-62	1	1
PLP2	0	-0.2764062	6.10E-17	1.11E-12	0.722	0.88
RBP4	0	-0.2768237	4.12E-16	7.52E-12	0.617	0.818
SNRPE	0	-0.2773969	1.72E-15	3.13E-11	0.606	0.757
PPA1	0	-0.277767	1.37E-16	2.49E-12	0.356	0.563
FAM200B	0	-0.2781441	4.17E-16	7.62E-12	0.258	0.468
SNRPA	0	-0.2787717	9.76E-22	1.78E-17	0.164	0.413
AP2M1	0	-0.2798015	2.50E-14	4.57E-10	0.464	0.625
MOXD1	0	-0.280918	1.91E-19	3.48E-15	0.106	0.322
RP11-983P16.4	0	-0.2809693	2.76E-20	5.04E-16	0.285	0.554
RPS3	0	-0.2816281	3.37E-68	6.15E-64	1	1
SOCS3	0	-0.2817068	2.21E-21	4.03E-17	0.13	0.372
RPL11	0	-0.2824429	1.82E-70	3.32E-66	1	1
RPL28	0	-0.2825902	2.11E-58	3.84E-54	1	1
RPL12	0	-0.2831471	5.77E-53	1.05E-48	1	1
GYPC	0	-0.2832294	9.79E-13	1.79E-08	0.518	0.685
ARPC1A	0	-0.2834029	1.31E-16	2.39E-12	0.365	0.583
MSRB1	0	-0.2834893	1.23E-19	2.24E-15	0.137	0.361
HSPD1	0	-0.2867537	1.65E-13	3.02E-09	0.57	0.699
TCEAL8	0	-0.2875418	1.13E-19	2.06E-15	0.65	0.842
GLTSCR2	0	-0.2891186	1.16E-24	2.12E-20	0.937	0.981
RPL15	0	-0.2897806	1.23E-83	2.25E-79	1	1
SNAI2	0	-0.28988	1.57E-10	2.87E-06	0.31	0.482
SNHG8	0	-0.2899284	3.66E-20	6.67E-16	0.821	0.95
PPT1	0	-0.2900564	6.65E-18	1.21E-13	0.251	0.478
ST13	0	-0.290139	8.35E-26	1.52E-21	0.962	0.99
DBP	0	-0.2911877	2.24E-17	4.09E-13	0.186	0.403
PDLIM4	0	-0.2912296	4.06E-14	7.42E-10	0.76	0.854
ANGPTL2	0	-0.2918173	1.81E-14	3.30E-10	0.366	0.568
CHMP3	0	-0.2929285	1.28E-16	2.34E-12	0.437	0.654
CRYAB	0	-0.2934538	5.15E-10	9.39E-06	0.626	0.787
RPL27A	0	-0.2951791	4.22E-58	7.71E-54	1	1
BEX2	0	-0.2960886	1.31E-19	2.39E-15	0.247	0.501
ITM2A	0	-0.2966835	1.30E-18	2.37E-14	0.345	0.597
KHDRBS3	0	-0.2994683	9.90E-29	1.81E-24	0.018	0.241

ERGIC3	0	-0.3004659	2.11E-19	3.85E-15	0.722	0.892
RNF130	0	-0.3015111	1.42E-18	2.59E-14	0.477	0.695
RPL22	0	-0.3015537	1.28E-55	2.33E-51	1	1
ZNF90	0	-0.3021543	3.86E-19	7.05E-15	0.518	0.704
CMB9-22P13.1	0	-0.3025595	9.01E-17	1.64E-12	0.321	0.535
HSPB1	0	-0.3025602	2.88E-17	5.25E-13	0.579	0.778
FBXO2	0	-0.3046345	2.29E-19	4.18E-15	0.659	0.866
GALNT15	0	-0.3046345	6.17E-25	1.13E-20	0.106	0.365
NINJ1	0	-0.3051826	1.26E-21	2.30E-17	0.466	0.73
SSR4	0	-0.3061951	8.42E-18	1.54E-13	0.903	0.95
C19orf43	0	-0.3074905	3.66E-24	6.68E-20	0.794	0.938
GSTP1	0	-0.3083137	1.76E-26	3.21E-22	0.912	0.976
TSPO	0	-0.3083398	3.73E-22	6.81E-18	0.706	0.895
SPINT2	0	-0.3085165	2.16E-22	3.95E-18	0.204	0.466
COX7A2L	0	-0.308926	1.45E-25	2.65E-21	0.859	0.95
RPS24	0	-0.3093853	2.71E-51	4.94E-47	1	1
FXYD6	0	-0.3094189	3.68E-23	6.71E-19	0.702	0.902
EIF3F	0	-0.3099469	1.69E-23	3.08E-19	0.805	0.91
ATP5A1	0	-0.3102596	7.69E-20	1.40E-15	0.489	0.699
VCAN	0	-0.3109459	2.84E-09	5.18E-05	0.292	0.43
RPL39	0	-0.3116243	4.78E-59	8.73E-55	1	1
LITAF	0	-0.3117201	6.70E-20	1.22E-15	0.357	0.592
NBEAL1	0	-0.3119976	3.19E-31	5.82E-27	0.986	0.995
DNM3OS	0	-0.3121658	3.12E-20	5.70E-16	0.13	0.36
TTC3	0	-0.3125165	2.09E-21	3.82E-17	0.561	0.809
RPS9	0	-0.3128804	4.83E-74	8.80E-70	1	1
PIGP	0	-0.3129466	2.25E-19	4.11E-15	0.514	0.728
EIF2S3	0	-0.3140207	5.37E-17	9.80E-13	0.527	0.702
RPS6	0	-0.3158735	3.74E-87	6.82E-83	1	1
SP5	0	-0.3161039	2.68E-29	4.88E-25	0	0.205
ATP5D	0	-0.317219	5.18E-25	9.45E-21	0.834	0.938
SDF4	0	-0.3188984	4.76E-20	8.69E-16	0.505	0.756
RPL35A	0	-0.3211479	1.08E-73	1.97E-69	1	1
RPL36AL	0	-0.3235237	3.02E-47	5.50E-43	1	1
MYLK	0	-0.3243686	3.42E-21	6.24E-17	0.097	0.32
IGBP1	0	-0.3245853	4.08E-20	7.44E-16	0.619	0.788
EIF3E	0	-0.324623	1.21E-39	2.20E-35	1	1
RPS25	0	-0.325148	1.01E-80	1.85E-76	1	1
RBMX	0	-0.3270604	9.04E-20	1.65E-15	0.682	0.835

AHNAK2	0	-0.328842	1.32E-17	2.40E-13	0.296	0.52
COMMD6	0	-0.3289618	7.04E-40	1.28E-35	1	1
MYL12B	0	-0.3302377	1.17E-21	2.14E-17	0.439	0.67
LDHB	0	-0.3303779	9.10E-24	1.66E-19	0.745	0.888
RPS27	0	-0.3312591	9.34E-90	1.70E-85	1	1
HERPUD1	0	-0.3329362	2.24E-15	4.08E-11	0.394	0.578
ITM2C	0	-0.3330021	1.96E-20	3.58E-16	0.318	0.57
AC003090.1	0	-0.3333594	4.62E-34	8.43E-30	0.04	0.317
PDE4DIP	0	-0.3335863	1.32E-21	2.40E-17	0.273	0.525
TUBB2B	0	-0.3337498	3.48E-14	6.35E-10	0.244	0.437
EID1	0	-0.3345177	3.40E-32	6.21E-28	0.919	0.985
SLC29A1	0	-0.3349206	1.25E-23	2.28E-19	0.245	0.522
RPS12	0	-0.3364233	8.55E-69	1.56E-64	1	1
RPL7A	0	-0.3379401	7.16E-76	1.31E-71	1	1
ILF3-AS1	0	-0.3387185	6.40E-24	1.17E-19	0.209	0.472
KRT8	0	-0.338758	4.03E-20	7.36E-16	0.204	0.444
CUTA	0	-0.3390564	2.80E-26	5.11E-22	0.742	0.914
NUPR1	0	-0.3406909	1.70E-16	3.10E-12	0.978	0.991
LINC01503	0	-0.3409566	4.95E-16	9.03E-12	0.244	0.446
RPL27	0	-0.3425985	4.83E-53	8.82E-49	1	1
RPL35	0	-0.3426458	1.18E-73	2.15E-69	1	1
CHID1	0	-0.3460536	7.17E-20	1.31E-15	0.415	0.625
LTA4H	0	-0.3476435	6.33E-23	1.16E-18	0.522	0.721
RPL18A	0	-0.3483844	6.39E-83	1.17E-78	1	1
SSPN	0	-0.3485477	3.27E-25	5.96E-21	0.137	0.403
MALAT1	0	-0.3491655	4.47E-22	8.15E-18	1	1
RPL13	0	-0.3493834	5.49E-99	1.00E-94	1	1
PDIA6	0	-0.3510824	4.16E-20	7.59E-16	0.426	0.666
S100A13	0	-0.3518527	1.03E-38	1.88E-34	0.978	1
DMKN	0	-0.3520139	6.41E-24	1.17E-19	0.256	0.525
CRIP2	0	-0.3526196	3.19E-23	5.82E-19	0.217	0.472
UQCRH	0	-0.3540857	6.75E-36	1.23E-31	0.926	0.981
FHL1	0	-0.3544008	4.91E-18	8.97E-14	0.287	0.503
PPIA	0	-0.3570007	1.93E-37	3.51E-33	0.93	0.986
RPS5	0	-0.3573963	1.17E-76	2.14E-72	1	1
ZBTB20	0	-0.357879	1.63E-25	2.97E-21	0.2	0.472
UBA52	0	-0.3586053	1.68E-78	3.06E-74	1	1
HDLBP	0	-0.3586937	1.06E-20	1.94E-16	0.363	0.589
RPL37A	0	-0.3607762	8.20E-94	1.50E-89	1	1

RPLP1	0	-0.3616779	3.47E-96	6.34E-92	1	1
YBX1	0	-0.3617449	4.09E-42	7.47E-38	0.971	1
PTRF	0	-0.3646415	2.33E-24	4.26E-20	0.319	0.575
RPL29	0	-0.3663656	9.31E-81	1.70E-76	1	1
FMOD	0	-0.367116	3.61E-30	6.58E-26	0.912	0.981
HMGN2	0	-0.3683847	7.51E-31	1.37E-26	0.863	0.978
BRK1	0	-0.3686013	1.07E-31	1.95E-27	0.749	0.94
RPL3	0	-0.3688829	5.20E-86	9.49E-82	1	1
PLA2G16	0	-0.3712764	1.09E-24	1.98E-20	0.213	0.478
GNAS	0	-0.3717164	2.88E-27	5.25E-23	0.477	0.726
PTMS	0	-0.3721142	2.75E-29	5.02E-25	0.502	0.795
EIF3K	0	-0.3744505	3.73E-32	6.80E-28	0.787	0.943
NGFRAP1	0	-0.3746134	4.78E-28	8.72E-24	0.764	0.947
PSMA7	0	-0.3751671	1.12E-29	2.05E-25	0.785	0.919
RPL21	0	-0.375305	3.98E-107	7.26E-103	1	1
NFIA	0	-0.3779827	7.83E-29	1.43E-24	0.188	0.478
ATP5O	0	-0.3781198	2.94E-29	5.36E-25	0.736	0.907
PRCP	0	-0.3806253	1.29E-35	2.36E-31	0.112	0.435
RPS18	0	-0.3810791	7.07E-97	1.29E-92	1	1
RPL31	0	-0.3821947	1.96E-79	3.58E-75	1	1
HOXC10	0	-0.3822607	1.76E-25	3.21E-21	0.65	0.847
RPS7	0	-0.385376	6.89E-79	1.26E-74	1	1
PCBP2	0	-0.3853832	1.36E-39	2.48E-35	0.897	0.981
PSAP	0	-0.3882713	1.00E-31	1.83E-27	0.899	0.976
HSP90AB1	0	-0.389608	9.21E-35	1.68E-30	0.968	0.993
EIF3D	0	-0.3904094	8.65E-33	1.58E-28	0.738	0.902
RABAC1	0	-0.3924675	6.54E-33	1.19E-28	0.675	0.885
MYL6	0	-0.3929202	4.61E-36	8.40E-32	0.839	0.972
NMRK1	0	-0.3934604	2.11E-31	3.85E-27	0.255	0.561
RPL19	0	-0.3941651	9.95E-117	1.82E-112	1	1
CYBRD1	0	-0.3952185	9.16E-28	1.67E-23	0.484	0.728
TOMM7	0	-0.3972475	2.23E-86	4.08E-82	1	1
PLXDC2	0	-0.4019937	3.09E-24	5.65E-20	0.466	0.682
RGS10	0	-0.4032317	4.36E-29	7.96E-25	0.511	0.744
ATP5G2	0	-0.4038161	8.97E-55	1.64E-50	0.996	1
DDR2	0	-0.4043991	1.05E-21	1.92E-17	0.397	0.625
RPL8	0	-0.4062619	1.94E-105	3.54E-101	1	1
TPM1	0	-0.4075856	6.09E-18	1.11E-13	0.392	0.596
LRRC2	0	-0.4151504	5.69E-36	1.04E-31	0.144	0.48

S100A1	0	-0.4156035	3.87E-44	7.06E-40	0.996	1
SLC25A3	0	-0.4160111	4.80E-40	8.75E-36	0.845	0.974
CPE	0	-0.4172918	8.46E-30	1.54E-25	0.569	0.826
BTF3	0	-0.4186172	1.66E-71	3.04E-67	0.995	1
NPM1	0	-0.4189786	4.93E-62	8.99E-58	1	1
RPL10	0	-0.4225206	4.20E-115	7.67E-111	1	1
RPL14	0	-0.4254124	1.92E-108	3.50E-104	1	1
PLAC9	0	-0.4254514	1.19E-31	2.18E-27	0.653	0.917
RPS23	0	-0.4257551	1.14E-109	2.07E-105	1	1
THY1	0	-0.4276627	3.73E-28	6.80E-24	0.099	0.367
RPL13A	0	-0.4303259	4.71E-127	8.60E-123	1	1
ID1	0	-0.4318863	4.91E-21	8.96E-17	0.395	0.68
EIF2A	0	-0.437178	3.98E-37	7.26E-33	0.684	0.909
CFH	0	-0.437711	2.79E-18	5.09E-14	0.125	0.341
OXA1L	0	-0.4380044	1.11E-34	2.03E-30	0.48	0.749
RPL37	0	-0.4383207	1.43E-102	2.62E-98	1	1
NDUFA11	0	-0.4411281	1.58E-40	2.88E-36	0.749	0.928
HINT1	0	-0.4436172	6.80E-60	1.24E-55	0.958	0.998
RPL30	0	-0.4446135	2.20E-110	4.01E-106	1	1
RPL9	0	-0.4464657	2.98E-114	5.44E-110	1	1
ARHGAP29	0	-0.4464994	1.75E-26	3.18E-22	0.273	0.542
RPS13	0	-0.447895	1.33E-105	2.43E-101	1	1
RPSA	0	-0.4496293	2.57E-80	4.69E-76	1	1
RPS27A	0	-0.4544651	4.39E-123	8.01E-119	1	1
NAP1L1	0	-0.4596825	1.44E-57	2.64E-53	0.951	0.995
RPS2	0	-0.4597098	1.86E-119	3.39E-115	1	1
LECT1	0	-0.4685133	4.87E-22	8.89E-18	0.783	0.9
ATRNL1	0	-0.4717558	2.17E-42	3.96E-38	0.088	0.437
COL9A3	0	-0.473534	8.86E-24	1.62E-19	0.504	0.759
SSR2	0	-0.4746393	2.37E-48	4.32E-44	0.731	0.933
GAS6	0	-0.4782011	3.68E-33	6.71E-29	0.269	0.575
CEBPD	0	-0.4806061	3.74E-30	6.82E-26	0.773	0.94
UQCRB	0	-0.4902402	8.61E-58	1.57E-53	0.966	1
CD99	0	-0.4944199	1.38E-49	2.52E-45	0.883	0.972
TSC22D1	0	-0.4972642	1.55E-25	2.82E-21	0.579	0.823
H3F3A	0	-0.5003933	7.22E-56	1.32E-51	0.903	0.986
GREM1	0	-0.5007418	3.63E-31	6.62E-27	0.011	0.241
CTSC	0	-0.5018296	4.56E-17	8.33E-13	0.2	0.418
SEC11A	0	-0.5057992	3.63E-54	6.63E-50	0.783	0.967

RPL23A	0	-0.5093862	2.91E-135	5.31E-131	1	1
CYR61	0	-0.5126095	3.47E-29	6.32E-25	0.15	0.451
PABPC1	0	-0.513342	3.07E-61	5.60E-57	0.877	0.988
HLA-B	0	-0.5145525	3.09E-38	5.63E-34	0.718	0.919
SERPINA5	0	-0.5161674	3.05E-42	5.56E-38	0.253	0.632
RPL34	0	-0.5164448	2.81E-128	5.12E-124	1	1
RPS29	0	-0.5166689	7.53E-127	1.37E-122	1	1
CHRDL2	0	-0.5174182	1.85E-26	3.37E-22	0.135	0.403
HLA-A	0	-0.5190942	9.16E-37	1.67E-32	0.87	0.981
RPL23	0	-0.5228855	4.99E-97	9.10E-93	0.998	1
TCF4	0	-0.5230796	1.24E-39	2.27E-35	0.224	0.577
RPS3A	0	-0.5241873	3.26E-138	5.95E-134	1	1
PEBP1	0	-0.5258809	3.60E-55	6.57E-51	0.681	0.94
EEF1B2	0	-0.527837	7.71E-77	1.41E-72	0.987	0.997
ISLR	0	-0.5338022	7.47E-48	1.36E-43	0.166	0.558
CCDC102B	0	-0.5339761	1.32E-58	2.40E-54	0.029	0.437
CP	0	-0.5448195	4.66E-48	8.50E-44	0.599	0.941
MT-ND4	0	-0.5451784	1.56E-19	2.84E-15	0.955	0.905
RPS17	0	-0.5465259	3.51E-118	6.40E-114	1	1
BGN	0	-0.5502913	9.15E-58	1.67E-53	0.986	1
HIST1H4C	0	-0.551793	1.97E-37	3.59E-33	0.307	0.633
TPT1	0	-0.5520375	1.21E-122	2.21E-118	1	1
HNRNPA1	0	-0.5553224	5.76E-76	1.05E-71	0.996	1
RPS16	0	-0.5555843	1.52E-132	2.78E-128	1	1
TUBB	0	-0.55633	4.27E-52	7.80E-48	0.668	0.917
RPLP2	0	-0.5574053	3.62E-139	6.61E-135	1	1
SLC14A1	0	-0.5593469	1.39E-50	2.54E-46	0.191	0.599
CRISPLD1	0	-0.5670895	7.00E-43	1.28E-38	0.047	0.377
FTH1	0	-0.5674418	2.97E-76	5.42E-72	1	1
EIF4B	0	-0.5694374	1.05E-61	1.92E-57	0.832	0.972
EIF3L	0	-0.5731485	1.19E-70	2.18E-66	0.816	0.979
HLA-C	0	-0.5742464	3.23E-50	5.89E-46	0.836	0.966
CD9	0	-0.5773675	5.87E-45	1.07E-40	0.291	0.661
SSR3	0	-0.5836327	5.28E-52	9.64E-48	0.762	0.957
RPL18	0	-0.585663	1.47E-137	2.69E-133	1	1
NPC2	0	-0.5948873	5.08E-90	9.27E-86	0.977	1
MGP	0	-0.5954236	1.14E-37	2.07E-33	0.998	1
RPL7	0	-0.6014448	4.29E-125	7.84E-121	1	1
INHBA	0	-0.6015266	4.24E-37	7.73E-33	0.195	0.525

ACAN	0	-0.606664	3.22E-61	5.88E-57	0.924	0.998
CTGF	0	-0.6084944	1.19E-29	2.17E-25	0.206	0.506
EEF2	0	-0.6130293	2.22E-74	4.06E-70	0.998	1
NNMT	0	-0.6180746	5.92E-51	1.08E-46	0.854	0.981
EEF1A1	0	-0.6486385	1.41E-142	2.57E-138	1	1
MT-ND1	0	-0.6507158	5.10E-37	9.31E-33	0.417	0.738
RPL4	0	-0.6622202	3.29E-128	6.01E-124	1	1
PRUNE2	0	-0.6646353	1.16E-54	2.12E-50	0.22	0.649
CITED2	0	-0.6656102	1.11E-46	2.02E-42	0.2	0.587
COL2A1	0	-0.6723926	2.53E-57	4.61E-53	0.415	0.857
CCNI	0	-0.676862	1.34E-115	2.45E-111	0.998	1
P4HB	0	-0.6786	7.48E-62	1.36E-57	0.623	0.935
RPL10A	0	-0.680292	1.94E-132	3.54E-128	1	1
ID3	0	-0.6869741	8.51E-40	1.55E-35	0.323	0.714
HIST1H1C	0	-0.6950066	2.36E-34	4.31E-30	0.258	0.573
RHOBTB3	0	-0.7049755	8.19E-61	1.49E-56	0.451	0.836
VIM	0	-0.7064854	1.02E-86	1.85E-82	1	1
RPS8	0	-0.7140221	4.20E-160	7.66E-156	1	1
RPLP0	0	-0.7340358	2.49E-152	4.54E-148	1	1
MT-CO3	0	-0.7439173	1.42E-31	2.59E-27	0.821	0.874
XAGE2	0	-0.7449579	1.69E-35	3.08E-31	0.002	0.25
MT-CO2	0	-0.7473611	1.09E-31	1.98E-27	0.917	0.917
MT-ATP6	0	-0.7513142	7.14E-37	1.30E-32	0.699	0.857
YBX3	0	-0.7591452	1.08E-88	1.97E-84	0.724	0.969
SPARC	0	-0.7732216	8.73E-72	1.59E-67	0.19	0.694
SLC40A1	0	-0.7735968	2.64E-64	4.82E-60	0.065	0.513
CRTAP	0	-0.7795357	9.97E-98	1.82E-93	0.496	0.931
EIF3H	0	-0.7918116	2.08E-122	3.79E-118	0.957	1
MT-CO1	0	-0.7919248	6.30E-31	1.15E-26	0.937	0.909
B2M	0	-0.7939931	2.31E-106	4.22E-102	0.995	1
GPX3	0	-0.8024191	8.84E-79	1.61E-74	0.912	0.993
MT-CYB	0	-0.8550674	2.37E-49	4.32E-45	0.527	0.828
C2orf82	0	-0.8718169	1.06E-75	1.93E-71	0.921	1
MT-ND2	0	-0.908054	9.52E-45	1.74E-40	0.502	0.78
APOD	0	-0.9240069	6.30E-49	1.15E-44	0.171	0.561
NEAT1	0	-0.9274664	1.22E-100	2.23E-96	0.987	0.998
PAX1	0	-0.9333762	1.03E-95	1.87E-91	0.403	0.898
ACTG1	0	-0.9344587	6.02E-104	1.10E-99	0.937	0.998
CAPS	0	-0.9964306	2.83E-79	5.17E-75	0.489	0.912

RPS20	0	-1.0098311	5.00E-175	9.11E-171	0.998	1
RPS11	0	-1.0479369	3.53E-171	6.45E-167	0.987	1
MT-ND3	0	-1.0557432	1.39E-62	2.54E-58	0.489	0.809
ZFP36L2	0	-1.0654357	1.23E-70	2.24E-66	0.137	0.627
CLEC3A	0	-1.1588556	1.19E-92	2.18E-88	0.264	0.835
ADIRF	0	-1.1714212	8.10E-101	1.48E-96	0.3	0.855
OGN	0	-1.3303552	1.43E-101	2.60E-97	0.857	0.979
SPTSSB	0	-1.5514351	3.52E-128	6.42E-124	0.029	0.742
FRZB	0	-2.2033143	1.02E-126	1.85E-122	0.255	0.909
**gene**	**cluster**	**avg_logFC**	**p_val**	**p_val_adj**	**pct.1**	**pct.2**
SPP1	1	1.96075969	8.86E-108	1.62E-103	0.717	0.078
PLA2G2A	1	1.52866988	3.96E-70	7.22E-66	0.708	0.197
PRG4	1	1.3933776	2.12E-68	3.87E-64	0.479	0.04
COMP	1	1.3601691	4.12E-72	7.51E-68	0.993	0.802
CHI3L2	1	1.29754359	6.40E-85	1.17E-80	0.865	0.407
S100A2	1	1.2245374	7.80E-44	1.42E-39	0.603	0.246
VEGFA	1	1.2189331	1.95E-78	3.55E-74	0.776	0.306
PTGS2	1	1.15379578	3.02E-88	5.52E-84	0.575	0.045
MT1G	1	1.1154134	2.12E-41	3.88E-37	0.977	0.932
TGFBI	1	1.10608105	1.44E-70	2.62E-66	0.699	0.233
CTSD	1	1.0688436	2.85E-44	5.20E-40	0.989	0.94
MT1X	1	1.06235356	6.85E-56	1.25E-51	1	0.988
MT2A	1	1.00353418	5.71E-46	1.04E-41	1	0.997
PPP3CA	1	0.96623116	8.54E-72	1.56E-67	0.929	0.65
IGFBP3	1	0.92866469	6.00E-44	1.09E-39	0.379	0.055
CCDC85B	1	0.88412121	4.38E-48	8.00E-44	0.9	0.707
EPYC	1	0.87777138	2.35E-51	4.28E-47	0.539	0.131
DDIT4L	1	0.85445021	2.55E-54	4.65E-50	0.847	0.504
MRPS6	1	0.84212082	1.94E-30	3.53E-26	0.95	0.892
SOD2	1	0.83042719	6.17E-43	1.13E-38	0.982	0.894
FN1	1	0.82749888	7.58E-78	1.38E-73	1	0.995
PHLDA1	1	0.82050188	2.60E-45	4.74E-41	0.377	0.048
ADM	1	0.78626621	5.51E-28	1.01E-23	0.811	0.598
CYTL1	1	0.77992687	4.18E-25	7.64E-21	0.443	0.166
WTAP	1	0.76263308	7.75E-48	1.41E-43	0.808	0.481
DDIT3	1	0.75543177	4.65E-37	8.48E-33	0.689	0.373
MT1H	1	0.73369853	7.74E-17	1.41E-12	0.76	0.584
SLC2A1	1	0.73257448	8.42E-43	1.54E-38	0.491	0.143
CHI3L1	1	0.71095869	1.15E-21	2.09E-17	0.977	0.949

ERO1A	1	0.70902938	2.24E-40	4.09E-36	0.683	0.373
RGS2	1	0.70224149	8.94E-18	1.63E-13	0.477	0.261
SERPINE2	1	0.70058745	1.62E-32	2.95E-28	1	0.998
CCDC80	1	0.69729707	1.55E-36	2.83E-32	0.995	0.972
PTP4A1	1	0.67816037	1.62E-34	2.96E-30	0.632	0.35
TIMP1	1	0.67559793	2.73E-18	4.98E-14	1	0.998
TNFRSF12A	1	0.67336263	2.54E-25	4.64E-21	0.395	0.141
SLC3A2	1	0.66769403	1.40E-31	2.56E-27	0.548	0.249
FOSL1	1	0.65256057	6.77E-42	1.24E-37	0.317	0.026
CTHRC1	1	0.6467938	9.50E-56	1.73E-51	0.429	0.046
CA9	1	0.64518678	1.91E-30	3.49E-26	0.58	0.293
AEBP1	1	0.63603149	6.89E-41	1.26E-36	0.767	0.438
ENO2	1	0.63505147	1.30E-40	2.37E-36	0.653	0.315
NRN1	1	0.63353121	1.55E-21	2.83E-17	0.605	0.332
TMEM45A	1	0.63280694	1.54E-37	2.81E-33	0.927	0.797
BTG1	1	0.62532098	1.38E-46	2.51E-42	0.986	0.919
CFD	1	0.62360965	8.81E-32	1.61E-27	0.308	0.049
BMP2	1	0.59242567	6.16E-42	1.12E-37	0.333	0.034
CD44	1	0.57556886	1.62E-48	2.95E-44	0.9	0.535
MMP3	1	0.57319222	9.38E-18	1.71E-13	0.918	0.826
EGLN3	1	0.57238174	7.16E-34	1.31E-29	0.482	0.164
BZW1	1	0.56914875	1.19E-31	2.17E-27	0.655	0.382
STK17A	1	0.56151406	9.76E-27	1.78E-22	0.402	0.135
BHLHE40	1	0.560565	3.64E-22	6.65E-18	0.582	0.349
PPP1CB	1	0.54377092	2.34E-20	4.28E-16	0.845	0.702
HILPDA	1	0.5330127	2.79E-23	5.09E-19	0.555	0.289
C4orf48	1	0.52892028	9.27E-40	1.69E-35	0.555	0.187
ORM1	1	0.52590823	2.03E-19	3.71E-15	0.365	0.137
IGFBP5	1	0.51981203	1.46E-26	2.66E-22	0.352	0.097
ARRDC3	1	0.51530484	1.45E-16	2.64E-12	0.463	0.261
IFRD1	1	0.51143925	4.10E-31	7.48E-27	0.71	0.43
UPP1	1	0.50516334	2.54E-33	4.63E-29	0.42	0.12
SLC25A37	1	0.50322481	3.61E-33	6.59E-29	0.836	0.578
TPD52	1	0.50172587	2.44E-34	4.44E-30	0.817	0.568
GBE1	1	0.49909578	9.30E-18	1.70E-13	0.68	0.518
IBSP	1	0.49309012	7.91E-13	1.44E-08	0.139	0.026
AK2	1	0.49230539	2.80E-25	5.10E-21	0.769	0.604
FAM162A	1	0.49037068	6.73E-29	1.23E-24	0.947	0.876
NOVA1	1	0.47814924	6.26E-14	1.14E-09	0.916	0.865

ARF6	1	0.47577645	1.05E-26	1.92E-22	0.623	0.367
GAPDH	1	0.47211195	4.74E-13	8.65E-09	1	1
TREM1	1	0.47105081	1.22E-56	2.22E-52	0.356	0.008
NDRG1	1	0.46711678	5.16E-27	9.42E-23	0.616	0.338
NT5C3A	1	0.46562543	1.72E-23	3.14E-19	0.413	0.164
VAPA	1	0.45500878	1.23E-33	2.24E-29	0.925	0.854
MT1F	1	0.44943567	5.00E-11	9.12E-07	0.957	0.937
HLA-DRB1	1	0.44843223	5.78E-33	1.05E-28	0.461	0.152
PPP1R15A	1	0.44610444	1.33E-30	2.43E-26	0.411	0.123
CD55	1	0.44265096	2.73E-22	4.97E-18	0.648	0.418
SPRY2	1	0.44074249	1.32E-32	2.41E-28	0.292	0.04
PMP22	1	0.43949949	9.16E-17	1.67E-12	0.772	0.604
PLA2G4A	1	0.43902086	8.85E-40	1.62E-35	0.452	0.112
PDE4B	1	0.43804764	1.52E-36	2.77E-32	0.397	0.084
EGR1	1	0.43524182	1.08E-15	1.98E-11	0.438	0.243
ATP6V1G1	1	0.4301069	4.61E-39	8.42E-35	0.986	0.942
RABGGTB	1	0.43004921	1.34E-30	2.45E-26	0.751	0.487
STK26	1	0.41676104	2.76E-29	5.03E-25	0.505	0.198
DNER	1	0.41570882	3.27E-21	5.96E-17	0.527	0.281
PGK1	1	0.41151462	2.74E-16	5.00E-12	0.97	0.934
DNAJB9	1	0.40650693	3.31E-20	6.05E-16	0.703	0.501
ADAMTS6	1	0.40355293	4.46E-30	8.13E-26	0.299	0.052
C10orf10	1	0.39948053	2.16E-10	3.94E-06	0.584	0.438
RNF145	1	0.39159037	1.01E-21	1.84E-17	0.484	0.229
WWP2	1	0.38971766	9.61E-18	1.75E-13	0.82	0.677
INSIG2	1	0.38641189	7.46E-13	1.36E-08	0.603	0.464
GPX1	1	0.3818058	2.03E-23	3.70E-19	0.897	0.759
NAMPT	1	0.38094516	1.35E-26	2.47E-22	0.639	0.336
RP11-125O18.1	1	0.38054237	2.97E-32	5.41E-28	0.352	0.072
ARPP19	1	0.37979351	1.55E-18	2.83E-14	0.626	0.442
CTSL	1	0.37783465	4.78E-09	8.72E-05	0.507	0.372
WDR45B	1	0.3766582	5.38E-20	9.82E-16	0.459	0.227
ARF4	1	0.36799722	1.66E-18	3.02E-14	0.744	0.588
RAB1A	1	0.3651727	5.37E-20	9.80E-16	0.804	0.67
BAALC	1	0.36477552	6.50E-14	1.19E-09	0.507	0.32
CRLF1	1	0.35879442	5.10E-23	9.30E-19	0.425	0.166
EMP3	1	0.35647066	1.12E-19	2.05E-15	0.918	0.854
XBP1	1	0.35641304	1.45E-14	2.65E-10	0.687	0.519
SLC39A8	1	0.3559052	8.28E-21	1.51E-16	0.514	0.266

ERRFI1	1	0.35029132	1.39E-11	2.53E-07	0.358	0.189
CXCL14	1	0.34651374	1.92E-13	3.51E-09	0.103	0.008
PAM	1	0.34150175	1.18E-18	2.16E-14	0.806	0.659
GOLT1B	1	0.34006106	8.79E-22	1.60E-17	0.47	0.214
RNMT	1	0.33945092	4.37E-14	7.97E-10	0.495	0.306
WDR33	1	0.33688833	1.74E-14	3.17E-10	0.548	0.359
CLK1	1	0.33675497	2.25E-19	4.10E-15	0.667	0.43
COX17	1	0.33372785	3.71E-20	6.77E-16	0.84	0.648
BNIP3	1	0.33265295	2.43E-16	4.43E-12	0.998	0.998
C11orf96	1	0.3322331	7.96E-21	1.45E-16	0.235	0.048
PLAUR	1	0.33192183	2.14E-18	3.91E-14	0.4	0.174
PDPN	1	0.32948448	8.75E-08	0.00159578	0.664	0.565
CDK5RAP2	1	0.32942091	4.77E-08	0.0008706	0.263	0.141
MTHFD2	1	0.32928002	9.27E-16	1.69E-11	0.671	0.462
ISG20	1	0.3289252	4.78E-14	8.72E-10	0.507	0.312
SAT1	1	0.32737713	9.35E-20	1.71E-15	0.991	0.962
SMOC2	1	0.32623408	3.36E-22	6.13E-18	0.32	0.089
CRTAC1	1	0.32390772	3.82E-17	6.98E-13	0.402	0.183
SLC5A3	1	0.32155147	3.68E-08	0.00067223	0.534	0.409
PPFIBP1	1	0.31895226	1.21E-11	2.20E-07	0.5	0.339
PTGES3	1	0.31756441	1.81E-21	3.30E-17	0.941	0.911
HIF1A	1	0.31756026	5.69E-15	1.04E-10	0.945	0.879
C11orf31	1	0.31735002	4.08E-18	7.45E-14	0.795	0.641
MED21	1	0.31731697	2.38E-16	4.34E-12	0.541	0.33
AVPI1	1	0.31676166	1.16E-13	2.12E-09	0.397	0.215
CCNG2	1	0.31185241	5.05E-10	9.21E-06	0.491	0.344
CLU	1	0.3108765	2.27E-12	4.15E-08	1	1
TMEM38B	1	0.31003981	2.35E-14	4.29E-10	0.543	0.355
NPR3	1	0.3099752	3.72E-33	6.78E-29	0.212	0.003
CLIC4	1	0.30974449	7.33E-15	1.34E-10	0.568	0.364
SLC25A36	1	0.30927935	1.11E-17	2.03E-13	0.635	0.401
DDX41	1	0.30878783	5.36E-17	9.78E-13	0.313	0.118
NTMT1	1	0.30707498	1.59E-17	2.91E-13	0.489	0.264
DDIT4	1	0.30501119	9.62E-07	0.01756174	0.534	0.416
COL10A1	1	0.30484128	1.28E-13	2.33E-09	0.112	0.011
CTNNB1	1	0.30211627	2.84E-08	0.00051739	0.447	0.303
CD68	1	0.30195095	3.38E-14	6.16E-10	0.637	0.425
SRGN	1	0.30139199	2.27E-12	4.15E-08	0.484	0.289
ZNF385D	1	0.29975831	5.33E-13	9.72E-09	0.658	0.476

SNRNP27	1	0.29812585	5.63E-18	1.03E-13	0.518	0.289
TSHZ2	1	0.29557676	5.95E-30	1.08E-25	0.237	0.02
EIF4EBP1	1	0.29165889	3.59E-10	6.55E-06	0.546	0.387
SGIP1	1	0.28824925	5.08E-25	9.28E-21	0.205	0.02
MIF	1	0.28659628	5.45E-26	9.95E-22	1	1
SEP7	1	0.28630953	5.51E-12	1.00E-07	0.664	0.515
NOL3	1	0.28524515	7.58E-12	1.38E-07	0.53	0.346
EPS8	1	0.28430481	3.57E-15	6.51E-11	0.352	0.157
CLDND1	1	0.28361757	1.97E-14	3.59E-10	0.457	0.261
ATP6V0E1	1	0.28336601	2.16E-16	3.94E-12	0.911	0.857
C4orf3	1	0.2823558	8.36E-21	1.53E-16	0.998	0.975
PCSK1N	1	0.27933517	1.64E-09	2.99E-05	0.365	0.203
WSB1	1	0.2785077	3.78E-15	6.89E-11	0.87	0.757
TNFRSF11B	1	0.27577172	1.01E-06	0.018516	0.669	0.565
SAR1A	1	0.2738137	6.11E-14	1.11E-09	0.605	0.419
ANGPTL4	1	0.26915731	1.39E-12	2.53E-08	0.306	0.134
RPS27L	1	0.26696987	4.52E-13	8.25E-09	0.968	0.932
SLC20A1	1	0.26456096	1.59E-14	2.90E-10	0.249	0.084
RAB7A	1	0.2626497	6.62E-10	1.21E-05	0.683	0.594
GPRC5A	1	0.2605895	1.89E-15	3.44E-11	0.272	0.095
RNF126	1	0.25962078	1.80E-13	3.29E-09	0.352	0.169
PID1	1	0.25886907	5.93E-09	0.00010822	0.546	0.404
EIF4A2	1	0.25840376	4.79E-15	8.75E-11	0.998	0.994
TRMT112	1	0.25830499	4.46E-18	8.14E-14	0.943	0.883
MEF2A	1	0.25735993	8.19E-13	1.49E-08	0.493	0.301
ANKRD37	1	0.25735392	1.50E-07	0.00274357	0.374	0.252
CLDN12	1	0.25695815	9.29E-16	1.69E-11	0.422	0.206
RTN4	1	0.25676992	1.35E-11	2.47E-07	0.694	0.536
C7orf50	1	0.25644566	4.72E-09	8.61E-05	0.632	0.512
EVI2A	1	0.25610676	2.12E-19	3.88E-15	0.276	0.077
SMOX	1	0.25559085	2.00E-07	0.00364093	0.299	0.178
RORA	1	0.25426141	3.44E-14	6.27E-10	0.463	0.253
LTBP1	1	0.25204112	1.85E-26	3.38E-22	0.235	0.029
GLRX	1	0.25155189	3.48E-08	0.00063427	0.361	0.221
FTL	1	-0.2500986	2.55E-29	4.65E-25	1	1
MDFI	1	-0.2506436	1.16E-11	2.12E-07	0.491	0.691
HADHA	1	-0.2509986	3.41E-13	6.23E-09	0.356	0.555
PTGFR	1	-0.2520806	9.25E-15	1.69E-10	0.112	0.306
PTPRD-AS1	1	-0.2526743	8.70E-14	1.59E-09	0.269	0.481

GABPB1-AS1	1	-0.2540484	2.39E-15	4.35E-11	0.167	0.381
SLC25A5	1	-0.2544972	9.28E-13	1.69E-08	0.368	0.556
EIF2S3	1	-0.2552101	1.89E-13	3.45E-09	0.507	0.684
SNHG18	1	-0.2555748	4.06E-14	7.40E-10	0.205	0.413
IFI27L2	1	-0.2557548	4.56E-14	8.31E-10	0.801	0.916
MYL12B	1	-0.2559523	2.72E-13	4.96E-09	0.475	0.662
MZT2B	1	-0.256057	3.29E-21	6.01E-17	0.945	0.98
MSMP	1	-0.2608719	7.23E-07	0.01318821	0.123	0.246
KTN1	1	-0.2609597	6.10E-13	1.11E-08	0.562	0.711
RPS4Y1	1	-0.2609727	1.03E-25	1.88E-21	0.977	0.997
CCNG1	1	-0.2613498	3.28E-13	5.99E-09	0.384	0.582
DPYSL3	1	-0.2614197	1.20E-15	2.20E-11	0.155	0.372
BEX2	1	-0.2623703	2.20E-13	4.01E-09	0.247	0.453
FAU	1	-0.2629002	1.12E-44	2.04E-40	1	1
PRKCSH	1	-0.2633338	3.95E-11	7.21E-07	0.349	0.521
QARS	1	-0.2634873	1.41E-15	2.57E-11	0.171	0.382
RPL6	1	-0.2637262	8.57E-37	1.56E-32	1	1
AC005152.3	1	-0.2637956	1.62E-15	2.96E-11	0.1	0.301
RPL28	1	-0.2642941	1.27E-42	2.32E-38	1	1
RPL32	1	-0.2643111	8.98E-50	1.64E-45	1	1
RPL5	1	-0.2654257	3.76E-35	6.85E-31	1	1
PDE4DIP	1	-0.265534	4.04E-13	7.37E-09	0.393	0.581
PGAM1	1	-0.26584	2.87E-15	5.24E-11	0.783	0.905
HMGB1	1	-0.2668041	2.92E-13	5.32E-09	0.642	0.817
ATP5A1	1	-0.2673171	4.82E-13	8.80E-09	0.5	0.671
HERPUD1	1	-0.2695916	2.77E-12	5.06E-08	0.425	0.607
RPL24	1	-0.2715839	7.44E-47	1.36E-42	1	1
MYLK	1	-0.2721412	1.55E-11	2.84E-07	0.194	0.373
ARL2BP	1	-0.272246	2.30E-12	4.19E-08	0.521	0.651
HOXB8	1	-0.2726575	5.13E-20	9.36E-16	0.096	0.33
INSIG1	1	-0.2726904	4.74E-09	8.66E-05	0.345	0.493
SLC29A1	1	-0.2727173	6.21E-15	1.13E-10	0.256	0.472
COL6A1	1	-0.272858	2.02E-14	3.69E-10	0.413	0.621
RARRES3	1	-0.2729094	1.28E-18	2.33E-14	0.096	0.321
UQCRHL	1	-0.2733068	4.15E-15	7.56E-11	0.331	0.544
TIMP2	1	-0.2734909	9.83E-14	1.79E-09	0.539	0.719
RPS4X	1	-0.2743365	9.26E-52	1.69E-47	1	1
BRK1	1	-0.275159	7.87E-21	1.44E-16	0.852	0.946
SNRPE	1	-0.2764654	1.59E-13	2.89E-09	0.584	0.734

HIST1H2AC	1	-0.2766537	2.19E-16	3.99E-12	0.116	0.33
TSPO	1	-0.2769122	5.32E-19	9.71E-15	0.822	0.934
RPA3	1	-0.2778217	5.33E-16	9.72E-12	0.436	0.641
RPL36AL	1	-0.2786368	1.09E-26	1.99E-22	0.995	1
FBXO2	1	-0.278668	2.23E-16	4.07E-12	0.858	0.949
RPL26	1	-0.2793231	1.42E-45	2.58E-41	1	1
CAV1	1	-0.2817383	5.84E-18	1.07E-13	0.568	0.82
LITAF	1	-0.2819207	9.87E-14	1.80E-09	0.459	0.639
EIF3E	1	-0.2822142	4.06E-23	7.40E-19	0.995	1
IGBP1	1	-0.2837925	1.72E-13	3.14E-09	0.532	0.693
C19orf43	1	-0.2855386	3.08E-16	5.62E-12	0.772	0.899
TRPS1	1	-0.2857706	9.75E-13	1.78E-08	0.438	0.625
PRCP	1	-0.2865034	5.45E-17	9.95E-13	0.196	0.424
EIF3M	1	-0.2865603	1.11E-13	2.02E-09	0.495	0.665
HMGN2	1	-0.2865941	2.93E-21	5.35E-17	0.893	0.972
KHDRBS3	1	-0.2866465	1.89E-23	3.45E-19	0.011	0.224
MTDH	1	-0.2870947	4.85E-15	8.85E-11	0.571	0.745
NCOR1	1	-0.2871382	3.38E-18	6.18E-14	0.162	0.395
SSR4	1	-0.2875442	1.12E-18	2.04E-14	0.973	0.992
SMIM19	1	-0.2879314	1.14E-15	2.08E-11	0.336	0.544
PPT1	1	-0.2888066	4.41E-14	8.05E-10	0.416	0.604
CYBRD1	1	-0.2888883	8.03E-16	1.47E-11	0.459	0.671
CA11	1	-0.2900238	2.40E-20	4.37E-16	0.158	0.413
SERTAD4-AS1	1	-0.2908141	4.00E-18	7.30E-14	0.123	0.349
CTSK	1	-0.2934951	1.01E-14	1.84E-10	0.288	0.493
FBL	1	-0.2935572	3.91E-16	7.14E-12	0.459	0.667
NGFRAP1	1	-0.293803	2.81E-21	5.12E-17	0.909	0.983
RPL15	1	-0.294526	1.34E-66	2.44E-62	1	1
ARPC1A	1	-0.2947709	1.39E-16	2.53E-12	0.425	0.651
SP5	1	-0.2957142	9.95E-23	1.82E-18	0	0.195
KDELR2	1	-0.2983727	2.39E-19	4.36E-15	0.769	0.939
NACA	1	-0.2986633	3.93E-44	7.18E-40	1	1
RPL7A	1	-0.2990311	6.92E-51	1.26E-46	1	1
LPP	1	-0.2996654	1.11E-08	0.00020325	0.381	0.525
COX7C	1	-0.3005037	5.13E-37	9.36E-33	1	1
RPS14	1	-0.3026946	1.90E-68	3.46E-64	1	1
EIF3F	1	-0.3028063	6.34E-20	1.16E-15	0.747	0.892
LTA4H	1	-0.305502	1.33E-15	2.42E-11	0.409	0.607
ITM2C	1	-0.3074731	3.35E-15	6.12E-11	0.694	0.853

ATP5D	1	-0.3084345	1.99E-24	3.63E-20	0.826	0.955
MIA	1	-0.3084484	6.68E-18	1.22E-13	0.998	0.997
ST13	1	-0.3092858	4.10E-25	7.48E-21	0.927	0.992
CCDC8	1	-0.3102243	6.01E-21	1.10E-16	0.098	0.339
GNB2L1	1	-0.3105073	9.47E-43	1.73E-38	0.998	1
PPIC	1	-0.3122461	1.25E-11	2.28E-07	0.45	0.622
PEBP1	1	-0.3124466	4.47E-24	8.16E-20	0.808	0.975
RPS28	1	-0.3125015	1.01E-69	1.84E-65	1	1
GLTSCR2	1	-0.3158579	1.25E-22	2.29E-18	0.886	0.965
OAF	1	-0.3161621	3.58E-18	6.53E-14	0.155	0.384
PLP2	1	-0.3165639	4.50E-20	8.22E-16	0.644	0.842
RPS19	1	-0.3171075	2.03E-60	3.71E-56	1	1
NUCB2	1	-0.3173223	7.80E-12	1.42E-07	0.516	0.644
HSP90AB1	1	-0.3173721	9.27E-20	1.69E-15	0.954	0.994
C12orf57	1	-0.3175389	2.05E-26	3.74E-22	0.952	0.988
DBP	1	-0.3178758	1.32E-14	2.41E-10	0.267	0.478
SNAI2	1	-0.3185702	2.83E-14	5.17E-10	0.409	0.634
RPS15A	1	-0.3188916	3.93E-59	7.16E-55	1	1
MT-ND5	1	-0.3191021	4.48E-10	8.18E-06	0.587	0.697
PLAC9	1	-0.3193139	3.19E-15	5.82E-11	0.886	0.983
PLXDC2	1	-0.3201463	6.57E-17	1.20E-12	0.555	0.747
NINJ1	1	-0.3205262	1.62E-16	2.95E-12	0.511	0.716
COL6A2	1	-0.3209212	1.62E-13	2.96E-09	0.632	0.808
ILF3-AS1	1	-0.3215491	5.42E-20	9.89E-16	0.265	0.519
NMRK1	1	-0.3248303	1.74E-19	3.18E-15	0.256	0.499
LRRC2	1	-0.3256216	3.27E-18	5.98E-14	0.148	0.378
CXXC5	1	-0.3263981	1.94E-18	3.54E-14	0.244	0.482
RPS25	1	-0.3276736	6.00E-64	1.09E-59	1	1
C2orf82	1	-0.3282748	2.22E-16	4.05E-12	0.984	1
RPS12	1	-0.3289567	4.20E-58	7.67E-54	1	1
ZIC1	1	-0.3295898	4.12E-16	7.51E-12	0.199	0.424
RP11-71N10.1	1	-0.3299173	3.78E-26	6.89E-22	0.071	0.346
DDR2	1	-0.3322922	2.01E-14	3.66E-10	0.559	0.711
ZNF90	1	-0.3323463	6.61E-22	1.21E-17	0.406	0.661
ZFAS1	1	-0.3324435	2.91E-26	5.32E-22	0.982	0.998
ID2	1	-0.3343434	4.52E-10	8.25E-06	0.42	0.594
AP2M1	1	-0.3350441	3.41E-18	6.22E-14	0.466	0.662
RPS6	1	-0.3368482	1.66E-76	3.04E-72	1	1
CRIP2	1	-0.3375428	1.47E-16	2.69E-12	0.288	0.496

CYSTM1	1	-0.3384014	1.61E-20	2.94E-16	0.418	0.67
RPS24	1	-0.3402734	1.40E-51	2.55E-47	1	1
NDUFA11	1	-0.3406391	3.02E-27	5.52E-23	0.836	0.962
RPL27A	1	-0.3424492	9.68E-64	1.77E-59	1	1
AC003090.1	1	-0.3425446	3.93E-25	7.17E-21	0.11	0.389
SPTLC3	1	-0.3432543	2.81E-14	5.14E-10	0.164	0.369
AFF3	1	-0.3441417	9.63E-31	1.76E-26	0.073	0.382
MALAT1	1	-0.3441891	2.83E-22	5.16E-18	1	1
RPS9	1	-0.3455566	2.18E-70	3.97E-66	1	1
PNISR	1	-0.3459756	5.26E-22	9.60E-18	0.644	0.843
HOXB6	1	-0.3463492	1.65E-21	3.02E-17	0.199	0.461
RGS10	1	-0.3478088	3.52E-22	6.42E-18	0.347	0.616
ERGIC3	1	-0.3492922	4.69E-26	8.56E-22	0.815	0.945
ID3	1	-0.3496134	2.05E-15	3.74E-11	0.422	0.687
PSMA7	1	-0.3512942	3.26E-25	5.94E-21	0.785	0.942
EIF3K	1	-0.3519011	4.97E-28	9.08E-24	0.774	0.919
COL9A3	1	-0.3545085	3.61E-15	6.58E-11	0.724	0.88
PLA2G16	1	-0.354821	7.59E-20	1.39E-15	0.301	0.551
RPS3	1	-0.35638	3.18E-74	5.80E-70	1	1
RPS21	1	-0.3570557	2.70E-56	4.93E-52	1	1
SPINT2	1	-0.3574004	1.84E-16	3.36E-12	0.4	0.613
ATP5G2	1	-0.3584232	2.02E-40	3.68E-36	0.998	1
ITM2A	1	-0.360001	3.94E-19	7.20E-15	0.621	0.833
MPP6	1	-0.3661009	1.84E-26	3.36E-22	0.185	0.485
NPM1	1	-0.3662256	4.38E-35	7.99E-31	1	1
TXNIP	1	-0.3664141	1.06E-28	1.93E-24	0.068	0.359
ATP5L	1	-0.3664574	7.67E-33	1.40E-28	0.904	0.974
GYPC	1	-0.3684788	6.92E-22	1.26E-17	0.306	0.576
RPL35	1	-0.3703837	5.69E-72	1.04E-67	1	1
RPL3	1	-0.3704113	1.20E-71	2.19E-67	1	1
COMMD6	1	-0.370414	8.43E-46	1.54E-41	0.982	0.998
SLC25A3	1	-0.3705866	8.54E-32	1.56E-27	0.838	0.96
RPL37A	1	-0.371289	8.93E-83	1.63E-78	1	1
EID1	1	-0.3715217	1.70E-36	3.10E-32	0.92	0.989
PLPP1	1	-0.3732572	1.09E-18	1.99E-14	0.178	0.415
PRUNE2	1	-0.3735036	7.62E-19	1.39E-14	0.231	0.482
EIF3D	1	-0.3736658	1.22E-23	2.22E-19	0.646	0.833
RPL22	1	-0.3746473	4.37E-63	7.97E-59	1	1
RABAC1	1	-0.3754443	1.33E-26	2.43E-22	0.756	0.912

CP	1	-0.3754653	4.44E-28	8.10E-24	0.721	0.955
OXA1L	1	-0.3768992	1.52E-24	2.77E-20	0.427	0.676
RPL11	1	-0.3786444	4.93E-87	9.00E-83	1	1
RPL13	1	-0.3789087	9.87E-91	1.80E-86	1	1
LDHB	1	-0.3790945	2.19E-27	4.00E-23	0.737	0.906
PPIA	1	-0.3829558	1.90E-36	3.48E-32	0.92	0.991
NKX3-2	1	-0.383692	5.45E-23	9.94E-19	0.073	0.321
MEG3	1	-0.3866219	3.48E-19	6.35E-15	0.196	0.441
RPS27	1	-0.3886086	6.40E-85	1.17E-80	1	1
FHL1	1	-0.3910261	3.46E-17	6.31E-13	0.336	0.544
RPL39	1	-0.3917889	6.67E-69	1.22E-64	1	1
ATP5O	1	-0.3919124	8.62E-31	1.57E-26	0.705	0.892
RPL27	1	-0.3934147	6.38E-57	1.16E-52	0.998	1
RPS7	1	-0.3977647	9.69E-73	1.77E-68	1	1
RPL19	1	-0.3983751	8.45E-96	1.54E-91	1	1
HLA-A	1	-0.3987521	4.24E-26	7.74E-22	0.918	0.989
TUBB2B	1	-0.4027244	1.81E-16	3.31E-12	0.418	0.61
HS3ST1	1	-0.402963	3.61E-28	6.59E-24	0.068	0.352
RPS23	1	-0.4032725	1.51E-87	2.76E-83	1	1
RPL18A	1	-0.4037754	3.01E-90	5.48E-86	1	1
NFIA	1	-0.4045911	2.22E-26	4.06E-22	0.272	0.565
RPL35A	1	-0.406725	1.71E-82	3.12E-78	1	1
ZBTB20	1	-0.4098291	1.94E-28	3.53E-24	0.292	0.601
TTC3	1	-0.4113188	1.04E-29	1.90E-25	0.671	0.882
RPSA	1	-0.4132118	2.56E-57	4.68E-53	1	1
RPL21	1	-0.415225	2.84E-102	5.18E-98	1	1
RPLP1	1	-0.4154731	2.70E-95	4.92E-91	1	1
RPS5	1	-0.415819	1.20E-83	2.19E-79	1	1
YBX1	1	-0.4166191	3.79E-48	6.92E-44	0.966	0.997
UBA52	1	-0.4178585	1.54E-85	2.81E-81	1	1
MYL6	1	-0.4180643	3.29E-35	6.00E-31	0.884	0.971
BTF3	1	-0.4196651	3.57E-54	6.52E-50	0.989	1
RPL10	1	-0.4213924	2.46E-98	4.48E-94	1	1
TOMM7	1	-0.4215192	1.00E-81	1.83E-77	1	1
RPS2	1	-0.4223925	4.34E-97	7.92E-93	1	1
NNMT	1	-0.4253348	4.23E-25	7.71E-21	0.877	0.974
HNRNPA1	1	-0.4258966	1.29E-47	2.36E-43	0.986	1
PTMS	1	-0.4261006	9.97E-28	1.82E-23	0.568	0.826
LECT1	1	-0.4298508	6.33E-26	1.15E-21	0.95	0.988

RPL29	1	-0.4319082	5.89E-85	1.07E-80	1	1
EIF2A	1	-0.4351889	8.23E-33	1.50E-28	0.628	0.863
RPL9	1	-0.4357456	1.79E-94	3.27E-90	1	1
HINT1	1	-0.4358368	8.41E-49	1.53E-44	0.963	0.994
PDIA6	1	-0.4367973	3.27E-27	5.97E-23	0.726	0.9
RPS18	1	-0.4368328	4.09E-97	7.46E-93	1	1
HDLBP	1	-0.4383966	5.50E-29	1.00E-24	0.395	0.684
RPL8	1	-0.4394533	1.41E-98	2.57E-94	1	1
RPL14	1	-0.4408129	1.34E-86	2.44E-82	1	1
RPL31	1	-0.4433892	1.21E-86	2.21E-82	1	1
ATRNL1	1	-0.4447169	6.88E-33	1.25E-28	0.158	0.499
GAS6	1	-0.44537	4.45E-27	8.12E-23	0.441	0.699
CHRDL2	1	-0.4458083	2.46E-19	4.49E-15	0.144	0.389
NAP1L1	1	-0.4504471	1.96E-47	3.57E-43	0.922	0.989
UQCRH	1	-0.45092	3.70E-55	6.76E-51	0.895	0.992
HLA-C	1	-0.4535923	1.71E-34	3.13E-30	0.852	0.974
HIST1H4C	1	-0.4566251	6.22E-27	1.14E-22	0.372	0.662
ENPP1	1	-0.4585772	1.58E-33	2.89E-29	0.203	0.555
PCBP2	1	-0.4629226	2.28E-47	4.17E-43	0.881	0.982
GNAS	1	-0.4634043	1.24E-34	2.26E-30	0.566	0.817
PABPC1	1	-0.4659922	4.38E-44	7.99E-40	0.854	0.974
HOXC10	1	-0.4667046	1.75E-33	3.20E-29	0.591	0.84
TPM1	1	-0.4709793	9.82E-24	1.79E-19	0.381	0.645
CYR61	1	-0.4717019	2.45E-21	4.48E-17	0.311	0.613
RPL37	1	-0.4751083	3.90E-100	7.11E-96	1	1
RPS27A	1	-0.4766624	2.05E-120	3.75E-116	1	1
VIM	1	-0.479089	5.53E-43	1.01E-38	1	1
RPS13	1	-0.4821495	2.98E-99	5.44E-95	1	1
RPL13A	1	-0.483419	5.46E-122	9.96E-118	1	1
HLA-B	1	-0.4928451	3.81E-33	6.96E-29	0.767	0.934
MAGED1	1	-0.5015916	3.89E-11	7.11E-07	0.242	0.421
H3F3A	1	-0.5017351	9.09E-59	1.66E-54	0.934	0.998
GPX3	1	-0.5050638	9.45E-39	1.72E-34	0.959	0.997
SEC11A	1	-0.5051824	5.84E-50	1.07E-45	0.747	0.939
SSPN	1	-0.5068556	1.67E-37	3.04E-33	0.233	0.608
TUBB	1	-0.5096702	5.19E-40	9.46E-36	0.646	0.888
ARHGAP29	1	-0.5113703	2.00E-29	3.65E-25	0.352	0.645
CCDC102B	1	-0.5151531	5.84E-46	1.07E-41	0.037	0.425
PTRF	1	-0.5155056	1.04E-37	1.90E-33	0.345	0.691

UQCRB	1	-0.5166824	2.30E-63	4.20E-59	0.963	1
EEF1B2	1	-0.5192653	9.63E-66	1.76E-61	0.977	0.997
RPL23A	1	-0.5257577	1.13E-123	2.06E-119	1	1
SLC44A1	1	-0.5337512	1.89E-38	3.45E-34	0.178	0.535
RPS29	1	-0.5342767	3.86E-112	7.05E-108	1	1
CTGF	1	-0.5348426	1.54E-19	2.80E-15	0.358	0.627
MOXD1	1	-0.5356866	2.79E-41	5.10E-37	0.135	0.519
RPL30	1	-0.536289	7.69E-117	1.40E-112	1	1
SSR3	1	-0.5396284	1.61E-47	2.94E-43	0.858	0.966
MT-ND4	1	-0.5406167	2.25E-31	4.10E-27	0.963	0.966
SSR2	1	-0.5463849	2.86E-54	5.22E-50	0.733	0.954
RPL34	1	-0.5578183	1.22E-122	2.23E-118	1	1
NPC2	1	-0.5578434	5.30E-71	9.67E-67	0.966	0.998
EIF3L	1	-0.5592207	8.36E-59	1.53E-54	0.795	0.977
RPS3A	1	-0.5604116	1.45E-127	2.64E-123	1	1
RPL23	1	-0.5652015	1.01E-95	1.84E-91	0.995	1
EIF4B	1	-0.5658737	5.65E-51	1.03E-46	0.765	0.951
CD9	1	-0.5673619	2.64E-39	4.82E-35	0.409	0.75
TPT1	1	-0.5708823	2.14E-108	3.90E-104	1	1
RPS17	1	-0.5743028	3.30E-112	6.03E-108	1	1
RPLP2	1	-0.5753994	2.46E-127	4.48E-123	1	1
CITED2	1	-0.5792091	1.87E-33	3.42E-29	0.301	0.651
RHOBTB3	1	-0.5873571	4.31E-46	7.87E-42	0.553	0.865
RPL7	1	-0.6000961	1.88E-110	3.43E-106	1	1
RPL4	1	-0.6037221	2.27E-102	4.15E-98	1	1
RPS16	1	-0.6068979	5.71E-126	1.04E-121	1	1
RPL18	1	-0.6085816	2.98E-126	5.44E-122	1	1
RPL10A	1	-0.6169387	1.43E-111	2.61E-107	1	1
SERPINA5	1	-0.6201736	2.71E-43	4.94E-39	0.612	0.854
EEF1A1	1	-0.6307747	5.21E-119	9.51E-115	1	1
P4HB	1	-0.6309404	1.96E-54	3.57E-50	0.813	0.978
YBX3	1	-0.6333882	9.17E-65	1.67E-60	0.719	0.971
THY1	1	-0.6367243	8.02E-36	1.46E-31	0.308	0.648
ACTG1	1	-0.6466559	1.64E-50	2.99E-46	0.888	0.975
TCF4	1	-0.6480408	1.14E-44	2.07E-40	0.368	0.728
EEF2	1	-0.6641939	8.33E-68	1.52E-63	0.975	1
NEAT1	1	-0.6718664	7.44E-64	1.36E-59	0.986	1
RPS8	1	-0.6738944	7.11E-136	1.30E-131	1	1
MT-CO2	1	-0.6967343	1.99E-42	3.64E-38	0.945	0.972

SLC14A1	1	-0.6976955	7.53E-53	1.37E-48	0.326	0.737
HIST1H1C	1	-0.711753	8.18E-33	1.49E-28	0.189	0.528
TSC22D1	1	-0.7184128	3.30E-35	6.02E-31	0.779	0.935
CCNI	1	-0.7257413	6.27E-104	1.14E-99	0.986	1
MT-ND1	1	-0.7299649	4.89E-45	8.92E-41	0.584	0.851
RPLP0	1	-0.7761294	5.96E-141	1.09E-136	1	1
B2M	1	-0.7907609	1.73E-103	3.16E-99	0.993	1
PAX1	1	-0.8065734	1.81E-72	3.31E-68	0.594	0.949
MT-CO1	1	-0.8153655	1.26E-51	2.30E-47	0.947	0.969
MT-ATP6	1	-0.8280482	4.89E-51	8.93E-47	0.756	0.922
CRTAP	1	-0.8339431	3.83E-100	6.99E-96	0.523	0.946
ISLR	1	-0.8436491	1.13E-73	2.06E-69	0.416	0.862
MT-CO3	1	-0.8680193	1.57E-56	2.87E-52	0.84	0.948
XAGE2	1	-0.8701211	3.39E-26	6.19E-22	0.016	0.253
FTH1	1	-0.883484	4.34E-127	7.91E-123	1	1
MT-ND2	1	-0.8907815	2.13E-48	3.88E-44	0.603	0.857
CAPS	1	-0.8934464	1.99E-67	3.63E-63	0.733	0.972
MT-CYB	1	-0.904296	1.54E-57	2.81E-53	0.619	0.905
EIF3H	1	-0.9081249	5.91E-121	1.08E-116	0.945	1
COL2A1	1	-0.9371047	4.83E-46	8.81E-42	0.717	0.969
SLC40A1	1	-0.983289	5.04E-72	9.19E-68	0.16	0.688
RPS20	1	-1.0073216	3.92E-160	7.16E-156	0.995	1
SPARC	1	-1.0304494	4.10E-68	7.48E-64	0.53	0.902
RPS11	1	-1.0316865	1.21E-157	2.21E-153	0.982	1
ZFP36L2	1	-1.049991	1.98E-64	3.62E-60	0.169	0.664
MT-ND3	1	-1.0642473	2.42E-74	4.41E-70	0.587	0.897
CRISPLD1	1	-1.0760114	1.50E-53	2.73E-49	0.146	0.588
ADIRF	1	-1.0843082	1.28E-73	2.33E-69	0.381	0.856
OGN	1	-1.089475	2.33E-94	4.25E-90	0.938	1
APOD	1	-1.1182894	2.74E-64	5.01E-60	0.226	0.722
CLEC3A	1	-1.2390386	6.53E-80	1.19E-75	0.587	0.949
SPTSSB	1	-1.2941568	1.69E-82	3.09E-78	0.043	0.641
FRZB	1	-1.5319439	2.32E-74	4.23E-70	0.26	0.794
**gene**	**cluster**	**avg_logFC**	**p_val**	**p_val_adj**	**pct.1**	**pct.2**
SPP1	2	1.44110631	1.93E-58	3.53E-54	0.638	0.068
PRG4	2	1.3586778	9.44E-45	1.72E-40	0.477	0.031
CHI3L2	2	1.32984446	4.87E-69	8.88E-65	0.928	0.452
PLA2G2A	2	1.31123869	7.41E-37	1.35E-32	0.749	0.299
S100A2	2	1.14148248	1.36E-27	2.48E-23	0.59	0.254

VEGFA	2	1.12180214	4.16E-63	7.59E-59	0.828	0.35
PTGS2	2	1.05995462	5.22E-64	9.53E-60	0.629	0.042
COMP	2	0.965253	3.71E-38	6.77E-34	0.941	0.74
DDIT4L	2	0.95242239	1.04E-44	1.90E-40	0.844	0.435
G0S2	2	0.94252857	3.90E-09	7.11E-05	0.376	0.203
CCDC85B	2	0.93270897	4.00E-41	7.30E-37	0.914	0.701
PPP3CA	2	0.87880683	4.91E-47	8.96E-43	0.934	0.644
CTSL	2	0.85131129	3.47E-19	6.34E-15	0.622	0.393
BMP2	2	0.84873235	6.56E-37	1.20E-32	0.448	0.051
UPP1	2	0.8406463	3.43E-34	6.26E-30	0.468	0.085
MRPS6	2	0.83255811	7.88E-27	1.44E-22	0.957	0.879
TGFBI	2	0.83118944	1.98E-43	3.61E-39	0.64	0.184
MT2A	2	0.81265381	7.76E-41	1.42E-36	1	1
SOD2	2	0.79644767	2.45E-34	4.46E-30	0.993	0.907
MT1G	2	0.77783518	3.43E-40	6.26E-36	1	0.997
WTAP	2	0.77318099	3.62E-31	6.60E-27	0.812	0.554
MT1X	2	0.76063181	3.01E-48	5.49E-44	1	1
PHLDA1	2	0.74319627	4.63E-32	8.45E-28	0.394	0.04
FN1	2	0.72857922	7.20E-57	1.31E-52	1	0.994
IGFBP3	2	0.71614489	9.17E-24	1.67E-19	0.355	0.065
SLC25A37	2	0.70111707	5.73E-40	1.05E-35	0.912	0.588
ERO1A	2	0.68406959	3.34E-30	6.10E-26	0.661	0.339
BTG1	2	0.68296818	1.73E-36	3.16E-32	0.982	0.912
ADM	2	0.66680984	5.33E-21	9.72E-17	0.803	0.54
HILPDA	2	0.66581907	4.86E-28	8.87E-24	0.643	0.291
CTSD	2	0.66460333	9.11E-17	1.66E-12	0.991	0.935
TNFRSF12A	2	0.6546399	3.42E-19	6.24E-15	0.4	0.133
FOSL1	2	0.64746911	5.03E-28	9.18E-24	0.335	0.025
BHLHE40	2	0.63900629	6.83E-22	1.25E-17	0.575	0.291
ENO2	2	0.61278473	2.44E-33	4.45E-29	0.654	0.28
PTP4A1	2	0.61226461	1.36E-19	2.49E-15	0.586	0.35
PMP22	2	0.60694649	1.57E-26	2.86E-22	0.796	0.537
DDIT3	2	0.6029596	1.83E-27	3.35E-23	0.645	0.308
TM4SF1	2	0.59528264	1.21E-07	0.00221453	0.443	0.305
ORM1	2	0.59027687	1.48E-08	0.00026933	0.371	0.203
MT1H	2	0.58141667	6.07E-24	1.11E-19	0.984	0.929
ARF6	2	0.57968512	5.00E-26	9.12E-22	0.661	0.373
BZW1	2	0.56962911	1.18E-26	2.15E-22	0.692	0.393
STK17A	2	0.56798404	8.12E-17	1.48E-12	0.396	0.158

SERPINE2	2	0.55799368	4.40E-25	8.02E-21	0.998	0.986
CD44	2	0.54799727	8.03E-37	1.47E-32	0.916	0.636
CTHRC1	2	0.54685992	1.40E-33	2.56E-29	0.423	0.051
IGFBP5	2	0.53688559	2.28E-17	4.16E-13	0.344	0.096
NDRG1	2	0.52359379	2.96E-20	5.40E-16	0.645	0.398
TREM1	2	0.51912149	5.12E-35	9.34E-31	0.362	0.006
PPP1CB	2	0.50689997	1.28E-14	2.33E-10	0.826	0.701
CA9	2	0.50320109	5.30E-20	9.66E-16	0.543	0.271
SLC2A1	2	0.50212596	2.00E-20	3.64E-16	0.428	0.15
FAM162A	2	0.50185738	6.04E-27	1.10E-22	0.959	0.842
EGLN3	2	0.49959373	1.25E-20	2.29E-16	0.475	0.189
AK2	2	0.49643471	2.40E-14	4.38E-10	0.697	0.585
EGR1	2	0.49118765	8.33E-10	1.52E-05	0.416	0.243
EPYC	2	0.47803873	1.88E-14	3.43E-10	0.351	0.124
HLA-DRB1	2	0.47113421	3.99E-18	7.28E-14	0.455	0.192
NRP2	2	0.46587104	6.68E-24	1.22E-19	0.369	0.071
SLC3A2	2	0.46308588	1.74E-14	3.18E-10	0.491	0.254
C11orf96	2	0.46014509	1.57E-16	2.87E-12	0.231	0.028
NAMPT	2	0.45328498	1.46E-20	2.66E-16	0.633	0.37
WDR45B	2	0.44373524	1.80E-19	3.29E-15	0.498	0.215
ARRDC3	2	0.43762222	1.63E-09	2.97E-05	0.473	0.316
IFRD1	2	0.43607444	1.43E-17	2.61E-13	0.629	0.41
RNF145	2	0.43035721	4.78E-17	8.73E-13	0.511	0.254
PGK1	2	0.42792721	1.00E-16	1.83E-12	0.966	0.904
CLK1	2	0.42071935	1.54E-19	2.81E-15	0.654	0.404
NRN1	2	0.41793972	1.22E-06	0.02224207	0.5	0.331
PPP1R15A	2	0.41687135	8.73E-20	1.59E-15	0.446	0.158
CRTAC1	2	0.40869196	1.90E-19	3.47E-15	0.425	0.144
ERRFI1	2	0.40659569	2.61E-13	4.76E-09	0.373	0.153
TMEM45A	2	0.40542996	2.58E-16	4.71E-12	0.885	0.782
ISG20	2	0.40179454	6.72E-16	1.23E-11	0.568	0.333
ATP6V1G1	2	0.40160815	1.61E-25	2.95E-21	0.986	0.927
ZNF385D	2	0.39516815	5.50E-16	1.00E-11	0.717	0.523
PDE4B	2	0.39247943	1.12E-21	2.04E-17	0.369	0.082
SPRY2	2	0.39244194	2.30E-22	4.20E-18	0.317	0.045
NT5C3A	2	0.38924705	6.15E-19	1.12E-14	0.398	0.124
SGIP1	2	0.38763249	7.25E-22	1.32E-17	0.274	0.023
GPX1	2	0.38727755	2.57E-19	4.70E-15	0.928	0.768
SLC5A3	2	0.38580775	5.14E-08	0.00093713	0.543	0.41

VAPA	2	0.38353289	1.59E-19	2.90E-15	0.934	0.845
RABGGTB	2	0.38016243	9.12E-19	1.66E-14	0.756	0.537
SLC39A8	2	0.37677697	2.84E-14	5.18E-10	0.493	0.266
ARPP19	2	0.37463936	4.24E-15	7.73E-11	0.62	0.401
MMP3	2	0.37384492	4.82E-09	8.80E-05	0.921	0.797
CFD	2	0.37349574	2.97E-14	5.42E-10	0.299	0.082
GAPDH	2	0.37160953	1.52E-10	2.77E-06	1	1
GBE1	2	0.3697097	8.26E-10	1.51E-05	0.627	0.525
EIF4EBP1	2	0.36133482	1.76E-09	3.21E-05	0.561	0.39
TMSB10	2	0.35993266	4.29E-13	7.83E-09	0.993	0.977
HMGA1	2	0.35487441	4.77E-07	0.00871057	0.353	0.218
COX17	2	0.35439853	1.02E-13	1.87E-09	0.819	0.706
RNMT	2	0.35296047	6.25E-13	1.14E-08	0.509	0.299
SMOX	2	0.34726473	1.45E-09	2.64E-05	0.321	0.15
PPFIBP1	2	0.34649177	4.15E-09	7.57E-05	0.509	0.35
MT1M	2	0.34148255	5.34E-09	9.74E-05	0.808	0.675
XBP1	2	0.33842824	7.79E-10	1.42E-05	0.665	0.534
PTGES3	2	0.33815339	2.47E-19	4.50E-15	0.962	0.898
SLC25A36	2	0.33734162	1.28E-11	2.33E-07	0.577	0.41
SNHG12	2	0.3334791	8.42E-11	1.54E-06	0.466	0.28
CD55	2	0.33233335	6.17E-11	1.13E-06	0.611	0.421
DNAJB9	2	0.32916395	3.18E-09	5.81E-05	0.649	0.497
CLEC2B	2	0.32899444	6.22E-07	0.01134522	0.219	0.093
CDK5RAP2	2	0.3288973	6.94E-09	0.00012669	0.344	0.175
PLA2G4A	2	0.32775052	8.01E-16	1.46E-11	0.385	0.141
GPRC5A	2	0.32643784	1.54E-17	2.80E-13	0.337	0.09
SELK	2	0.32576836	6.52E-16	1.19E-11	0.885	0.774
ZNF593	2	0.3251803	9.96E-14	1.82E-09	0.527	0.291
EMP3	2	0.32246462	8.88E-12	1.62E-07	0.896	0.831
MIF	2	0.31568041	2.68E-22	4.90E-18	1	1
ARF4	2	0.31516535	7.99E-10	1.46E-05	0.706	0.576
ADAMTS6	2	0.3128732	6.23E-10	1.14E-05	0.244	0.085
CTNNB1	2	0.31200096	1.50E-07	0.00274355	0.441	0.282
RAB1A	2	0.31106635	5.75E-11	1.05E-06	0.769	0.633
C4orf48	2	0.30784437	3.81E-11	6.95E-07	0.419	0.212
ANKRD37	2	0.30768114	1.78E-11	3.24E-07	0.407	0.203
MED21	2	0.30675597	3.78E-14	6.89E-10	0.523	0.282
GOLT1B	2	0.30458171	2.24E-10	4.08E-06	0.43	0.246
KLF3	2	0.30418682	1.14E-09	2.07E-05	0.353	0.175

BNIP3	2	0.30131803	3.37E-09	6.15E-05	0.998	0.997
SLC20A1	2	0.29920236	7.46E-15	1.36E-10	0.287	0.073
RELB	2	0.29833892	5.34E-15	9.74E-11	0.333	0.102
CCNG2	2	0.29696646	7.84E-08	0.00143124	0.484	0.336
RAB13	2	0.29693178	1.92E-11	3.50E-07	0.889	0.836
TRMT112	2	0.28932932	1.06E-12	1.93E-08	0.921	0.859
CCND1	2	0.28881265	2.18E-16	3.98E-12	0.274	0.051
WSB1	2	0.28798071	6.79E-11	1.24E-06	0.837	0.729
BAALC	2	0.28773681	9.25E-08	0.00168694	0.523	0.359
A4GALT	2	0.28550139	2.78E-18	5.07E-14	0.244	0.025
MGST1	2	0.28523483	3.18E-11	5.80E-07	0.378	0.167
TMEM38B	2	0.28423075	9.21E-10	1.68E-05	0.527	0.347
SERPINE1	2	0.28331814	2.37E-16	4.33E-12	0.231	0.028
CLU	2	0.28197171	8.80E-08	0.00160657	1	1
PLAUR	2	0.28189221	4.59E-10	8.37E-06	0.369	0.181
NTMT1	2	0.27981096	9.15E-10	1.67E-05	0.482	0.308
ZFYVE16	2	0.27946679	1.98E-16	3.61E-12	0.326	0.088
CLDND1	2	0.2783687	9.08E-12	1.66E-07	0.473	0.257
SMIM3	2	0.27607363	6.69E-13	1.22E-08	0.31	0.105
ATP6V0E1	2	0.27589735	4.96E-11	9.05E-07	0.93	0.836
TPD52	2	0.27268327	1.44E-08	0.00026343	0.676	0.556
EIF1B	2	0.27244004	1.30E-11	2.38E-07	0.74	0.545
NBPF14	2	0.2723623	1.23E-17	2.24E-13	0.31	0.068
DNER	2	0.27126835	7.93E-08	0.00144618	0.518	0.373
MTHFD2	2	0.27002528	3.65E-07	0.0066684	0.6	0.46
AVPI1	2	0.26995719	5.75E-10	1.05E-05	0.385	0.195
IER3	2	0.26776955	7.19E-07	0.01311843	0.274	0.138
EPS8	2	0.26594884	1.14E-11	2.07E-07	0.321	0.124
HMGA2	2	0.26479778	3.95E-19	7.22E-15	0.201	0
SNRNP27	2	0.2636541	6.33E-10	1.15E-05	0.48	0.288
EVI2A	2	0.25544975	1.06E-13	1.94E-09	0.285	0.079
CSGALNACT2	2	0.25517034	9.81E-09	0.00017894	0.281	0.124
NOL7	2	0.25362902	1.21E-07	0.00221547	0.627	0.5
FAM210A	2	0.25353731	4.28E-10	7.81E-06	0.376	0.186
RNF126	2	0.25353731	1.49E-08	0.00027162	0.348	0.184
PRR34-AS1	2	0.25052552	2.11E-13	3.84E-09	0.314	0.102
OAF	2	-0.2502901	3.45E-12	6.30E-08	0.145	0.35
TBL1XR1	2	-0.2504341	1.69E-10	3.08E-06	0.206	0.401
KHDRBS3	2	-0.2504924	6.66E-20	1.22E-15	0.016	0.215

CMB9-22P13.1	2	-0.2515136	1.04E-07	0.00189258	0.387	0.542
PRDX5	2	-0.2525598	8.61E-14	1.57E-09	0.867	0.949
COL9A2	2	-0.2527396	1.58E-07	0.00289144	0.303	0.475
MKX	2	-0.2532943	1.51E-11	2.76E-07	0.17	0.373
RPL26	2	-0.2533017	5.18E-31	9.45E-27	1	1
GNB2L1	2	-0.2537016	5.25E-23	9.58E-19	1	1
KDELR2	2	-0.2538536	2.79E-10	5.10E-06	0.769	0.918
PSAP	2	-0.255395	5.33E-12	9.72E-08	0.923	0.975
PARK7	2	-0.2566451	4.91E-12	8.96E-08	0.808	0.927
ARL6IP5	2	-0.2568597	3.86E-09	7.04E-05	0.507	0.684
NME2	2	-0.2576306	5.90E-14	1.08E-09	0.176	0.404
ANGPTL5	2	-0.258118	4.65E-12	8.49E-08	0.176	0.387
ZFAS1	2	-0.2582488	8.63E-14	1.58E-09	0.998	0.997
DSEL	2	-0.2589024	1.59E-12	2.89E-08	0.072	0.249
IDH2	2	-0.2590542	2.26E-17	4.13E-13	0.077	0.305
ZCRB1	2	-0.2592666	9.59E-11	1.75E-06	0.396	0.602
PRKCSH	2	-0.2601286	4.10E-13	7.48E-09	0.267	0.503
PTPRD-AS1	2	-0.2602952	2.34E-10	4.28E-06	0.31	0.508
RPS28	2	-0.2608206	3.52E-38	6.42E-34	1	1
SDC2	2	-0.2612612	1.05E-11	1.91E-07	0.787	0.927
GDI2	2	-0.2614718	7.14E-09	0.00013031	0.611	0.751
H1FX	2	-0.2618733	8.50E-11	1.55E-06	0.179	0.367
ISM1	2	-0.2620183	4.80E-14	8.75E-10	0.09	0.294
DMKN	2	-0.2626436	2.12E-14	3.86E-10	0.29	0.556
RPS14	2	-0.263762	5.19E-42	9.47E-38	1	1
HOXB6	2	-0.2638309	4.06E-11	7.40E-07	0.233	0.435
RPS15A	2	-0.2638424	1.69E-39	3.08E-35	1	1
PRCP	2	-0.2639239	1.61E-13	2.95E-09	0.138	0.353
SET	2	-0.2642285	2.22E-09	4.06E-05	0.656	0.816
ARPC1B	2	-0.2643646	9.10E-12	1.66E-07	0.262	0.477
RPS19	2	-0.2647972	6.70E-38	1.22E-33	1	1
DPYSL3	2	-0.2659551	4.51E-14	8.23E-10	0.14	0.362
RCN3	2	-0.266538	6.56E-12	1.20E-07	0.242	0.463
NUCKS1	2	-0.2676309	7.60E-13	1.39E-08	0.538	0.74
ZNF90	2	-0.2688273	7.75E-11	1.41E-06	0.5	0.661
IVNS1ABP	2	-0.2693724	1.44E-07	0.00263476	0.719	0.822
RP11-553L6.5	2	-0.2696831	3.83E-14	7.00E-10	0.21	0.446
C6orf48	2	-0.270224	1.03E-10	1.87E-06	0.765	0.91
TIMP2	2	-0.2710816	2.66E-12	4.85E-08	0.511	0.723

IGBP1	2	-0.2720525	9.61E-11	1.75E-06	0.543	0.709
LITAF	2	-0.2720592	7.01E-11	1.28E-06	0.403	0.588
TRPS1	2	-0.2721628	8.86E-09	0.00016176	0.391	0.556
IFI27L2	2	-0.2724314	1.16E-10	2.12E-06	0.76	0.901
RPL5	2	-0.2724459	2.25E-27	4.11E-23	1	1
TSPAN4	2	-0.2727701	1.70E-12	3.11E-08	0.339	0.565
CHPT1	2	-0.2735466	1.49E-12	2.72E-08	0.532	0.734
HSPA8	2	-0.2738706	5.72E-09	0.00010443	0.317	0.489
COL6A1	2	-0.2742745	3.19E-09	5.82E-05	0.355	0.531
VCAN	2	-0.2744594	1.46E-07	0.00266404	0.405	0.568
DAAM1	2	-0.2751531	4.52E-11	8.25E-07	0.204	0.41
IGFBP6	2	-0.2753985	4.80E-09	8.76E-05	0.554	0.737
RPL36AL	2	-0.2757431	9.43E-20	1.72E-15	0.998	0.997
MALAT1	2	-0.2776192	2.81E-10	5.13E-06	1	1
FXYD6	2	-0.2777834	4.26E-14	7.78E-10	0.758	0.932
BRK1	2	-0.2788382	1.43E-14	2.62E-10	0.812	0.932
EIF2S3	2	-0.2793722	7.84E-11	1.43E-06	0.482	0.698
NDRG2	2	-0.2798804	1.91E-20	3.48E-16	0.063	0.311
SEP1	2	-0.2807454	7.22E-14	1.32E-09	0.161	0.387
BEX2	2	-0.2810884	2.51E-13	4.58E-09	0.253	0.503
ZIC1	2	-0.2817592	4.70E-09	8.57E-05	0.179	0.347
HSPB1	2	-0.2818764	2.60E-11	4.75E-07	0.64	0.808
MTDH	2	-0.2827098	2.12E-12	3.86E-08	0.511	0.715
RPS25	2	-0.2828624	6.18E-39	1.13E-34	1	1
COL6A2	2	-0.2830203	2.21E-08	0.00040241	0.545	0.709
PRDX6	2	-0.283521	9.74E-14	1.78E-09	0.724	0.893
SPON2	2	-0.2842958	2.14E-11	3.90E-07	0.251	0.477
RPS4X	2	-0.2843425	4.84E-41	8.84E-37	1	1
CYSTM1	2	-0.2851341	4.81E-12	8.78E-08	0.428	0.633
AHNAK2	2	-0.2861571	1.22E-10	2.23E-06	0.296	0.511
SLC29A1	2	-0.2863385	8.62E-14	1.57E-09	0.244	0.48
HMGB1	2	-0.2864309	9.44E-13	1.72E-08	0.618	0.822
RBMX	2	-0.2865515	2.13E-12	3.88E-08	0.636	0.794
RARRES3	2	-0.2868214	1.31E-20	2.40E-16	0.043	0.277
PGAM1	2	-0.2875963	1.65E-13	3.02E-09	0.704	0.879
RPL27A	2	-0.2878737	3.34E-38	6.10E-34	1	1
RPS3	2	-0.2895055	1.45E-47	2.64E-43	1	1
RPS21	2	-0.2897192	1.81E-30	3.31E-26	1	1
RNF130	2	-0.2897751	1.33E-14	2.43E-10	0.473	0.729

DDR2	2	-0.2898753	1.38E-11	2.52E-07	0.455	0.678
AFF3	2	-0.291279	3.69E-17	6.73E-13	0.09	0.322
HERPUD1	2	-0.2913968	4.63E-13	8.45E-09	0.378	0.605
MYL12B	2	-0.2937049	9.35E-13	1.71E-08	0.462	0.664
EIF3E	2	-0.2939896	7.20E-19	1.31E-14	0.998	0.994
RAC1	2	-0.2940626	2.26E-15	4.13E-11	0.851	0.955
RPL15	2	-0.2947023	2.07E-54	3.78E-50	1	1
IGFBP7	2	-0.2953656	1.95E-08	0.00035575	0.855	0.907
COMMD6	2	-0.2965949	5.56E-26	1.01E-21	0.998	1
RPL28	2	-0.2968612	6.72E-41	1.23E-36	1	1
NFIC	2	-0.296931	4.23E-13	7.72E-09	0.296	0.525
FBL	2	-0.2970179	3.15E-14	5.75E-10	0.473	0.698
PLXDC2	2	-0.2979989	4.72E-09	8.62E-05	0.475	0.638
RPL11	2	-0.2984471	1.10E-45	2.01E-41	1	1
CEBPD	2	-0.2990218	3.80E-09	6.94E-05	0.731	0.864
NACA	2	-0.2996828	2.90E-32	5.29E-28	1	1
NMRK1	2	-0.3000609	2.20E-14	4.02E-10	0.251	0.492
RP5-1097P24.1	2	-0.3007656	2.91E-16	5.30E-12	0.163	0.415
RPL7A	2	-0.3009925	5.15E-41	9.40E-37	1	1
ST13	2	-0.3019268	7.90E-16	1.44E-11	0.921	0.963
MYLK	2	-0.3032384	1.31E-15	2.38E-11	0.131	0.364
NINJ1	2	-0.30457	2.10E-13	3.84E-09	0.5	0.701
C19orf43	2	-0.3045841	1.02E-16	1.87E-12	0.751	0.912
NDUFC2	2	-0.3056499	8.53E-12	1.56E-07	0.606	0.771
S100A13	2	-0.3072096	7.27E-24	1.33E-19	0.995	1
CD63	2	-0.3078577	4.81E-18	8.77E-14	1	1
SNAI2	2	-0.3080331	3.86E-11	7.04E-07	0.38	0.593
KRT8	2	-0.3084455	7.81E-15	1.43E-10	0.24	0.492
LRRC2	2	-0.3085534	1.32E-18	2.41E-14	0.154	0.429
GLTSCR2	2	-0.3092759	2.48E-15	4.52E-11	0.889	0.958
PPIB	2	-0.3099143	8.48E-13	1.55E-08	0.833	0.915
NUCB2	2	-0.3099173	1.34E-09	2.45E-05	0.344	0.52
FTL	2	-0.3107751	8.12E-28	1.48E-23	1	1
RPS6	2	-0.3109903	1.54E-56	2.82E-52	1	1
PLP2	2	-0.3112883	6.11E-15	1.12E-10	0.681	0.879
PRDX4	2	-0.3127334	4.73E-12	8.63E-08	0.706	0.859
CRYAB	2	-0.3143114	7.61E-08	0.00138795	0.656	0.794
EIF3M	2	-0.3147291	7.45E-15	1.36E-10	0.466	0.698
RPS12	2	-0.3154933	5.73E-41	1.05E-36	1	1

RPL35	2	-0.3156868	2.59E-46	4.72E-42	1	1
RPS9	2	-0.3157938	2.81E-47	5.13E-43	1	1
FCGRT	2	-0.3165244	1.23E-12	2.25E-08	0.48	0.678
TMED10	2	-0.3166642	3.98E-16	7.26E-12	0.828	0.941
EIF3F	2	-0.3169261	3.18E-16	5.80E-12	0.726	0.89
BBX	2	-0.3173231	2.06E-19	3.76E-15	0.219	0.508
MSRB1	2	-0.3182054	8.83E-23	1.61E-18	0.136	0.441
ATP5D	2	-0.3189672	2.24E-17	4.09E-13	0.81	0.949
MATN4	2	-0.3192117	1.98E-11	3.61E-07	0.136	0.328
DCN	2	-0.3196303	1.18E-18	2.16E-14	1	1
PPT1	2	-0.3197284	4.19E-15	7.65E-11	0.271	0.525
RPL35A	2	-0.3205264	5.20E-51	9.49E-47	1	1
CHID1	2	-0.320535	3.57E-13	6.52E-09	0.486	0.689
RPS24	2	-0.3212492	2.49E-37	4.55E-33	1	1
ARL2BP	2	-0.321465	1.64E-14	2.99E-10	0.369	0.613
LGALS1	2	-0.3227031	3.57E-13	6.52E-09	0.964	0.983
RPS27	2	-0.3236711	5.77E-49	1.05E-44	1	1
MZT2B	2	-0.324005	3.33E-24	6.08E-20	0.912	0.983
RGS10	2	-0.3259212	1.65E-13	3.01E-09	0.446	0.664
PPA1	2	-0.3260437	1.59E-19	2.89E-15	0.29	0.593
HOXB8	2	-0.3263158	3.30E-25	6.03E-21	0.068	0.359
DNM3OS	2	-0.3280512	4.01E-16	7.32E-12	0.222	0.489
RPL22	2	-0.3288171	2.65E-40	4.84E-36	1	1
PLA2G16	2	-0.3289108	1.40E-16	2.56E-12	0.256	0.52
EBPL	2	-0.3289851	2.94E-18	5.37E-14	0.317	0.605
CPE	2	-0.3296887	5.38E-16	9.82E-12	0.563	0.794
ATP5L	2	-0.3317721	4.90E-22	8.94E-18	0.928	0.975
EDF1	2	-0.332108	2.91E-27	5.32E-23	0.955	0.98
MPP6	2	-0.3359572	1.32E-20	2.41E-16	0.161	0.449
EIF2A	2	-0.3381655	1.14E-13	2.07E-09	0.64	0.802
PDIA3	2	-0.3384233	3.24E-13	5.91E-09	0.477	0.698
AC005152.3	2	-0.3386107	3.05E-21	5.57E-17	0.059	0.308
LPP	2	-0.3417434	2.25E-10	4.11E-06	0.344	0.54
ID4	2	-0.3423529	4.20E-14	7.66E-10	0.419	0.684
CUTA	2	-0.3430181	5.44E-16	9.93E-12	0.753	0.91
AP2M1	2	-0.343292	6.56E-16	1.20E-11	0.4	0.647
EIF3K	2	-0.3442378	3.30E-19	6.03E-15	0.796	0.918
CXXC5	2	-0.3467536	8.77E-19	1.60E-14	0.181	0.466
SLPI	2	-0.3485181	1.32E-08	0.00024067	0.98	0.994

MT-ND5	2	-0.3486361	1.04E-08	0.00019014	0.525	0.658
ATP5A1	2	-0.3501992	2.32E-17	4.23E-13	0.385	0.641
PDE4DIP	2	-0.3503137	4.24E-17	7.74E-13	0.265	0.534
RPL13	2	-0.3504189	4.77E-64	8.70E-60	1	1
CST3	2	-0.3509774	1.38E-16	2.52E-12	0.998	0.997
INSIG1	2	-0.3538201	8.38E-15	1.53E-10	0.281	0.542
ATP5O	2	-0.3552124	3.11E-19	5.68E-15	0.715	0.901
EIF3D	2	-0.3578298	4.90E-17	8.94E-13	0.661	0.847
DBP	2	-0.3591222	3.69E-20	6.73E-16	0.21	0.506
BSG	2	-0.3596834	7.98E-18	1.46E-13	0.557	0.794
UQCRH	2	-0.3598136	4.75E-27	8.67E-23	0.923	0.992
LDHB	2	-0.3600377	8.15E-18	1.49E-13	0.665	0.856
AC003090.1	2	-0.36036	3.91E-24	7.13E-20	0.106	0.407
S100A1	2	-0.3607891	2.80E-24	5.11E-20	0.995	1
NPM1	2	-0.3618148	1.28E-20	2.34E-16	1	1
SDF4	2	-0.362072	7.53E-19	1.37E-14	0.543	0.78
RPL37A	2	-0.3630442	1.82E-65	3.32E-61	1	1
RPL29	2	-0.3649735	4.31E-52	7.86E-48	1	1
RPL39	2	-0.3658353	1.45E-50	2.64E-46	1	1
PLPP1	2	-0.3670881	2.94E-18	5.37E-14	0.124	0.373
RPS23	2	-0.3690706	2.75E-61	5.02E-57	1	1
RPL18A	2	-0.3702739	2.05E-58	3.74E-54	1	1
TUBB	2	-0.370654	2.70E-14	4.92E-10	0.688	0.847
SPTLC3	2	-0.3708378	4.23E-16	7.72E-12	0.104	0.328
RPL21	2	-0.3736463	5.52E-72	1.01E-67	1	1
RPL19	2	-0.3740841	1.83E-67	3.35E-63	1	1
EID1	2	-0.379574	8.02E-31	1.46E-26	0.932	0.986
CYBRD1	2	-0.3816885	5.36E-20	9.77E-16	0.421	0.698
COL5A2	2	-0.3817266	1.84E-13	3.36E-09	0.208	0.432
TXNIP	2	-0.3822761	1.92E-30	3.51E-26	0.052	0.373
TOMM7	2	-0.3835564	1.26E-54	2.30E-50	1	1
S100A4	2	-0.3848396	5.30E-09	9.67E-05	0.749	0.879
RPL31	2	-0.3850906	1.14E-57	2.08E-53	1	1
RPL8	2	-0.3855593	1.31E-67	2.39E-63	1	1
TTC3	2	-0.3859372	6.36E-19	1.16E-14	0.595	0.833
MYL6	2	-0.3874652	5.29E-27	9.65E-23	0.896	0.972
BTF3	2	-0.3884256	4.05E-38	7.38E-34	0.986	0.997
PEBP1	2	-0.3892505	2.45E-24	4.47E-20	0.744	0.946
NKX3-2	2	-0.3895507	9.25E-21	1.69E-16	0.068	0.316

GREM1	2	-0.3920467	7.86E-13	1.43E-08	0.041	0.198
RPL37	2	-0.3939183	4.45E-62	8.12E-58	1	1
OXA1L	2	-0.3948558	4.54E-19	8.28E-15	0.38	0.644
HOXC10	2	-0.3953097	8.46E-21	1.54E-16	0.577	0.816
ENPP1	2	-0.3955718	9.58E-24	1.75E-19	0.17	0.5
UBA52	2	-0.3955871	7.01E-59	1.28E-54	1	1
ATP5G2	2	-0.3966398	5.54E-34	1.01E-29	0.995	1
PRUNE2	2	-0.3972473	2.29E-19	4.18E-15	0.24	0.528
FMOD	2	-0.3979643	1.52E-25	2.77E-21	0.923	0.992
COL11A1	2	-0.3988034	2.05E-13	3.73E-09	0.706	0.89
RPS18	2	-0.3993299	1.21E-64	2.22E-60	1	1
TPM1	2	-0.4003689	1.01E-11	1.85E-07	0.403	0.605
RPS5	2	-0.4009336	2.56E-59	4.67E-55	1	1
TSPO	2	-0.402345	3.57E-24	6.51E-20	0.71	0.921
HSP90AB1	2	-0.4024133	8.12E-23	1.48E-18	0.93	0.983
RPLP1	2	-0.406064	2.05E-73	3.75E-69	1	1
RPS7	2	-0.4070071	3.27E-58	5.97E-54	1	1
RPS13	2	-0.4072381	1.25E-62	2.29E-58	1	1
RPL10	2	-0.4093129	5.12E-75	9.35E-71	1	1
PTMS	2	-0.4102561	2.73E-23	4.99E-19	0.52	0.828
RPL3	2	-0.4114066	4.05E-66	7.39E-62	1	1
YBX1	2	-0.4153008	1.57E-37	2.86E-33	0.959	0.989
NGFRAP1	2	-0.4169068	3.49E-26	6.38E-22	0.837	0.966
NFIA	2	-0.4173217	1.35E-27	2.46E-23	0.206	0.568
PSMA7	2	-0.4200361	5.76E-23	1.05E-18	0.747	0.927
GNAS	2	-0.420732	2.67E-24	4.87E-20	0.457	0.746
GSTP1	2	-0.4217166	2.30E-26	4.20E-22	0.912	0.986
MIA	2	-0.4219258	2.00E-14	3.64E-10	0.975	0.992
RPL9	2	-0.423629	4.70E-74	8.58E-70	1	1
ERGIC3	2	-0.4242247	1.71E-26	3.13E-22	0.695	0.924
SLC44A1	2	-0.4244105	9.48E-28	1.73E-23	0.163	0.508
RBP4	2	-0.425874	1.66E-22	3.04E-18	0.722	0.921
SPINT2	2	-0.4281782	4.09E-22	7.46E-18	0.251	0.559
RPS27A	2	-0.4311873	3.75E-79	6.85E-75	1	1
RPL27	2	-0.4403419	1.55E-53	2.83E-49	1	1
TUBB2B	2	-0.4409435	3.06E-17	5.59E-13	0.283	0.551
ACAN	2	-0.4418578	2.92E-28	5.32E-24	0.946	0.997
INHBA	2	-0.4420364	1.58E-17	2.88E-13	0.251	0.54
PPIA	2	-0.4421834	2.71E-35	4.94E-31	0.948	0.986

RPL14	2	-0.4421929	3.73E-63	6.80E-59	1	1
FBXO2	2	-0.4422033	1.14E-27	2.08E-23	0.692	0.921
RPL13A	2	-0.4458935	2.68E-85	4.88E-81	1	1
SSPN	2	-0.4462488	7.45E-30	1.36E-25	0.192	0.562
NAP1L1	2	-0.4463692	1.45E-32	2.64E-28	0.91	0.997
NNMT	2	-0.453459	1.84E-22	3.36E-18	0.916	0.975
HINT1	2	-0.4543391	5.06E-40	9.23E-36	0.959	0.997
CTSC	2	-0.4553897	1.98E-16	3.61E-12	0.133	0.373
RPS2	2	-0.4557097	3.25E-78	5.93E-74	1	1
HLA-B	2	-0.4609523	3.90E-23	7.12E-19	0.724	0.912
HDLBP	2	-0.4612759	1.83E-24	3.34E-20	0.351	0.647
BGN	2	-0.4631184	1.70E-26	3.10E-22	0.977	1
RABAC1	2	-0.4655617	6.61E-32	1.21E-27	0.683	0.932
ID1	2	-0.466818	1.30E-12	2.36E-08	0.491	0.743
PPIC	2	-0.4676223	1.75E-20	3.19E-16	0.269	0.565
HNRNPA1	2	-0.4679197	7.00E-43	1.28E-38	0.989	0.997
RPSA	2	-0.4695821	2.09E-55	3.81E-51	1	1
PTRF	2	-0.4697367	1.28E-24	2.33E-20	0.357	0.655
NDUFA11	2	-0.4702763	2.13E-34	3.89E-30	0.774	0.949
RPL30	2	-0.4728611	7.16E-80	1.31E-75	1	1
MOXD1	2	-0.4730959	1.13E-33	2.06E-29	0.081	0.438
ID3	2	-0.4731512	6.15E-22	1.12E-17	0.414	0.771
FHL1	2	-0.475316	8.30E-21	1.51E-16	0.283	0.571
SSR4	2	-0.4758708	1.25E-32	2.28E-28	0.95	0.989
RHOBTB3	2	-0.4773597	1.59E-22	2.89E-18	0.527	0.811
ATRNL1	2	-0.4783258	4.46E-37	8.13E-33	0.115	0.523
ZBTB20	2	-0.479106	1.65E-30	3.01E-26	0.215	0.579
SLC25A3	2	-0.480824	2.55E-35	4.65E-31	0.808	0.972
PCBP2	2	-0.482241	2.84E-39	5.18E-35	0.878	0.98
RPL23	2	-0.4870696	3.17E-59	5.79E-55	0.998	1
HIST1H4C	2	-0.4900123	1.81E-23	3.29E-19	0.333	0.636
UQCRB	2	-0.4900319	3.45E-42	6.30E-38	0.975	1
CP	2	-0.4903933	6.91E-31	1.26E-26	0.658	0.963
CD99	2	-0.4905576	2.35E-37	4.29E-33	0.912	0.994
RPS29	2	-0.4910719	6.36E-83	1.16E-78	1	1
ITM2C	2	-0.4967916	2.72E-23	4.97E-19	0.439	0.743
SSR3	2	-0.4985619	3.15E-31	5.75E-27	0.817	0.96
RPL34	2	-0.503879	5.09E-87	9.28E-83	1	1
RPL23A	2	-0.5057831	1.90E-90	3.48E-86	1	1

PABPC1	2	-0.5067085	3.07E-39	5.61E-35	0.846	0.977
GAS6	2	-0.5080544	5.86E-25	1.07E-20	0.285	0.599
CRIP2	2	-0.5128834	3.07E-34	5.59E-30	0.217	0.605
RPS3A	2	-0.5149408	3.71E-93	6.77E-89	1	1
HLA-A	2	-0.520091	3.95E-30	7.20E-26	0.851	0.986
PDIA6	2	-0.5209554	4.37E-27	7.97E-23	0.511	0.805
SSR2	2	-0.5209946	2.72E-39	4.97E-35	0.706	0.946
CYR61	2	-0.5221692	8.20E-16	1.50E-11	0.294	0.545
EIF4B	2	-0.5253864	2.68E-31	4.89E-27	0.715	0.921
CITED2	2	-0.528145	1.13E-21	2.07E-17	0.26	0.562
GPX3	2	-0.5284901	1.22E-30	2.23E-26	0.93	0.986
PLAC9	2	-0.5306895	1.21E-28	2.20E-24	0.796	0.966
CCDC102B	2	-0.5312942	1.53E-47	2.79E-43	0.036	0.466
RPLP2	2	-0.5328527	1.03E-92	1.88E-88	1	1
CHRDL2	2	-0.5365063	3.11E-21	5.68E-17	0.152	0.441
RPS17	2	-0.5371477	3.26E-85	5.95E-81	1	1
MGP	2	-0.5408321	4.97E-30	9.07E-26	1	1
MAGED1	2	-0.549815	1.34E-21	2.44E-17	0.115	0.395
EEF1B2	2	-0.5505498	5.68E-55	1.04E-50	0.975	0.997
ARHGAP29	2	-0.5512934	1.13E-26	2.06E-22	0.285	0.616
RPL7	2	-0.5522325	1.35E-74	2.45E-70	1	1
TPT1	2	-0.5549318	1.40E-85	2.56E-81	1	1
RPS16	2	-0.5573653	3.52E-90	6.43E-86	1	1
MT-ND4	2	-0.5641868	1.19E-24	2.18E-20	0.966	0.955
HLA-C	2	-0.5694753	9.49E-37	1.73E-32	0.835	0.977
RPL18	2	-0.5746862	8.99E-90	1.64E-85	1	1
EIF3L	2	-0.5808319	3.20E-43	5.84E-39	0.76	0.966
P4HB	2	-0.5852912	3.11E-34	5.67E-30	0.729	0.941
TCF4	2	-0.5940778	1.85E-33	3.38E-29	0.29	0.667
COL9A3	2	-0.5970247	4.77E-23	8.71E-19	0.59	0.816
ITM2A	2	-0.5975989	1.49E-30	2.72E-26	0.419	0.74
SEC11A	2	-0.5998653	3.68E-51	6.72E-47	0.733	0.96
SLC14A1	2	-0.6102209	1.16E-39	2.12E-35	0.208	0.636
NPC2	2	-0.6174461	4.58E-65	8.35E-61	0.968	0.997
H3F3A	2	-0.6252803	2.91E-58	5.31E-54	0.9	1
RPL10A	2	-0.6276631	4.25E-87	7.76E-83	1	1
SERPINA5	2	-0.627929	5.01E-35	9.14E-31	0.448	0.76
HIST1H1C	2	-0.6328573	2.95E-18	5.38E-14	0.233	0.486
CCNI	2	-0.6350908	2.37E-67	4.33E-63	0.977	1

YBX3	2	-0.6358947	4.00E-52	7.30E-48	0.762	0.98
VIM	2	-0.6520509	1.96E-48	3.57E-44	1	1
RPL4	2	-0.6597815	2.06E-83	3.76E-79	0.998	1
RPS8	2	-0.6739271	2.06E-106	3.75E-102	1	1
CD9	2	-0.679401	1.68E-40	3.07E-36	0.353	0.754
THY1	2	-0.6798461	1.37E-35	2.50E-31	0.152	0.54
MT-ND1	2	-0.68063	3.91E-34	7.13E-30	0.563	0.876
LECT1	2	-0.6840437	6.62E-26	1.21E-21	0.86	0.952
EEF1A1	2	-0.685956	1.54E-102	2.82E-98	1	1
EEF2	2	-0.6868858	8.18E-57	1.49E-52	0.98	1
ISLR	2	-0.7267498	2.40E-47	4.37E-43	0.303	0.751
XAGE2	2	-0.7283743	1.06E-23	1.94E-19	0.014	0.24
RPLP0	2	-0.7624503	1.30E-106	2.37E-102	1	1
CTGF	2	-0.7936555	5.72E-33	1.04E-28	0.244	0.641
MT-CO3	2	-0.8045678	5.55E-39	1.01E-34	0.873	0.941
NEAT1	2	-0.8076964	5.04E-60	9.20E-56	0.977	1
MT-CO2	2	-0.8158382	3.52E-39	6.42E-35	0.946	0.966
MT-ATP6	2	-0.8191801	2.94E-38	5.36E-34	0.794	0.91
TSC22D1	2	-0.8227657	4.26E-29	7.78E-25	0.647	0.91
ACTG1	2	-0.8230521	2.42E-57	4.41E-53	0.876	0.989
CRTAP	2	-0.8405446	3.80E-72	6.94E-68	0.471	0.91
PAX1	2	-0.846734	1.08E-64	1.98E-60	0.529	0.963
APOD	2	-0.8506216	9.28E-34	1.69E-29	0.206	0.573
SLC40A1	2	-0.8553132	6.22E-58	1.13E-53	0.072	0.588
MT-CO1	2	-0.8625966	4.54E-39	8.28E-35	0.968	0.972
EIF3H	2	-0.8713126	1.16E-90	2.12E-86	0.95	0.997
B2M	2	-0.8744569	2.02E-86	3.68E-82	0.977	1
MT-ND2	2	-0.8840604	3.27E-39	5.96E-35	0.6	0.862
FTH1	2	-0.8977603	1.52E-84	2.77E-80	1	1
C2orf82	2	-0.9464014	1.83E-50	3.34E-46	0.957	1
MT-CYB	2	-0.9618583	2.92E-47	5.32E-43	0.593	0.893
CRISPLD1	2	-0.9712115	8.17E-52	1.49E-47	0.063	0.531
RPS20	2	-0.9887685	1.40E-118	2.55E-114	0.995	1
CAPS	2	-1.0121777	9.03E-64	1.65E-59	0.624	0.963
ZFP36L2	2	-1.0171251	6.61E-55	1.21E-50	0.133	0.641
RPS11	2	-1.018521	3.72E-118	6.79E-114	0.986	1
MT-ND3	2	-1.0186145	4.36E-56	7.96E-52	0.6	0.91
SPARC	2	-1.0457017	1.84E-59	3.36E-55	0.269	0.802
COL2A1	2	-1.1307089	2.12E-47	3.87E-43	0.541	0.941

ADIRF	2	-1.2586168	1.33E-72	2.42E-68	0.276	0.847
OGN	2	-1.3108085	1.19E-66	2.18E-62	0.894	1
CLEC3A	2	-1.3293304	1.07E-75	1.95E-71	0.355	0.907
SPTSSB	2	-1.5210974	1.45E-92	2.65E-88	0.014	0.706
MSMP	2	-1.5309741	4.97E-14	9.06E-10	0.124	0.339
FRZB	2	-1.9474534	2.93E-65	5.35E-61	0.283	0.816
**gene**	**cluster**	**avg_logFC**	**p_val**	**p_val_adj**	**pct.1**	**pct.2**
PRG4	3	1.65854155	8.41E-34	1.54E-29	0.537	0.066
SPP1	3	1.56672108	1.12E-45	2.04E-41	0.779	0.172
CCL20	3	1.48994485	9.45E-11	1.72E-06	0.192	0.02
PTGS2	3	1.14541394	3.52E-48	6.43E-44	0.694	0.102
PLA2G2A	3	1.14267119	6.03E-31	1.10E-26	0.678	0.188
VEGFA	3	1.111942	1.38E-50	2.51E-46	0.876	0.387
IGFBP3	3	1.07109267	5.43E-28	9.90E-24	0.492	0.082
G0S2	3	1.06604824	1.07E-15	1.94E-11	0.707	0.422
UPP1	3	1.04587567	6.56E-39	1.20E-34	0.691	0.199
CCDC85B	3	0.97010448	8.18E-33	1.49E-28	0.948	0.797
CTSL	3	0.94295722	2.87E-16	5.25E-12	0.795	0.613
S100A2	3	0.93099374	5.42E-18	9.89E-14	0.678	0.398
COMP	3	0.91913017	1.99E-26	3.63E-22	0.948	0.785
BMP2	3	0.91224934	3.33E-37	6.08E-33	0.645	0.105
ERO1A	3	0.84214077	4.63E-30	8.44E-26	0.752	0.434
STK17A	3	0.80037181	2.88E-27	5.26E-23	0.593	0.188
TREM1	3	0.78341059	1.05E-40	1.92E-36	0.554	0.023
PHLDA1	3	0.78053785	3.49E-23	6.37E-19	0.56	0.16
TNFRSF12A	3	0.75550632	8.67E-15	1.58E-10	0.55	0.289
CHI3L2	3	0.74544145	9.75E-21	1.78E-16	0.964	0.766
FOSL1	3	0.73605824	4.37E-27	7.98E-23	0.482	0.074
TNFAIP6	3	0.72323511	1.80E-18	3.29E-14	0.306	0.023
HILPDA	3	0.69364207	9.88E-22	1.80E-17	0.72	0.383
MRPS6	3	0.69154789	7.40E-16	1.35E-11	0.977	0.93
TGFBI	3	0.6779144	9.65E-26	1.76E-21	0.58	0.176
IGFBP5	3	0.67536473	6.15E-14	1.12E-09	0.358	0.098
PPP3CA	3	0.66490942	9.81E-20	1.79E-15	0.922	0.691
MT1X	3	0.64974092	4.46E-20	8.13E-16	1	1
DNAJB9	3	0.64741437	5.97E-21	1.09E-16	0.759	0.465
TMEM45A	3	0.64005416	3.72E-24	6.79E-20	0.928	0.688
ARF6	3	0.61726649	9.41E-24	1.72E-19	0.792	0.539
CTHRC1	3	0.61631094	6.12E-23	1.12E-18	0.466	0.098

MT2A	3	0.61044927	1.52E-22	2.78E-18	1	1
NRP2	3	0.57774495	1.82E-17	3.33E-13	0.573	0.258
SLC2A1	3	0.56055736	1.85E-17	3.38E-13	0.531	0.211
BZW1	3	0.53298308	9.45E-18	1.72E-13	0.808	0.625
WTAP	3	0.53208498	9.49E-14	1.73E-09	0.961	0.84
PPP1R15A	3	0.50611493	1.95E-17	3.55E-13	0.619	0.305
PTP4A1	3	0.49605241	1.95E-12	3.55E-08	0.59	0.348
ERRFI1	3	0.48874863	1.70E-09	3.10E-05	0.463	0.246
CA9	3	0.48855324	2.25E-14	4.10E-10	0.534	0.254
SELK	3	0.48365018	2.72E-19	4.96E-15	0.945	0.793
RNF145	3	0.47832467	8.98E-16	1.64E-11	0.521	0.227
SLC25A37	3	0.4777184	3.34E-21	6.09E-17	0.974	0.789
HMGA2	3	0.47468667	3.91E-27	7.13E-23	0.417	0.027
ENO2	3	0.46576169	3.29E-14	6.00E-10	0.655	0.43
ARF4	3	0.4470625	2.53E-10	4.62E-06	0.72	0.602
FAM162A	3	0.44670604	1.58E-16	2.88E-12	0.958	0.793
SLC39A8	3	0.44148132	1.63E-15	2.97E-11	0.648	0.359
C15orf48	3	0.43799751	4.24E-11	7.74E-07	0.511	0.23
HLA-DRB1	3	0.43736891	3.24E-12	5.90E-08	0.534	0.297
HMGA1	3	0.43503989	1.24E-06	0.02254533	0.596	0.441
XBP1	3	0.42918373	5.34E-13	9.75E-09	0.743	0.531
BHLHE40	3	0.42542602	3.00E-08	0.00054654	0.609	0.414
TNFSF11	3	0.41733789	2.96E-19	5.40E-15	0.27	0
PTGES3	3	0.4166747	3.91E-22	7.13E-18	0.974	0.891
NDRG1	3	0.4134271	1.14E-12	2.08E-08	0.762	0.535
WDR45B	3	0.41323844	6.61E-16	1.21E-11	0.573	0.262
PPP1CB	3	0.4100338	1.99E-07	0.00363482	0.844	0.734
SGIP1	3	0.40570322	8.70E-20	1.59E-15	0.368	0.051
INSIG2	3	0.40292147	2.47E-09	4.50E-05	0.609	0.422
SLC5A3	3	0.40267402	5.90E-08	0.00107662	0.645	0.484
RABGGTB	3	0.40220246	1.18E-15	2.16E-11	0.818	0.582
PLAUR	3	0.40108174	1.19E-10	2.18E-06	0.528	0.293
IL11	3	0.40002144	1.15E-12	2.10E-08	0.179	0
CCND1	3	0.39526001	2.08E-12	3.79E-08	0.446	0.18
SPRY2	3	0.39435152	1.95E-11	3.57E-07	0.332	0.105
ISG20	3	0.39135351	2.30E-10	4.20E-06	0.71	0.543
EIF4EBP1	3	0.37965693	1.17E-09	2.13E-05	0.599	0.395
NAMPT	3	0.37866551	1.93E-12	3.53E-08	0.808	0.645
IL32	3	0.37832434	1.89E-11	3.44E-07	0.498	0.227

GAPDH	3	0.37276338	8.88E-13	1.62E-08	1	1
DDIT4L	3	0.37003838	1.11E-09	2.02E-05	0.899	0.734
PPFIBP1	3	0.36819587	8.88E-08	0.00162035	0.612	0.484
EVI2A	3	0.36331913	1.12E-12	2.04E-08	0.43	0.168
CLDND1	3	0.36071718	4.46E-13	8.14E-09	0.606	0.348
ARPP19	3	0.3588232	2.81E-12	5.13E-08	0.71	0.492
EGLN3	3	0.354519	3.08E-08	0.00056218	0.407	0.211
CFD	3	0.35400944	6.13E-09	0.00011192	0.29	0.094
CD44	3	0.35313729	4.57E-13	8.35E-09	0.974	0.898
SMOX	3	0.35020873	1.08E-06	0.01968354	0.479	0.32
GOLT1B	3	0.34168396	8.50E-09	0.00015518	0.502	0.305
CLMP	3	0.33847629	4.65E-13	8.49E-09	0.414	0.145
TFPI	3	0.33736892	8.27E-12	1.51E-07	0.345	0.102
RNMT	3	0.33516463	1.54E-11	2.80E-07	0.57	0.312
TMEM38B	3	0.33370925	6.29E-10	1.15E-05	0.707	0.52
CSGALNACT2	3	0.32787655	2.33E-08	0.00042539	0.394	0.203
TNFAIP8	3	0.32510673	1.33E-10	2.42E-06	0.342	0.113
GBE1	3	0.32270736	1.20E-07	0.00218679	0.713	0.586
RELB	3	0.32060826	3.55E-10	6.49E-06	0.625	0.391
AK2	3	0.31961532	3.83E-09	6.98E-05	0.814	0.684
RAB13	3	0.31935132	4.21E-12	7.68E-08	0.954	0.895
NT5C3A	3	0.31929088	1.43E-06	0.02603462	0.394	0.227
CDK5RAP2	3	0.31623437	2.60E-06	0.0474066	0.283	0.133
C11orf31	3	0.31522901	6.33E-11	1.16E-06	0.805	0.637
RAB1A	3	0.31034388	4.05E-09	7.40E-05	0.805	0.676
PHLDA2	3	0.30741533	8.77E-10	1.60E-05	0.267	0.07
SLC7A2	3	0.30723541	2.71E-06	0.04951181	0.518	0.336
VIMP	3	0.30543577	1.08E-07	0.00197043	0.717	0.59
PGK1	3	0.2997677	1.67E-07	0.00304013	0.958	0.938
VAPA	3	0.29386767	1.66E-08	0.00030377	0.941	0.883
C4orf48	3	0.29229373	2.43E-10	4.43E-06	0.42	0.184
A4GALT	3	0.29199112	1.29E-09	2.35E-05	0.388	0.164
RNF126	3	0.28792608	1.58E-10	2.89E-06	0.423	0.188
SMIM15	3	0.28620037	1.19E-09	2.17E-05	0.537	0.309
COX17	3	0.28526113	3.18E-09	5.79E-05	0.86	0.695
SEC61G	3	0.28009616	2.07E-08	0.00037686	0.997	0.988
MGST1	3	0.27626586	5.14E-07	0.00937129	0.476	0.266
MIF	3	0.27534169	1.73E-14	3.15E-10	1	1
PTGES	3	0.27335177	3.35E-11	6.12E-07	0.254	0.051

BCL2A1	3	0.26891524	2.17E-10	3.96E-06	0.173	0.012
CISD2	3	0.26871865	1.07E-07	0.00196118	0.599	0.426
TNS1	3	0.26683656	4.66E-09	8.51E-05	0.319	0.121
TMSB10	3	0.26620553	5.04E-07	0.0091955	0.984	0.988
SERPINE1	3	0.26319735	5.51E-10	1.00E-05	0.277	0.074
KDM7A	3	0.26136829	3.31E-07	0.00604429	0.316	0.145
LTBP1	3	0.26042894	6.51E-13	1.19E-08	0.257	0.035
PAIP2	3	0.25871708	2.71E-06	0.04951133	0.743	0.672
TVP23B	3	0.25708078	7.61E-07	0.01388138	0.427	0.258
SPHK1	3	0.25568817	2.98E-08	0.00054417	0.303	0.117
VPS37A	3	0.25499691	4.40E-10	8.02E-06	0.384	0.148
BNIP3	3	0.25461202	3.59E-07	0.0065549	1	0.988
TRAM1	3	0.25332392	4.75E-07	0.00866311	0.736	0.652
SNRNP27	3	0.25196568	1.52E-07	0.00278079	0.557	0.371
UBE2I	3	0.25189684	1.44E-07	0.00263419	0.759	0.602
SLC25A36	3	0.25100208	9.11E-07	0.01662222	0.599	0.422
MZT2B	3	-0.2509082	8.29E-09	0.00015121	0.915	0.953
ATP5A1	3	-0.2529876	1.27E-07	0.00232547	0.427	0.621
HMGB2	3	-0.2532068	8.84E-07	0.01613316	0.316	0.512
RPL39	3	-0.253489	4.73E-16	8.64E-12	1	1
RPL12	3	-0.253632	1.41E-17	2.57E-13	1	1
SP5	3	-0.2539152	4.24E-14	7.74E-10	0	0.172
RBMX	3	-0.2549095	7.26E-07	0.01324175	0.632	0.77
TTC3	3	-0.2550834	1.50E-08	0.00027401	0.567	0.77
CTSK	3	-0.2551728	7.19E-10	1.31E-05	0.14	0.355
AC003090.1	3	-0.2559987	2.39E-13	4.36E-09	0.052	0.273
MRC2	3	-0.2566333	1.84E-13	3.36E-09	0.085	0.328
ZNF90	3	-0.2587727	1.90E-07	0.00346735	0.453	0.633
IL13RA1	3	-0.258817	1.41E-10	2.58E-06	0.238	0.492
CD59	3	-0.259312	1.22E-09	2.23E-05	0.909	0.957
ATP5D	3	-0.2598011	5.27E-08	0.00096132	0.785	0.91
TOMM7	3	-0.260467	2.84E-20	5.18E-16	1	1
RPS6	3	-0.2614557	1.67E-24	3.05E-20	1	1
IFI27L2	3	-0.2629576	3.60E-09	6.57E-05	0.713	0.875
GSTP1	3	-0.2629923	4.31E-10	7.86E-06	0.945	0.973
ANGPTL2	3	-0.2631633	1.89E-06	0.03453298	0.283	0.445
PTMA	3	-0.2639632	8.25E-17	1.50E-12	1	1
SLC25A5	3	-0.2639635	1.91E-08	0.00034898	0.407	0.621
SDF4	3	-0.2643883	2.27E-06	0.04149088	0.58	0.719

HNRNPA3	3	-0.2644243	6.96E-07	0.01270759	0.459	0.621
BRK1	3	-0.2648323	3.29E-11	6.01E-07	0.811	0.934
RPL7A	3	-0.2651549	1.50E-20	2.74E-16	1	1
C19orf43	3	-0.2659191	7.17E-11	1.31E-06	0.801	0.953
RPL31	3	-0.2659881	1.67E-20	3.05E-16	1	1
PARK7	3	-0.2660446	2.65E-10	4.83E-06	0.801	0.926
CUTA	3	-0.2661324	6.86E-09	0.00012523	0.759	0.898
DPYSL3	3	-0.2667696	7.24E-11	1.32E-06	0.121	0.344
NDUFB1	3	-0.2683985	3.98E-11	7.26E-07	0.818	0.922
SMIM19	3	-0.2690719	1.78E-08	0.00032442	0.319	0.527
EIF2A	3	-0.269297	2.14E-07	0.00390854	0.606	0.77
H2AFJ	3	-0.2693553	9.31E-08	0.00169887	0.658	0.828
SNRPE	3	-0.2697581	7.41E-09	0.00013527	0.632	0.781
RPS12	3	-0.269866	1.07E-23	1.95E-19	1	1
RPS4X	3	-0.2698882	1.96E-22	3.58E-18	1	1
PDE4DIP	3	-0.2710598	1.80E-09	3.29E-05	0.208	0.43
AP2M1	3	-0.2711686	1.22E-08	0.00022291	0.453	0.656
PPT1	3	-0.2713478	6.31E-09	0.0001151	0.332	0.551
RPL8	3	-0.2718141	4.07E-25	7.43E-21	1	1
CNBP	3	-0.2720163	2.76E-09	5.04E-05	0.834	0.934
RPL21	3	-0.2723655	6.84E-29	1.25E-24	1	1
SSR2	3	-0.2725869	2.23E-08	0.00040628	0.73	0.871
EIF3K	3	-0.2733966	3.54E-09	6.46E-05	0.814	0.922
RPL13	3	-0.2734184	2.25E-26	4.10E-22	1	1
RPS5	3	-0.2734293	7.72E-23	1.41E-18	1	1
BBX	3	-0.27389	1.04E-09	1.90E-05	0.221	0.438
PRKCSH	3	-0.274875	4.95E-09	9.03E-05	0.218	0.438
EDF1	3	-0.2754409	5.25E-12	9.58E-08	0.932	0.977
ENPP1	3	-0.2762076	4.46E-10	8.14E-06	0.127	0.34
ZBTB20	3	-0.2769805	3.71E-10	6.77E-06	0.202	0.438
WBP2	3	-0.2776587	1.29E-06	0.0234616	0.505	0.633
OS9	3	-0.277979	1.47E-09	2.67E-05	0.349	0.594
MYL12B	3	-0.2788003	3.93E-08	0.00071772	0.485	0.676
AHNAK	3	-0.2788726	1.70E-09	3.10E-05	0.378	0.609
PGAM1	3	-0.2798135	2.80E-09	5.11E-05	0.697	0.855
CHID1	3	-0.2809253	4.85E-10	8.86E-06	0.352	0.602
MTDH	3	-0.2810196	2.66E-09	4.86E-05	0.528	0.742
HDLBP	3	-0.2813378	2.25E-06	0.04102322	0.371	0.52
LTA4H	3	-0.2826569	2.58E-09	4.71E-05	0.508	0.691

SSPN	3	-0.2856253	2.18E-13	3.97E-09	0.134	0.398
CYSTM1	3	-0.2868328	1.08E-09	1.97E-05	0.469	0.672
UBE2R2	3	-0.2874771	3.39E-09	6.18E-05	0.358	0.57
TCEA3	3	-0.2877768	4.66E-12	8.50E-08	0.228	0.504
ERGIC3	3	-0.2887914	8.23E-10	1.50E-05	0.642	0.848
MYLK	3	-0.2888149	8.62E-12	1.57E-07	0.088	0.309
ITM2C	3	-0.2892118	1.62E-08	0.00029481	0.316	0.531
RPL14	3	-0.2896371	3.92E-22	7.14E-18	1	1
DST	3	-0.2898839	1.16E-08	0.0002114	0.375	0.598
ARPC1A	3	-0.2914514	2.02E-09	3.69E-05	0.404	0.602
BSG	3	-0.2918886	2.63E-09	4.81E-05	0.534	0.727
CXXC5	3	-0.2926019	1.19E-09	2.17E-05	0.15	0.355
SEPP1	3	-0.2928959	4.03E-10	7.35E-06	0.244	0.484
RP11-983P16.4	3	-0.2931442	7.78E-11	1.42E-06	0.303	0.547
RPL37A	3	-0.2935344	9.09E-31	1.66E-26	1	1
ITGA10	3	-0.2942892	3.69E-07	0.00672656	0.362	0.547
H1FX	3	-0.29439	8.77E-13	1.60E-08	0.124	0.379
ATP5F1	3	-0.297001	1.79E-09	3.27E-05	0.436	0.656
CRISPLD1	3	-0.2976993	2.13E-10	3.89E-06	0.029	0.191
RPS7	3	-0.2987508	1.69E-24	3.09E-20	1	1
PDIA3	3	-0.299655	8.80E-08	0.00160617	0.433	0.656
MPP6	3	-0.3000739	2.60E-13	4.74E-09	0.15	0.414
KRT8	3	-0.3008041	3.39E-09	6.18E-05	0.274	0.496
NFIA	3	-0.3012933	1.41E-12	2.57E-08	0.143	0.402
LRP10	3	-0.3015088	1.28E-09	2.33E-05	0.3	0.531
GRN	3	-0.3016296	4.80E-11	8.75E-07	0.212	0.473
COL6A1	3	-0.302651	5.10E-09	9.31E-05	0.329	0.543
PSMA7	3	-0.3030611	2.96E-10	5.41E-06	0.785	0.926
RPL27	3	-0.3044553	5.07E-23	9.26E-19	1	1
CHMP3	3	-0.3049935	1.74E-11	3.18E-07	0.401	0.648
HADHA	3	-0.3050067	2.04E-09	3.72E-05	0.303	0.512
PRELP	3	-0.3053353	3.33E-07	0.00606716	0.928	0.977
TPD52L1	3	-0.305338	2.52E-09	4.60E-05	0.3	0.52
IGFBP6	3	-0.3055659	8.69E-07	0.01585972	0.518	0.691
PKM	3	-0.3059126	1.65E-06	0.03002476	0.899	0.98
ANXA4	3	-0.3075252	3.36E-10	6.13E-06	0.319	0.551
RPS18	3	-0.3081521	4.81E-29	8.77E-25	1	1
NBEAL1	3	-0.3098649	3.38E-13	6.16E-09	0.984	0.992
PIK3R1	3	-0.3119155	7.36E-08	0.00134221	0.169	0.363

RPL13A	3	-0.3141491	2.28E-36	4.17E-32	1	1
UQCRHL	3	-0.315505	4.75E-12	8.66E-08	0.287	0.547
UQCRH	3	-0.3162138	4.74E-16	8.65E-12	0.912	0.988
RPA3	3	-0.3164712	2.62E-10	4.78E-06	0.43	0.641
ANXA1	3	-0.317366	3.29E-09	6.01E-05	0.632	0.844
RPL19	3	-0.3175765	5.84E-36	1.07E-31	1	1
UBA52	3	-0.3181634	1.75E-28	3.19E-24	1	1
METTL7A	3	-0.3184954	6.18E-09	0.00011269	0.094	0.277
RPS2	3	-0.3193403	1.66E-30	3.02E-26	1	1
ATP5G2	3	-0.3197594	2.81E-18	5.12E-14	0.997	0.992
ATP5O	3	-0.3199076	1.09E-12	1.99E-08	0.723	0.875
COMMD6	3	-0.3209632	2.79E-21	5.10E-17	1	1
EIF3D	3	-0.321724	1.76E-09	3.21E-05	0.694	0.828
XAGE2	3	-0.3232598	1.99E-09	3.64E-05	0.007	0.129
CRYAB	3	-0.3237435	6.52E-09	0.00011891	0.564	0.785
CST3	3	-0.324489	7.55E-11	1.38E-06	0.984	1
GSN	3	-0.3246446	4.69E-11	8.55E-07	0.436	0.664
HSP90AB1	3	-0.3253928	5.15E-13	9.39E-09	0.941	0.969
OXA1L	3	-0.3268523	8.20E-11	1.50E-06	0.42	0.633
ATRNL1	3	-0.3278528	1.52E-17	2.77E-13	0.055	0.328
PDLIM4	3	-0.3286441	1.97E-08	0.00036019	0.713	0.863
PRCP	3	-0.3290756	1.87E-15	3.42E-11	0.098	0.371
SLC39A14	3	-0.3295483	4.27E-11	7.79E-07	0.801	0.902
CRIP2	3	-0.3299969	3.06E-12	5.57E-08	0.186	0.441
PPIC	3	-0.3310477	1.27E-11	2.32E-07	0.153	0.395
SCARA3	3	-0.3323865	7.08E-12	1.29E-07	0.179	0.434
SSR3	3	-0.3327423	1.67E-10	3.04E-06	0.785	0.91
EIF2S3	3	-0.3331915	1.32E-11	2.41E-07	0.469	0.711
TSPAN4	3	-0.3335405	1.00E-09	1.83E-05	0.329	0.539
RGS10	3	-0.3336865	5.15E-13	9.41E-09	0.482	0.734
COL11A1	3	-0.3346034	2.46E-10	4.49E-06	0.45	0.703
ATPIF1	3	-0.3360668	1.00E-11	1.83E-07	0.502	0.766
LITAF	3	-0.3367191	9.91E-13	1.81E-08	0.404	0.652
RPS13	3	-0.3370681	1.03E-32	1.88E-28	1	1
GNAS	3	-0.3372688	8.26E-12	1.51E-07	0.456	0.656
RPS23	3	-0.3383728	6.38E-36	1.16E-31	1	1
NAP1L1	3	-0.3386098	1.05E-15	1.91E-11	0.967	0.977
ILF3-AS1	3	-0.3388083	1.64E-14	2.99E-10	0.205	0.5
PRDX5	3	-0.3390108	3.45E-16	6.30E-12	0.818	0.961

CYBRD1	3	-0.3390827	1.21E-10	2.21E-06	0.401	0.629
RPL10	3	-0.3412254	1.29E-33	2.35E-29	1	1
RPL30	3	-0.3427073	4.93E-35	9.00E-31	1	1
NMRK1	3	-0.3430464	2.02E-14	3.68E-10	0.235	0.527
PFN1	3	-0.345399	2.27E-11	4.14E-07	0.814	0.941
DBP	3	-0.3473818	1.61E-16	2.94E-12	0.107	0.398
HMGB1	3	-0.347881	4.33E-12	7.91E-08	0.632	0.855
LINC01503	3	-0.3481185	5.52E-12	1.01E-07	0.179	0.43
RPLP1	3	-0.3493832	1.38E-39	2.52E-35	1	1
NFIC	3	-0.3500056	3.86E-11	7.04E-07	0.287	0.523
ARL6IP5	3	-0.3502026	2.90E-14	5.28E-10	0.476	0.789
MIA	3	-0.3506076	4.59E-07	0.00838199	0.974	0.965
CMB9-22P13.1	3	-0.352104	7.27E-13	1.33E-08	0.27	0.535
SPINT2	3	-0.3524459	4.08E-11	7.44E-07	0.231	0.453
GAS6	3	-0.3545941	9.74E-13	1.78E-08	0.215	0.488
DMKN	3	-0.3550523	5.53E-14	1.01E-09	0.27	0.551
LUM	3	-0.3552749	1.25E-19	2.27E-15	1	1
PDIA6	3	-0.3580995	8.11E-12	1.48E-07	0.427	0.699
PCBP2	3	-0.3586265	5.66E-16	1.03E-11	0.87	0.961
RPS29	3	-0.3595662	1.43E-41	2.62E-37	1	1
ASS1	3	-0.3599185	3.46E-12	6.31E-08	0.495	0.754
CPE	3	-0.3610112	2.37E-12	4.32E-08	0.57	0.805
CTSC	3	-0.3618799	5.09E-08	0.0009296	0.169	0.367
KLF6	3	-0.3622984	1.12E-10	2.05E-06	0.202	0.441
POLR2L	3	-0.3627878	4.27E-11	7.79E-07	0.743	0.863
NR1D1	3	-0.362823	2.96E-08	0.00054066	0.277	0.48
BTF3	3	-0.3641456	1.35E-25	2.46E-21	0.993	1
FHL1	3	-0.3684643	1.80E-16	3.29E-12	0.244	0.57
DDR2	3	-0.3740422	1.61E-12	2.94E-08	0.29	0.559
AHNAK2	3	-0.376643	3.71E-13	6.77E-09	0.283	0.566
C9orf16	3	-0.3809333	9.86E-10	1.80E-05	0.466	0.668
SSR4	3	-0.3816585	1.06E-12	1.93E-08	0.866	0.977
NOVA1	3	-0.3817134	1.94E-09	3.54E-05	0.801	0.918
RPL9	3	-0.3831885	3.22E-41	5.87E-37	1	1
CD9	3	-0.3841219	8.54E-13	1.56E-08	0.322	0.59
PPIA	3	-0.3846768	1.76E-18	3.21E-14	0.906	0.977
RPL37	3	-0.3866551	2.86E-39	5.22E-35	1	1
RPS27A	3	-0.3886282	5.72E-43	1.04E-38	1	1
RPL3	3	-0.3891292	7.14E-35	1.30E-30	1	1

SLC14A1	3	-0.3911711	5.19E-14	9.47E-10	0.163	0.434
IGFBP7	3	-0.3925199	8.15E-07	0.01487025	0.831	0.859
LPP	3	-0.392727	7.76E-12	1.42E-07	0.508	0.715
RBP4	3	-0.3938106	1.32E-08	0.00024001	0.648	0.805
MALAT1	3	-0.3939635	1.43E-13	2.62E-09	1	1
LDHB	3	-0.3951868	1.13E-15	2.06E-11	0.642	0.848
FGF7	3	-0.4034585	1.78E-11	3.24E-07	0.156	0.414
NDUFA11	3	-0.4040916	4.27E-17	7.79E-13	0.733	0.93
COL9A3	3	-0.4058532	8.17E-08	0.00149044	0.404	0.613
RPS16	3	-0.4105488	1.63E-42	2.97E-38	1	1
SLC29A1	3	-0.4119677	1.16E-14	2.12E-10	0.235	0.516
F13A1	3	-0.4131127	5.33E-12	9.73E-08	0.189	0.441
RPL23A	3	-0.4178442	1.04E-45	1.90E-41	1	1
NUPR1	3	-0.4184134	3.33E-13	6.07E-09	0.951	0.988
HTRA1	3	-0.4196722	1.18E-17	2.16E-13	0.655	0.918
MAF	3	-0.4204668	8.90E-14	1.62E-09	0.186	0.461
ITM2A	3	-0.4213082	4.04E-15	7.37E-11	0.309	0.617
FBXO2	3	-0.4225081	2.02E-16	3.68E-12	0.573	0.852
YBX1	3	-0.4227847	4.13E-28	7.53E-24	0.971	0.988
NGFRAP1	3	-0.4242684	1.52E-19	2.77E-15	0.691	0.93
MYL6	3	-0.4245441	2.94E-23	5.36E-19	0.886	0.984
TUBB	3	-0.428656	2.67E-15	4.88E-11	0.691	0.879
RPL23	3	-0.4302741	3.61E-36	6.58E-32	0.997	1
SLC25A3	3	-0.4353997	1.09E-22	1.98E-18	0.814	0.988
SPON2	3	-0.4372904	2.07E-13	3.78E-09	0.176	0.457
S100A13	3	-0.4382536	3.18E-27	5.80E-23	0.967	1
TSPO	3	-0.4383654	4.57E-17	8.33E-13	0.681	0.902
CCDC102B	3	-0.4396432	8.42E-25	1.54E-20	0.023	0.352
HLA-A	3	-0.4407631	1.04E-12	1.90E-08	0.883	0.977
RPSA	3	-0.4409796	4.89E-31	8.93E-27	1	1
SEC11A	3	-0.4411458	3.94E-24	7.19E-20	0.743	0.949
DCN	3	-0.4414994	2.49E-24	4.55E-20	1	1
SLC40A1	3	-0.4419534	8.77E-16	1.60E-11	0.075	0.336
ID3	3	-0.4484649	3.71E-14	6.77E-10	0.287	0.625
CITED2	3	-0.4518199	4.59E-13	8.38E-09	0.16	0.414
EEF1B2	3	-0.4566195	4.03E-29	7.35E-25	0.98	0.992
RPLP2	3	-0.456727	3.86E-53	7.05E-49	1	1
FXYD6	3	-0.4570346	1.11E-20	2.02E-16	0.684	0.922
RPL18	3	-0.4671147	2.96E-45	5.40E-41	1	1

EIF4B	3	-0.4702745	1.29E-17	2.36E-13	0.756	0.883
HLA-B	3	-0.4704213	4.92E-14	8.98E-10	0.749	0.898
PEBP1	3	-0.4747215	1.58E-24	2.88E-20	0.658	0.926
PTMS	3	-0.4795565	5.32E-19	9.71E-15	0.466	0.797
INHBA	3	-0.4830769	1.22E-19	2.22E-15	0.156	0.504
UQCRB	3	-0.4864167	5.01E-26	9.14E-22	0.948	0.996
CD99	3	-0.487052	3.66E-26	6.68E-22	0.876	0.93
RABAC1	3	-0.4872901	1.40E-21	2.56E-17	0.632	0.879
RPS17	3	-0.4884654	1.27E-51	2.31E-47	1	1
RPS3A	3	-0.4919672	2.58E-52	4.71E-48	1	1
FMOD	3	-0.4923649	9.86E-19	1.80E-14	0.824	0.949
RPL34	3	-0.4928108	8.62E-48	1.57E-43	1	1
HINT1	3	-0.4939735	3.04E-32	5.55E-28	0.935	0.996
EID1	3	-0.4992316	6.75E-30	1.23E-25	0.876	0.988
SERPINA5	3	-0.500124	1.31E-20	2.38E-16	0.189	0.535
HIST1H4C	3	-0.5055421	2.19E-15	4.00E-11	0.394	0.66
MT-ND5	3	-0.5069291	4.30E-13	7.85E-09	0.603	0.773
RHOBTB3	3	-0.5121496	2.40E-15	4.38E-11	0.375	0.68
HIST1H1C	3	-0.5147056	2.18E-12	3.97E-08	0.264	0.512
ACAN	3	-0.5159284	7.95E-20	1.45E-15	0.857	0.965
HSPB1	3	-0.5189131	1.45E-21	2.65E-17	0.489	0.801
SOD3	3	-0.519496	1.48E-16	2.70E-12	0.967	0.996
PSAP	3	-0.5205507	2.40E-25	4.37E-21	0.883	0.977
PABPC1	3	-0.5227081	6.60E-25	1.21E-20	0.831	0.969
TCF4	3	-0.528934	1.48E-25	2.70E-21	0.186	0.602
EIF3L	3	-0.5324388	7.40E-29	1.35E-24	0.775	0.977
THY1	3	-0.5350563	3.85E-26	7.03E-22	0.081	0.465
MOXD1	3	-0.5453701	7.99E-23	1.46E-18	0.195	0.566
CTGF	3	-0.5479761	4.72E-17	8.61E-13	0.192	0.52
HNRNPA1	3	-0.5514958	2.05E-30	3.74E-26	0.971	1
RPL7	3	-0.5605293	2.48E-46	4.53E-42	1	1
PTRF	3	-0.5611599	8.51E-22	1.55E-17	0.306	0.648
ARHGAP29	3	-0.5612858	6.78E-20	1.24E-15	0.264	0.594
NPC2	3	-0.5719613	3.59E-39	6.54E-35	0.928	0.996
CEBPD	3	-0.5855643	1.24E-20	2.27E-16	0.814	0.941
EIF3H	3	-0.5881285	1.39E-36	2.53E-32	0.951	0.988
CCNI	3	-0.5900351	1.42E-45	2.60E-41	0.997	1
TPT1	3	-0.5959845	6.51E-56	1.19E-51	1	1
CP	3	-0.5980051	3.06E-30	5.58E-26	0.609	0.961

ISLR	3	-0.6064464	1.42E-32	2.59E-28	0.127	0.594
BGN	3	-0.6127605	5.65E-32	1.03E-27	0.925	1
RPL4	3	-0.6143371	5.68E-54	1.04E-49	1	1
EEF2	3	-0.6148909	6.86E-26	1.25E-21	0.99	0.992
SPARC	3	-0.6306205	8.99E-31	1.64E-26	0.13	0.582
P4HB	3	-0.6322406	4.68E-25	8.55E-21	0.537	0.883
VIM	3	-0.6369923	9.54E-38	1.74E-33	1	1
HLA-C	3	-0.6396752	8.22E-26	1.50E-21	0.795	0.961
H3F3A	3	-0.6438029	1.20E-40	2.18E-36	0.893	0.992
RPS8	3	-0.6509203	9.58E-66	1.75E-61	1	1
GADD45A	3	-0.6539036	4.17E-15	7.62E-11	0.365	0.656
TPM1	3	-0.6541351	1.02E-19	1.87E-15	0.453	0.746
FTH1	3	-0.6556321	4.81E-20	8.78E-16	1	1
RPL10A	3	-0.6573164	6.69E-57	1.22E-52	1	1
YBX3	3	-0.6603149	2.05E-35	3.74E-31	0.668	0.969
RPLP0	3	-0.6658396	1.58E-61	2.89E-57	1	1
SLPI	3	-0.6679375	7.96E-14	1.45E-09	0.951	1
S100A4	3	-0.6695544	2.38E-15	4.35E-11	0.629	0.867
PRUNE2	3	-0.6779954	1.11E-29	2.02E-25	0.208	0.645
EEF1A1	3	-0.6787017	3.39E-66	6.18E-62	1	1
MSMP	3	-0.6788438	8.12E-16	1.48E-11	0.088	0.363
S100A1	3	-0.6838578	3.21E-39	5.85E-35	0.977	0.996
BAMBI	3	-0.6875201	7.36E-12	1.34E-07	0.505	0.691
CRTAP	3	-0.687996	8.30E-38	1.51E-33	0.42	0.859
NNMT	3	-0.6883819	2.20E-34	4.01E-30	0.863	0.984
COL2A1	3	-0.6949448	2.61E-27	4.75E-23	0.42	0.824
PLAC9	3	-0.6991481	8.47E-33	1.55E-28	0.645	0.961
CYR61	3	-0.7215278	1.20E-19	2.19E-15	0.134	0.473
TSC22D1	3	-0.7490916	5.01E-25	9.14E-21	0.518	0.852
B2M	3	-0.7806325	3.19E-56	5.82E-52	0.993	1
C2orf82	3	-0.7928156	4.39E-27	8.02E-23	0.86	0.988
GPX3	3	-0.8259654	3.35E-43	6.11E-39	0.902	1
MT-ND4	3	-0.8460437	2.14E-38	3.91E-34	0.971	0.977
CAPS	3	-0.8502548	6.79E-37	1.24E-32	0.463	0.887
MT-CO3	3	-0.8810186	4.81E-31	8.78E-27	0.886	0.953
PAX1	3	-0.8822525	1.94E-43	3.54E-39	0.342	0.863
RPS20	3	-0.8989436	1.28E-80	2.33E-76	0.997	1
MT-ND1	3	-0.9084404	4.68E-36	8.53E-32	0.482	0.855
RPS11	3	-0.9273548	8.92E-79	1.63E-74	0.984	1

CCL2	3	-0.9315377	2.07E-14	3.78E-10	0.026	0.234
ACTG1	3	-0.9342111	4.49E-49	8.20E-45	0.899	0.992
MGP	3	-1.0304227	7.55E-40	1.38E-35	0.997	1
APOD	3	-1.041314	1.40E-27	2.55E-23	0.202	0.613
SPTSSB	3	-1.043607	1.12E-38	2.04E-34	0.01	0.465
MT-CO1	3	-1.0685374	1.23E-42	2.24E-38	0.971	0.98
ADIRF	3	-1.0704599	2.81E-51	5.13E-47	0.228	0.828
CLEC3A	3	-1.0727708	5.06E-41	9.23E-37	0.182	0.719
MT-ATP6	3	-1.0738792	9.66E-46	1.76E-41	0.798	0.945
MT-CO2	3	-1.0904068	1.19E-40	2.17E-36	0.935	0.98
NEAT1	3	-1.1047211	1.79E-55	3.27E-51	0.958	0.996
OGN	3	-1.1451567	3.43E-29	6.26E-25	0.681	0.918
MT-ND2	3	-1.1749745	5.87E-45	1.07E-40	0.56	0.895
MT-CYB	3	-1.2123513	7.37E-48	1.34E-43	0.609	0.926
MT-ND3	3	-1.2749634	3.59E-56	6.55E-52	0.567	0.93
ZFP36L2	3	-1.2784743	2.92E-46	5.33E-42	0.163	0.711
FRZB	3	-1.5815535	6.30E-43	1.15E-38	0.143	0.699
**gene**	**cluster**	**avg_logFC**	**p_val**	**p_val_adj**	**pct.1**	**pct.2**
PLA2G2A	4	1.72279508	3.48E-43	6.34E-39	0.841	0.28
MT1G	4	1.28050728	1.99E-18	3.64E-14	0.995	0.872
CHI3L2	4	1.27704128	2.47E-52	4.51E-48	0.914	0.412
TGFBI	4	1.24432252	3.95E-44	7.21E-40	0.777	0.243
EPYC	4	1.16667225	1.21E-39	2.21E-35	0.718	0.206
PRG4	4	1.14319155	3.05E-28	5.57E-24	0.518	0.098
SPP1	4	1.13662918	9.43E-45	1.72E-40	0.582	0.027
CYP1B1	4	1.02761942	1.53E-23	2.79E-19	0.559	0.179
MT1X	4	0.94071142	4.97E-24	9.08E-20	1	0.993
CTHRC1	4	0.93727732	9.18E-41	1.68E-36	0.605	0.078
PPP3CA	4	0.9349202	5.97E-31	1.09E-26	0.941	0.659
COMP	4	0.9180967	3.38E-30	6.17E-26	0.95	0.679
MT2A	4	0.91260638	4.47E-18	8.15E-14	1	0.993
MT1H	4	0.84292468	1.06E-07	0.00194048	0.736	0.557
COL1A2	4	0.76049002	1.80E-17	3.29E-13	0.655	0.351
SLC25A37	4	0.74568213	6.78E-30	1.24E-25	0.905	0.622
BTG1	4	0.70712459	3.58E-33	6.54E-29	0.986	0.949
NOVA1	4	0.67662017	1.40E-18	2.56E-14	0.918	0.74
SOD2	4	0.66404525	2.02E-13	3.68E-09	0.959	0.818
CTSD	4	0.65680894	2.04E-09	3.71E-05	0.995	0.959
SAT1	4	0.65600763	7.58E-21	1.38E-16	0.986	0.929

CYTL1	4	0.64917255	1.42E-19	2.60E-15	0.505	0.149
PTGS2	4	0.63276176	1.14E-19	2.08E-15	0.405	0.081
CHI3L1	4	0.62201206	5.48E-12	1.00E-07	0.977	0.929
VEGFA	4	0.55790285	2.72E-15	4.96E-11	0.705	0.47
FN1	4	0.54717564	5.66E-23	1.03E-18	1	1
CFD	4	0.52392522	8.06E-12	1.47E-07	0.314	0.081
CA9	4	0.51813481	4.76E-10	8.69E-06	0.564	0.351
ADAMTS6	4	0.51542111	3.41E-22	6.23E-18	0.409	0.064
CCDC85B	4	0.51164291	7.57E-14	1.38E-09	0.9	0.703
DDIT4L	4	0.50197031	7.91E-19	1.44E-14	0.614	0.25
PMP22	4	0.50114442	2.60E-12	4.74E-08	0.732	0.551
PPP1CB	4	0.49900184	1.58E-09	2.89E-05	0.85	0.733
MT1M	4	0.48897559	4.44E-12	8.10E-08	0.705	0.476
TNFRSF11B	4	0.48718772	1.24E-08	0.00022562	0.686	0.497
SORBS2	4	0.47759468	1.12E-06	0.02049236	0.286	0.125
COL11A1	4	0.47658269	8.07E-09	0.00014733	0.945	0.963
CD44	4	0.47196626	4.23E-15	7.71E-11	0.782	0.5
AEBP1	4	0.47062752	1.88E-15	3.42E-11	0.868	0.699
TIMP1	4	0.46080403	1.36E-06	0.02480794	0.995	1
SERPINE2	4	0.45705652	4.88E-07	0.00890387	1	0.976
HAPLN1	4	0.45492584	8.26E-11	1.51E-06	0.795	0.662
C7orf73	4	0.44595352	3.85E-15	7.02E-11	0.959	0.875
SCRG1	4	0.43853862	5.11E-09	9.33E-05	1	1
CLEC11A	4	0.43754519	6.88E-11	1.25E-06	0.65	0.419
TMSB4X	4	0.42858929	1.03E-11	1.87E-07	0.909	0.794
C4orf48	4	0.42626596	8.61E-11	1.57E-06	0.591	0.355
CCDC80	4	0.42303807	2.10E-11	3.83E-07	0.986	0.966
WWP2	4	0.41690751	4.29E-10	7.83E-06	0.723	0.591
SMOC2	4	0.41180124	7.75E-17	1.41E-12	0.391	0.091
BAMBI	4	0.40513128	6.76E-10	1.23E-05	0.768	0.568
RNF145	4	0.40046319	2.93E-10	5.34E-06	0.573	0.361
WTAP	4	0.39424925	1.33E-10	2.43E-06	0.741	0.544
MT1F	4	0.39244093	1.19E-08	0.00021633	0.973	0.885
PDE4B	4	0.38347356	4.05E-18	7.40E-14	0.305	0.03
SRGN	4	0.37548095	1.26E-09	2.30E-05	0.582	0.355
IFITM10	4	0.37361295	2.27E-11	4.13E-07	0.645	0.385
RABGGTB	4	0.36683067	7.67E-09	0.00013989	0.6	0.419
HIF1A	4	0.3636777	1.07E-10	1.96E-06	0.955	0.905
BZW1	4	0.36054342	5.03E-09	9.18E-05	0.645	0.463

BAALC	4	0.35775301	2.45E-09	4.46E-05	0.582	0.358
CRIP1	4	0.35754091	5.73E-14	1.05E-09	0.7	0.361
FKBP9	4	0.34858527	2.49E-12	4.54E-08	0.445	0.172
TSHZ2	4	0.33569788	1.08E-17	1.96E-13	0.268	0.017
LTBP1	4	0.31927715	2.11E-21	3.85E-17	0.345	0.03
FOSL1	4	0.31891982	7.69E-13	1.40E-08	0.25	0.037
WSB1	4	0.31830439	1.78E-08	0.00032421	0.9	0.841
CRLF1	4	0.31498535	2.14E-11	3.90E-07	0.482	0.213
PHLDA1	4	0.3149695	2.27E-06	0.04134883	0.227	0.084
PAM	4	0.30953618	7.11E-10	1.30E-05	0.864	0.75
DDIT3	4	0.30260852	1.00E-06	0.01827282	0.536	0.361
BMP2	4	0.29999455	1.81E-09	3.31E-05	0.182	0.027
C11orf31	4	0.29977605	9.60E-09	0.00017517	0.8	0.689
EVI2A	4	0.29352163	7.51E-13	1.37E-08	0.268	0.047
COX17	4	0.28659424	5.58E-07	0.01018838	0.818	0.733
CCDC151	4	0.2812961	2.02E-06	0.03690321	0.545	0.385
BIRC2	4	0.27091121	1.87E-06	0.03410422	0.645	0.483
ANXA1	4	-0.2501837	6.74E-07	0.01230298	0.686	0.824
ZNF90	4	-0.2517189	4.69E-07	0.00855967	0.486	0.676
DPYSL3	4	-0.2520024	5.21E-09	9.51E-05	0.168	0.402
RPS15A	4	-0.2520882	3.31E-24	6.04E-20	1	1
CHMP2A	4	-0.2520909	1.87E-07	0.00341164	0.418	0.611
HEBP1	4	-0.2545033	9.59E-09	0.00017495	0.25	0.486
DNAJC1	4	-0.254511	1.59E-08	0.00029087	0.155	0.372
FAU	4	-0.2545489	7.99E-17	1.46E-12	1	1
SF1	4	-0.2564281	1.07E-07	0.00195819	0.373	0.584
MZT2A	4	-0.2564281	3.74E-08	0.00068175	0.732	0.878
KCNQ1OT1	4	-0.2571947	1.67E-06	0.03039441	0.2	0.382
CHID1	4	-0.2577319	3.09E-07	0.00563381	0.536	0.723
UQCRHL	4	-0.258992	1.27E-09	2.32E-05	0.355	0.611
RGS10	4	-0.2590671	1.48E-08	0.00027026	0.264	0.514
RPL26	4	-0.2598436	1.63E-20	2.98E-16	1	1
P3H2	4	-0.2606971	1.82E-06	0.03316826	0.277	0.47
SLC25A5	4	-0.2620071	2.43E-09	4.44E-05	0.309	0.578
NDUFC2	4	-0.2625699	4.39E-07	0.00800582	0.605	0.78
SLC44A1	4	-0.2628839	2.01E-06	0.03671436	0.355	0.537
TXN	4	-0.2635461	1.16E-07	0.00211118	0.791	0.912
NPDC1	4	-0.2639738	1.34E-07	0.00244188	0.182	0.378
PLXDC2	4	-0.2643258	7.19E-07	0.01311453	0.505	0.679

NUCB2	4	-0.2646036	1.47E-06	0.02684801	0.341	0.527
RPS12	4	-0.2646204	5.42E-21	9.88E-17	1	1
RNH1	4	-0.2651633	1.42E-06	0.02582481	0.327	0.507
EID1	4	-0.2661945	1.67E-10	3.04E-06	0.945	0.986
EEF1D	4	-0.2666428	1.57E-13	2.87E-09	0.991	1
BEX2	4	-0.2669572	2.23E-08	0.00040614	0.205	0.432
ARPC1A	4	-0.2678467	1.04E-08	0.00019056	0.545	0.75
BNIP3L	4	-0.2681106	1.52E-08	0.00027773	0.768	0.909
TENM3	4	-0.2682942	3.82E-12	6.98E-08	0.068	0.321
RPL28	4	-0.2684607	5.57E-24	1.02E-19	1	1
RPL3	4	-0.2690076	5.68E-21	1.04E-16	1	1
C14orf1	4	-0.2705762	7.56E-07	0.01379216	0.286	0.473
PDIA6	4	-0.2713102	3.15E-07	0.00574942	0.7	0.868
UQCRH	4	-0.2716997	1.77E-11	3.24E-07	0.964	0.983
HOXB6	4	-0.2732369	4.35E-07	0.00793215	0.309	0.497
RABAC1	4	-0.2733028	5.35E-11	9.77E-07	0.818	0.949
RPS25	4	-0.2734299	2.58E-24	4.71E-20	1	1
PPIA	4	-0.2734402	4.74E-10	8.64E-06	0.945	0.997
RWDD1	4	-0.2741767	2.26E-07	0.00411599	0.645	0.774
HADHA	4	-0.2746104	2.69E-08	0.00049003	0.336	0.568
RP11-71N10.1	4	-0.2751148	1.86E-08	0.00033997	0.159	0.378
UBB	4	-0.2754384	3.74E-08	0.00068154	0.709	0.895
RPL27A	4	-0.2758275	3.85E-25	7.03E-21	1	1
RPL36AL	4	-0.2758668	2.03E-14	3.70E-10	0.995	1
ATP5G2	4	-0.2767568	5.50E-11	1.00E-06	0.982	0.993
RPL24	4	-0.277097	8.65E-23	1.58E-18	1	1
RPS9	4	-0.2771242	3.26E-25	5.95E-21	1	1
TUBB4B	4	-0.2772468	7.28E-07	0.01328982	0.323	0.517
ATP5L	4	-0.2776317	1.35E-10	2.46E-06	0.932	0.97
MPP6	4	-0.2797598	1.25E-10	2.28E-06	0.214	0.48
GNB2L1	4	-0.2805237	6.68E-16	1.22E-11	0.995	0.997
KRTCAP2	4	-0.2811659	7.70E-09	0.00014051	0.655	0.851
PRKCSH	4	-0.2821559	5.03E-08	0.00091835	0.291	0.514
NFIC	4	-0.2823967	1.32E-08	0.00024174	0.391	0.615
HSP90AB1	4	-0.282471	1.35E-07	0.00246441	0.877	0.946
MALAT1	4	-0.2826625	5.53E-08	0.00100996	1	1
RPL11	4	-0.2834777	3.45E-26	6.29E-22	1	1
GPC1	4	-0.2837421	2.32E-13	4.24E-09	0.1	0.385
ARPC2	4	-0.2843579	6.88E-07	0.01255237	0.714	0.831

PCBP2	4	-0.286582	1.62E-11	2.95E-07	0.941	0.983
C19orf43	4	-0.2882017	2.42E-10	4.41E-06	0.764	0.882
NFIA	4	-0.2883916	2.72E-08	0.00049581	0.432	0.642
RPS21	4	-0.2885217	7.21E-23	1.32E-18	1	1
RPS6	4	-0.2888516	2.03E-29	3.70E-25	1	1
RPL35	4	-0.2891312	8.61E-28	1.57E-23	1	1
CIRBP	4	-0.2908788	1.70E-09	3.10E-05	0.968	0.98
RPL22	4	-0.290942	1.95E-21	3.56E-17	1	1
RPS2	4	-0.2909991	2.34E-26	4.27E-22	1	1
EIF2A	4	-0.2913011	9.45E-07	0.0172367	0.586	0.747
AP2M1	4	-0.2936222	1.61E-09	2.93E-05	0.455	0.709
EIF3F	4	-0.2940839	1.26E-08	0.00022982	0.736	0.889
ZFHX4	4	-0.2947653	3.64E-11	6.64E-07	0.2	0.47
ATP5A1	4	-0.2947826	2.87E-08	0.00052375	0.455	0.655
FBL	4	-0.2951239	2.63E-08	0.00047909	0.355	0.571
EIF1	4	-0.2955972	1.42E-15	2.59E-11	1	1
RPL27	4	-0.2973367	3.84E-22	7.00E-18	1	1
SRP14	4	-0.2984044	1.93E-14	3.53E-10	0.982	0.997
RPS7	4	-0.3007548	7.94E-24	1.45E-19	1	1
RPL13	4	-0.3008355	2.70E-33	4.93E-29	1	1
HSPD1	4	-0.3014175	3.90E-09	7.12E-05	0.295	0.551
HOXB8	4	-0.3019244	1.53E-10	2.78E-06	0.109	0.345
ISM1	4	-0.3019551	3.86E-10	7.05E-06	0.232	0.497
CREG1	4	-0.3024277	4.38E-10	8.00E-06	0.355	0.625
REEP5	4	-0.3034248	4.70E-11	8.58E-07	0.414	0.709
SQLE	4	-0.3036609	7.24E-09	0.0001322	0.136	0.355
RPL29	4	-0.3057791	1.12E-27	2.04E-23	1	1
NACA	4	-0.3059923	3.20E-19	5.84E-15	1	1
VIM	4	-0.3061186	1.06E-10	1.93E-06	1	1
PFN1	4	-0.3082919	8.58E-10	1.57E-05	0.877	0.959
RARRES3	4	-0.3097719	2.56E-11	4.67E-07	0.114	0.365
RAC1	4	-0.3100997	1.25E-11	2.29E-07	0.827	0.939
HDLBP	4	-0.3124689	8.11E-08	0.00147912	0.523	0.662
TOMM7	4	-0.3143676	7.81E-25	1.43E-20	1	1
HNRNPA1	4	-0.3143899	4.75E-10	8.67E-06	0.977	0.997
ITGA11	4	-0.3149999	4.71E-12	8.60E-08	0.095	0.355
RPL10	4	-0.3152705	2.81E-33	5.12E-29	1	1
TBL1XR1	4	-0.3159429	2.73E-06	0.04976411	0.586	0.75
CCDC8	4	-0.3167085	1.42E-11	2.59E-07	0.118	0.378

MT-ND5	4	-0.3167867	4.51E-08	0.00082349	0.586	0.78
EDF1	4	-0.3177972	6.80E-16	1.24E-11	0.941	0.986
GADD45B	4	-0.3179146	8.61E-07	0.01571432	0.291	0.503
PRDX6	4	-0.318401	3.37E-10	6.16E-06	0.659	0.851
RPS5	4	-0.3207004	1.28E-27	2.34E-23	1	1
PKM	4	-0.3220426	5.51E-11	1.01E-06	0.895	0.953
NPM1	4	-0.3236013	5.51E-10	1.01E-05	0.973	0.993
RPS23	4	-0.3253018	3.89E-33	7.10E-29	1	1
PTRF	4	-0.3260116	6.92E-10	1.26E-05	0.591	0.801
GLTSCR2	4	-0.3283798	8.74E-11	1.59E-06	0.877	0.953
PSAP	4	-0.328974	5.62E-12	1.02E-07	0.886	0.956
LDHB	4	-0.3293631	4.28E-11	7.81E-07	0.586	0.855
RPL18A	4	-0.3295418	3.18E-33	5.79E-29	1	1
ATP5H	4	-0.3300644	7.62E-11	1.39E-06	0.509	0.76
ID4	4	-0.3301852	1.22E-08	0.00022234	0.705	0.872
EIF3D	4	-0.331419	6.18E-10	1.13E-05	0.541	0.77
RPS19	4	-0.3323246	9.02E-33	1.65E-28	1	1
LGALS1	4	-0.3331364	1.89E-10	3.45E-06	0.991	0.993
C14orf166	4	-0.3337881	8.30E-12	1.51E-07	0.627	0.834
UBA52	4	-0.3342208	3.63E-33	6.63E-29	1	1
COL14A1	4	-0.3344485	3.62E-10	6.61E-06	0.05	0.257
CTGF	4	-0.3364656	1.94E-07	0.00354721	0.836	0.97
CD63	4	-0.3385182	3.71E-11	6.77E-07	0.995	1
PABPC1	4	-0.3396137	4.99E-12	9.11E-08	0.891	0.97
RPS18	4	-0.3398235	1.31E-35	2.39E-31	1	1
FTL	4	-0.3406741	7.99E-21	1.46E-16	1	1
SLC25A3	4	-0.3412489	7.09E-13	1.29E-08	0.782	0.949
ITM2A	4	-0.3427464	3.34E-08	0.00061027	0.568	0.76
MSMO1	4	-0.3443856	1.01E-07	0.00184495	0.227	0.429
NINJ1	4	-0.3457559	3.51E-10	6.41E-06	0.555	0.743
GSTP1	4	-0.3473717	8.12E-12	1.48E-07	0.918	0.966
RPL21	4	-0.3484704	9.03E-44	1.65E-39	1	1
COX7C	4	-0.3487182	2.06E-24	3.77E-20	1	1
B2M	4	-0.3487914	2.79E-17	5.10E-13	0.995	1
KHDRBS3	4	-0.3526799	1.35E-16	2.47E-12	0.009	0.284
RPL35A	4	-0.356493	6.05E-35	1.10E-30	1	1
RPS27	4	-0.3570262	9.06E-36	1.65E-31	1	1
RPL31	4	-0.3587749	1.48E-32	2.69E-28	1	1
PLP2	4	-0.3597979	3.97E-12	7.24E-08	0.514	0.801

MGP	4	-0.360526	3.18E-09	5.79E-05	1	1
DMKN	4	-0.3605905	1.76E-12	3.21E-08	0.395	0.669
CST3	4	-0.3625401	4.27E-14	7.79E-10	0.991	1
NGFRAP1	4	-0.3628153	6.12E-15	1.12E-10	0.932	0.98
IGFBP7	4	-0.3656961	4.07E-08	0.00074228	0.932	0.946
SEC11A	4	-0.3662782	5.80E-16	1.06E-11	0.814	0.946
MOXD1	4	-0.3670014	1.08E-09	1.97E-05	0.223	0.453
NAP1L1	4	-0.3690003	7.60E-14	1.39E-09	0.914	0.956
RPSA	4	-0.3690855	5.96E-25	1.09E-20	1	1
TSPO	4	-0.3700776	7.28E-15	1.33E-10	0.786	0.929
PEBP1	4	-0.3740143	3.61E-16	6.58E-12	0.727	0.953
CPE	4	-0.3742311	7.63E-13	1.39E-08	0.5	0.794
HOXC10	4	-0.3744364	2.94E-12	5.36E-08	0.523	0.76
RPL8	4	-0.3754563	1.22E-42	2.23E-38	1	1
CALM3	4	-0.3764498	6.38E-12	1.16E-07	0.545	0.791
ERGIC3	4	-0.3772454	7.65E-15	1.40E-10	0.795	0.939
RPL37	4	-0.3779345	5.32E-37	9.70E-33	1	1
AFF3	4	-0.378266	3.74E-15	6.83E-11	0.195	0.541
RPL19	4	-0.3798681	5.54E-41	1.01E-36	1	1
PDE4DIP	4	-0.3799765	8.47E-13	1.55E-08	0.459	0.709
SLC25A6	4	-0.3803636	1.16E-20	2.11E-16	0.964	0.997
YBX3	4	-0.3840504	4.00E-15	7.30E-11	0.745	0.939
SPTLC3	4	-0.3891636	2.64E-07	0.00481184	0.286	0.5
CAV1	4	-0.3892614	6.06E-13	1.11E-08	0.573	0.807
RPL13A	4	-0.390582	2.95E-47	5.39E-43	1	1
HIF1A-AS2	4	-0.391369	5.76E-08	0.00105022	0.264	0.47
RPS29	4	-0.3918228	4.34E-42	7.91E-38	1	1
RPL4	4	-0.3920062	2.40E-29	4.39E-25	1	1
ARL2BP	4	-0.3925217	1.62E-14	2.95E-10	0.505	0.77
RHOA	4	-0.3946807	1.66E-14	3.02E-10	0.718	0.916
SLC14A1	4	-0.3964153	4.30E-13	7.85E-09	0.318	0.622
ARHGAP29	4	-0.3977131	2.52E-11	4.61E-07	0.427	0.689
CXXC5	4	-0.4017344	5.40E-12	9.85E-08	0.459	0.689
RPL9	4	-0.406068	1.71E-41	3.12E-37	1	1
TSC22D1	4	-0.407948	7.89E-08	0.00143937	0.673	0.845
SSR2	4	-0.4106713	9.81E-19	1.79E-14	0.777	0.943
LPP	4	-0.4115671	1.91E-09	3.49E-05	0.45	0.682
TPT1	4	-0.4127313	9.75E-38	1.78E-33	1	1
KDELR2	4	-0.4133019	3.08E-15	5.62E-11	0.791	0.956

AHNAK2	4	-0.4154635	4.28E-12	7.81E-08	0.236	0.507
RPL14	4	-0.4170346	1.33E-37	2.42E-33	1	1
EIF3L	4	-0.4181629	7.36E-19	1.34E-14	0.814	0.99
RPS3A	4	-0.4185924	7.23E-47	1.32E-42	1	1
PSMA7	4	-0.4198082	7.59E-14	1.38E-09	0.614	0.848
RPL10A	4	-0.4213892	2.78E-37	5.07E-33	1	1
C2orf82	4	-0.4215395	1.92E-13	3.50E-09	0.955	1
MYL12B	4	-0.4242116	8.84E-16	1.61E-11	0.509	0.787
PLA2G16	4	-0.4292051	1.60E-14	2.93E-10	0.209	0.514
ISLR	4	-0.4305624	8.42E-12	1.54E-07	0.582	0.801
ST13	4	-0.4308906	5.74E-19	1.05E-14	0.895	0.98
UMAD1	4	-0.4316189	7.74E-12	1.41E-07	0.341	0.581
NKX3-2	4	-0.4344289	1.61E-13	2.94E-09	0.159	0.459
DAB2	4	-0.434909	3.34E-10	6.10E-06	0.418	0.625
EIF3K	4	-0.4352275	6.21E-18	1.13E-13	0.7	0.909
RPS13	4	-0.4371232	1.19E-43	2.18E-39	1	1
DSEL	4	-0.4372373	3.09E-07	0.00563676	0.295	0.48
BTF3	4	-0.4385305	4.80E-28	8.77E-24	1	1
RPL23	4	-0.4421603	4.10E-37	7.49E-33	0.995	1
RPL23A	4	-0.4425396	1.29E-46	2.35E-42	1	1
CCNI	4	-0.4429762	3.32E-26	6.06E-22	0.991	1
RPS17	4	-0.446854	4.41E-47	8.04E-43	1	1
RPLP2	4	-0.4470842	3.19E-53	5.82E-49	1	1
RPL34	4	-0.4487295	7.03E-51	1.28E-46	1	1
EEF1B2	4	-0.4529687	1.65E-24	3.01E-20	0.973	1
MYLK	4	-0.4538399	7.74E-13	1.41E-08	0.314	0.595
RPS27A	4	-0.4565797	1.82E-48	3.32E-44	1	1
HLA-C	4	-0.4616976	1.11E-16	2.03E-12	0.814	0.966
CALM1	4	-0.4641953	1.26E-12	2.30E-08	0.432	0.699
UQCRB	4	-0.4672016	2.14E-26	3.91E-22	0.959	0.99
HLA-B	4	-0.4692851	4.41E-18	8.05E-14	0.736	0.939
MT-CO2	4	-0.4705628	5.21E-21	9.50E-17	0.977	0.98
RPS8	4	-0.4705716	6.03E-49	1.10E-44	1	1
SSR3	4	-0.4775886	2.90E-20	5.29E-16	0.786	0.963
ATP5O	4	-0.4780526	3.08E-21	5.62E-17	0.641	0.902
ACTB	4	-0.4796401	6.45E-16	1.18E-11	0.845	0.966
RPL30	4	-0.4832956	2.79E-47	5.09E-43	1	1
HINT1	4	-0.4849371	1.08E-33	1.98E-29	0.964	1
MT-ND4	4	-0.4855809	1.56E-20	2.84E-16	0.986	0.973

CRIP2	4	-0.4860274	8.53E-16	1.56E-11	0.382	0.672
SPINT2	4	-0.4957646	8.93E-18	1.63E-13	0.223	0.588
NEAT1	4	-0.5024424	6.05E-16	1.10E-11	0.991	1
RPL7	4	-0.5085314	9.08E-45	1.66E-40	1	1
TUBB2B	4	-0.5116743	6.41E-09	0.00011702	0.55	0.747
MAGED1	4	-0.5129766	1.02E-06	0.01857691	0.368	0.554
MYL6	4	-0.5144393	9.29E-29	1.69E-24	0.932	0.983
RPS16	4	-0.5180298	2.18E-51	3.98E-47	1	1
EIF4B	4	-0.5232577	6.95E-20	1.27E-15	0.691	0.895
TCF4	4	-0.523282	2.00E-16	3.65E-12	0.541	0.774
ZFAS1	4	-0.524824	2.57E-22	4.68E-18	0.968	0.993
ATRNL1	4	-0.5263013	2.15E-22	3.91E-18	0.232	0.652
DCN	4	-0.5304061	1.11E-27	2.03E-23	1	1
LINC01503	4	-0.5306648	1.61E-11	2.94E-07	0.505	0.733
CRYAB	4	-0.5398239	5.54E-15	1.01E-10	0.895	0.966
NPC2	4	-0.5400468	7.84E-32	1.43E-27	0.982	0.997
SDC2	4	-0.5402974	2.64E-21	4.82E-17	0.864	0.963
EEF2	4	-0.5425338	2.76E-25	5.04E-21	0.991	1
CRTAP	4	-0.5425481	2.77E-27	5.05E-23	0.668	0.916
FBXO2	4	-0.5459077	4.47E-19	8.16E-15	0.805	0.936
INSIG1	4	-0.5461195	2.43E-12	4.43E-08	0.286	0.568
HIST1H1C	4	-0.5574461	3.49E-13	6.38E-09	0.177	0.459
RPL18	4	-0.5732253	1.53E-57	2.80E-53	1	1
TUBB	4	-0.5802107	2.12E-21	3.88E-17	0.714	0.926
APOD	4	-0.586284	4.16E-15	7.58E-11	0.173	0.486
PAX1	4	-0.5971622	3.60E-24	6.57E-20	0.673	0.905
HLA-A	4	-0.6095085	1.37E-25	2.49E-21	0.786	0.966
MT-CO3	4	-0.6102879	3.71E-30	6.78E-26	0.977	0.956
GREM1	4	-0.6131894	6.11E-10	1.12E-05	0.077	0.294
EEF1A1	4	-0.6149488	2.98E-56	5.43E-52	1	1
RPLP0	4	-0.6163979	7.04E-65	1.28E-60	1	1
CITED2	4	-0.6195588	4.27E-16	7.79E-12	0.477	0.774
MT-ND1	4	-0.6329123	5.94E-24	1.08E-19	0.745	0.912
OGN	4	-0.6520344	5.44E-20	9.92E-16	1	1
TPM1	4	-0.662913	3.63E-16	6.63E-12	0.65	0.865
OSBPL9	4	-0.6696352	1.62E-26	2.96E-22	0.277	0.689
MT-CO1	4	-0.6917164	3.11E-30	5.67E-26	0.977	0.966
CLEC3A	4	-0.6969906	1.23E-16	2.24E-12	0.409	0.77
MT-ATP6	4	-0.7164844	2.02E-27	3.68E-23	0.936	0.949

RGCC	4	-0.7224965	4.35E-11	7.95E-07	0.723	0.851
MSMP	4	-0.7400347	4.19E-10	7.64E-06	0.077	0.297
PLPP1	4	-0.741753	1.27E-26	2.31E-22	0.4	0.794
ACTG1	4	-0.7541412	1.30E-32	2.38E-28	0.864	0.986
THY1	4	-0.7585601	2.19E-21	4.00E-17	0.7	0.892
CD9	4	-0.7625193	4.48E-26	8.17E-22	0.386	0.787
CCDC102B	4	-0.766985	8.80E-38	1.61E-33	0.095	0.652
MT-ND2	4	-0.7723303	3.42E-25	6.24E-21	0.782	0.916
EIF3H	4	-0.7743928	2.37E-49	4.33E-45	0.9	1
MT-ND3	4	-0.8252386	1.38E-38	2.52E-34	0.855	0.932
FHL1	4	-0.8439919	3.33E-27	6.07E-23	0.545	0.841
FTH1	4	-0.8493686	5.66E-57	1.03E-52	1	1
XAGE2	4	-0.8711722	3.63E-15	6.62E-11	0.014	0.27
CRISPLD1	4	-0.8775357	4.07E-27	7.43E-23	0.105	0.551
MT-CYB	4	-0.8815357	4.75E-35	8.68E-31	0.855	0.946
ZFP36L2	4	-0.8913423	5.24E-24	9.57E-20	0.291	0.682
RPS11	4	-0.909619	5.02E-72	9.15E-68	0.995	1
SLC40A1	4	-0.9836826	8.79E-24	1.60E-19	0.264	0.676
ADIRF	4	-1.0168031	5.86E-35	1.07E-30	0.3	0.794
RPS20	4	-1.0318771	1.33E-76	2.42E-72	0.995	1
CAPS	4	-1.092489	1.28E-41	2.33E-37	0.732	0.993
FRZB	4	-1.1687865	1.72E-25	3.14E-21	0.336	0.743
SPTSSB	4	-1.215181	7.08E-38	1.29E-33	0.018	0.568
**gene**	**cluster**	**avg_logFC**	**p_val**	**p_val_adj**	**pct.1**	**pct.2**
SPP1	5	2.20204647	5.54E-55	1.01E-50	0.743	0.047
PRG4	5	1.56580969	9.15E-40	1.67E-35	0.555	0.016
COMP	5	1.46276481	2.08E-46	3.79E-42	0.991	0.559
PLA2G2A	5	1.31326096	9.62E-31	1.76E-26	0.927	0.645
CHI3L2	5	1.25618701	6.17E-42	1.13E-37	0.968	0.617
SERPINE2	5	1.14660246	2.28E-32	4.16E-28	1	0.977
VEGFA	5	1.13669235	3.17E-43	5.79E-39	0.862	0.316
MT1G	5	1.13363874	8.10E-29	1.48E-24	0.977	0.91
MT1H	5	1.07823018	6.75E-16	1.23E-11	0.789	0.539
PTGS2	5	1.06780844	6.46E-34	1.18E-29	0.587	0.086
MT2A	5	1.01222777	1.08E-19	1.97E-15	1	0.996
TGFBI	5	0.96963357	1.54E-43	2.80E-39	0.67	0.066
DDIT4L	5	0.96775159	7.84E-29	1.43E-24	0.917	0.57
EPYC	5	0.91525966	3.37E-31	6.15E-27	0.573	0.094
ADM	5	0.87543189	5.18E-18	9.45E-14	0.803	0.523

FN1	5	0.86803988	7.02E-44	1.28E-39	1	0.992
S100A2	5	0.86149243	1.73E-19	3.16E-15	0.569	0.203
CTSD	5	0.83951476	2.01E-16	3.67E-12	0.986	0.961
CCDC85B	5	0.82703883	1.06E-23	1.94E-19	0.936	0.695
MT1X	5	0.80460703	1.28E-18	2.33E-14	1	0.992
CHI3L1	5	0.80090189	7.68E-19	1.40E-14	1	0.961
STK17A	5	0.78814491	2.80E-19	5.10E-15	0.459	0.113
SOD2	5	0.78688191	6.61E-20	1.21E-15	0.991	0.914
CTHRC1	5	0.78392387	1.83E-32	3.34E-28	0.541	0.059
PPP3CA	5	0.78104318	1.25E-29	2.29E-25	0.963	0.672
C2orf40	5	0.73571698	7.68E-25	1.40E-20	1	1
MRPS6	5	0.73275163	1.97E-14	3.60E-10	0.972	0.906
BMP2	5	0.73106254	2.15E-21	3.92E-17	0.367	0.031
ORM1	5	0.72912608	2.21E-07	0.00402701	0.427	0.215
TMEM45A	5	0.7199251	9.83E-24	1.79E-19	0.95	0.801
HILPDA	5	0.71914852	5.89E-16	1.07E-11	0.606	0.301
IGFBP3	5	0.7118593	1.59E-16	2.91E-12	0.344	0.055
TNFRSF12A	5	0.70844126	3.82E-13	6.97E-09	0.413	0.145
CA9	5	0.69454165	1.35E-23	2.47E-19	0.578	0.176
WTAP	5	0.6917737	3.48E-19	6.35E-15	0.839	0.547
DDIT3	5	0.69156973	4.37E-16	7.97E-12	0.679	0.367
GAPDH	5	0.6831234	9.40E-16	1.72E-11	1	1
UPP1	5	0.66800537	1.70E-20	3.10E-16	0.472	0.109
FAM162A	5	0.65832493	7.48E-28	1.36E-23	0.982	0.836
ERO1A	5	0.65733031	1.23E-18	2.25E-14	0.693	0.398
MT1F	5	0.64608804	1.58E-13	2.89E-09	0.959	0.883
TIMP1	5	0.6350791	1.78E-07	0.00324245	1	1
SLC25A37	5	0.61864362	4.99E-18	9.10E-14	0.862	0.645
ARRDC3	5	0.6134442	2.83E-13	5.16E-09	0.56	0.277
AK2	5	0.60614052	8.46E-16	1.54E-11	0.784	0.598
FOSL1	5	0.60497516	4.88E-17	8.91E-13	0.289	0.02
WWP2	5	0.59372082	3.81E-18	6.96E-14	0.858	0.613
BAALC	5	0.58981723	5.01E-21	9.13E-17	0.61	0.215
PPP1CB	5	0.56028237	2.31E-15	4.22E-11	0.904	0.699
PHLDA1	5	0.55903002	3.19E-18	5.82E-14	0.39	0.066
CTSL	5	0.55784869	1.60E-06	0.02928454	0.628	0.465
ANGPTL7	5	0.55583812	3.99E-12	7.27E-08	0.211	0.016
EGR1	5	0.55057432	2.46E-11	4.48E-07	0.408	0.156
PTP4A1	5	0.54779835	5.58E-11	1.02E-06	0.633	0.422

BZW1	5	0.54573862	1.49E-13	2.72E-09	0.628	0.371
DNER	5	0.53456592	7.08E-16	1.29E-11	0.633	0.309
CYTL1	5	0.53188543	1.53E-09	2.80E-05	0.312	0.102
RGCC	5	0.53059087	1.02E-10	1.86E-06	0.729	0.523
SLC3A2	5	0.52586101	1.14E-08	0.00020778	0.491	0.281
PMP22	5	0.52184164	1.02E-13	1.86E-09	0.826	0.641
PDPN	5	0.52079179	2.28E-14	4.16E-10	0.679	0.352
BTG1	5	0.51254013	1.37E-14	2.51E-10	0.995	0.988
AEBP1	5	0.50760729	2.43E-13	4.43E-09	0.812	0.621
RNF145	5	0.50084239	2.45E-15	4.48E-11	0.592	0.281
ENO2	5	0.49640443	1.83E-14	3.34E-10	0.624	0.344
C4orf48	5	0.49051008	6.00E-20	1.09E-15	0.495	0.121
HLA-DRB1	5	0.48893164	2.40E-14	4.38E-10	0.495	0.195
PTGDS	5	0.48372409	1.39E-10	2.54E-06	0.161	0.004
CCDC80	5	0.4801615	2.84E-12	5.18E-08	0.995	0.973
PDE4B	5	0.47947401	1.72E-21	3.14E-17	0.394	0.043
C11orf96	5	0.46869215	9.71E-11	1.77E-06	0.294	0.074
BHLHE40	5	0.45727603	1.16E-08	0.00021147	0.518	0.293
ARF6	5	0.45462622	7.88E-12	1.44E-07	0.628	0.406
CLK1	5	0.45331496	1.22E-14	2.23E-10	0.67	0.363
TREM1	5	0.45224795	1.36E-22	2.49E-18	0.33	0.004
GBE1	5	0.44935997	2.08E-09	3.80E-05	0.72	0.5
CRLF1	5	0.44745163	1.42E-19	2.59E-15	0.399	0.059
PPFIBP1	5	0.44640942	2.19E-12	3.99E-08	0.601	0.336
EIF4EBP1	5	0.4452656	7.19E-07	0.01312308	0.532	0.371
EGLN3	5	0.44506956	7.55E-12	1.38E-07	0.413	0.141
SLC2A1	5	0.43930717	2.02E-10	3.68E-06	0.394	0.156
ADAMTS6	5	0.43731462	7.39E-12	1.35E-07	0.275	0.051
CDK5RAP2	5	0.43070952	7.98E-08	0.00145527	0.326	0.137
SPRY2	5	0.43001532	3.64E-15	6.65E-11	0.349	0.066
ISG20	5	0.42778375	4.02E-09	7.33E-05	0.615	0.383
INSIG2	5	0.4261906	1.68E-09	3.07E-05	0.711	0.531
TPD52	5	0.42515022	4.42E-13	8.06E-09	0.853	0.68
ATP6V1G1	5	0.42242998	2.34E-19	4.26E-15	0.995	0.969
ZNF385D	5	0.41740223	6.78E-12	1.24E-07	0.711	0.461
CRTAC1	5	0.41733234	1.68E-19	3.07E-15	0.385	0.055
DNAJB9	5	0.41295092	1.69E-08	0.00030911	0.748	0.598
NDRG1	5	0.41227806	1.22E-08	0.00022301	0.573	0.363
IER3	5	0.41199681	3.01E-09	5.50E-05	0.275	0.074

NOVA1	5	0.40644717	3.08E-08	0.00056142	0.945	0.898
CFD	5	0.39737016	1.04E-07	0.00189481	0.349	0.137
SGIP1	5	0.39543148	3.54E-20	6.47E-16	0.298	0.004
VAPA	5	0.39209691	4.42E-13	8.07E-09	0.931	0.867
IFITM3	5	0.38857117	5.11E-11	9.32E-07	0.95	0.832
NAMPT	5	0.38811267	2.79E-12	5.10E-08	0.665	0.383
ERRFI1	5	0.38699551	5.56E-08	0.00101438	0.376	0.168
GPX1	5	0.38660758	6.82E-12	1.25E-07	0.908	0.781
MTHFD2	5	0.38558805	3.35E-10	6.11E-06	0.78	0.57
STK26	5	0.38552421	1.86E-10	3.39E-06	0.509	0.254
SRGN	5	0.38503732	3.83E-12	6.99E-08	0.463	0.18
MIF	5	0.38095178	2.94E-20	5.36E-16	1	1
RAB1A	5	0.38011535	2.41E-09	4.39E-05	0.835	0.73
RNMT	5	0.37518657	3.78E-10	6.89E-06	0.564	0.32
NRP2	5	0.37483037	5.52E-15	1.01E-10	0.307	0.043
ABI3BP	5	0.37033045	4.10E-10	7.48E-06	0.569	0.301
IFRD1	5	0.36906405	1.94E-07	0.00353443	0.67	0.523
RP11-125O18.1	5	0.36897942	9.67E-13	1.76E-08	0.358	0.094
PPP1R15A	5	0.36872883	8.84E-10	1.61E-05	0.399	0.168
WDR45B	5	0.36737719	2.16E-08	0.00039493	0.459	0.258
SLC25A36	5	0.36405735	2.08E-10	3.80E-06	0.706	0.453
CSGALNACT2	5	0.36104276	7.38E-09	0.00013466	0.349	0.145
NT5C3A	5	0.36045258	2.35E-08	0.00042889	0.376	0.172
EMP3	5	0.35817679	1.51E-10	2.76E-06	0.927	0.785
GLRX	5	0.35297516	1.11E-08	0.0002032	0.417	0.191
AVPI1	5	0.35090912	2.68E-08	0.00048976	0.399	0.188
SLC39A8	5	0.34789392	3.35E-10	6.11E-06	0.514	0.262
NPR3	5	0.34235268	1.36E-15	2.48E-11	0.225	0
XBP1	5	0.33716585	1.03E-06	0.01875627	0.716	0.59
TSHZ2	5	0.33621588	5.69E-14	1.04E-09	0.294	0.043
RABGGTB	5	0.33613106	1.78E-10	3.24E-06	0.78	0.57
IL11	5	0.3351914	2.87E-10	5.23E-06	0.156	0.004
PHPT1	5	0.33104773	1.47E-09	2.69E-05	0.812	0.711
PLA2G4A	5	0.32822014	5.39E-09	9.84E-05	0.436	0.211
DDX41	5	0.32720027	3.78E-09	6.90E-05	0.326	0.113
SAR1A	5	0.32190023	7.11E-08	0.00129729	0.633	0.434
CD55	5	0.32102503	1.64E-06	0.02996667	0.587	0.418
RAB13	5	0.32033436	3.62E-09	6.61E-05	0.899	0.797
CLIC4	5	0.31702845	4.62E-08	0.00084368	0.573	0.363

PID1	5	0.31448877	8.75E-07	0.0159571	0.573	0.391
MED21	5	0.31403017	9.99E-08	0.00182314	0.518	0.316
NOL7	5	0.31123063	2.20E-06	0.04013819	0.596	0.434
COX17	5	0.30964527	7.53E-08	0.00137473	0.83	0.672
RND3	5	0.30960566	1.80E-07	0.00327667	0.22	0.059
SCOC	5	0.30781549	1.56E-08	0.00028391	0.661	0.488
PGK1	5	0.30496819	1.58E-06	0.02881022	0.959	0.934
VDAC1	5	0.30378323	8.64E-08	0.00157723	0.876	0.77
NDUFC1	5	0.30306007	1.25E-09	2.29E-05	0.94	0.863
PAM	5	0.30044432	1.28E-06	0.02343703	0.72	0.617
CD68	5	0.29720689	1.01E-08	0.00018461	0.628	0.387
SMIM15	5	0.29557524	5.78E-07	0.01054041	0.532	0.355
ATP6V0E1	5	0.29512026	3.46E-08	0.00063065	0.931	0.859
KLF3	5	0.29421377	1.13E-06	0.0205279	0.39	0.207
ARF4	5	0.29382246	6.85E-07	0.01249722	0.798	0.711
HNRNPDL	5	0.29165547	1.85E-07	0.0033718	0.835	0.664
CD44	5	0.29056493	1.99E-10	3.63E-06	0.927	0.766
SMIM3	5	0.29055746	2.69E-11	4.90E-07	0.289	0.062
C4orf3	5	0.28999049	4.96E-11	9.05E-07	1	0.988
MRPL17	5	0.28957339	2.08E-06	0.03799412	0.514	0.328
WSB1	5	0.28906355	7.32E-08	0.00133524	0.913	0.797
A4GALT	5	0.2883601	7.21E-16	1.32E-11	0.239	0.004
FAM210A	5	0.28680938	2.84E-08	0.00051851	0.362	0.152
SAMD4A	5	0.28452065	3.49E-08	0.00063672	0.376	0.164
PLAUR	5	0.28452065	2.57E-06	0.04691856	0.358	0.188
CCND1	5	0.28313733	9.26E-16	1.69E-11	0.266	0.016
IFITM10	5	0.27771943	4.61E-07	0.00841077	0.33	0.148
CDKN1A	5	0.27731929	1.63E-07	0.00297778	0.298	0.109
SNRPG	5	0.27533062	7.29E-07	0.01330921	0.789	0.645
CALD1	5	0.27497988	2.83E-07	0.00517279	0.679	0.457
EPS8	5	0.27401107	9.10E-10	1.66E-05	0.39	0.148
RELB	5	0.27375953	2.56E-09	4.68E-05	0.298	0.09
ATP6V1C1	5	0.27335554	8.06E-07	0.01470598	0.427	0.234
RNF126	5	0.27275112	2.75E-10	5.01E-06	0.431	0.172
C11orf31	5	0.27078573	7.30E-08	0.00133288	0.803	0.688
GPRC5A	5	0.26880724	1.82E-07	0.00331433	0.303	0.117
SUMO3	5	0.26692163	9.70E-07	0.0177082	0.651	0.461
BCAT1	5	0.26687243	2.56E-07	0.00467072	0.661	0.453
BNIP3	5	0.26477256	2.29E-07	0.00417605	0.995	0.996

LY6E	5	0.26422306	4.18E-07	0.00762604	0.638	0.43
ZFYVE16	5	0.2636672	1.30E-10	2.38E-06	0.381	0.129
NOP10	5	0.26282746	2.40E-06	0.04386329	0.826	0.688
IFT22	5	0.26189228	3.34E-07	0.00609806	0.472	0.262
ZNF593	5	0.25698561	1.92E-06	0.03500985	0.518	0.32
SLC20A1	5	0.25662368	6.10E-09	0.00011128	0.307	0.098
RARRES3	5	-0.2505134	3.16E-08	0.00057659	0.06	0.242
UBB	5	-0.2509453	7.45E-07	0.01358643	0.798	0.922
FAU	5	-0.252174	1.29E-20	2.36E-16	1	1
EDF1	5	-0.2530547	8.82E-09	0.00016097	0.936	0.992
RPL32	5	-0.253966	3.59E-26	6.54E-22	1	1
DNM3OS	5	-0.2541432	1.33E-06	0.02421328	0.275	0.48
CD302	5	-0.2552267	4.63E-09	8.45E-05	0.151	0.387
RPL24	5	-0.2553976	1.57E-20	2.86E-16	1	1
ENPP1	5	-0.2558734	4.37E-07	0.00798233	0.174	0.379
BRK1	5	-0.2563844	8.04E-08	0.00146734	0.812	0.938
MTDH	5	-0.2568311	8.51E-07	0.01552329	0.573	0.738
SLC38A1	5	-0.2568748	1.03E-07	0.00188246	0.193	0.41
FMOD	5	-0.2579898	5.04E-08	0.0009204	0.977	0.996
EPB41L4A-AS1	5	-0.2584149	2.45E-07	0.00447671	0.766	0.91
NUCB2	5	-0.2586142	6.72E-07	0.01225344	0.399	0.617
RPL6	5	-0.2594889	1.05E-17	1.91E-13	1	1
CIRBP	5	-0.2595126	3.16E-08	0.00057598	1	1
BGN	5	-0.2615898	1.66E-07	0.00302755	0.995	1
RPL12	5	-0.2624919	9.30E-18	1.70E-13	1	1
ATP5A1	5	-0.2632773	2.56E-06	0.04671125	0.472	0.645
MZT2B	5	-0.2648951	5.33E-10	9.73E-06	0.931	0.992
RPS15	5	-0.2656435	1.09E-24	1.99E-20	1	1
TOMM20	5	-0.2672556	1.38E-08	0.00025145	0.798	0.949
RBM3	5	-0.2680096	6.45E-07	0.01176112	0.5	0.656
RGS10	5	-0.2687519	2.39E-06	0.04353921	0.463	0.641
NCOR1	5	-0.2701005	2.41E-07	0.00438915	0.179	0.375
MPST	5	-0.2712253	1.83E-07	0.00333407	0.307	0.52
ARPC1B	5	-0.271513	1.77E-07	0.00323556	0.22	0.438
CDKN2C	5	-0.2736507	1.15E-10	2.10E-06	0.096	0.34
SNHG8	5	-0.2754434	7.45E-08	0.00135884	0.835	0.953
PRCP	5	-0.2760353	2.01E-09	3.67E-05	0.142	0.379
GNB2L1	5	-0.2772619	1.73E-16	3.16E-12	1	1
SCG5	5	-0.2774329	6.74E-08	0.00123011	0.046	0.215

EIF3F	5	-0.2778306	4.17E-08	0.00076005	0.794	0.922
AC003090.1	5	-0.2779643	1.85E-09	3.38E-05	0.124	0.355
PLP2	5	-0.2793351	2.00E-07	0.00364731	0.651	0.82
C14orf166	5	-0.2804553	6.90E-08	0.00125882	0.688	0.824
RPS24	5	-0.2820384	1.92E-20	3.50E-16	1	1
IDH2	5	-0.2820541	2.51E-09	4.58E-05	0.087	0.301
NBEAL1	5	-0.2832915	3.52E-11	6.43E-07	0.977	0.996
RAC1	5	-0.2844826	8.85E-11	1.61E-06	0.876	0.973
PTMS	5	-0.2853432	4.64E-08	0.00084757	0.583	0.773
RPS14	5	-0.286134	1.81E-32	3.31E-28	1	1
ATP5D	5	-0.2869708	5.27E-09	9.62E-05	0.821	0.938
RPS4Y1	5	-0.2908288	2.18E-15	3.99E-11	0.991	0.996
COX7C	5	-0.2918437	4.04E-17	7.37E-13	1	1
YBX1	5	-0.2926634	8.14E-13	1.48E-08	0.963	0.992
CLDN11	5	-0.2929321	2.50E-12	4.56E-08	0.018	0.242
PPIC	5	-0.2930495	5.39E-07	0.00983678	0.339	0.582
RSPO3	5	-0.2950149	1.75E-11	3.20E-07	0.005	0.199
SLC44A1	5	-0.2957028	8.96E-08	0.00163571	0.211	0.414
CUTA	5	-0.2958089	1.76E-07	0.00321111	0.757	0.891
KDELR2	5	-0.296263	5.76E-07	0.01051196	0.803	0.91
MYL12B	5	-0.2966078	6.83E-08	0.00124616	0.486	0.66
C10orf107	5	-0.2969685	2.66E-13	4.86E-09	0.028	0.273
GSTP1	5	-0.2984149	1.04E-10	1.90E-06	0.931	0.973
MATN4	5	-0.2986849	3.99E-07	0.00728384	0.206	0.426
BEX2	5	-0.301423	2.68E-08	0.00048873	0.239	0.465
MRAP2	5	-0.3019411	2.72E-12	4.96E-08	0.009	0.223
AHNAK	5	-0.3038029	1.16E-07	0.0021134	0.317	0.535
HSP90AB1	5	-0.3069893	6.99E-09	0.00012747	0.936	0.969
RPS4X	5	-0.3074635	1.15E-28	2.11E-24	1	1
RPL36AL	5	-0.3091696	2.96E-16	5.39E-12	0.995	1
EIF3D	5	-0.3095602	3.39E-08	0.00061911	0.697	0.824
C19orf43	5	-0.3108007	1.12E-10	2.04E-06	0.752	0.918
SEP1	5	-0.3116334	9.98E-10	1.82E-05	0.202	0.445
PSAP	5	-0.3119773	5.21E-09	9.50E-05	0.94	0.965
SDF4	5	-0.3126939	6.35E-09	0.00011583	0.697	0.859
TTC3	5	-0.3127245	1.71E-08	0.00031114	0.697	0.863
RABAC1	5	-0.3131945	8.94E-11	1.63E-06	0.775	0.906
ATP5L	5	-0.3141118	6.85E-11	1.25E-06	0.927	0.969
RPL28	5	-0.3150448	1.33E-29	2.44E-25	1	1

RPS3	5	-0.3173534	6.11E-34	1.12E-29	1	1
RPL5	5	-0.3177204	2.19E-21	4.00E-17	1	1
FHL1	5	-0.3181556	2.99E-07	0.00545101	0.326	0.535
CCNG1	5	-0.3183772	4.72E-08	0.00086153	0.5	0.668
SLC25A6	5	-0.3185541	1.65E-12	3.01E-08	0.963	0.992
SPINT2	5	-0.3187519	2.29E-10	4.18E-06	0.321	0.613
CYSTM1	5	-0.3202903	3.37E-08	0.00061442	0.404	0.617
RPA3	5	-0.3221117	5.74E-10	1.05E-05	0.459	0.695
RPL26	5	-0.3227072	5.52E-32	1.01E-27	1	1
TIMP2	5	-0.3228603	6.40E-09	0.00011681	0.518	0.727
CYBRD1	5	-0.3237826	9.28E-10	1.69E-05	0.427	0.688
CHID1	5	-0.3263593	3.30E-09	6.03E-05	0.587	0.762
RPS28	5	-0.3280129	5.77E-38	1.05E-33	1	1
RPS9	5	-0.3282228	5.67E-32	1.03E-27	1	1
EIF2S3	5	-0.3287866	5.83E-08	0.00106457	0.495	0.684
MALAT1	5	-0.3288264	4.65E-09	8.48E-05	1	1
LDHB	5	-0.3292114	2.09E-11	3.82E-07	0.697	0.898
IGBP1	5	-0.3293401	6.92E-09	0.00012632	0.573	0.738
RPL15	5	-0.3293496	1.54E-41	2.82E-37	1	1
RPL7A	5	-0.3297765	7.05E-29	1.29E-24	1	1
PSMA7	5	-0.3302696	1.53E-10	2.80E-06	0.729	0.938
HTR2A	5	-0.3310785	1.16E-09	2.12E-05	0.041	0.242
EIF3E	5	-0.3319009	2.93E-16	5.35E-12	1	1
PRDX6	5	-0.332526	4.06E-10	7.42E-06	0.752	0.898
EIF3M	5	-0.3346391	2.90E-11	5.30E-07	0.477	0.754
BSG	5	-0.3386489	2.91E-11	5.32E-07	0.674	0.875
COMMD6	5	-0.3386871	3.58E-18	6.54E-14	0.991	1
CCDC8	5	-0.3416326	7.06E-12	1.29E-07	0.078	0.332
ANGPTL5	5	-0.3473224	1.21E-09	2.20E-05	0.252	0.492
LRRC2	5	-0.348561	2.04E-10	3.71E-06	0.151	0.398
CEBPD	5	-0.348644	2.05E-06	0.03731718	0.757	0.91
DBP	5	-0.3494236	1.97E-07	0.00358628	0.243	0.426
RPS19	5	-0.3500337	1.16E-33	2.11E-29	1	1
GPX3	5	-0.350896	4.22E-08	0.00077069	0.936	0.984
ST13	5	-0.3551938	5.84E-15	1.07E-10	0.931	0.98
HERPUD1	5	-0.3566792	9.96E-10	1.82E-05	0.413	0.66
SSPN	5	-0.357416	2.55E-09	4.65E-05	0.206	0.445
RPS21	5	-0.3575507	1.71E-27	3.12E-23	1	1
RPS12	5	-0.3595792	6.27E-32	1.14E-27	1	1

CPE	5	-0.3622033	2.60E-09	4.74E-05	0.628	0.777
RPL3	5	-0.3650636	3.37E-32	6.16E-28	1	1
HDLBP	5	-0.365156	5.54E-11	1.01E-06	0.45	0.676
GLTSCR2	5	-0.3677544	1.10E-14	2.01E-10	0.894	0.996
MYL6	5	-0.3678428	3.38E-14	6.17E-10	0.904	0.965
TXNIP	5	-0.3696934	1.37E-15	2.49E-11	0.06	0.363
ARL2BP	5	-0.371305	1.52E-11	2.78E-07	0.39	0.672
ZNF90	5	-0.3718641	2.65E-10	4.83E-06	0.399	0.652
BTF3	5	-0.3740072	2.65E-24	4.84E-20	0.991	1
PCBP2	5	-0.3751354	9.37E-15	1.71E-10	0.899	0.984
RPS15A	5	-0.3763323	3.48E-39	6.34E-35	1	1
RPL27	5	-0.3794955	4.64E-28	8.46E-24	1	1
H3F3A	5	-0.3802289	7.05E-18	1.29E-13	0.954	0.996
NDUFA11	5	-0.3817073	9.24E-15	1.69E-10	0.826	0.957
RPL35	5	-0.3817919	2.24E-38	4.08E-34	1	1
ATP5O	5	-0.3855678	1.95E-14	3.57E-10	0.706	0.887
RPS6	5	-0.3866819	6.75E-48	1.23E-43	1	1
MFGE8	5	-0.3875956	3.02E-09	5.51E-05	0.83	0.941
PPT1	5	-0.3885143	1.34E-12	2.45E-08	0.339	0.625
ATP5G2	5	-0.3901779	3.62E-21	6.61E-17	0.995	0.996
RPS25	5	-0.3942113	7.39E-43	1.35E-38	1	1
TCF4	5	-0.3962679	2.26E-08	0.00041218	0.353	0.547
SERPINI1	5	-0.3967332	2.52E-07	0.00460432	0.151	0.344
NFIA	5	-0.3969193	5.45E-13	9.94E-09	0.229	0.527
EIF3K	5	-0.3971867	1.15E-15	2.09E-11	0.766	0.922
RPL27A	5	-0.3973213	5.10E-44	9.30E-40	1	1
HIST1H4C	5	-0.3989334	5.67E-10	1.03E-05	0.353	0.609
ATRNL1	5	-0.4031088	2.13E-14	3.88E-10	0.092	0.387
PPIA	5	-0.4034345	9.15E-20	1.67E-15	0.972	0.992
RPL11	5	-0.4040788	1.71E-46	3.12E-42	1	1
KHDRBS3	5	-0.4052346	4.45E-18	8.12E-14	0.028	0.348
CD99	5	-0.4092387	1.15E-19	2.10E-15	0.972	0.996
UQCRH	5	-0.411521	7.47E-21	1.36E-16	0.931	0.992
LGALS1	5	-0.4122834	3.58E-10	6.53E-06	0.922	0.98
RPL13	5	-0.412381	6.74E-50	1.23E-45	1	1
ZBTB20	5	-0.4131668	6.23E-14	1.14E-09	0.271	0.574
PLPP1	5	-0.4136439	3.00E-11	5.47E-07	0.101	0.348
RPL22	5	-0.4140658	2.47E-36	4.52E-32	1	1
SSR2	5	-0.4220078	4.49E-19	8.19E-15	0.78	0.969

ZFAS1	5	-0.4248878	7.24E-23	1.32E-18	0.991	1
HNRNPA1	5	-0.4262724	2.26E-19	4.12E-15	0.986	0.996
RPS27	5	-0.4266338	7.26E-50	1.32E-45	1	1
RPL39	5	-0.4295598	1.56E-36	2.85E-32	1	1
HINT1	5	-0.4297408	2.08E-20	3.79E-16	0.959	0.984
HOXC10	5	-0.4334894	4.97E-12	9.07E-08	0.633	0.789
RPL35A	5	-0.4340461	1.15E-47	2.10E-43	1	1
UBA52	5	-0.4344446	3.68E-42	6.72E-38	1	1
ITM2A	5	-0.4347976	8.24E-10	1.50E-05	0.528	0.742
RPL37A	5	-0.43547	2.42E-52	4.42E-48	1	1
APOD	5	-0.4365645	5.85E-10	1.07E-05	0.239	0.508
RPL29	5	-0.4372156	5.66E-43	1.03E-38	1	1
RPS7	5	-0.4383321	1.38E-38	2.51E-34	1	1
TOMM7	5	-0.4387809	1.50E-42	2.73E-38	1	1
GAS6	5	-0.4389387	4.85E-14	8.86E-10	0.349	0.648
RPS2	5	-0.4401219	1.09E-48	1.98E-44	1	1
SLC25A3	5	-0.4405061	4.24E-18	7.73E-14	0.826	0.965
GNAS	5	-0.4415567	1.18E-17	2.16E-13	0.431	0.785
ISLR	5	-0.4422585	2.06E-11	3.75E-07	0.376	0.625
MT-ND4	5	-0.4460265	2.88E-10	5.25E-06	0.972	0.953
RPLP1	5	-0.4472734	1.32E-49	2.41E-45	1	1
DSEL	5	-0.4496728	1.58E-11	2.89E-07	0.151	0.418
NPM1	5	-0.4520817	9.89E-26	1.80E-21	1	1
C2orf82	5	-0.4539907	3.06E-15	5.58E-11	0.977	1
PTRF	5	-0.4556324	1.61E-14	2.94E-10	0.33	0.617
RPS18	5	-0.4584906	1.60E-49	2.92E-45	1	1
RPL19	5	-0.4605431	1.41E-55	2.58E-51	1	1
RPL9	5	-0.4610925	8.71E-50	1.59E-45	1	1
RPS23	5	-0.46207	2.59E-51	4.72E-47	1	1
RPL21	5	-0.464387	2.69E-57	4.92E-53	1	1
RPL37	5	-0.4645586	3.49E-45	6.37E-41	1	1
COL9A3	5	-0.467353	3.55E-14	6.48E-10	0.826	0.934
RPS5	5	-0.4679462	1.01E-45	1.84E-41	1	1
OXA1L	5	-0.4681195	5.60E-18	1.02E-13	0.394	0.734
PABPC1	5	-0.4683487	1.57E-19	2.86E-15	0.862	0.98
EIF2A	5	-0.4705894	7.08E-17	1.29E-12	0.679	0.852
RPL8	5	-0.4747944	1.28E-50	2.34E-46	1	1
PEBP1	5	-0.4748489	1.57E-18	2.86E-14	0.748	0.953
COL2A1	5	-0.4760485	9.31E-09	0.00016997	0.725	0.93

UQCRB	5	-0.4769521	1.57E-25	2.87E-21	0.972	1
CRIP2	5	-0.4769977	1.36E-12	2.48E-08	0.225	0.5
RPSA	5	-0.4789152	8.88E-35	1.62E-30	1	1
PDIA6	5	-0.4797697	8.03E-17	1.46E-12	0.651	0.895
RPL18A	5	-0.4808532	5.64E-53	1.03E-48	1	1
RHOBTB3	5	-0.4816212	1.37E-16	2.50E-12	0.683	0.898
NNMT	5	-0.482387	5.82E-15	1.06E-10	0.839	0.98
HLA-B	5	-0.4832876	4.48E-17	8.18E-13	0.693	0.918
RPL13A	5	-0.4872999	3.44E-58	6.28E-54	1	1
RPL10	5	-0.4878635	4.35E-55	7.93E-51	1	1
SLC14A1	5	-0.4924246	6.21E-13	1.13E-08	0.257	0.527
RPL14	5	-0.4925911	6.45E-48	1.18E-43	1	1
HLA-A	5	-0.4969344	2.58E-17	4.71E-13	0.904	0.988
RPL31	5	-0.4997112	9.77E-49	1.78E-44	1	1
PLXDC2	5	-0.5035108	2.78E-16	5.07E-12	0.541	0.805
HLA-C	5	-0.5084519	1.93E-17	3.52E-13	0.826	0.965
PRUNE2	5	-0.5106231	1.31E-14	2.39E-10	0.298	0.629
RPS13	5	-0.5144991	2.91E-55	5.31E-51	1	1
NAP1L1	5	-0.5194776	1.94E-27	3.54E-23	0.94	0.984
SEC11A	5	-0.5205498	1.22E-23	2.23E-19	0.761	0.941
RPS27A	5	-0.5216722	1.72E-60	3.14E-56	1	1
MT-CO3	5	-0.52852	7.65E-12	1.40E-07	0.922	0.934
EEF1B2	5	-0.5311863	1.19E-32	2.17E-28	0.945	1
RPL30	5	-0.5313954	1.57E-55	2.87E-51	1	1
RPL23	5	-0.5407421	7.58E-44	1.38E-39	1	1
CCDC102B	5	-0.5455941	1.90E-25	3.46E-21	0.041	0.465
MT-ND1	5	-0.5551073	1.20E-12	2.20E-08	0.518	0.781
MT-ATP6	5	-0.5596617	1.03E-12	1.87E-08	0.807	0.898
RPL23A	5	-0.5602615	1.75E-62	3.19E-58	1	1
RPS29	5	-0.562032	4.12E-58	7.51E-54	1	1
EIF4B	5	-0.5759665	9.06E-26	1.65E-21	0.803	0.961
RPLP2	5	-0.5797397	7.39E-63	1.35E-58	1	1
TUBB	5	-0.587079	1.70E-23	3.09E-19	0.601	0.922
NPC2	5	-0.5878319	3.58E-33	6.54E-29	0.963	1
RPL4	5	-0.5891077	9.55E-45	1.74E-40	1	1
RPL34	5	-0.5896377	6.15E-61	1.12E-56	1	1
MT-CO2	5	-0.5937187	7.53E-14	1.37E-09	0.936	0.965
INHBA	5	-0.598278	1.63E-17	2.97E-13	0.225	0.59
VIM	5	-0.598863	3.90E-28	7.13E-24	1	1

MT-CO1	5	-0.6003683	3.88E-13	7.09E-09	0.963	0.969
EIF3L	5	-0.6069107	4.99E-31	9.10E-27	0.771	0.996
XAGE2	5	-0.6086714	1.68E-10	3.06E-06	0.009	0.191
ZFP36L2	5	-0.6087268	3.23E-16	5.90E-12	0.151	0.48
TPT1	5	-0.6108689	3.70E-57	6.76E-53	1	1
RPL18	5	-0.6183312	2.40E-61	4.39E-57	1	1
RPS3A	5	-0.6193799	4.58E-66	8.36E-62	1	1
RPL10A	5	-0.619507	1.08E-54	1.97E-50	1	1
YBX3	5	-0.623334	2.25E-30	4.10E-26	0.812	0.984
CTGF	5	-0.6431579	4.38E-16	8.00E-12	0.261	0.617
SLC40A1	5	-0.6471443	1.01E-16	1.85E-12	0.119	0.453
HIST1H1C	5	-0.648858	5.00E-11	9.13E-07	0.271	0.531
RPL7	5	-0.6512818	3.25E-56	5.93E-52	1	1
RPS17	5	-0.6520849	2.05E-60	3.75E-56	1	1
RPS16	5	-0.6574506	1.32E-65	2.41E-61	1	1
RPS8	5	-0.6638292	1.27E-63	2.32E-59	1	1
EEF2	5	-0.6694624	1.56E-33	2.85E-29	1	1
CITED2	5	-0.677483	1.80E-21	3.29E-17	0.271	0.684
CCNI	5	-0.678491	2.43E-44	4.43E-40	0.995	1
P4HB	5	-0.6933203	1.51E-30	2.76E-26	0.789	0.977
EEF1A1	5	-0.6948047	1.26E-62	2.30E-58	1	1
SSR3	5	-0.7272031	9.92E-32	1.81E-27	0.807	0.973
NEAT1	5	-0.728996	2.22E-35	4.06E-31	1	1
MT-CYB	5	-0.7412196	6.15E-20	1.12E-15	0.656	0.875
MT-ND2	5	-0.7609417	4.34E-15	7.91E-11	0.619	0.785
B2M	5	-0.7645681	5.13E-46	9.35E-42	1	1
CHRDL2	5	-0.7848787	1.41E-17	2.57E-13	0.394	0.719
CLEC3A	5	-0.790828	3.50E-17	6.39E-13	0.693	0.906
SPARC	5	-0.8077599	1.84E-28	3.36E-24	0.39	0.832
PAX1	5	-0.8209689	1.79E-29	3.27E-25	0.528	0.875
FTH1	5	-0.8243489	6.77E-54	1.24E-49	1	1
EIF3H	5	-0.8389578	1.25E-55	2.28E-51	0.963	1
RPLP0	5	-0.8452373	2.75E-70	5.01E-66	1	1
CRTAP	5	-0.8467567	2.45E-49	4.48E-45	0.495	0.969
CAPS	5	-0.8563313	1.16E-27	2.11E-23	0.716	0.953
CD9	5	-0.8590509	1.81E-36	3.30E-32	0.372	0.828
CFH	5	-0.859149	4.77E-15	8.71E-11	0.183	0.512
MT-ND3	5	-0.8858117	1.81E-22	3.30E-18	0.647	0.871
ACTG1	5	-0.8858735	6.48E-39	1.18E-34	0.881	0.988

GREM1	5	-0.9488709	4.02E-21	7.33E-17	0.115	0.52
OGN	5	-0.9769744	4.58E-29	8.35E-25	0.908	0.988
RPS11	5	-1.0523182	6.52E-74	1.19E-69	0.995	1
RPS20	5	-1.0548791	4.79E-75	8.74E-71	1	1
CRISPLD1	5	-1.1355991	1.06E-34	1.93E-30	0.183	0.707
ADIRF	5	-1.2935023	6.57E-48	1.20E-43	0.289	0.895
SPTSSB	5	-1.9345241	3.47E-67	6.34E-63	0.041	0.875
FRZB	5	-1.9827525	5.14E-63	9.37E-59	0.739	0.992
**gene**	**cluster**	**avg_logFC**	**p_val**	**p_val_adj**	**pct.1**	**pct.2**
SPP1	6	2.40594361	1.42E-23	2.60E-19	0.749	0.131
BMP2	6	1.49056394	9.71E-27	1.77E-22	0.733	0.071
PLA2G2A	6	1.46193036	3.00E-16	5.48E-12	0.712	0.253
PHLDA1	6	1.44057551	1.45E-20	2.64E-16	0.691	0.141
PRG4	6	1.41856484	1.66E-20	3.03E-16	0.617	0.051
TGFBI	6	1.38413386	2.78E-21	5.07E-17	0.745	0.222
FOSL1	6	1.31136251	1.28E-12	2.33E-08	0.56	0.162
CHI3L2	6	1.22546307	2.27E-24	4.14E-20	0.901	0.455
VEGFA	6	1.20734118	3.87E-28	7.06E-24	0.926	0.434
PTGS2	6	1.19728382	1.60E-25	2.92E-21	0.728	0.071
MRPS6	6	1.16168897	4.72E-22	8.62E-18	0.988	0.909
COMP	6	1.11750329	4.12E-15	7.51E-11	0.947	0.687
CTHRC1	6	1.08760386	1.28E-22	2.33E-18	0.663	0.061
IL11	6	1.07501647	2.23E-13	4.07E-09	0.403	0
MT2A	6	1.06891995	6.72E-17	1.23E-12	1	1
STK17A	6	1.05435835	4.14E-21	7.56E-17	0.819	0.323
CCDC85B	6	1.04381369	2.17E-20	3.96E-16	0.975	0.788
CTSL	6	1.04321293	1.80E-12	3.28E-08	0.831	0.535
MT1X	6	0.96169082	5.01E-13	9.14E-09	0.996	0.98
IGFBP3	6	0.93703017	6.82E-14	1.24E-09	0.683	0.253
CTSD	6	0.92232911	6.54E-08	0.00119325	0.979	0.939
UPP1	6	0.86200401	2.52E-17	4.60E-13	0.745	0.253
DDIT4L	6	0.85813334	1.22E-17	2.23E-13	0.914	0.525
PPP3CA	6	0.83935796	1.98E-16	3.61E-12	0.942	0.667
ARRDC3	6	0.8291378	4.80E-15	8.76E-11	0.741	0.323
IGFBP5	6	0.8024681	5.09E-08	0.00092922	0.469	0.172
WTAP	6	0.78220389	3.70E-16	6.76E-12	0.889	0.556
MT1G	6	0.77866989	4.00E-07	0.00729131	0.955	0.919
SOD2	6	0.77187271	3.71E-15	6.76E-11	0.988	0.869
CA9	6	0.7665561	6.33E-12	1.15E-07	0.691	0.333

HILPDA	6	0.76604189	2.20E-11	4.01E-07	0.593	0.212
TREM1	6	0.7487921	1.19E-16	2.16E-12	0.494	0.01
EIF4A2	6	0.73935327	1.52E-19	2.78E-15	0.979	0.889
ENO2	6	0.73602951	3.87E-16	7.06E-12	0.765	0.364
TNFRSF12A	6	0.73233041	9.20E-14	1.68E-09	0.914	0.616
ERO1A	6	0.72864572	4.45E-13	8.11E-09	0.786	0.444
PMP22	6	0.70399159	3.65E-16	6.67E-12	0.955	0.707
EPYC	6	0.70100671	7.41E-11	1.35E-06	0.453	0.081
NRN1	6	0.69705812	4.82E-08	0.00087866	0.675	0.414
SLC25A37	6	0.69061873	1.23E-14	2.24E-10	0.918	0.646
SLC5A3	6	0.68549754	1.22E-10	2.22E-06	0.807	0.566
RABGGTB	6	0.65624784	1.47E-17	2.69E-13	0.827	0.364
EGLN3	6	0.65393902	3.38E-13	6.16E-09	0.547	0.121
SMOX	6	0.64530065	2.70E-11	4.92E-07	0.695	0.333
ISG20	6	0.63663144	3.00E-16	5.48E-12	0.848	0.364
HLA-DRB1	6	0.62605851	7.40E-11	1.35E-06	0.58	0.222
ANGPTL4	6	0.59810363	2.96E-07	0.00539418	0.44	0.162
TNFSF11	6	0.59271278	7.41E-11	1.35E-06	0.35	0.01
SERPINE2	6	0.59125907	1.21E-09	2.20E-05	1	1
GAPDH	6	0.5912441	6.48E-12	1.18E-07	1	1
FAM162A	6	0.58801526	3.37E-16	6.16E-12	0.984	0.838
C11orf96	6	0.58534503	2.77E-09	5.05E-05	0.461	0.111
CFD	6	0.58449663	1.58E-07	0.00288601	0.329	0.061
TMEM45A	6	0.58144319	2.31E-11	4.22E-07	0.947	0.778
SPRY2	6	0.57835624	3.12E-13	5.69E-09	0.654	0.212
AK2	6	0.57676373	3.50E-10	6.39E-06	0.852	0.667
BTG1	6	0.57532791	8.77E-17	1.60E-12	0.992	0.909
EPS8	6	0.57425029	7.50E-14	1.37E-09	0.667	0.232
IFRD1	6	0.56939398	3.39E-10	6.19E-06	0.695	0.404
NT5C3A	6	0.56107431	3.06E-11	5.58E-07	0.523	0.152
PPFIBP1	6	0.55100373	1.73E-11	3.15E-07	0.765	0.455
INSIG2	6	0.54777619	1.65E-09	3.01E-05	0.774	0.525
BNIP3	6	0.54511779	2.89E-15	5.28E-11	1	0.99
PPP1CB	6	0.53878362	1.90E-07	0.00346867	0.877	0.717
HMGA2	6	0.53487278	6.99E-14	1.27E-09	0.477	0.04
VAPA	6	0.53065515	1.02E-14	1.87E-10	0.975	0.859
PDE4B	6	0.52464277	2.50E-12	4.55E-08	0.547	0.141
SERPINE1	6	0.5241989	2.76E-08	0.00050296	0.539	0.212
GBE1	6	0.51883578	1.06E-07	0.0019273	0.774	0.596

PTP4A1	6	0.51623425	5.24E-09	9.57E-05	0.716	0.444
BZW1	6	0.51553891	2.71E-11	4.95E-07	0.881	0.616
PPP1R15A	6	0.50095419	6.68E-09	0.00012197	0.58	0.263
DDIT3	6	0.49672102	6.27E-08	0.00114423	0.757	0.505
ARF6	6	0.49487595	2.78E-10	5.07E-06	0.815	0.606
ADM	6	0.48835277	3.07E-08	0.00056034	0.848	0.586
CLK1	6	0.48657814	4.67E-09	8.53E-05	0.63	0.323
EVI2A	6	0.48118428	1.00E-11	1.83E-07	0.44	0.061
SLC2A1	6	0.4806899	5.76E-07	0.01051596	0.506	0.232
NTMT1	6	0.46127181	9.41E-11	1.72E-06	0.621	0.263
WDR45B	6	0.45908239	5.62E-10	1.03E-05	0.605	0.253
PTGES3	6	0.4568895	2.78E-14	5.08E-10	0.988	0.909
DDX41	6	0.4516712	6.87E-07	0.01254254	0.428	0.162
SGIP1	6	0.44782279	1.96E-09	3.57E-05	0.395	0.071
SRGN	6	0.44685304	1.06E-07	0.00194257	0.457	0.162
WSB1	6	0.44470561	6.99E-08	0.00127581	0.848	0.667
BAALC	6	0.44143585	1.02E-06	0.01863393	0.683	0.424
SMIM3	6	0.43278292	1.67E-10	3.05E-06	0.473	0.111
VDAC1	6	0.43262109	3.56E-09	6.50E-05	0.93	0.788
S100A2	6	0.4280955	5.85E-09	0.00010673	0.835	0.505
RGS2	6	0.42381425	8.74E-07	0.01594476	0.63	0.354
WDR33	6	0.42148052	2.44E-07	0.00444346	0.605	0.333
A4GALT	6	0.42124685	1.37E-10	2.51E-06	0.399	0.051
CD55	6	0.41992663	4.90E-07	0.00895011	0.712	0.434
PHF1	6	0.41828521	7.46E-09	0.00013611	0.584	0.253
PLAUR	6	0.40856565	1.29E-06	0.02353243	0.634	0.343
ARPP19	6	0.40690314	6.61E-09	0.00012068	0.753	0.465
NRP2	6	0.39828094	1.65E-07	0.00301484	0.444	0.152
ZNF385D	6	0.39235929	3.39E-08	0.00061903	0.724	0.485
MIF	6	0.39112131	1.91E-16	3.49E-12	1	1
RNF126	6	0.38607392	9.76E-07	0.01781561	0.498	0.232
GRB10	6	0.38516853	4.45E-08	0.00081273	0.3	0.03
RELB	6	0.37685726	2.39E-07	0.00436501	0.37	0.101
NAMPT	6	0.37502408	1.06E-06	0.01926249	0.63	0.384
ADSS	6	0.3682976	2.31E-06	0.04217703	0.724	0.475
SLC39A8	6	0.36751629	4.67E-08	0.00085125	0.519	0.212
FN1	6	0.36498321	6.17E-08	0.0011264	1	1
CLDND1	6	0.36283745	1.29E-07	0.00236297	0.63	0.354
PLA2G4A	6	0.36101335	9.18E-09	0.00016746	0.469	0.141

COX17	6	0.36070721	3.07E-08	0.00056037	0.897	0.677
NDRG1	6	0.35712287	5.85E-08	0.00106687	0.811	0.535
LTBP1	6	0.35482138	7.74E-10	1.41E-05	0.342	0.02
HMGA1	6	0.35482138	1.47E-08	0.00026912	0.7	0.384
TSHZ2	6	0.34174929	4.67E-08	0.00085197	0.251	0
SNHG12	6	0.34137058	2.59E-06	0.04730113	0.514	0.232
LINC00493	6	0.33702016	2.96E-07	0.00540177	0.901	0.707
CD44	6	0.33015265	2.66E-06	0.04860608	0.918	0.697
SLC25A11	6	0.32767039	7.24E-07	0.01321166	0.527	0.242
RORA	6	0.32583384	6.57E-07	0.0119871	0.593	0.323
SELK	6	0.32063001	2.23E-06	0.04065016	0.914	0.747
EMP3	6	0.32038552	6.44E-07	0.01174391	0.992	0.919
FOXA3	6	0.31220679	2.71E-06	0.04940793	0.342	0.091
PTGES	6	0.30905693	2.45E-08	0.00044717	0.259	0
YWHAE	6	0.30810008	2.30E-06	0.04192041	0.955	0.869
ZFYVE16	6	0.30245339	1.73E-07	0.00314795	0.391	0.101
SPHK1	6	0.29873191	1.27E-06	0.02309001	0.366	0.111
ATP6V1C1	6	0.2903534	2.47E-06	0.04498368	0.56	0.283
CHMP4B	6	0.28616301	1.06E-06	0.01928055	0.77	0.475
CYGB	6	0.2595112	2.25E-06	0.04113698	0.333	0.081
TNS1	6	0.25750417	1.19E-06	0.02178929	0.379	0.111
RPL13	6	-0.2524994	6.94E-14	1.27E-09	1	1
ENPP1	6	-0.2525851	3.05E-07	0.00557113	0.14	0.384
RPL6	6	-0.2547448	1.24E-08	0.00022624	1	1
RPS25	6	-0.2565442	1.59E-09	2.90E-05	1	1
SERTAD4-AS1	6	-0.2586674	5.22E-09	9.53E-05	0.086	0.343
RP11-71N10.1	6	-0.2588616	3.05E-11	5.57E-07	0.033	0.273
RPS15A	6	-0.2601775	1.98E-09	3.61E-05	1	1
IDH1	6	-0.2602615	2.31E-08	0.00042068	0.074	0.303
HOXB8	6	-0.2628378	3.91E-09	7.14E-05	0.033	0.232
COMMD6	6	-0.2649401	5.98E-08	0.00109098	0.996	1
RPS4X	6	-0.2661394	1.62E-11	2.95E-07	1	1
MBTPS1	6	-0.2669717	1.71E-09	3.12E-05	0.091	0.354
DPYSL3	6	-0.2669717	5.07E-09	9.25E-05	0.078	0.323
RPS27	6	-0.2673307	2.81E-09	5.12E-05	1	1
ISM1	6	-0.2688464	1.16E-07	0.00211898	0.045	0.232
HS3ST1	6	-0.2688733	2.84E-07	0.0051752	0.086	0.303
SCD	6	-0.2693329	4.24E-07	0.0077299	0.099	0.313
MPP6	6	-0.2693329	2.14E-07	0.00391099	0.156	0.404

TMEM160	6	-0.2701772	1.49E-06	0.02720327	0.185	0.424
RPL14	6	-0.2720081	5.76E-11	1.05E-06	1	1
OSBPL9	6	-0.2768668	3.59E-07	0.00654951	0.144	0.384
ITGA11	6	-0.2842586	2.87E-07	0.00524554	0.091	0.303
COX7C	6	-0.2856196	2.47E-09	4.51E-05	0.996	1
RPL12	6	-0.2878356	5.44E-12	9.92E-08	1	1
CTDNEP1	6	-0.2879093	1.53E-07	0.00278411	0.206	0.475
RPL19	6	-0.289172	2.54E-16	4.64E-12	1	1
SPINT2	6	-0.2940255	6.49E-07	0.01183411	0.342	0.626
RPS6	6	-0.2982121	1.06E-15	1.93E-11	1	1
DSEL	6	-0.2987262	1.52E-06	0.02770212	0.074	0.263
C12orf57	6	-0.2988234	1.89E-07	0.00345488	0.947	0.98
RPN1	6	-0.3007584	1.54E-07	0.00280559	0.132	0.374
RPL27A	6	-0.3013637	6.54E-14	1.19E-09	1	1
GSTP1	6	-0.3020497	6.67E-08	0.00121778	0.979	0.99
TSPAN5	6	-0.3020933	5.16E-08	0.00094155	0.123	0.374
RPS7	6	-0.3022026	3.06E-14	5.58E-10	1	1
RPS5	6	-0.3028586	6.82E-13	1.24E-08	1	1
SLC25A3	6	-0.3042703	1.22E-06	0.02224138	0.864	0.98
FMOD	6	-0.3048418	7.23E-07	0.01320088	0.926	0.99
CKAP4	6	-0.3062639	3.07E-07	0.00560555	0.218	0.485
TOMM7	6	-0.3092514	4.71E-11	8.59E-07	1	1
RPL22	6	-0.3098838	1.70E-09	3.11E-05	1	1
BTF3	6	-0.3121911	2.12E-08	0.00038669	0.984	0.99
MAF	6	-0.3122803	7.08E-08	0.00129146	0.14	0.394
COL14A1	6	-0.312591	7.88E-10	1.44E-05	0.012	0.192
KDELR3	6	-0.3136332	1.61E-09	2.94E-05	0.16	0.465
MYEOV2	6	-0.314055	9.29E-07	0.01695399	0.683	0.889
SSPN	6	-0.3167123	1.20E-06	0.02184476	0.198	0.434
ARL2	6	-0.3179241	1.92E-06	0.03503971	0.37	0.616
DCN	6	-0.3184923	1.16E-06	0.02125626	1	1
FTL	6	-0.3186723	1.86E-09	3.40E-05	1	1
RPS24	6	-0.3202791	1.05E-11	1.92E-07	1	1
NME2	6	-0.321462	1.02E-06	0.01861447	0.222	0.455
TMEM219	6	-0.3227915	1.34E-06	0.02451691	0.646	0.798
CHCHD2	6	-0.323497	3.81E-09	6.95E-05	0.963	1
RPL29	6	-0.3259024	1.50E-15	2.74E-11	1	1
FXYD6	6	-0.3275003	1.53E-08	0.00027868	0.778	0.96
EDF1	6	-0.3288471	9.34E-10	1.70E-05	0.942	0.99

RPS19	6	-0.3307851	8.11E-13	1.48E-08	1	1
ATP5E	6	-0.3309541	1.00E-09	1.83E-05	0.992	1
ATRNL1	6	-0.3310265	7.19E-09	0.00013117	0.123	0.384
EIF4B	6	-0.3317033	1.38E-06	0.02526193	0.72	0.879
NENF	6	-0.3323556	5.90E-07	0.01075769	0.93	0.939
RPS21	6	-0.332934	4.91E-10	8.96E-06	1	1
SLC44A1	6	-0.3349302	1.62E-06	0.02963485	0.206	0.434
GREM1	6	-0.3353333	6.12E-07	0.01116719	0.029	0.182
AP2M1	6	-0.3377429	2.45E-06	0.04474167	0.514	0.697
RPL18A	6	-0.3397199	1.47E-15	2.69E-11	1	1
HNRNPA2B1	6	-0.3400766	1.48E-06	0.02709387	0.494	0.727
NBEAL1	6	-0.3402223	4.98E-09	9.08E-05	0.979	0.99
DMKN	6	-0.3419216	2.64E-07	0.00481042	0.313	0.586
RPL35	6	-0.3426136	7.14E-15	1.30E-10	1	1
TCEB2	6	-0.3426504	1.75E-07	0.00320179	0.971	0.99
TXN	6	-0.3428665	1.15E-06	0.02093868	0.852	0.96
RPL3	6	-0.343877	1.90E-14	3.47E-10	1	1
MRC2	6	-0.345873	1.13E-09	2.07E-05	0.193	0.505
PPIA	6	-0.3494418	4.23E-10	7.72E-06	0.963	0.97
SSR2	6	-0.3524304	1.39E-09	2.54E-05	0.823	0.949
LRP10	6	-0.3530104	4.50E-07	0.00821126	0.317	0.566
MLEC	6	-0.3548077	8.83E-07	0.01610732	0.333	0.576
C14orf1	6	-0.3556173	1.37E-08	0.00025003	0.206	0.495
PRKCDBP	6	-0.357374	1.37E-06	0.02493631	0.37	0.606
SLC38A1	6	-0.3584607	2.64E-11	4.81E-07	0.173	0.515
RPS13	6	-0.3596299	4.75E-14	8.66E-10	1	1
RPL37A	6	-0.3602386	2.18E-18	3.97E-14	1	1
RPL31	6	-0.3608184	1.13E-14	2.05E-10	1	1
RPL9	6	-0.3658967	1.76E-16	3.21E-12	1	1
NFIA	6	-0.3666374	2.59E-08	0.00047212	0.173	0.444
RPL21	6	-0.3683846	2.51E-18	4.57E-14	1	1
TTC3	6	-0.3688625	1.55E-07	0.00282285	0.609	0.828
ZBTB20	6	-0.3692981	6.72E-09	0.00012257	0.202	0.495
ERGIC3	6	-0.3695296	2.74E-07	0.00500583	0.802	0.869
RPL10	6	-0.3698007	5.94E-19	1.08E-14	1	1
COX6C	6	-0.3701184	5.77E-07	0.01052469	0.852	0.949
SRP14	6	-0.3709529	6.04E-11	1.10E-06	0.988	0.99
PRDX5	6	-0.3729313	1.27E-11	2.32E-07	0.877	0.97
RPL8	6	-0.3762298	7.42E-23	1.35E-18	1	1

EID1	6	-0.377605	1.51E-09	2.76E-05	0.93	0.97
RPL39	6	-0.3782852	2.36E-15	4.31E-11	1	1
CTSK	6	-0.3785045	1.54E-08	0.00028157	0.243	0.515
HNRNPA1	6	-0.378762	1.11E-08	0.00020283	0.988	0.98
HLA-B	6	-0.379622	1.50E-07	0.00273575	0.778	0.929
BEX2	6	-0.3804714	1.24E-07	0.00226992	0.202	0.465
RPL13A	6	-0.3805484	9.29E-23	1.70E-18	1	1
RPS27A	6	-0.3825875	1.39E-18	2.54E-14	1	1
CFL1	6	-0.3827139	5.91E-09	0.00010779	0.749	0.909
TUBB	6	-0.3830113	1.23E-06	0.02249609	0.72	0.859
MIA	6	-0.386685	1.91E-08	0.00034859	0.988	0.99
SSR3	6	-0.3880275	1.59E-06	0.02906928	0.807	0.899
SERF2	6	-0.3889334	1.53E-14	2.80E-10	1	1
NDUFA3	6	-0.3936148	6.09E-08	0.00111048	0.444	0.707
HMGB1	6	-0.3959218	1.23E-07	0.00225219	0.683	0.869
NREP	6	-0.3976981	9.32E-10	1.70E-05	0.148	0.434
LITAF	6	-0.3989504	1.63E-07	0.00298291	0.453	0.677
LINC01503	6	-0.4033765	3.45E-07	0.00629644	0.218	0.475
S100A1	6	-0.4035903	1.69E-09	3.08E-05	1	1
SERPINA5	6	-0.4045077	2.47E-08	0.00045126	0.465	0.747
PFN1	6	-0.4070313	2.52E-09	4.59E-05	0.922	0.96
IDI1	6	-0.4112388	2.46E-06	0.04493982	0.226	0.455
PPT1	6	-0.4120693	1.41E-07	0.00257478	0.313	0.566
GPX4	6	-0.4124338	6.76E-10	1.23E-05	0.996	0.98
RPS2	6	-0.4126948	1.81E-21	3.31E-17	1	1
RPS23	6	-0.4138807	4.45E-19	8.12E-15	1	1
GNAS	6	-0.4167647	3.91E-09	7.14E-05	0.551	0.758
RPL23	6	-0.4172231	5.78E-17	1.05E-12	0.996	1
TMBIM6	6	-0.4227431	2.15E-06	0.03931306	0.7	0.808
NDUFA11	6	-0.4228652	5.52E-11	1.01E-06	0.84	0.929
BSG	6	-0.4234165	3.08E-08	0.00056174	0.609	0.818
COL5A2	6	-0.4240087	1.31E-06	0.02391264	0.374	0.606
RPS18	6	-0.4243853	3.55E-20	6.48E-16	1	1
NINJ1	6	-0.4246539	3.28E-07	0.00597975	0.481	0.707
SEC11A	6	-0.4257308	2.86E-12	5.22E-08	0.815	0.919
DBI	6	-0.4309586	5.14E-10	9.39E-06	0.7	0.869
HLA-C	6	-0.4312953	3.18E-10	5.81E-06	0.868	0.97
RPL23A	6	-0.4314866	1.17E-22	2.14E-18	1	1
RPL18	6	-0.431757	2.13E-20	3.89E-16	1	1

MOXD1	6	-0.4349421	7.21E-09	0.00013152	0.239	0.515
PLXDC2	6	-0.4356205	2.61E-08	0.00047655	0.428	0.667
UBA52	6	-0.4372263	1.60E-23	2.92E-19	1	1
MYL12B	6	-0.4398328	1.77E-10	3.24E-06	0.551	0.788
HERPUD1	6	-0.4398665	6.44E-08	0.00117451	0.444	0.697
RPS29	6	-0.4400064	7.30E-21	1.33E-16	1	1
INHBA	6	-0.4425795	7.72E-07	0.01409161	0.255	0.495
CITED2	6	-0.4448791	2.88E-07	0.00526063	0.333	0.586
RHOBTB3	6	-0.4470436	1.10E-09	2.01E-05	0.387	0.697
PEBP1	6	-0.4470497	3.02E-11	5.51E-07	0.749	0.949
NGFRAP1	6	-0.4485388	3.54E-10	6.45E-06	0.84	0.96
PPIB	6	-0.4510198	1.65E-07	0.00300767	0.938	0.939
PCBP2	6	-0.4511803	1.10E-13	2.00E-09	0.844	0.97
RPL27	6	-0.4522896	6.41E-19	1.17E-14	1	1
SSR4	6	-0.4525526	4.30E-12	7.84E-08	0.975	0.99
EIF3L	6	-0.4536367	4.80E-13	8.77E-09	0.798	0.949
RPL37	6	-0.4596037	5.65E-22	1.03E-17	1	1
PDIA3	6	-0.4596867	2.76E-07	0.00503419	0.63	0.818
H3F3A	6	-0.4649732	9.23E-14	1.68E-09	0.951	1
POLR2L	6	-0.4652205	1.08E-08	0.00019648	0.86	0.939
RPL30	6	-0.4652617	8.25E-20	1.51E-15	1	1
SNAI2	6	-0.4680425	1.54E-06	0.02817015	0.416	0.646
MALL	6	-0.4688347	1.09E-07	0.00199693	0.23	0.485
RPS12	6	-0.4709264	1.05E-19	1.92E-15	1	1
EEF2	6	-0.4722025	1.44E-09	2.63E-05	0.979	1
PRDX4	6	-0.4748116	7.35E-09	0.00013418	0.794	0.929
CCNI	6	-0.4786699	1.65E-17	3.02E-13	0.996	1
RPL34	6	-0.4788297	5.04E-24	9.20E-20	1	1
LGALS1	6	-0.4818971	7.98E-10	1.46E-05	0.979	0.99
RPS3A	6	-0.4856449	4.84E-26	8.83E-22	1	1
FHL1	6	-0.4867458	6.36E-08	0.00116091	0.288	0.556
HINT1	6	-0.4880062	1.04E-15	1.89E-11	0.971	1
TIMP2	6	-0.4889739	2.52E-08	0.00046031	0.469	0.707
TMED10	6	-0.4911493	2.93E-13	5.34E-09	0.84	0.97
PDE4DIP	6	-0.4924765	1.29E-14	2.35E-10	0.239	0.626
UQCRH	6	-0.4936806	2.86E-14	5.22E-10	0.909	0.97
PLPP1	6	-0.4959547	4.00E-11	7.29E-07	0.14	0.455
CD9	6	-0.4965498	1.49E-10	2.71E-06	0.564	0.808
NUCB2	6	-0.4989827	5.46E-08	0.00099541	0.399	0.636

UQCRB	6	-0.4997213	2.83E-12	5.17E-08	0.959	0.98
RPS16	6	-0.5012163	1.86E-27	3.39E-23	1	1
RPLP1	6	-0.5026455	4.45E-28	8.11E-24	1	1
SDC2	6	-0.5071288	2.45E-10	4.46E-06	0.852	0.949
CCDC102B	6	-0.5144116	5.97E-21	1.09E-16	0.021	0.394
C2orf82	6	-0.5154792	8.30E-08	0.0015138	0.963	0.99
ACTG1	6	-0.5292105	3.92E-08	0.00071573	0.951	0.99
SLC14A1	6	-0.5354279	7.93E-11	1.45E-06	0.263	0.576
CXXC5	6	-0.5356487	2.10E-12	3.83E-08	0.206	0.566
IGFBP7	6	-0.536863	1.82E-07	0.00332918	0.914	0.96
EEF1B2	6	-0.5379389	1.38E-19	2.52E-15	0.988	1
CD99	6	-0.5385906	1.92E-16	3.51E-12	0.93	1
RABAC1	6	-0.5387811	2.34E-15	4.28E-11	0.733	0.949
DNM3OS	6	-0.5394904	2.78E-14	5.08E-10	0.189	0.596
RPSA	6	-0.5396106	4.93E-18	9.00E-14	1	1
ANXA1	6	-0.5410717	3.14E-08	0.00057231	0.716	0.879
MSMO1	6	-0.5417172	2.31E-06	0.04209582	0.243	0.465
PDIA6	6	-0.5420303	2.56E-10	4.68E-06	0.663	0.879
MFGE8	6	-0.5492806	1.87E-09	3.42E-05	0.831	0.98
RPL7	6	-0.5494271	3.99E-23	7.27E-19	1	1
ARHGAP29	6	-0.5547631	3.32E-08	0.00060554	0.416	0.636
SLC40A1	6	-0.5548297	7.11E-13	1.30E-08	0.099	0.414
XAGE2	6	-0.5581717	9.47E-12	1.73E-07	0	0.182
HSPB1	6	-0.5607445	2.04E-12	3.71E-08	0.63	0.869
EEF1A1	6	-0.5660655	2.44E-23	4.45E-19	1	1
ARPC1B	6	-0.5746809	3.99E-10	7.29E-06	0.379	0.687
NUPR1	6	-0.5747047	3.39E-10	6.19E-06	0.955	0.97
ITM2A	6	-0.5765214	3.65E-13	6.66E-09	0.42	0.778
RPN2	6	-0.5786542	2.39E-14	4.37E-10	0.198	0.586
RPS17	6	-0.5791962	8.96E-27	1.64E-22	1	1
OAF	6	-0.58248	7.47E-12	1.36E-07	0.169	0.495
RPL10A	6	-0.587883	2.75E-25	5.02E-21	1	1
COL11A1	6	-0.5929594	1.35E-07	0.00245794	0.757	0.879
SLPI	6	-0.5996577	3.48E-08	0.00063411	0.984	1
RPS8	6	-0.6024251	7.10E-30	1.29E-25	1	1
TPT1	6	-0.6084143	1.12E-31	2.04E-27	1	1
MYL6	6	-0.609919	1.29E-18	2.35E-14	0.951	0.99
RPLP2	6	-0.6101469	7.05E-34	1.29E-29	1	1
CRISPLD1	6	-0.6102595	5.54E-15	1.01E-10	0.07	0.404

FBXO2	6	-0.61511	5.44E-15	9.92E-11	0.691	0.939
YBX1	6	-0.6276513	2.19E-21	3.99E-17	0.959	1
PTRF	6	-0.6283373	7.33E-11	1.34E-06	0.432	0.687
MYLK	6	-0.6301937	1.55E-15	2.82E-11	0.148	0.535
RPL4	6	-0.6312718	1.51E-29	2.76E-25	0.996	1
TCF4	6	-0.6326605	3.62E-12	6.60E-08	0.226	0.566
GPX3	6	-0.648077	2.71E-14	4.94E-10	0.942	0.97
NPC2	6	-0.6482994	4.26E-22	7.77E-18	0.951	1
TSPO	6	-0.663149	1.44E-21	2.63E-17	0.737	0.96
YBX3	6	-0.6665675	7.06E-24	1.29E-19	0.745	0.97
CRYAB	6	-0.6686315	3.28E-12	5.99E-08	0.708	0.929
IGFBP6	6	-0.6779576	6.79E-11	1.24E-06	0.704	0.859
PAX1	6	-0.6996473	9.29E-17	1.69E-12	0.535	0.848
CRTAP	6	-0.7146762	4.80E-18	8.76E-14	0.494	0.828
PTMS	6	-0.7164707	6.78E-24	1.24E-19	0.465	0.929
ID3	6	-0.7341354	1.40E-09	2.55E-05	0.362	0.687
RPLP0	6	-0.7349579	2.34E-36	4.27E-32	1	1
P4HB	6	-0.7414377	1.16E-15	2.12E-11	0.852	0.97
MT-CO2	6	-0.7693312	7.87E-10	1.44E-05	0.955	0.96
B2M	6	-0.7845565	8.33E-26	1.52E-21	0.988	1
ZFP36L2	6	-0.789923	8.77E-15	1.60E-10	0.181	0.556
MGP	6	-0.7902949	3.32E-09	6.06E-05	0.996	1
MT-CO1	6	-0.7929383	2.21E-10	4.04E-06	0.951	0.939
APOD	6	-0.7952874	1.26E-14	2.30E-10	0.202	0.586
EIF3H	6	-0.8055348	9.99E-26	1.82E-21	0.922	1
THY1	6	-0.8055683	3.65E-15	6.66E-11	0.292	0.687
ISLR	6	-0.8160906	3.05E-18	5.57E-14	0.35	0.747
PPIC	6	-0.8199076	2.87E-18	5.23E-14	0.296	0.747
MT-ND1	6	-0.8250029	6.30E-13	1.15E-08	0.519	0.828
CRIP2	6	-0.8278492	2.79E-25	5.08E-21	0.276	0.798
TSC22D1	6	-0.8434168	5.67E-07	0.01033701	0.765	0.909
FTH1	6	-0.8647495	2.36E-25	4.30E-21	1	1
MT-CO3	6	-0.8744111	5.50E-11	1.00E-06	0.848	0.939
OGN	6	-0.9406831	1.93E-16	3.51E-12	0.901	0.99
MT-ND2	6	-0.9500981	1.08E-12	1.98E-08	0.613	0.838
MT-ATP6	6	-0.9533661	3.01E-15	5.49E-11	0.749	0.909
PLAC9	6	-0.9827619	1.68E-20	3.07E-16	0.733	0.939
RPS11	6	-1.002838	3.21E-42	5.85E-38	0.979	1
RPS20	6	-1.0147113	1.76E-43	3.21E-39	0.992	1

MT-ND3	6	-1.0294742	1.14E-14	2.08E-10	0.597	0.828
MT-CYB	6	-1.0725581	1.07E-17	1.95E-13	0.556	0.848
CYR61	6	-1.1110348	1.05E-19	1.92E-15	0.222	0.697
CAPS	6	-1.1205152	1.65E-23	3.01E-19	0.683	0.97
ADIRF	6	-1.1323792	3.72E-28	6.78E-24	0.333	0.879
NEAT1	6	-1.1702245	2.54E-24	4.63E-20	0.963	1
CLEC3A	6	-1.2737309	1.22E-21	2.22E-17	0.383	0.838
SPARC	6	-1.278278	4.63E-26	8.45E-22	0.506	0.929
SPTSSB	6	-1.4602904	1.30E-40	2.37E-36	0.025	0.677
FRZB	6	-1.4762204	7.27E-24	1.33E-19	0.251	0.778
CTGF	6	-1.4874391	3.75E-17	6.84E-13	0.362	0.828
COL2A1	6	-1.5596144	3.02E-26	5.50E-22	0.568	0.96
**gene**	**cluster**	**avg_logFC**	**p_val**	**p_val_adj**	**pct.1**	**pct.2**
PLA2G2A	7	1.59159912	2.42E-16	4.42E-12	0.718	0.306
CTHRC1	7	1.56383792	5.57E-30	1.02E-25	0.889	0.376
TGFBI	7	1.38947724	1.09E-28	1.99E-24	0.897	0.422
COL1A1	7	1.35985487	2.84E-09	5.18E-05	0.496	0.197
SPP1	7	1.04526268	4.40E-22	8.02E-18	0.547	0.052
CYP1B1	7	0.95564746	9.26E-10	1.69E-05	0.598	0.283
CHI3L2	7	0.87633948	4.48E-12	8.18E-08	0.65	0.347
COL3A1	7	0.86395817	1.69E-08	0.00030907	0.897	0.775
PHLDA1	7	0.81600085	5.56E-12	1.01E-07	0.538	0.179
TIMP1	7	0.79725594	1.68E-12	3.06E-08	1	0.988
MMP2	7	0.71526941	3.34E-08	0.00060981	0.197	0.012
ADAMTS6	7	0.67993031	5.64E-15	1.03E-10	0.607	0.197
LTBP1	7	0.67141962	7.35E-17	1.34E-12	0.581	0.15
TNC	7	0.65871297	3.94E-10	7.19E-06	0.632	0.324
TNFRSF11B	7	0.64571401	3.13E-08	0.00057189	0.684	0.428
COMP	7	0.64512332	4.33E-07	0.00790482	0.812	0.595
VMP1	7	0.63322222	1.13E-09	2.07E-05	0.735	0.52
EPYC	7	0.60903016	1.48E-09	2.70E-05	0.521	0.214
PPP3CA	7	0.60202735	9.84E-10	1.80E-05	0.897	0.746
MT1X	7	0.58451426	1.13E-06	0.02058364	1	0.936
COL1A2	7	0.58156034	8.73E-07	0.01592634	0.923	0.85
IL11	7	0.51827284	9.11E-07	0.01662441	0.179	0.017
PTGES	7	0.50775456	1.34E-10	2.45E-06	0.41	0.098
CRLF1	7	0.48841657	5.42E-10	9.90E-06	0.701	0.393
FN1	7	0.47544335	1.43E-09	2.61E-05	0.991	1
CYTL1	7	0.4722432	3.76E-10	6.86E-06	0.812	0.451

ITM2B	7	0.4413074	2.30E-08	0.00041901	0.983	0.925
BAALC	7	0.41282394	2.60E-06	0.0474943	0.692	0.532
PRRX2	7	0.4047853	4.56E-09	8.32E-05	0.53	0.225
SLC25A37	7	0.39111766	1.89E-06	0.03448445	0.769	0.584
SOD2	7	0.37326004	1.05E-07	0.0019122	0.761	0.59
AEBP1	7	0.37221351	1.13E-07	0.00205553	0.923	0.769
GJA1	7	0.36979489	4.36E-07	0.00795686	0.547	0.266
BMP2	7	0.3686448	5.34E-07	0.00975159	0.231	0.04
DUSP4	7	0.36356771	5.51E-09	0.00010048	0.308	0.058
PDE4B	7	0.3336606	6.32E-08	0.00115387	0.385	0.121
CYGB	7	0.30842594	4.84E-08	0.00088332	0.316	0.075
TREM1	7	0.30823	4.51E-13	8.23E-09	0.316	0.017
SMIM3	7	0.29891147	2.24E-08	0.00040815	0.376	0.11
LEF1	7	0.29748205	9.33E-10	1.70E-05	0.274	0.029
SAMD11	7	0.29630987	4.88E-08	0.00089113	0.385	0.116
TNFSF11	7	0.26405878	6.20E-08	0.00113057	0.205	0.017
ARFGEF3	7	-0.2507362	1.31E-07	0.00239136	0.026	0.26
CHCHD2	7	-0.2508147	1.19E-06	0.02174942	0.991	1
DHCR7	7	-0.2528393	1.43E-07	0.00260618	0.051	0.306
PLA2G16	7	-0.2566609	3.21E-07	0.00585554	0.051	0.295
DAP	7	-0.2570781	1.57E-06	0.0285621	0.701	0.89
RPL3	7	-0.2572936	6.51E-09	0.00011886	1	1
PPDPF	7	-0.2598489	7.65E-09	0.00013957	1	1
RPS17	7	-0.2622806	5.19E-11	9.47E-07	1	1
SRGAP1	7	-0.2640021	6.03E-07	0.01099569	0.12	0.387
CYB5D2	7	-0.2644183	7.22E-07	0.01316536	0.231	0.52
AC003090.1	7	-0.2644468	1.22E-06	0.02227536	0.043	0.26
RPL31	7	-0.2668103	1.37E-09	2.51E-05	1	1
SYNGR1	7	-0.2676619	2.61E-07	0.00476104	0.214	0.52
RP4-621F18.2	7	-0.2683815	1.07E-06	0.01959792	0.145	0.41
RPL23A	7	-0.268634	1.81E-12	3.30E-08	1	1
TOMM7	7	-0.2695942	5.04E-10	9.19E-06	0.991	1
EDF1	7	-0.2727591	1.95E-07	0.00355109	0.949	0.988
PDXK	7	-0.2755379	1.48E-06	0.02697669	0.256	0.538
RPS8	7	-0.2757736	3.91E-12	7.13E-08	1	1
ENKUR	7	-0.2764962	2.25E-08	0.00041077	0.017	0.266
PXDN	7	-0.2775955	6.43E-07	0.01173964	0.205	0.491
OAZ1	7	-0.2793274	4.46E-07	0.00813041	1	1
SRP14	7	-0.2798499	9.48E-08	0.00173026	0.991	0.994

YIPF2	7	-0.2836322	2.56E-06	0.04670799	0.427	0.717
RPS3A	7	-0.2849616	1.49E-12	2.72E-08	1	1
RPS2	7	-0.285482	7.99E-11	1.46E-06	1	1
CCNI	7	-0.2893669	1.91E-06	0.03487109	0.983	0.994
TSPAN5	7	-0.2908562	4.94E-08	0.00090207	0.137	0.439
PLCD3	7	-0.2944249	4.28E-08	0.0007811	0.12	0.416
GTF3A	7	-0.2953702	2.21E-06	0.04034002	0.556	0.786
RPL30	7	-0.2966387	3.58E-14	6.54E-10	1	1
RBM39	7	-0.2981156	1.28E-06	0.02343124	0.684	0.844
ARSJ	7	-0.2984771	3.01E-07	0.00549755	0.188	0.468
PGAM1	7	-0.3008468	1.61E-06	0.02928792	0.855	0.948
RPL37	7	-0.302239	7.02E-15	1.28E-10	1	1
GPX4	7	-0.3047881	1.07E-07	0.00194854	0.983	0.988
PRPSAP1	7	-0.3054251	8.38E-09	0.00015285	0.197	0.543
PCBP2	7	-0.3060537	4.28E-09	7.82E-05	0.949	1
DBI	7	-0.3067244	1.55E-06	0.02821027	0.821	0.965
GNB2L1	7	-0.3094645	1.10E-08	0.00019998	1	1
DNAJC4	7	-0.3113032	3.08E-09	5.62E-05	0.282	0.653
CCSER2	7	-0.3114784	3.68E-07	0.00670947	0.274	0.566
NQO1	7	-0.3121819	4.77E-07	0.00870502	0.197	0.48
C10orf54	7	-0.3141509	5.39E-08	0.00098288	0.051	0.312
RPA3	7	-0.3151703	7.26E-07	0.01325151	0.419	0.682
ANXA11	7	-0.3160158	1.84E-07	0.0033622	0.171	0.462
ZNF706	7	-0.3168446	8.65E-07	0.01578885	0.513	0.751
RPL23	7	-0.3175204	2.40E-12	4.38E-08	1	1
FLNA	7	-0.3187803	8.24E-07	0.01502826	0.231	0.503
FUS	7	-0.319124	2.70E-06	0.04924918	0.487	0.751
ODC1	7	-0.3196047	4.61E-08	0.00084182	0.162	0.474
RPL14	7	-0.3218197	1.85E-13	3.38E-09	1	1
ARL2BP	7	-0.3218321	1.27E-06	0.02322165	0.59	0.757
ALDOA	7	-0.3224616	3.66E-09	6.68E-05	0.983	1
KDELR1	7	-0.3236202	2.47E-06	0.04504308	0.667	0.821
AFF3	7	-0.3240084	2.81E-08	0.00051338	0.12	0.422
CYSTM1	7	-0.325019	2.32E-06	0.04224881	0.368	0.624
FTH1	7	-0.32838	2.11E-10	3.86E-06	1	1
EIF3K	7	-0.3314723	1.12E-06	0.02040458	0.667	0.855
ZIC1	7	-0.3332279	3.10E-08	0.000565	0.154	0.474
GNAS	7	-0.3340555	9.32E-08	0.00170003	0.795	0.913
ZCRB1	7	-0.3341707	4.41E-08	0.00080486	0.504	0.757

RPS16	7	-0.3362567	1.22E-16	2.22E-12	1	1
COPZ2	7	-0.3365507	3.71E-07	0.00676621	0.564	0.838
LPIN1	7	-0.3376205	1.20E-06	0.02187638	0.171	0.422
TPT1	7	-0.3385573	5.36E-14	9.77E-10	1	1
ATP5A1	7	-0.3424631	7.79E-07	0.01420827	0.521	0.723
MGLL	7	-0.3425967	4.39E-07	0.00801672	0.231	0.526
C1orf21	7	-0.3442718	2.60E-07	0.00474988	0.479	0.751
CAPNS1	7	-0.3464813	4.12E-07	0.00750866	0.402	0.665
GPC6	7	-0.3479868	2.50E-08	0.0004562	0.496	0.792
CALM3	7	-0.3485495	1.55E-06	0.02832604	0.667	0.815
OTOS	7	-0.3487166	4.79E-09	8.75E-05	0.111	0.439
GPC1	7	-0.3491763	8.33E-10	1.52E-05	0.214	0.566
CD81	7	-0.3494318	2.46E-06	0.04485053	0.513	0.699
CETN2	7	-0.3508197	3.84E-08	0.00070021	0.419	0.705
RBM3	7	-0.351175	5.12E-08	0.00093431	0.615	0.844
NKX3-2	7	-0.3525818	6.49E-07	0.01184208	0.137	0.399
RPL4	7	-0.3579676	1.33E-13	2.43E-09	1	1
MXD4	7	-0.3579882	2.89E-08	0.00052683	0.188	0.486
MLEC	7	-0.360806	1.47E-07	0.00268963	0.564	0.821
PDE4DIP	7	-0.368969	3.73E-08	0.0006806	0.462	0.74
SSR2	7	-0.371886	3.07E-09	5.60E-05	0.846	0.983
NFIC	7	-0.3747511	2.34E-08	0.00042729	0.59	0.815
ZBTB20	7	-0.3769926	4.16E-09	7.60E-05	0.299	0.647
BTF3	7	-0.3772072	8.04E-13	1.47E-08	0.983	1
DSTN	7	-0.3786309	4.97E-11	9.07E-07	1	1
HDLBP	7	-0.3795878	2.73E-09	4.98E-05	0.709	0.89
ITGA10	7	-0.3834208	4.68E-07	0.00853213	0.325	0.618
FTL	7	-0.3839556	3.00E-11	5.47E-07	1	1
GSTP1	7	-0.3860092	4.72E-08	0.00086182	0.923	0.965
ARPC1B	7	-0.3861456	2.09E-06	0.03821425	0.821	0.96
SLC25A3	7	-0.3886926	9.29E-09	0.00016954	0.812	0.919
MFGE8	7	-0.3888187	3.36E-07	0.00612797	0.752	0.908
PEBP1	7	-0.3889034	1.74E-09	3.18E-05	0.761	0.908
TSPO	7	-0.3902439	7.03E-10	1.28E-05	0.906	0.977
MYL6	7	-0.3909088	1.09E-06	0.01997645	0.983	0.988
TSPAN4	7	-0.39211	3.19E-07	0.00582943	0.641	0.844
C1QTNF8	7	-0.3942	4.49E-09	8.19E-05	0.222	0.538
MOXD1	7	-0.3954307	6.65E-07	0.01213437	0.197	0.462
RPL18	7	-0.3969475	6.31E-18	1.15E-13	1	1

AHNAK2	7	-0.3973397	2.76E-08	0.00050398	0.231	0.543
SPARC	7	-0.3980107	9.59E-09	0.000175	0.932	0.994
CXXC5	7	-0.4000672	1.91E-08	0.00034939	0.556	0.803
TKT	7	-0.4044911	4.68E-08	0.0008548	0.35	0.676
KHDRBS3	7	-0.4048869	3.29E-10	6.00E-06	0.026	0.329
YBX1	7	-0.4054079	3.56E-11	6.50E-07	0.983	1
MALAT1	7	-0.405487	3.21E-12	5.85E-08	1	1
S100A6	7	-0.4056118	2.36E-08	0.00043138	1	1
NFIA	7	-0.4065422	2.26E-11	4.13E-07	0.368	0.763
HEBP2	7	-0.4085522	5.43E-08	0.00099029	0.624	0.867
SLC9A3R2	7	-0.4085728	4.00E-08	0.0007298	0.316	0.618
CALR	7	-0.4124928	1.30E-07	0.00237847	0.863	0.948
ARHGAP29	7	-0.4131113	6.03E-09	0.00010997	0.359	0.665
MMP24-AS1	7	-0.4141468	4.54E-09	8.28E-05	0.573	0.832
ATRNL1	7	-0.4154966	3.37E-11	6.15E-07	0.094	0.457
AHNAK	7	-0.4159738	2.21E-07	0.00403082	0.701	0.827
RHOA	7	-0.4171565	1.29E-11	2.35E-07	0.846	0.954
C14orf1	7	-0.4204473	2.25E-08	0.00040968	0.359	0.671
FXYD5	7	-0.4208907	2.70E-08	0.00049227	0.812	0.942
AP2M1	7	-0.4228344	1.53E-09	2.79E-05	0.624	0.908
HSPB1	7	-0.4285287	2.45E-08	0.00044694	0.829	0.908
NDUFA11	7	-0.4311147	2.29E-12	4.18E-08	0.906	0.983
HSPB6	7	-0.4328398	2.03E-09	3.70E-05	0.274	0.618
LGALS1	7	-0.4330716	4.95E-07	0.00904099	1	1
RPL7	7	-0.4374891	4.70E-18	8.57E-14	1	1
OSBPL9	7	-0.4392306	3.02E-07	0.00551506	0.265	0.532
IGFBP6	7	-0.4395478	1.25E-07	0.00228422	0.769	0.936
MT-ND5	7	-0.4416154	4.74E-09	8.65E-05	0.658	0.832
NUCB2	7	-0.444204	7.15E-09	0.00013042	0.487	0.798
CRISPLD1	7	-0.4463922	6.80E-08	0.00124059	0.12	0.399
HRCT1	7	-0.4493542	7.52E-09	0.00013731	0.077	0.37
RPSA	7	-0.4498986	1.54E-16	2.81E-12	1	1
SPINT2	7	-0.4499497	3.77E-09	6.89E-05	0.154	0.474
PAX1	7	-0.4505021	8.56E-07	0.01562294	0.718	0.838
TPM2	7	-0.4516919	8.30E-10	1.51E-05	0.735	0.919
S100A10	7	-0.4541774	8.41E-08	0.00153535	0.983	0.994
S100A1	7	-0.4545774	7.02E-07	0.01281422	0.949	0.988
SCD	7	-0.4680147	5.30E-07	0.00967018	0.308	0.595
ELL2	7	-0.4715304	5.51E-11	1.01E-06	0.282	0.642

HCFC1R1	7	-0.4879381	2.11E-10	3.86E-06	0.889	0.988
METRN	7	-0.4885091	1.73E-10	3.16E-06	0.427	0.757
ACTG1	7	-0.4886478	1.20E-07	0.00219677	0.906	0.971
PDLIM2	7	-0.4971416	8.54E-12	1.56E-07	0.692	0.873
HINT1	7	-0.4977741	9.84E-17	1.80E-12	0.94	1
GAPDH	7	-0.5004331	5.45E-13	9.94E-09	1	1
LINC01503	7	-0.5010755	1.39E-06	0.02527336	0.308	0.555
CKAP4	7	-0.5093177	1.07E-10	1.95E-06	0.59	0.85
NPDC1	7	-0.5121389	2.15E-15	3.92E-11	0.171	0.63
EEF2	7	-0.5181644	1.39E-09	2.53E-05	0.966	1
TMEM106C	7	-0.5226699	1.07E-13	1.95E-09	0.299	0.711
TBL1XR1	7	-0.5279599	1.26E-08	0.0002304	0.547	0.798
NINJ1	7	-0.5299808	2.20E-14	4.02E-10	0.282	0.723
NGFRAP1	7	-0.5303287	1.42E-14	2.59E-10	0.932	0.994
P3H2	7	-0.533423	7.25E-11	1.32E-06	0.282	0.665
RPLP0	7	-0.5389845	3.61E-29	6.59E-25	1	1
DAB2	7	-0.5398792	3.34E-09	6.09E-05	0.521	0.78
PKM	7	-0.5415893	1.52E-13	2.77E-09	0.915	0.983
C2orf82	7	-0.541819	2.85E-07	0.0051925	0.846	0.919
CCDC102B	7	-0.5492706	1.59E-15	2.89E-11	0.051	0.491
CRYAB	7	-0.5494054	1.32E-07	0.00240783	0.761	0.913
LOXL1	7	-0.5653795	4.91E-08	0.00089577	0.282	0.561
CLU	7	-0.5655528	1.47E-06	0.02687245	0.957	0.988
FBXO2	7	-0.5695954	2.80E-09	5.12E-05	0.641	0.85
HIST1H1C	7	-0.57507	1.96E-09	3.57E-05	0.085	0.399
MAGED1	7	-0.5769182	2.69E-12	4.91E-08	0.35	0.757
EEF1A1	7	-0.5780714	2.93E-23	5.35E-19	1	1
PTRF	7	-0.5921126	1.03E-16	1.88E-12	0.675	0.919
SPTLC3	7	-0.5935861	5.82E-08	0.00106134	0.299	0.566
COL14A1	7	-0.6046372	6.02E-11	1.10E-06	0.111	0.468
NEAT1	7	-0.6219931	2.97E-12	5.42E-08	1	1
ITGA11	7	-0.6478822	1.50E-16	2.74E-12	0.197	0.659
COL8A1	7	-0.6618491	7.89E-11	1.44E-06	0.077	0.416
MT-CO2	7	-0.6783066	3.10E-20	5.66E-16	0.957	0.988
SDC2	7	-0.6813995	1.49E-17	2.72E-13	0.872	0.983
RPS11	7	-0.6966022	3.24E-34	5.91E-30	0.991	1
FRZB	7	-0.7242081	2.54E-06	0.04639055	0.239	0.509
SPTSSB	7	-0.7272414	1.53E-09	2.79E-05	0	0.266
CRIP2	7	-0.7323406	3.60E-17	6.57E-13	0.47	0.85

DOK1	7	-0.7348936	5.60E-10	1.02E-05	0.333	0.676
MT-ND4	7	-0.7575953	5.07E-28	9.24E-24	0.949	0.994
CITED2	7	-0.7871026	2.26E-12	4.12E-08	0.35	0.694
ZFP36L2	7	-0.8062465	1.15E-11	2.09E-07	0.248	0.63
EIF3H	7	-0.8304323	2.41E-25	4.39E-21	0.829	0.96
PLPP1	7	-0.8309994	2.83E-18	5.17E-14	0.179	0.676
COL2A1	7	-0.8385229	2.01E-07	0.00366235	0.897	0.942
RPS20	7	-0.839652	2.06E-39	3.77E-35	1	1
PRSS23	7	-0.8755707	2.20E-09	4.02E-05	0.632	0.838
THY1	7	-0.8947854	3.42E-15	6.24E-11	0.812	0.983
CAPS	7	-0.9014849	1.45E-18	2.65E-14	0.573	0.936
MT-ATP6	7	-0.911867	4.29E-29	7.83E-25	0.923	0.988
CYR61	7	-0.9391886	1.99E-12	3.64E-08	0.615	0.873
VIM	7	-0.9413073	8.29E-15	1.51E-10	1	1
MT-ND1	7	-0.9721355	2.57E-29	4.69E-25	0.803	0.988
MT-CO3	7	-0.9865317	3.88E-34	7.08E-30	0.94	0.988
MT-ND3	7	-1.018359	2.00E-36	3.64E-32	0.872	0.988
MT-CO1	7	-1.0224252	2.58E-35	4.71E-31	0.949	0.994
TPM1	7	-1.0652244	1.05E-23	1.91E-19	0.718	0.965
FHL1	7	-1.0913335	1.82E-20	3.32E-16	0.299	0.792
MT-CYB	7	-1.1673559	7.31E-33	1.33E-28	0.838	0.988
MT-ND2	7	-1.2439353	1.40E-36	2.56E-32	0.752	0.988
ADIRF	7	-1.3064904	7.58E-26	1.38E-21	0.385	0.908
CTGF	7	-1.5748348	2.35E-12	4.29E-08	0.667	0.85
MSMP	7	-1.8139589	2.32E-12	4.24E-08	0.248	0.636
**gene**	**cluster**	**avg_logFC**	**p_val**	**p_val_adj**	**pct.1**	**pct.2**
PLA2G2A	8	1.94419782	4.04E-28	7.38E-24	0.838	0.338
CHI3L2	8	1.36634745	2.06E-33	3.77E-29	0.87	0.384
EPYC	8	1.2177949	6.12E-33	1.12E-28	0.792	0.197
CCDC80	8	1.20939086	9.71E-37	1.77E-32	0.994	0.939
FN1	8	1.17927638	8.35E-43	1.52E-38	1	1
MT1G	8	1.11573937	2.55E-14	4.65E-10	0.987	0.894
TGFBI	8	1.0757038	4.43E-31	8.09E-27	0.74	0.177
AEBP1	8	0.94446161	3.28E-25	5.98E-21	0.844	0.444
CHI3L1	8	0.91129795	1.04E-10	1.90E-06	0.929	0.854
CTHRC1	8	0.88575401	5.88E-23	1.07E-18	0.578	0.106
CYTL1	8	0.87450302	3.19E-11	5.83E-07	0.461	0.152
SPP1	8	0.87174494	9.07E-30	1.65E-25	0.623	0.061
MT1H	8	0.83463348	1.37E-07	0.00249836	0.74	0.51

PPP3CA	8	0.82640561	4.41E-20	8.06E-16	0.909	0.631
NOVA1	8	0.78728845	8.54E-21	1.56E-16	0.961	0.808
BTG1	8	0.75855019	3.32E-22	6.07E-18	0.994	0.859
MT1X	8	0.75246674	8.58E-16	1.57E-11	1	1
SOD2	8	0.75018772	2.39E-11	4.36E-07	0.883	0.667
VEGFA	8	0.74965366	1.11E-17	2.02E-13	0.669	0.273
CTSD	8	0.69560175	1.05E-07	0.00191207	0.968	0.944
TIMP1	8	0.67098202	4.23E-09	7.71E-05	0.981	1
PRG4	8	0.63890741	9.53E-16	1.74E-11	0.357	0.03
ANGPTL7	8	0.63316967	2.14E-09	3.90E-05	0.195	0.01
MMP3	8	0.63138609	1.01E-11	1.83E-07	0.825	0.495
SCRG1	8	0.56708226	1.59E-13	2.90E-09	1	1
DDIT4L	8	0.56639547	2.28E-12	4.17E-08	0.643	0.318
SMOC2	8	0.5582897	1.59E-12	2.90E-08	0.455	0.136
PTGS2	8	0.55721194	1.71E-16	3.12E-12	0.364	0.03
COX17	8	0.53844569	3.66E-15	6.68E-11	0.877	0.662
CD44	8	0.53055763	2.31E-10	4.21E-06	0.695	0.419
HILPDA	8	0.5278232	1.38E-09	2.51E-05	0.539	0.273
SLC25A37	8	0.52618014	5.69E-09	0.00010382	0.727	0.515
WWP2	8	0.51109211	2.65E-10	4.83E-06	0.857	0.727
TMSB4X	8	0.5066221	5.06E-09	9.23E-05	0.896	0.803
MT2A	8	0.50611108	2.65E-08	0.00048366	1	0.995
C7orf73	8	0.49139909	1.48E-19	2.70E-15	0.987	0.919
ADAMTS6	8	0.48986381	1.58E-10	2.89E-06	0.37	0.101
COL1A2	8	0.48745723	2.15E-09	3.92E-05	0.74	0.465
PAM	8	0.47316124	2.58E-13	4.71E-09	0.87	0.717
C4orf48	8	0.46654142	1.72E-09	3.14E-05	0.558	0.278
BAALC	8	0.46332614	3.54E-10	6.46E-06	0.571	0.278
SLC26A2	8	0.44399877	8.82E-10	1.61E-05	0.695	0.424
RNF145	8	0.44251507	1.10E-09	2.01E-05	0.63	0.394
LUM	8	0.43390278	1.36E-10	2.48E-06	1	1
SRGN	8	0.42073258	3.56E-07	0.0064978	0.468	0.242
CD59	8	0.41430743	2.08E-10	3.80E-06	0.974	0.944
SAMD11	8	0.40403552	5.97E-14	1.09E-09	0.279	0.01
SEPW1	8	0.3965635	1.96E-07	0.00358261	0.766	0.611
IFITM10	8	0.39556404	2.97E-08	0.00054171	0.591	0.338
CFD	8	0.3896191	1.97E-09	3.59E-05	0.26	0.04
HIF1A	8	0.38837731	3.32E-07	0.00605102	0.929	0.899
SERF2	8	0.38302192	3.13E-19	5.71E-15	1	1

ACAN	8	0.38272554	7.07E-13	1.29E-08	1	1
PNRC1	8	0.37900154	2.69E-10	4.90E-06	0.994	0.965
CRIP1	8	0.37843143	5.79E-09	0.00010557	0.695	0.379
MT1F	8	0.37759806	1.10E-07	0.00201063	0.994	0.864
IGFBP3	8	0.36560357	1.34E-08	0.00024394	0.292	0.066
CCND2-AS1	8	0.36398759	2.08E-11	3.80E-07	0.377	0.086
TSHZ2	8	0.35518129	5.37E-14	9.80E-10	0.292	0.015
C4orf3	8	0.34621317	2.65E-12	4.84E-08	1	0.985
CRLF1	8	0.34404491	6.89E-07	0.01258049	0.416	0.187
FAM162A	8	0.34390722	2.57E-07	0.0046975	0.929	0.783
TPD52	8	0.33116336	2.26E-06	0.04118211	0.734	0.596
CNPY2	8	0.3228302	3.75E-07	0.00684657	0.87	0.793
GJA1	8	0.2796182	1.10E-06	0.02012055	0.227	0.056
LTBP1	8	0.26598462	5.32E-10	9.70E-06	0.24	0.025
COL9A1	8	0.25608771	2.20E-07	0.00402055	0.227	0.045
RPLP1	8	-0.2519564	8.57E-18	1.56E-13	1	1
ARFGAP3	8	-0.2531417	3.87E-08	0.0007056	0.182	0.465
CLNS1A	8	-0.2534056	2.34E-06	0.04265259	0.37	0.611
RPL24	8	-0.2535642	1.74E-12	3.18E-08	1	1
DNAJC1	8	-0.2541493	4.51E-07	0.0082255	0.175	0.429
RPS14	8	-0.2550429	1.64E-18	3.00E-14	1	1
RPL11	8	-0.2565679	3.46E-16	6.31E-12	1	1
CDIPT	8	-0.2572229	7.12E-07	0.01299961	0.13	0.354
SOD1	8	-0.2582682	9.00E-07	0.01641392	0.903	0.98
RRAGD	8	-0.258334	7.69E-08	0.0014029	0.104	0.343
ACP1	8	-0.2602656	2.14E-06	0.03909016	0.474	0.692
SCFD1	8	-0.2626313	4.22E-07	0.00770493	0.201	0.46
C6orf48	8	-0.2648608	1.38E-06	0.02519968	0.89	0.955
GHITM	8	-0.2657176	1.65E-06	0.03005726	0.416	0.677
COL14A1	8	-0.2659421	1.05E-07	0.00190995	0.006	0.182
PPDPF	8	-0.2679688	5.66E-11	1.03E-06	1	1
COMTD1	8	-0.2680635	1.20E-09	2.19E-05	0.052	0.313
RPL36AL	8	-0.2695946	6.34E-10	1.16E-05	0.994	0.99
RPS12	8	-0.2697992	1.02E-15	1.87E-11	1	1
RAC1	8	-0.2708009	6.50E-07	0.01186056	0.734	0.949
FTL	8	-0.2710043	1.91E-10	3.48E-06	1	1
RPL27	8	-0.2739805	5.57E-15	1.02E-10	1	1
HMGN2	8	-0.2740233	1.82E-08	0.00033167	0.916	0.96
RPS28	8	-0.2742395	5.76E-20	1.05E-15	1	1

ATP5L	8	-0.2751931	9.69E-09	0.00017679	0.922	0.985
RPS6	8	-0.275861	1.15E-20	2.09E-16	1	1
PPIB	8	-0.2763518	1.41E-06	0.02580386	0.935	0.995
BEX2	8	-0.2768852	5.87E-08	0.00107113	0.162	0.449
NDUFC2	8	-0.277787	2.59E-06	0.04729564	0.597	0.803
RPL26	8	-0.2779035	2.80E-16	5.10E-12	1	1
RPL15	8	-0.2785862	5.93E-19	1.08E-14	1	1
RPS3	8	-0.2809142	3.82E-18	6.97E-14	1	1
VPS28	8	-0.2815834	8.97E-07	0.01636528	0.357	0.606
RRAGA	8	-0.2817119	3.28E-08	0.00059784	0.195	0.49
RPS9	8	-0.2820179	5.75E-17	1.05E-12	1	1
PCBP2	8	-0.282044	3.65E-08	0.00066685	0.948	0.99
SERTAD4	8	-0.2840523	1.45E-09	2.64E-05	0.045	0.298
HS3ST1	8	-0.2852135	1.45E-07	0.00264619	0.091	0.318
RPS4Y1	8	-0.286968	2.64E-10	4.81E-06	0.987	0.995
FDFT1	8	-0.2876821	3.02E-07	0.00551576	0.182	0.434
FAU	8	-0.2887848	6.01E-17	1.10E-12	1	1
COX5A	8	-0.2889935	1.86E-08	0.0003397	0.39	0.722
SQSTM1	8	-0.2906538	1.85E-07	0.00337941	0.708	0.904
HSPD1	8	-0.2938493	1.88E-07	0.00342835	0.338	0.621
ENPP1	8	-0.2947995	4.03E-07	0.00734618	0.37	0.646
NDUFA11	8	-0.2949078	5.70E-09	0.00010409	0.903	0.99
RPL39	8	-0.2950744	1.55E-15	2.82E-11	1	1
RPS15A	8	-0.295349	6.12E-23	1.12E-18	1	1
SNRPD2	8	-0.2955277	6.72E-09	0.00012261	0.851	0.955
RPL21	8	-0.2961152	2.52E-22	4.60E-18	1	1
RPL37A	8	-0.2966561	9.21E-24	1.68E-19	1	1
RPS25	8	-0.2979739	1.25E-17	2.28E-13	1	1
MPC2	8	-0.2982257	5.38E-08	0.00098255	0.331	0.621
RPL13	8	-0.2986732	4.03E-23	7.36E-19	1	1
RPL36	8	-0.2987974	3.44E-17	6.28E-13	1	1
APOA1BP	8	-0.2999522	7.91E-07	0.01444107	0.182	0.419
TMEM147	8	-0.3002145	8.53E-08	0.00155566	0.617	0.833
SLC38A1	8	-0.3003672	2.03E-07	0.00370961	0.357	0.626
CIRBP	8	-0.302378	1.18E-07	0.00215319	0.968	0.985
SLC25A5	8	-0.3037662	9.49E-08	0.00173144	0.351	0.611
IGBP1	8	-0.3037662	2.02E-06	0.03691078	0.338	0.561
EDF1	8	-0.3041103	5.99E-10	1.09E-05	0.961	0.99
RPL22	8	-0.30472	1.55E-14	2.83E-10	1	1

PRNP	8	-0.3076241	4.16E-07	0.00759813	0.253	0.515
MTDH	8	-0.3079033	5.84E-07	0.01065449	0.656	0.833
FADS1	8	-0.3090263	1.04E-07	0.00189141	0.175	0.434
PDE4DIP	8	-0.3098363	6.96E-08	0.00127082	0.506	0.768
RNH1	8	-0.3107197	2.01E-06	0.03660163	0.344	0.576
MZT2B	8	-0.3108869	9.91E-11	1.81E-06	0.909	0.985
RPS21	8	-0.3109553	4.10E-17	7.49E-13	1	1
PLP2	8	-0.3114313	2.72E-07	0.0049552	0.506	0.783
TMED3	8	-0.3115963	3.47E-07	0.00633046	0.532	0.763
GYPC	8	-0.3119206	2.13E-07	0.00387893	0.234	0.495
PSMB4	8	-0.3120735	9.21E-09	0.00016799	0.208	0.49
RPL28	8	-0.3132025	5.81E-23	1.06E-18	1	1
CHID1	8	-0.313407	2.94E-07	0.00535565	0.519	0.768
KDELR2	8	-0.3163551	1.94E-08	0.00035466	0.844	0.98
CREG1	8	-0.3166696	3.59E-07	0.00654555	0.429	0.667
MPP6	8	-0.3171903	3.52E-09	6.42E-05	0.201	0.5
GLTSCR2	8	-0.3180385	1.38E-08	0.00025127	0.792	0.965
RCN3	8	-0.3181651	8.18E-07	0.0149333	0.403	0.657
PRDX4	8	-0.3184537	3.77E-07	0.00688466	0.753	0.934
AC003090.1	8	-0.318739	8.16E-09	0.00014898	0.117	0.384
NACA	8	-0.3199095	2.95E-15	5.38E-11	1	1
RHOBTB3	8	-0.3202158	6.95E-07	0.01268	0.747	0.904
RPL35A	8	-0.321003	3.09E-22	5.64E-18	1	1
ALDOC	8	-0.3212048	1.31E-06	0.02386808	0.221	0.455
CCDC8	8	-0.3223312	7.55E-09	0.00013778	0.169	0.449
TAF1D	8	-0.3237871	4.99E-08	0.00091062	0.344	0.626
RPS19	8	-0.323841	4.26E-20	7.77E-16	1	1
ATRNL1	8	-0.3260968	5.07E-07	0.00924912	0.156	0.384
GLO1	8	-0.3265219	2.54E-10	4.63E-06	0.247	0.581
ERGIC3	8	-0.3269092	7.78E-09	0.00014191	0.792	0.929
RP11-71N10.1	8	-0.3289918	2.35E-11	4.29E-07	0.039	0.333
TCF4	8	-0.3292642	2.44E-08	0.00044475	0.409	0.697
RPL10	8	-0.3293027	2.38E-22	4.35E-18	1	1
MYL12B	8	-0.3294339	4.53E-07	0.00826078	0.539	0.758
ATP5G2	8	-0.3307673	1.39E-09	2.53E-05	1	0.995
C10orf54	8	-0.3318319	2.90E-10	5.29E-06	0.091	0.379
PGAM1	8	-0.3349696	1.54E-09	2.81E-05	0.773	0.934
PPIA	8	-0.3364722	2.88E-10	5.26E-06	0.942	0.985
PPT1	8	-0.3364722	2.01E-08	0.00036612	0.37	0.641

ATP5H	8	-0.3368899	7.91E-08	0.00144294	0.481	0.712
TOMM7	8	-0.3381715	4.35E-19	7.94E-15	1	1
LDHA	8	-0.3387465	7.27E-10	1.33E-05	0.968	1
CAV1	8	-0.3391323	1.26E-07	0.00229716	0.474	0.717
YBX1	8	-0.3410611	4.18E-13	7.62E-09	0.987	1
SLC44A1	8	-0.3411342	2.43E-06	0.04425488	0.312	0.535
RPS23	8	-0.3414105	4.41E-23	8.04E-19	1	1
CD63	8	-0.341513	3.24E-10	5.92E-06	1	1
EIF3K	8	-0.3416842	1.03E-09	1.88E-05	0.721	0.934
RHOA	8	-0.3416842	9.08E-08	0.00165652	0.695	0.894
EIF2A	8	-0.3456889	2.54E-09	4.63E-05	0.532	0.793
MDH2	8	-0.3460417	8.49E-09	0.000155	0.286	0.566
PRDX5	8	-0.3461075	3.13E-09	5.70E-05	0.825	0.944
AP2M1	8	-0.3463288	6.75E-09	0.00012317	0.416	0.702
PPA1	8	-0.3477354	7.62E-10	1.39E-05	0.26	0.576
KHDRBS3	8	-0.3478038	2.53E-12	4.62E-08	0.013	0.298
PKM	8	-0.3487791	3.94E-09	7.19E-05	0.935	0.97
HNRNPA1	8	-0.3498286	2.34E-12	4.27E-08	1	0.995
CYBRD1	8	-0.3537513	4.36E-09	7.95E-05	0.396	0.697
RPL8	8	-0.3551683	9.72E-28	1.77E-23	1	1
C12orf57	8	-0.3561653	8.01E-14	1.46E-09	0.922	0.995
EEF1D	8	-0.3563875	1.83E-16	3.34E-12	1	1
NFIA	8	-0.3571497	9.97E-09	0.00018201	0.364	0.646
RPL29	8	-0.3574982	2.64E-24	4.82E-20	1	1
H3F3A	8	-0.3577147	2.55E-15	4.65E-11	0.987	1
NKX3-2	8	-0.3605698	4.21E-08	0.00076773	0.214	0.49
ATP2B1	8	-0.3609807	9.05E-09	0.0001652	0.221	0.53
SLC25A6	8	-0.3609807	7.93E-12	1.45E-07	0.916	0.995
TSPAN4	8	-0.3613182	4.60E-07	0.00839014	0.487	0.697
LTA4H	8	-0.3619963	5.68E-10	1.04E-05	0.24	0.551
RPS27	8	-0.362322	9.83E-26	1.79E-21	1	1
PABPC1	8	-0.3643863	3.61E-10	6.60E-06	0.844	0.96
TMED10	8	-0.364648	5.94E-10	1.08E-05	0.89	0.99
LECT1	8	-0.3658545	6.26E-07	0.01143126	0.994	0.995
RPS29	8	-0.3677831	4.51E-26	8.23E-22	1	1
TSPO	8	-0.3678691	3.36E-13	6.14E-09	0.805	0.955
CCPG1	8	-0.3682987	1.67E-07	0.00304767	0.461	0.712
IDI1	8	-0.3686293	3.75E-07	0.00684726	0.403	0.631
MT-ND4	8	-0.3688302	2.42E-08	0.00044145	0.987	0.99

UBA52	8	-0.3692521	3.73E-25	6.80E-21	1	1
SERPINA5	8	-0.370881	4.66E-07	0.00850469	0.779	0.914
RPL9	8	-0.3724849	2.15E-25	3.92E-21	1	1
RPL31	8	-0.3734112	2.16E-22	3.93E-18	1	1
CNBP	8	-0.3737793	5.49E-09	0.00010025	0.643	0.823
UQCRH	8	-0.3741816	1.27E-13	2.32E-09	0.948	0.995
RPL19	8	-0.3746111	1.32E-33	2.42E-29	1	1
RPS2	8	-0.3752216	7.63E-26	1.39E-21	1	1
ARL6IP5	8	-0.3753121	5.99E-08	0.0010928	0.545	0.758
GPC1	8	-0.375343	6.39E-12	1.17E-07	0.11	0.439
NGFRAP1	8	-0.3755834	4.91E-12	8.96E-08	0.922	0.985
RPS7	8	-0.3757011	2.21E-22	4.03E-18	1	1
GAS6	8	-0.3757132	6.41E-09	0.000117	0.266	0.566
RPL35	8	-0.3770786	1.42E-24	2.60E-20	1	1
RPLP2	8	-0.3795162	5.37E-31	9.80E-27	1	1
ZBTB20	8	-0.3797603	1.11E-08	0.0002018	0.331	0.596
ATP5O	8	-0.3797744	4.90E-10	8.95E-06	0.656	0.869
RPL18A	8	-0.3798619	1.55E-28	2.82E-24	1	1
ATP5F1	8	-0.3801392	3.32E-08	0.00060541	0.383	0.626
HOXB6	8	-0.381513	6.15E-10	1.12E-05	0.253	0.566
MT-CO2	8	-0.3822914	5.15E-10	9.39E-06	0.968	0.99
PRDX2	8	-0.3857149	2.45E-09	4.47E-05	0.604	0.828
LRPAP1	8	-0.3859523	6.91E-08	0.00126158	0.494	0.707
EIF3M	8	-0.386617	1.10E-09	2.01E-05	0.37	0.677
RPL23A	8	-0.3871347	1.72E-26	3.14E-22	1	1
EIF3F	8	-0.3874715	3.23E-11	5.90E-07	0.662	0.904
RPL34	8	-0.3880917	1.40E-24	2.55E-20	1	1
COX7C	8	-0.3903705	3.98E-19	7.26E-15	1	1
RGS10	8	-0.3905395	2.90E-09	5.30E-05	0.318	0.586
ACTB	8	-0.3927261	5.94E-07	0.01083916	0.838	0.909
SQLE	8	-0.392917	2.59E-06	0.04732784	0.214	0.429
GNB2L1	8	-0.3930294	3.21E-17	5.85E-13	0.994	1
MEG3	8	-0.3945121	3.80E-07	0.0069328	0.338	0.576
RPL13A	8	-0.3949347	9.14E-32	1.67E-27	1	1
ARHGAP29	8	-0.3962066	1.90E-07	0.0034706	0.273	0.51
DDOST	8	-0.3969822	1.08E-08	0.00019732	0.331	0.606
RPS27A	8	-0.3971926	2.19E-29	4.00E-25	1	1
ITM2A	8	-0.3992731	7.36E-08	0.00134215	0.584	0.808
ARPC2	8	-0.3998496	3.00E-09	5.47E-05	0.649	0.889

ST13	8	-0.3999367	3.43E-13	6.27E-09	0.896	0.955
FXYD5	8	-0.4003822	2.45E-09	4.47E-05	0.753	0.914
C14orf166	8	-0.4027347	7.59E-12	1.38E-07	0.597	0.848
CXXC5	8	-0.4044063	4.52E-07	0.00824968	0.448	0.631
ARL2BP	8	-0.4046537	4.29E-10	7.82E-06	0.442	0.732
GSTP1	8	-0.4049726	2.36E-12	4.31E-08	0.916	0.99
NAP1L1	8	-0.4054035	1.05E-14	1.91E-10	0.909	0.98
TKT	8	-0.4064934	8.02E-10	1.46E-05	0.286	0.596
RPS5	8	-0.4079592	1.74E-27	3.17E-23	1	1
RPSA	8	-0.4099203	1.48E-20	2.70E-16	1	1
ATP5A1	8	-0.4103341	3.44E-10	6.28E-06	0.429	0.722
EPB41L4A-AS1	8	-0.4106614	2.83E-09	5.16E-05	0.669	0.879
LGALS1	8	-0.411466	9.30E-09	0.00016964	0.994	0.98
HERPUD1	8	-0.4169352	1.40E-06	0.02560144	0.513	0.742
ALDOA	8	-0.4177835	5.74E-15	1.05E-10	0.981	1
HSPA8	8	-0.4195601	2.87E-11	5.23E-07	0.299	0.657
TXNIP	8	-0.420523	2.59E-12	4.72E-08	0.065	0.389
UQCRB	8	-0.4218834	5.63E-15	1.03E-10	0.968	0.995
RPS18	8	-0.4219327	3.71E-35	6.77E-31	1	1
SSPN	8	-0.4231406	3.82E-10	6.97E-06	0.305	0.611
VDAC2	8	-0.4232946	5.17E-10	9.44E-06	0.403	0.677
EEF1B2	8	-0.4268699	2.23E-17	4.08E-13	0.981	1
HIST1H1C	8	-0.4307215	4.42E-08	0.00080629	0.13	0.384
PDIA3	8	-0.4330479	1.26E-08	0.00023018	0.61	0.803
C14orf1	8	-0.4344162	1.98E-13	3.61E-09	0.247	0.636
B2M	8	-0.4353181	3.30E-16	6.02E-12	1	1
EIF1	8	-0.4374047	5.28E-19	9.64E-15	1	1
HDLBP	8	-0.4436297	2.21E-11	4.03E-07	0.513	0.763
PRDX6	8	-0.4449529	8.30E-13	1.51E-08	0.584	0.859
NINJ1	8	-0.4475854	1.03E-10	1.88E-06	0.442	0.717
RPL10A	8	-0.4482526	1.43E-29	2.60E-25	1	1
HNRNPC	8	-0.4486355	2.87E-14	5.24E-10	0.532	0.854
FBXO2	8	-0.4502432	7.95E-15	1.45E-10	0.838	0.98
RPS13	8	-0.4503723	2.44E-29	4.46E-25	1	1
RPS3A	8	-0.4515435	1.59E-33	2.90E-29	1	1
SSR3	8	-0.4521068	3.30E-13	6.02E-09	0.89	0.975
TPT1	8	-0.4528062	6.10E-29	1.11E-24	1	1
CLEC3A	8	-0.4548707	6.33E-11	1.15E-06	0.734	0.909
NPM1	8	-0.4585543	7.60E-15	1.39E-10	1	1

ZFP36L2	8	-0.4607778	6.84E-11	1.25E-06	0.136	0.455
NPC2	8	-0.4609538	3.81E-14	6.95E-10	0.929	1
CALM1	8	-0.4614938	2.23E-10	4.07E-06	0.455	0.737
MT-CO3	8	-0.4659681	1.03E-09	1.87E-05	0.974	0.965
RPL37	8	-0.4669149	2.72E-35	4.96E-31	1	1
SLC25A3	8	-0.4676914	1.29E-15	2.35E-11	0.753	0.97
IGFBP7	8	-0.4693866	4.48E-09	8.17E-05	0.818	0.934
GNAS	8	-0.4696553	2.96E-13	5.40E-09	0.617	0.859
HLA-B	8	-0.4726747	6.67E-10	1.22E-05	0.734	0.864
CCNI	8	-0.4768585	3.22E-21	5.88E-17	0.987	1
HSP90AA1	8	-0.4771602	3.26E-11	5.95E-07	0.494	0.798
HOXC10	8	-0.4791704	5.13E-14	9.37E-10	0.513	0.833
RPS8	8	-0.4811669	7.73E-37	1.41E-32	1	1
YBX3	8	-0.4815731	5.12E-17	9.35E-13	0.662	0.97
MT-ND1	8	-0.4817655	1.37E-07	0.00250064	0.74	0.879
MT-ATP6	8	-0.4819841	2.36E-08	0.00043067	0.929	0.96
EIF3L	8	-0.4834188	4.44E-17	8.11E-13	0.773	0.97
BTF3	8	-0.4838875	9.14E-23	1.67E-18	0.968	1
EEF2	8	-0.4846274	1.06E-13	1.93E-09	0.981	0.995
EIF4B	8	-0.4858243	2.31E-13	4.21E-09	0.669	0.919
RPL4	8	-0.4860006	4.09E-28	7.47E-24	1	1
PEBP1	8	-0.4860532	2.76E-13	5.03E-09	0.649	0.894
CITED2	8	-0.4866309	8.11E-09	0.00014797	0.377	0.652
RPS16	8	-0.4935143	1.81E-36	3.30E-32	1	1
RPL14	8	-0.4942292	1.93E-33	3.51E-29	1	1
RPL23	8	-0.4963619	4.07E-28	7.42E-24	0.994	1
SEC11A	8	-0.4969735	1.86E-18	3.40E-14	0.812	0.965
HSP90AB1	8	-0.4970954	1.06E-12	1.94E-08	0.883	0.975
SLC14A1	8	-0.4981857	3.69E-13	6.72E-09	0.221	0.606
SLC40A1	8	-0.5051142	7.22E-08	0.00131718	0.234	0.475
RPS17	8	-0.5054679	1.18E-35	2.15E-31	1	1
RPL30	8	-0.5067083	4.05E-35	7.39E-31	1	1
MYLK	8	-0.513009	9.13E-10	1.67E-05	0.357	0.646
CTGF	8	-0.5156715	2.35E-07	0.00429549	0.448	0.717
MSMO1	8	-0.5179229	1.64E-09	2.99E-05	0.273	0.581
PDIA6	8	-0.5222188	2.97E-16	5.42E-12	0.76	0.96
PLXDC2	8	-0.526598	2.41E-15	4.40E-11	0.487	0.793
PTRF	8	-0.5309605	3.92E-13	7.16E-09	0.383	0.697
MT-CO1	8	-0.5315051	3.08E-12	5.62E-08	0.994	0.99

TUBB2B	8	-0.5326392	1.64E-10	2.99E-06	0.39	0.697
HINT1	8	-0.5343356	1.52E-23	2.78E-19	0.948	0.995
TUBB	8	-0.5352243	3.07E-14	5.60E-10	0.558	0.859
RPL18	8	-0.5360652	6.48E-37	1.18E-32	1	1
HLA-C	8	-0.5431284	2.54E-15	4.63E-11	0.792	0.975
LDHB	8	-0.5500083	1.07E-19	1.95E-15	0.617	0.919
P4HB	8	-0.5500834	2.14E-17	3.91E-13	0.89	0.995
PSMA7	8	-0.5507268	1.98E-16	3.61E-12	0.558	0.894
PAX1	8	-0.5511998	6.66E-15	1.22E-10	0.669	0.909
IGFBP6	8	-0.5562173	8.68E-09	0.00015839	0.701	0.859
RPL7	8	-0.5586737	5.70E-35	1.04E-30	1	1
APOD	8	-0.5596158	1.60E-09	2.91E-05	0.201	0.475
VIM	8	-0.5603206	5.53E-12	1.01E-07	0.994	1
SSR2	8	-0.5605986	7.45E-21	1.36E-16	0.792	0.985
COL2A1	8	-0.5668463	1.87E-13	3.41E-09	1	1
FHL1	8	-0.5753641	3.42E-15	6.25E-11	0.286	0.662
ISLR	8	-0.5794972	2.80E-14	5.11E-10	0.591	0.848
NUCB2	8	-0.5912102	9.22E-13	1.68E-08	0.494	0.783
THY1	8	-0.5922866	1.58E-14	2.88E-10	0.409	0.742
EEF1A1	8	-0.5997358	3.25E-37	5.94E-33	1	1
CHRDL2	8	-0.6067074	7.63E-11	1.39E-06	0.214	0.525
INSIG1	8	-0.6076498	2.19E-10	3.99E-06	0.383	0.692
HLA-A	8	-0.6114959	3.82E-15	6.97E-11	0.786	0.965
CCDC102B	8	-0.620081	9.47E-21	1.73E-16	0.058	0.525
SDC2	8	-0.6202638	5.47E-23	9.98E-19	0.87	0.985
CRIP2	8	-0.6303216	2.29E-17	4.19E-13	0.344	0.727
MSMP	8	-0.6398102	6.15E-07	0.011229	0.058	0.263
CRTAP	8	-0.6441903	2.47E-24	4.51E-20	0.61	0.949
SPINT2	8	-0.6575413	7.26E-22	1.32E-17	0.156	0.662
CD9	8	-0.6627096	2.62E-13	4.77E-09	0.448	0.717
TPM1	8	-0.6657033	2.86E-12	5.23E-08	0.455	0.742
C2orf82	8	-0.6733503	1.82E-18	3.33E-14	0.955	1
MT-ND3	8	-0.6832605	1.33E-18	2.43E-14	0.896	0.949
RPLP0	8	-0.7026403	3.77E-49	6.87E-45	1	1
ZFAS1	8	-0.7028711	8.40E-26	1.53E-21	0.987	1
PLPP1	8	-0.7034236	3.33E-14	6.07E-10	0.227	0.591
MT-CYB	8	-0.7343252	5.95E-17	1.09E-12	0.805	0.949
ACTG1	8	-0.7361442	3.47E-22	6.33E-18	0.851	0.98
SPARC	8	-0.7531308	7.15E-22	1.31E-17	0.909	1

MT-ND2	8	-0.7679073	2.06E-13	3.75E-09	0.779	0.889
EIF3H	8	-0.875105	8.13E-38	1.48E-33	0.883	1
OGN	8	-0.8852488	1.31E-20	2.39E-16	0.961	0.995
ADIRF	8	-0.9262863	4.99E-21	9.10E-17	0.409	0.823
RPS20	8	-0.9404279	8.33E-53	1.52E-48	0.994	1
RPS11	8	-0.9616252	1.38E-54	2.52E-50	0.987	1
FTH1	8	-1.0136604	1.13E-49	2.07E-45	1	1
CAPS	8	-1.0459003	1.40E-32	2.55E-28	0.74	0.99
CRISPLD1	8	-1.267277	1.32E-27	2.42E-23	0.318	0.798
FRZB	8	-1.5388665	1.78E-23	3.25E-19	0.422	0.833
SPTSSB	8	-1.7710606	5.27E-38	9.62E-34	0.019	0.727
**gene**	**cluster**	**avg_logFC**	**p_val**	**p_val_adj**	**pct.1**	**pct.2**
EPYC	9	1.75101624	3.49E-18	6.36E-14	0.901	0.386
TGFBI	9	1.65259324	1.68E-21	3.07E-17	0.96	0.53
CTHRC1	9	1.55141957	7.65E-21	1.40E-16	0.861	0.289
PLA2G2A	9	1.52054076	1.83E-10	3.33E-06	0.752	0.337
CHI3L2	9	1.43311262	3.91E-14	7.14E-10	0.901	0.542
CYP1B1	9	1.17436468	7.31E-12	1.33E-07	0.673	0.205
PRG4	9	1.14109865	1.22E-11	2.23E-07	0.743	0.265
CFD	9	1.06166442	2.69E-11	4.91E-07	0.574	0.096
TNFRSF11B	9	1.01891996	1.37E-08	0.00025084	0.782	0.458
SPP1	9	0.99619852	5.29E-10	9.65E-06	0.554	0.108
MT1X	9	0.93070173	5.18E-08	0.0009443	1	0.976
PTGS2	9	0.71339558	1.20E-07	0.00219285	0.436	0.096
VEGFA	9	0.70817637	1.91E-06	0.03488384	0.683	0.386
COMP	9	0.70555709	6.32E-08	0.00115259	0.98	0.88
CTSD	9	0.70037757	1.51E-07	0.00276043	1	0.976
BTG1	9	0.63458452	3.65E-08	0.00066653	0.98	0.831
PMP22	9	0.58253983	5.45E-08	0.00099446	0.901	0.711
PPP3CA	9	0.56058309	5.29E-07	0.00964799	0.871	0.627
TNFAIP6	9	0.51321641	4.99E-07	0.00910542	0.406	0.084
EVI2A	9	0.45531827	1.73E-08	0.00031573	0.386	0.036
LTBP1	9	0.44660544	1.47E-07	0.00268698	0.554	0.169
DDIT4L	9	0.40671617	2.16E-06	0.0393943	0.564	0.205
PLA2G4A	9	0.39723743	1.03E-06	0.01870794	0.634	0.277
RPL19	9	-0.2625759	1.99E-07	0.00363488	1	1
RPL13	9	-0.2710991	2.93E-07	0.00534952	1	1
RPL7A	9	-0.2715573	1.53E-07	0.00279328	1	1
RPL15	9	-0.2729993	5.49E-07	0.01001629	1	1

UBA52	9	-0.2829863	4.29E-08	0.000783	1	1
MAEA	9	-0.2840555	2.15E-06	0.03915723	0.069	0.349
RPL6	9	-0.2852893	2.69E-06	0.04907257	1	1
COX7C	9	-0.2914437	1.74E-06	0.03181502	1	1
RPL8	9	-0.2943702	3.15E-09	5.75E-05	1	1
RPS18	9	-0.2988002	7.45E-07	0.01359545	1	1
EIF1	9	-0.2993651	4.32E-07	0.00788929	1	1
CLEC3B	9	-0.2996583	1.98E-07	0.00361132	0	0.241
GFRA1	9	-0.2996583	4.34E-07	0.00791839	0	0.229
RPLP1	9	-0.3005753	9.71E-07	0.01772613	1	1
MXD4	9	-0.3035254	1.56E-07	0.00284724	0.05	0.349
HOXC8	9	-0.3060948	1.39E-06	0.02535878	0.109	0.41
RPS29	9	-0.3106572	3.24E-07	0.00591569	1	1
RPL35A	9	-0.3118305	9.79E-08	0.00178653	1	1
RPS13	9	-0.3195726	2.49E-06	0.04540455	1	1
GNB2L1	9	-0.3227937	8.84E-09	0.00016125	1	1
H3F3A	9	-0.3246611	6.84E-07	0.01248897	0.98	1
RPL13A	9	-0.3255522	1.12E-07	0.00204761	1	1
RPL18A	9	-0.3259795	5.89E-07	0.01074698	1	1
OAZ1	9	-0.3294932	4.57E-07	0.00833934	1	1
RPL4	9	-0.3333544	2.00E-06	0.03645255	0.99	1
RAC1	9	-0.3452222	1.54E-07	0.00280749	0.901	0.964
RPS27A	9	-0.3466923	1.47E-07	0.0026772	1	1
KLHDC2	9	-0.3482961	5.06E-07	0.00923485	0.257	0.614
RPL10A	9	-0.3537772	7.47E-09	0.00013635	1	1
RPSA	9	-0.3637129	1.68E-06	0.03071885	0.99	1
EIF3K	9	-0.3672377	5.67E-07	0.01035264	0.723	0.892
RPL29	9	-0.3737938	1.88E-11	3.44E-07	1	1
RPL10	9	-0.3773078	6.23E-11	1.14E-06	1	1
RPL9	9	-0.3787269	6.80E-09	0.00012413	1	1
HNRNPC	9	-0.3853812	3.54E-07	0.00646081	0.703	0.904
EIF3L	9	-0.3894124	1.10E-07	0.00201127	0.683	0.928
BTF3	9	-0.3898318	3.18E-08	0.0005799	0.97	0.988
RPL23	9	-0.3959199	4.67E-10	8.52E-06	1	1
RPL14	9	-0.3959538	8.11E-11	1.48E-06	1	1
MYL6	9	-0.3986484	1.33E-06	0.02420038	0.99	0.988
RPLP2	9	-0.4034858	1.67E-11	3.04E-07	1	1
RPL31	9	-0.4037185	1.94E-07	0.00353342	1	1
RPL34	9	-0.4072099	9.59E-08	0.00175005	1	1

CCDC8	9	-0.4141924	2.35E-08	0.00042831	0.129	0.494
RPS16	9	-0.4156781	4.74E-11	8.66E-07	1	1
PDLIM2	9	-0.4214832	8.28E-07	0.01510057	0.584	0.807
RPL23A	9	-0.4230447	1.30E-10	2.38E-06	1	1
RPL30	9	-0.4272478	7.10E-11	1.30E-06	1	1
RPS3A	9	-0.4274071	2.47E-10	4.51E-06	1	1
TMEM106C	9	-0.4302177	1.82E-07	0.00332166	0.208	0.578
CCNI	9	-0.433006	1.34E-07	0.0024435	0.97	1
RPL37	9	-0.4368101	3.12E-11	5.70E-07	1	1
C14orf1	9	-0.4442967	4.07E-07	0.00743095	0.267	0.602
YBX1	9	-0.4487252	3.21E-08	0.00058614	0.96	0.976
ITGA11	9	-0.454109	2.20E-08	0.00040213	0.158	0.53
ARPC2	9	-0.4544203	5.36E-07	0.00977806	0.822	0.94
RPS17	9	-0.4571854	4.39E-12	8.00E-08	1	1
RPS8	9	-0.458318	2.20E-10	4.02E-06	1	1
NPDC1	9	-0.4617164	8.65E-10	1.58E-05	0.208	0.639
CCDC102B	9	-0.4634835	4.85E-07	0.0088527	0.089	0.386
ITGBL1	9	-0.4762399	7.86E-08	0.00143361	0.228	0.602
HRCT1	9	-0.4769933	3.42E-08	0.0006249	0.05	0.373
FTH1	9	-0.4788923	2.02E-10	3.68E-06	1	1
FADS1	9	-0.4817857	1.83E-07	0.00333516	0.109	0.434
MEG3	9	-0.498282	3.60E-07	0.00657667	0.347	0.675
RPL18	9	-0.4989687	1.28E-14	2.33E-10	1	1
KDELR2	9	-0.5009403	2.31E-09	4.22E-05	0.941	0.988
GPC1	9	-0.5042467	1.35E-08	0.00024549	0.198	0.566
NUCB2	9	-0.5076162	3.08E-07	0.00561983	0.762	0.892
ARPC1B	9	-0.5244268	4.81E-07	0.00878273	0.584	0.831
RPL7	9	-0.5293222	7.11E-11	1.30E-06	1	1
MAP1B	9	-0.5441159	6.39E-07	0.01165656	0.208	0.53
ISLR	9	-0.5589119	1.05E-06	0.01915214	0.832	0.867
HDLBP	9	-0.5622142	3.14E-09	5.73E-05	0.495	0.795
IGFBP6	9	-0.5827871	1.31E-07	0.00238342	0.842	0.916
HINT1	9	-0.5831886	2.34E-14	4.26E-10	0.921	1
COL14A1	9	-0.5954358	1.72E-11	3.14E-07	0.04	0.458
EEF2	9	-0.5988423	2.66E-11	4.85E-07	0.98	1
SCD	9	-0.601745	1.49E-07	0.00271434	0.158	0.494
TPT1	9	-0.6025637	5.89E-14	1.07E-09	1	1
DAB2	9	-0.607937	6.56E-10	1.20E-05	0.416	0.771
SPTSSB	9	-0.6115973	1.87E-08	0.00034132	0	0.277

LOXL1	9	-0.6266426	5.42E-08	0.00098877	0.149	0.506
PTRF	9	-0.6350022	1.08E-10	1.98E-06	0.604	0.88
RPLP0	9	-0.6379358	6.95E-19	1.27E-14	1	1
SDC2	9	-0.6476324	4.93E-14	9.00E-10	0.99	0.976
PAX1	9	-0.653647	1.94E-08	0.0003537	0.624	0.855
INSIG1	9	-0.6943665	6.49E-07	0.01184408	0.465	0.771
MT-ND4	9	-0.7161304	1.07E-13	1.96E-09	0.99	0.988
PLPP1	9	-0.7219302	2.20E-09	4.02E-05	0.634	0.892
MT-CO2	9	-0.7283685	2.55E-11	4.65E-07	0.99	0.988
CLEC3A	9	-0.7314228	2.18E-07	0.00397796	0.307	0.651
CRIP2	9	-0.7358845	6.71E-12	1.22E-07	0.257	0.687
VIM	9	-0.7792955	9.94E-07	0.01813202	1	1
EEF1A1	9	-0.7858838	5.93E-21	1.08E-16	1	1
RPS11	9	-0.7888295	4.57E-21	8.34E-17	1	1
THY1	9	-0.794025	5.44E-12	9.92E-08	0.871	0.988
NEAT1	9	-0.8073935	5.32E-08	0.00097068	0.99	0.976
ZFP36L2	9	-0.877731	5.16E-08	0.00094124	0.208	0.542
EIF3H	9	-0.8877646	2.04E-19	3.73E-15	0.851	0.976
MT-ND1	9	-0.9001992	2.70E-16	4.93E-12	0.713	0.952
COL8A1	9	-0.9327239	4.06E-09	7.41E-05	0.089	0.458
RPS20	9	-0.9509704	2.49E-27	4.55E-23	0.99	1
NOV	9	-0.9527967	1.32E-07	0.00240237	0.05	0.349
FHL1	9	-0.9675919	1.61E-12	2.93E-08	0.574	0.904
MT-CO1	9	-0.967745	3.07E-19	5.60E-15	1	0.976
MT-CO3	9	-0.9729971	7.49E-20	1.37E-15	0.97	0.976
TPM1	9	-1.0155965	3.09E-11	5.65E-07	0.752	0.964
MT-ATP6	9	-1.0252439	3.91E-20	7.13E-16	0.901	1
CAPS	9	-1.0911204	4.29E-15	7.82E-11	0.614	0.928
MT-ND3	9	-1.0997464	8.55E-19	1.56E-14	0.822	0.964
MT-ND2	9	-1.1146737	1.68E-18	3.06E-14	0.772	0.976
MT-CYB	9	-1.1305891	4.20E-19	7.66E-15	0.822	0.976
ADIRF	9	-1.186295	4.84E-15	8.83E-11	0.277	0.771
CTGF	9	-1.1898402	5.34E-09	9.74E-05	0.851	0.988
MSMP	9	-2.1988854	1.08E-07	0.00197842	0.109	0.446
**gene**	**cluster**	**avg_logFC**	**p_val**	**p_val_adj**	**pct.1**	**pct.2**
SPP1	10	2.40740793	6.69E-14	1.22E-09	0.745	0.098
MT2A	10	1.57550069	1.71E-12	3.12E-08	1	0.98
VEGFA	10	1.52106208	3.01E-16	5.49E-12	0.908	0.373
PPP3CA	10	1.51189604	4.78E-22	8.72E-18	1	0.941

COMP	10	1.50219179	1.54E-14	2.82E-10	0.969	0.627
S100A2	10	1.49073675	1.27E-08	0.00023151	0.745	0.373
CHI3L2	10	1.46295719	9.37E-15	1.71E-10	0.888	0.333
PTGS2	10	1.42861184	7.30E-14	1.33E-09	0.714	0.039
CCDC85B	10	1.42120544	5.78E-18	1.06E-13	0.99	0.725
MT1X	10	1.39064749	4.44E-11	8.11E-07	1	0.961
MT1G	10	1.34101842	2.31E-09	4.22E-05	0.969	0.863
PLA2G2A	10	1.28286723	6.32E-08	0.00115348	0.724	0.275
DDIT4L	10	1.25561233	7.90E-14	1.44E-09	0.908	0.353
MRPS6	10	1.25357321	6.66E-10	1.22E-05	0.969	0.882
CTSD	10	1.24088695	1.70E-08	0.00030993	0.959	0.961
PHLDA1	10	1.15999248	5.59E-10	1.02E-05	0.582	0.059
STK17A	10	1.135637	4.60E-11	8.40E-07	0.653	0.098
FN1	10	1.13057094	1.03E-15	1.88E-11	1	1
PRG4	10	1.12948514	2.79E-09	5.08E-05	0.571	0.078
IGFBP3	10	1.10996237	1.59E-08	0.0002896	0.582	0.118
BMP2	10	1.10666602	1.29E-10	2.35E-06	0.582	0.02
UPP1	10	1.09755614	1.31E-12	2.38E-08	0.735	0.137
ERO1A	10	1.07437535	5.08E-14	9.28E-10	0.857	0.333
FOSL1	10	1.0689377	6.55E-09	0.00011943	0.5	0.02
PTP4A1	10	1.0501622	1.53E-13	2.79E-09	0.806	0.196
WTAP	10	1.04466522	7.62E-12	1.39E-07	0.969	0.706
SERPINE2	10	1.03354197	8.20E-13	1.50E-08	1	0.98
IGFBP5	10	1.01284824	1.24E-08	0.00022712	0.551	0.078
TNFRSF12A	10	1.00589301	8.91E-11	1.63E-06	0.663	0.098
ARF6	10	0.97740503	2.49E-12	4.55E-08	0.837	0.294
PPP1CB	10	0.96943254	6.20E-17	1.13E-12	1	0.922
ADM	10	0.92685939	4.34E-08	0.00079175	0.888	0.588
TGFBI	10	0.92683403	2.25E-10	4.11E-06	0.694	0.196
CTNNB1	10	0.88867588	1.84E-12	3.36E-08	0.98	0.804
ARRDC3	10	0.84387836	2.59E-06	0.04726583	0.684	0.333
ISG20	10	0.83493521	5.59E-09	0.00010207	0.653	0.176
ENO2	10	0.82194801	1.01E-10	1.84E-06	0.786	0.294
PPFIBP1	10	0.82075057	2.12E-09	3.87E-05	0.724	0.255
BHLHE40	10	0.82075057	4.82E-07	0.00878653	0.663	0.333
HILPDA	10	0.77089284	4.23E-07	0.00772208	0.724	0.314
DDIT3	10	0.76996649	1.95E-07	0.00355382	0.735	0.373
NDRG1	10	0.7512799	2.64E-08	0.00048179	0.765	0.412
AK2	10	0.74408159	1.82E-08	0.00033135	0.806	0.529

SLC2A1	10	0.73731053	3.11E-07	0.00568259	0.571	0.176
RGS2	10	0.71209911	7.95E-07	0.01449791	0.653	0.235
SLC3A2	10	0.70227528	2.01E-07	0.00366212	0.663	0.235
WDR45B	10	0.70196959	7.58E-10	1.38E-05	0.633	0.118
SLC25A37	10	0.70119016	1.30E-10	2.38E-06	0.98	0.804
VAPA	10	0.68185922	1.40E-10	2.55E-06	0.969	0.863
SPRY2	10	0.68088409	2.51E-08	0.0004582	0.52	0.059
BAALC	10	0.66963911	6.63E-08	0.00120958	0.653	0.196
C11orf96	10	0.66861399	2.57E-06	0.04694879	0.388	0.02
SOD2	10	0.66101652	3.12E-08	0.00056959	0.99	0.765
PGK1	10	0.66041723	8.38E-09	0.00015288	0.98	0.922
ARPP19	10	0.65994911	3.64E-10	6.64E-06	0.857	0.412
HLA-DRB1	10	0.65780308	1.83E-06	0.03337777	0.571	0.216
CA9	10	0.63630269	1.90E-06	0.03473905	0.592	0.235
GAPDH	10	0.62747671	5.23E-07	0.00954815	1	1
FAM162A	10	0.62078146	1.63E-08	0.00029793	1	0.824
SDC4	10	0.61931737	3.36E-07	0.0061234	0.755	0.333
TREM1	10	0.61903921	1.79E-09	3.27E-05	0.5	0
COX17	10	0.61352955	9.87E-08	0.00180116	0.837	0.588
ZNF385D	10	0.60465047	1.56E-06	0.02845995	0.724	0.333
RABGGTB	10	0.59817708	1.36E-07	0.00247409	0.847	0.51
CTHRC1	10	0.59468284	9.29E-07	0.01695341	0.5	0.098
CD44	10	0.59019203	1.38E-07	0.00251764	0.918	0.647
SGIP1	10	0.58857129	9.16E-07	0.01671925	0.398	0.02
RNMT	10	0.55395109	8.93E-07	0.01629383	0.602	0.196
BTG1	10	0.54402212	1.19E-07	0.00216993	1	0.922
MRPL14	10	0.54376379	4.96E-07	0.00904305	0.694	0.294
PTGES3	10	0.54027824	5.81E-09	0.00010598	0.98	0.941
FAM210A	10	0.53784376	6.39E-07	0.01166355	0.602	0.196
HMGA2	10	0.53302633	1.46E-07	0.0026612	0.408	0
ATP6V1G1	10	0.52339376	1.62E-07	0.00295133	1	0.961
NRP2	10	0.52000349	1.49E-06	0.02718457	0.469	0.078
PLA2G4A	10	0.49199046	2.03E-06	0.03702537	0.48	0.098
EVI2A	10	0.48855403	1.93E-07	0.00351679	0.459	0.039
EPS8	10	0.48425647	1.37E-06	0.02501043	0.551	0.157
RTN4	10	0.47445798	2.23E-08	0.00040782	0.918	0.686
TRMT112	10	0.47155016	9.40E-07	0.01715092	0.949	0.941
CLDND1	10	0.46577571	5.93E-07	0.01081586	0.653	0.235
NBPF14	10	0.44547044	1.77E-06	0.03222016	0.439	0.059

A4GALT	10	0.41897883	2.26E-07	0.00411954	0.398	0
EIF4A2	10	0.41156889	8.71E-07	0.01590106	1	1
MIF	10	0.38619383	8.37E-07	0.01527611	1	1
RPL5	10	-0.2599368	1.18E-06	0.02159454	1	1
RPS19	10	-0.2619095	3.53E-08	0.00064383	1	1
RPS15A	10	-0.2638761	3.52E-08	0.00064147	1	1
RPS14	10	-0.2683722	7.44E-09	0.00013575	1	1
RPL26	10	-0.2815783	1.42E-08	0.00025943	1	1
RPL32	10	-0.2834958	3.73E-11	6.81E-07	1	1
NACA	10	-0.2896637	8.37E-07	0.01528149	1	1
ODF2L	10	-0.3007012	8.23E-07	0.01501728	0.031	0.314
RPS28	10	-0.3021819	8.27E-13	1.51E-08	1	1
GNB2L1	10	-0.3025517	1.68E-07	0.00306715	1	1
COX7C	10	-0.3030344	1.32E-06	0.024039	1	1
RPL6	10	-0.3041889	1.17E-07	0.00214048	1	1
AFF3	10	-0.3146874	2.54E-07	0.00464052	0.031	0.333
RPL24	10	-0.3160712	4.90E-10	8.95E-06	1	1
RPL15	10	-0.3186254	1.83E-13	3.35E-09	1	1
TXNIP	10	-0.3262391	1.37E-07	0.00249687	0.041	0.373
RPS21	10	-0.3264576	8.24E-09	0.00015033	1	1
RPS3	10	-0.3267902	1.83E-11	3.34E-07	1	1
RPL7A	10	-0.3268838	7.24E-10	1.32E-05	1	1
NBEAL1	10	-0.3316855	1.65E-06	0.0300744	0.99	1
SNRPA	10	-0.3370218	4.13E-07	0.0075404	0.082	0.431
RPL11	10	-0.3386961	3.87E-12	7.06E-08	1	1
RPL27A	10	-0.3394624	3.78E-11	6.90E-07	1	1
RPL28	10	-0.3408602	3.33E-12	6.08E-08	1	1
CLDN11	10	-0.3420864	2.38E-06	0.04339096	0.031	0.294
RPS25	10	-0.3517911	3.31E-13	6.03E-09	1	1
ATP5G2	10	-0.3528427	1.05E-06	0.01908062	1	1
GSTA4	10	-0.353899	9.68E-07	0.01766053	0.153	0.529
RPS24	10	-0.3544813	1.01E-08	0.00018391	1	1
RPS9	10	-0.3634987	1.02E-11	1.87E-07	1	1
KHDRBS3	10	-0.3654598	4.83E-07	0.00880902	0.01	0.275
RPS6	10	-0.3654598	2.90E-14	5.29E-10	1	1
RPS2	10	-0.368656	3.51E-11	6.40E-07	1	1
SERTAD4-AS1	10	-0.3687547	7.36E-08	0.00134388	0.031	0.353
RPL35	10	-0.3751437	2.09E-12	3.81E-08	1	1
RPL39	10	-0.37745	8.70E-14	1.59E-09	1	1

RPL12	10	-0.3776	3.27E-13	5.96E-09	1	1
COMMD6	10	-0.379446	1.25E-09	2.28E-05	0.99	1
TOMM7	10	-0.3797404	9.29E-10	1.70E-05	1	1
YBX1	10	-0.3815592	1.62E-07	0.00295607	0.98	1
RPL29	10	-0.3845431	2.21E-12	4.02E-08	1	1
CCDC8	10	-0.3907776	9.49E-07	0.01732509	0.061	0.373
PLP2	10	-0.3944916	2.42E-06	0.0440908	0.582	0.941
RPS27A	10	-0.3982973	5.57E-14	1.02E-09	1	1
RPL22	10	-0.4010718	2.62E-11	4.78E-07	1	1
RPS12	10	-0.4011555	3.27E-13	5.96E-09	1	1
RPL37A	10	-0.4016999	1.33E-13	2.43E-09	1	1
ATP5L	10	-0.4029173	1.81E-06	0.03303139	0.959	1
RPS5	10	-0.4042322	5.67E-13	1.03E-08	1	1
FUS	10	-0.4076414	2.40E-06	0.04372015	0.388	0.745
GLTSCR2	10	-0.4103836	9.37E-09	0.00017106	0.959	0.98
CXXC5	10	-0.4104112	2.29E-07	0.0041711	0.153	0.549
EID1	10	-0.4142067	1.61E-07	0.002939	0.918	1
RPL3	10	-0.4163831	2.33E-13	4.26E-09	1	1
RPL27	10	-0.4205452	3.65E-11	6.66E-07	1	1
RPL7	10	-0.4221309	4.12E-10	7.52E-06	1	1
RPS13	10	-0.4227373	1.86E-13	3.40E-09	1	1
RPS27	10	-0.4240059	1.40E-14	2.55E-10	1	1
CD99	10	-0.4282607	1.66E-06	0.03035859	0.929	1
RPL18A	10	-0.4290153	2.42E-15	4.42E-11	1	1
RPL13	10	-0.4298348	3.50E-17	6.39E-13	1	1
LRRC2	10	-0.4299983	2.12E-07	0.0038605	0.122	0.49
SDF4	10	-0.4299983	2.33E-06	0.04254408	0.653	0.843
TUBB	10	-0.4319049	1.43E-06	0.02615487	0.684	0.961
SSR4	10	-0.4393296	1.88E-06	0.0343382	0.898	0.98
SLC44A1	10	-0.4442407	3.04E-07	0.00554423	0.214	0.608
RPS7	10	-0.4462395	9.71E-13	1.77E-08	1	1
RPL19	10	-0.4473392	3.02E-18	5.51E-14	1	1
HINT1	10	-0.4479597	5.15E-07	0.0093923	0.959	0.98
CP	10	-0.4542277	1.84E-06	0.03352613	0.602	0.902
RPLP1	10	-0.4609078	4.92E-16	8.98E-12	1	1
RPA3	10	-0.462452	1.58E-07	0.00288106	0.378	0.765
RPL21	10	-0.4627842	4.49E-18	8.20E-14	1	1
RPL8	10	-0.4659175	1.64E-16	2.99E-12	1	1
NGFRAP1	10	-0.4679994	1.87E-07	0.00340319	0.796	0.961

RPS18	10	-0.4725307	7.54E-18	1.38E-13	1	1
PRKCSH	10	-0.4772512	1.31E-07	0.00239535	0.204	0.608
UBA52	10	-0.478048	9.81E-16	1.79E-11	1	1
RPL35A	10	-0.4789987	4.12E-16	7.52E-12	1	1
RPS23	10	-0.4792653	2.76E-15	5.03E-11	1	1
RPL31	10	-0.4849972	7.47E-14	1.36E-09	1	1
RPL34	10	-0.4869929	1.98E-16	3.61E-12	1	1
HIST1H1C	10	-0.4882672	1.95E-06	0.0355554	0.173	0.529
EEF1B2	10	-0.4920098	7.21E-08	0.00131608	0.969	1
PPIA	10	-0.4955129	1.02E-07	0.0018687	0.827	0.941
RPL37	10	-0.4980077	2.29E-14	4.18E-10	1	1
PRDX6	10	-0.4989912	4.66E-08	0.00085016	0.765	0.941
RPL10	10	-0.5007233	9.98E-19	1.82E-14	1	1
PPIC	10	-0.5014573	3.08E-07	0.00561942	0.296	0.667
NAP1L1	10	-0.5024816	1.29E-09	2.36E-05	0.98	1
RPL9	10	-0.5065865	1.76E-18	3.20E-14	1	1
RPS3A	10	-0.5079692	5.13E-16	9.35E-12	1	1
PCBP2	10	-0.5080496	4.39E-08	0.00080128	0.908	1
RPL14	10	-0.5097124	1.04E-16	1.89E-12	1	1
PLPP1	10	-0.5143054	8.62E-08	0.00157214	0.102	0.471
RPL13A	10	-0.5153472	5.93E-17	1.08E-12	1	1
RPSA	10	-0.5156343	2.15E-13	3.92E-09	1	1
HNRNPA1	10	-0.5157118	1.35E-08	0.00024646	1	1
RPL30	10	-0.5173315	6.58E-16	1.20E-11	1	1
MYL6	10	-0.5181856	1.17E-07	0.00212724	0.867	0.98
EIF3K	10	-0.5196105	4.17E-10	7.61E-06	0.704	0.961
FMOD	10	-0.520213	1.25E-07	0.00228101	0.827	0.98
OXA1L	10	-0.5222363	9.83E-07	0.01794162	0.449	0.784
COL6A2	10	-0.5276041	1.37E-07	0.00249756	0.52	0.863
HDLBP	10	-0.5362205	1.11E-06	0.02024505	0.367	0.706
HIST1H4C	10	-0.5395447	2.73E-07	0.00497275	0.337	0.745
EIF2A	10	-0.540155	5.37E-08	0.0009804	0.673	0.882
PTRF	10	-0.5477813	1.33E-06	0.02424996	0.347	0.686
PABPC1	10	-0.5493022	1.22E-07	0.00223499	0.878	1
EIF3D	10	-0.5510124	1.29E-08	0.00023577	0.663	0.902
RPL23	10	-0.5526439	3.23E-15	5.90E-11	1	1
RABAC1	10	-0.5587322	2.64E-08	0.00048129	0.643	0.922
RPS29	10	-0.5609714	7.89E-18	1.44E-13	1	1
MT-ND4	10	-0.5664551	2.34E-07	0.00426494	0.969	0.98

NDUFA11	10	-0.5799384	2.57E-09	4.68E-05	0.765	0.961
RHOBTB3	10	-0.5869599	7.00E-07	0.01277889	0.51	0.824
GPX3	10	-0.5904909	1.10E-08	0.00020053	0.918	0.98
NNMT	10	-0.5906215	5.18E-08	0.00094577	0.857	0.961
CITED2	10	-0.5920542	2.68E-09	4.89E-05	0.296	0.745
FHL1	10	-0.5959834	1.04E-09	1.91E-05	0.255	0.725
VIM	10	-0.5990073	2.38E-10	4.35E-06	1	1
CD9	10	-0.6002994	1.53E-06	0.02788601	0.286	0.627
NFIA	10	-0.6035449	4.68E-08	0.00085443	0.214	0.608
GAS6	10	-0.6126505	1.48E-08	0.00026996	0.204	0.627
ATRNL1	10	-0.6154015	8.39E-11	1.53E-06	0.092	0.569
TUBB2B	10	-0.6156843	2.63E-06	0.04790583	0.306	0.608
RPLP2	10	-0.620919	1.90E-19	3.47E-15	1	1
RPS17	10	-0.6232695	2.11E-16	3.85E-12	1	1
EIF3L	10	-0.6308124	7.48E-12	1.36E-07	0.816	0.961
RPL23A	10	-0.6321385	2.87E-20	5.24E-16	1	1
ISLR	10	-0.6322352	2.70E-08	0.00049319	0.327	0.725
SSPN	10	-0.6363347	8.31E-08	0.00151712	0.204	0.588
NPC2	10	-0.6409217	2.97E-13	5.42E-09	0.969	1
UQCRB	10	-0.6421164	7.77E-12	1.42E-07	0.98	1
SSR3	10	-0.6428147	2.04E-08	0.00037165	0.776	0.961
CCDC102B	10	-0.6433857	2.11E-13	3.85E-09	0.041	0.569
FTH1	10	-0.6494654	7.50E-11	1.37E-06	1	1
SEC11A	10	-0.6531418	1.45E-09	2.64E-05	0.765	0.961
TPT1	10	-0.6581005	1.34E-17	2.44E-13	1	1
PRUNE2	10	-0.6612391	2.42E-10	4.41E-06	0.224	0.706
P4HB	10	-0.668646	3.13E-11	5.71E-07	0.714	1
CCNI	10	-0.6689733	1.77E-16	3.22E-12	0.98	1
RPS16	10	-0.6723048	2.29E-18	4.18E-14	1	1
H3F3A	10	-0.6945986	6.33E-15	1.16E-10	0.918	1
EIF4B	10	-0.6974421	8.63E-12	1.57E-07	0.786	0.98
RPL4	10	-0.7115139	4.09E-17	7.46E-13	0.99	1
EEF1A1	10	-0.7176432	5.33E-19	9.73E-15	1	1
RPL18	10	-0.7208646	7.01E-20	1.28E-15	1	1
XAGE2	10	-0.7221347	5.41E-09	9.88E-05	0	0.314
SERPINA5	10	-0.7254625	6.45E-10	1.18E-05	0.418	0.824
THY1	10	-0.7260486	1.53E-12	2.79E-08	0.143	0.706
SLC14A1	10	-0.744809	6.62E-13	1.21E-08	0.194	0.765
RPLP0	10	-0.7633075	6.74E-20	1.23E-15	1	1

RPL10A	10	-0.7705208	7.79E-19	1.42E-14	1	1
CYR61	10	-0.7897174	5.01E-09	9.14E-05	0.112	0.549
YBX3	10	-0.7946414	8.08E-13	1.47E-08	0.694	1
APOD	10	-0.7956419	8.87E-12	1.62E-07	0.092	0.608
LECT1	10	-0.80522	5.16E-08	0.00094076	0.816	0.961
MT-ND1	10	-0.8177229	8.85E-11	1.61E-06	0.5	0.902
TCF4	10	-0.8208062	5.63E-12	1.03E-07	0.245	0.745
B2M	10	-0.8218098	5.55E-14	1.01E-09	1	1
C2orf82	10	-0.824383	4.09E-10	7.46E-06	0.949	1
MT-CO2	10	-0.8322615	1.15E-09	2.09E-05	0.959	0.961
RPS8	10	-0.8342398	1.68E-21	3.06E-17	1	1
SPARC	10	-0.8491695	3.51E-07	0.00640866	0.418	0.765
EEF2	10	-0.858405	1.12E-15	2.05E-11	0.99	1
ACTG1	10	-0.8614977	7.70E-13	1.40E-08	0.898	1
MT-CO3	10	-0.8968608	2.74E-10	5.00E-06	0.857	0.98
PAX1	10	-0.9044563	3.27E-12	5.96E-08	0.418	0.902
NEAT1	10	-0.9121101	2.24E-13	4.09E-09	0.969	1
CRISPLD1	10	-0.917621	7.42E-10	1.35E-05	0.082	0.51
MT-ND2	10	-0.9337137	1.39E-09	2.54E-05	0.551	0.882
RPS20	10	-0.9450218	5.64E-22	1.03E-17	1	1
CRTAP	10	-0.9479414	2.41E-18	4.39E-14	0.541	0.961
MT-CO1	10	-0.9523707	4.13E-11	7.53E-07	0.939	0.961
MT-ATP6	10	-0.9657403	1.96E-10	3.57E-06	0.735	0.961
EIF3H	10	-0.9701111	9.37E-20	1.71E-15	0.949	1
ZFP36L2	10	-0.9753451	1.12E-10	2.04E-06	0.194	0.667
COL2A1	10	-1.0649562	1.62E-08	0.00029538	0.531	0.902
RPS11	10	-1.0659063	8.15E-22	1.49E-17	1	1
SLC40A1	10	-1.070877	8.64E-17	1.58E-12	0.071	0.706
MT-CYB	10	-1.100156	7.87E-11	1.44E-06	0.561	0.902
ADIRF	10	-1.2346491	1.16E-15	2.11E-11	0.306	0.882
MT-ND3	10	-1.2749124	4.35E-14	7.94E-10	0.469	0.922
CLEC3A	10	-1.2761795	2.77E-16	5.05E-12	0.306	0.941
CAPS	10	-1.3629759	1.88E-16	3.43E-12	0.51	0.941
SPTSSB	10	-1.4516495	2.43E-18	4.43E-14	0.01	0.647
FRZB	10	-1.6412971	4.86E-13	8.88E-09	0.306	0.843
OGN	10	-1.6656193	2.85E-18	5.21E-14	0.878	1
SPP1	11	2.36128865	8.47E-13	1.55E-08	0.671	0.061
**gene**	**cluster**	**avg_logFC**	**p_val**	**p_val_adj**	**pct.1**	**pct.2**
COMP	11	1.38278093	1.36E-09	2.48E-05	0.857	0.5

FN1	11	1.29006633	4.55E-07	0.00830923	0.971	1
MGST1	11	1.16618571	8.50E-12	1.55E-07	0.8	0.242
PLA2G2A	11	1.15916384	6.15E-07	0.01122893	0.543	0.136
DDIT4L	11	1.04780396	2.67E-08	0.00048722	0.743	0.318
TGFBI	11	1.02107966	3.75E-09	6.84E-05	0.529	0.076
SDCBP	11	0.99852883	2.34E-08	0.00042685	0.829	0.53
PRG4	11	0.98950995	1.62E-07	0.00295218	0.4	0.03
VEGFA	11	0.98771543	1.57E-07	0.00286347	0.643	0.303
CHI3L2	11	0.82947638	6.28E-08	0.00114655	0.743	0.318
DNAJB9	11	0.81101025	1.83E-06	0.03346588	0.943	0.727
NT5C3A	11	0.80615694	6.82E-07	0.01245336	0.571	0.197
ARF4	11	0.77754816	6.21E-07	0.01133921	0.943	0.773
SLC3A2	11	0.76457188	2.20E-07	0.00400579	0.943	0.742
PTGS2	11	0.7586044	3.48E-07	0.00635578	0.386	0.03
CD55	11	0.74278732	7.65E-10	1.40E-05	0.771	0.379
ARF6	11	0.70903023	4.77E-09	8.71E-05	0.829	0.379
IFRD1	11	0.65072741	7.84E-10	1.43E-05	0.914	0.576
SPHK1	11	0.64849132	1.14E-09	2.08E-05	0.529	0.045
DDIT3	11	0.63021111	1.48E-06	0.0270117	0.929	0.758
STK17A	11	0.62826437	1.27E-10	2.31E-06	0.714	0.182
ATP6V0B	11	0.60445372	2.02E-07	0.00369265	0.957	0.667
VAPA	11	0.60040513	1.55E-10	2.83E-06	1	0.864
EMP3	11	0.59773439	1.26E-07	0.00229181	0.929	0.758
PID1	11	0.511174	2.56E-06	0.04670102	0.557	0.182
SMIM3	11	0.43759639	2.10E-06	0.03829082	0.4	0.061
KIAA1033	11	0.43206981	3.89E-07	0.00710099	0.457	0.076
YBX1	11	-0.3003026	2.23E-06	0.04070019	1	1
RPL35	11	-0.312594	1.30E-07	0.00236658	1	1
RPL15	11	-0.3253437	1.08E-06	0.01971576	1	1
RPS7	11	-0.3310814	2.42E-08	0.00044102	1	1
RPL22	11	-0.3370612	3.32E-07	0.00605026	1	1
RPL21	11	-0.3491214	3.84E-07	0.00700938	1	1
RPL13A	11	-0.3617253	7.29E-08	0.00133022	1	1
RPL13	11	-0.366451	6.79E-07	0.0123921	1	1
RPL27	11	-0.3727636	1.23E-07	0.00224549	1	1
RPS10	11	-0.3771028	1.86E-07	0.00338505	1	1
CCDC102B	11	-0.3811281	7.77E-07	0.01416984	0.014	0.333
RPS2	11	-0.3918505	8.65E-08	0.00157921	1	1
RPL18A	11	-0.3963486	3.76E-09	6.86E-05	1	1

RPS6	11	-0.4015937	3.54E-09	6.47E-05	1	1
RPS18	11	-0.4017462	6.13E-07	0.01117959	1	1
RPL37A	11	-0.4066084	2.62E-10	4.79E-06	1	1
RPL31	11	-0.4106693	6.54E-07	0.01193814	1	1
RPS27A	11	-0.4118398	5.98E-09	0.00010918	1	1
BRK1	11	-0.4155154	8.13E-07	0.01484205	0.7	0.955
RPL18	11	-0.4229578	2.09E-09	3.82E-05	1	1
RPS23	11	-0.4252295	1.09E-07	0.00199312	1	1
RPL19	11	-0.4337376	2.30E-11	4.19E-07	1	1
RPS19	11	-0.4356126	4.92E-10	8.98E-06	1	1
SERTAD4-AS1	11	-0.4361347	3.01E-07	0.00548921	0.029	0.379
RPLP1	11	-0.4400499	1.12E-11	2.04E-07	1	1
RPS12	11	-0.4477579	4.49E-08	0.0008195	1	1
EID1	11	-0.4486803	1.95E-08	0.00035672	0.929	1
FTH1	11	-0.4734049	2.53E-07	0.00461034	1	1
RPL29	11	-0.4773676	8.10E-10	1.48E-05	1	1
RPL37	11	-0.4862501	4.20E-12	7.67E-08	1	1
RPS29	11	-0.4972782	4.72E-14	8.61E-10	1	1
ZBTB20	11	-0.4998123	2.18E-06	0.03985438	0.171	0.53
UQCRB	11	-0.5002253	2.56E-08	0.00046734	0.971	1
NPC2	11	-0.5025268	2.39E-07	0.00436065	0.957	0.97
RPL30	11	-0.5048047	2.07E-13	3.77E-09	1	1
RPS13	11	-0.5080748	1.05E-11	1.92E-07	1	1
TOMM7	11	-0.5089099	4.16E-10	7.59E-06	0.986	1
RPS17	11	-0.5136211	3.72E-16	6.79E-12	1	1
KHDRBS3	11	-0.5146595	1.44E-08	0.00026246	0.014	0.409
RPL9	11	-0.5155455	1.12E-13	2.04E-09	1	1
CCDC8	11	-0.5219712	9.72E-08	0.00177312	0.043	0.424
RPL3	11	-0.5251664	9.37E-08	0.00171023	0.986	1
TUBB	11	-0.5257142	4.28E-07	0.00781503	0.757	0.924
CP	11	-0.5299029	5.26E-08	0.00096001	0.329	0.803
RPS16	11	-0.5313713	1.44E-11	2.63E-07	1	1
MALAT1	11	-0.5336387	1.19E-07	0.00217413	1	1
YBX3	11	-0.5357646	1.82E-07	0.00331299	0.729	0.97
B2M	11	-0.5388648	4.18E-08	0.00076236	1	1
RPLP2	11	-0.5421644	3.74E-13	6.82E-09	1	1
P4HB	11	-0.5425852	3.10E-07	0.00565514	0.771	0.985
GLTSCR2	11	-0.5539178	2.60E-07	0.00475105	0.757	0.985
EIF3L	11	-0.5609324	7.68E-07	0.01400457	0.771	0.955

PEBP1	11	-0.5675914	2.51E-07	0.0045758	0.757	0.924
S100A13	11	-0.5677686	4.75E-08	0.00086604	0.957	1
NGFRAP1	11	-0.5716945	1.36E-07	0.00248762	0.771	0.939
RPL23	11	-0.5768238	2.92E-09	5.33E-05	0.986	1
EEF1B2	11	-0.5788828	9.93E-09	0.00018118	0.957	1
RPSA	11	-0.5797277	7.06E-09	0.00012889	0.971	1
RPL7	11	-0.5826358	1.21E-11	2.21E-07	1	1
RPL23A	11	-0.5955264	5.19E-12	9.47E-08	1	1
RPS3A	11	-0.5964054	1.68E-14	3.06E-10	1	1
NEAT1	11	-0.6050749	7.05E-08	0.00128566	0.986	1
CHRDL2	11	-0.6125681	2.14E-06	0.03896429	0.043	0.364
EIF4B	11	-0.6148785	1.21E-07	0.00220239	0.629	0.909
EIF3H	11	-0.6377495	1.04E-08	0.00018994	0.871	0.985
CITED2	11	-0.6382288	1.14E-06	0.02088373	0.229	0.606
FXYD6	11	-0.6662742	8.66E-11	1.58E-06	0.6	0.97
TPT1	11	-0.6735686	3.28E-12	5.98E-08	1	1
RPS8	11	-0.6790103	8.66E-13	1.58E-08	1	1
CRTAP	11	-0.6817834	1.03E-08	0.0001876	0.443	0.818
SPARC	11	-0.6929893	5.25E-09	9.58E-05	0.257	0.758
RPL10A	11	-0.740579	6.73E-12	1.23E-07	1	0.985
CAPS	11	-0.7545225	1.11E-06	0.0203453	0.543	0.909
HNRNPA1	11	-0.7608766	2.72E-10	4.96E-06	0.943	1
RPL4	11	-0.7693492	2.48E-17	4.52E-13	1	1
EEF2	11	-0.80294	8.81E-10	1.61E-05	0.957	0.97
CCNI	11	-0.8064971	3.78E-15	6.89E-11	0.971	1
PLAC9	11	-0.831619	9.04E-08	0.00164883	0.6	0.879
RPLP0	11	-0.8604061	3.97E-16	7.25E-12	1	1
EEF1A1	11	-0.8874561	1.03E-08	0.00018724	1	1
ADIRF	11	-0.8897201	1.24E-08	0.00022565	0.457	0.894
RPS11	11	-0.9544895	8.97E-21	1.64E-16	0.986	1
PAX1	11	-0.9687618	1.50E-11	2.74E-07	0.357	0.909
COL2A1	11	-0.9849192	2.17E-13	3.95E-09	0.3	0.909
RPS20	11	-1.0527418	3.16E-20	5.76E-16	0.957	1
OGN	11	-1.1033124	6.47E-10	1.18E-05	0.529	0.955
CLEC3A	11	-1.2628133	8.42E-10	1.54E-05	0.243	0.727
SPTSSB	11	-1.3938416	3.69E-14	6.74E-10	0	0.606
SLPI	11	-1.7754131	3.50E-07	0.00639374	0.929	1
FRZB	11	-2.185239	5.92E-14	1.08E-09	0.114	0.742
**gene**	**cluster**	**avg_logFC**	**p_val**	**p_val_adj**	**pct.1**	**pct.2**

SPP1	12	2.1653184	2.99E-12	5.46E-08	0.75	0.087
COMP	12	2.13197035	1.71E-17	3.11E-13	0.99	0.674
PRG4	12	1.9056579	1.48E-09	2.70E-05	0.56	0.022
PTGS2	12	1.69615063	1.56E-15	2.85E-11	0.8	0.043
CHI3L2	12	1.47984346	1.31E-10	2.39E-06	0.96	0.783
PLA2G2A	12	1.37491036	2.00E-10	3.65E-06	0.77	0.152
MT1X	12	1.35512314	1.63E-06	0.02981531	1	0.978
VEGFA	12	1.35135271	3.41E-15	6.22E-11	0.91	0.283
TGFBI	12	1.32760536	2.61E-14	4.76E-10	0.82	0.13
IGFBP3	12	1.19392247	1.51E-07	0.00275223	0.44	0
ERO1A	12	1.17144961	2.91E-10	5.30E-06	0.79	0.304
SLC2A1	12	1.0182564	1.02E-11	1.86E-07	0.75	0.152
CA9	12	1.01262312	8.43E-11	1.54E-06	0.77	0.217
BHLHE40	12	0.95291192	1.10E-08	0.00020031	0.79	0.37
SOD2	12	0.92615775	4.74E-07	0.00864894	1	0.957
FN1	12	0.90736366	8.82E-13	1.61E-08	1	1
PTP4A1	12	0.86956244	2.73E-09	4.99E-05	0.78	0.326
PPP3CA	12	0.86777795	4.34E-09	7.92E-05	0.93	0.696
BZW1	12	0.85589864	8.92E-09	0.00016273	0.85	0.435
TIMP1	12	0.84144403	1.25E-06	0.02282832	1	1
TMEM45A	12	0.82851994	1.91E-08	0.00034796	0.94	0.783
SLC3A2	12	0.80785562	7.89E-08	0.00144017	0.81	0.522
GAPDH	12	0.74388554	1.06E-12	1.94E-08	1	1
TREM1	12	0.72754861	6.09E-08	0.00111111	0.46	0
CCDC85B	12	0.71548573	9.51E-08	0.00173515	0.96	0.848
ENO2	12	0.69080678	4.88E-08	0.00088975	0.81	0.413
GBE1	12	0.68980828	1.34E-06	0.02446321	0.8	0.565
AK2	12	0.62794789	1.09E-07	0.00199483	0.92	0.63
SLC25A36	12	0.59472769	5.67E-08	0.00103525	0.75	0.348
ARPP19	12	0.5827369	2.75E-07	0.00501103	0.79	0.413
ATP6V1G1	12	0.56144699	5.90E-08	0.00107708	0.98	0.978
NAMPT	12	0.5282157	2.38E-06	0.04350577	0.82	0.543
PHPT1	12	0.49014261	1.20E-06	0.02181302	0.89	0.565
RPS14	12	-0.2657032	1.91E-06	0.03483105	1	1
RPL35A	12	-0.2872744	1.32E-07	0.00240187	1	1
KHDRBS3	12	-0.2885427	8.14E-07	0.01484988	0.01	0.261
AGT	12	-0.2933547	2.26E-06	0.04122732	0.06	0.37
RPS21	12	-0.2973327	1.62E-06	0.02957353	1	1
RPS28	12	-0.2980332	4.22E-08	0.00077056	1	1

RPL7A	12	-0.2982749	1.89E-07	0.00345086	1	1
RPS25	12	-0.3020927	1.39E-07	0.00254347	1	1
RPL35	12	-0.3117989	8.94E-09	0.00016312	1	1
RPL27A	12	-0.3169626	7.95E-09	0.00014506	1	1
RPS3	12	-0.3175156	1.98E-09	3.62E-05	1	1
RPL3	12	-0.3179153	1.21E-07	0.00221205	1	1
FTL	12	-0.3247783	7.74E-07	0.01412453	1	1
RPS9	12	-0.3325275	5.27E-09	9.62E-05	1	1
RPSA	12	-0.3344645	1.56E-06	0.02838444	1	1
C14orf1	12	-0.334696	2.20E-06	0.04017465	0.11	0.457
RPL37A	12	-0.3448822	1.92E-09	3.50E-05	1	1
RPS6	12	-0.3465861	5.85E-10	1.07E-05	1	1
RPL13	12	-0.3478486	1.34E-10	2.45E-06	1	1
RPS4X	12	-0.3533099	6.14E-09	0.00011211	1	1
NACA	12	-0.3540809	1.73E-08	0.00031493	1	1
SNRPA	12	-0.361847	8.38E-07	0.01528893	0.09	0.435
RPL21	12	-0.3621964	4.51E-11	8.22E-07	1	1
RPLP1	12	-0.371695	7.33E-09	0.00013374	1	1
RPS27	12	-0.3744513	1.46E-11	2.67E-07	1	1
RPL8	12	-0.3896107	4.94E-10	9.01E-06	1	1
BGN	12	-0.3945471	1.68E-06	0.03059281	1	1
CXXC5	12	-0.3948178	1.37E-06	0.02502633	0.04	0.326
RPL18A	12	-0.3965494	1.75E-10	3.19E-06	1	1
RPS5	12	-0.4019529	2.64E-10	4.81E-06	1	1
RPL13A	12	-0.4055957	4.78E-13	8.73E-09	1	1
UBA52	12	-0.4077179	4.45E-11	8.11E-07	1	1
RPS18	12	-0.4085125	1.85E-10	3.37E-06	1	1
RPL29	12	-0.4181202	9.72E-11	1.77E-06	1	1
RPL9	12	-0.4219641	7.26E-12	1.32E-07	1	1
RPS23	12	-0.4251653	1.20E-12	2.18E-08	1	1
VIM	12	-0.4270429	5.12E-08	0.00093508	1	1
S100A13	12	-0.4279007	1.39E-06	0.02531325	0.97	1
BTF3	12	-0.4279896	6.57E-10	1.20E-05	1	1
HINT1	12	-0.4308283	2.16E-06	0.03936143	0.93	1
RPL27	12	-0.4322871	3.66E-09	6.68E-05	1	1
RPL37	12	-0.4340183	2.73E-12	4.98E-08	1	1
TXN	12	-0.438203	1.84E-07	0.0033609	0.56	0.935
ATP5G2	12	-0.4426469	3.17E-08	0.00057844	1	1
RPL14	12	-0.449427	6.37E-14	1.16E-09	1	1

RPL30	12	-0.4498543	5.79E-11	1.06E-06	1	1
RAC1	12	-0.4560569	3.73E-07	0.00680071	0.82	0.978
COMMD6	12	-0.4572984	2.87E-09	5.25E-05	1	1
NPM1	12	-0.4583933	1.18E-08	0.00021494	0.99	1
S100A1	12	-0.4597637	1.37E-06	0.02493779	1	1
GSTP1	12	-0.4622159	3.33E-09	6.08E-05	0.96	1
EIF3D	12	-0.4626564	1.32E-06	0.02417121	0.74	0.891
RPL10	12	-0.465285	2.20E-12	4.01E-08	1	1
PPIA	12	-0.4718053	4.38E-07	0.0079886	0.87	1
RPL19	12	-0.473458	3.63E-16	6.62E-12	1	1
RPS2	12	-0.4761027	5.50E-14	1.00E-09	1	1
IER2	12	-0.4772859	5.88E-07	0.01072938	0.13	0.5
SEC11A	12	-0.4801348	1.97E-07	0.00360062	0.72	0.978
MTDH	12	-0.4806834	1.14E-06	0.02089216	0.4	0.804
PLAC9	12	-0.4897595	1.78E-06	0.0324549	0.72	0.978
C19orf43	12	-0.4929535	1.05E-06	0.01917931	0.68	0.913
PFN1	12	-0.4966899	6.44E-07	0.01175552	0.74	0.935
PSMA7	12	-0.5077152	5.24E-08	0.00095583	0.72	0.957
HNRNPA1	12	-0.5080295	6.85E-09	0.00012503	0.98	1
CCDC102B	12	-0.5082648	1.82E-11	3.32E-07	0.02	0.457
MYL6	12	-0.5084937	5.21E-08	0.00095122	0.84	0.978
RPS27A	12	-0.5101565	2.10E-16	3.84E-12	1	1
EIF3L	12	-0.5108256	1.93E-07	0.00352835	0.8	1
RPL23	12	-0.5112008	1.81E-09	3.30E-05	0.99	1
RPS7	12	-0.5188029	1.84E-13	3.36E-09	1	1
RPS3A	12	-0.5218197	9.20E-16	1.68E-11	1	1
HLA-C	12	-0.5236506	4.31E-09	7.86E-05	0.95	1
P4HB	12	-0.5298121	2.52E-06	0.04605434	0.68	0.913
RPS17	12	-0.5339444	8.31E-13	1.52E-08	1	1
RPL23A	12	-0.5353327	2.10E-15	3.83E-11	1	1
BRK1	12	-0.5381178	1.01E-07	0.0018452	0.67	0.935
PRUNE2	12	-0.5384437	6.28E-09	0.00011461	0.16	0.609
RPS29	12	-0.5400445	3.28E-13	5.99E-09	1	1
RPS13	12	-0.5528283	1.77E-15	3.23E-11	1	1
TCF4	12	-0.5555381	2.91E-07	0.00531863	0.12	0.5
EEF1B2	12	-0.5569002	7.87E-10	1.44E-05	0.95	1
HOXC10	12	-0.5673183	2.06E-07	0.00375733	0.55	0.87
HLA-B	12	-0.5710469	2.55E-08	0.00046529	0.91	1
KDELR2	12	-0.5721651	2.03E-08	0.00037024	0.53	0.913

SSR3	12	-0.5819133	5.25E-08	0.00095857	0.77	0.935
TUBB	12	-0.5830339	1.38E-07	0.00251505	0.51	0.913
HLA-A	12	-0.5851871	1.50E-07	0.00273109	0.99	0.978
RPL34	12	-0.5861989	9.25E-17	1.69E-12	1	1
ISLR	12	-0.5893172	7.73E-07	0.01410586	0.21	0.565
ATRNL1	12	-0.5904265	2.47E-10	4.51E-06	0.06	0.5
RPS16	12	-0.5924019	2.46E-17	4.49E-13	1	1
RPLP2	12	-0.5950844	2.02E-15	3.69E-11	1	1
RPL18	12	-0.5981102	5.68E-17	1.04E-12	1	1
EID1	12	-0.6128361	3.60E-10	6.56E-06	0.93	1
RPL7	12	-0.6138012	1.40E-15	2.55E-11	1	1
TPM1	12	-0.6223781	1.37E-06	0.02496595	0.26	0.674
THY1	12	-0.623766	1.62E-06	0.02952433	0.14	0.5
EEF1A1	12	-0.6544882	9.27E-19	1.69E-14	1	1
CP	12	-0.6603119	1.53E-09	2.79E-05	0.56	1
RPLP0	12	-0.6777741	2.07E-15	3.78E-11	1	1
CD9	12	-0.6959899	8.72E-07	0.01590906	0.4	0.761
SLC40A1	12	-0.6964861	1.39E-11	2.54E-07	0.04	0.5
TPT1	12	-0.7103659	6.34E-20	1.16E-15	1	1
RPL10A	12	-0.7215033	1.59E-16	2.90E-12	1	1
COL2A1	12	-0.7300088	3.72E-08	0.00067796	0.49	0.848
NPC2	12	-0.7301027	2.65E-15	4.83E-11	0.96	1
RPS8	12	-0.7308014	2.24E-16	4.09E-12	1	1
SLC25A3	12	-0.7367885	6.40E-12	1.17E-07	0.54	0.978
RPL4	12	-0.7412238	6.18E-17	1.13E-12	1	1
H3F3A	12	-0.7516828	1.79E-10	3.26E-06	0.82	1
YBX3	12	-0.7518362	6.76E-12	1.23E-07	0.63	0.957
INHBA	12	-0.7852625	4.06E-10	7.40E-06	0.1	0.565
GREM1	12	-0.795577	2.85E-08	0.00052065	0.03	0.37
EIF3H	12	-0.7988511	1.11E-14	2.02E-10	0.95	1
CRTAP	12	-0.8009202	7.91E-14	1.44E-09	0.39	0.935
CCNI	12	-0.8038342	2.18E-14	3.97E-10	0.97	1
ID3	12	-0.8082775	6.00E-08	0.00109398	0.23	0.652
RHOBTB3	12	-0.8135701	2.31E-08	0.00042234	0.35	0.739
MT-ND4	12	-0.8319911	6.47E-08	0.00118066	0.89	0.957
CITED2	12	-0.8395418	5.89E-07	0.01075431	0.16	0.522
NEAT1	12	-0.8479518	7.47E-12	1.36E-07	0.99	1
ACTG1	12	-0.8488495	1.34E-11	2.44E-07	0.91	1
MT-ND1	12	-0.8532214	4.86E-10	8.86E-06	0.24	0.717

CAPS	12	-0.8605595	3.24E-09	5.91E-05	0.64	0.957
FTH1	12	-0.8774083	2.98E-15	5.43E-11	1	1
ZFP36L2	12	-0.889202	3.47E-11	6.33E-07	0.07	0.543
TSC22D1	12	-0.8924268	4.04E-09	7.38E-05	0.52	0.935
B2M	12	-0.8984013	3.28E-16	5.98E-12	1	1
SPARC	12	-0.9318217	5.76E-13	1.05E-08	0.19	0.761
MT-ATP6	12	-0.9788692	2.54E-11	4.63E-07	0.64	0.935
GPX3	12	-0.9828652	1.10E-11	2.01E-07	0.92	1
MT-CYB	12	-0.9893096	2.38E-09	4.34E-05	0.42	0.804
C2orf82	12	-1.002728	1.97E-12	3.59E-08	0.92	1
RPS11	12	-1.0150968	1.41E-20	2.58E-16	0.99	1
CYR61	12	-1.0395182	1.93E-06	0.03525837	0.16	0.5
RPS20	12	-1.0482208	2.28E-21	4.15E-17	0.99	1
PAX1	12	-1.0498221	5.45E-14	9.95E-10	0.42	0.935
MT-CO3	12	-1.0966143	2.83E-10	5.16E-06	0.74	0.935
MT-ND2	12	-1.0978018	3.40E-13	6.21E-09	0.3	0.848
CLEC3A	12	-1.1061018	1.18E-10	2.15E-06	0.41	0.87
MGP	12	-1.1491314	1.76E-10	3.22E-06	0.99	1
MT-CO1	12	-1.162885	4.47E-11	8.16E-07	0.89	0.978
OGN	12	-1.2486699	4.96E-10	9.05E-06	0.93	1
MT-CO2	12	-1.3130904	6.40E-12	1.17E-07	0.78	1
ADIRF	12	-1.3883303	5.01E-15	9.15E-11	0.29	0.848
MT-ND3	12	-1.4187312	5.02E-18	9.16E-14	0.26	0.913
SPTSSB	12	-1.6847873	4.14E-22	7.55E-18	0	0.739
FRZB	12	-2.4847232	6.44E-19	1.18E-14	0.28	0.935
**gene**	**cluster**	**avg_logFC**	**p_val**	**p_val_adj**	**pct.1**	**pct.2**
PLA2G2A	13	2.20542134	2.73E-07	0.00497642	0.867	0.143
MT1G	13	1.97263322	1.90E-06	0.03472442	1	0.619
CHI3L2	13	1.81807678	2.99E-07	0.00545487	0.933	0.19
TGFBI	13	1.56800299	3.63E-07	0.0066215	0.933	0.381
RPS20	13	-0.8020567	7.62E-08	0.00139086	1	1
RPS11	13	-0.9206104	3.44E-07	0.00627246	0.967	1
S100A4	13	-1.5733487	2.45E-06	0.04470656	0.7	0.952
