# Supplementary material for: Single-Cell RNA-Seq Analysis of Cells from Degenerating and Non-Degenerating Intervertebral Discs from the Same Individual Reveals New Biomarkers for Intervertebral Disc Degeneration
Source: Int J Mol Sci. 2022 Apr 3;23(7):3993. doi: 10.3390/ijms23073993 (PMC8999935; doi:10.3390/ijms23073993)
Supplement: Supplementary file 1 [file ijms-23-03993-s001.zip › ijms-1655681-SI/Table S13.pdf]

Table S13. Differentially expressed genes between NPD and NPnD.

gene	cluster	avg_logFC	p_val	p_val_adj	pct.1	pct.2
SPP1	0	2.78782255	1.54E-199	2.82E-195	0.897	0.086
PRG4	0	1.90126201	1.11E-136	2.03E-132	0.637	0.018
MT2A	0	1.89772779	6.00E-172	1.10E-167	1	0.996
UPP1	0	1.41408436	2.39E-198	4.35E-194	0.875	0.106
HMGA1	0	1.30616375	8.76E-161	1.60E-156	0.858	0.208
PTGS2	0	1.30474284	2.09E-145	3.82E-141	0.692	0.047
SOD2	0	1.29017351	5.31E-117	9.68E-113	1	0.932
CTSL	0	1.1837381	3.65E-92	6.65E-88	0.809	0.383
WTAP	0	1.18225712	2.11E-129	3.86E-125	0.942	0.519
COMP	0	1.16669886	3.20E-99	5.85E-95	0.951	0.6
VEGFA	0	1.14778831	2.47E-136	4.50E-132	0.863	0.313
IGFBP3	0	1.13277203	1.23E-109	2.25E-105	0.569	0.037
S100A2	0	1.13116397	1.35E-93	2.46E-89	0.72	0.233
NRP2	0	1.08826992	1.28E-107	2.33E-103	0.606	0.079
TM4SF1	0	1.07669152	4.17E-89	7.62E-85	0.777	0.324
PLCG2	0	1.00152984	8.90E-38	1.62E-33	0.279	0.037
SERPINE2	0	0.98230006	3.37E-70	6.15E-66	0.997	0.985
CD55	0	0.97524178	4.64E-116	8.46E-112	0.922	0.528
BHLHE40	0	0.95666799	6.14E-85	1.12E-80	0.728	0.314
C11orf96	0	0.95603502	8.01E-99	1.46E-94	0.528	0.033
MT1X	0	0.92181199	3.03E-47	5.53E-43	0.996	0.994
BZW1	0	0.89574182	1.28E-114	2.33E-110	0.85	0.349
STK17A	0	0.8907205	2.64E-93	4.82E-89	0.623	0.134
DDIT4L	0	0.88975078	6.67E-78	1.22E-73	0.899	0.553
ERO1A	0	0.88788093	1.58E-112	2.88E-108	0.751	0.217
TIMP3	0	0.87113364	1.28E-53	2.34E-49	0.749	0.448
TNFRSF12A	0	0.85553223	6.97E-93	1.27E-88	0.601	0.11
FN1	0	0.8379634	3.23E-86	5.89E-82	1	0.994
NDRG1	0	0.83766747	2.13E-96	3.88E-92	0.767	0.326
MIF	0	0.81931684	4.24E-162	7.73E-158	1	1
SMOX	0	0.79743283	1.10E-64	2.00E-60	0.581	0.204
MRPS6	0	0.78122619	3.90E-49	7.12E-45	0.958	0.883
CXCL8	0	0.77989509	3.16E-30	5.77E-26	0.182	0.007
PMP22	0	0.75197113	4.28E-87	7.82E-83	0.926	0.617
RNF145	0	0.75157743	2.42E-72	4.42E-68	0.633	0.24
SLC3A2	0	0.75032419	3.23E-70	5.90E-66	0.619	0.232

SNHG12	0	0.74656166	7.09E-77	1.29E-72	0.743	0.364
RGCC	0	0.74192845	1.78E-59	3.24E-55	0.911	0.654
CCDC85B	0	0.73548172	1.02E-63	1.86E-59	0.899	0.652
BMP2	0	0.70424744	1.13E-73	2.07E-69	0.412	0.021
RABGGTB	0	0.70166566	2.17E-93	3.95E-89	0.878	0.527
PPP1R15A	0	0.69668284	4.14E-79	7.55E-75	0.589	0.146
SLC2A1	0	0.69434838	2.99E-79	5.45E-75	0.531	0.09
RELB	0	0.69029411	7.42E-77	1.35E-72	0.566	0.131
TREM1	0	0.67697834	1.03E-85	1.87E-81	0.434	0.006
TIMP1	0	0.66474358	2.84E-16	5.19E-12	1	0.999
ARF6	0	0.66270048	4.26E-72	7.77E-68	0.728	0.36
VAPA	0	0.65037407	1.54E-90	2.80E-86	0.984	0.865
TMSB10	0	0.6460066	2.13E-63	3.89E-59	0.996	0.981
ENO2	0	0.64427492	3.74E-78	6.82E-74	0.668	0.252
TMSB4X	0	0.63092405	5.83E-27	1.06E-22	0.841	0.651
ARPP19	0	0.62451258	1.34E-66	2.44E-62	0.724	0.394
GSTO1	0	0.6127789	1.94E-73	3.54E-69	0.906	0.648
TGFBI	0	0.60966341	6.61E-61	1.21E-56	0.513	0.132
EIF4EBP1	0	0.60529452	2.10E-38	3.83E-34	0.602	0.343
PGK1	0	0.60487818	5.84E-66	1.07E-61	0.958	0.778
AK2	0	0.60255144	1.63E-57	2.97E-53	0.765	0.476
ATP6V1G1	0	0.59342763	1.87E-76	3.40E-72	0.988	0.923
BAALC	0	0.58119376	8.86E-31	1.62E-26	0.545	0.323
GOLT1B	0	0.57801398	6.30E-59	1.15E-54	0.577	0.217
ENO1	0	0.57301533	8.03E-56	1.46E-51	0.993	0.972
G0S2	0	0.57280882	1.86E-09	3.40E-05	0.265	0.152
CSGALNACT2	0	0.56449252	3.93E-59	7.16E-55	0.442	0.081
GBE1	0	0.5605454	1.55E-40	2.83E-36	0.675	0.444
HLA-DRB1	0	0.5577166	8.16E-56	1.49E-51	0.484	0.127
PTP4A1	0	0.55718528	3.19E-41	5.82E-37	0.582	0.306
PHLDA1	0	0.55337871	2.27E-50	4.15E-46	0.301	0.017
ISG20	0	0.55148282	1.55E-46	2.83E-42	0.606	0.297
MMP3	0	0.55099964	1.86E-21	3.40E-17	0.79	0.554
DNER	0	0.5502186	7.02E-41	1.28E-36	0.637	0.388
DNAJB9	0	0.54856595	1.77E-43	3.22E-39	0.78	0.524
SLC25A37	0	0.54624056	2.18E-60	3.98E-56	0.935	0.694
PFDN2	0	0.54464457	1.25E-59	2.29E-55	0.741	0.448
ABI3BP	0	0.5445588	6.97E-33	1.27E-28	0.549	0.294
PPFIBP1	0	0.54351266	5.42E-47	9.89E-43	0.605	0.294

PPP3CA	0	0.54156198	6.12E-44	1.12E-39	0.858	0.597
CCND1	0	0.53654814	2.93E-62	5.34E-58	0.424	0.058
ZNF593	0	0.53403785	2.52E-53	4.60E-49	0.663	0.338
RGS16	0	0.53251056	2.96E-16	5.40E-12	0.33	0.168
MT1G	0	0.52559053	1.52E-23	2.77E-19	0.936	0.846
KLF3	0	0.51974845	2.57E-36	4.69E-32	0.464	0.19
CLEC2B	0	0.51689653	1.99E-13	3.64E-09	0.285	0.143
TGM2	0	0.51645548	2.04E-53	3.72E-49	0.504	0.148
SGIP1	0	0.51590244	1.55E-66	2.82E-62	0.363	0.011
RNF114	0	0.51399922	1.80E-43	3.28E-39	0.698	0.461
HILPDA	0	0.51382255	8.71E-22	1.59E-17	0.561	0.357
PPP1CB	0	0.51165008	1.47E-34	2.68E-30	0.847	0.628
C15orf48	0	0.5081878	1.81E-37	3.31E-33	0.219	0.007
COX17	0	0.50201812	2.62E-50	4.77E-46	0.851	0.611
DNAJB1	0	0.50134688	1.29E-25	2.36E-21	0.374	0.152
SPRY2	0	0.49922008	1.28E-64	2.33E-60	0.393	0.032
DDIT3	0	0.49671231	1.32E-37	2.40E-33	0.598	0.32
EMP3	0	0.4882122	2.48E-42	4.52E-38	0.924	0.771
SLC7A2	0	0.48729151	5.35E-46	9.76E-42	0.32	0.037
ARF4	0	0.48348043	5.11E-43	9.32E-39	0.817	0.568
PLAUR	0	0.48341142	2.86E-47	5.22E-43	0.464	0.141
TMEM45A	0	0.48077328	1.38E-34	2.51E-30	0.847	0.661
MED21	0	0.47508721	3.18E-38	5.80E-34	0.599	0.317
TMEM38B	0	0.47504725	1.27E-45	2.32E-41	0.7	0.433
EIF5	0	0.47144216	2.82E-42	5.15E-38	0.712	0.426
HMGA2	0	0.47077812	3.56E-67	6.49E-63	0.347	0.001
GPX1	0	0.46652536	2.96E-46	5.40E-42	0.955	0.794
CLK1	0	0.46626177	1.74E-39	3.18E-35	0.694	0.444
ZNF385D	0	0.4654165	2.65E-37	4.83E-33	0.741	0.512
NTMT1	0	0.46168462	2.16E-43	3.94E-39	0.55	0.248
NBPF14	0	0.46036022	5.94E-58	1.08E-53	0.435	0.076
CHD2	0	0.45511017	1.01E-23	1.85E-19	0.386	0.178
GRB10	0	0.45507561	4.21E-58	7.69E-54	0.324	0.01
EPS8	0	0.45423799	1.96E-45	3.57E-41	0.471	0.153
EIF1B	0	0.45318228	2.35E-47	4.29E-43	0.837	0.606
COX7A2	0	0.45181149	5.79E-71	1.06E-66	0.992	0.953
IER3	0	0.44725588	2.87E-26	5.24E-22	0.365	0.139
NPR3	0	0.44649899	3.26E-59	5.95E-55	0.312	0.001
WDR33	0	0.44561927	3.10E-36	5.65E-32	0.595	0.335

ANXA5	0	0.44463907	2.02E-43	3.69E-39	0.916	0.73
EIF1	0	0.44380114	1.60E-86	2.92E-82	1	1
SELK	0	0.44278268	1.16E-46	2.12E-42	0.926	0.771
TFPI	0	0.44158686	1.07E-36	1.95E-32	0.302	0.054
FAM162A	0	0.43972377	2.29E-41	4.18E-37	0.927	0.737
XBP1	0	0.43930062	5.15E-30	9.40E-26	0.706	0.513
RND3	0	0.43870898	9.68E-38	1.77E-33	0.279	0.037
WDR45B	0	0.43528907	3.44E-42	6.29E-38	0.473	0.168
KMT2E-AS1	0	0.43192456	3.04E-32	5.54E-28	0.276	0.052
CHI3L2	0	0.43034422	5.90E-13	1.08E-08	0.554	0.415
SQSTM1	0	0.42630864	8.67E-38	1.58E-33	0.977	0.909
CTHRC1	0	0.42252523	8.58E-44	1.57E-39	0.314	0.04
VIMP	0	0.42075901	8.47E-39	1.55E-34	0.828	0.567
VMP1	0	0.4195411	3.84E-23	7.01E-19	0.609	0.414
ADSS	0	0.41417286	7.88E-29	1.44E-24	0.625	0.412
PTGES3	0	0.41232085	1.14E-51	2.08E-47	0.991	0.93
TRMT112	0	0.41152653	4.23E-45	7.72E-41	0.955	0.865
SH3BP5	0	0.41084195	3.00E-26	5.48E-22	0.511	0.291
SAR1A	0	0.40989703	3.89E-32	7.11E-28	0.553	0.301
IFRD1	0	0.40826968	3.13E-28	5.71E-24	0.635	0.455
EVI2A	0	0.40757259	4.37E-39	7.97E-35	0.351	0.079
VDAC1	0	0.40547767	2.07E-34	3.78E-30	0.907	0.752
NOL7	0	0.40384862	5.85E-35	1.07E-30	0.711	0.483
ZFAND5	0	0.40117494	6.03E-29	1.10E-24	0.618	0.396
SH3BGRL3	0	0.39948407	1.09E-42	2.00E-38	0.991	0.96
HNRNPDL	0	0.39279463	3.28E-36	5.98E-32	0.87	0.672
PID1	0	0.3906557	6.25E-27	1.14E-22	0.645	0.433
YAF2	0	0.39043162	5.16E-34	9.42E-30	0.463	0.193
PDE4B	0	0.38852208	5.59E-29	1.02E-24	0.389	0.141
NFKBIA	0	0.38764989	1.50E-15	2.74E-11	0.39	0.221
CRTAC1	0	0.38653075	3.84E-30	7.01E-26	0.321	0.09
CTNNB1	0	0.38527351	2.74E-14	5.01E-10	0.406	0.247
BTG3	0	0.38465314	2.67E-32	4.87E-28	0.477	0.208
RIPK2	0	0.38418941	1.78E-37	3.26E-33	0.387	0.112
GADD45GIP1	0	0.38380464	8.77E-36	1.60E-31	0.855	0.663
NT5E	0	0.38363614	6.24E-29	1.14E-24	0.407	0.164
SNRNP27	0	0.38183602	3.63E-30	6.62E-26	0.54	0.295
FOSL1	0	0.38090155	4.07E-43	7.43E-39	0.284	0.025
FABP5	0	0.37452847	4.60E-08	0.00083927	0.362	0.248

RGS2	0	0.36878569	8.38E-09	0.00015292	0.499	0.383
SEC61G	0	0.3685909	1.21E-28	2.21E-24	0.995	0.992
CLDND1	0	0.36730428	1.14E-28	2.08E-24	0.493	0.25
C2orf49	0	0.36544086	1.93E-39	3.52E-35	0.428	0.135
EIF4A2	0	0.36491972	5.76E-50	1.05E-45	1	0.999
RNMT	0	0.36277337	4.16E-23	7.59E-19	0.475	0.261
FAP	0	0.36241542	3.60E-40	6.56E-36	0.358	0.08
RHEB	0	0.36188234	2.47E-27	4.51E-23	0.873	0.735
FNIP2	0	0.3587928	2.07E-24	3.77E-20	0.403	0.179
SLC20A1	0	0.35746691	2.91E-33	5.32E-29	0.344	0.094
SAP18	0	0.35658248	1.24E-30	2.26E-26	0.873	0.734
SERPINE1	0	0.35633887	1.70E-42	3.11E-38	0.279	0.023
CGGBP1	0	0.35530445	1.76E-27	3.21E-23	0.538	0.312
POMP	0	0.35493206	8.72E-33	1.59E-28	0.877	0.709
TNS1	0	0.35416343	1.73E-38	3.16E-34	0.382	0.101
CAPZA2	0	0.35366561	1.61E-26	2.94E-22	0.753	0.578
OSER1	0	0.35322903	7.73E-25	1.41E-20	0.462	0.237
TVP23B	0	0.35056658	2.76E-30	5.03E-26	0.464	0.208
C11orf31	0	0.34706115	5.28E-30	9.63E-26	0.84	0.641
RNF126	0	0.34601765	2.48E-31	4.52E-27	0.412	0.157
CDC37L1	0	0.34578244	4.34E-21	7.92E-17	0.485	0.299
SLC25A36	0	0.34444347	7.10E-24	1.30E-19	0.56	0.348
PPP1R14C	0	0.3422112	1.09E-41	1.99E-37	0.313	0.046
CTTN	0	0.34160047	2.56E-15	4.67E-11	0.467	0.316
SMS	0	0.33984022	3.99E-18	7.28E-14	0.599	0.447
MSANTD3	0	0.33957615	7.78E-33	1.42E-28	0.394	0.131
RPL22L1	0	0.33909127	4.12E-23	7.52E-19	0.725	0.56
S100A16	0	0.33715143	6.51E-18	1.19E-13	0.41	0.236
SVIP	0	0.33536516	8.72E-21	1.59E-16	0.592	0.411
LINC00152	0	0.33426325	1.59E-18	2.89E-14	0.35	0.167
OAZ1	0	0.33140884	3.07E-44	5.60E-40	1	0.994
RAB13	0	0.33001813	3.61E-23	6.59E-19	0.908	0.801
TRAM1	0	0.32972918	2.93E-23	5.35E-19	0.842	0.681
HSPA5	0	0.32664878	2.27E-14	4.14E-10	0.435	0.276
UFM1	0	0.32641229	1.44E-21	2.63E-17	0.605	0.399
CD44	0	0.32571908	1.90E-27	3.46E-23	0.922	0.737
DAB2	0	0.32507763	5.46E-22	9.96E-18	0.46	0.244
SH3GLB1	0	0.32439306	3.26E-21	5.94E-17	0.676	0.526
ADM	0	0.3240606	1.12E-09	2.04E-05	0.757	0.634

AKIRIN2	0	0.32373748	5.48E-31	1.00E-26	0.378	0.128
IL11	0	0.32350485	1.15E-24	2.10E-20	0.135	0
RPF2	0	0.32275091	1.25E-27	2.28E-23	0.435	0.19
MRPL41	0	0.31695159	6.19E-21	1.13E-16	0.707	0.559
C4orf48	0	0.31603142	1.20E-26	2.19E-22	0.344	0.116
GPRC5A	0	0.31556803	1.21E-34	2.21E-30	0.344	0.084
PAIP2	0	0.31402605	1.14E-21	2.08E-17	0.747	0.577
SRSF3	0	0.31380436	6.85E-18	1.25E-13	0.765	0.628
PAPOLA	0	0.31370816	2.09E-19	3.81E-15	0.646	0.492
LINC-PINT	0	0.31138287	1.36E-23	2.49E-19	0.281	0.087
FAM210A	0	0.31135379	4.88E-29	8.91E-25	0.405	0.156
WSB1	0	0.31045304	4.54E-18	8.28E-14	0.796	0.644
NXT1	0	0.30993114	1.26E-24	2.31E-20	0.508	0.272
CISD2	0	0.30936159	8.03E-19	1.47E-14	0.537	0.353
SFT2D1	0	0.30933626	4.72E-24	8.61E-20	0.744	0.585
METAP2	0	0.30922182	1.10E-25	2.00E-21	0.838	0.684
TIMM10B	0	0.30668634	7.02E-24	1.28E-19	0.386	0.168
STRAP	0	0.30647051	1.15E-16	2.09E-12	0.561	0.412
CRLF1	0	0.30572539	2.68E-26	4.89E-22	0.281	0.074
BNIP3	0	0.30483416	4.51E-25	8.23E-21	0.995	0.985
KLHL21	0	0.30432494	4.66E-27	8.50E-23	0.321	0.101
ATP6V0B	0	0.30430237	1.27E-18	2.32E-14	0.592	0.426
GULP1	0	0.30422493	1.66E-23	3.03E-19	0.353	0.142
HDAC2	0	0.30356512	1.10E-18	2.00E-14	0.497	0.323
POLR1D	0	0.30212105	1.02E-23	1.87E-19	0.863	0.713
CTD-2095E4.5	0	0.30089801	9.56E-28	1.74E-23	0.206	0.025
LINC00493	0	0.29960286	1.17E-30	2.13E-26	0.95	0.826
SLC5A3	0	0.29941733	1.17E-07	0.00212609	0.479	0.381
TYMP	0	0.29828833	1.22E-14	2.22E-10	0.415	0.247
UBE2B	0	0.29677601	1.10E-20	2.01E-16	0.824	0.712
CCT8	0	0.29544157	9.54E-20	1.74E-15	0.755	0.614
SREK1IP1	0	0.29394126	1.06E-18	1.94E-14	0.446	0.254
CLIC4	0	0.29271958	1.50E-15	2.74E-11	0.467	0.302
USMG5	0	0.2917268	1.14E-25	2.08E-21	0.954	0.892
ZFAND2A	0	0.29168648	5.50E-32	1.00E-27	0.338	0.091
NDUFB8	0	0.29136452	1.63E-13	2.97E-09	0.764	0.677
PDS5A	0	0.28977951	1.87E-30	3.42E-26	0.342	0.101
ACTR10	0	0.28952723	9.15E-20	1.67E-15	0.488	0.292
PTBP3	0	0.28933243	6.74E-30	1.23E-25	0.357	0.112

GJA1	0	0.28882348	2.72E-23	4.97E-19	0.261	0.073
CNIH1	0	0.28827201	5.65E-18	1.03E-13	0.805	0.651
RAB1A	0	0.28811556	4.96E-17	9.05E-13	0.729	0.57
ATP6V1C1	0	0.28804365	2.23E-20	4.06E-16	0.42	0.223
MT1H	0	0.28791255	1.32E-09	2.41E-05	0.638	0.468
CCDC107	0	0.28730797	3.15E-17	5.76E-13	0.52	0.352
SYF2	0	0.28695277	1.45E-23	2.64E-19	0.943	0.825
AC090498.1	0	0.28665436	3.92E-66	7.16E-62	1	1
KCNMA1	0	0.28636838	3.62E-09	6.61E-05	0.548	0.423
ZFYVE16	0	0.28571363	1.25E-29	2.27E-25	0.324	0.09
NFKBIZ	0	0.28507403	9.31E-22	1.70E-17	0.326	0.126
VMA21	0	0.28446407	5.95E-28	1.09E-23	0.367	0.128
SNRPB2	0	0.28368279	1.25E-14	2.29E-10	0.638	0.512
CHMP5	0	0.28347077	3.85E-18	7.02E-14	0.642	0.481
OVOS2	0	0.28272805	1.85E-22	3.37E-18	0.202	0.039
UQCC2	0	0.28189066	1.20E-15	2.19E-11	0.505	0.349
MRPL51	0	0.28177863	3.05E-22	5.56E-18	0.889	0.761
ATP6V0E1	0	0.28120346	2.31E-21	4.22E-17	0.902	0.76
SNHG15	0	0.28070736	4.87E-28	8.88E-24	0.321	0.095
NUDT21	0	0.28044903	3.00E-17	5.48E-13	0.52	0.35
MRPL57	0	0.27963698	3.00E-18	5.47E-14	0.834	0.716
IMP4	0	0.27735037	2.40E-19	4.38E-15	0.504	0.305
NOP10	0	0.27649606	2.26E-19	4.11E-15	0.765	0.603
RHOD	0	0.27631969	1.67E-14	3.05E-10	0.459	0.302
COX6B1	0	0.27535218	5.48E-30	9.99E-26	0.992	0.964
LAMTOR5	0	0.27495297	3.56E-19	6.50E-15	0.845	0.701
NDUFC1	0	0.2748172	7.95E-20	1.45E-15	0.897	0.774
AKAP12	0	0.27293135	1.01E-23	1.85E-19	0.237	0.055
TAF1D	0	0.27280366	1.04E-14	1.90E-10	0.706	0.601
POLR2H	0	0.2727698	1.82E-16	3.33E-12	0.483	0.309
CCDC59	0	0.27175927	3.58E-14	6.53E-10	0.503	0.354
AIMP1	0	0.27040074	3.93E-15	7.18E-11	0.676	0.526
MOB4	0	0.26987063	2.60E-18	4.75E-14	0.363	0.175
HSP90AA1	0	0.26945164	4.72E-09	8.61E-05	0.798	0.734
LRRFIP1	0	0.26929015	2.67E-15	4.87E-11	0.427	0.255
FHL2	0	0.2692123	1.90E-28	3.47E-24	0.277	0.063
NDUFA6	0	0.26890317	1.68E-14	3.07E-10	0.553	0.406
PPP1R2	0	0.26820676	8.18E-17	1.49E-12	0.496	0.321
MRPL50	0	0.2665149	3.65E-14	6.65E-10	0.361	0.208

RNF19B	0	0.26443041	3.57E-31	6.51E-27	0.269	0.05
CDKN2B	0	0.26388238	9.26E-37	1.69E-32	0.243	0.019
SCAND1	0	0.26368356	5.65E-17	1.03E-12	0.635	0.469
EXOSC4	0	0.26270372	5.16E-21	9.42E-17	0.363	0.16
SNRPD1	0	0.26175461	4.18E-16	7.63E-12	0.622	0.468
PELI1	0	0.26126563	1.00E-20	1.83E-16	0.237	0.068
NAMPT	0	0.26055016	7.01E-15	1.28E-10	0.537	0.37
KITLG	0	0.26040615	2.35E-19	4.30E-15	0.334	0.145
CCDC174	0	0.26032134	6.41E-20	1.17E-15	0.42	0.208
ATF4	0	0.26001642	3.91E-19	7.13E-15	0.966	0.919
PLOD2	0	0.25960907	1.19E-12	2.18E-08	0.741	0.608
MAR5	0	0.25947642	7.44E-19	1.36E-14	0.451	0.252
EPB41L4A-AS1	0	0.25771212	2.12E-21	3.87E-17	0.964	0.892
SPHK1	0	0.25585333	4.85E-23	8.84E-19	0.252	0.068
C16orf13	0	0.25580005	4.88E-16	8.91E-12	0.806	0.68
MRPL54	0	0.25574471	1.08E-13	1.97E-09	0.472	0.314
DDX21	0	0.25557883	7.37E-22	1.34E-17	0.393	0.178
ANKRD10	0	0.25408884	1.31E-15	2.39E-11	0.358	0.188
CA9	0	0.25337537	1.69E-18	3.08E-14	0.268	0.097
EMC2	0	0.25321607	1.49E-11	2.72E-07	0.541	0.412
ITGA5	0	0.25075833	7.28E-26	1.33E-21	0.24	0.05
ZC3H15	0	0.2503728	5.02E-14	9.16E-10	0.585	0.428
ERRFI1	0	0.25033728	4.20E-08	0.00076636	0.305	0.201
SMDT1	0	-0.2503721	1.07E-16	1.95E-12	0.55	0.716
EIF4EBP2	0	-0.2504724	8.22E-30	1.50E-25	0.09	0.327
IGBP1	0	-0.251099	9.86E-14	1.80E-09	0.617	0.732
TRAPPC1	0	-0.2516322	3.32E-22	6.07E-18	0.229	0.466
NHP2	0	-0.2518615	4.73E-21	8.63E-17	0.296	0.535
CYHR1	0	-0.2520831	7.80E-23	1.42E-18	0.184	0.41
MRPS21	0	-0.2521365	1.26E-20	2.30E-16	0.797	0.899
RPS3	0	-0.2523708	1.90E-77	3.47E-73	1	1
ANAPC16	0	-0.2525476	2.27E-22	4.14E-18	0.822	0.927
UGP2	0	-0.2531141	2.50E-19	4.56E-15	0.416	0.652
MT-ND5	0	-0.254056	1.32E-10	2.41E-06	0.431	0.553
RPL6	0	-0.2542469	1.20E-50	2.18E-46	1	1
NDUFA3	0	-0.2553968	5.40E-19	9.85E-15	0.349	0.578
PDGFRA	0	-0.2563159	7.11E-33	1.30E-28	0.046	0.272
PGLS	0	-0.2565167	9.63E-17	1.76E-12	0.541	0.705
MAOB	0	-0.2565382	4.28E-19	7.81E-15	0.2	0.407

SOCS3	0	-0.2567865	1.18E-23	2.15E-19	0.081	0.277
TMEM98	0	-0.2578248	2.20E-27	4.02E-23	0.095	0.32
NUCB2	0	-0.2579099	4.41E-20	8.05E-16	0.187	0.396
CCDC14	0	-0.2582177	5.42E-24	9.89E-20	0.129	0.349
MIR99AHG	0	-0.258545	1.19E-36	2.17E-32	0.038	0.276
CAMLG	0	-0.2598145	8.62E-19	1.57E-14	0.729	0.85
ZFAND1	0	-0.2601801	4.46E-17	8.13E-13	0.491	0.687
FKBP10	0	-0.2604496	8.15E-24	1.49E-19	0.182	0.418
CHPT1	0	-0.260496	1.98E-17	3.62E-13	0.46	0.661
NUCKS1	0	-0.2614478	9.50E-17	1.73E-12	0.452	0.619
JUN	0	-0.2614589	3.09E-20	5.64E-16	0.109	0.298
GSTP1	0	-0.2617049	3.32E-21	6.05E-17	0.927	0.963
TMEM230	0	-0.2619507	1.24E-18	2.27E-14	0.521	0.709
FGGY	0	-0.2626061	2.18E-32	3.97E-28	0.084	0.331
SLC44A1	0	-0.2628096	3.10E-22	5.66E-18	0.155	0.367
RP11-553L6.5	0	-0.2638021	1.93E-30	3.52E-26	0.09	0.33
GPAA1	0	-0.2644193	6.58E-27	1.20E-22	0.142	0.381
FHL1	0	-0.2646605	1.88E-22	3.44E-18	0.237	0.473
LRPAP1	0	-0.2659665	3.19E-17	5.82E-13	0.289	0.481
CSDE1	0	-0.267804	2.22E-18	4.06E-14	0.651	0.817
TUBB	0	-0.2681127	3.78E-20	6.89E-16	0.775	0.89
DPP7	0	-0.2681468	1.55E-19	2.83E-15	0.467	0.683
GADD45A	0	-0.2684592	1.21E-09	2.20E-05	0.407	0.545
RPS29	0	-0.268615	1.81E-69	3.31E-65	1	1
CD302	0	-0.2687952	1.27E-33	2.31E-29	0.06	0.298
ASPN	0	-0.2704944	8.17E-20	1.49E-15	0.023	0.157
ATP5A1	0	-0.2716041	5.06E-17	9.24E-13	0.398	0.582
H1F0	0	-0.2722047	6.68E-11	1.22E-06	0.21	0.352
FAM200B	0	-0.2728805	1.46E-25	2.66E-21	0.18	0.423
SCARA3	0	-0.273455	1.05E-35	1.91E-31	0.061	0.312
SSR4	0	-0.2740518	1.44E-23	2.63E-19	0.908	0.96
MTDH	0	-0.2741433	7.78E-17	1.42E-12	0.492	0.666
FIS1	0	-0.2745116	8.45E-20	1.54E-15	0.496	0.702
CCDC88A	0	-0.2745932	2.22E-29	4.05E-25	0.089	0.32
TMED10	0	-0.2755063	1.84E-20	3.36E-16	0.747	0.88
RARRES3	0	-0.2758151	8.79E-44	1.60E-39	0.017	0.268
CALM3	0	-0.2765489	2.85E-19	5.21E-15	0.488	0.691
BEX2	0	-0.2770106	9.18E-23	1.67E-18	0.203	0.437
LAMTOR1	0	-0.2795162	1.57E-21	2.86E-17	0.411	0.63

SMIM19	0	-0.2800329	1.86E-21	3.39E-17	0.269	0.492
TRPS1	0	-0.2808965	7.50E-20	1.37E-15	0.224	0.433
COL6A2	0	-0.2816762	9.84E-16	1.80E-11	0.344	0.521
HINT1	0	-0.282762	5.61E-35	1.02E-30	0.987	1
RPS9	0	-0.2829346	1.25E-82	2.28E-78	1	1
ARL2BP	0	-0.2832822	5.70E-18	1.04E-13	0.34	0.531
KDELR2	0	-0.2835349	1.07E-24	1.95E-20	0.711	0.897
N4BP2L2	0	-0.2844201	9.07E-22	1.66E-17	0.597	0.79
C19orf43	0	-0.2844893	2.80E-26	5.11E-22	0.763	0.901
GRN	0	-0.2853979	3.29E-37	6.01E-33	0.068	0.33
RPS4X	0	-0.2854146	2.42E-87	4.41E-83	1	1
PLPP1	0	-0.2855785	5.26E-28	9.59E-24	0.068	0.279
SRSF5	0	-0.2856383	3.43E-21	6.25E-17	0.444	0.669
RPL15	0	-0.286385	9.66E-106	1.76E-101	1	1
BHLHE41	0	-0.2868331	4.62E-28	8.43E-24	0.118	0.357
HSPD1	0	-0.288129	3.10E-19	5.66E-15	0.499	0.679
AHNAK2	0	-0.289075	9.65E-25	1.76E-20	0.223	0.473
DBP	0	-0.290668	1.31E-32	2.40E-28	0.094	0.345
COL9A2	0	-0.2913247	8.77E-29	1.60E-24	0.092	0.323
RPL13	0	-0.291582	2.88E-107	5.25E-103	1	1
SNRPA	0	-0.2916973	2.42E-31	4.41E-27	0.123	0.379
RPL8	0	-0.2917324	1.73E-93	3.16E-89	1	1
RPSA	0	-0.2918012	1.44E-48	2.62E-44	1	1
COMMD6	0	-0.2929122	1.90E-48	3.46E-44	0.991	1
RPS17	0	-0.2940428	3.96E-70	7.22E-66	1	1
TMEM59	0	-0.2941527	6.97E-28	1.27E-23	0.955	0.99
PAM	0	-0.2954267	6.23E-23	1.14E-18	0.279	0.519
CMPK1	0	-0.2955614	1.02E-23	1.86E-19	0.607	0.818
DYNLT1	0	-0.2961858	1.61E-27	2.95E-23	0.239	0.501
TNFRSF1A	0	-0.2965732	1.89E-23	3.45E-19	0.22	0.446
CD320	0	-0.297344	6.04E-24	1.10E-19	0.186	0.412
RPL7A	0	-0.2974366	4.48E-81	8.17E-77	1	1
NUPR1	0	-0.2982075	4.49E-33	8.19E-29	0.969	0.992
IFI27L2	0	-0.2985301	5.01E-23	9.15E-19	0.666	0.844
HMGN3	0	-0.2988278	2.86E-31	5.23E-27	0.145	0.414
ETFB	0	-0.2989025	4.92E-24	8.98E-20	0.268	0.499
SERPING1	0	-0.2994374	5.47E-18	9.99E-14	0.418	0.612
CHMP3	0	-0.2996703	1.05E-21	1.92E-17	0.358	0.578
RPS5	0	-0.3001259	1.03E-83	1.87E-79	1	1

MYLK	0	-0.3016808	1.79E-33	3.27E-29	0.058	0.292
PNISR	0	-0.3018993	2.06E-21	3.76E-17	0.521	0.701
IGFBP6	0	-0.3019767	2.00E-18	3.65E-14	0.302	0.517
GSTA4	0	-0.3023832	8.08E-34	1.47E-29	0.13	0.401
ERGIC3	0	-0.302402	2.65E-24	4.84E-20	0.623	0.837
EIF3J-AS1	0	-0.3037752	4.38E-38	7.99E-34	0.105	0.389
RPL13A	0	-0.3039261	8.27E-111	1.51E-106	1	1
RPS4Y1	0	-0.3041947	7.67E-47	1.40E-42	0.989	1
CANX	0	-0.3070134	2.70E-24	4.92E-20	0.521	0.742
PPIC	0	-0.3076169	3.88E-27	7.07E-23	0.149	0.383
PLA2G16	0	-0.3087883	8.15E-28	1.49E-23	0.191	0.448
RPL10	0	-0.3099167	5.48E-109	1.00E-104	1	1
AC005152.3	0	-0.3107264	9.58E-43	1.75E-38	0.029	0.287
MKX	0	-0.3109774	4.02E-33	7.34E-29	0.065	0.301
CMB9-22P13.1	0	-0.3118538	5.20E-23	9.48E-19	0.264	0.491
RPS6	0	-0.3120995	6.74E-109	1.23E-104	1	1
ARL6IP5	0	-0.3122103	1.56E-24	2.84E-20	0.375	0.617
DUT	0	-0.3122188	9.87E-27	1.80E-22	0.398	0.657
CTSF	0	-0.3130713	4.60E-40	8.38E-36	0.073	0.352
RPS7	0	-0.3134477	4.39E-83	8.01E-79	1	1
ITM2B	0	-0.3139721	1.37E-33	2.51E-29	0.952	0.988
RNF130	0	-0.3160739	8.77E-25	1.60E-20	0.386	0.621
LRP10	0	-0.3161907	2.28E-25	4.15E-21	0.218	0.455
SEP1	0	-0.3173562	8.93E-41	1.63E-36	0.097	0.394
C10orf10	0	-0.3185345	1.18E-10	2.14E-06	0.444	0.583
MORF4L2	0	-0.3207261	1.11E-23	2.03E-19	0.387	0.617
RPS13	0	-0.3232376	2.23E-90	4.07E-86	1	1
C1S	0	-0.3243585	4.67E-27	8.53E-23	0.408	0.672
ABHD14B	0	-0.3247953	3.95E-34	7.21E-30	0.206	0.501
OS9	0	-0.3252073	9.00E-28	1.64E-23	0.279	0.534
UMAD1	0	-0.3266271	2.63E-31	4.81E-27	0.174	0.44
TMED2	0	-0.3267604	3.25E-29	5.93E-25	0.605	0.844
RPL5	0	-0.3277681	1.07E-80	1.96E-76	1	1
GLTSCR2	0	-0.3277708	1.53E-34	2.79E-30	0.897	0.971
OXA1L	0	-0.3278796	4.25E-24	7.76E-20	0.459	0.668
TPD52L1	0	-0.3283987	2.81E-26	5.13E-22	0.239	0.484
H1FX	0	-0.3284316	1.58E-36	2.88E-32	0.069	0.327
ATP5D	0	-0.3286449	6.59E-34	1.20E-29	0.817	0.941
RPS23	0	-0.3290307	7.28E-107	1.33E-102	1	1

TCEA2	0	-0.3304407	1.01E-34	1.84E-30	0.186	0.472
SARAF	0	-0.3314053	3.36E-26	6.14E-22	0.495	0.714
ARPC1A	0	-0.3315954	8.94E-31	1.63E-26	0.282	0.566
SPINT2	0	-0.331656	8.81E-34	1.61E-29	0.142	0.418
VAMP5	0	-0.3318283	3.61E-30	6.59E-26	0.225	0.491
HEXB	0	-0.3319508	9.85E-26	1.80E-21	0.253	0.487
SUMF2	0	-0.3321411	1.48E-31	2.69E-27	0.207	0.483
PRCP	0	-0.3367916	1.25E-43	2.28E-39	0.064	0.35
HSPB1	0	-0.33691	2.81E-25	5.12E-21	0.54	0.749
SLC25A3	0	-0.337895	7.14E-36	1.30E-31	0.873	0.972
CRIP2	0	-0.3407812	4.61E-32	8.42E-28	0.216	0.492
HADHA	0	-0.3427821	4.72E-33	8.61E-29	0.24	0.523
MYL6	0	-0.3430422	8.94E-38	1.63E-33	0.857	0.957
AC003090.1	0	-0.3445343	3.37E-55	6.15E-51	0.023	0.331
PTPRD-AS1	0	-0.3462457	1.96E-30	3.58E-26	0.211	0.474
RPL30	0	-0.3467772	6.17E-93	1.13E-88	1	1
TCEA3	0	-0.347269	5.30E-36	9.68E-32	0.151	0.434
LTA4H	0	-0.3479562	9.11E-28	1.66E-23	0.411	0.655
ANGPTL2	0	-0.3488194	1.44E-27	2.62E-23	0.206	0.455
MFGE8	0	-0.349763	3.52E-24	6.42E-20	0.699	0.873
HLA-B	0	-0.3512859	9.95E-27	1.82E-22	0.595	0.826
HSP90AB1	0	-0.3515489	1.55E-42	2.83E-38	0.968	0.994
RPS27A	0	-0.3517893	3.46E-119	6.31E-115	1	1
RPLP2	0	-0.3524268	9.41E-115	1.72E-110	1	1
GREM1	0	-0.3527254	4.04E-39	7.37E-35	0.001	0.208
ENAH	0	-0.3532082	3.75E-46	6.85E-42	0.07	0.375
SDC2	0	-0.3533916	8.69E-37	1.59E-32	0.577	0.848
PABPC4	0	-0.3539961	3.83E-28	6.98E-24	0.422	0.658
ILF3-AS1	0	-0.3539987	1.30E-36	2.38E-32	0.153	0.441
DNM3OS	0	-0.3545347	5.03E-42	9.18E-38	0.068	0.349
POLR2L	0	-0.3549956	8.18E-33	1.49E-28	0.688	0.865
ARL6IP4	0	-0.3551216	1.34E-40	2.44E-36	0.78	0.938
CHRDL2	0	-0.3551776	1.80E-34	3.29E-30	0.053	0.288
BEX4	0	-0.355184	4.18E-34	7.64E-30	0.314	0.626
VKORC1	0	-0.3556469	3.08E-38	5.62E-34	0.847	0.953
MIA	0	-0.3574351	5.31E-24	9.68E-20	0.915	0.953
ZBTB20	0	-0.3575686	1.70E-42	3.10E-38	0.119	0.428
TPM2	0	-0.3600912	1.59E-30	2.91E-26	0.269	0.546
SLC29A1	0	-0.361092	6.06E-37	1.10E-32	0.202	0.508

LDHB	0	-0.3614173	6.32E-34	1.15E-29	0.671	0.868
TOMM7	0	-0.361877	4.60E-89	8.39E-85	1	1
LITAF	0	-0.3627075	5.52E-32	1.01E-27	0.281	0.56
VCAN	0	-0.3627874	2.22E-39	4.05E-35	0.093	0.381
PRDX5	0	-0.3629582	4.03E-39	7.35E-35	0.779	0.938
CPE	0	-0.3637168	7.07E-28	1.29E-23	0.565	0.779
DMKN	0	-0.364127	1.05E-36	1.91E-32	0.215	0.519
RABAC1	0	-0.364147	7.89E-34	1.44E-29	0.655	0.876
NPM1	0	-0.3641623	1.52E-59	2.76E-55	1	1
RBMX	0	-0.3647455	2.46E-31	4.49E-27	0.592	0.808
NMRK1	0	-0.3647622	1.02E-34	1.86E-30	0.196	0.483
C1orf21	0	-0.3658147	2.15E-35	3.91E-31	0.292	0.6
PPIA	0	-0.3664499	5.80E-47	1.06E-42	0.926	0.982
ATRAID	0	-0.3665605	5.80E-35	1.06E-30	0.601	0.844
PPT1	0	-0.3670277	3.46E-42	6.31E-38	0.125	0.432
RPL14	0	-0.3673208	1.06E-118	1.93E-114	1	1
IER2	0	-0.3684792	2.94E-29	5.36E-25	0.184	0.441
PDE4DIP	0	-0.3690684	5.67E-36	1.03E-31	0.204	0.499
ENPP1	0	-0.3693437	4.69E-51	8.55E-47	0.048	0.356
PNRC1	0	-0.3704567	6.94E-49	1.27E-44	0.923	0.996
PIK3R1	0	-0.3708355	4.85E-33	8.84E-29	0.046	0.269
RPL23	0	-0.3709534	2.13E-77	3.88E-73	0.999	1
TMEM219	0	-0.3713081	1.16E-34	2.12E-30	0.519	0.759
SH3BGRL	0	-0.3724593	2.49E-34	4.54E-30	0.298	0.583
RPS2	0	-0.3740862	1.64E-126	3.00E-122	1	1
RPL19	0	-0.3741764	6.44E-138	1.17E-133	1	1
PLD3	0	-0.3754103	1.86E-37	3.40E-33	0.237	0.548
RPL3	0	-0.3755027	1.78E-109	3.25E-105	1	1
BSG	0	-0.3759079	9.75E-35	1.78E-30	0.428	0.71
HDLBP	0	-0.3769551	3.33E-32	6.07E-28	0.267	0.539
EBPL	0	-0.3777183	5.37E-44	9.79E-40	0.18	0.509
PPP1R3C	0	-0.3783513	6.32E-24	1.15E-19	0.221	0.443
FAM46A	0	-0.3785435	4.16E-19	7.59E-15	0.326	0.516
ST13	0	-0.3788697	4.43E-51	8.07E-47	0.943	0.986
LRRC2	0	-0.3793359	9.70E-45	1.77E-40	0.085	0.386
SSPN	0	-0.3807055	7.06E-54	1.29E-49	0.049	0.374
RPL9	0	-0.3814822	1.51E-120	2.75E-116	1	1
C7orf73	0	-0.3821885	1.09E-36	2.00E-32	0.459	0.77
LINC01503	0	-0.3905378	8.06E-30	1.47E-25	0.16	0.418

NDUFA11	0	-0.3916614	1.33E-42	2.42E-38	0.772	0.938
RPL18	0	-0.3973797	8.85E-120	1.61E-115	1	1
CALR	0	-0.4000368	1.39E-31	2.54E-27	0.391	0.648
BTF3	0	-0.4005725	1.24E-77	2.26E-73	0.996	0.999
SERPINA5	0	-0.4010882	3.77E-38	6.88E-34	0.186	0.48
PDIA3	0	-0.4027949	2.13E-36	3.89E-32	0.284	0.572
EIF3E	0	-0.4049702	1.53E-62	2.79E-58	0.997	1
RP11-983P16.4	0	-0.4061522	3.67E-51	6.70E-47	0.131	0.486
AHNAK	0	-0.4071046	4.03E-41	7.36E-37	0.214	0.532
HIST1H1C	0	-0.407794	8.34E-19	1.52E-14	0.275	0.481
BAMBI	0	-0.4083637	8.50E-21	1.55E-16	0.375	0.579
CYBRD1	0	-0.4103259	1.33E-39	2.43E-35	0.389	0.691
CTSD	0	-0.4111631	2.03E-46	3.71E-42	0.721	0.931
TCEAL8	0	-0.4140478	2.05E-38	3.74E-34	0.497	0.752
NUCB1	0	-0.4148058	3.63E-38	6.62E-34	0.345	0.639
THY1	0	-0.4149807	1.39E-42	2.54E-38	0.076	0.36
CUTA	0	-0.4157201	7.87E-44	1.44E-39	0.639	0.891
AP2M1	0	-0.4167485	1.27E-36	2.31E-32	0.344	0.617
SSR2	0	-0.4168002	7.87E-48	1.44E-43	0.739	0.917
CREG1	0	-0.4175269	8.04E-41	1.47E-36	0.374	0.677
FGF7	0	-0.4179972	9.52E-38	1.74E-33	0.09	0.374
EIF3D	0	-0.4183049	3.37E-43	6.15E-39	0.67	0.863
PRDX4	0	-0.4186315	1.13E-39	2.06E-35	0.443	0.749
RPS16	0	-0.4190397	3.77E-136	6.88E-132	1	1
S100A4	0	-0.4206886	2.10E-21	3.83E-17	0.533	0.731
RPL34	0	-0.4283634	2.57E-144	4.69E-140	1	1
TSPAN4	0	-0.4288239	1.16E-44	2.12E-40	0.186	0.516
EFNA1	0	-0.4299254	3.23E-46	5.90E-42	0.158	0.495
CXXC5	0	-0.4355457	1.57E-59	2.87E-55	0.061	0.417
FCGRT	0	-0.4370522	1.50E-41	2.74E-37	0.286	0.599
FTL	0	-0.4398594	4.41E-91	8.06E-87	1	1
SEPP1	0	-0.4400003	6.69E-44	1.22E-39	0.17	0.498
CHID1	0	-0.4406125	4.46E-48	8.13E-44	0.236	0.579
GNAS	0	-0.4423683	6.84E-43	1.25E-38	0.354	0.662
PIM3	0	-0.4437918	9.37E-33	1.71E-28	0.328	0.597
CTGF	0	-0.4439868	3.63E-26	6.62E-22	0.163	0.4
RPL23A	0	-0.4453627	1.80E-153	3.28E-149	1	1
IFITM2	0	-0.4455085	2.46E-47	4.48E-43	0.775	0.942
EEF1B2	0	-0.4457941	1.93E-73	3.52E-69	0.985	1

ATRNL1	0	-0.4484618	3.63E-60	6.63E-56	0.048	0.396
EIF2S3	0	-0.4521457	4.90E-40	8.94E-36	0.423	0.723
TTC3	0	-0.454512	1.71E-44	3.12E-40	0.401	0.724
ITM2A	0	-0.457438	1.92E-45	3.50E-41	0.204	0.532
RPS3A	0	-0.458207	1.11E-155	2.02E-151	1	1
EIF2A	0	-0.4583025	8.33E-47	1.52E-42	0.603	0.84
MT-ND4	0	-0.4597139	1.32E-21	2.41E-17	0.928	0.892
S100A1	0	-0.4603073	2.50E-68	4.57E-64	0.999	1
HLA-A	0	-0.4621974	7.90E-50	1.44E-45	0.764	0.949
TSPO	0	-0.4630243	5.47E-50	9.98E-46	0.607	0.883
ATPIF1	0	-0.4659293	2.92E-49	5.32E-45	0.402	0.732
SSR3	0	-0.4680195	2.21E-52	4.04E-48	0.806	0.949
CCDC102B	0	-0.4726868	7.74E-76	1.41E-71	0.011	0.394
CCNG1	0	-0.4746581	8.48E-53	1.55E-48	0.233	0.597
GOLIM4	0	-0.4747287	4.37E-38	7.97E-34	0.167	0.459
SEC11A	0	-0.4761774	3.41E-68	6.22E-64	0.797	0.977
MDFI	0	-0.4766445	7.90E-55	1.44E-50	0.15	0.512
PTRF	0	-0.4884989	1.87E-54	3.40E-50	0.162	0.523
HIST1H4C	0	-0.4899506	8.53E-42	1.56E-37	0.281	0.593
SDF4	0	-0.4908179	1.37E-51	2.51E-47	0.385	0.705
NAP1L1	0	-0.4911779	1.04E-78	1.90E-74	0.947	0.993
S100A13	0	-0.4936164	2.16E-86	3.94E-82	0.987	1
ARHGAP29	0	-0.4948037	1.50E-51	2.73E-47	0.149	0.506
NFIA	0	-0.4990751	3.69E-75	6.73E-71	0.069	0.494
PLXDC2	0	-0.5039815	1.06E-51	1.94E-47	0.227	0.588
PCBP2	0	-0.5110359	8.63E-72	1.58E-67	0.805	0.971
PSAP	0	-0.5142557	1.80E-63	3.29E-59	0.743	0.95
HLA-C	0	-0.5143687	8.59E-53	1.57E-48	0.753	0.932
CYR61	0	-0.5175429	4.76E-43	8.69E-39	0.084	0.374
NOVA1	0	-0.5190402	2.69E-33	4.91E-29	0.794	0.921
XAGE2	0	-0.5191038	2.58E-33	4.70E-29	0.009	0.199
YBX1	0	-0.5211905	1.05E-95	1.92E-91	0.962	0.999
RPLP0	0	-0.5264855	5.20E-150	9.48E-146	1	1
IGFBP7	0	-0.5265603	3.53E-38	6.44E-34	0.599	0.846
RPL7	0	-0.5342597	4.67E-140	8.52E-136	1	1
GSN	0	-0.5382119	2.06E-51	3.75E-47	0.235	0.586
DDR2	0	-0.5421004	1.87E-62	3.42E-58	0.184	0.585
BGN	0	-0.5460818	6.84E-76	1.25E-71	0.967	1
SPON2	0	-0.5462076	2.83E-71	5.16E-67	0.036	0.421

PDIA6	0	-0.5464786	2.34E-65	4.27E-61	0.3	0.699
TPT1	0	-0.5475027	6.16E-153	1.12E-148	1	1
BRK1	0	-0.5506386	5.53E-72	1.01E-67	0.609	0.903
FTH1	0	-0.5520779	1.76E-98	3.22E-94	1	1
HMGN2	0	-0.5527749	5.57E-78	1.02E-73	0.779	0.966
NPC2	0	-0.5531937	3.42E-95	6.24E-91	0.955	0.997
COL11A1	0	-0.5602096	5.32E-67	9.70E-63	0.279	0.708
CRISPLD1	0	-0.5602706	1.74E-65	3.18E-61	0.007	0.342
CLU	0	-0.5627176	1.76E-42	3.21E-38	0.996	1
PCOLCE2	0	-0.5644972	4.39E-63	8.01E-59	0.72	0.942
SOD3	0	-0.56579	9.66E-45	1.76E-40	0.867	0.971
H3F3A	0	-0.5687586	3.88E-92	7.08E-88	0.899	0.994
ITM2C	0	-0.5695851	4.07E-64	7.43E-60	0.145	0.537
DUSP1	0	-0.5714515	5.79E-54	1.06E-49	0.267	0.65
PRUNE2	0	-0.5762622	3.10E-70	5.66E-66	0.141	0.57
SLC14A1	0	-0.5771407	8.13E-72	1.48E-67	0.102	0.524
SNAI2	0	-0.5991182	8.82E-48	1.61E-43	0.221	0.553
EIF4B	0	-0.6002239	5.94E-85	1.08E-80	0.712	0.949
CFH	0	-0.6028101	7.96E-33	1.45E-28	0.131	0.385
INHBA	0	-0.6028153	6.99E-69	1.27E-64	0.088	0.501
PEBP1	0	-0.603178	1.98E-80	3.62E-76	0.593	0.903
RPL10A	0	-0.6114019	4.58E-162	8.36E-158	1	1
CD9	0	-0.6132973	2.52E-66	4.60E-62	0.245	0.655
ISLR	0	-0.6138744	6.65E-88	1.21E-83	0.045	0.499
EIF3L	0	-0.6150135	8.11E-91	1.48E-86	0.719	0.967
SLC40A1	0	-0.6237985	5.23E-84	9.54E-80	0.012	0.429
FBXO2	0	-0.6327672	4.04E-87	7.38E-83	0.418	0.846
TCF4	0	-0.6332041	1.82E-74	3.32E-70	0.11	0.539
VIM	0	-0.6352794	2.31E-111	4.22E-107	1	1
RPS8	0	-0.640163	1.71E-193	3.12E-189	1	1
GAS6	0	-0.6414544	2.70E-86	4.92E-82	0.07	0.531
RPL4	0	-0.6500806	7.89E-152	1.44E-147	1	1
ACTG1	0	-0.6539366	8.51E-91	1.55E-86	0.955	0.996
EID1	0	-0.65598	7.19E-105	1.31E-100	0.744	0.979
CTSC	0	-0.6618985	2.88E-52	5.26E-48	0.077	0.404
RPS11	0	-0.6633326	1.91E-158	3.48E-154	0.995	1
NNMT	0	-0.6640032	2.69E-71	4.90E-67	0.782	0.954
CRTAP	0	-0.6720623	4.14E-94	7.55E-90	0.428	0.843
ID4	0	-0.6734361	1.59E-68	2.90E-64	0.288	0.705

HNRNPA1	0	-0.683615	4.96E-125	9.04E-121	0.983	1
PTMS	0	-0.688513	1.02E-95	1.87E-91	0.332	0.81
PABPC1	0	-0.6918922	7.83E-109	1.43E-104	0.755	0.972
FOS	0	-0.6969234	1.34E-83	2.45E-79	0.297	0.767
RPS20	0	-0.6978453	1.46E-179	2.66E-175	0.997	1
CITED2	0	-0.7145248	3.71E-72	6.77E-68	0.147	0.579
EIF3H	0	-0.7149858	1.10E-140	2.01E-136	0.956	0.999
HTRA1	0	-0.7153292	1.13E-90	2.06E-86	0.418	0.866
SPARC	0	-0.7191531	3.98E-107	7.26E-103	0.125	0.673
CCNI	0	-0.7354868	1.17E-156	2.13E-152	0.988	1
S100B	0	-0.7356834	6.96E-94	1.27E-89	0.436	0.873
FMOD	0	-0.7358102	1.50E-105	2.74E-101	0.627	0.95
FXYD6	0	-0.7470278	4.28E-101	7.80E-97	0.497	0.902
ID2	0	-0.7489817	2.73E-69	4.98E-65	0.306	0.71
MALAT1	0	-0.7516333	1.14E-92	2.09E-88	1	1
PRELP	0	-0.753914	6.72E-93	1.23E-88	0.771	0.978
MT-ND1	0	-0.7703336	1.59E-78	2.90E-74	0.272	0.71
MT-CO1	0	-0.7721942	2.68E-44	4.89E-40	0.914	0.913
P4HB	0	-0.7894883	1.16E-106	2.12E-102	0.495	0.921
MT-CO3	0	-0.7895884	4.37E-51	7.98E-47	0.805	0.866
CCDC80	0	-0.794751	2.95E-82	5.38E-78	0.706	0.96
CEBPD	0	-0.7977284	3.80E-80	6.93E-76	0.664	0.93
C2orf40	0	-0.8006764	3.44E-88	6.27E-84	0.93	0.999
YBX3	0	-0.8094213	1.01E-121	1.85E-117	0.621	0.967
RBP4	0	-0.8211461	2.59E-100	4.73E-96	0.312	0.797
EEF2	0	-0.8226749	5.16E-130	9.42E-126	0.979	0.999
JUNB	0	-0.8299082	8.11E-93	1.48E-88	0.334	0.812
NGFRAP1	0	-0.8303483	3.21E-128	5.85E-124	0.492	0.953
APOD	0	-0.8312576	5.88E-64	1.07E-59	0.141	0.535
EEF1A1	0	-0.8504049	1.11E-219	2.02E-215	1	1
B2M	0	-0.8567177	1.40E-152	2.55E-148	0.958	1
CST3	0	-0.8635364	8.68E-132	1.58E-127	0.954	1
TSC22D1	0	-0.8640457	1.96E-82	3.57E-78	0.398	0.823
COL9A3	0	-0.8795755	1.61E-88	2.94E-84	0.22	0.703
CD99	0	-0.8798483	1.14E-151	2.08E-147	0.711	0.99
MT-ND3	0	-0.8943939	4.00E-81	7.30E-77	0.314	0.752
COL2A1	0	-0.9027009	6.20E-134	1.13E-129	0.194	0.811
CP	0	-0.917685	3.08E-116	5.61E-112	0.357	0.861
CHI3L1	0	-0.9219301	1.69E-74	3.08E-70	0.886	0.992

DCN	0	-0.9272984	6.48E-165	1.18E-160	0.999	1
MT-ND2	0	-0.9383228	7.42E-82	1.35E-77	0.31	0.739
LECT1	0	-0.960562	6.45E-109	1.18E-104	0.334	0.857
RHOBTB3	0	-0.9659423	4.89E-133	8.91E-129	0.249	0.823
MT-CYB	0	-0.9762734	3.16E-86	5.77E-82	0.361	0.782
MT-ATP6	0	-0.9770279	3.68E-76	6.71E-72	0.562	0.843
MT-CO2	0	-0.9987681	4.11E-67	7.51E-63	0.85	0.919
ZFP36L2	0	-1.0329534	1.46E-101	2.67E-97	0.141	0.661
PAX1	0	-1.035802	5.60E-151	1.02E-146	0.296	0.879
NEAT1	0	-1.0670119	4.71E-157	8.60E-153	0.984	0.999
ID1	0	-1.090274	8.17E-103	1.49E-98	0.231	0.77
ADIRF	0	-1.122142	3.16E-121	5.77E-117	0.281	0.826
GPX3	0	-1.1439251	1.04E-160	1.90E-156	0.769	0.996
C2orf82	0	-1.1678843	3.05E-137	5.57E-133	0.919	1
ACAN	0	-1.1787417	7.73E-168	1.41E-163	0.71	0.994
PLAC9	0	-1.2177514	1.35E-173	2.46E-169	0.34	0.952
MGP	0	-1.2419585	1.43E-131	2.60E-127	0.997	1
CAPS	0	-1.2630857	9.57E-151	1.75E-146	0.371	0.941
SLPI	0	-1.2711762	7.93E-139	1.45E-134	0.735	0.993
ID3	0	-1.3412884	2.55E-144	4.65E-140	0.143	0.792
SPTSSB	0	-1.5913478	3.27E-159	5.97E-155	0.02	0.698
CLEC3A	0	-1.6602139	4.04E-205	7.38E-201	0.066	0.866
OGN	0	-1.7488939	5.94E-178	1.08E-173	0.537	0.977
FRZB	0	-1.8932571	4.54E-149	8.28E-145	0.253	0.897
**gene**	**cluster**	**avg_logFC**	**p_val**	**p_val_adj**	**pct.1**	**pct.2**
SPP1	1	2.93989577	9.32E-166	1.70E-161	0.891	0.104
PRG4	1	2.42779019	1.80E-124	3.28E-120	0.651	0.033
MT2A	1	2.01806853	7.98E-142	1.46E-137	1	0.99
SOD2	1	1.66428839	1.28E-137	2.34E-133	1	0.811
COMP	1	1.55726827	6.31E-108	1.15E-103	0.979	0.763
S100A2	1	1.45585353	4.64E-88	8.47E-84	0.723	0.21
IGFBP3	1	1.45408205	5.38E-112	9.82E-108	0.605	0.03
UPP1	1	1.41491547	1.21E-156	2.21E-152	0.804	0.082
PTGS2	1	1.40580688	1.29E-144	2.35E-140	0.721	0.033
VEGFA	1	1.22869872	9.56E-117	1.74E-112	0.866	0.343
CTSL	1	1.18473345	2.64E-85	4.82E-81	0.795	0.332
DDIT4L	1	1.15222151	1.60E-94	2.92E-90	0.909	0.523
FN1	1	1.13708404	1.06E-123	1.94E-119	1	0.992
HMGA1	1	1.12767504	1.79E-94	3.26E-90	0.725	0.207

TM4SF1	1	1.09938032	2.33E-75	4.25E-71	0.748	0.297
CD55	1	1.08105414	2.21E-106	4.04E-102	0.926	0.503
MMP3	1	1.0336714	5.60E-44	1.02E-39	0.895	0.643
MT1X	1	1.01908047	3.80E-46	6.94E-42	0.996	0.99
TIMP3	1	1.00496349	5.58E-54	1.02E-49	0.766	0.422
STK17A	1	0.99735264	2.56E-98	4.67E-94	0.647	0.104
TIMP1	1	0.99223098	5.99E-34	1.09E-29	1	0.999
BZW1	1	0.99065477	2.05E-101	3.73E-97	0.857	0.383
C11orf96	1	0.97762761	8.75E-92	1.60E-87	0.533	0.035
NRP2	1	0.95333764	2.33E-88	4.26E-84	0.597	0.094
ERO1A	1	0.91990776	1.12E-88	2.05E-84	0.812	0.365
TNFRSF12A	1	0.90821976	1.73E-91	3.15E-87	0.628	0.105
WTAP	1	0.90556872	1.01E-91	1.85E-87	0.891	0.447
BHLHE40	1	0.90424984	1.58E-69	2.89E-65	0.754	0.382
SMOX	1	0.89147039	1.29E-64	2.35E-60	0.601	0.184
MRPS6	1	0.87842841	5.85E-48	1.07E-43	0.961	0.905
SLC3A2	1	0.87469511	5.47E-72	9.98E-68	0.667	0.243
MIF	1	0.8181842	1.77E-134	3.24E-130	1	1
SLC2A1	1	0.81816329	2.15E-82	3.92E-78	0.595	0.111
PMP22	1	0.81777986	7.91E-84	1.44E-79	0.934	0.589
ORM1	1	0.78746165	3.58E-28	6.54E-24	0.368	0.116
CHI3L2	1	0.75711127	3.17E-32	5.79E-28	0.628	0.373
CCDC85B	1	0.74844106	3.52E-58	6.42E-54	0.895	0.674
BMP2	1	0.73077542	3.74E-69	6.82E-65	0.428	0.029
NDRG1	1	0.72766202	4.00E-65	7.29E-61	0.721	0.297
TMSB10	1	0.69979709	2.10E-58	3.84E-54	0.992	0.97
TGFBI	1	0.69897578	8.34E-47	1.52E-42	0.556	0.216
RGCC	1	0.69316489	1.30E-46	2.37E-42	0.95	0.78
AK2	1	0.69003044	8.34E-56	1.52E-51	0.828	0.577
DDIT3	1	0.68656259	3.94E-44	7.18E-40	0.659	0.354
CD44	1	0.68576558	3.38E-73	6.16E-69	0.907	0.454
ABI3BP	1	0.67883916	1.37E-29	2.50E-25	0.659	0.438
RGS2	1	0.67880715	3.51E-33	6.40E-29	0.545	0.242
VAPA	1	0.67005804	1.51E-77	2.76E-73	0.977	0.851
PPP3CA	1	0.66871992	3.12E-46	5.70E-42	0.864	0.606
PTP4A1	1	0.66698926	4.67E-55	8.52E-51	0.68	0.313
DNER	1	0.66220454	9.06E-57	1.65E-52	0.694	0.306
HLA-DRB1	1	0.65928042	8.00E-62	1.46E-57	0.541	0.131
GBE1	1	0.659023	1.48E-52	2.70E-48	0.779	0.479

ATP6V1G1	1	0.65900781	9.43E-70	1.72E-65	0.986	0.929
RNF145	1	0.65536594	3.31E-47	6.04E-43	0.57	0.231
TREM1	1	0.65164378	4.84E-72	8.83E-68	0.403	0.011
SPRY2	1	0.64289157	2.27E-66	4.14E-62	0.452	0.051
EIF1	1	0.62585092	9.12E-106	1.66E-101	1	1
SNHG12	1	0.62200067	3.53E-58	6.43E-54	0.626	0.225
PPP1R15A	1	0.62171315	2.48E-54	4.52E-50	0.527	0.147
ISG20	1	0.61564556	3.49E-49	6.38E-45	0.657	0.306
IFRD1	1	0.61559694	1.38E-51	2.51E-47	0.705	0.364
PGK1	1	0.60985295	2.38E-51	4.34E-47	0.963	0.842
NRN1	1	0.60920266	1.14E-21	2.09E-17	0.541	0.303
FOSL1	1	0.60820044	5.17E-54	9.43E-50	0.331	0.017
SLC25A37	1	0.60236051	2.44E-56	4.45E-52	0.897	0.573
RABGGTB	1	0.60009705	1.02E-60	1.85E-56	0.829	0.477
GSTO1	1	0.59745065	1.11E-57	2.02E-53	0.857	0.533
ZNF385D	1	0.59641997	4.26E-50	7.77E-46	0.764	0.433
PHLDA1	1	0.59039308	4.19E-46	7.64E-42	0.331	0.036
SGIP1	1	0.58543723	7.15E-60	1.30E-55	0.359	0.017
ENO1	1	0.58532175	3.24E-42	5.91E-38	0.99	0.976
ARPP19	1	0.57687312	5.42E-49	9.90E-45	0.715	0.418
CCND1	1	0.57420271	6.86E-51	1.25E-46	0.477	0.113
ENO2	1	0.57352623	2.10E-52	3.82E-48	0.628	0.253
RNF114	1	0.57117516	8.23E-51	1.50E-46	0.734	0.426
PPP1CB	1	0.56516792	4.68E-33	8.53E-29	0.853	0.649
GPX1	1	0.55714092	6.84E-52	1.25E-47	0.95	0.716
DNAJB9	1	0.55551346	3.33E-40	6.08E-36	0.785	0.505
BAALC	1	0.55544739	2.93E-23	5.35E-19	0.554	0.339
NBPF14	1	0.55499782	8.08E-65	1.47E-60	0.471	0.066
EPS8	1	0.54637152	5.16E-54	9.42E-50	0.508	0.127
PID1	1	0.54450236	7.17E-40	1.31E-35	0.678	0.394
FAP	1	0.53741044	8.68E-51	1.58E-46	0.459	0.101
TMEM45A	1	0.53638257	4.58E-34	8.36E-30	0.922	0.781
PPFIBP1	1	0.53077951	5.76E-33	1.05E-28	0.589	0.331
SERPINE1	1	0.52970813	4.91E-53	8.96E-49	0.341	0.024
MED21	1	0.51881854	6.20E-35	1.13E-30	0.624	0.355
EIF4EBP1	1	0.51210518	2.47E-26	4.51E-22	0.62	0.402
RND3	1	0.51186774	6.13E-40	1.12E-35	0.312	0.041
RELB	1	0.50969516	2.25E-59	4.11E-55	0.448	0.065
WDR33	1	0.50316207	1.69E-41	3.09E-37	0.649	0.333

EIF1B	1	0.50270801	8.17E-45	1.49E-40	0.828	0.567
ARF6	1	0.50270632	1.47E-41	2.67E-37	0.676	0.375
GOLT1B	1	0.50263657	2.03E-43	3.70E-39	0.597	0.268
KLF3	1	0.50119031	2.44E-29	4.46E-25	0.444	0.183
FAM162A	1	0.49859708	8.60E-40	1.57E-35	0.938	0.826
TGM2	1	0.49615903	2.27E-45	4.15E-41	0.494	0.145
CSGALNACT2	1	0.49321092	2.41E-39	4.39E-35	0.417	0.115
SLC20A1	1	0.48805025	1.30E-43	2.36E-39	0.419	0.1
PDE4B	1	0.48496246	3.22E-39	5.88E-35	0.403	0.1
RGS16	1	0.48278674	2.33E-19	4.25E-15	0.382	0.177
PLAUR	1	0.48270215	3.75E-36	6.84E-32	0.471	0.172
SERPINE2	1	0.47999904	1.67E-11	3.04E-07	0.998	0.997
ZNF593	1	0.47293145	5.49E-42	1.00E-37	0.632	0.308
SQSTM1	1	0.47168354	2.58E-39	4.72E-35	0.967	0.913
WDR45B	1	0.46827084	4.13E-37	7.54E-33	0.498	0.189
WSB1	1	0.46435481	1.04E-30	1.90E-26	0.853	0.656
XBP1	1	0.46200594	3.85E-28	7.03E-24	0.731	0.541
MT1G	1	0.46077065	4.89E-09	8.92E-05	0.93	0.855
CRLF1	1	0.46012915	5.55E-36	1.01E-31	0.453	0.149
AIMP1	1	0.45702321	1.02E-32	1.85E-28	0.698	0.44
ANXA1	1	0.45633061	9.42E-19	1.72E-14	0.822	0.715
NTMT1	1	0.45133042	1.42E-35	2.59E-31	0.533	0.234
NPR3	1	0.4434925	1.31E-55	2.38E-51	0.3	0
SH3BP5	1	0.44348483	1.02E-28	1.86E-24	0.529	0.27
TNFRSF11B	1	0.44168593	2.02E-11	3.68E-07	0.626	0.477
CLK1	1	0.43470299	1.37E-34	2.51E-30	0.7	0.434
ZFAND5	1	0.43303935	2.31E-24	4.22E-20	0.581	0.358
VMP1	1	0.43233795	9.98E-17	1.82E-12	0.651	0.513
PTGES3	1	0.43065247	2.69E-43	4.90E-39	0.983	0.925
PLCG2	1	0.42862062	5.27E-43	9.61E-39	0.328	0.041
TRMT112	1	0.4262311	4.31E-38	7.86E-34	0.946	0.842
ADSS	1	0.42469428	2.54E-26	4.64E-22	0.61	0.375
SYF2	1	0.42357899	1.58E-36	2.89E-32	0.913	0.784
SLC25A36	1	0.42343161	1.15E-30	2.10E-26	0.653	0.383
HMGA2	1	0.41957695	5.11E-62	9.33E-58	0.331	0
ARF4	1	0.41748894	1.63E-26	2.97E-22	0.806	0.614
LGALS3	1	0.41582511	2.75E-27	5.02E-23	0.965	0.935
GRB10	1	0.41375601	5.29E-48	9.65E-44	0.287	0.01
BTG3	1	0.41141043	4.37E-32	7.98E-28	0.479	0.192

GDF15	1	0.40711355	4.08E-29	7.45E-25	0.192	0.011
SAR1A	1	0.40594003	3.22E-28	5.88E-24	0.645	0.416
EMP3	1	0.40570168	8.21E-29	1.50E-24	0.93	0.855
CHD2	1	0.4047927	7.54E-21	1.38E-16	0.413	0.199
CTTN	1	0.40474374	1.19E-22	2.17E-18	0.517	0.293
EIF5	1	0.40174748	1.27E-26	2.31E-22	0.727	0.52
TMEM38B	1	0.40081586	6.43E-31	1.17E-26	0.609	0.326
COX17	1	0.39781241	9.40E-26	1.71E-21	0.804	0.624
BTG1	1	0.39580863	1.26E-22	2.30E-18	0.965	0.909
PLA2G2A	1	0.39574879	1.60E-08	0.00029204	0.388	0.245
CCT8	1	0.39543363	2.53E-29	4.62E-25	0.756	0.528
C11orf31	1	0.39529896	4.45E-30	8.12E-26	0.835	0.622
CTNNB1	1	0.39435031	7.22E-15	1.32E-10	0.442	0.259
NOL7	1	0.39380603	1.15E-26	2.10E-22	0.719	0.51
SLC5A3	1	0.39100091	9.99E-12	1.82E-07	0.525	0.379
RHOC	1	0.39057105	5.80E-23	1.06E-18	0.872	0.744
COX7A2	1	0.38923521	1.03E-46	1.88E-42	0.988	0.961
AEBP1	1	0.3878492	2.71E-19	4.95E-15	0.628	0.415
CTHRC1	1	0.38769095	1.54E-31	2.81E-27	0.298	0.058
PFDN2	1	0.38355817	3.54E-30	6.45E-26	0.7	0.477
GPRC5A	1	0.38208606	3.56E-37	6.49E-33	0.37	0.084
DNAJB1	1	0.37981317	3.71E-15	6.77E-11	0.333	0.16
ESD	1	0.37764241	3.92E-15	7.15E-11	0.829	0.732
EIF4A2	1	0.37727844	8.04E-44	1.47E-39	1	0.996
ADM	1	0.37635483	4.48E-09	8.18E-05	0.783	0.685
VIMP	1	0.3742625	7.89E-26	1.44E-21	0.818	0.577
ERRFI1	1	0.37406335	8.07E-14	1.47E-09	0.349	0.185
MSANTD3	1	0.36600062	1.82E-35	3.33E-31	0.36	0.083
VDAC1	1	0.36533478	1.43E-26	2.62E-22	0.891	0.734
KMT2E-AS1	1	0.36434854	8.14E-31	1.49E-26	0.289	0.055
IER3	1	0.36434702	4.35E-13	7.94E-09	0.275	0.123
RNF126	1	0.3639707	1.57E-28	2.87E-24	0.422	0.163
ANXA5	1	0.36304692	4.21E-25	7.68E-21	0.926	0.765
RNMT	1	0.36303041	9.68E-21	1.77E-16	0.554	0.329
TFPI	1	0.36164703	1.46E-24	2.67E-20	0.262	0.059
LINC00152	1	0.36101046	1.33E-19	2.42E-15	0.417	0.202
METAP2	1	0.35748118	1.43E-26	2.60E-22	0.849	0.665
BNIP3	1	0.35419554	7.12E-28	1.30E-23	0.998	0.99
ATF4	1	0.34915094	2.82E-28	5.14E-24	0.961	0.849

S100A16	1	0.34913691	5.61E-15	1.02E-10	0.405	0.231
SVIP	1	0.34765549	2.81E-20	5.12E-16	0.556	0.343
LINC00493	1	0.34335885	1.96E-28	3.57E-24	0.93	0.777
CA9	1	0.3424865	1.93E-16	3.52E-12	0.428	0.238
ORM2	1	0.34245047	7.28E-13	1.33E-08	0.176	0.053
TVP23B	1	0.34051617	1.15E-19	2.10E-15	0.453	0.238
PPP1R14C	1	0.33863668	2.22E-44	4.05E-40	0.32	0.032
PPDPF	1	0.33836784	8.47E-41	1.55E-36	1	1
RIPK2	1	0.33730246	2.48E-33	4.53E-29	0.333	0.073
CAPZA2	1	0.33531109	1.77E-22	3.23E-18	0.783	0.618
CCDC174	1	0.33500624	8.17E-28	1.49E-23	0.45	0.183
NT5E	1	0.33189411	1.00E-17	1.82E-13	0.424	0.221
ATP6V0E1	1	0.33101465	1.77E-24	3.24E-20	0.915	0.811
EVI2A	1	0.33077928	2.88E-38	5.25E-34	0.333	0.055
GADD45GIP1	1	0.33072938	8.93E-22	1.63E-17	0.829	0.697
NAMPT	1	0.3307142	2.11E-19	3.85E-15	0.547	0.336
CLIC4	1	0.33050961	7.84E-18	1.43E-13	0.523	0.329
MAP1LC3B	1	0.33008872	2.15E-19	3.92E-15	0.601	0.389
CNIH1	1	0.32787732	4.46E-22	8.13E-18	0.818	0.642
IGFBP5	1	0.32651745	4.04E-12	7.37E-08	0.203	0.075
TNS1	1	0.32572676	2.81E-28	5.12E-24	0.312	0.077
CHMP5	1	0.32218882	4.29E-21	7.83E-17	0.665	0.474
OSER1	1	0.31782132	1.13E-19	2.06E-15	0.459	0.249
CLDN12	1	0.31599988	5.23E-21	9.54E-17	0.384	0.167
ABL2	1	0.31445882	3.96E-28	7.23E-24	0.335	0.093
PFKP	1	0.31265853	3.78E-16	6.89E-12	0.502	0.317
RAB1A	1	0.31144167	1.30E-17	2.38E-13	0.791	0.625
FNIP2	1	0.3111727	2.72E-15	4.96E-11	0.324	0.151
AC090498.1	1	0.31089863	1.41E-51	2.58E-47	1	1
CDC37L1	1	0.31041356	3.02E-18	5.51E-14	0.49	0.282
TMSB4X	1	0.30889879	1.08E-08	0.00019781	0.8	0.687
NDUFB8	1	0.30803207	2.14E-17	3.91E-13	0.816	0.73
CLDND1	1	0.30757628	1.09E-19	1.99E-15	0.483	0.261
LINC-PINT	1	0.30684325	1.92E-21	3.50E-17	0.302	0.1
CHMP4B	1	0.30591833	1.51E-18	2.76E-14	0.688	0.528
SH3BGRL3	1	0.30514535	1.82E-22	3.33E-18	0.984	0.974
SNRNP27	1	0.30510652	1.51E-16	2.76E-12	0.506	0.311
FERMT2	1	0.30373136	1.45E-18	2.65E-14	0.467	0.257
C16orf13	1	0.30344049	1.39E-20	2.53E-16	0.862	0.687

SEC61G	1	0.30318955	5.92E-21	1.08E-16	0.998	0.996
SERPINA1	1	0.30182436	4.08E-07	0.00743597	0.961	0.975
PAIP2	1	0.2991784	1.22E-18	2.23E-14	0.793	0.663
EPB41L4A-AS1	1	0.29720424	8.60E-20	1.57E-15	0.932	0.822
MTHFD2	1	0.29493606	1.86E-12	3.39E-08	0.643	0.488
C7orf50	1	0.29358415	1.96E-15	3.58E-11	0.632	0.469
ZFYVE16	1	0.29244005	9.79E-29	1.79E-24	0.302	0.069
FAM210A	1	0.29212975	1.16E-21	2.11E-17	0.426	0.195
POLR1D	1	0.29066671	1.96E-17	3.58E-13	0.793	0.682
UBL3	1	0.29043352	9.28E-12	1.69E-07	0.523	0.376
SELK	1	0.28833242	6.99E-16	1.28E-11	0.88	0.748
UBE2B	1	0.28754691	4.53E-19	8.27E-15	0.82	0.675
HILPDA	1	0.28742237	8.90E-15	1.62E-10	0.585	0.385
SMS	1	0.28711892	1.55E-10	2.84E-06	0.564	0.461
SAP18	1	0.28664004	7.99E-18	1.46E-13	0.907	0.804
C2orf49	1	0.28631334	2.74E-21	4.99E-17	0.401	0.176
CRTAC1	1	0.28620577	1.35E-17	2.46E-13	0.275	0.097
ZFAND2A	1	0.28421556	6.00E-28	1.10E-23	0.326	0.084
PELI1	1	0.2820979	3.36E-20	6.13E-16	0.262	0.076
RHOD	1	0.28176173	1.23E-14	2.24E-10	0.473	0.289
ACTB	1	0.27928469	2.22E-14	4.05E-10	0.967	0.917
KITLG	1	0.27857717	8.77E-27	1.60E-22	0.306	0.076
PLA2G4A	1	0.27750881	1.19E-19	2.17E-15	0.329	0.124
MRPL54	1	0.27606908	3.22E-15	5.88E-11	0.514	0.328
NDUFC1	1	0.27522783	6.90E-18	1.26E-13	0.934	0.823
PDLIM4	1	0.27401472	2.16E-12	3.95E-08	0.901	0.813
LOX	1	0.27399018	3.67E-17	6.69E-13	0.279	0.101
SLC7A2	1	0.27383288	2.74E-27	5.00E-23	0.217	0.026
KAT5	1	0.27355539	5.96E-20	1.09E-15	0.362	0.149
AKAP12	1	0.27346544	8.48E-22	1.55E-17	0.225	0.047
UBE2I	1	0.272374	9.26E-17	1.69E-12	0.723	0.538
HNRNPDL	1	0.27192707	5.41E-16	9.87E-12	0.851	0.711
RPF2	1	0.26947986	1.53E-17	2.80E-13	0.436	0.23
COPS2	1	0.26876252	3.58E-13	6.54E-09	0.674	0.549
AKIRIN2	1	0.26720616	1.27E-18	2.31E-14	0.351	0.148
SH3GLB1	1	0.26689882	3.74E-14	6.83E-10	0.684	0.544
YAF2	1	0.26671355	2.52E-20	4.60E-16	0.372	0.154
IL11	1	0.26563856	1.91E-26	3.49E-22	0.147	0
SREK1IP1	1	0.26485332	3.57E-14	6.52E-10	0.461	0.284

CCDC59	1	0.26483615	5.88E-14	1.07E-09	0.506	0.328
TYMP	1	0.26463127	6.42E-16	1.17E-11	0.333	0.148
TRAM1	1	0.2631105	2.84E-15	5.18E-11	0.845	0.732
DDX41	1	0.26309963	7.91E-14	1.44E-09	0.273	0.118
MAR5	1	0.262598	2.08E-15	3.80E-11	0.409	0.227
PTBP3	1	0.26242879	5.16E-23	9.41E-19	0.318	0.1
TMOD1	1	0.26218183	5.04E-26	9.19E-22	0.25	0.047
CAMK2D	1	0.26167386	4.64E-23	8.46E-19	0.335	0.113
KLHL21	1	0.26144911	1.09E-23	1.99E-19	0.31	0.093
TBCA	1	0.26122015	1.78E-19	3.25E-15	0.973	0.916
SCAND1	1	0.26099426	2.40E-12	4.37E-08	0.618	0.491
SEMA3A	1	0.26023545	2.12E-31	3.87E-27	0.264	0.037
TIMM10B	1	0.26007111	4.88E-16	8.90E-12	0.393	0.202
DEFB1	1	0.25968297	7.87E-13	1.44E-08	0.211	0.076
COX6B1	1	0.25929682	9.15E-21	1.67E-16	0.979	0.946
SUN1	1	0.25850276	3.08E-17	5.61E-13	0.38	0.18
GAPDH	1	0.25726687	2.81E-12	5.13E-08	1	1
ACTR10	1	0.25692094	9.79E-13	1.79E-08	0.455	0.286
ABHD2	1	0.25517699	3.88E-25	7.08E-21	0.252	0.051
TRA2B	1	0.25311694	1.44E-11	2.62E-07	0.484	0.331
USMG5	1	0.25298052	3.75E-19	6.84E-15	0.961	0.892
GRPEL1	1	0.25241712	9.04E-15	1.65E-10	0.39	0.207
FGFR1OP2	1	0.25218887	4.15E-13	7.57E-09	0.486	0.318
CGGBP1	1	0.2515071	2.09E-12	3.81E-08	0.508	0.355
C4orf48	1	0.25029108	7.98E-12	1.46E-07	0.391	0.224
HSPA5	1	0.2501393	6.87E-07	0.0125435	0.516	0.412
ATP2B1	1	-0.2508693	1.93E-18	3.51E-14	0.109	0.315
MRC2	1	-0.2510658	3.10E-23	5.66E-19	0.093	0.336
HIST1H2AC	1	-0.2510761	6.15E-20	1.12E-15	0.078	0.286
GABPB1-AS1	1	-0.2512582	1.87E-19	3.42E-15	0.097	0.31
OTOS	1	-0.2516719	5.37E-19	9.81E-15	0.079	0.279
CHD9	1	-0.2525664	1.44E-15	2.63E-11	0.252	0.474
CDC42EP5	1	-0.2526914	3.50E-15	6.39E-11	0.291	0.495
CALM3	1	-0.2535208	1.00E-14	1.83E-10	0.533	0.716
PPP1CA	1	-0.2538231	2.70E-21	4.93E-17	0.141	0.38
SLC26A2	1	-0.2539003	8.44E-23	1.54E-18	0.062	0.284
SOD1	1	-0.2541929	1.49E-17	2.71E-13	0.928	0.974
IDH2	1	-0.254591	1.00E-22	1.83E-18	0.045	0.253
NUCB1	1	-0.2547486	1.38E-11	2.52E-07	0.496	0.642

ATP6AP1	1	-0.2550793	8.02E-21	1.46E-16	0.116	0.343
LSM2	1	-0.2557293	4.33E-14	7.91E-10	0.397	0.589
SSR1	1	-0.2562026	6.34E-15	1.16E-10	0.357	0.563
HOXA9	1	-0.2562503	5.62E-26	1.03E-21	0.041	0.271
KTN1	1	-0.2580287	1.26E-14	2.30E-10	0.49	0.672
RPL15	1	-0.2586706	8.31E-69	1.52E-64	1	1
ZFHX4	1	-0.2587573	2.31E-29	4.21E-25	0.035	0.284
RPS29	1	-0.2589569	1.98E-49	3.62E-45	1	1
H1F0	1	-0.2591548	1.18E-14	2.16E-10	0.209	0.42
PPP1CC	1	-0.2602365	2.38E-16	4.35E-12	0.428	0.638
ARL2	1	-0.2609399	1.99E-18	3.64E-14	0.314	0.564
PRKCDBP	1	-0.2612918	3.01E-14	5.50E-10	0.304	0.499
LAGE3	1	-0.2613427	5.44E-17	9.93E-13	0.366	0.602
SEC63	1	-0.2622643	1.23E-18	2.24E-14	0.285	0.537
ZCRB1	1	-0.26381	1.97E-17	3.60E-13	0.331	0.574
FGFBP2	1	-0.2646005	2.70E-09	4.93E-05	0.988	0.996
SLC38A1	1	-0.2649124	1.11E-19	2.02E-15	0.14	0.367
CBR1	1	-0.2650775	9.63E-23	1.76E-18	0.114	0.357
CHMP3	1	-0.2654778	4.54E-14	8.28E-10	0.38	0.564
KRT8	1	-0.2656101	1.29E-14	2.35E-10	0.233	0.433
PRKCSH	1	-0.2668497	2.00E-17	3.65E-13	0.308	0.541
GSTP1	1	-0.267147	4.62E-21	8.43E-17	0.946	0.985
EIF3J-AS1	1	-0.2675915	2.68E-22	4.89E-18	0.124	0.367
RPS3	1	-0.2679802	1.43E-60	2.60E-56	1	1
PCOLCE	1	-0.2680842	1.13E-16	2.05E-12	0.14	0.342
ST13	1	-0.2685603	3.67E-22	6.69E-18	0.917	0.972
RPS4Y1	1	-0.2689159	7.71E-30	1.41E-25	0.994	0.997
RCN3	1	-0.269077	2.52E-15	4.60E-11	0.295	0.512
C19orf43	1	-0.2691673	1.35E-19	2.46E-15	0.736	0.9
CRELD2	1	-0.26933	1.82E-25	3.32E-21	0.056	0.293
CYB5D2	1	-0.2696235	8.17E-25	1.49E-20	0.099	0.353
CA11	1	-0.2698104	3.36E-25	6.13E-21	0.074	0.318
TNFRSF1A	1	-0.2698929	4.64E-15	8.46E-11	0.264	0.465
MLEC	1	-0.2700341	7.29E-16	1.33E-11	0.264	0.469
TMED9	1	-0.2700547	4.12E-14	7.52E-10	0.51	0.69
RBM3	1	-0.2700687	5.98E-16	1.09E-11	0.388	0.603
RPS18	1	-0.2704696	2.80E-59	5.12E-55	1	1
PODNL1	1	-0.2705801	1.97E-16	3.60E-12	0.182	0.387
RPS23	1	-0.2708948	8.37E-55	1.53E-50	1	1

TCEA2	1	-0.2709067	1.09E-19	1.99E-15	0.229	0.47
IFITM2	1	-0.2717412	8.96E-14	1.63E-09	0.816	0.882
HNRNPM	1	-0.2717616	2.98E-18	5.44E-14	0.205	0.436
HDDC2	1	-0.2721457	3.77E-16	6.88E-12	0.399	0.614
COPZ2	1	-0.2722405	2.31E-25	4.21E-21	0.105	0.369
NXPH4	1	-0.2725706	2.00E-13	3.65E-09	0.155	0.328
BTF3	1	-0.2730204	3.87E-30	7.06E-26	0.994	0.999
SLC29A1	1	-0.2734311	7.01E-20	1.28E-15	0.203	0.444
C9orf3	1	-0.27365	1.48E-21	2.70E-17	0.202	0.462
P4HA1	1	-0.2743253	2.37E-14	4.33E-10	0.562	0.723
RPS4X	1	-0.2749549	8.22E-64	1.50E-59	1	1
PIGT	1	-0.2751768	1.49E-21	2.71E-17	0.138	0.379
CFH	1	-0.2762816	7.52E-09	0.0001372	0.118	0.243
SUMO1	1	-0.2767794	3.20E-18	5.85E-14	0.531	0.743
ERLEC1	1	-0.27815	3.79E-17	6.91E-13	0.281	0.505
HOXC10	1	-0.2781561	3.92E-14	7.16E-10	0.556	0.712
CKAP4	1	-0.2783003	1.13E-23	2.06E-19	0.112	0.362
CD302	1	-0.2789525	2.61E-22	4.76E-18	0.112	0.35
LAMTOR1	1	-0.2789823	1.55E-18	2.83E-14	0.382	0.615
NQO2	1	-0.2793995	8.50E-19	1.55E-14	0.227	0.456
DMKN	1	-0.279403	9.40E-19	1.72E-14	0.264	0.502
PRDX1	1	-0.2812339	9.75E-15	1.78E-10	0.733	0.844
C1orf21	1	-0.2820151	3.38E-16	6.17E-12	0.39	0.603
B3GNT7	1	-0.2820617	4.83E-11	8.81E-07	0.236	0.409
RPL6	1	-0.2822166	1.91E-48	3.48E-44	1	1
SLC1A5	1	-0.2831021	1.32E-26	2.41E-22	0.079	0.337
CCDC8	1	-0.2833142	3.22E-29	5.88E-25	0.033	0.279
RCN2	1	-0.2844631	3.41E-16	6.22E-12	0.486	0.687
RPL5	1	-0.2850325	4.45E-47	8.13E-43	1	1
NEDD8	1	-0.2863507	1.37E-17	2.50E-13	0.531	0.736
HERPUD1	1	-0.2872233	1.50E-17	2.74E-13	0.324	0.559
TRAPPC1	1	-0.2877913	6.51E-20	1.19E-15	0.24	0.487
SMIM14	1	-0.2879069	5.87E-24	1.07E-19	0.14	0.404
NMRK1	1	-0.2883669	1.29E-20	2.35E-16	0.196	0.436
RPL7A	1	-0.2900418	3.19E-59	5.82E-55	1	1
RPL14	1	-0.2908612	1.33E-58	2.42E-54	1	1
HSPA8	1	-0.2911205	1.95E-15	3.57E-11	0.271	0.479
SUMO2	1	-0.2914747	4.29E-30	7.82E-26	0.965	0.989
C14orf1	1	-0.2915306	5.31E-24	9.70E-20	0.126	0.382

ANKRD35	1	-0.2916665	6.61E-27	1.21E-22	0.074	0.326
MKX	1	-0.2922317	3.03E-25	5.53E-21	0.099	0.355
SRSF9	1	-0.2928318	2.64E-20	4.82E-16	0.341	0.599
RPL13	1	-0.2929626	7.75E-78	1.41E-73	1	1
SERTAD4-AS1	1	-0.2935859	1.71E-28	3.12E-24	0.068	0.331
HOXB6	1	-0.2937644	8.04E-20	1.47E-15	0.188	0.419
SEP1	1	-0.2938628	6.46E-22	1.18E-17	0.132	0.375
FKBP10	1	-0.2938639	1.49E-14	2.71E-10	0.37	0.562
NENF	1	-0.2943171	1.94E-24	3.53E-20	0.882	0.972
IFITM1	1	-0.2949122	4.07E-12	7.43E-08	0.324	0.506
ATP5D	1	-0.2955372	1.04E-22	1.90E-18	0.775	0.921
C12orf57	1	-0.297096	2.11E-28	3.85E-24	0.94	0.986
ATP5A1	1	-0.2972764	8.71E-19	1.59E-14	0.411	0.632
TOMM7	1	-0.297789	4.18E-58	7.63E-54	1	1
MAGEF1	1	-0.2978441	1.08E-30	1.97E-26	0.078	0.36
LY6E	1	-0.2991549	1.26E-15	2.29E-11	0.32	0.519
CYBRD1	1	-0.2994411	1.46E-19	2.66E-15	0.44	0.669
RPS5	1	-0.3003754	3.19E-61	5.81E-57	1	1
MXRA8	1	-0.3012781	3.42E-22	6.24E-18	0.149	0.398
RPS13	1	-0.3016685	1.67E-60	3.05E-56	1	1
FHL1	1	-0.3029719	6.05E-16	1.10E-11	0.324	0.537
FXYD1	1	-0.3036098	9.82E-10	1.79E-05	0.293	0.423
FIS1	1	-0.303733	6.89E-20	1.26E-15	0.517	0.725
S100A1	1	-0.3038477	6.89E-30	1.26E-25	1	1
MEG3	1	-0.304037	1.75E-21	3.19E-17	0.136	0.379
RPS6	1	-0.3043146	1.64E-80	2.99E-76	1	1
CTSA	1	-0.3049314	1.40E-29	2.56E-25	0.066	0.335
S100A13	1	-0.3050318	1.34E-38	2.44E-34	0.998	1
TPD52L1	1	-0.3054249	6.90E-15	1.26E-10	0.341	0.542
CCDC14	1	-0.3054585	4.46E-26	8.14E-22	0.11	0.372
RARRES3	1	-0.3061213	1.77E-31	3.23E-27	0.031	0.292
GPAA1	1	-0.3066066	5.72E-25	1.04E-20	0.159	0.427
TCEB2	1	-0.3066078	1.58E-31	2.89E-27	0.948	0.989
HSPB1	1	-0.3066509	5.91E-19	1.08E-14	0.578	0.79
PAM	1	-0.3071976	4.06E-20	7.40E-16	0.403	0.643
ERP29	1	-0.3074299	9.90E-22	1.81E-17	0.641	0.794
AFF3	1	-0.3080541	6.88E-36	1.26E-31	0.017	0.293
PCOLCE2	1	-0.3089047	5.23E-18	9.54E-14	0.907	0.956
ABHD14B	1	-0.3090294	7.33E-24	1.34E-19	0.186	0.459

LITAF	1	-0.3098671	4.40E-17	8.04E-13	0.374	0.573
SUMF2	1	-0.3100873	3.99E-20	7.28E-16	0.324	0.567
AHNAK	1	-0.3106145	4.85E-22	8.84E-18	0.267	0.527
SARAF	1	-0.310804	1.79E-21	3.26E-17	0.51	0.728
HMGB1	1	-0.311466	1.92E-21	3.50E-17	0.669	0.841
PDIA4	1	-0.3121131	4.91E-23	8.95E-19	0.184	0.445
GLTSCR2	1	-0.3121463	3.78E-26	6.89E-22	0.853	0.947
LUC7L3	1	-0.3123596	3.70E-22	6.76E-18	0.269	0.534
BEX4	1	-0.3123726	3.41E-20	6.21E-16	0.413	0.65
UMAD1	1	-0.3127311	9.15E-22	1.67E-17	0.184	0.433
PDGFRA	1	-0.3136753	1.42E-26	2.59E-22	0.068	0.317
NKX3-2	1	-0.3136929	1.21E-31	2.21E-27	0.027	0.285
RNASET2	1	-0.3139579	3.25E-24	5.93E-20	0.207	0.481
RPL10	1	-0.3140978	4.18E-86	7.63E-82	1	1
GSTA4	1	-0.3148587	3.79E-25	6.91E-21	0.184	0.458
SGCB	1	-0.3151887	1.91E-16	3.48E-12	0.316	0.533
FAM200B	1	-0.3163075	7.86E-26	1.43E-21	0.176	0.459
RPL8	1	-0.3165075	8.56E-77	1.56E-72	1	1
OS9	1	-0.3169398	7.17E-19	1.31E-14	0.347	0.578
PPIA	1	-0.3187903	2.65E-30	4.83E-26	0.946	0.986
RP11-71N10.1	1	-0.3189115	1.27E-35	2.32E-31	0.014	0.285
EFNA1	1	-0.3208353	6.31E-23	1.15E-18	0.196	0.463
NDRG2	1	-0.3214245	3.21E-36	5.85E-32	0.029	0.317
ITM2B	1	-0.322382	4.95E-22	9.03E-18	0.971	0.993
DDOST	1	-0.3228477	1.02E-20	1.87E-16	0.297	0.548
RPS2	1	-0.3234187	3.03E-72	5.53E-68	1	1
INSIG1	1	-0.324096	9.66E-15	1.76E-10	0.271	0.469
RPL9	1	-0.3242587	2.73E-79	4.98E-75	1	1
NME4	1	-0.3243815	6.97E-25	1.27E-20	0.242	0.52
CCDC88A	1	-0.325437	4.38E-24	7.99E-20	0.147	0.401
NDUFA3	1	-0.3260296	1.52E-25	2.78E-21	0.359	0.66
RPL19	1	-0.3285649	5.56E-90	1.01E-85	1	1
DYNLT1	1	-0.3292945	3.86E-27	7.04E-23	0.238	0.541
TCEA3	1	-0.329384	2.63E-27	4.79E-23	0.211	0.505
CRIP2	1	-0.3299812	5.21E-20	9.50E-16	0.266	0.503
SPTLC3	1	-0.3302242	8.32E-18	1.52E-13	0.107	0.308
SLC25A3	1	-0.3305424	4.29E-30	7.83E-26	0.868	0.956
TCEAL8	1	-0.3314708	3.52E-24	6.42E-20	0.574	0.794
JTB	1	-0.3318804	7.19E-24	1.31E-19	0.574	0.802

CAMTA1	1	-0.3325632	1.30E-23	2.38E-19	0.506	0.728
PLA2G16	1	-0.3333195	9.83E-24	1.79E-19	0.217	0.48
ZIC1	1	-0.3336026	4.07E-22	7.43E-18	0.126	0.364
RPL30	1	-0.3337826	7.93E-71	1.45E-66	1	1
RP11-553L6.5	1	-0.3337882	1.28E-32	2.34E-28	0.085	0.387
COMMD6	1	-0.3357894	1.88E-45	3.43E-41	0.994	1
EBPL	1	-0.3359207	4.64E-27	8.47E-23	0.221	0.512
H1FX	1	-0.3370185	7.54E-28	1.38E-23	0.079	0.342
TMED3	1	-0.3373501	6.70E-21	1.22E-16	0.328	0.571
RPL13A	1	-0.3375754	4.02E-98	7.34E-94	1	1
NDUFA11	1	-0.3377213	2.14E-29	3.91E-25	0.808	0.952
RP11-983P16.4	1	-0.3377979	8.12E-25	1.48E-20	0.203	0.472
HSP90AB1	1	-0.3396375	1.37E-33	2.50E-29	0.963	0.992
ANGPTL5	1	-0.3406802	1.99E-28	3.63E-24	0.128	0.412
RPS27A	1	-0.343528	2.44E-89	4.45E-85	1	1
LMAN2	1	-0.3438685	1.27E-25	2.31E-21	0.283	0.573
PTPRD-AS1	1	-0.3441851	3.79E-26	6.91E-22	0.19	0.472
NFIC	1	-0.344754	1.67E-24	3.06E-20	0.246	0.515
PIM3	1	-0.3450192	6.14E-21	1.12E-16	0.304	0.546
DUT	1	-0.3450767	3.21E-22	5.85E-18	0.471	0.694
CREG1	1	-0.3462293	5.14E-26	9.38E-22	0.413	0.687
GADD45B	1	-0.3466512	2.39E-21	4.36E-17	0.112	0.344
DDIT4	1	-0.3474059	9.72E-19	1.77E-14	0.32	0.555
ETFB	1	-0.3487776	5.16E-31	9.41E-27	0.287	0.613
PLD3	1	-0.3495356	4.50E-23	8.21E-19	0.36	0.618
UQCRH	1	-0.3512981	3.76E-37	6.87E-33	0.921	0.981
SEPP1	1	-0.3520323	2.20E-27	4.01E-23	0.205	0.502
RPS7	1	-0.3527158	7.37E-68	1.34E-63	1	1
ARL6IP4	1	-0.3531143	1.11E-34	2.02E-30	0.795	0.942
ENAH	1	-0.3532326	3.15E-33	5.75E-29	0.089	0.396
MPP6	1	-0.3533077	3.19E-33	5.82E-29	0.12	0.434
MYEOV2	1	-0.3537892	6.85E-26	1.25E-21	0.517	0.761
MSMP	1	-0.3539633	1.14E-08	0.00020726	0.244	0.4
LDHB	1	-0.3550758	5.28E-29	9.64E-25	0.715	0.884
HOXB8	1	-0.3558447	9.79E-41	1.79E-36	0.023	0.333
AC005152.3	1	-0.3558447	3.60E-32	6.57E-28	0.048	0.322
OAF	1	-0.3567534	6.14E-34	1.12E-29	0.052	0.343
CHAD	1	-0.3567702	9.35E-35	1.71E-30	0.041	0.328
CNPY2	1	-0.3575137	8.28E-26	1.51E-21	0.444	0.703

PRDX5	1	-0.3584625	9.06E-34	1.65E-29	0.837	0.946
SOCS3	1	-0.3585584	4.08E-31	7.45E-27	0.064	0.339
COL6A2	1	-0.3593526	4.23E-18	7.71E-14	0.498	0.683
EIF2S3	1	-0.3600629	1.29E-23	2.35E-19	0.37	0.64
PGAM1	1	-0.3616132	8.15E-31	1.49E-26	0.649	0.876
PSAP	1	-0.3632309	1.44E-26	2.63E-22	0.853	0.963
RPLP2	1	-0.3640597	2.46E-92	4.48E-88	1	1
MYLK	1	-0.3645178	4.68E-30	8.53E-26	0.078	0.357
RPL18	1	-0.3657693	1.30E-77	2.37E-73	1	1
COL5A2	1	-0.3657798	3.33E-22	6.09E-18	0.18	0.43
CD8B	1	-0.3658676	2.13E-37	3.89E-33	0.056	0.367
TSPAN4	1	-0.3660109	2.19E-28	4.00E-24	0.285	0.591
RPL23	1	-0.3670071	3.18E-62	5.81E-58	1	1
CANX	1	-0.3707603	1.81E-25	3.30E-21	0.583	0.801
MTDH	1	-0.3709965	4.18E-24	7.63E-20	0.465	0.701
TMEM219	1	-0.3712011	5.18E-30	9.46E-26	0.508	0.761
RPS17	1	-0.3725528	1.07E-76	1.96E-72	1	1
PNKD	1	-0.3732798	8.77E-37	1.60E-32	0.14	0.476
SRSF5	1	-0.3740521	6.61E-27	1.21E-22	0.444	0.712
ABHD14A	1	-0.3741619	8.09E-38	1.48E-33	0.085	0.416
PDE4DIP	1	-0.3752489	8.01E-31	1.46E-26	0.264	0.567
CUTA	1	-0.3780303	1.15E-33	2.10E-29	0.721	0.911
PRCP	1	-0.3797927	1.68E-40	3.07E-36	0.076	0.414
TPM2	1	-0.381041	8.39E-29	1.53E-24	0.411	0.686
POLR2L	1	-0.3816002	4.44E-25	8.10E-21	0.758	0.869
PRUNE2	1	-0.3841093	5.60E-34	1.02E-29	0.12	0.438
VKORC1	1	-0.3855109	1.06E-31	1.93E-27	0.934	0.989
RPS16	1	-0.3858657	6.50E-88	1.19E-83	1	1
CD63	1	-0.3862937	2.44E-34	4.45E-30	1	1
CD320	1	-0.3864119	2.56E-31	4.68E-27	0.314	0.631
ARL2BP	1	-0.389353	2.05E-27	3.75E-23	0.343	0.617
PPIB	1	-0.3895215	1.14E-29	2.08E-25	0.859	0.96
HIST1H1C	1	-0.3900105	1.13E-10	2.07E-06	0.302	0.467
TMEM230	1	-0.3907959	1.29E-29	2.35E-25	0.527	0.766
CMPK1	1	-0.3922455	5.59E-29	1.02E-24	0.607	0.797
RABAC1	1	-0.3926845	8.10E-35	1.48E-30	0.736	0.927
SERF2	1	-0.3931668	5.92E-44	1.08E-39	1	1
TUBB	1	-0.3939965	1.21E-32	2.20E-28	0.696	0.906
PNRC1	1	-0.3953319	5.46E-41	9.96E-37	0.868	0.986

NAP1L1	1	-0.3955018	8.17E-41	1.49E-36	0.926	0.978
TMEM98	1	-0.3983688	3.64E-40	6.64E-36	0.11	0.465
RPL23A	1	-0.3989243	8.42E-109	1.54E-104	1	1
IER2	1	-0.4040281	1.42E-30	2.58E-26	0.14	0.443
MORF4L2	1	-0.4044218	7.13E-33	1.30E-28	0.444	0.746
TXNIP	1	-0.4049059	1.87E-47	3.40E-43	0.021	0.369
ATPIF1	1	-0.4054337	7.48E-34	1.37E-29	0.564	0.799
ACTG1	1	-0.4054645	2.85E-29	5.19E-25	0.94	0.988
HLA-C	1	-0.4115436	4.41E-28	8.04E-24	0.884	0.957
NINJ1	1	-0.4123569	2.55E-30	4.66E-26	0.426	0.715
RPL3	1	-0.4125059	4.12E-100	7.52E-96	1	1
ARL6IP5	1	-0.4126716	2.04E-34	3.73E-30	0.378	0.707
COL9A2	1	-0.4147275	2.69E-27	4.91E-23	0.229	0.513
ARPC1A	1	-0.4152955	6.96E-38	1.27E-33	0.267	0.617
MYL6	1	-0.4159986	4.52E-43	8.24E-39	0.864	0.978
SSR4	1	-0.4186211	3.14E-42	5.72E-38	0.971	0.992
EEF1B2	1	-0.4188828	1.69E-56	3.08E-52	0.983	0.999
KDELR3	1	-0.4191347	6.24E-40	1.14E-35	0.06	0.387
ILF3-AS1	1	-0.420436	2.10E-39	3.84E-35	0.13	0.476
PNISR	1	-0.4215458	7.96E-36	1.45E-31	0.51	0.793
PLXDC2	1	-0.4231179	4.48E-33	8.17E-29	0.331	0.653
HLA-A	1	-0.426029	2.84E-29	5.18E-25	0.86	0.968
CHRDL2	1	-0.4268702	4.37E-30	7.98E-26	0.064	0.332
CCDC102B	1	-0.4273692	1.32E-50	2.40E-46	0.017	0.382
RPS3A	1	-0.4277178	3.15E-117	5.75E-113	1	1
RPL34	1	-0.4281204	2.12E-116	3.87E-112	1	1
DBP	1	-0.4333286	2.98E-33	5.43E-29	0.143	0.451
IGFBP7	1	-0.4334092	1.09E-23	1.98E-19	0.82	0.945
PKM	1	-0.4335537	8.83E-34	1.61E-29	0.874	0.974
PPP1R3C	1	-0.4359953	9.39E-27	1.71E-22	0.269	0.544
HEXB	1	-0.4363875	2.09E-33	3.81E-29	0.341	0.656
ASPN	1	-0.4381254	5.28E-15	9.64E-11	0.072	0.242
C7orf73	1	-0.4393037	1.64E-39	2.99E-35	0.504	0.835
SH3BGRL	1	-0.4394377	5.90E-37	1.08E-32	0.335	0.661
RP5-1097P24.1	1	-0.4394681	8.16E-41	1.49E-36	0.109	0.458
SSR2	1	-0.4425254	3.14E-41	5.73E-37	0.729	0.923
AC003090.1	1	-0.4452452	3.87E-53	7.06E-49	0.037	0.43
LRPAP1	1	-0.4458463	7.71E-32	1.41E-27	0.366	0.676
KDELR2	1	-0.4462288	5.08E-42	9.27E-38	0.713	0.925

BSG	1	-0.452837	1.02E-36	1.86E-32	0.547	0.812
SEC11A	1	-0.453401	9.12E-54	1.66E-49	0.773	0.96
CTSK	1	-0.4544186	2.70E-48	4.93E-44	0.112	0.508
CTSD	1	-0.4569717	5.94E-38	1.08E-33	0.767	0.95
ATRNL1	1	-0.45852	7.33E-41	1.34E-36	0.103	0.45
SPINT2	1	-0.4598344	4.65E-35	8.48E-31	0.287	0.615
PLPP1	1	-0.4615708	1.41E-39	2.56E-35	0.056	0.378
HSP90B1	1	-0.462315	8.92E-31	1.63E-26	0.725	0.898
TSPO	1	-0.4625495	2.69E-44	4.91E-40	0.661	0.92
PRELP	1	-0.4639212	1.57E-36	2.87E-32	0.948	0.997
CMB9-22P13.1	1	-0.4641554	1.26E-37	2.29E-33	0.215	0.557
EIF4B	1	-0.4652223	2.98E-42	5.44E-38	0.692	0.909
NUCB2	1	-0.4652667	1.54E-35	2.81E-31	0.318	0.651
HMGN3	1	-0.4665567	2.08E-50	3.80E-46	0.136	0.555
ZBTB20	1	-0.4667177	8.33E-49	1.52E-44	0.132	0.534
SLC44A1	1	-0.4668986	7.48E-41	1.36E-36	0.151	0.51
PCBP2	1	-0.469896	3.06E-53	5.59E-49	0.831	0.965
RNF130	1	-0.4701364	8.45E-38	1.54E-33	0.382	0.689
BHLHE41	1	-0.4714133	9.04E-40	1.65E-35	0.178	0.545
SOD3	1	-0.4725208	7.64E-25	1.39E-20	0.903	0.979
MT-ND5	1	-0.4746374	3.07E-27	5.61E-23	0.397	0.66
TMED10	1	-0.4767343	7.47E-43	1.36E-38	0.82	0.952
ERGIC3	1	-0.483092	8.39E-49	1.53E-44	0.734	0.905
GSN	1	-0.485531	4.36E-36	7.96E-32	0.364	0.676
SDC2	1	-0.4857198	8.40E-47	1.53E-42	0.703	0.938
VIM	1	-0.487177	2.35E-56	4.30E-52	0.996	1
PRDX4	1	-0.4889399	2.43E-39	4.43E-35	0.709	0.906
RPL7	1	-0.491871	1.01E-105	1.85E-101	1	1
AP2M1	1	-0.4931141	1.10E-44	2.02E-40	0.328	0.692
TSPAN3	1	-0.493885	1.12E-43	2.05E-39	0.318	0.672
HTRA1	1	-0.5004953	9.76E-49	1.78E-44	0.665	0.938
COL11A2	1	-0.5018136	1.45E-39	2.64E-35	0.36	0.701
HDLBP	1	-0.502075	1.30E-43	2.36E-39	0.233	0.602
CXXC5	1	-0.5023267	8.14E-52	1.48E-47	0.074	0.473
RPN2	1	-0.5028906	5.23E-52	9.55E-48	0.083	0.485
CALR	1	-0.5042819	4.22E-34	7.71E-30	0.502	0.769
ENPP1	1	-0.5054257	2.72E-51	4.96E-47	0.062	0.458
DNM3OS	1	-0.5089994	1.63E-46	2.97E-42	0.095	0.47
FMOD	1	-0.5110795	8.04E-53	1.47E-48	0.88	0.99

BRK1	1	-0.5113454	2.56E-60	4.68E-56	0.678	0.945
CHID1	1	-0.511793	1.15E-44	2.10E-40	0.374	0.712
MOXD1	1	-0.5118858	4.75E-43	8.68E-39	0.153	0.516
HMGN2	1	-0.5121143	2.96E-56	5.39E-52	0.828	0.961
BAMBI	1	-0.5129237	1.54E-23	2.82E-19	0.428	0.676
SPON2	1	-0.5156737	2.17E-38	3.96E-34	0.083	0.412
ATRAID	1	-0.5232247	6.44E-48	1.17E-43	0.707	0.931
INHBA	1	-0.5241581	3.07E-41	5.61E-37	0.126	0.49
DCN	1	-0.5267742	2.17E-62	3.96E-58	1	1
TRPS1	1	-0.5304784	1.38E-51	2.52E-47	0.172	0.593
NFIA	1	-0.531002	1.59E-58	2.91E-54	0.089	0.53
SDF4	1	-0.5323012	3.69E-50	6.73E-46	0.463	0.801
ITM2A	1	-0.533347	5.15E-42	9.40E-38	0.465	0.804
CCNG1	1	-0.5363186	1.82E-57	3.31E-53	0.182	0.627
TMED2	1	-0.5383159	4.74E-53	8.65E-49	0.576	0.884
RPL10A	1	-0.5388076	3.91E-117	7.13E-113	1	1
MALAT1	1	-0.5401304	1.17E-47	2.13E-43	1	1
YBX1	1	-0.5466886	1.22E-83	2.22E-79	0.953	0.997
PPT1	1	-0.5500938	3.14E-55	5.73E-51	0.184	0.614
GNAS	1	-0.5514462	1.98E-48	3.62E-44	0.442	0.758
CST3	1	-0.5538385	1.34E-66	2.44E-62	0.99	1
TPT1	1	-0.5541043	5.97E-126	1.09E-121	1	1
MIA	1	-0.5567925	1.02E-61	1.86E-57	0.988	1
VCAN	1	-0.563933	1.52E-35	2.78E-31	0.231	0.556
FCGRT	1	-0.5650491	1.19E-53	2.17E-49	0.434	0.779
FAM46A	1	-0.5691337	1.20E-34	2.18E-30	0.391	0.689
DUSP1	1	-0.5691386	1.34E-42	2.45E-38	0.269	0.631
FTL	1	-0.57481	4.95E-108	9.03E-104	1	1
CTGF	1	-0.576231	6.69E-21	1.22E-16	0.223	0.473
GRN	1	-0.5772715	7.43E-64	1.36E-59	0.105	0.573
S100A4	1	-0.577966	3.42E-25	6.25E-21	0.671	0.849
SSPN	1	-0.5807199	4.29E-60	7.83E-56	0.099	0.545
NNMT	1	-0.5823933	1.59E-43	2.89E-39	0.851	0.965
GOLIM4	1	-0.5824706	3.65E-33	6.65E-29	0.215	0.535
EIF3L	1	-0.5845938	1.73E-68	3.16E-64	0.725	0.954
RBP4	1	-0.5875638	4.00E-50	7.31E-46	0.655	0.927
HIST1H4C	1	-0.5886769	2.96E-51	5.41E-47	0.246	0.663
SSR3	1	-0.5911096	8.34E-62	1.52E-57	0.789	0.952
RPS8	1	-0.5915358	1.44E-143	2.62E-139	1	1

FOS	1	-0.592746	4.10E-54	7.49E-50	0.372	0.78
PTRF	1	-0.5969537	5.45E-58	9.94E-54	0.234	0.665
DDR2	1	-0.6067559	5.05E-54	9.22E-50	0.262	0.657
PTMS	1	-0.609033	5.11E-65	9.32E-61	0.384	0.817
RPLP0	1	-0.6096365	2.02E-136	3.69E-132	1	1
MAGED1	1	-0.6157156	1.65E-39	3.02E-35	0.056	0.378
PEBP1	1	-0.6184639	1.93E-72	3.53E-68	0.638	0.932
SERPINA5	1	-0.6205771	1.07E-53	1.95E-49	0.417	0.772
YBX3	1	-0.6208559	1.37E-66	2.50E-62	0.636	0.945
JUNB	1	-0.623276	1.04E-56	1.90E-52	0.331	0.797
ARHGAP29	1	-0.6267197	3.03E-52	5.53E-48	0.225	0.635
PPIC	1	-0.6283074	1.93E-58	3.51E-54	0.223	0.674
RPS20	1	-0.6284855	3.77E-129	6.88E-125	1	1
ID4	1	-0.6463677	2.18E-53	3.99E-49	0.221	0.64
COL11A1	1	-0.6465449	5.19E-63	9.46E-59	0.496	0.899
TTC3	1	-0.6491146	6.32E-68	1.15E-63	0.415	0.833
HNRNPA1	1	-0.6498419	2.16E-97	3.93E-93	0.973	1
PDIA3	1	-0.6577465	2.36E-59	4.31E-55	0.362	0.755
CTSC	1	-0.6604874	3.57E-38	6.52E-34	0.074	0.396
PABPC1	1	-0.6613887	1.42E-80	2.60E-76	0.694	0.963
CYR61	1	-0.6631298	2.25E-48	4.11E-44	0.141	0.544
MDFI	1	-0.6652726	5.27E-63	9.61E-59	0.2	0.671
CITED2	1	-0.6668413	9.53E-54	1.74E-49	0.215	0.628
RPS11	1	-0.6682852	4.09E-126	7.47E-122	0.99	1
CEBPD	1	-0.682039	9.84E-51	1.80E-46	0.638	0.916
RPL4	1	-0.6825729	5.38E-126	9.81E-122	0.998	1
GAS6	1	-0.6921987	2.47E-69	4.51E-65	0.134	0.622
NPC2	1	-0.6929718	1.18E-109	2.15E-105	0.936	1
MT-ND4	1	-0.7004949	8.14E-55	1.48E-50	0.915	0.953
FBXO2	1	-0.716477	6.03E-79	1.10E-74	0.541	0.924
THY1	1	-0.7175529	1.75E-61	3.19E-57	0.18	0.631
H3F3A	1	-0.7176209	1.60E-103	2.93E-99	0.882	0.996
SNAI2	1	-0.7226601	4.27E-57	7.79E-53	0.207	0.645
EID1	1	-0.7303049	1.93E-104	3.53E-100	0.767	0.992
XAGE2	1	-0.7322098	3.77E-29	6.87E-25	0.01	0.234
SLC14A1	1	-0.7363293	5.20E-77	9.49E-73	0.147	0.66
CCNI	1	-0.7377713	7.33E-121	1.34E-116	0.988	0.999
S100B	1	-0.7388868	2.44E-72	4.46E-68	0.634	0.939
CD9	1	-0.740901	1.58E-64	2.87E-60	0.264	0.712

ID2	1	-0.7424591	6.99E-52	1.27E-47	0.24	0.642
C2orf40	1	-0.7480685	2.51E-62	4.58E-58	0.986	0.999
CRTAP	1	-0.7567227	5.58E-94	1.02E-89	0.453	0.888
IGFBP6	1	-0.7620427	3.53E-51	6.44E-47	0.541	0.842
FXYD6	1	-0.8035126	1.30E-102	2.38E-98	0.554	0.97
NGFRAP1	1	-0.8069821	1.68E-106	3.07E-102	0.694	0.979
GPX3	1	-0.8081826	1.82E-87	3.31E-83	0.857	0.999
EEF2	1	-0.8188934	2.45E-102	4.48E-98	0.973	0.999
EIF3H	1	-0.8189114	2.39E-133	4.36E-129	0.95	1
APOD	1	-0.8267272	2.58E-45	4.71E-41	0.262	0.64
LECT1	1	-0.8388456	4.75E-72	8.67E-68	0.736	0.978
COL9A3	1	-0.848247	1.07E-58	1.95E-54	0.455	0.833
EEF1A1	1	-0.850051	2.71E-177	4.94E-173	1	1
TCF4	1	-0.8517759	7.02E-92	1.28E-87	0.12	0.7
NEAT1	1	-0.8577943	1.68E-107	3.07E-103	0.986	1
RHOBTB3	1	-0.8674965	1.75E-90	3.20E-86	0.264	0.794
FTH1	1	-0.8774478	3.15E-137	5.75E-133	1	1
CD99	1	-0.8916233	1.50E-131	2.74E-127	0.839	0.996
SLC40A1	1	-0.9186777	1.62E-91	2.95E-87	0.029	0.599
C2orf82	1	-0.9257884	1.73E-89	3.15E-85	0.973	0.999
MGP	1	-0.933233	3.21E-83	5.85E-79	0.994	1
PDIA6	1	-0.935711	1.54E-107	2.81E-103	0.38	0.9
B2M	1	-0.939543	2.06E-134	3.76E-130	0.977	0.999
ACAN	1	-0.9407962	3.06E-115	5.59E-111	0.847	0.994
CP	1	-0.9786229	3.20E-98	5.83E-94	0.359	0.882
CRISPLD1	1	-0.9815285	2.30E-79	4.21E-75	0.017	0.528
ADIRF	1	-0.9873936	2.28E-83	4.17E-79	0.324	0.833
MT-CO3	1	-1.0058752	1.42E-89	2.59E-85	0.781	0.938
P4HB	1	-1.0137568	1.93E-115	3.53E-111	0.62	0.978
MT-CO1	1	-1.0168855	1.12E-79	2.04E-75	0.895	0.963
SPARC	1	-1.0260532	3.63E-98	6.62E-94	0.289	0.871
ID1	1	-1.0503243	1.06E-67	1.93E-63	0.211	0.696
MT-ND3	1	-1.0544475	5.77E-103	1.05E-98	0.331	0.873
ITM2C	1	-1.0613243	2.43E-116	4.43E-112	0.234	0.84
MT-ND1	1	-1.0825146	1.80E-102	3.28E-98	0.242	0.827
ZFP36L2	1	-1.0831364	1.02E-87	1.85E-83	0.081	0.643
ISLR	1	-1.1118651	2.57E-128	4.69E-124	0.13	0.816
MT-CYB	1	-1.1406553	1.59E-101	2.91E-97	0.372	0.88
SLPI	1	-1.1508486	5.63E-42	1.03E-37	0.891	0.996

MT-CO2	1	-1.1579549	1.02E-94	1.87E-90	0.824	0.963
CAPS	1	-1.1744057	1.77E-110	3.24E-106	0.618	0.967
MT-ATP6	1	-1.1883699	1.46E-105	2.67E-101	0.558	0.936
MT-ND2	1	-1.2099094	2.79E-107	5.09E-103	0.266	0.855
PAX1	1	-1.2130984	4.89E-141	8.93E-137	0.32	0.929
TSC22D1	1	-1.3347035	2.26E-114	4.13E-110	0.45	0.928
ID3	1	-1.352387	3.41E-106	6.23E-102	0.147	0.77
OGN	1	-1.4029847	5.10E-136	9.30E-132	0.841	0.994
SPTSSB	1	-1.4088168	2.66E-108	4.86E-104	0.01	0.649
PLAC9	1	-1.4256722	1.60E-147	2.92E-143	0.517	0.99
COL2A1	1	-1.4424359	5.02E-137	9.16E-133	0.326	0.952
FRZB	1	-1.5239984	2.78E-94	5.07E-90	0.182	0.761
CLEC3A	1	-2.1198946	1.76E-172	3.20E-168	0.233	0.97
**gene**	**cluster**	**avg_logFC**	**p_val**	**p_val_adj**	**pct.1**	**pct.2**
SPP1	2	2.46846227	9.05E-112	1.65E-107	0.862	0.093
MT2A	2	1.70877456	1.42E-122	2.60E-118	1	1
PRG4	2	1.69357023	1.35E-73	2.47E-69	0.591	0.021
CTSL	2	1.42620285	8.18E-81	1.49E-76	0.877	0.406
HMGA1	2	1.32901031	1.21E-95	2.21E-91	0.831	0.182
UPP1	2	1.29720318	1.73E-106	3.15E-102	0.809	0.082
SOD2	2	1.26181872	1.59E-81	2.89E-77	0.996	0.872
S100A2	2	1.23798166	1.53E-47	2.80E-43	0.68	0.254
TM4SF1	2	1.1535034	1.92E-67	3.50E-63	0.818	0.333
PTGS2	2	1.11148959	1.30E-80	2.36E-76	0.669	0.056
CD55	2	1.03278673	8.70E-75	1.59E-70	0.892	0.429
VEGFA	2	1.02138342	1.87E-77	3.42E-73	0.86	0.333
IGFBP3	2	1.00309022	6.52E-54	1.19E-49	0.502	0.04
TNFRSF12A	2	0.99721361	5.56E-73	1.01E-68	0.71	0.135
FN1	2	0.95901593	8.87E-58	1.62E-53	0.998	0.993
BMP2	2	0.92266865	1.05E-61	1.92E-57	0.519	0.019
WTAP	2	0.92037518	7.00E-64	1.28E-59	0.879	0.459
COMP	2	0.89199081	9.14E-25	1.67E-20	0.903	0.709
MT1X	2	0.89132918	7.21E-49	1.32E-44	1	1
PMP22	2	0.88573962	3.12E-68	5.70E-64	0.922	0.545
STK17A	2	0.88153502	8.82E-60	1.61E-55	0.638	0.138
C11orf96	2	0.87502218	4.52E-51	8.25E-47	0.492	0.049
MRPS6	2	0.86393233	4.27E-52	7.79E-48	0.985	0.848
FABP5	2	0.86390377	2.63E-17	4.81E-13	0.458	0.245
ERO1A	2	0.81550087	7.02E-57	1.28E-52	0.731	0.28

NRP2	2	0.80659621	6.56E-48	1.20E-43	0.519	0.086
CCDC85B	2	0.8035535	3.23E-46	5.90E-42	0.873	0.576
DDIT4L	2	0.77888341	8.94E-37	1.63E-32	0.816	0.494
MIF	2	0.77775291	2.62E-101	4.77E-97	1	1
NDRG1	2	0.77340843	1.72E-57	3.14E-53	0.758	0.287
SERPINE2	2	0.72297339	1.04E-29	1.89E-25	1	0.995
SNHG12	2	0.71903515	3.20E-48	5.84E-44	0.678	0.245
BZW1	2	0.70631342	1.13E-49	2.06E-45	0.794	0.385
TMSB10	2	0.70073591	1.04E-44	1.90E-40	0.998	0.993
RABGGTB	2	0.67736131	1.80E-50	3.29E-46	0.833	0.501
TIMP3	2	0.65644813	1.86E-23	3.39E-19	0.689	0.427
PPP3CA	2	0.64789649	3.74E-34	6.82E-30	0.881	0.573
SLC25A37	2	0.64737682	1.94E-51	3.55E-47	0.928	0.597
CXCL8	2	0.6395576	6.14E-17	1.12E-12	0.167	0.007
MT1M	2	0.62086868	1.21E-18	2.21E-14	0.784	0.65
MT1G	2	0.5966878	1.56E-16	2.85E-12	1	0.998
C15orf48	2	0.59420241	1.52E-25	2.78E-21	0.256	0.014
ISG20	2	0.59350479	5.73E-34	1.05E-29	0.655	0.324
IER3	2	0.59247842	2.79E-30	5.09E-26	0.445	0.119
SMOX	2	0.58521445	1.56E-30	2.84E-26	0.504	0.172
TREM1	2	0.5788892	1.82E-44	3.31E-40	0.381	0.005
GSTO1	2	0.57215735	1.88E-35	3.43E-31	0.845	0.578
BAALC	2	0.55466654	1.11E-20	2.02E-16	0.591	0.338
SLC2A1	2	0.55375843	2.16E-35	3.94E-31	0.434	0.082
SLC3A2	2	0.5513741	8.37E-21	1.53E-16	0.443	0.186
TGFBI	2	0.54654149	8.99E-26	1.64E-21	0.443	0.145
SPRY2	2	0.54598626	1.11E-39	2.03E-35	0.439	0.061
RGCC	2	0.54463461	6.81E-31	1.24E-26	0.939	0.725
BHLHE40	2	0.53820169	5.80E-19	1.06E-14	0.602	0.38
EIF4EBP1	2	0.53668883	2.15E-27	3.93E-23	0.682	0.394
ENO2	2	0.52908586	8.88E-36	1.62E-31	0.597	0.228
EIF1	2	0.52124361	8.34E-55	1.52E-50	1	1
MT1H	2	0.5206268	7.53E-14	1.37E-09	0.958	0.942
CLEC2B	2	0.52062232	7.60E-18	1.39E-13	0.316	0.093
COX17	2	0.51681302	2.08E-32	3.79E-28	0.854	0.587
ZNF593	2	0.515339	4.99E-34	9.11E-30	0.646	0.308
CCND1	2	0.50963152	3.10E-35	5.66E-31	0.422	0.07
ARF6	2	0.508897	1.05E-32	1.92E-28	0.689	0.357
HLA-DRB1	2	0.49764819	1.69E-33	3.09E-29	0.487	0.131

NT5E	2	0.48762422	8.19E-27	1.49E-22	0.532	0.214
PLAUR	2	0.48611986	4.01E-28	7.32E-24	0.513	0.191
S100A16	2	0.47072809	9.32E-22	1.70E-17	0.502	0.233
VAPA	2	0.46679365	3.36E-31	6.13E-27	0.962	0.839
PPP1R15A	2	0.46581341	1.61E-27	2.95E-23	0.458	0.147
TGM2	2	0.46355039	8.27E-28	1.51E-23	0.511	0.191
ERRFI1	2	0.46246284	2.28E-18	4.16E-14	0.426	0.182
CD44	2	0.4588083	3.27E-29	5.96E-25	0.898	0.639
CTHRC1	2	0.4529481	5.79E-25	1.06E-20	0.328	0.056
SELK	2	0.44909541	6.24E-28	1.14E-23	0.892	0.727
PHLDA1	2	0.44716311	2.85E-22	5.21E-18	0.273	0.037
PPP1CB	2	0.44578871	9.54E-18	1.74E-13	0.82	0.648
RNF145	2	0.4423916	1.60E-16	2.92E-12	0.445	0.219
ZNF385D	2	0.44202119	6.59E-20	1.20E-15	0.68	0.462
RELB	2	0.44184621	2.96E-27	5.41E-23	0.367	0.075
AK2	2	0.43911713	7.21E-17	1.32E-12	0.701	0.529
BTG1	2	0.43791386	7.59E-23	1.38E-18	0.977	0.918
FNIP2	2	0.43371019	1.40E-26	2.55E-22	0.468	0.156
DDIT3	2	0.43217488	3.94E-16	7.19E-12	0.519	0.298
EIF4A2	2	0.43180993	9.44E-33	1.72E-28	0.996	0.977
PPFIBP1	2	0.42784593	8.69E-17	1.59E-12	0.547	0.34
RGS2	2	0.42757365	2.60E-12	4.74E-08	0.494	0.294
MT1E	2	0.42603571	5.73E-13	1.05E-08	1	1
CHI3L2	2	0.42198224	9.41E-09	0.00017167	0.544	0.399
DNER	2	0.41801481	1.57E-21	2.86E-17	0.614	0.336
SLC5A3	2	0.41705641	7.34E-15	1.34E-10	0.57	0.359
TNFRSF11B	2	0.41650765	2.49E-06	0.04539279	0.663	0.576
ADSS	2	0.40390654	2.55E-16	4.66E-12	0.585	0.389
ARRDC3	2	0.40283679	8.44E-15	1.54E-10	0.462	0.254
VIMP	2	0.40275297	1.61E-20	2.94E-16	0.79	0.557
RNF114	2	0.40171471	1.36E-17	2.49E-13	0.593	0.366
HMGA2	2	0.39798508	8.20E-33	1.50E-28	0.294	0.005
TRMT112	2	0.39792786	4.27E-22	7.79E-18	0.939	0.828
EMP3	2	0.39463023	3.59E-22	6.54E-18	0.93	0.828
CTNNB1	2	0.39453182	1.09E-09	1.99E-05	0.411	0.252
FOSL1	2	0.39193801	1.94E-29	3.54E-25	0.278	0.009
ARPP19	2	0.38831729	1.64E-17	2.98E-13	0.597	0.371
EIF1B	2	0.38790458	1.22E-21	2.23E-17	0.778	0.541
TMEM38B	2	0.38739779	2.10E-17	3.84E-13	0.576	0.361

SFT2D1	2	0.38307048	1.73E-21	3.15E-17	0.773	0.566
FAM162A	2	0.38272943	2.55E-20	4.65E-16	0.915	0.788
CLK1	2	0.37973296	3.26E-17	5.94E-13	0.593	0.366
CSGALNACT2	2	0.37926753	7.94E-24	1.45E-19	0.364	0.089
GBE1	2	0.37233333	2.39E-13	4.37E-09	0.591	0.406
ATP6V1G1	2	0.37046664	8.98E-18	1.64E-13	0.96	0.93
AC090498.1	2	0.36406413	5.84E-52	1.07E-47	1	1
SLC7A2	2	0.36389037	1.46E-25	2.67E-21	0.273	0.023
GPRC5A	2	0.35990211	1.78E-20	3.24E-16	0.347	0.098
GRB10	2	0.35939132	9.70E-27	1.77E-22	0.261	0.012
PFDN2	2	0.35659073	1.47E-15	2.68E-11	0.642	0.483
SERPINE1	2	0.35525824	9.39E-19	1.71E-14	0.265	0.051
PTGES3	2	0.35391317	1.39E-22	2.54E-18	0.96	0.893
SH3BGRL3	2	0.35201191	5.52E-24	1.01E-19	0.996	0.981
LRRFIP1	2	0.35112021	1.43E-17	2.61E-13	0.494	0.245
DNAJB9	2	0.34987575	8.54E-13	1.56E-08	0.718	0.541
GOLT1B	2	0.34949794	1.38E-15	2.52E-11	0.449	0.231
SLC20A1	2	0.34900248	1.59E-17	2.91E-13	0.328	0.103
ABI3BP	2	0.3489452	2.18E-06	0.03975274	0.504	0.413
SMS	2	0.34725009	5.27E-14	9.61E-10	0.583	0.413
VMP1	2	0.34495659	5.91E-08	0.0010792	0.578	0.436
GPX1	2	0.34416015	5.53E-14	1.01E-09	0.883	0.758
AIMP1	2	0.34358389	3.57E-14	6.51E-10	0.604	0.417
SGIP1	2	0.33685049	1.89E-20	3.45E-16	0.235	0.026
VDAC1	2	0.33649862	2.00E-15	3.65E-11	0.852	0.706
USMG5	2	0.33632266	9.17E-24	1.67E-19	0.975	0.893
PTP4A1	2	0.33433449	5.14E-10	9.38E-06	0.451	0.294
GADD45GIP1	2	0.33381052	1.02E-16	1.86E-12	0.809	0.608
CCL20	2	0.33374455	4.23E-12	7.72E-08	0.106	0
LINC00152	2	0.33360202	2.43E-11	4.44E-07	0.388	0.203
TFPI	2	0.33074617	1.85E-22	3.37E-18	0.254	0.026
PGK1	2	0.32973655	2.67E-10	4.88E-06	0.909	0.841
COX7A2	2	0.32930125	2.13E-24	3.89E-20	0.996	0.972
METAP2	2	0.32729542	6.42E-19	1.17E-14	0.814	0.564
CAMK2D	2	0.32479814	1.61E-17	2.93E-13	0.364	0.131
SH3BP5	2	0.3222563	4.42E-12	8.06E-08	0.449	0.266
FAP	2	0.31933957	1.22E-21	2.23E-17	0.314	0.065
NTMT1	2	0.31777677	6.55E-16	1.19E-11	0.47	0.238
RHOC	2	0.31597442	2.19E-13	4.00E-09	0.845	0.737

NOL7	2	0.31355968	3.94E-13	7.19E-09	0.644	0.469
LINC00493	2	0.31273089	1.11E-16	2.02E-12	0.9	0.755
FAM96B	2	0.31148506	4.36E-17	7.95E-13	0.788	0.606
PPP1R14C	2	0.31068516	1.35E-23	2.46E-19	0.286	0.037
FHL2	2	0.30923508	4.86E-20	8.87E-16	0.326	0.082
PDE4B	2	0.30371249	2.71E-17	4.94E-13	0.337	0.11
GNG5	2	0.30328079	8.88E-12	1.62E-07	0.642	0.492
TGIF1	2	0.30294059	3.16E-09	5.77E-05	0.479	0.326
C11orf31	2	0.30292028	6.03E-15	1.10E-10	0.799	0.613
CLDND1	2	0.3016047	6.08E-15	1.11E-10	0.494	0.268
WDR45B	2	0.29718777	5.77E-13	1.05E-08	0.4	0.203
AKAP12	2	0.29586352	3.42E-17	6.25E-13	0.246	0.047
ANXA5	2	0.29531912	1.26E-13	2.30E-09	0.873	0.762
SNRPD1	2	0.29385183	8.94E-11	1.63E-06	0.604	0.457
NBPF14	2	0.29346112	1.02E-16	1.85E-12	0.301	0.086
POMP	2	0.28929834	1.37E-15	2.50E-11	0.886	0.737
POLE4	2	0.28850271	1.89E-13	3.45E-09	0.604	0.406
KITLG	2	0.2855786	4.25E-21	7.75E-17	0.301	0.058
CCDC59	2	0.28513646	1.80E-12	3.28E-08	0.477	0.277
CNIH1	2	0.28322599	6.99E-13	1.28E-08	0.786	0.632
KDM7A	2	0.28303559	2.30E-20	4.20E-16	0.275	0.047
NPR3	2	0.28237139	1.73E-20	3.15E-16	0.182	0
SEC61G	2	0.28169459	7.75E-13	1.41E-08	0.994	0.993
TYMP	2	0.28072967	2.44E-10	4.46E-06	0.339	0.168
UFM1	2	0.27832529	1.73E-11	3.15E-07	0.64	0.483
CDKN2B	2	0.27588402	2.19E-22	4.00E-18	0.244	0.021
C4orf48	2	0.27442185	2.95E-09	5.38E-05	0.367	0.2
EPB41L4A-AS1	2	0.27375865	6.18E-12	1.13E-07	0.915	0.844
EPS8	2	0.27327906	1.20E-15	2.20E-11	0.369	0.145
RAB13	2	0.27267817	2.11E-12	3.85E-08	0.917	0.79
TBCA	2	0.27154099	6.56E-16	1.20E-11	0.975	0.907
CLMP	2	0.26971824	1.31E-15	2.39E-11	0.284	0.082
BTG3	2	0.26959175	5.48E-11	9.99E-07	0.383	0.2
GJA1	2	0.26954093	1.03E-13	1.89E-09	0.252	0.072
EVI2A	2	0.26936204	7.16E-18	1.31E-13	0.286	0.068
KLF3	2	0.26837862	1.80E-06	0.03277259	0.309	0.191
TMEM45A	2	0.26652763	2.01E-07	0.00366283	0.82	0.737
SVIP	2	0.26544302	1.40E-08	0.00025565	0.487	0.333
SAR1A	2	0.26462785	1.15E-09	2.10E-05	0.502	0.333

RND3	2	0.26290189	2.95E-12	5.39E-08	0.22	0.061
SLC39A14	2	0.26062294	3.33E-09	6.08E-05	0.606	0.441
SH3GLB1	2	0.26016473	1.99E-10	3.63E-06	0.674	0.534
ATP6V0B	2	0.25860005	1.19E-08	0.00021659	0.585	0.445
NDUFB8	2	0.25858304	8.59E-10	1.57E-05	0.818	0.692
TVP23B	2	0.25601637	1.65E-10	3.02E-06	0.379	0.207
MRPL41	2	0.25583114	3.76E-09	6.86E-05	0.68	0.564
MGST1	2	0.25331827	6.73E-08	0.0012278	0.324	0.175
SLC25A36	2	0.25015269	5.34E-08	0.0009748	0.506	0.366
SLC38A1	2	-0.2501978	1.34E-16	2.44E-12	0.138	0.371
CMPK1	2	-0.2507265	3.88E-14	7.07E-10	0.627	0.832
GABARAPL2	2	-0.2507528	7.40E-14	1.35E-09	0.769	0.914
ANXA4	2	-0.2515085	2.51E-10	4.58E-06	0.246	0.427
ISM1	2	-0.2526322	9.40E-25	1.72E-20	0.021	0.252
C1orf122	2	-0.2530799	3.76E-11	6.85E-07	0.199	0.385
MLEC	2	-0.2535394	1.54E-12	2.82E-08	0.21	0.413
COX7A2L	2	-0.2538718	2.66E-15	4.85E-11	0.826	0.921
MRC2	2	-0.2538904	4.86E-20	8.87E-16	0.066	0.296
HINT1	2	-0.2545868	2.52E-19	4.59E-15	0.987	0.993
TUBB2A	2	-0.2551192	1.58E-19	2.88E-15	0.051	0.266
YPEL3	2	-0.2551522	5.93E-19	1.08E-14	0.091	0.326
GPAA1	2	-0.2552101	8.51E-16	1.55E-11	0.165	0.401
GADD45B	2	-0.2558034	1.33E-15	2.43E-11	0.129	0.354
MPC2	2	-0.2561554	6.11E-14	1.11E-09	0.282	0.51
LRRC2	2	-0.2567093	1.09E-17	1.98E-13	0.1	0.329
CDC42EP5	2	-0.2574187	3.71E-12	6.77E-08	0.294	0.503
SLC29A1	2	-0.2578715	2.28E-14	4.16E-10	0.197	0.427
PRPSAP1	2	-0.2581311	3.17E-15	5.78E-11	0.153	0.378
RCN3	2	-0.2587097	1.16E-13	2.12E-09	0.195	0.417
RPL37	2	-0.259641	9.44E-37	1.72E-32	1	1
EIF3K	2	-0.2617445	5.41E-16	9.87E-12	0.822	0.932
NENF	2	-0.2622727	7.08E-16	1.29E-11	0.85	0.958
MATN4	2	-0.2634737	9.10E-16	1.66E-11	0.066	0.259
C14orf1	2	-0.2639866	4.84E-17	8.83E-13	0.131	0.368
CTSF	2	-0.2649402	9.70E-21	1.77E-16	0.085	0.336
C19orf43	2	-0.2653196	8.72E-15	1.59E-10	0.744	0.89
MAGEF1	2	-0.2653615	2.15E-19	3.92E-15	0.093	0.336
NPM1	2	-0.265432	2.19E-16	4.00E-12	0.996	1
C12orf57	2	-0.2656198	1.38E-16	2.51E-12	0.943	0.984

UBA52	2	-0.2662148	1.07E-38	1.95E-34	1	1
RPS12	2	-0.2664404	8.28E-41	1.51E-36	1	1
JTB	2	-0.2676862	1.34E-13	2.44E-09	0.57	0.781
PIK3R1	2	-0.2677933	6.30E-15	1.15E-10	0.051	0.226
ANG	2	-0.2681133	2.16E-15	3.94E-11	0.176	0.406
MYL12B	2	-0.2681204	8.82E-12	1.61E-07	0.494	0.678
RNASET2	2	-0.2682224	4.51E-18	8.23E-14	0.157	0.41
COMMD6	2	-0.2682606	4.20E-23	7.67E-19	0.994	1
CD8B	2	-0.2684286	8.45E-24	1.54E-19	0.055	0.308
AFF3	2	-0.2686983	2.60E-29	4.75E-25	0.008	0.259
JUN	2	-0.268875	5.02E-12	9.16E-08	0.144	0.333
SERBP1	2	-0.2694244	1.99E-13	3.63E-09	0.686	0.848
SMIM19	2	-0.2694498	4.50E-14	8.21E-10	0.299	0.531
DMKN	2	-0.26963	3.17E-15	5.78E-11	0.273	0.527
PLA2G16	2	-0.2705709	4.21E-15	7.68E-11	0.199	0.427
RPL22	2	-0.2707016	5.82E-31	1.06E-26	1	1
PRKAR1A	2	-0.2710758	1.11E-06	0.0202915	0.407	0.552
CD302	2	-0.2718616	3.47E-24	6.33E-20	0.064	0.326
PIGT	2	-0.2718616	4.14E-21	7.55E-17	0.066	0.303
MSMO1	2	-0.2720055	6.73E-13	1.23E-08	0.127	0.319
RPS3	2	-0.2724101	2.28E-47	4.16E-43	1	1
SOX9	2	-0.2726114	5.45E-11	9.95E-07	0.564	0.73
RPL18A	2	-0.273067	2.36E-43	4.31E-39	1	1
CCDC8	2	-0.2737284	4.72E-25	8.61E-21	0.032	0.275
RBMX	2	-0.2744403	2.14E-10	3.90E-06	0.528	0.667
MIR99AHG	2	-0.2744709	9.10E-27	1.66E-22	0.044	0.315
DSTN	2	-0.2744967	8.23E-19	1.50E-14	0.992	0.998
PAM	2	-0.2759718	4.17E-16	7.61E-12	0.324	0.594
VAMP5	2	-0.2773283	1.48E-14	2.70E-10	0.271	0.508
RARRES3	2	-0.2775041	2.39E-27	4.36E-23	0.032	0.296
COL6A1	2	-0.2775773	8.36E-14	1.53E-09	0.225	0.45
TCEA2	2	-0.2778436	2.18E-16	3.97E-12	0.206	0.455
BEX2	2	-0.2778436	2.33E-16	4.26E-12	0.201	0.457
RPL37A	2	-0.278103	6.11E-51	1.11E-46	1	1
HSPA8	2	-0.2783177	8.18E-11	1.49E-06	0.333	0.529
SCPEP1	2	-0.2784869	3.88E-21	7.07E-17	0.087	0.338
MYDGF	2	-0.2792303	3.01E-11	5.49E-07	0.642	0.788
OS9	2	-0.2794073	2.80E-13	5.11E-09	0.273	0.485
IFI27L2	2	-0.2794644	5.25E-17	9.58E-13	0.733	0.886

EBPL	2	-0.2816243	1.16E-14	2.11E-10	0.248	0.48
NAA38	2	-0.2817892	1.77E-11	3.24E-07	0.434	0.615
CCPG1	2	-0.2819128	6.92E-13	1.26E-08	0.271	0.492
LINC01503	2	-0.2823229	6.46E-11	1.18E-06	0.191	0.375
RPL14	2	-0.2830638	3.02E-42	5.50E-38	1	1
QARS	2	-0.2832985	3.82E-22	6.97E-18	0.083	0.343
SOCS3	2	-0.2837342	8.94E-17	1.63E-12	0.104	0.326
GSTP1	2	-0.2838535	2.70E-15	4.92E-11	0.934	0.967
GREM1	2	-0.2841132	2.67E-17	4.87E-13	0.006	0.159
EIF3J-AS1	2	-0.2841986	2.41E-19	4.40E-15	0.146	0.406
IDI1	2	-0.2843161	3.67E-16	6.69E-12	0.125	0.347
CHAD	2	-0.2844743	7.05E-27	1.29E-22	0.008	0.24
NFIC	2	-0.2852363	4.16E-16	7.59E-12	0.182	0.422
HOXC10	2	-0.2857399	2.44E-13	4.45E-09	0.485	0.704
SERTAD4-AS1	2	-0.2858758	1.98E-25	3.61E-21	0.044	0.303
DDOST	2	-0.2874131	5.06E-14	9.23E-10	0.256	0.478
ARL6IP4	2	-0.2878753	1.48E-18	2.70E-14	0.805	0.923
SARAF	2	-0.2882064	7.09E-14	1.29E-09	0.519	0.702
ETFB	2	-0.2885309	1.76E-14	3.20E-10	0.316	0.543
LTA4H	2	-0.2889394	7.78E-12	1.42E-07	0.373	0.562
IFITM1	2	-0.2890169	1.05E-07	0.00190721	0.311	0.457
PDGFRA	2	-0.2891068	1.16E-23	2.12E-19	0.07	0.338
SUMF2	2	-0.289392	4.01E-18	7.33E-14	0.22	0.503
CRYAB	2	-0.2893952	1.23E-10	2.24E-06	0.648	0.807
RPS29	2	-0.2899509	5.15E-50	9.40E-46	1	1
PDIA4	2	-0.2915875	4.37E-18	7.98E-14	0.144	0.392
CSDE1	2	-0.2916232	2.63E-14	4.80E-10	0.581	0.788
MKX	2	-0.2920283	2.50E-20	4.57E-16	0.097	0.347
TMED3	2	-0.2925005	1.10E-14	2.00E-10	0.307	0.538
IER2	2	-0.292561	1.31E-13	2.39E-09	0.239	0.466
DPP7	2	-0.2927852	8.01E-14	1.46E-09	0.398	0.636
SLC25A3	2	-0.2938575	2.95E-19	5.39E-15	0.864	0.932
LCN2	2	-0.2940438	3.44E-07	0.00627223	0.337	0.503
CHPT1	2	-0.2941171	9.46E-15	1.73E-10	0.443	0.671
SERPING1	2	-0.2949223	9.71E-14	1.77E-09	0.32	0.557
DUT	2	-0.2951592	3.26E-15	5.95E-11	0.441	0.66
OXA1L	2	-0.2954285	3.67E-12	6.70E-08	0.375	0.569
SGCB	2	-0.2963403	1.81E-17	3.29E-13	0.229	0.49
RPS4Y1	2	-0.2963505	4.34E-24	7.91E-20	0.983	0.998

PIM3	2	-0.2970041	9.92E-14	1.81E-09	0.288	0.517
AC005152.3	2	-0.2973214	3.38E-28	6.17E-24	0.023	0.282
RPL13	2	-0.2977996	2.38E-57	4.35E-53	1	1
SSR1	2	-0.2992361	1.23E-14	2.24E-10	0.311	0.555
NMRK1	2	-0.2992541	2.55E-17	4.65E-13	0.22	0.471
SLC1A5	2	-0.2998857	2.88E-24	5.25E-20	0.074	0.347
RPL15	2	-0.2999974	4.97E-62	9.07E-58	1	1
UQCRH	2	-0.3001163	2.09E-21	3.82E-17	0.939	0.979
ANGPTL5	2	-0.3007454	3.78E-22	6.90E-18	0.089	0.347
RPS23	2	-0.3024428	1.06E-50	1.93E-46	1	1
DDIT4	2	-0.3026566	4.90E-10	8.94E-06	0.32	0.517
TUBB2B	2	-0.3029909	1.29E-09	2.36E-05	0.316	0.494
PRKCSH	2	-0.3031614	1.99E-18	3.63E-14	0.214	0.48
ABHD14A	2	-0.3039037	2.49E-24	4.54E-20	0.068	0.331
BEX4	2	-0.3055363	3.72E-15	6.80E-11	0.371	0.613
TCEAL2	2	-0.3059728	1.06E-16	1.93E-12	0.282	0.543
BBX	2	-0.3066589	7.25E-18	1.32E-13	0.148	0.392
HADHA	2	-0.3067528	6.50E-18	1.19E-13	0.239	0.497
CALM1	2	-0.3078318	6.05E-13	1.10E-08	0.383	0.601
CANX	2	-0.3086265	3.67E-17	6.70E-13	0.504	0.748
TMED2	2	-0.3105267	3.66E-17	6.68E-13	0.678	0.879
CFH	2	-0.3112569	3.30E-09	6.02E-05	0.121	0.273
RBM3	2	-0.3113561	1.38E-14	2.51E-10	0.354	0.578
RPS18	2	-0.3117426	1.42E-54	2.59E-50	1	1
RPS9	2	-0.311971	3.19E-57	5.83E-53	1	1
MPP6	2	-0.3128516	1.25E-22	2.27E-18	0.146	0.434
EIF3E	2	-0.3131509	1.08E-20	1.97E-16	0.998	1
NME4	2	-0.3143588	4.47E-17	8.15E-13	0.222	0.476
RPL6	2	-0.3149379	2.85E-43	5.20E-39	1	1
NUPR1	2	-0.3158573	3.80E-16	6.94E-12	0.932	0.984
OAF	2	-0.3159221	4.18E-25	7.62E-21	0.072	0.347
SEP1	2	-0.3163641	9.40E-23	1.72E-18	0.089	0.354
TOMM7	2	-0.3165216	1.35E-45	2.47E-41	1	1
RCN1	2	-0.317033	3.54E-15	6.46E-11	0.242	0.471
NINJ1	2	-0.3172529	5.37E-17	9.80E-13	0.426	0.685
HOXB8	2	-0.3174429	1.63E-28	2.97E-24	0.034	0.308
MEG3	2	-0.31756	3.16E-14	5.76E-10	0.127	0.326
TMED9	2	-0.3176342	1.72E-14	3.13E-10	0.407	0.627
RCN2	2	-0.3176799	2.77E-15	5.05E-11	0.424	0.653

EIF3D	2	-0.319038	1.45E-14	2.64E-10	0.621	0.793
RP11-553L6.5	2	-0.3190394	4.06E-23	7.42E-19	0.117	0.396
RPL7A	2	-0.3191346	1.61E-50	2.94E-46	1	1
PRDX5	2	-0.3202392	2.91E-22	5.31E-18	0.822	0.953
RPS13	2	-0.3203354	8.79E-50	1.60E-45	1	1
C9orf3	2	-0.3210724	2.50E-19	4.55E-15	0.189	0.455
RPS7	2	-0.3215563	7.08E-48	1.29E-43	1	1
SLC44A1	2	-0.3219271	3.98E-21	7.26E-17	0.142	0.415
LY6E	2	-0.3219389	1.32E-17	2.40E-13	0.208	0.464
ASPN	2	-0.3224607	8.66E-14	1.58E-09	0.03	0.182
NKX3-2	2	-0.3228064	9.90E-25	1.81E-20	0.019	0.247
WWP2	2	-0.3228673	1.12E-12	2.05E-08	0.383	0.594
ERP29	2	-0.3237134	4.80E-18	8.77E-14	0.53	0.751
GSTA4	2	-0.3239613	3.97E-21	7.24E-17	0.172	0.445
LSP1	2	-0.3276624	3.09E-22	5.64E-18	0.059	0.305
ARPC1A	2	-0.3291965	1.92E-19	3.50E-15	0.356	0.615
CCDC88A	2	-0.3292959	4.41E-25	8.06E-21	0.087	0.373
CD320	2	-0.3293401	3.28E-18	5.98E-14	0.246	0.506
ENAH	2	-0.3294484	4.57E-22	8.35E-18	0.117	0.387
INSIG1	2	-0.3295689	7.30E-10	1.33E-05	0.288	0.457
P4HA1	2	-0.3315252	2.96E-14	5.41E-10	0.428	0.646
UQCRB	2	-0.332936	1.50E-20	2.74E-16	0.941	1
RPLP1	2	-0.3333125	2.92E-65	5.32E-61	1	1
RPL8	2	-0.3343618	4.83E-60	8.80E-56	1	1
RPS6	2	-0.3352183	1.85E-67	3.38E-63	1	1
RPS17	2	-0.3361916	3.39E-48	6.19E-44	1	1
RPS5	2	-0.336268	1.00E-55	1.83E-51	1	1
EIF2S3	2	-0.3364101	4.12E-15	7.51E-11	0.398	0.625
EFNA1	2	-0.3364378	3.62E-20	6.60E-16	0.172	0.445
PLPP1	2	-0.3371084	1.01E-24	1.84E-20	0.066	0.331
KDELR3	2	-0.3378106	1.64E-24	3.00E-20	0.068	0.333
GLTSCR2	2	-0.3378762	6.09E-21	1.11E-16	0.824	0.951
RPS27A	2	-0.3406053	3.37E-65	6.16E-61	1	1
C1S	2	-0.34149	3.83E-19	6.99E-15	0.386	0.664
TCEA3	2	-0.3429302	1.17E-19	2.13E-15	0.184	0.443
CD63	2	-0.3447511	9.03E-21	1.65E-16	0.996	1
CCDC102B	2	-0.3447978	4.04E-32	7.37E-28	0.03	0.326
COL5A2	2	-0.3448921	4.45E-17	8.13E-13	0.142	0.38
FIS1	2	-0.3456162	8.34E-20	1.52E-15	0.456	0.72

ILF3-AS1	2	-0.3458422	7.44E-24	1.36E-19	0.155	0.452
LUC7L3	2	-0.3464721	1.28E-21	2.34E-17	0.225	0.517
PPP1R3C	2	-0.3464843	1.96E-15	3.58E-11	0.25	0.483
TUBB	2	-0.3470279	1.81E-18	3.31E-14	0.672	0.86
RPS4X	2	-0.3494147	4.96E-65	9.05E-61	1	1
EIF2A	2	-0.3499024	4.45E-16	8.12E-12	0.559	0.741
RP11-983P16.4	2	-0.3505013	5.35E-25	9.76E-21	0.174	0.49
RPN2	2	-0.3512623	1.34E-28	2.45E-24	0.057	0.347
TXNIP	2	-0.3519267	2.73E-37	4.99E-33	0.015	0.331
NOVA1	2	-0.3521895	1.07E-12	1.96E-08	0.748	0.879
TMEM230	2	-0.3527449	1.25E-19	2.28E-15	0.517	0.751
PNKD	2	-0.3539856	3.83E-27	6.99E-23	0.176	0.508
S100A13	2	-0.3560977	2.81E-34	5.13E-30	0.996	1
SRSF5	2	-0.3568241	3.02E-17	5.52E-13	0.453	0.676
TRPS1	2	-0.3572899	1.22E-20	2.22E-16	0.254	0.545
RPL23	2	-0.357707	1.79E-45	3.26E-41	0.998	1
NUCKS1	2	-0.357791	1.48E-18	2.70E-14	0.432	0.683
C1orf21	2	-0.3582312	4.11E-19	7.49E-15	0.314	0.576
GRN	2	-0.3588999	5.56E-27	1.01E-22	0.093	0.396
PRCP	2	-0.3604931	4.56E-31	8.32E-27	0.059	0.368
ST13	2	-0.3610276	1.69E-27	3.09E-23	0.909	0.977
ATP5A1	2	-0.3622436	2.35E-21	4.29E-17	0.333	0.615
NUCB1	2	-0.3624933	1.69E-20	3.08E-16	0.318	0.59
C7orf73	2	-0.3636723	1.43E-21	2.60E-17	0.578	0.821
FKBP10	2	-0.3636872	1.02E-21	1.86E-17	0.191	0.483
RPL5	2	-0.3639105	4.86E-50	8.86E-46	1	1
SOD1	2	-0.3639498	3.89E-26	7.10E-22	0.917	0.974
CMB9-22P13.1	2	-0.363969	7.08E-21	1.29E-16	0.273	0.559
CPE	2	-0.3639781	2.44E-18	4.46E-14	0.487	0.727
PNISR	2	-0.3657816	1.20E-20	2.18E-16	0.485	0.737
HMGB1	2	-0.3666411	1.33E-22	2.43E-18	0.633	0.86
RPL13A	2	-0.367992	2.95E-80	5.38E-76	1	1
HMGN3	2	-0.368014	4.13E-24	7.54E-20	0.21	0.52
H1FX	2	-0.3689791	4.64E-23	8.46E-19	0.1	0.371
DST	2	-0.3692179	1.55E-20	2.82E-16	0.335	0.615
FHL1	2	-0.3694443	7.65E-20	1.40E-15	0.229	0.501
TMEM98	2	-0.3702636	2.49E-31	4.54E-27	0.1	0.434
PTPRD-AS1	2	-0.3717148	2.15E-25	3.92E-21	0.169	0.483
PGAM1	2	-0.3735887	1.94E-23	3.54E-19	0.614	0.869

TSPAN3	2	-0.3737667	7.96E-22	1.45E-17	0.337	0.629
MYLK	2	-0.3740275	3.04E-23	5.55E-19	0.089	0.354
MOXD1	2	-0.3754018	1.53E-20	2.79E-16	0.161	0.431
MYL6	2	-0.3771222	3.45E-24	6.29E-20	0.856	0.939
TSPAN4	2	-0.3773732	3.27E-25	5.97E-21	0.191	0.515
KDELR2	2	-0.3779578	1.30E-24	2.37E-20	0.758	0.909
DUSP1	2	-0.3789689	6.16E-19	1.12E-14	0.328	0.604
PPIB	2	-0.3805983	6.05E-18	1.10E-13	0.831	0.928
BTF3	2	-0.3818247	4.78E-43	8.72E-39	0.996	1
BHLHE41	2	-0.3822863	5.03E-26	9.18E-22	0.131	0.434
RPL30	2	-0.3823111	3.54E-64	6.45E-60	1	1
CREG1	2	-0.3824967	4.86E-22	8.87E-18	0.356	0.639
GOLIM4	2	-0.3835622	6.33E-14	1.15E-09	0.273	0.503
AHNAK	2	-0.3856284	3.04E-24	5.55E-20	0.237	0.548
ENPP1	2	-0.3890013	2.24E-30	4.09E-26	0.076	0.394
HERPUD1	2	-0.3891672	2.46E-24	4.49E-20	0.248	0.557
PPIA	2	-0.3895594	7.34E-30	1.34E-25	0.905	0.988
FXYD1	2	-0.3898664	2.69E-18	4.91E-14	0.146	0.385
HSPB1	2	-0.3904996	3.49E-20	6.37E-16	0.521	0.783
POLR2L	2	-0.3914057	6.98E-24	1.27E-19	0.701	0.909
RPL19	2	-0.3923599	1.90E-81	3.47E-77	1	1
DBP	2	-0.394765	3.48E-31	6.35E-27	0.081	0.406
ARL2BP	2	-0.3954062	7.49E-24	1.37E-19	0.297	0.608
MTDH	2	-0.395672	9.22E-22	1.68E-17	0.439	0.69
CYBRD1	2	-0.3959872	1.85E-23	3.37E-19	0.367	0.657
PLD3	2	-0.3980928	3.19E-28	5.82E-24	0.197	0.543
LITAF	2	-0.3982769	4.60E-29	8.40E-25	0.265	0.618
RPL10	2	-0.4008391	7.18E-89	1.31E-84	1	1
PPT1	2	-0.4012691	4.10E-28	7.49E-24	0.184	0.515
RPL18	2	-0.4027014	1.43E-63	2.61E-59	1	1
RPL9	2	-0.4029267	5.81E-74	1.06E-69	1	1
SERF2	2	-0.4039177	2.00E-41	3.66E-37	0.998	1
CTGF	2	-0.405677	2.96E-18	5.40E-14	0.176	0.429
TCEAL8	2	-0.4059066	7.07E-26	1.29E-21	0.47	0.762
HIST1H4C	2	-0.4059398	1.49E-22	2.72E-18	0.358	0.655
HEXB	2	-0.4067931	3.21E-27	5.85E-23	0.212	0.541
LRPAP1	2	-0.406936	2.27E-23	4.14E-19	0.265	0.559
RP5-1097P24.1	2	-0.4076552	4.46E-36	8.13E-32	0.064	0.413
COL9A2	2	-0.407762	6.92E-26	1.26E-21	0.11	0.41

RPLP2	2	-0.4097679	1.23E-78	2.25E-74	1	1
NDUFA11	2	-0.4115495	3.25E-28	5.93E-24	0.769	0.925
CRIP2	2	-0.412618	5.27E-26	9.61E-22	0.203	0.517
ZBTB20	2	-0.4159645	1.25E-32	2.29E-28	0.131	0.49
ATRNL1	2	-0.4160877	2.89E-32	5.28E-28	0.081	0.41
AC003090.1	2	-0.416952	1.11E-42	2.02E-38	0.028	0.396
NAP1L1	2	-0.4176545	3.54E-32	6.47E-28	0.898	0.979
VKORC1	2	-0.4202691	2.57E-29	4.68E-25	0.862	0.96
AP2M1	2	-0.4202858	1.85E-25	3.38E-21	0.316	0.627
RPS2	2	-0.4218868	4.31E-79	7.86E-75	1	1
PRUNE2	2	-0.4239561	4.86E-27	8.87E-23	0.165	0.485
SEPP1	2	-0.4250951	5.96E-34	1.09E-29	0.144	0.52
RPS16	2	-0.4254661	2.30E-76	4.20E-72	1	1
DNM3OS	2	-0.4298518	6.18E-31	1.13E-26	0.104	0.438
SPINT2	2	-0.4298563	5.69E-27	1.04E-22	0.208	0.531
CTSK	2	-0.4303056	1.00E-34	1.82E-30	0.068	0.408
SEC11A	2	-0.4307542	6.37E-36	1.16E-31	0.792	0.928
ATPIF1	2	-0.4323368	7.84E-28	1.43E-23	0.445	0.769
S100A1	2	-0.4338271	1.29E-37	2.35E-33	0.996	1
ARHGAP29	2	-0.4340895	1.24E-23	2.26E-19	0.222	0.517
PSAP	2	-0.4389439	8.02E-33	1.46E-28	0.761	0.944
MORF4L2	2	-0.439485	1.22E-26	2.23E-22	0.411	0.704
HDLBP	2	-0.4397916	5.60E-26	1.02E-21	0.193	0.494
HLA-B	2	-0.4402208	2.95E-22	5.38E-18	0.653	0.86
CCNG1	2	-0.4406362	1.08E-29	1.97E-25	0.225	0.569
GNAS	2	-0.4429668	1.72E-28	3.14E-24	0.398	0.706
TPM2	2	-0.4430511	1.96E-28	3.58E-24	0.309	0.641
TPD52L1	2	-0.4434173	1.66E-34	3.04E-30	0.169	0.55
LDHB	2	-0.4435536	1.06E-30	1.93E-26	0.631	0.879
CUTA	2	-0.4444525	7.15E-32	1.31E-27	0.602	0.883
CHRDL2	2	-0.4459027	6.91E-21	1.26E-16	0.072	0.308
PKM	2	-0.4498468	7.30E-33	1.33E-28	0.818	0.946
RPL34	2	-0.4513345	1.37E-91	2.50E-87	1	1
CALR	2	-0.4520767	6.96E-21	1.27E-16	0.428	0.692
RPSA	2	-0.4549688	5.67E-58	1.03E-53	1	1
RPL23A	2	-0.4551426	6.19E-88	1.13E-83	1	1
ATRAID	2	-0.4581102	2.61E-30	4.76E-26	0.636	0.897
BRK1	2	-0.4582781	3.32E-38	6.05E-34	0.691	0.942
RNF130	2	-0.4596087	4.10E-29	7.47E-25	0.331	0.662

ARL6IP5	2	-0.4622727	4.75E-35	8.67E-31	0.333	0.713
RABAC1	2	-0.4623925	5.90E-37	1.08E-32	0.693	0.932
HSP90AB1	2	-0.4626657	2.31E-36	4.22E-32	0.919	0.984
PDE4DIP	2	-0.4637818	2.38E-36	4.35E-32	0.186	0.569
COL6A2	2	-0.4671839	4.87E-25	8.88E-21	0.309	0.625
PDIA3	2	-0.4713114	1.15E-25	2.11E-21	0.322	0.618
SCRG1	2	-0.4716224	1.11E-27	2.02E-23	0.913	0.991
EEF1B2	2	-0.4744831	3.85E-46	7.02E-42	0.987	0.995
RPS3A	2	-0.4746271	3.93E-96	7.16E-92	1	1
JUNB	2	-0.4753528	7.47E-25	1.36E-20	0.481	0.788
HMGN2	2	-0.4864316	5.96E-36	1.09E-31	0.758	0.963
ERGIC3	2	-0.4904538	1.30E-34	2.37E-30	0.612	0.865
CTSD	2	-0.5019952	2.87E-29	5.24E-25	0.682	0.918
SSR2	2	-0.5033277	2.72E-40	4.97E-36	0.695	0.937
MAGED1	2	-0.5036826	2.15E-27	3.92E-23	0.038	0.305
TMEM59	2	-0.5041453	5.70E-42	1.04E-37	0.949	0.988
ACTG1	2	-0.5050746	1.45E-32	2.65E-28	0.924	0.984
MT-ND5	2	-0.5059133	2.00E-22	3.64E-18	0.419	0.676
TMED10	2	-0.5105908	1.55E-36	2.83E-32	0.729	0.93
INHBA	2	-0.5115048	8.56E-32	1.56E-27	0.14	0.499
MFGE8	2	-0.5138684	9.05E-30	1.65E-25	0.608	0.883
SSPN	2	-0.5152639	1.43E-48	2.62E-44	0.068	0.501
PTRF	2	-0.5154721	1.52E-39	2.77E-35	0.186	0.594
FAM46A	2	-0.5173195	2.78E-22	5.08E-18	0.364	0.625
CHID1	2	-0.5173334	1.81E-37	3.30E-33	0.244	0.634
ITM2B	2	-0.5201782	4.02E-37	7.34E-33	0.917	0.986
CXXC5	2	-0.5209299	1.23E-49	2.24E-45	0.068	0.51
COL11A2	2	-0.5223327	1.64E-35	2.99E-31	0.218	0.601
GSN	2	-0.52268	2.76E-39	5.04E-35	0.227	0.632
BSG	2	-0.5227969	1.12E-36	2.04E-32	0.388	0.76
YBX1	2	-0.5254782	1.77E-57	3.23E-53	0.958	1
SDF4	2	-0.5263715	3.12E-38	5.70E-34	0.379	0.755
SH3BGRL	2	-0.5274537	2.62E-42	4.78E-38	0.216	0.641
SSR3	2	-0.529007	2.93E-41	5.35E-37	0.752	0.953
SPON2	2	-0.5310745	1.53E-43	2.79E-39	0.055	0.445
PLXDC2	2	-0.5357911	1.44E-38	2.62E-34	0.256	0.655
VCAN	2	-0.537223	1.76E-34	3.21E-30	0.106	0.462
NUCB2	2	-0.5387063	4.98E-39	9.08E-35	0.186	0.583
SNAI2	2	-0.5401717	7.77E-27	1.42E-22	0.305	0.615

RPL3	2	-0.5423066	1.78E-95	3.25E-91	1	1
BGN	2	-0.5450291	1.90E-42	3.47E-38	0.934	0.998
HSP90B1	2	-0.5464909	1.82E-27	3.32E-23	0.608	0.834
RPL7	2	-0.5470826	3.10E-88	5.66E-84	1	1
EIF4B	2	-0.5472539	9.02E-39	1.65E-34	0.621	0.909
TTC3	2	-0.5492188	4.54E-37	8.29E-33	0.403	0.765
TPT1	2	-0.5514922	1.31E-96	2.39E-92	1	1
FOS	2	-0.5535789	6.07E-37	1.11E-32	0.407	0.765
FCGRT	2	-0.5538079	2.73E-37	4.98E-33	0.301	0.669
LUM	2	-0.5542566	3.04E-33	5.54E-29	1	1
FTL	2	-0.5543044	4.17E-70	7.60E-66	1	1
PPIC	2	-0.5570081	4.92E-34	8.98E-30	0.231	0.592
SLC14A1	2	-0.5574863	5.96E-42	1.09E-37	0.163	0.571
PRDX4	2	-0.55889	1.21E-35	2.20E-31	0.553	0.814
PCBP2	2	-0.5590824	3.26E-52	5.94E-48	0.775	0.967
SSR4	2	-0.5605316	1.40E-44	2.55E-40	0.888	0.986
TSPO	2	-0.5615039	1.10E-48	2.01E-44	0.623	0.932
NFIA	2	-0.5626031	4.36E-53	7.95E-49	0.083	0.543
MDFI	2	-0.5688353	1.20E-36	2.20E-32	0.248	0.625
GAS6	2	-0.5688726	8.82E-51	1.61E-46	0.083	0.536
RPLP0	2	-0.5845059	2.92E-96	5.33E-92	1	1
DDR2	2	-0.5862848	2.52E-42	4.60E-38	0.195	0.604
MIA	2	-0.5869974	2.18E-36	3.98E-32	0.962	1
H3F3A	2	-0.5880883	3.96E-60	7.22E-56	0.907	0.998
MT-ND4	2	-0.5917959	5.14E-45	9.38E-41	0.953	0.988
CYR61	2	-0.5937292	4.39E-32	8.02E-28	0.161	0.52
PCOLCE2	2	-0.5938847	3.62E-41	6.60E-37	0.744	0.958
S100A4	2	-0.595324	3.96E-29	7.22E-25	0.547	0.851
XAGE2	2	-0.5991641	2.01E-23	3.67E-19	0.004	0.203
HLA-A	2	-0.6069016	9.83E-44	1.79E-39	0.754	0.965
THY1	2	-0.6074341	1.97E-42	3.60E-38	0.138	0.545
CHI3L1	2	-0.6090889	1.61E-19	2.94E-15	0.886	0.97
PABPC1	2	-0.6121843	3.54E-50	6.47E-46	0.735	0.942
BAMBI	2	-0.6163636	4.14E-29	7.55E-25	0.324	0.646
ID2	2	-0.6174955	4.31E-30	7.86E-26	0.324	0.676
HTRA1	2	-0.6286007	7.98E-57	1.46E-52	0.487	0.921
HLA-C	2	-0.6286203	5.00E-44	9.12E-40	0.725	0.949
CTSC	2	-0.6294067	1.48E-30	2.70E-26	0.091	0.408
EID1	2	-0.6342777	1.05E-59	1.91E-55	0.797	0.97

MSMP	2	-0.6358211	5.90E-14	1.08E-09	0.193	0.438
RPL10A	2	-0.6368677	1.08E-106	1.97E-102	1	1
RPS8	2	-0.6388557	1.53E-112	2.79E-108	1	1
PTMS	2	-0.6413757	1.34E-49	2.45E-45	0.369	0.8
SDC2	2	-0.6436141	1.58E-50	2.89E-46	0.54	0.911
SOD3	2	-0.6465015	1.41E-31	2.58E-27	0.85	0.97
ITM2A	2	-0.6469853	2.84E-42	5.19E-38	0.244	0.657
ID4	2	-0.6489073	7.46E-39	1.36E-34	0.278	0.674
RPS20	2	-0.6554801	2.04E-99	3.73E-95	1	1
NPC2	2	-0.6703496	8.88E-75	1.62E-70	0.936	1
PEBP1	2	-0.6774738	3.91E-59	7.14E-55	0.523	0.918
EIF3L	2	-0.6817044	3.01E-65	5.49E-61	0.657	0.963
CCNI	2	-0.6831833	8.16E-84	1.49E-79	0.981	1
TCF4	2	-0.6861296	8.88E-52	1.62E-47	0.186	0.648
NNMT	2	-0.6862848	9.42E-40	1.72E-35	0.807	0.951
RPS11	2	-0.689535	2.10E-98	3.82E-94	0.985	1
VIM	2	-0.692695	4.84E-66	8.83E-62	0.998	1
HNRNPA1	2	-0.6942557	3.57E-70	6.52E-66	0.975	1
CCDC80	2	-0.7004727	2.23E-48	4.07E-44	0.72	0.984
YBX3	2	-0.7043642	3.54E-60	6.46E-56	0.572	0.953
CP	2	-0.7130167	6.36E-47	1.16E-42	0.485	0.837
MALAT1	2	-0.7140551	7.47E-53	1.36E-48	1	1
SLC40A1	2	-0.7209901	3.19E-63	5.83E-59	0.036	0.531
SERPINA5	2	-0.7280979	1.14E-59	2.07E-55	0.189	0.692
IGFBP6	2	-0.7316481	1.31E-29	2.40E-25	0.39	0.706
RPL4	2	-0.7323325	4.24E-99	7.74E-95	0.996	1
IGFBP7	2	-0.7377807	2.61E-49	4.76E-45	0.585	0.937
CD9	2	-0.742956	2.19E-56	4.00E-52	0.261	0.744
EIF3H	2	-0.7444079	1.01E-88	1.84E-84	0.947	1
FGFBP2	2	-0.7479289	1.25E-29	2.28E-25	0.883	0.981
PDIA6	2	-0.7537251	4.82E-58	8.79E-54	0.35	0.809
RHOBTB3	2	-0.7613097	3.02E-57	5.52E-53	0.307	0.774
CITED2	2	-0.7796683	2.70E-52	4.93E-48	0.138	0.599
GPX3	2	-0.786684	5.25E-63	9.58E-59	0.841	0.995
FXYD6	2	-0.7897604	1.22E-72	2.22E-68	0.498	0.93
CRTAP	2	-0.7945115	3.09E-74	5.64E-70	0.405	0.883
FTH1	2	-0.7999439	2.52E-85	4.60E-81	1	1
CRISPLD1	2	-0.8083897	6.22E-53	1.13E-48	0.011	0.427
COL11A1	2	-0.8153526	3.10E-67	5.66E-63	0.297	0.841

MT-CO1	2	-0.8189419	4.27E-63	7.80E-59	0.956	0.993
ZFP36L2	2	-0.8247092	1.78E-48	3.24E-44	0.121	0.557
CEBPD	2	-0.8261817	2.52E-52	4.59E-48	0.619	0.911
FBXO2	2	-0.829392	9.62E-77	1.76E-72	0.426	0.902
ID1	2	-0.8597808	2.58E-40	4.70E-36	0.347	0.753
CST3	2	-0.8627552	2.12E-82	3.87E-78	0.947	0.998
MT-CO3	2	-0.8629122	3.14E-68	5.72E-64	0.871	0.981
PRELP	2	-0.8653003	2.79E-68	5.08E-64	0.771	0.998
ITM2C	2	-0.8677685	6.87E-75	1.25E-70	0.155	0.727
APOD	2	-0.8696105	1.44E-44	2.63E-40	0.174	0.594
MT-CO2	2	-0.8904597	5.65E-70	1.03E-65	0.919	0.993
NGFRAP1	2	-0.8916276	1.93E-80	3.52E-76	0.572	0.956
ISLR	2	-0.9021026	2.77E-86	5.06E-82	0.07	0.697
SPARC	2	-0.9025892	5.56E-70	1.01E-65	0.195	0.758
CD99	2	-0.9140385	2.34E-90	4.26E-86	0.727	0.984
MT-ND1	2	-0.9177936	1.33E-63	2.43E-59	0.419	0.869
FMOD	2	-0.9243699	2.45E-83	4.47E-79	0.627	0.979
EEF2	2	-0.9256302	3.39E-89	6.19E-85	0.962	0.998
S100B	2	-0.9377389	4.73E-75	8.63E-71	0.436	0.925
EEF1A1	2	-0.9435162	1.64E-134	2.99E-130	1	1
MT-ND3	2	-0.9480462	1.35E-70	2.46E-66	0.485	0.925
P4HB	2	-0.9545927	9.39E-86	1.71E-81	0.508	0.967
MGP	2	-0.9731874	4.51E-61	8.23E-57	0.989	1
DCN	2	-0.9742361	3.68E-97	6.72E-93	1	1
MT-ATP6	2	-1.0101585	1.39E-76	2.54E-72	0.71	0.97
C2orf40	2	-1.0217513	4.33E-73	7.90E-69	0.928	1
MT-ND2	2	-1.022607	9.24E-74	1.69E-69	0.411	0.9
RBP4	2	-1.0271132	3.61E-72	6.59E-68	0.413	0.893
MT-CYB	2	-1.0337917	4.53E-74	8.27E-70	0.508	0.937
ACAN	2	-1.0780419	8.75E-99	1.60E-94	0.752	0.998
NEAT1	2	-1.0861065	3.07E-94	5.60E-90	0.972	1
B2M	2	-1.0916728	2.02E-110	3.68E-106	0.96	1
PAX1	2	-1.1116513	4.38E-101	8.00E-97	0.28	0.9
TSC22D1	2	-1.1474929	1.08E-61	1.98E-57	0.5	0.9
COL9A3	2	-1.1539568	2.29E-77	4.18E-73	0.227	0.816
ADIRF	2	-1.1560581	7.92E-84	1.45E-79	0.244	0.851
ID3	2	-1.1776362	9.92E-74	1.81E-69	0.214	0.788
PLAC9	2	-1.302882	3.14E-105	5.72E-101	0.511	0.981
CAPS	2	-1.3443426	2.25E-99	4.11E-95	0.468	0.96

LECT1	2	-1.3599112	6.65E-99	1.21E-94	0.394	0.953
C2orf82	2	-1.3790356	1.93E-97	3.52E-93	0.953	0.998
SPTSSB	2	-1.5169783	9.85E-100	1.80E-95	0.021	0.706
COL2A1	2	-1.5741714	5.18E-103	9.45E-99	0.254	0.921
FRZB	2	-1.582901	1.02E-67	1.85E-63	0.233	0.779
SLPI	2	-1.7245219	1.42E-66	2.60E-62	0.824	1
OGN	2	-1.8141119	2.47E-117	4.51E-113	0.606	0.995
CLEC3A	2	-1.9338016	1.90E-136	3.46E-132	0.112	0.939
**gene**	**cluster**	**avg_logFC**	**p_val**	**p_val_adj**	**pct.1**	**pct.2**
SPP1	3	2.66044525	2.21E-72	4.03E-68	0.86	0.157
PRG4	3	2.29986475	6.25E-68	1.14E-63	0.722	0.045
CTSL	3	1.46477471	1.96E-58	3.57E-54	0.874	0.422
IGFBP3	3	1.31031804	1.75E-52	3.20E-48	0.625	0.052
UPP1	3	1.2980046	5.92E-77	1.08E-72	0.853	0.185
PTGS2	3	1.22097911	9.62E-59	1.76E-54	0.736	0.132
MT2A	3	1.22090635	3.23E-68	5.90E-64	1	1
HMGA1	3	1.22059159	2.68E-53	4.89E-49	0.838	0.408
CCL20	3	1.19956526	1.25E-15	2.29E-11	0.268	0.035
C11orf96	3	1.09283593	6.57E-52	1.20E-47	0.684	0.105
BMP2	3	1.08187066	8.32E-51	1.52E-46	0.691	0.118
NRP2	3	1.00510517	1.14E-37	2.09E-33	0.679	0.247
TIMP1	3	0.99727054	1.96E-09	3.58E-05	0.993	0.986
VEGFA	3	0.99680184	3.53E-52	6.45E-48	0.827	0.366
CXCL8	3	0.97246262	4.93E-27	9.00E-23	0.409	0.042
WTAP	3	0.95464659	3.62E-44	6.61E-40	0.971	0.774
TFPI2	3	0.9308656	3.19E-19	5.82E-15	0.276	0.017
ERO1A	3	0.90271578	1.22E-54	2.22E-50	0.798	0.275
COMP	3	0.90119392	2.32E-21	4.23E-17	0.929	0.798
CD55	3	0.89700532	3.47E-47	6.34E-43	0.919	0.54
G0S2	3	0.88151081	1.00E-11	1.83E-07	0.496	0.272
STK17A	3	0.87576874	2.66E-48	4.85E-44	0.701	0.181
MRPS6	3	0.84994541	3.32E-40	6.06E-36	0.967	0.878
TREM1	3	0.83524471	5.29E-51	9.64E-47	0.584	0.021
MIF	3	0.83445529	2.30E-77	4.19E-73	1	0.997
CCDC85B	3	0.82936925	3.84E-34	7.00E-30	0.895	0.666
RABGGTB	3	0.81528541	2.28E-53	4.15E-49	0.874	0.509
S100A2	3	0.81178566	1.90E-26	3.46E-22	0.646	0.293
C15orf48	3	0.79921955	2.08E-28	3.80E-24	0.615	0.185
TNFRSF12A	3	0.79119993	5.63E-38	1.03E-33	0.698	0.24

TM4SF1	3	0.7791973	3.25E-17	5.93E-13	0.734	0.516
TIMP3	3	0.77549194	3.98E-22	7.27E-18	0.67	0.362
SLC7A2	3	0.77495877	1.50E-36	2.74E-32	0.698	0.223
HILPDA	3	0.71487773	1.14E-17	2.09E-13	0.686	0.439
SNHG12	3	0.70895909	2.10E-34	3.84E-30	0.634	0.206
MT1X	3	0.69126756	1.58E-23	2.89E-19	1	1
SMOX	3	0.68998259	1.09E-23	1.99E-19	0.561	0.223
GSTO1	3	0.67868694	1.17E-37	2.13E-33	0.891	0.672
SLC2A1	3	0.67316553	8.79E-29	1.60E-24	0.527	0.146
PMP22	3	0.66887173	3.87E-36	7.05E-32	0.912	0.666
BZW1	3	0.66344628	3.20E-29	5.83E-25	0.836	0.554
BHLHE40	3	0.65181164	5.32E-25	9.70E-21	0.627	0.272
NDRG1	3	0.62543552	4.42E-33	8.06E-29	0.772	0.404
SLC3A2	3	0.61479648	4.90E-23	8.95E-19	0.532	0.202
PHLDA1	3	0.60964096	2.15E-24	3.92E-20	0.439	0.091
PPP1R15A	3	0.60459908	3.06E-26	5.59E-22	0.615	0.261
PPP3CA	3	0.59538242	1.22E-20	2.22E-16	0.869	0.655
HMGA2	3	0.5934033	7.68E-40	1.40E-35	0.487	0.021
ARF6	3	0.59092094	2.78E-32	5.07E-28	0.777	0.425
DNAJB9	3	0.5835939	5.06E-24	9.23E-20	0.81	0.568
IER3	3	0.58345664	8.43E-18	1.54E-13	0.691	0.362
PPP1CB	3	0.58294294	1.49E-22	2.72E-18	0.862	0.631
CCND1	3	0.58220871	3.44E-25	6.28E-21	0.513	0.153
ENO2	3	0.57890853	8.37E-28	1.53E-23	0.603	0.233
PGK1	3	0.57781983	9.54E-22	1.74E-17	0.905	0.76
TFPI	3	0.57334653	5.17E-27	9.43E-23	0.449	0.08
VAPA	3	0.57201427	7.52E-33	1.37E-28	0.969	0.847
SELK	3	0.56550202	7.75E-32	1.41E-27	0.936	0.763
PLAUR	3	0.5623788	2.65E-26	4.84E-22	0.594	0.23
CLEC2B	3	0.55400357	5.95E-12	1.09E-07	0.378	0.153
RELB	3	0.54680296	1.57E-25	2.87E-21	0.613	0.258
SGIP1	3	0.53246649	1.74E-28	3.17E-24	0.401	0.035
EIF4A2	3	0.53065188	1.54E-37	2.81E-33	0.998	0.962
TMEM38B	3	0.52706107	3.33E-25	6.08E-21	0.708	0.39
SLC5A3	3	0.52612869	5.13E-15	9.36E-11	0.632	0.376
MGST1	3	0.5223596	5.50E-17	1.00E-12	0.563	0.279
CTHRC1	3	0.517342	1.62E-19	2.95E-15	0.375	0.08
ISG20	3	0.51518324	1.21E-21	2.20E-17	0.694	0.404
RND3	3	0.51219049	2.29E-17	4.17E-13	0.366	0.091

ARPP19	3	0.50035029	1.90E-20	3.47E-16	0.66	0.425
GBE1	3	0.49923856	8.18E-17	1.49E-12	0.658	0.443
SOD2	3	0.49760328	1.06E-23	1.94E-19	1	0.993
SPRY2	3	0.49579545	1.49E-27	2.72E-23	0.444	0.07
CTNNB1	3	0.49473067	8.65E-12	1.58E-07	0.537	0.314
TMSB10	3	0.48098228	2.73E-21	4.98E-17	0.998	0.99
CSGALNACT2	3	0.4798956	4.36E-23	7.96E-19	0.461	0.122
EIF1	3	0.47723869	3.49E-44	6.37E-40	1	1
BCL2A1	3	0.47651445	1.82E-24	3.32E-20	0.302	0
GOLT1B	3	0.47429961	3.73E-22	6.80E-18	0.565	0.237
TRMT112	3	0.47073455	5.06E-25	9.23E-21	0.943	0.829
SERPINE2	3	0.46917071	6.97E-15	1.27E-10	1	0.99
IL11	3	0.46116661	4.12E-13	7.53E-09	0.173	0.003
RNF114	3	0.46038076	8.70E-16	1.59E-11	0.663	0.446
HLA-DRB1	3	0.46000988	1.03E-18	1.87E-14	0.485	0.188
ATP6V1G1	3	0.45990545	1.75E-22	3.20E-18	0.979	0.958
TNFAIP6	3	0.45989915	2.04E-18	3.72E-14	0.287	0.028
COX17	3	0.45925127	8.47E-25	1.55E-20	0.895	0.697
FOSL1	3	0.45615284	6.54E-24	1.19E-19	0.378	0.045
ABI3BP	3	0.45355813	1.25E-11	2.27E-07	0.546	0.341
ENO1	3	0.45080601	2.80E-14	5.10E-10	0.969	0.93
DDIT3	3	0.43992773	2.36E-16	4.30E-12	0.568	0.293
RNF145	3	0.43920829	2.27E-15	4.14E-11	0.511	0.244
FAM162A	3	0.4387719	1.05E-17	1.91E-13	0.884	0.749
PTP4A1	3	0.43865861	4.21E-16	7.68E-12	0.544	0.279
ARF4	3	0.43580428	1.85E-15	3.38E-11	0.786	0.589
SLC25A37	3	0.43570675	4.56E-23	8.32E-19	0.971	0.843
NT5E	3	0.4277796	6.26E-14	1.14E-09	0.537	0.293
TGFBI	3	0.42052124	3.07E-15	5.60E-11	0.42	0.164
PPP1R14C	3	0.41975914	1.16E-24	2.11E-20	0.401	0.059
ZNF593	3	0.41877422	3.71E-20	6.78E-16	0.634	0.314
RIPK2	3	0.41492595	1.28E-20	2.34E-16	0.489	0.167
TMEM45A	3	0.41449919	2.60E-10	4.75E-06	0.805	0.7
BTG3	3	0.41339986	1.86E-18	3.40E-14	0.513	0.209
PFDN2	3	0.40966405	2.84E-19	5.18E-15	0.698	0.425
DDIT4L	3	0.4087314	1.91E-11	3.48E-07	0.817	0.652
TGM2	3	0.4050828	7.25E-19	1.32E-14	0.62	0.314
FAP	3	0.40502784	7.03E-20	1.28E-15	0.435	0.122
DNAJB1	3	0.40327319	1.04E-08	0.00019031	0.399	0.216

VIMP	3	0.40202407	2.07E-14	3.78E-10	0.803	0.617
NRN1	3	0.40197895	1.26E-09	2.30E-05	0.601	0.394
SEC61G	3	0.40194601	1.61E-18	2.94E-14	0.998	0.99
GRB10	3	0.40156376	4.77E-22	8.70E-18	0.306	0.017
TVP23B	3	0.40132466	2.63E-16	4.80E-12	0.518	0.244
VDAC1	3	0.40006698	5.23E-17	9.54E-13	0.881	0.735
EIF1B	3	0.39549032	3.25E-17	5.93E-13	0.803	0.617
PTGES3	3	0.38903791	2.58E-20	4.71E-16	0.964	0.892
PPFIBP1	3	0.38712257	1.18E-13	2.15E-09	0.615	0.408
TNFAIP8	3	0.38590988	7.47E-18	1.36E-13	0.382	0.098
DNER	3	0.38508701	4.67E-13	8.52E-09	0.705	0.502
SERPINE1	3	0.38231722	1.49E-16	2.71E-12	0.29	0.045
CGGBP1	3	0.38132588	1.73E-14	3.15E-10	0.568	0.328
IL32	3	0.37794722	1.14E-16	2.08E-12	0.496	0.192
BAALC	3	0.37400189	1.20E-07	0.00218466	0.556	0.436
GJA1	3	0.3728525	2.58E-19	4.71E-15	0.387	0.091
CRLF1	3	0.37227941	6.80E-15	1.24E-10	0.347	0.098
MMP14	3	0.37216798	3.90E-21	7.11E-17	0.363	0.059
C11orf31	3	0.37121519	1.28E-15	2.33E-11	0.805	0.638
PLCG2	3	0.37120729	3.16E-12	5.76E-08	0.238	0.045
SMS	3	0.36972952	9.10E-11	1.66E-06	0.672	0.516
EVI2A	3	0.36297961	4.67E-17	8.52E-13	0.437	0.146
TYMP	3	0.36066596	9.22E-13	1.68E-08	0.639	0.401
PPDPF	3	0.35955272	3.12E-18	5.69E-14	1	1
SPHK1	3	0.35651658	4.49E-18	8.19E-14	0.375	0.091
PLOD2	3	0.35582983	2.26E-13	4.13E-09	0.812	0.638
ADSS	3	0.35557944	7.62E-10	1.39E-05	0.599	0.439
CRTAC1	3	0.35479984	5.13E-12	9.36E-08	0.454	0.216
WDR45B	3	0.35292161	5.87E-15	1.07E-10	0.439	0.178
AKAP12	3	0.35255618	8.57E-15	1.56E-10	0.259	0.038
COX7A2	3	0.35214992	2.62E-21	4.78E-17	0.988	0.958
WDR33	3	0.35185429	3.53E-10	6.45E-06	0.492	0.307
CLDND1	3	0.35113347	5.64E-16	1.03E-11	0.558	0.293
KDM7A	3	0.34249573	3.35E-15	6.11E-11	0.418	0.157
ANXA5	3	0.34223949	2.28E-16	4.17E-12	0.91	0.77
CD44	3	0.3405074	8.69E-16	1.59E-11	0.979	0.829
EMP3	3	0.3404001	5.87E-10	1.07E-05	0.919	0.826
NBPF14	3	0.33807953	3.34E-15	6.10E-11	0.352	0.101
ERRFI1	3	0.33480587	1.96E-08	0.0003585	0.439	0.254

FN1	3	0.33325342	1.92E-10	3.51E-06	0.995	1
AK2	3	0.33281124	9.32E-08	0.00170043	0.708	0.596
SNRNP27	3	0.33097204	5.68E-11	1.04E-06	0.537	0.331
FNIP2	3	0.33015402	2.03E-12	3.70E-08	0.587	0.345
CHMP5	3	0.33011797	1.23E-13	2.24E-09	0.71	0.498
GPRC5A	3	0.32688108	1.15E-14	2.10E-10	0.363	0.111
SFT2D1	3	0.32540019	3.38E-13	6.18E-09	0.793	0.659
CA9	3	0.32517136	1.25E-10	2.28E-06	0.318	0.118
LRRFIP1	3	0.32510435	1.28E-10	2.33E-06	0.561	0.362
ATP6V0B	3	0.32257534	1.85E-11	3.37E-07	0.646	0.456
CLK1	3	0.32195973	1.94E-09	3.54E-05	0.596	0.432
ACTR10	3	0.31813772	1.34E-14	2.44E-10	0.504	0.233
CTD-2095E4.5	3	0.3148236	6.67E-09	0.00012164	0.242	0.08
HSPA5	3	0.3124267	2.09E-06	0.03821286	0.437	0.282
MED21	3	0.30999656	5.92E-08	0.00108076	0.527	0.362
ISG15	3	0.308408	1.94E-07	0.00354406	0.24	0.091
NPR3	3	0.30825288	2.44E-16	4.44E-12	0.207	0
FERMT2	3	0.30600985	2.76E-11	5.03E-07	0.489	0.265
SH3BP5	3	0.30598854	1.21E-09	2.21E-05	0.447	0.244
CAPZA2	3	0.30565439	5.29E-11	9.65E-07	0.76	0.61
KLHL21	3	0.30539885	9.72E-15	1.77E-10	0.385	0.129
TNS1	3	0.3022983	1.28E-12	2.34E-08	0.361	0.132
AC090498.1	3	0.30083779	4.38E-29	8.00E-25	1	1
PHLDA2	3	0.30042711	1.78E-11	3.24E-07	0.297	0.094
SAR1A	3	0.29971663	4.90E-10	8.94E-06	0.594	0.411
LTBP1	3	0.29810177	9.97E-18	1.82E-13	0.283	0.031
MSANTD3	3	0.29723948	3.54E-11	6.46E-07	0.439	0.223
XBP1	3	0.29550973	2.34E-07	0.00427682	0.705	0.589
DDX21	3	0.293879	2.09E-12	3.82E-08	0.406	0.171
RGCC	3	0.29373604	7.90E-07	0.01442048	0.862	0.77
BET1	3	0.29265509	1.36E-12	2.49E-08	0.439	0.199
ZFAND5	3	0.29033737	4.96E-07	0.00905308	0.629	0.484
C2orf49	3	0.29022406	4.45E-11	8.12E-07	0.366	0.153
OVOS2	3	0.28955789	8.68E-10	1.58E-05	0.285	0.098
MRPL57	3	0.28940993	1.52E-09	2.77E-05	0.805	0.672
ATP6V1C1	3	0.28894315	2.20E-12	4.01E-08	0.461	0.226
MOB4	3	0.28858247	2.45E-11	4.48E-07	0.437	0.209
NDUFB8	3	0.2884155	9.22E-09	0.00016821	0.831	0.753
S100A16	3	0.28690085	7.65E-07	0.01395984	0.515	0.38

LINC00493	3	0.28566446	3.65E-12	6.65E-08	0.872	0.749
CLMP	3	0.28526803	2.34E-12	4.26E-08	0.418	0.178
BNIP3	3	0.28448099	4.83E-10	8.82E-06	0.995	0.958
PTX3	3	0.28279374	7.75E-09	0.00014143	0.283	0.105
RHOD	3	0.27991911	8.55E-10	1.56E-05	0.525	0.324
RHEB	3	0.27828981	6.47E-09	0.00011806	0.853	0.756
KMT2E-AS1	3	0.27709886	4.31E-11	7.86E-07	0.261	0.07
LINC00152	3	0.27664097	1.65E-06	0.03003806	0.444	0.286
METAP2	3	0.27577118	4.90E-11	8.95E-07	0.831	0.631
PTGES	3	0.27553868	1.02E-12	1.85E-08	0.28	0.066
SQSTM1	3	0.2754133	1.22E-07	0.00222792	0.943	0.93
C4orf48	3	0.27469162	8.35E-10	1.52E-05	0.511	0.286
EIF5	3	0.27405454	6.33E-09	0.00011544	0.743	0.603
CNIH1	3	0.27175763	2.82E-09	5.14E-05	0.86	0.728
PHF1	3	0.27027321	1.64E-09	3.00E-05	0.513	0.31
NOL7	3	0.26953717	6.02E-08	0.0010988	0.653	0.54
USMG5	3	0.26903352	5.46E-12	9.97E-08	0.96	0.899
GPX1	3	0.26739325	2.54E-07	0.00463165	0.924	0.882
GNPNAT1	3	0.26687214	8.69E-15	1.59E-10	0.314	0.073
NTMT1	3	0.26459491	1.73E-10	3.16E-06	0.489	0.282
HNRNPDL	3	0.26281126	2.51E-09	4.57E-05	0.853	0.707
SLC20A1	3	0.26193927	2.13E-06	0.03878945	0.316	0.171
ZFYVE16	3	0.26088686	9.33E-14	1.70E-09	0.318	0.084
QSOX1	3	0.26086387	3.37E-08	0.00061493	0.409	0.226
PDE4B	3	0.26070292	2.74E-10	5.00E-06	0.549	0.314
CCDC59	3	0.26001819	2.11E-07	0.00384969	0.485	0.317
NDUFC1	3	0.25745405	2.38E-10	4.34E-06	0.9	0.787
CLDN12	3	0.25678487	1.27E-10	2.32E-06	0.428	0.206
SAP18	3	0.25626896	5.31E-09	9.70E-05	0.86	0.774
ACKR3	3	0.25419172	2.48E-15	4.53E-11	0.283	0.045
SYF2	3	0.25407577	2.20E-09	4.02E-05	0.898	0.78
ZNF581	3	0.25253951	1.07E-08	0.0001959	0.496	0.31
TRAM1	3	0.25178283	4.71E-07	0.0085939	0.834	0.725
FAM96B	3	0.25176093	4.75E-08	0.00086721	0.76	0.669
C16orf13	3	0.25125211	3.90E-08	0.00071172	0.793	0.683
NAA38	3	-0.2510091	9.22E-10	1.68E-05	0.444	0.659
COX7A2L	3	-0.2513472	8.34E-11	1.52E-06	0.836	0.923
ZFP36	3	-0.2528146	1.73E-07	0.00315729	0.126	0.279
RPL21	3	-0.2529971	5.47E-34	9.97E-30	1	1

BHLHE41	3	-0.2531549	2.69E-11	4.91E-07	0.124	0.324
PNKD	3	-0.2535954	1.69E-14	3.08E-10	0.171	0.429
HERPUD1	3	-0.2538816	1.03E-09	1.89E-05	0.309	0.519
CHID1	3	-0.2552393	4.26E-09	7.78E-05	0.299	0.488
SLC29A1	3	-0.2553172	2.51E-11	4.58E-07	0.204	0.425
NFIC	3	-0.2556373	2.26E-10	4.13E-06	0.271	0.491
TUBB	3	-0.2559484	4.48E-08	0.000818	0.691	0.847
TMEM14C	3	-0.2562904	1.96E-11	3.58E-07	0.475	0.711
COL5A2	3	-0.2563989	8.17E-13	1.49E-08	0.093	0.296
COL11A2	3	-0.2564508	2.92E-10	5.33E-06	0.19	0.394
RPL27	3	-0.2564815	1.48E-24	2.70E-20	1	1
MPP6	3	-0.2571708	5.42E-11	9.90E-07	0.154	0.355
VAMP5	3	-0.2572094	9.70E-09	0.00017694	0.328	0.516
ATP5A1	3	-0.2572704	1.51E-09	2.75E-05	0.375	0.596
LTA4H	3	-0.2573225	6.83E-09	0.00012471	0.439	0.631
TMEM219	3	-0.2590516	6.21E-10	1.13E-05	0.539	0.721
VCAN	3	-0.2592137	1.23E-11	2.24E-07	0.119	0.324
METTL7A	3	-0.2602525	2.33E-12	4.25E-08	0.043	0.209
TRAPPC1	3	-0.260766	1.28E-10	2.34E-06	0.302	0.53
ATP5H	3	-0.260861	3.46E-09	6.31E-05	0.556	0.732
PABPC4	3	-0.2636701	1.19E-08	0.00021715	0.485	0.659
UBA52	3	-0.2638513	2.83E-30	5.17E-26	1	1
HMGN3	3	-0.2646571	4.24E-13	7.73E-09	0.192	0.443
SPINT2	3	-0.2653676	4.39E-11	8.02E-07	0.261	0.488
BBX	3	-0.2658615	1.49E-13	2.73E-09	0.169	0.411
PRKCSH	3	-0.2665528	2.44E-12	4.45E-08	0.204	0.439
TMEM98	3	-0.2666168	3.50E-14	6.39E-10	0.159	0.408
KRT8	3	-0.2689012	3.16E-08	0.00057584	0.266	0.446
RPL18A	3	-0.2690207	9.10E-34	1.66E-29	1	1
ERP29	3	-0.2693917	2.46E-11	4.48E-07	0.561	0.784
RASD1	3	-0.2704567	1.62E-17	2.96E-13	0.045	0.265
CHMP3	3	-0.2706012	8.55E-10	1.56E-05	0.425	0.617
SRSF5	3	-0.2706137	6.32E-12	1.15E-07	0.546	0.763
UGP2	3	-0.2706726	1.10E-08	0.00020144	0.539	0.707
ITM2B	3	-0.2710706	8.12E-10	1.48E-05	0.952	0.986
MYEOV2	3	-0.2721333	4.76E-11	8.69E-07	0.565	0.749
GOLIM4	3	-0.2727182	8.96E-07	0.01634559	0.261	0.425
ATP1A1	3	-0.2730198	1.40E-13	2.56E-09	0.228	0.484
ST13	3	-0.2731202	1.87E-14	3.41E-10	0.917	0.958

CD320	3	-0.2741719	1.23E-09	2.25E-05	0.219	0.411
SERTAD4-AS1	3	-0.2754672	1.49E-22	2.72E-18	0.024	0.268
UQCRH	3	-0.2765809	2.24E-16	4.09E-12	0.95	0.979
GALNT15	3	-0.2765946	6.71E-14	1.22E-09	0.09	0.303
SEP1	3	-0.277548	2.25E-18	4.10E-14	0.083	0.341
TGFBR2	3	-0.2775629	1.23E-12	2.25E-08	0.128	0.348
H2AFJ	3	-0.2780337	9.55E-11	1.74E-06	0.582	0.77
HNRNPM	3	-0.278622	4.39E-13	8.01E-09	0.23	0.481
GBP2	3	-0.279323	9.33E-11	1.70E-06	0.173	0.376
MRPS21	3	-0.2801119	4.92E-13	8.98E-09	0.781	0.909
ATRAID	3	-0.2801266	2.88E-09	5.26E-05	0.656	0.822
MYLK	3	-0.2808022	7.58E-19	1.38E-14	0.05	0.286
H1FX	3	-0.2810665	7.15E-14	1.31E-09	0.107	0.328
AC005152.3	3	-0.2811204	8.47E-22	1.55E-17	0.021	0.258
GSTA4	3	-0.2815088	1.97E-13	3.59E-09	0.176	0.411
SNHG8	3	-0.2815708	1.88E-11	3.43E-07	0.815	0.899
TCEAL8	3	-0.2820006	4.31E-12	7.86E-08	0.525	0.728
PARK7	3	-0.2821474	4.56E-13	8.31E-09	0.786	0.923
PAM	3	-0.2833895	1.51E-12	2.76E-08	0.371	0.627
OXA1L	3	-0.2842847	1.20E-11	2.19E-07	0.406	0.627
IFI27L2	3	-0.2850427	6.69E-12	1.22E-07	0.705	0.868
FTL	3	-0.2853683	8.35E-17	1.52E-12	1	1
FKBP10	3	-0.2855121	7.42E-15	1.35E-10	0.183	0.446
TSPAN3	3	-0.2865238	1.75E-12	3.18E-08	0.335	0.575
C9orf3	3	-0.2869037	6.89E-11	1.26E-06	0.216	0.432
PDIA3	3	-0.2871866	1.83E-09	3.33E-05	0.333	0.533
EIF2A	3	-0.2874849	1.88E-08	0.00034381	0.556	0.7
RPN2	3	-0.2878404	3.20E-17	5.84E-13	0.076	0.321
SERPING1	3	-0.2882714	7.05E-10	1.29E-05	0.423	0.634
PRKCDBP	3	-0.2890045	1.29E-10	2.35E-06	0.325	0.544
SERF2	3	-0.2896674	2.97E-18	5.41E-14	1	1
SOD1	3	-0.2907383	2.24E-13	4.08E-09	0.914	0.969
BEX2	3	-0.2917164	1.01E-11	1.84E-07	0.185	0.404
MYO10	3	-0.2920777	4.00E-11	7.30E-07	0.1	0.289
CRIP2	3	-0.292351	5.32E-12	9.70E-08	0.245	0.477
RPL12	3	-0.292466	1.22E-28	2.23E-24	1	1
WWP2	3	-0.2926203	7.11E-09	0.00012974	0.361	0.568
EBPL	3	-0.2942031	2.13E-14	3.88E-10	0.207	0.46
COL6A2	3	-0.2949805	6.21E-08	0.00113324	0.359	0.523

HLA-B	3	-0.2955033	1.02E-06	0.01858939	0.648	0.742
SSR2	3	-0.2982135	1.41E-13	2.57E-09	0.715	0.882
F13A1	3	-0.2996296	2.44E-08	0.00044536	0.311	0.505
ARL6IP5	3	-0.29971	4.68E-11	8.54E-07	0.485	0.672
CMPK1	3	-0.2999832	4.87E-12	8.88E-08	0.682	0.847
C19orf43	3	-0.3000055	3.01E-14	5.49E-10	0.781	0.892
C12orf57	3	-0.3003899	1.15E-19	2.09E-15	0.955	0.993
PGAM1	3	-0.3005936	4.54E-13	8.29E-09	0.634	0.805
TMED10	3	-0.3019983	1.19E-09	2.18E-05	0.708	0.85
RPL14	3	-0.3033113	1.79E-33	3.27E-29	1	1
TCEA3	3	-0.303777	1.25E-18	2.27E-14	0.162	0.456
TNFRSF1A	3	-0.3039012	1.58E-13	2.89E-09	0.216	0.456
RPS10	3	-0.3045751	2.60E-22	4.75E-18	0.995	0.993
PTMA	3	-0.3054576	1.95E-22	3.57E-18	1	1
RPS3	3	-0.305538	9.39E-39	1.71E-34	1	1
SCRG1	3	-0.3056347	4.15E-07	0.00757765	0.891	0.951
PDE4DIP	3	-0.3064168	4.06E-16	7.41E-12	0.209	0.481
SOCS3	3	-0.3065002	3.83E-10	6.99E-06	0.183	0.376
NUCB1	3	-0.3065741	9.26E-14	1.69E-09	0.352	0.613
FCGRT	3	-0.3067776	1.13E-12	2.06E-08	0.354	0.578
SARAF	3	-0.3086806	1.58E-12	2.88E-08	0.589	0.787
PRCP	3	-0.3088758	9.22E-21	1.68E-16	0.083	0.366
MSRB1	3	-0.3093466	5.14E-19	9.37E-15	0.173	0.481
PRDX4	3	-0.309433	6.50E-11	1.19E-06	0.494	0.718
BTG2	3	-0.3138679	2.35E-12	4.28E-08	0.145	0.359
SCARA3	3	-0.314311	4.68E-24	8.54E-20	0.088	0.408
RBMX	3	-0.3144262	6.37E-13	1.16E-08	0.544	0.749
COMMD6	3	-0.3148047	4.63E-21	8.45E-17	0.998	1
RPL37A	3	-0.3149476	2.41E-45	4.40E-41	1	1
ARL6IP4	3	-0.3163388	3.87E-15	7.05E-11	0.786	0.93
POLR2L	3	-0.3170715	2.78E-10	5.07E-06	0.72	0.878
HMGB1	3	-0.3184221	2.88E-14	5.26E-10	0.72	0.871
GAS6	3	-0.3186121	2.11E-17	3.85E-13	0.121	0.383
DUT	3	-0.3186121	6.18E-13	1.13E-08	0.473	0.679
BSG	3	-0.3188887	9.97E-14	1.82E-09	0.454	0.69
UQCRB	3	-0.3195212	2.41E-14	4.39E-10	0.938	0.993
TSPAN4	3	-0.3203849	2.80E-14	5.12E-10	0.221	0.467
TOMM7	3	-0.3211992	4.54E-34	8.28E-30	1	1
IFITM3	3	-0.3213192	1.66E-12	3.04E-08	0.8	0.93

RPS18	3	-0.3224453	7.39E-43	1.35E-38	1	1
GRN	3	-0.3233037	1.20E-17	2.18E-13	0.119	0.383
ATRNL1	3	-0.3239617	1.02E-24	1.86E-20	0.031	0.303
ANKH	3	-0.3241273	1.87E-11	3.41E-07	0.39	0.61
RPS29	3	-0.3242693	1.68E-38	3.07E-34	1	1
PPIA	3	-0.3254997	1.05E-15	1.91E-11	0.895	0.962
CUTA	3	-0.3259922	3.34E-13	6.10E-09	0.622	0.829
OS9	3	-0.3263171	2.19E-16	4.00E-12	0.242	0.526
HADHA	3	-0.3270973	3.86E-16	7.05E-12	0.216	0.491
INHBA	3	-0.327136	6.57E-15	1.20E-10	0.135	0.383
RPS12	3	-0.3285966	3.29E-35	6.00E-31	1	1
RPS7	3	-0.3286922	1.61E-32	2.93E-28	1	1
EIF3E	3	-0.3304338	2.73E-18	4.99E-14	0.993	0.997
HSP90AB1	3	-0.3306571	7.11E-15	1.30E-10	0.914	0.972
DNM3OS	3	-0.3307369	3.97E-19	7.24E-15	0.102	0.376
RPS5	3	-0.3309362	6.03E-36	1.10E-31	1	1
PFN1	3	-0.3311294	3.21E-14	5.86E-10	0.86	0.93
RPL13	3	-0.3316822	1.68E-50	3.06E-46	1	1
ZBTB20	3	-0.3320757	2.10E-21	3.84E-17	0.124	0.432
ENPP1	3	-0.3353154	1.40E-21	2.56E-17	0.107	0.408
RP11-983P16.4	3	-0.3353327	9.05E-17	1.65E-12	0.195	0.474
RPS4Y1	3	-0.3354919	5.94E-23	1.08E-18	0.983	0.99
CTSK	3	-0.3358977	8.73E-21	1.59E-16	0.074	0.345
NACA	3	-0.3368573	3.46E-32	6.32E-28	0.998	1
RPL15	3	-0.3389884	1.26E-49	2.30E-45	1	1
AC003090.1	3	-0.3404901	3.10E-28	5.66E-24	0.021	0.314
PLXDC2	3	-0.3406696	1.33E-16	2.42E-12	0.23	0.523
NFIA	3	-0.3407714	2.62E-20	4.79E-16	0.121	0.415
LRRC2	3	-0.3411555	2.39E-22	4.36E-18	0.09	0.387
RPL6	3	-0.343089	2.64E-36	4.82E-32	1	1
SH3BGRL	3	-0.3437895	2.33E-18	4.25E-14	0.268	0.578
FIS1	3	-0.3440884	1.39E-16	2.53E-12	0.511	0.749
VKORC1	3	-0.3451969	1.02E-15	1.87E-11	0.812	0.934
NUPR1	3	-0.3452128	2.27E-25	4.14E-21	0.962	0.986
CTSD	3	-0.3454399	1.60E-16	2.93E-12	0.72	0.902
LDHB	3	-0.3460885	1.52E-15	2.77E-11	0.622	0.822
RPL7A	3	-0.346323	1.14E-38	2.07E-34	1	1
PIM3	3	-0.3463366	2.93E-11	5.35E-07	0.394	0.613
RPL22	3	-0.3465396	1.77E-36	3.24E-32	1	1

MAOB	3	-0.3468459	5.88E-14	1.07E-09	0.285	0.547
RPS9	3	-0.3469312	4.38E-43	7.99E-39	1	1
HIST1H1C	3	-0.3470219	5.75E-09	0.00010495	0.283	0.477
RNF130	3	-0.3478906	3.93E-14	7.18E-10	0.385	0.624
RPLP1	3	-0.3486856	1.91E-52	3.49E-48	1	1
PRDX5	3	-0.3497849	1.42E-17	2.59E-13	0.805	0.944
RPL8	3	-0.3500538	9.85E-48	1.80E-43	1	1
CMB9-22P13.1	3	-0.3526398	4.68E-16	8.53E-12	0.333	0.606
PTPRD-AS1	3	-0.35365	1.87E-18	3.41E-14	0.197	0.488
PDGFRA	3	-0.3539679	1.91E-26	3.48E-22	0.067	0.387
AP2M1	3	-0.3541163	5.18E-18	9.45E-14	0.378	0.652
C7orf73	3	-0.3567068	2.61E-17	4.76E-13	0.542	0.815
SERPINA1	3	-0.3569038	6.61E-13	1.21E-08	0.924	0.99
KLF6	3	-0.3572712	2.86E-12	5.22E-08	0.226	0.46
ARPC1B	3	-0.3576429	5.47E-13	9.99E-09	0.392	0.627
CCDC102B	3	-0.3577744	3.87E-25	7.06E-21	0.036	0.317
RPS13	3	-0.3578978	2.46E-44	4.49E-40	1	1
ASS1	3	-0.3581309	1.36E-15	2.48E-11	0.425	0.676
JUN	3	-0.358269	4.01E-10	7.31E-06	0.202	0.394
PNISR	3	-0.3590853	6.39E-15	1.17E-10	0.513	0.721
SERPINA5	3	-0.3600646	5.86E-20	1.07E-15	0.128	0.418
TPM2	3	-0.3621708	7.89E-15	1.44E-10	0.273	0.53
ARPC1A	3	-0.3624657	4.77E-18	8.71E-14	0.375	0.672
IFITM1	3	-0.3635421	1.07E-11	1.95E-07	0.278	0.505
SSPN	3	-0.365451	8.26E-25	1.51E-20	0.076	0.387
SSR4	3	-0.366021	1.93E-16	3.52E-12	0.874	0.958
MFGE8	3	-0.3664426	1.08E-11	1.97E-07	0.615	0.815
ERGIC3	3	-0.3665232	1.32E-16	2.40E-12	0.613	0.836
RPL23	3	-0.3668548	1.66E-33	3.04E-29	1	1
CPE	3	-0.3684804	4.64E-15	8.46E-11	0.539	0.77
RPL13A	3	-0.3691126	8.36E-59	1.53E-54	1	1
FHL1	3	-0.3692477	3.45E-17	6.29E-13	0.178	0.456
MAF	3	-0.3694384	2.01E-18	3.66E-14	0.162	0.453
MIA	3	-0.369491	1.38E-10	2.51E-06	0.919	0.962
PPIC	3	-0.3715226	1.22E-16	2.22E-12	0.2	0.467
ILF3-AS1	3	-0.3717653	1.77E-17	3.23E-13	0.214	0.498
EIF2S3	3	-0.3721766	2.31E-11	4.22E-07	0.499	0.669
HINT1	3	-0.3726218	1.65E-29	3.02E-25	0.971	1
LINC01503	3	-0.373337	2.92E-15	5.33E-11	0.169	0.425

LITAF	3	-0.3734418	2.90E-19	5.29E-15	0.333	0.638
MYL6	3	-0.3747117	3.99E-19	7.28E-15	0.848	0.972
TPD52L1	3	-0.3756599	1.60E-17	2.92E-13	0.221	0.505
ENAH	3	-0.3767472	7.41E-25	1.35E-20	0.109	0.436
CTGF	3	-0.3774281	3.48E-08	0.0006347	0.252	0.439
SEPP1	3	-0.3792596	8.35E-20	1.52E-15	0.181	0.481
SLC25A3	3	-0.3796282	7.01E-23	1.28E-18	0.846	0.976
RPL37	3	-0.3798067	8.29E-50	1.51E-45	1	1
RPS27A	3	-0.3817868	1.31E-55	2.39E-51	1	1
CDO1	3	-0.3831506	3.14E-20	5.72E-16	0.133	0.432
RPS23	3	-0.3838758	1.98E-49	3.61E-45	1	1
RPL19	3	-0.3857956	1.51E-56	2.76E-52	1	1
RPL10	3	-0.3862707	1.81E-52	3.29E-48	1	1
C1orf21	3	-0.3867157	3.35E-21	6.11E-17	0.264	0.592
EFNA1	3	-0.3870417	1.20E-17	2.19E-13	0.176	0.467
RPS4X	3	-0.3875848	1.13E-52	2.06E-48	1	1
ATPIF1	3	-0.3888405	2.97E-18	5.42E-14	0.458	0.711
CCDC88A	3	-0.3896092	4.13E-23	7.53E-19	0.09	0.394
TTC3	3	-0.3900807	5.61E-17	1.02E-12	0.473	0.707
GNAS	3	-0.3906978	8.35E-17	1.52E-12	0.39	0.659
NMRK1	3	-0.3908135	6.78E-21	1.24E-16	0.195	0.512
RPS2	3	-0.3917227	6.74E-48	1.23E-43	1	1
ITGA10	3	-0.393677	2.07E-22	3.78E-18	0.257	0.617
EIF3D	3	-0.3955219	1.66E-19	3.03E-15	0.658	0.868
MSMP	3	-0.3957492	5.34E-09	9.75E-05	0.197	0.401
HIST1H4C	3	-0.3963764	3.39E-15	6.18E-11	0.371	0.62
CCNG1	3	-0.3971369	7.39E-19	1.35E-14	0.278	0.578
RPL30	3	-0.397321	2.44E-50	4.45E-46	1	1
THY1	3	-0.3978722	1.10E-18	2.02E-14	0.1	0.366
PIK3R1	3	-0.4008502	7.55E-24	1.38E-19	0.045	0.324
RPS6	3	-0.4014986	1.46E-57	2.66E-53	1	1
SDF4	3	-0.4054091	8.66E-20	1.58E-15	0.368	0.655
HLA-A	3	-0.4092618	6.52E-15	1.19E-10	0.781	0.909
GLTSCR2	3	-0.409993	1.83E-23	3.34E-19	0.85	0.955
C1S	3	-0.4102793	6.58E-18	1.20E-13	0.527	0.787
GADD45B	3	-0.4146056	2.85E-14	5.20E-10	0.185	0.443
RPSA	3	-0.417342	5.94E-36	1.08E-31	0.99	1
CD9	3	-0.4176085	1.49E-15	2.72E-11	0.29	0.557
RPL5	3	-0.417686	3.67E-41	6.70E-37	1	1

SLC14A1	3	-0.4182419	1.65E-25	3.00E-21	0.119	0.453
CXXC5	3	-0.4189816	5.02E-29	9.15E-25	0.09	0.446
PSAP	3	-0.4192667	4.15E-22	7.58E-18	0.81	0.937
MDFI	3	-0.4228909	7.24E-25	1.32E-20	0.159	0.516
SSR3	3	-0.4270944	1.88E-21	3.44E-17	0.784	0.934
RABAC1	3	-0.4278735	7.25E-22	1.32E-17	0.644	0.902
DMKN	3	-0.4316676	6.73E-27	1.23E-22	0.216	0.585
SPON2	3	-0.4317253	4.96E-30	9.05E-26	0.045	0.376
RPS17	3	-0.437657	2.99E-54	5.45E-50	1	1
CITED2	3	-0.4389272	3.37E-21	6.15E-17	0.164	0.481
ITM2A	3	-0.4391725	1.37E-15	2.51E-11	0.223	0.474
RPLP2	3	-0.4391977	1.44E-70	2.62E-66	1	1
CYBRD1	3	-0.4412221	1.35E-23	2.47E-19	0.439	0.735
NDUFA11	3	-0.4425206	2.77E-24	5.05E-20	0.767	0.927
FAM46A	3	-0.4428699	4.85E-13	8.85E-09	0.366	0.575
NAP1L1	3	-0.4468113	1.23E-29	2.24E-25	0.912	0.979
GSN	3	-0.4495427	1.68E-23	3.07E-19	0.219	0.564
GADD45A	3	-0.4524363	1.06E-09	1.94E-05	0.347	0.537
RPL18	3	-0.4538579	2.91E-58	5.32E-54	1	1
TPM1	3	-0.4599792	3.03E-11	5.52E-07	0.513	0.69
RPL9	3	-0.4600602	1.44E-61	2.62E-57	1	1
MOXD1	3	-0.4649401	2.89E-23	5.28E-19	0.2	0.537
SLC40A1	3	-0.4667157	2.42E-36	4.42E-32	0.033	0.404
LUM	3	-0.4712881	8.40E-30	1.53E-25	1	1
PEBP1	3	-0.4752402	7.65E-25	1.40E-20	0.658	0.875
RPL23A	3	-0.4755063	3.77E-71	6.88E-67	1	1
BTF3	3	-0.4775428	1.48E-42	2.70E-38	0.99	0.997
AHNAK	3	-0.4779675	1.44E-28	2.62E-24	0.235	0.627
IER2	3	-0.4780008	1.32E-19	2.40E-15	0.271	0.582
RPS16	3	-0.4792389	1.28E-67	2.33E-63	1	1
SEC11A	3	-0.4795278	2.27E-34	4.14E-30	0.765	0.948
S100A13	3	-0.4848857	8.42E-38	1.54E-33	0.969	1
TSPO	3	-0.4946509	5.31E-29	9.70E-25	0.646	0.927
ITM2C	3	-0.4987696	1.36E-25	2.47E-21	0.188	0.533
NPC2	3	-0.5009337	1.55E-38	2.82E-34	0.95	1
ACTG1	3	-0.5024084	9.56E-26	1.74E-21	0.955	0.997
HMGN2	3	-0.5039166	1.22E-29	2.23E-25	0.748	0.948
HLA-C	3	-0.5077133	5.56E-21	1.02E-16	0.743	0.909
SNAI2	3	-0.5089529	3.69E-19	6.74E-15	0.228	0.523

RPL34	3	-0.5091944	4.08E-70	7.44E-66	1	1
RPS3A	3	-0.5092837	8.55E-73	1.56E-68	1	1
PDIA6	3	-0.510454	9.26E-26	1.69E-21	0.337	0.686
PCBP2	3	-0.5161649	3.45E-32	6.29E-28	0.805	0.962
ID4	3	-0.5217198	8.26E-24	1.51E-19	0.311	0.652
RPL3	3	-0.5218932	2.99E-71	5.46E-67	1	1
S100A4	3	-0.5289718	2.43E-12	4.44E-08	0.508	0.721
CLU	3	-0.529171	1.13E-17	2.07E-13	0.995	1
PTRF	3	-0.5316356	1.89E-32	3.45E-28	0.204	0.613
EEF1B2	3	-0.5318805	1.63E-43	2.97E-39	0.976	0.997
BAMBI	3	-0.5324053	1.76E-15	3.21E-11	0.347	0.603
BRK1	3	-0.5326257	2.16E-34	3.94E-30	0.677	0.937
DDR2	3	-0.5387147	3.60E-37	6.56E-33	0.166	0.606
SPARC	3	-0.5398643	2.81E-32	5.12E-28	0.147	0.554
HSPB1	3	-0.5404544	1.14E-27	2.08E-23	0.473	0.794
DUSP1	3	-0.5423352	4.69E-26	8.56E-22	0.409	0.753
IGFBP7	3	-0.5444838	1.20E-16	2.19E-12	0.641	0.85
RGS2	3	-0.5559761	1.57E-09	2.86E-05	0.423	0.613
PCOLCE2	3	-0.5564223	5.89E-28	1.07E-23	0.732	0.92
YBX1	3	-0.5614818	2.62E-47	4.77E-43	0.964	1
ISLR	3	-0.5618024	6.84E-37	1.25E-32	0.078	0.481
S100A1	3	-0.5667951	1.57E-43	2.86E-39	0.986	1
EIF4B	3	-0.5693529	3.86E-33	7.05E-29	0.639	0.913
PRUNE2	3	-0.5746191	7.13E-32	1.30E-27	0.195	0.603
VIM	3	-0.5778004	1.04E-39	1.90E-35	0.993	1
FBXO2	3	-0.5789907	3.70E-32	6.74E-28	0.435	0.805
RPLP0	3	-0.5857685	2.73E-72	4.97E-68	0.998	1
PABPC1	3	-0.5858156	1.32E-36	2.41E-32	0.76	0.958
RPL7	3	-0.5953333	2.01E-66	3.66E-62	1	1
EIF3L	3	-0.599586	1.71E-41	3.11E-37	0.694	0.951
H3F3A	3	-0.601454	2.37E-50	4.32E-46	0.895	1
TCF4	3	-0.6112532	3.50E-42	6.39E-38	0.185	0.666
FOS	3	-0.6195394	2.65E-29	4.84E-25	0.371	0.756
FGF7	3	-0.6237202	3.33E-28	6.08E-24	0.152	0.544
IFITM2	3	-0.6251204	2.47E-35	4.51E-31	0.743	0.965
S100B	3	-0.6303526	6.71E-35	1.22E-30	0.392	0.819
ARHGAP29	3	-0.6493685	1.24E-39	2.27E-35	0.195	0.645
MT-ND5	3	-0.6498057	5.00E-31	9.12E-27	0.494	0.815
NOVA1	3	-0.6582328	9.44E-28	1.72E-23	0.753	0.948

COL9A3	3	-0.6635334	1.46E-27	2.66E-23	0.185	0.554
CYR61	3	-0.665957	1.76E-34	3.21E-30	0.105	0.512
COL11A1	3	-0.6660386	2.05E-36	3.75E-32	0.235	0.693
P4HB	3	-0.666513	2.73E-32	4.98E-28	0.477	0.812
EIF3H	3	-0.6679979	1.26E-52	2.31E-48	0.941	0.997
BGN	3	-0.6686116	5.45E-38	9.95E-34	0.893	0.993
CTSC	3	-0.6718652	3.70E-30	6.76E-26	0.119	0.488
PTMS	3	-0.6722521	3.17E-41	5.78E-37	0.401	0.829
NGFRAP1	3	-0.6738731	1.33E-44	2.44E-40	0.513	0.934
CCNI	3	-0.6748848	4.20E-62	7.67E-58	0.988	0.997
ID2	3	-0.6768243	8.90E-25	1.62E-20	0.489	0.78
HTRA1	3	-0.7013831	6.73E-46	1.23E-41	0.466	0.906
FTH1	3	-0.7021831	1.12E-25	2.04E-21	1	1
MT-ND4	3	-0.7049128	3.57E-35	6.52E-31	0.933	0.972
JUNB	3	-0.7123687	5.32E-34	9.71E-30	0.534	0.895
RPS8	3	-0.7144676	5.30E-94	9.68E-90	1	1
APOD	3	-0.7145078	5.82E-28	1.06E-23	0.185	0.551
RPS20	3	-0.7249624	2.56E-82	4.68E-78	1	1
FXYD6	3	-0.7257678	5.48E-55	9.99E-51	0.494	0.937
CRTAP	3	-0.7275402	3.06E-50	5.59E-46	0.397	0.843
EID1	3	-0.7282399	2.97E-57	5.42E-53	0.781	0.972
PRELP	3	-0.7318053	7.82E-40	1.43E-35	0.67	0.951
CEBPD	3	-0.7376956	2.22E-38	4.06E-34	0.755	0.965
TPT1	3	-0.7389289	1.76E-88	3.21E-84	1	1
CD99	3	-0.7426678	1.24E-53	2.26E-49	0.732	0.969
RPS11	3	-0.7455075	6.90E-86	1.26E-81	0.99	1
HNRNPA1	3	-0.752611	2.65E-64	4.83E-60	0.962	0.997
MALAT1	3	-0.7538494	9.79E-42	1.79E-37	1	1
RBP4	3	-0.7551271	8.31E-35	1.52E-30	0.34	0.735
CP	3	-0.7561723	1.73E-42	3.15E-38	0.53	0.909
LECT1	3	-0.7600737	6.66E-33	1.22E-28	0.259	0.679
RHOBTB3	3	-0.7647645	1.05E-41	1.92E-37	0.292	0.728
PAX1	3	-0.7647901	4.92E-54	8.98E-50	0.228	0.76
RPL10A	3	-0.7766366	2.67E-84	4.88E-80	1	1
RPL4	3	-0.787351	1.76E-81	3.21E-77	0.993	1
FMOD	3	-0.8108613	5.81E-49	1.06E-44	0.501	0.909
B2M	3	-0.813565	2.38E-65	4.34E-61	0.988	1
YBX3	3	-0.8321235	6.67E-62	1.22E-57	0.608	0.969
SOD3	3	-0.8446208	3.26E-42	5.94E-38	0.907	0.993

EEF2	3	-0.8565789	3.12E-61	5.70E-57	0.964	0.997
MT-CO3	3	-0.9003637	6.80E-49	1.24E-44	0.822	0.941
ID1	3	-0.9041066	2.16E-34	3.94E-30	0.371	0.77
CCL2	3	-0.9143039	6.71E-11	1.23E-06	0.024	0.157
TSC22D1	3	-0.9175177	2.93E-37	5.35E-33	0.477	0.833
ZFP36L2	3	-0.9230692	5.67E-43	1.03E-38	0.2	0.662
NNMT	3	-0.9283439	1.10E-57	2.01E-53	0.781	0.979
MT-CO1	3	-0.9354126	1.82E-50	3.32E-46	0.922	0.976
CST3	3	-0.9500077	1.51E-67	2.76E-63	0.931	0.997
EEF1A1	3	-0.9521759	5.40E-102	9.85E-98	1	1
DCN	3	-0.9533296	1.30E-75	2.38E-71	0.995	1
CHI3L1	3	-0.9557609	3.72E-42	6.79E-38	0.857	0.983
MT-CO2	3	-0.962029	7.02E-50	1.28E-45	0.903	0.969
COL2A1	3	-0.9652335	4.61E-54	8.41E-50	0.2	0.756
ACAN	3	-1.0143348	1.26E-60	2.30E-56	0.646	0.972
MT-ND3	3	-1.0490966	6.13E-59	1.12E-54	0.485	0.889
CCDC80	3	-1.0548503	1.80E-51	3.29E-47	0.618	0.958
CAPS	3	-1.0611689	4.56E-59	8.32E-55	0.387	0.875
MT-ND1	3	-1.0834577	7.55E-58	1.38E-53	0.466	0.909
SPTSSB	3	-1.0973021	1.29E-49	2.36E-45	0.007	0.449
C2orf40	3	-1.1127339	7.82E-63	1.43E-58	0.886	0.997
MGP	3	-1.1352368	1.75E-57	3.19E-53	0.974	1
NEAT1	3	-1.1699854	2.81E-76	5.12E-72	0.969	1
MT-ND2	3	-1.1733666	1.10E-56	2.01E-52	0.492	0.885
GPX3	3	-1.1779882	3.38E-73	6.17E-69	0.729	0.997
MT-ATP6	3	-1.1784809	2.21E-60	4.04E-56	0.675	0.941
ID3	3	-1.1888447	8.19E-57	1.50E-52	0.223	0.774
ADIRF	3	-1.245136	1.14E-74	2.08E-70	0.216	0.854
C2orf82	3	-1.2454916	7.13E-62	1.30E-57	0.85	0.979
MT-CYB	3	-1.2477814	1.84E-65	3.36E-61	0.527	0.913
CLEC3A	3	-1.2658561	7.57E-84	1.38E-79	0.033	0.721
PLAC9	3	-1.2979173	4.03E-88	7.36E-84	0.42	0.976
SLPI	3	-1.4647916	4.56E-65	8.32E-61	0.765	1
FRZB	3	-1.4971478	5.63E-53	1.03E-48	0.124	0.659
OGN	3	-1.5198907	1.74E-68	3.17E-64	0.42	0.927
**gene**	**cluster**	**avg_logFC**	**p_val**	**p_val_adj**	**pct.1**	**pct.2**
MT2A	4	2.23586763	3.67E-49	6.70E-45	1	0.989
PRG4	4	1.95137271	1.92E-44	3.51E-40	0.667	0.097
TGFBI	4	1.46522699	1.50E-50	2.74E-46	0.865	0.284

SPP1	4	1.4374114	3.21E-68	5.86E-64	0.769	0.046
IGFBP3	4	1.3016353	1.23E-29	2.25E-25	0.615	0.154
CTHRC1	4	1.16561503	3.31E-39	6.05E-35	0.615	0.105
COMP	4	1.11912911	3.93E-27	7.17E-23	0.936	0.684
SOD2	4	1.09865118	5.86E-23	1.07E-18	0.942	0.738
IL11	4	1.07044141	1.93E-23	3.52E-19	0.25	0
LINC00152	4	1.05858911	1.41E-25	2.58E-21	0.846	0.549
MT1E	4	1.05701839	5.84E-24	1.07E-19	1	0.957
MT1G	4	1.02456717	4.53E-14	8.26E-10	0.955	0.835
MT1X	4	1.01595509	4.75E-22	8.67E-18	1	0.986
SERPINE2	4	1.00245752	3.67E-28	6.69E-24	1	0.986
FN1	4	0.97981597	1.99E-40	3.64E-36	1	1
COL1A2	4	0.8950369	5.50E-10	1.00E-05	0.635	0.435
MT1H	4	0.87978204	1.31E-07	0.00239581	0.679	0.476
CYP1B1	4	0.83358693	1.10E-12	2.01E-08	0.397	0.146
PTGS2	4	0.82432345	2.13E-35	3.89E-31	0.494	0.049
SLC25A37	4	0.79590648	1.38E-26	2.53E-22	0.91	0.595
TNFRSF12A	4	0.75942349	4.01E-16	7.32E-12	0.673	0.373
MT1M	4	0.75873501	1.51E-16	2.76E-12	0.75	0.47
SORBS2	4	0.71654688	5.02E-16	9.17E-12	0.397	0.111
VMP1	4	0.70782826	7.46E-22	1.36E-17	0.821	0.511
SAT1	4	0.69883902	3.10E-13	5.66E-09	0.955	0.935
RND3	4	0.69062783	8.27E-16	1.51E-11	0.436	0.141
MIR4435-2HG	4	0.68955005	5.83E-17	1.06E-12	0.673	0.373
CXCL8	4	0.68611396	8.21E-16	1.50E-11	0.212	0.014
PHLDA1	4	0.68132544	2.56E-18	4.67E-14	0.321	0.049
S100A2	4	0.68029927	5.58E-10	1.02E-05	0.609	0.362
IER3	4	0.674405	4.11E-12	7.50E-08	0.449	0.181
CTSL	4	0.66841224	4.32E-15	7.88E-11	0.622	0.327
BTG1	4	0.66689461	6.15E-18	1.12E-13	0.994	0.943
UPP1	4	0.66376031	3.19E-23	5.82E-19	0.513	0.127
VEGFA	4	0.66063085	1.51E-19	2.76E-15	0.795	0.432
CTNNB1	4	0.65656088	7.79E-08	0.00142175	0.635	0.441
BMP2	4	0.64310423	5.98E-19	1.09E-14	0.288	0.03
TM4SF1	4	0.64123098	5.04E-13	9.20E-09	0.763	0.486
TMSB4X	4	0.64093864	5.25E-09	9.58E-05	0.942	0.865
STK17A	4	0.63174427	2.32E-18	4.24E-14	0.474	0.149
THBS1	4	0.61645629	1.09E-10	1.98E-06	0.462	0.205
CCDC85B	4	0.61336191	1.52E-14	2.77E-10	0.897	0.738

LTBP1	4	0.6115921	3.98E-40	7.26E-36	0.506	0.03
CD44	4	0.60871179	1.08E-23	1.96E-19	0.885	0.505
PMP22	4	0.59655822	8.23E-15	1.50E-10	0.827	0.608
WTAP	4	0.58921015	8.41E-13	1.54E-08	0.769	0.541
CD55	4	0.5829513	3.20E-13	5.84E-09	0.571	0.303
COL3A1	4	0.57688278	1.82E-06	0.03328718	0.622	0.47
HMGA1	4	0.56535072	9.31E-14	1.70E-09	0.423	0.146
SMOX	4	0.56247801	2.00E-07	0.00364964	0.301	0.13
BZW1	4	0.55804141	7.59E-13	1.39E-08	0.731	0.476
AEBP1	4	0.55711152	4.39E-15	8.01E-11	0.885	0.714
PPP3CA	4	0.55367702	2.07E-12	3.77E-08	0.846	0.643
TNC	4	0.5497167	1.30E-34	2.37E-30	0.455	0.03
CHI3L2	4	0.54929668	1.47E-07	0.00268757	0.577	0.389
C11orf96	4	0.54779405	7.97E-19	1.45E-14	0.288	0.03
LOX	4	0.54673529	1.29E-12	2.36E-08	0.75	0.497
CRLF1	4	0.53949525	2.55E-21	4.66E-17	0.622	0.232
GJA1	4	0.53907704	6.94E-17	1.27E-12	0.519	0.197
PLAUR	4	0.53566939	3.39E-22	6.18E-18	0.494	0.119
EVI2A	4	0.52413986	2.69E-27	4.90E-23	0.455	0.065
CCND1	4	0.52070225	4.38E-15	7.98E-11	0.558	0.241
ADAMTS6	4	0.50554958	1.72E-17	3.14E-13	0.365	0.078
SERPINE1	4	0.5017227	1.85E-14	3.37E-10	0.237	0.03
FAP	4	0.48588449	7.84E-22	1.43E-17	0.558	0.173
ERRFI1	4	0.46649795	2.14E-06	0.03904397	0.391	0.224
RCAN1	4	0.46153198	4.67E-11	8.51E-07	0.372	0.132
NRP2	4	0.46046761	2.19E-14	4.00E-10	0.436	0.149
PDE4B	4	0.4531441	2.68E-20	4.90E-16	0.385	0.068
SLC20A1	4	0.45213958	7.84E-14	1.43E-09	0.359	0.097
FOSL1	4	0.44907317	1.39E-19	2.54E-15	0.321	0.041
GSTO1	4	0.44685674	6.99E-08	0.00127478	0.686	0.505
DDIT4L	4	0.44175442	3.19E-08	0.00058205	0.455	0.241
TMSB10	4	0.43524282	5.75E-11	1.05E-06	1	0.997
PPP1CB	4	0.43232222	4.77E-07	0.00871143	0.833	0.746
RABGGTB	4	0.43160225	8.77E-11	1.60E-06	0.679	0.443
NT5E	4	0.42181425	4.19E-10	7.65E-06	0.679	0.451
CYTL1	4	0.42111076	1.05E-08	0.00019124	0.423	0.195
ERO1A	4	0.41215607	2.21E-08	0.00040241	0.615	0.441
PTP4A1	4	0.41062048	1.96E-07	0.00356893	0.558	0.386
ITGB1	4	0.40913981	4.50E-08	0.00082134	0.737	0.641

FSTL1	4	0.40772608	7.85E-08	0.00143253	0.679	0.522
WSB1	4	0.40077577	4.81E-09	8.78E-05	0.853	0.719
SMOC1	4	0.39980988	3.62E-13	6.60E-09	0.538	0.254
SLC2A1	4	0.39784997	2.56E-07	0.00468015	0.327	0.151
FHL2	4	0.39406433	1.26E-08	0.00022987	0.429	0.219
TGM2	4	0.39317706	3.26E-09	5.95E-05	0.353	0.149
SGIP1	4	0.38569631	1.77E-25	3.23E-21	0.346	0.024
RNF145	4	0.37699174	6.76E-09	0.00012339	0.571	0.354
MIF	4	0.36981196	1.06E-15	1.94E-11	1	1
CLEC11A	4	0.36520543	2.17E-07	0.00395355	0.635	0.424
COL6A3	4	0.35389434	4.38E-08	0.00079914	0.622	0.419
CNIH1	4	0.35323129	3.44E-08	0.00062792	0.782	0.673
ISG20	4	0.34987369	7.29E-09	0.00013297	0.404	0.189
SPRY2	4	0.3426172	2.02E-10	3.68E-06	0.256	0.065
TFPI	4	0.33301875	5.87E-08	0.00107116	0.205	0.054
VAPA	4	0.33084247	1.96E-07	0.00358028	0.936	0.811
COL1A1	4	0.32502936	8.35E-12	1.52E-07	0.167	0.014
ABL2	4	0.31360066	3.88E-07	0.0070821	0.359	0.176
SMIM3	4	0.29713247	2.31E-14	4.21E-10	0.34	0.081
HLA-DRB1	4	0.29566296	1.42E-07	0.00259537	0.385	0.186
NGF	4	0.28971737	6.81E-14	1.24E-09	0.263	0.043
HIF1A	4	0.28678022	1.52E-06	0.02779777	0.878	0.824
RELB	4	0.2860127	5.79E-14	1.06E-09	0.282	0.051
CLK1	4	0.28549123	7.05E-07	0.01286418	0.564	0.359
BRI3	4	0.2828664	1.43E-08	0.00026049	0.955	0.862
TREM1	4	0.28172545	3.12E-14	5.69E-10	0.186	0.011
CRTAC1	4	0.27877579	7.95E-08	0.0014509	0.282	0.105
CHMP1B	4	0.27759795	2.14E-06	0.03913651	0.404	0.219
MEDAG	4	0.27533388	7.65E-15	1.40E-10	0.237	0.027
PPP1R14C	4	0.27243986	4.88E-09	8.91E-05	0.288	0.095
HMGA2	4	0.27210999	2.52E-15	4.61E-11	0.199	0.011
KIFC3	4	0.26647986	5.93E-12	1.08E-07	0.333	0.095
SGK1	4	0.26452877	4.70E-09	8.57E-05	0.314	0.111
FZD8	4	0.25827543	7.03E-09	0.0001282	0.385	0.162
CXCL14	4	0.25795912	1.66E-09	3.04E-05	0.115	0.005
EPS8	4	0.25751119	1.11E-07	0.00201676	0.333	0.141
C11orf31	4	0.25354922	9.03E-07	0.01647044	0.814	0.678
MIR222HG	4	0.25273792	1.20E-08	0.00021822	0.179	0.035
USMG5	4	0.25138221	4.55E-08	0.00083095	0.942	0.897

FRMD6	4	0.25122303	3.96E-07	0.00723245	0.391	0.192
DSTN	4	-0.2507648	1.87E-09	3.42E-05	1	1
GABARAPL2	4	-0.2519336	1.33E-07	0.00243462	0.795	0.916
RPL18A	4	-0.2524865	1.62E-17	2.96E-13	1	1
GSTA4	4	-0.2526336	5.88E-07	0.010737	0.192	0.403
ENKUR	4	-0.2550867	4.21E-09	7.68E-05	0.051	0.278
COPZ2	4	-0.2552265	2.03E-06	0.03708919	0.333	0.554
CSTB	4	-0.2557652	3.45E-10	6.30E-06	0.981	0.997
PRNP	4	-0.2557827	2.33E-07	0.004259	0.25	0.486
RPS4X	4	-0.2558975	6.41E-17	1.17E-12	1	1
POLD2	4	-0.2564715	1.44E-07	0.00263354	0.167	0.392
RPS18	4	-0.2568828	1.01E-19	1.85E-15	1	1
BTF3	4	-0.2569023	8.05E-12	1.47E-07	1	0.997
ZFHX4	4	-0.2569442	7.01E-08	0.00127956	0.128	0.362
BHLHE41	4	-0.2573126	7.17E-07	0.01307999	0.321	0.562
NCOR1	4	-0.258243	3.54E-08	0.00064592	0.167	0.411
SOX9	4	-0.2587194	4.09E-07	0.00746347	0.654	0.859
PTMS	4	-0.2595741	2.71E-07	0.00495095	0.635	0.851
GLO1	4	-0.2597805	8.40E-08	0.00153246	0.237	0.476
SSPN	4	-0.2613374	2.17E-06	0.03961122	0.321	0.538
PRUNE2	4	-0.2614672	1.89E-06	0.03455273	0.256	0.476
JTB	4	-0.2617951	2.98E-08	0.00054319	0.673	0.868
RP11-983P16.4	4	-0.2620856	2.70E-07	0.004927	0.212	0.438
CPE	4	-0.2625456	2.66E-06	0.04859559	0.494	0.67
CMB9-22P13.1	4	-0.2628001	7.26E-07	0.0132505	0.404	0.611
RPL11	4	-0.2630394	1.97E-19	3.60E-15	1	1
RPL31	4	-0.2631179	2.73E-16	4.98E-12	1	1
CKAP4	4	-0.2634167	7.26E-08	0.00132516	0.288	0.551
COX7C	4	-0.2635902	1.63E-14	2.97E-10	1	1
CYBRD1	4	-0.2637673	3.04E-07	0.00555236	0.577	0.757
SNX3	4	-0.2638702	9.65E-10	1.76E-05	0.974	0.997
TOMM7	4	-0.2653911	9.03E-16	1.65E-11	1	1
RPS3	4	-0.2665447	2.07E-20	3.77E-16	1	1
GTF2A2	4	-0.2683523	4.55E-08	0.00083032	0.282	0.541
BBX	4	-0.2687034	5.46E-08	0.00099703	0.244	0.495
RPL22	4	-0.268832	3.44E-14	6.28E-10	1	1
RPL15	4	-0.2691895	1.20E-23	2.19E-19	1	1
PRDX5	4	-0.2695948	9.78E-08	0.00178501	0.788	0.941
VIM	4	-0.2698336	2.30E-09	4.20E-05	1	1

RNF130	4	-0.2727605	3.28E-07	0.0059783	0.321	0.546
SMIM5	4	-0.274407	1.75E-10	3.20E-06	0.077	0.354
TCEAL2	4	-0.2745744	8.86E-08	0.00161696	0.218	0.457
PLA2G16	4	-0.2751454	1.54E-07	0.0028086	0.173	0.4
RPL5	4	-0.2760318	1.17E-14	2.13E-10	1	1
CCNG1	4	-0.2761234	1.04E-07	0.00190105	0.378	0.619
RPS4Y1	4	-0.2762799	2.21E-10	4.03E-06	0.962	0.995
RPL10	4	-0.2764432	1.06E-21	1.93E-17	1	1
H3F3A	4	-0.2765811	1.66E-12	3.02E-08	0.981	0.997
ILF3-AS1	4	-0.2778054	2.68E-08	0.00048882	0.218	0.473
IDI1	4	-0.2778509	1.88E-06	0.03435889	0.263	0.465
PCBP2	4	-0.2782005	7.96E-09	0.00014532	0.929	0.97
MLEC	4	-0.2787459	1.56E-07	0.00283823	0.288	0.53
TCEAL8	4	-0.2791714	1.31E-07	0.0023864	0.647	0.846
RPL35A	4	-0.279186	8.21E-21	1.50E-16	1	1
PIM3	4	-0.2794877	2.70E-07	0.00492355	0.212	0.454
KAZALD1	4	-0.2796655	2.86E-08	0.00052138	0.103	0.332
UGP2	4	-0.2796821	1.60E-07	0.00291247	0.59	0.77
MPC2	4	-0.2797793	1.72E-07	0.00313577	0.385	0.614
DPYSL3	4	-0.2804277	1.76E-10	3.21E-06	0.096	0.37
RPS7	4	-0.2804742	2.91E-16	5.30E-12	1	1
EEF1D	4	-0.2815436	1.02E-14	1.85E-10	1	1
CALM2	4	-0.2834442	9.60E-10	1.75E-05	0.936	0.992
RPA3	4	-0.2839547	1.03E-06	0.01871955	0.404	0.603
HMGN2	4	-0.2850233	2.20E-11	4.02E-07	0.795	0.951
CCDC14	4	-0.2866004	4.95E-08	0.00090306	0.199	0.449
RPL23	4	-0.2871438	1.79E-16	3.27E-12	1	1
ERGIC3	4	-0.2872882	8.89E-10	1.62E-05	0.795	0.951
TTC3	4	-0.2902119	1.23E-09	2.24E-05	0.731	0.916
SMIM19	4	-0.2902445	4.43E-08	0.0008075	0.308	0.549
SH3BGRL	4	-0.2914775	3.97E-08	0.0007236	0.442	0.662
TPD52L1	4	-0.2926084	5.67E-08	0.00103416	0.173	0.408
ACTG1	4	-0.2946267	3.28E-08	0.0005986	0.923	0.984
NDUFA4L2	4	-0.2954795	6.32E-15	1.15E-10	1	1
CLTB	4	-0.2978055	1.76E-08	0.00032115	0.314	0.568
LTA4H	4	-0.2980493	8.49E-07	0.01549515	0.295	0.505
DDR2	4	-0.2999701	1.37E-08	0.00024939	0.417	0.692
FIS1	4	-0.3003931	1.16E-08	0.00021137	0.519	0.732
RPL3	4	-0.3006306	1.76E-20	3.21E-16	1	1

INHBA	4	-0.301444	3.70E-08	0.00067487	0.494	0.754
HNRNPA1	4	-0.3016134	2.56E-08	0.00046669	0.962	0.997
RPL21	4	-0.303391	2.31E-26	4.21E-22	1	1
KDELR2	4	-0.3034025	2.01E-09	3.66E-05	0.827	0.965
NUCB2	4	-0.304523	1.16E-06	0.02108249	0.353	0.557
ITM2B	4	-0.3048862	1.08E-06	0.01970784	0.904	0.976
ISM1	4	-0.3051395	1.74E-09	3.17E-05	0.147	0.419
PRDX2	4	-0.3055829	9.84E-08	0.00179498	0.571	0.765
SSR3	4	-0.3057697	2.29E-09	4.18E-05	0.833	0.951
TMEM59	4	-0.3057913	3.45E-07	0.00629111	0.878	0.959
SDF4	4	-0.3064243	2.43E-07	0.004436	0.513	0.7
KDELR3	4	-0.306759	5.40E-09	9.86E-05	0.237	0.503
PPIC	4	-0.308371	1.40E-08	0.00025586	0.571	0.811
TMEM106C	4	-0.3088186	6.46E-10	1.18E-05	0.192	0.459
PRDX1	4	-0.3089071	4.32E-09	7.88E-05	0.744	0.884
HOXB6	4	-0.3093239	3.87E-07	0.00706173	0.186	0.397
TMEM98	4	-0.3097665	2.34E-09	4.28E-05	0.269	0.535
ATRAID	4	-0.3105603	4.46E-08	0.00081404	0.731	0.868
MDFI	4	-0.3118906	1.40E-07	0.002559	0.558	0.776
MAP1B	4	-0.3120612	3.04E-09	5.55E-05	0.115	0.373
TUBB4B	4	-0.3125004	7.02E-09	0.00012815	0.25	0.527
MKX	4	-0.31337	1.31E-10	2.39E-06	0.167	0.459
HLA-B	4	-0.3170848	5.05E-07	0.00920984	0.635	0.789
DBP	4	-0.3172034	4.52E-09	8.25E-05	0.109	0.354
ARL6IP5	4	-0.3173842	9.58E-09	0.00017478	0.571	0.751
RPLP1	4	-0.3175613	2.55E-31	4.66E-27	1	1
EEF1B2	4	-0.318253	6.37E-12	1.16E-07	0.962	0.997
MALAT1	4	-0.3189017	5.98E-08	0.00109042	1	1
EDF1	4	-0.324175	3.36E-15	6.14E-11	0.949	0.973
MPP6	4	-0.3242634	5.88E-10	1.07E-05	0.147	0.419
PSAP	4	-0.3253585	1.34E-10	2.45E-06	0.788	0.941
CRIP2	4	-0.3257672	9.89E-08	0.00180448	0.417	0.632
RPL13	4	-0.3279908	9.26E-32	1.69E-27	1	1
RPS27	4	-0.3280009	1.61E-26	2.93E-22	1	1
HIF1A-AS2	4	-0.3281591	9.82E-07	0.01791764	0.263	0.478
LITAF	4	-0.3284232	1.01E-08	0.0001835	0.474	0.689
RPL19	4	-0.3286366	5.67E-30	1.03E-25	1	1
YBX3	4	-0.3287528	1.01E-09	1.84E-05	0.756	0.932
RPL37	4	-0.3288432	4.31E-26	7.87E-22	1	1

MT-ND5	4	-0.3290846	4.10E-07	0.00748836	0.603	0.765
RPS25	4	-0.3291555	3.17E-26	5.78E-22	1	1
RPS12	4	-0.330229	6.41E-24	1.17E-19	1	1
BEX4	4	-0.3302755	1.91E-09	3.48E-05	0.436	0.695
NENF	4	-0.3311598	2.21E-12	4.03E-08	0.891	0.97
RPS5	4	-0.3327327	5.65E-24	1.03E-19	1	1
ZBTB20	4	-0.333053	6.87E-09	0.00012531	0.269	0.522
MT-ND4	4	-0.3335781	1.80E-09	3.28E-05	0.987	0.989
RPSA	4	-0.3350003	1.26E-16	2.31E-12	0.994	1
SOD1	4	-0.3354904	1.01E-10	1.85E-06	0.846	0.965
RPS19	4	-0.3357362	7.12E-26	1.30E-21	1	1
CCNI	4	-0.3367759	2.64E-15	4.82E-11	1	1
RPS9	4	-0.3369287	9.72E-28	1.77E-23	1	1
RPS23	4	-0.3374707	1.33E-29	2.43E-25	1	1
GOLIM4	4	-0.3377362	5.96E-07	0.01087091	0.397	0.649
MT-CO2	4	-0.3398565	1.75E-10	3.20E-06	0.962	0.995
COMMD6	4	-0.3407194	2.28E-20	4.16E-16	0.987	1
RPS13	4	-0.3421818	1.20E-23	2.19E-19	1	1
ARL2BP	4	-0.342896	9.91E-10	1.81E-05	0.429	0.703
JUN	4	-0.3430395	1.51E-07	0.00276007	0.135	0.357
ATP5O	4	-0.3450906	8.05E-12	1.47E-07	0.673	0.846
NDUFA11	4	-0.3464689	1.88E-13	3.42E-09	0.897	0.976
RBP4	4	-0.3493756	9.89E-09	0.00018047	0.654	0.892
TSPO	4	-0.3493756	2.97E-11	5.42E-07	0.763	0.943
RPS6	4	-0.3504651	3.70E-30	6.74E-26	1	1
RPLP2	4	-0.3511433	2.53E-31	4.62E-27	1	1
TSPAN3	4	-0.3517646	2.87E-10	5.24E-06	0.327	0.608
LDHB	4	-0.3536093	6.17E-10	1.13E-05	0.654	0.835
TCEA1	4	-0.3538265	1.88E-07	0.0034235	0.583	0.746
HINT1	4	-0.3538646	5.52E-18	1.01E-13	0.981	0.997
PABPC1	4	-0.3546733	2.35E-12	4.29E-08	0.782	0.962
C14orf1	4	-0.3553637	5.51E-11	1.01E-06	0.231	0.522
RP11-71N10.1	4	-0.3554547	1.85E-12	3.38E-08	0.038	0.33
RPL8	4	-0.3564232	6.38E-32	1.16E-27	1	1
RARRES3	4	-0.3597561	5.86E-16	1.07E-11	0.013	0.349
PDIA6	4	-0.3612072	5.71E-10	1.04E-05	0.577	0.786
NAP1L1	4	-0.3618017	6.27E-13	1.14E-08	0.904	0.97
HLA-C	4	-0.3650184	3.98E-09	7.26E-05	0.744	0.914
SRP14	4	-0.3669076	1.02E-16	1.87E-12	0.949	0.997

NEAT1	4	-0.3672583	2.38E-08	0.00043445	1	1
CALM3	4	-0.3673264	6.17E-11	1.13E-06	0.494	0.762
TBL1XR1	4	-0.3680921	5.17E-07	0.00942888	0.519	0.716
SPINT2	4	-0.3692863	4.69E-10	8.57E-06	0.237	0.524
RPS29	4	-0.371438	9.17E-32	1.67E-27	1	1
LRRC2	4	-0.3733123	3.10E-15	5.66E-11	0.045	0.386
ITM2C	4	-0.3744503	3.93E-09	7.17E-05	0.513	0.751
AP2M1	4	-0.3747272	1.39E-10	2.54E-06	0.372	0.643
SLC14A1	4	-0.3755472	1.88E-08	0.00034261	0.282	0.516
MORF4L2	4	-0.3756326	1.65E-11	3.02E-07	0.596	0.808
EID1	4	-0.3786197	1.12E-15	2.04E-11	0.878	0.986
RPL23A	4	-0.3795857	5.51E-32	1.00E-27	1	1
ENAH	4	-0.3821693	2.74E-12	4.99E-08	0.333	0.643
AC003090.1	4	-0.3827262	8.26E-18	1.51E-13	0.006	0.37
ZFAS1	4	-0.3829075	3.80E-11	6.94E-07	0.955	0.997
NKX3-2	4	-0.3833189	2.23E-11	4.06E-07	0.083	0.37
PKM	4	-0.3839718	1.13E-12	2.06E-08	0.776	0.951
MYL6	4	-0.384635	2.14E-14	3.91E-10	0.917	0.984
HSP90AB1	4	-0.3846417	9.42E-13	1.72E-08	0.859	0.962
RPS17	4	-0.3864615	4.85E-31	8.85E-27	1	1
CALM1	4	-0.389685	1.04E-10	1.90E-06	0.378	0.67
MT-CO3	4	-0.3921544	1.10E-11	2.00E-07	0.949	0.989
EIF4B	4	-0.3934837	3.05E-13	5.57E-09	0.686	0.881
ID3	4	-0.3951093	1.41E-12	2.58E-08	0.641	0.935
PPP1R3C	4	-0.3954579	5.65E-11	1.03E-06	0.365	0.649
BEX2	4	-0.3956626	6.05E-13	1.10E-08	0.09	0.408
DMKN	4	-0.3962658	1.87E-12	3.41E-08	0.321	0.616
MFGE8	4	-0.397529	2.46E-09	4.49E-05	0.641	0.835
SOD3	4	-0.397859	1.08E-07	0.00196723	0.872	0.946
HSPA8	4	-0.4005036	5.18E-10	9.45E-06	0.333	0.605
RDX	4	-0.400812	3.81E-10	6.95E-06	0.449	0.692
RPL10A	4	-0.4016746	2.74E-29	5.00E-25	1	1
OSBPL9	4	-0.4019039	1.93E-11	3.53E-07	0.333	0.622
NINJ1	4	-0.4022086	1.49E-13	2.72E-09	0.365	0.73
SLC38A1	4	-0.4035614	2.80E-13	5.10E-09	0.288	0.635
ST13	4	-0.4045549	1.14E-14	2.08E-10	0.853	0.959
MSMO1	4	-0.4053525	1.25E-10	2.28E-06	0.173	0.476
FXYD6	4	-0.4058658	9.57E-16	1.75E-11	0.782	0.943
CREG1	4	-0.4062221	3.12E-13	5.69E-09	0.244	0.584

RPL13A	4	-0.407605	1.21E-38	2.21E-34	1	1
COL2A1	4	-0.4078539	1.57E-11	2.87E-07	0.647	0.957
MOXD1	4	-0.4121408	4.72E-14	8.61E-10	0.16	0.503
INSIG1	4	-0.4134867	2.00E-06	0.03651886	0.314	0.519
RP5-1097P24.1	4	-0.4166828	1.12E-11	2.05E-07	0.173	0.465
RPL18	4	-0.4170197	1.23E-30	2.25E-26	1	1
VCAN	4	-0.4205709	8.62E-07	0.01572028	0.513	0.681
TPT1	4	-0.4278187	1.68E-32	3.06E-28	1	1
MT-CO1	4	-0.4293079	4.20E-14	7.66E-10	0.974	0.986
RPL9	4	-0.4314338	6.59E-38	1.20E-33	1	1
SEC11A	4	-0.4335986	5.50E-17	1.00E-12	0.731	0.927
RPL34	4	-0.4339851	2.66E-35	4.85E-31	1	1
RPS27A	4	-0.4365514	3.16E-34	5.76E-30	1	1
RPL30	4	-0.4406738	2.76E-34	5.03E-30	1	1
GSTP1	4	-0.4410561	4.92E-14	8.97E-10	0.865	0.954
RPS16	4	-0.4429952	1.68E-39	3.06E-35	1	1
C2orf40	4	-0.4449537	9.52E-12	1.74E-07	0.974	0.997
HLA-A	4	-0.4469355	8.01E-13	1.46E-08	0.75	0.911
PEBP1	4	-0.4508285	2.32E-15	4.23E-11	0.679	0.905
RPS3A	4	-0.452627	3.48E-38	6.35E-34	1	1
GADD45B	4	-0.4545939	1.43E-07	0.00261302	0.224	0.446
NFIA	4	-0.4547851	9.26E-16	1.69E-11	0.276	0.657
DNM3OS	4	-0.4548853	6.77E-12	1.23E-07	0.462	0.732
AHNAK2	4	-0.4570931	2.28E-11	4.15E-07	0.205	0.503
AFF3	4	-0.461571	1.36E-18	2.48E-14	0.096	0.505
OAF	4	-0.4634422	3.39E-15	6.18E-11	0.167	0.527
DYNLL1	4	-0.4658773	5.86E-14	1.07E-09	0.788	0.938
S100B	4	-0.4663289	4.72E-12	8.62E-08	0.635	0.859
PFN2	4	-0.4665388	8.22E-16	1.50E-11	0.41	0.749
MT-ND1	4	-0.466789	4.02E-11	7.33E-07	0.705	0.908
COL9A3	4	-0.467151	2.80E-13	5.12E-09	0.5	0.811
TCF4	4	-0.4672699	4.57E-13	8.33E-09	0.494	0.776
SPON2	4	-0.4673682	1.10E-13	2.01E-09	0.071	0.395
RPL4	4	-0.4687207	2.76E-29	5.03E-25	1	1
ITM2A	4	-0.4783745	1.45E-13	2.65E-09	0.365	0.692
PDE4DIP	4	-0.4812621	1.29E-15	2.36E-11	0.34	0.668
NPC2	4	-0.4934003	2.22E-27	4.05E-23	0.955	0.995
RHOBTB3	4	-0.4968632	1.03E-15	1.88E-11	0.494	0.762
UMAD1	4	-0.5014751	3.32E-17	6.07E-13	0.135	0.519

TUBB	4	-0.5038675	7.54E-16	1.38E-11	0.731	0.922
EIF3L	4	-0.5042438	3.70E-22	6.76E-18	0.712	0.957
ATRNL1	4	-0.5103437	3.63E-18	6.62E-14	0.154	0.554
RPLP0	4	-0.5105699	2.95E-40	5.39E-36	1	1
TMED10	4	-0.5107566	2.71E-20	4.94E-16	0.763	0.943
GREM1	4	-0.5120492	2.43E-09	4.43E-05	0.026	0.249
CCDC102B	4	-0.5141847	6.33E-19	1.16E-14	0.122	0.535
CST3	4	-0.5178613	2.97E-21	5.43E-17	0.994	1
SNAI2	4	-0.5181731	7.52E-11	1.37E-06	0.564	0.768
FGFBP2	4	-0.5197019	7.15E-09	0.00013044	0.814	0.943
FOS	4	-0.5217071	1.09E-14	1.99E-10	0.372	0.697
RPS8	4	-0.5330237	1.57E-46	2.87E-42	1	1
MYLK	4	-0.5393465	7.36E-18	1.34E-13	0.167	0.57
PTRF	4	-0.5464921	1.54E-21	2.81E-17	0.468	0.808
NUPR1	4	-0.5497565	6.85E-15	1.25E-10	0.923	0.97
RPL7	4	-0.5527992	4.99E-41	9.11E-37	1	1
LINC01503	4	-0.5540636	1.27E-16	2.31E-12	0.295	0.676
MT-ATP6	4	-0.5583399	3.53E-19	6.45E-15	0.872	0.984
SDC2	4	-0.558545	1.32E-20	2.41E-16	0.833	0.97
APOD	4	-0.5639344	2.25E-12	4.11E-08	0.083	0.384
MT-ND3	4	-0.5652987	6.62E-19	1.21E-14	0.782	0.949
CRTAP	4	-0.5655987	1.85E-23	3.37E-19	0.577	0.895
MSMP	4	-0.5676365	5.01E-07	0.00914565	0.25	0.495
MAGED1	4	-0.5746685	6.80E-12	1.24E-07	0.224	0.535
COL11A2	4	-0.57791	2.57E-16	4.69E-12	0.34	0.692
EEF2	4	-0.6055837	1.55E-25	2.82E-21	0.981	1
MIA	4	-0.6083833	6.75E-20	1.23E-15	0.962	0.995
MT-CYB	4	-0.6091826	3.07E-19	5.59E-15	0.795	0.968
XAGE2	4	-0.6100593	3.55E-09	6.47E-05	0	0.195
CXXC5	4	-0.6179575	6.75E-27	1.23E-22	0.269	0.765
B2M	4	-0.6362634	1.73E-34	3.15E-30	0.987	0.997
CD9	4	-0.6702834	9.37E-18	1.71E-13	0.34	0.695
RPS11	4	-0.6703241	2.72E-47	4.96E-43	0.987	1
ID4	4	-0.6717303	4.49E-21	8.19E-17	0.494	0.841
CRISPLD1	4	-0.6824786	6.98E-16	1.27E-11	0.045	0.389
ISLR	4	-0.6891975	7.41E-25	1.35E-20	0.301	0.762
THY1	4	-0.6906273	4.59E-20	8.37E-16	0.564	0.878
S100A1	4	-0.6979945	1.07E-35	1.95E-31	0.981	1
RPS20	4	-0.7095159	1.67E-52	3.04E-48	0.994	1

EEF1A1	4	-0.7142991	3.08E-55	5.61E-51	1	1
ZFP36L2	4	-0.7154281	1.13E-18	2.06E-14	0.25	0.643
CP	4	-0.725528	4.99E-23	9.11E-19	0.487	0.841
CITED2	4	-0.725744	8.72E-18	1.59E-13	0.372	0.741
LECT1	4	-0.7344205	9.76E-19	1.78E-14	0.641	0.914
ACAN	4	-0.7390942	9.44E-33	1.72E-28	0.885	0.995
ID2	4	-0.7433489	1.37E-18	2.50E-14	0.449	0.808
SLC40A1	4	-0.7503005	1.08E-22	1.97E-18	0.096	0.562
MT-ND2	4	-0.7507559	2.09E-24	3.82E-20	0.679	0.919
ID1	4	-0.7705782	2.14E-19	3.90E-15	0.647	0.954
FHL1	4	-0.7724592	5.62E-25	1.03E-20	0.449	0.814
NGFRAP1	4	-0.8057047	6.86E-39	1.25E-34	0.692	0.97
PAX1	4	-0.8254583	2.84E-34	5.18E-30	0.474	0.911
EIF3H	4	-0.8290048	1.42E-47	2.58E-43	0.91	0.997
PLAC9	4	-0.8307514	1.28E-31	2.34E-27	0.744	0.986
CRYAB	4	-0.8532684	2.18E-25	3.97E-21	0.744	0.943
IGFBP6	4	-0.864261	2.09E-23	3.81E-19	0.481	0.822
FBXO2	4	-0.8645024	2.58E-36	4.70E-32	0.526	0.949
DCN	4	-0.8649619	2.98E-38	5.44E-34	1	1
FTL	4	-0.8667142	3.02E-55	5.50E-51	1	1
TSC22D1	4	-0.8669984	9.34E-23	1.70E-18	0.455	0.824
PLPP1	4	-0.89937	9.26E-32	1.69E-27	0.244	0.757
OGN	4	-0.9816686	3.31E-32	6.04E-28	0.917	0.997
MGP	4	-0.9919701	9.86E-34	1.80E-29	0.994	1
FTH1	4	-1.1053621	3.58E-56	6.53E-52	1	1
SPTSSB	4	-1.1422988	1.18E-27	2.15E-23	0.006	0.524
ADIRF	4	-1.1765738	9.81E-40	1.79E-35	0.256	0.841
C2orf82	4	-1.1951691	3.24E-43	5.91E-39	0.885	0.997
CLEC3A	4	-1.2100382	9.37E-37	1.71E-32	0.167	0.765
SLPI	4	-1.2201154	4.86E-33	8.86E-29	0.821	1
FRZB	4	-1.3775407	1.33E-29	2.42E-25	0.103	0.643
CAPS	4	-1.4418907	1.03E-48	1.88E-44	0.583	0.965
**gene**	**cluster**	**avg_logFC**	**p_val**	**p_val_adj**	**pct.1**	**pct.2**
SPP1	5	3.38171593	4.16E-76	7.60E-72	0.907	0.067
PRG4	5	2.31426809	1.31E-56	2.39E-52	0.678	0.011
MT2A	5	2.23114108	7.83E-55	1.43E-50	1	0.993
COMP	5	1.79077044	7.13E-48	1.30E-43	0.956	0.528
SOD2	5	1.70354017	2.77E-58	5.05E-54	1	0.856
S100A2	5	1.53468767	3.38E-31	6.17E-27	0.688	0.197

UPP1	5	1.33610254	3.88E-65	7.08E-61	0.834	0.088
FN1	5	1.30780515	4.13E-59	7.53E-55	1	0.996
PTGS2	5	1.25173373	8.09E-64	1.48E-59	0.771	0.035
CTSL	5	1.24655103	1.46E-35	2.67E-31	0.868	0.507
IGFBP3	5	1.24425967	5.68E-43	1.04E-38	0.61	0.053
TM4SF1	5	1.24040997	4.45E-33	8.13E-29	0.746	0.292
VEGFA	5	1.18733923	5.98E-53	1.09E-48	0.878	0.25
HMGA1	5	1.16797218	1.46E-49	2.67E-45	0.79	0.162
MT1X	5	1.16619418	6.24E-19	1.14E-14	1	0.982
DDIT4L	5	1.13448359	4.08E-42	7.45E-38	0.932	0.475
BZW1	5	1.10591289	6.76E-51	1.23E-46	0.878	0.306
CD55	5	1.07984601	7.82E-43	1.43E-38	0.917	0.479
STK17A	5	1.0654876	1.94E-45	3.54E-41	0.693	0.099
RGCC	5	1.06504192	7.47E-38	1.36E-33	0.917	0.479
WTAP	5	1.04115354	4.43E-40	8.08E-36	0.917	0.468
MMP3	5	1.02933857	1.06E-15	1.93E-11	0.888	0.665
C11orf96	5	1.009128	3.46E-37	6.31E-33	0.551	0.046
SERPINE2	5	1.00186513	5.71E-22	1.04E-17	1	0.996
TIMP1	5	0.98971387	3.48E-15	6.36E-11	1	1
MRPS6	5	0.97785532	2.02E-25	3.69E-21	0.976	0.923
ERO1A	5	0.97187867	2.37E-40	4.32E-36	0.82	0.335
ORM1	5	0.97130627	1.33E-09	2.43E-05	0.483	0.257
TNFRSF12A	5	0.96650967	1.55E-40	2.83E-36	0.663	0.116
MIF	5	0.90748282	5.31E-67	9.68E-63	1	0.996
ABI3BP	5	0.89617375	2.79E-26	5.09E-22	0.668	0.254
BAALC	5	0.88558005	1.19E-25	2.18E-21	0.629	0.229
PMP22	5	0.87084203	3.19E-36	5.82E-32	0.932	0.616
BMP2	5	0.85659251	9.29E-33	1.70E-28	0.449	0.014
RGS2	5	0.8492124	4.97E-14	9.06E-10	0.668	0.394
TIMP3	5	0.8400105	1.71E-15	3.12E-11	0.82	0.553
TGFBI	5	0.810982	6.05E-29	1.10E-24	0.541	0.102
TREM1	5	0.80034426	4.72E-44	8.61E-40	0.546	0.004
MT1G	5	0.7936158	9.29E-11	1.70E-06	0.932	0.792
CHI3L2	5	0.78411273	6.71E-14	1.22E-09	0.756	0.525
SMOX	5	0.77558873	2.19E-24	4.00E-20	0.571	0.18
NDRG1	5	0.75564363	8.82E-33	1.61E-28	0.785	0.345
SNHG12	5	0.7510462	2.37E-32	4.32E-28	0.737	0.289
PPP3CA	5	0.74346098	1.02E-25	1.87E-21	0.922	0.616
BHLHE40	5	0.72829457	4.06E-21	7.41E-17	0.707	0.352

FAP	5	0.70171289	9.74E-43	1.78E-38	0.595	0.039
SPRY2	5	0.70086672	2.00E-31	3.65E-27	0.493	0.046
SLC3A2	5	0.697616	3.52E-18	6.43E-14	0.595	0.282
ISG20	5	0.69405702	1.48E-23	2.69E-19	0.693	0.324
NRP2	5	0.68727958	2.08E-29	3.80E-25	0.488	0.06
DNER	5	0.67829187	1.10E-26	2.01E-22	0.741	0.31
EIF4EBP1	5	0.67784512	1.36E-19	2.47E-15	0.698	0.391
RNF145	5	0.67649793	2.79E-30	5.09E-26	0.634	0.158
AK2	5	0.67573204	2.54E-28	4.63E-24	0.868	0.489
SLC2A1	5	0.66463189	6.60E-25	1.20E-20	0.527	0.123
TMSB10	5	0.66322505	3.34E-20	6.10E-16	1	0.982
ZNF385D	5	0.6497981	4.69E-24	8.55E-20	0.756	0.38
VAPA	5	0.63401671	1.82E-33	3.33E-29	0.99	0.856
PPFIBP1	5	0.63330298	4.25E-23	7.76E-19	0.683	0.306
CCDC85B	5	0.63126628	2.58E-16	4.71E-12	0.859	0.648
MT1H	5	0.62431408	3.66E-09	6.67E-05	0.654	0.394
KLF3	5	0.60395561	1.13E-19	2.06E-15	0.502	0.148
RABGGTB	5	0.59422825	8.00E-27	1.46E-22	0.863	0.514
DNAJB9	5	0.59376496	3.12E-16	5.69E-12	0.829	0.577
PPP1CB	5	0.59217797	4.36E-15	7.96E-11	0.893	0.676
NPR3	5	0.58235187	5.17E-27	9.44E-23	0.351	0
TMEM45A	5	0.58150126	8.56E-17	1.56E-12	0.902	0.722
PPP1R15A	5	0.57798615	1.18E-27	2.15E-23	0.532	0.102
ZNF593	5	0.57640799	1.84E-23	3.35E-19	0.683	0.296
PLA2G2A	5	0.57438223	2.15E-10	3.92E-06	0.766	0.553
CSGALNACT2	5	0.57410911	4.84E-25	8.84E-21	0.507	0.102
DDIT3	5	0.56817179	2.89E-13	5.28E-09	0.663	0.405
HLA-DRB1	5	0.56690515	1.65E-25	3.01E-21	0.546	0.127
IFRD1	5	0.55661097	5.15E-17	9.39E-13	0.712	0.444
EMP3	5	0.55374819	2.86E-17	5.22E-13	0.907	0.754
PLAUR	5	0.55088664	2.00E-17	3.65E-13	0.488	0.158
SLC25A37	5	0.54910781	8.35E-21	1.52E-16	0.907	0.613
GOLT1B	5	0.54337714	3.82E-21	6.98E-17	0.576	0.204
SGIP1	5	0.54209024	1.32E-23	2.40E-19	0.327	0.007
PTP4A1	5	0.54107564	7.35E-16	1.34E-11	0.649	0.377
RNF114	5	0.53585692	8.03E-24	1.47E-19	0.8	0.493
CCND1	5	0.5344311	4.06E-24	7.40E-20	0.434	0.06
FNIP2	5	0.5330283	3.07E-20	5.59E-16	0.483	0.123
ADSS	5	0.53275867	6.34E-18	1.16E-13	0.717	0.398

EIF1	5	0.53031729	1.25E-40	2.29E-36	1	1
ATP6V1G1	5	0.52586096	1.59E-25	2.91E-21	0.99	0.937
SERPINA1	5	0.52410265	9.62E-10	1.76E-05	0.98	1
GBE1	5	0.51947601	1.08E-15	1.97E-11	0.766	0.514
ARF6	5	0.51752376	3.16E-16	5.77E-12	0.678	0.401
SLC20A1	5	0.51531857	3.57E-21	6.51E-17	0.429	0.081
S100A16	5	0.50779694	5.62E-14	1.03E-09	0.454	0.169
RELB	5	0.49991757	8.34E-24	1.52E-19	0.41	0.046
CLIC4	5	0.49973353	4.81E-15	8.77E-11	0.629	0.363
WDR45B	5	0.4981187	5.76E-19	1.05E-14	0.532	0.173
GPX1	5	0.49374671	9.78E-19	1.79E-14	0.932	0.746
MED21	5	0.49241247	7.52E-19	1.37E-14	0.629	0.275
FAM162A	5	0.4905545	2.50E-20	4.55E-16	0.941	0.754
PID1	5	0.48815705	1.19E-12	2.18E-08	0.673	0.437
SH3BGRL3	5	0.48745173	9.19E-22	1.68E-17	1	0.968
NTMT1	5	0.48545266	1.81E-18	3.30E-14	0.551	0.197
XBP1	5	0.4830154	1.87E-13	3.42E-09	0.746	0.472
GPRC5A	5	0.48196851	1.23E-22	2.25E-18	0.429	0.063
CDC37L1	5	0.47738642	6.60E-20	1.20E-15	0.615	0.246
GDF15	5	0.47664873	7.59E-11	1.38E-06	0.185	0.018
GSTO1	5	0.47651911	1.08E-14	1.97E-10	0.829	0.602
WSB1	5	0.47480989	2.43E-13	4.43E-09	0.859	0.623
TRMT112	5	0.47471827	3.47E-20	6.34E-16	0.927	0.887
WDR33	5	0.47082215	1.79E-15	3.27E-11	0.659	0.373
VIMP	5	0.46740999	2.05E-17	3.74E-13	0.893	0.609
GRB10	5	0.46648869	4.19E-23	7.65E-19	0.312	0.004
ENO2	5	0.4648007	1.60E-18	2.91E-14	0.61	0.257
RHOD	5	0.46402449	8.58E-18	1.56E-13	0.566	0.218
CTHRC1	5	0.4578918	1.87E-16	3.41E-12	0.371	0.074
TMEM38B	5	0.45658444	5.10E-15	9.30E-11	0.673	0.37
PFDN2	5	0.45461697	1.01E-16	1.84E-12	0.776	0.496
EIF5	5	0.45434543	4.37E-15	7.97E-11	0.756	0.458
SLC5A3	5	0.44497837	5.58E-07	0.01018817	0.532	0.366
CLK1	5	0.44326198	8.42E-15	1.54E-10	0.712	0.426
IER3	5	0.44268853	1.63E-15	2.98E-11	0.371	0.085
COX7A2	5	0.4415765	1.09E-22	1.99E-18	0.985	0.954
EPS8	5	0.43622075	3.99E-19	7.28E-15	0.488	0.137
FOSL1	5	0.43063488	3.43E-17	6.25E-13	0.278	0.021
SERPINE1	5	0.42928187	3.70E-19	6.75E-15	0.332	0.035

NT5E	5	0.42926383	1.17E-14	2.13E-10	0.449	0.144
DAB2	5	0.42618338	3.87E-11	7.06E-07	0.429	0.176
CD44	5	0.42593128	6.02E-15	1.10E-10	0.907	0.725
ANXA5	5	0.42579772	5.03E-13	9.18E-09	0.878	0.732
CRLF1	5	0.42411132	8.14E-17	1.48E-12	0.415	0.099
SAR1A	5	0.41847663	3.53E-15	6.44E-11	0.673	0.373
ARPP19	5	0.41750422	3.39E-13	6.18E-09	0.688	0.44
BCAT1	5	0.41467487	7.74E-12	1.41E-07	0.741	0.475
PDE4B	5	0.41457181	1.94E-19	3.53E-15	0.41	0.074
NBPF14	5	0.41379497	1.21E-17	2.21E-13	0.38	0.074
NOL7	5	0.41315639	6.51E-13	1.19E-08	0.683	0.437
SH3GLB1	5	0.41057352	5.35E-15	9.76E-11	0.795	0.525
HMGA2	5	0.41004738	6.77E-24	1.23E-19	0.322	0.004
SIVA1	5	0.40775998	5.12E-11	9.34E-07	0.932	0.789
TMSB4X	5	0.40510158	1.09E-06	0.01996852	0.8	0.683
CHMP4B	5	0.40503278	1.10E-12	2.02E-08	0.693	0.43
SELK	5	0.4029739	6.84E-15	1.25E-10	0.927	0.75
PHLDA1	5	0.40228653	2.01E-19	3.66E-15	0.307	0.021
SVIP	5	0.3983941	6.25E-15	1.14E-10	0.678	0.363
RHEB	5	0.39785962	2.19E-12	4.00E-08	0.888	0.697
TGM2	5	0.397211	4.89E-14	8.92E-10	0.395	0.113
COX17	5	0.39526909	6.04E-12	1.10E-07	0.844	0.637
VDAC1	5	0.3942835	7.51E-12	1.37E-07	0.868	0.761
SQSTM1	5	0.39368243	9.75E-15	1.78E-10	0.98	0.877
ARF4	5	0.39265563	1.68E-09	3.06E-05	0.849	0.655
PELI1	5	0.38658888	4.22E-15	7.71E-11	0.332	0.063
ZFAND5	5	0.38337517	6.21E-10	1.13E-05	0.61	0.401
SEC61G	5	0.38331414	8.82E-11	1.61E-06	1	0.986
DNAJB1	5	0.38244286	2.25E-08	0.00041078	0.341	0.141
POMP	5	0.37868104	1.01E-15	1.85E-11	0.932	0.718
S100A10	5	0.37861826	1.39E-09	2.53E-05	1	0.982
EIF4A2	5	0.37822878	7.30E-23	1.33E-18	1	1
CRTAC1	5	0.37656527	4.88E-20	8.91E-16	0.307	0.018
C4orf48	5	0.37532116	4.81E-14	8.77E-10	0.434	0.141
CLDND1	5	0.37006998	4.42E-11	8.06E-07	0.541	0.289
EIF1B	5	0.36890202	3.42E-13	6.25E-09	0.839	0.665
GAPDH	5	0.36831558	8.19E-16	1.49E-11	1	1
UBE2B	5	0.36647993	3.17E-10	5.79E-06	0.834	0.658
COPS2	5	0.36245832	9.06E-10	1.65E-05	0.751	0.56

RHOC	5	0.36205065	3.26E-07	0.00594986	0.834	0.725
SFT2D1	5	0.36124129	1.75E-11	3.20E-07	0.8	0.563
METAP2	5	0.36068473	5.06E-12	9.24E-08	0.854	0.623
SH3BP5	5	0.36049624	1.42E-10	2.59E-06	0.507	0.254
AEBP1	5	0.3583075	4.36E-09	7.96E-05	0.727	0.493
NDUFB8	5	0.35820251	1.96E-08	0.00035843	0.844	0.715
SNRNP27	5	0.35762917	4.57E-10	8.33E-06	0.507	0.278
RNMT	5	0.35552306	2.82E-12	5.14E-08	0.605	0.317
ZFYVE16	5	0.35449234	2.30E-13	4.20E-09	0.346	0.092
CNIH1	5	0.35267441	1.57E-11	2.86E-07	0.834	0.644
ATP6V1C1	5	0.35263251	4.61E-14	8.42E-10	0.454	0.158
PPP1R14C	5	0.34589847	1.54E-16	2.81E-12	0.317	0.042
USMG5	5	0.34438456	3.49E-14	6.37E-10	0.971	0.891
MRPL54	5	0.3431426	7.57E-12	1.38E-07	0.605	0.327
UFM1	5	0.3422579	3.01E-09	5.50E-05	0.659	0.447
TVP23B	5	0.34187971	3.10E-10	5.66E-06	0.532	0.282
GULP1	5	0.33846442	1.35E-14	2.47E-10	0.385	0.099
PTGES3	5	0.33820431	1.18E-16	2.15E-12	0.99	0.954
RNF126	5	0.338049	5.81E-13	1.06E-08	0.42	0.148
SPAG9	5	0.3375923	3.42E-12	6.23E-08	0.444	0.173
RAB13	5	0.33700055	8.30E-11	1.51E-06	0.902	0.778
IL11	5	0.33647224	4.71E-14	8.60E-10	0.185	0
TMOD1	5	0.3361511	7.52E-16	1.37E-11	0.278	0.028
LGALS3	5	0.3360416	2.29E-08	0.00041838	0.966	0.93
RPF2	5	0.33174462	1.85E-09	3.38E-05	0.415	0.183
MTHFD2	5	0.33067754	8.95E-08	0.0016334	0.732	0.588
CXCL8	5	0.32941849	4.52E-08	0.00082499	0.146	0.018
AKIRIN2	5	0.32910398	7.81E-12	1.43E-07	0.361	0.116
GRPEL1	5	0.32888397	6.10E-12	1.11E-07	0.449	0.176
PGK1	5	0.32791548	6.05E-09	0.00011032	0.941	0.831
NT5C3A	5	0.32596426	2.66E-10	4.85E-06	0.376	0.144
PAIP2	5	0.32255129	8.22E-10	1.50E-05	0.8	0.613
CAPZA2	5	0.32240554	2.76E-10	5.04E-06	0.8	0.563
GADD45GIP1	5	0.32116041	4.93E-09	8.99E-05	0.824	0.63
CTTN	5	0.32084916	3.45E-08	0.00062907	0.507	0.289
RGS16	5	0.32021712	1.75E-06	0.0320055	0.302	0.13
POPDC3	5	0.31955398	2.93E-12	5.34E-08	0.341	0.095
ATP6V0E1	5	0.31862028	1.38E-10	2.52E-06	0.912	0.813
BTG3	5	0.31780095	2.77E-08	0.00050528	0.459	0.257

YAF2	5	0.3173062	4.78E-10	8.72E-06	0.42	0.18
SQRDL	5	0.31639481	4.99E-10	9.11E-06	0.317	0.102
CHMP4A	5	0.31526897	4.22E-09	7.70E-05	0.668	0.479
CA9	5	0.31387952	6.33E-09	0.00011541	0.346	0.137
NDUFB4	5	0.31240891	1.51E-09	2.75E-05	0.902	0.792
SAP18	5	0.31161307	3.85E-10	7.03E-06	0.893	0.778
MRPL14	5	0.30985344	1.50E-09	2.73E-05	0.624	0.394
ACTR3	5	0.30958962	3.12E-08	0.00056852	0.576	0.37
HDAC2	5	0.3085292	1.11E-08	0.00020265	0.541	0.327
YWHAE	5	0.30816624	2.99E-09	5.46E-05	0.951	0.856
SYF2	5	0.3070735	1.77E-08	0.00032283	0.912	0.799
HNRNPDL	5	0.30688811	9.98E-09	0.00018213	0.839	0.669
ITGA5	5	0.30576155	1.91E-14	3.48E-10	0.307	0.053
DYNLT3	5	0.30526738	9.64E-07	0.01758878	0.698	0.542
C2orf49	5	0.30445805	8.05E-11	1.47E-06	0.39	0.144
NAMPT	5	0.30286854	1.10E-09	2.01E-05	0.615	0.37
KDM7A	5	0.30219404	2.25E-12	4.10E-08	0.268	0.049
PLCG2	5	0.30211304	4.80E-10	8.77E-06	0.224	0.042
AC090498.1	5	0.30177466	1.70E-20	3.10E-16	1	1
ZFAND2A	5	0.30048517	1.42E-14	2.59E-10	0.39	0.099
UBE2I	5	0.29920084	3.11E-08	0.00056759	0.741	0.577
MRPL41	5	0.2981073	1.23E-07	0.00225263	0.741	0.577
LSM12	5	0.29663664	3.05E-09	5.56E-05	0.429	0.201
IMP4	5	0.29643191	1.50E-09	2.74E-05	0.498	0.254
S100A6	5	0.29613993	1.64E-08	0.00029868	1	1
FERMT2	5	0.29586623	5.30E-09	9.67E-05	0.488	0.254
ADRM1	5	0.2957006	1.39E-09	2.54E-05	0.493	0.254
TMEM167A	5	0.29540161	1.93E-07	0.0035167	0.766	0.574
TFPI	5	0.29529746	2.97E-12	5.42E-08	0.263	0.046
NDUFA6	5	0.29505672	2.89E-07	0.00527119	0.62	0.423
LARP6	5	0.29376832	2.20E-06	0.04018465	0.644	0.486
KITLG	5	0.29338809	2.90E-12	5.30E-08	0.327	0.081
AIMP1	5	0.29335267	2.57E-06	0.04695	0.688	0.567
CDV3	5	0.29206271	6.49E-10	1.18E-05	0.546	0.299
MED10	5	0.29206271	1.50E-09	2.74E-05	0.546	0.306
NDUFS5	5	0.29091394	2.25E-10	4.10E-06	0.951	0.824
GSTM3	5	0.29045757	1.12E-09	2.04E-05	0.4	0.162
RIPK2	5	0.29041932	5.09E-11	9.29E-07	0.332	0.099
SNU13	5	0.29024618	2.06E-08	0.00037655	0.898	0.778

MRPL57	5	0.28964742	1.58E-07	0.0028861	0.849	0.725
MRPL52	5	0.28740527	6.90E-07	0.01258209	0.566	0.391
FAM207A	5	0.28685313	4.22E-09	7.70E-05	0.473	0.239
CGGBP1	5	0.28674355	1.18E-08	0.00021492	0.585	0.345
CCDC174	5	0.286299	1.43E-08	0.00026051	0.444	0.222
SLC25A36	5	0.28321309	1.83E-08	0.00033303	0.605	0.356
PRKRA	5	0.28242345	8.39E-08	0.00153014	0.527	0.32
TRAM1	5	0.28115024	1.21E-06	0.02208804	0.82	0.725
ACVR1	5	0.2805626	3.30E-07	0.00601693	0.483	0.285
MRPL51	5	0.27944424	9.25E-08	0.00168744	0.873	0.722
GYPC	5	0.27828684	1.41E-07	0.0025686	0.639	0.398
GNAI3	5	0.27730601	3.66E-09	6.68E-05	0.512	0.268
LRRFIP1	5	0.27650363	1.81E-06	0.03300477	0.42	0.236
NOP10	5	0.27625394	4.64E-07	0.00846582	0.785	0.62
ACTR10	5	0.27575556	1.22E-06	0.02221718	0.488	0.306
EVI2A	5	0.27467096	2.15E-12	3.93E-08	0.273	0.049
SREK1IP1	5	0.27265571	3.46E-08	0.00063146	0.454	0.236
OSER1	5	0.27265571	1.35E-06	0.02458271	0.439	0.254
AKAP12	5	0.27134317	7.00E-08	0.00127681	0.22	0.06
ABI1	5	0.27127437	7.03E-09	0.00012827	0.454	0.225
MRPL36	5	0.2706738	3.62E-08	0.00066029	0.571	0.352
CAMK2D	5	0.26952295	8.18E-08	0.0014921	0.341	0.148
SEMA3A	5	0.26921144	7.84E-15	1.43E-10	0.249	0.021
UBQLN1	5	0.26898682	9.10E-10	1.66E-05	0.356	0.13
RAB14	5	0.26849291	1.37E-07	0.00249259	0.478	0.271
PTBP3	5	0.26629193	5.12E-11	9.33E-07	0.39	0.137
LINC00493	5	0.26608076	3.19E-08	0.00058216	0.937	0.803
TNS1	5	0.26533964	1.67E-13	3.04E-09	0.322	0.067
SLC7A2	5	0.2618854	2.48E-11	4.53E-07	0.2	0.021
KLHL21	5	0.25927288	1.19E-11	2.17E-07	0.307	0.074
RHOF	5	0.25927288	2.15E-08	0.0003923	0.263	0.081
PPDPF	5	0.25728524	1.09E-11	1.99E-07	1	1
PHF1	5	0.25662957	2.55E-06	0.04653709	0.556	0.37
EMC2	5	0.25609458	1.70E-06	0.03105296	0.624	0.433
CPEB4	5	0.25505629	1.14E-07	0.0020835	0.356	0.162
NDUFC1	5	0.25501252	3.08E-07	0.00562255	0.917	0.824
CGRRF1	5	0.25439407	3.53E-08	0.00064363	0.341	0.137
RND3	5	0.25424895	6.76E-13	1.23E-08	0.215	0.018
BNIP3	5	0.25422689	7.13E-08	0.00130077	1	1

SNRPG	5	0.25011564	5.48E-07	0.01000713	0.776	0.57
EIF2A	5	-0.2509231	2.24E-06	0.04093885	0.673	0.792
TPGS2	5	-0.2518968	2.94E-09	5.37E-05	0.083	0.303
MPP6	5	-0.2521136	6.03E-09	0.00010997	0.107	0.331
PPM1K	5	-0.2522239	5.59E-09	0.00010195	0.068	0.275
MATN4	5	-0.2530696	5.99E-09	0.00010935	0.107	0.338
HNRNPM	5	-0.2541768	3.16E-08	0.00057652	0.185	0.415
SERTAD4-AS1	5	-0.2549857	1.37E-13	2.50E-09	0.024	0.285
PIGT	5	-0.2550656	4.67E-10	8.53E-06	0.102	0.349
CHST12	5	-0.2551437	4.86E-12	8.87E-08	0.049	0.299
SLC38A1	5	-0.2559573	5.59E-09	0.00010192	0.093	0.313
TMED4	5	-0.2568634	1.98E-06	0.03621199	0.307	0.507
TCTN1	5	-0.2568719	8.55E-11	1.56E-06	0.088	0.338
C9orf3	5	-0.2580559	3.58E-08	0.00065269	0.21	0.447
SNHG25	5	-0.2582888	5.19E-07	0.00946902	0.195	0.398
DUT	5	-0.258459	6.68E-07	0.01218888	0.459	0.655
KHDRBS3	5	-0.2592351	2.29E-10	4.18E-06	0.034	0.246
PHB2	5	-0.2593344	8.38E-07	0.01529913	0.38	0.581
SLC25A6	5	-0.2606552	6.18E-11	1.13E-06	0.99	0.996
SNRPA	5	-0.2622833	1.43E-09	2.61E-05	0.141	0.391
CYB5R1	5	-0.263252	4.92E-09	8.99E-05	0.127	0.356
OTOS	5	-0.2642885	1.07E-08	0.00019529	0.083	0.292
CD320	5	-0.2644378	5.73E-07	0.01045573	0.288	0.507
RARRES3	5	-0.2644644	1.61E-10	2.93E-06	0.024	0.229
C12orf57	5	-0.264922	9.01E-09	0.00016437	0.966	0.993
PNKD	5	-0.2667785	1.38E-07	0.0025196	0.161	0.37
RPL27A	5	-0.2668779	4.77E-27	8.71E-23	1	1
CDKN2C	5	-0.267948	1.91E-11	3.48E-07	0.054	0.296
SMIM19	5	-0.2684835	3.37E-07	0.0061515	0.254	0.458
SOCS3	5	-0.2685923	3.67E-09	6.70E-05	0.083	0.306
CANX	5	-0.2692025	1.33E-06	0.02429547	0.615	0.782
ARL6IP4	5	-0.2694621	9.15E-10	1.67E-05	0.844	0.937
PRSS23	5	-0.2698546	2.39E-06	0.04368162	0.161	0.349
SNHG8	5	-0.2703835	1.00E-09	1.83E-05	0.883	0.937
PCOLCE2	5	-0.2703996	3.70E-07	0.00675392	0.932	0.975
ARL2BP	5	-0.2710686	5.68E-07	0.0103665	0.371	0.57
ARPC1A	5	-0.2718727	2.99E-07	0.00544975	0.322	0.532
LRRC2	5	-0.2725333	3.31E-09	6.05E-05	0.088	0.31
FAM20C	5	-0.2743696	1.42E-10	2.59E-06	0.063	0.296

TMED2	5	-0.2747496	1.36E-08	0.00024778	0.712	0.866
RPL35A	5	-0.2754589	9.60E-28	1.75E-23	1	1
NDRG2	5	-0.2776431	3.44E-12	6.28E-08	0.054	0.306
ZFAND1	5	-0.2786552	4.10E-07	0.00748358	0.468	0.655
TMED9	5	-0.2787042	1.09E-07	0.00198142	0.463	0.683
EIF3E	5	-0.2787897	4.46E-12	8.14E-08	1	1
OXA1L	5	-0.2793667	1.14E-07	0.00207612	0.459	0.644
TPM2	5	-0.2796171	7.60E-07	0.01386989	0.395	0.592
IDH2	5	-0.2797365	5.50E-14	1.00E-09	0.02	0.278
CSGALNACT1	5	-0.2805487	1.86E-10	3.40E-06	0.132	0.398
DAAM1	5	-0.2808548	2.40E-06	0.04384379	0.215	0.412
RPL21	5	-0.2810222	2.03E-32	3.70E-28	1	1
ATP5D	5	-0.281367	3.30E-09	6.02E-05	0.824	0.919
SUMF2	5	-0.2814161	1.86E-08	0.00033899	0.298	0.542
LUC7L3	5	-0.2818984	7.56E-10	1.38E-05	0.2	0.472
NKX3-2	5	-0.2819183	3.21E-12	5.86E-08	0.015	0.239
EIF3D	5	-0.2820891	2.37E-08	0.00043217	0.663	0.838
PRKCDBP	5	-0.2826762	1.53E-08	0.00027974	0.302	0.542
GADD45B	5	-0.2830307	6.13E-07	0.01119028	0.151	0.352
NME4	5	-0.2841477	6.93E-10	1.26E-05	0.176	0.437
LRP10	5	-0.286167	1.33E-08	0.00024221	0.2	0.437
ANG	5	-0.2866684	1.53E-09	2.79E-05	0.22	0.482
RPL35	5	-0.286888	3.50E-23	6.39E-19	1	1
NINJ1	5	-0.2870235	2.03E-07	0.00369749	0.449	0.63
ABHD14B	5	-0.2871402	7.20E-09	0.00013136	0.215	0.458
BGN	5	-0.2881224	8.89E-09	0.00016221	1	1
COX7A2L	5	-0.288562	5.14E-10	9.38E-06	0.82	0.919
HIST1H2AC	5	-0.2891054	4.41E-10	8.05E-06	0.112	0.359
NHP2	5	-0.2895099	3.05E-10	5.57E-06	0.278	0.563
PRKCSH	5	-0.2899244	3.37E-08	0.00061463	0.259	0.489
HOXB8	5	-0.2901262	1.99E-14	3.63E-10	0.02	0.285
SLC1A5	5	-0.2920623	2.57E-12	4.69E-08	0.083	0.359
UMAD1	5	-0.2942762	3.95E-08	0.00072151	0.215	0.433
CMPK1	5	-0.2946796	5.90E-10	1.08E-05	0.639	0.863
NFIC	5	-0.295724	6.13E-10	1.12E-05	0.215	0.479
CNPY3	5	-0.2957969	1.58E-09	2.88E-05	0.205	0.458
RBMX	5	-0.2968509	2.29E-06	0.04172072	0.551	0.722
TMEM59	5	-0.2972551	2.76E-12	5.04E-08	0.971	0.996
YBX1	5	-0.2974044	1.90E-13	3.47E-09	0.966	0.993

MORF4L2	5	-0.2975553	1.26E-08	0.00022953	0.532	0.729
RPL18A	5	-0.2977042	1.29E-30	2.35E-26	1	1
NPM1	5	-0.297708	4.00E-15	7.30E-11	0.995	0.996
CREG1	5	-0.3005409	2.14E-08	0.00039037	0.478	0.669
IER2	5	-0.3005589	1.24E-07	0.00225674	0.166	0.384
TRPS1	5	-0.3006222	7.61E-11	1.39E-06	0.205	0.493
ETFB	5	-0.3010719	3.90E-08	0.00071099	0.337	0.556
OGFOD3	5	-0.302921	1.54E-11	2.80E-07	0.156	0.437
TXNIP	5	-0.3029633	1.92E-14	3.50E-10	0.02	0.285
ERGIC3	5	-0.3040898	3.04E-09	5.55E-05	0.712	0.877
GPAA1	5	-0.3051309	5.11E-10	9.33E-06	0.161	0.412
TP53I13	5	-0.3066942	3.14E-12	5.72E-08	0.117	0.398
DSEL	5	-0.306705	1.57E-10	2.86E-06	0.039	0.254
CD302	5	-0.3068398	1.37E-13	2.50E-09	0.063	0.352
VKORC1	5	-0.3075447	1.43E-10	2.61E-06	0.912	0.979
BTF3	5	-0.3078741	3.52E-17	6.43E-13	1	1
RPS4Y1	5	-0.3088473	1.07E-14	1.95E-10	0.995	0.996
CCDC8	5	-0.3094746	2.68E-13	4.90E-09	0.039	0.303
RPL37	5	-0.3095065	4.99E-28	9.11E-24	1	1
NAA38	5	-0.3100245	3.42E-09	6.24E-05	0.459	0.697
PTPRD-AS1	5	-0.3111683	1.60E-09	2.92E-05	0.205	0.458
RPS12	5	-0.3119049	3.15E-24	5.74E-20	1	1
GSTA4	5	-0.3139222	1.16E-10	2.11E-06	0.151	0.415
TMED10	5	-0.3141012	3.21E-10	5.86E-06	0.854	0.915
LDHB	5	-0.3145653	5.20E-10	9.49E-06	0.717	0.866
RPS19	5	-0.3147406	9.23E-27	1.68E-22	1	1
PRCP	5	-0.3154067	8.26E-14	1.51E-09	0.059	0.342
HMGN3	5	-0.3160993	9.54E-10	1.74E-05	0.171	0.426
S100A4	5	-0.3165293	5.02E-09	9.15E-05	0.585	0.806
RPS9	5	-0.3186044	1.82E-34	3.33E-30	1	1
TNFRSF1A	5	-0.3196002	1.94E-09	3.54E-05	0.22	0.475
MIR99AHG	5	-0.3204454	2.87E-12	5.23E-08	0.068	0.327
01-Sep	5	-0.3227312	2.95E-11	5.39E-07	0.098	0.359
TMEM219	5	-0.322791	2.94E-09	5.36E-05	0.483	0.697
CYBRD1	5	-0.3228625	4.81E-08	0.00087712	0.449	0.644
EIF3K	5	-0.3247769	1.25E-11	2.29E-07	0.79	0.898
TMEM230	5	-0.3249406	2.60E-10	4.74E-06	0.566	0.778
NMRK1	5	-0.3253358	8.48E-11	1.55E-06	0.161	0.43
UQCRH	5	-0.3259262	9.26E-15	1.69E-10	0.932	0.986

TSPAN4	5	-0.3264333	2.39E-09	4.35E-05	0.288	0.542
RPS25	5	-0.3269414	3.12E-36	5.70E-32	1	1
FKBP10	5	-0.3271047	5.54E-09	0.00010117	0.293	0.525
MYLK	5	-0.3272663	5.93E-11	1.08E-06	0.088	0.342
RPL37A	5	-0.3281878	4.18E-36	7.63E-32	1	1
RPL7A	5	-0.3283209	3.51E-27	6.41E-23	1	1
H1FX	5	-0.3290189	1.25E-11	2.28E-07	0.088	0.352
ENAH	5	-0.3306492	8.68E-15	1.58E-10	0.088	0.398
RPL14	5	-0.3312816	2.11E-32	3.85E-28	1	1
DMKN	5	-0.3313938	1.41E-09	2.58E-05	0.195	0.444
RP5-1097P24.1	5	-0.3343931	1.42E-15	2.59E-11	0.039	0.345
RABAC1	5	-0.3354917	2.88E-12	5.25E-08	0.746	0.894
EIF2S3	5	-0.3357966	3.98E-08	0.00072593	0.434	0.641
MTDH	5	-0.3360362	5.62E-10	1.03E-05	0.502	0.718
POLR2L	5	-0.3368201	2.00E-08	0.00036464	0.722	0.863
KDELR2	5	-0.3372278	1.71E-11	3.12E-07	0.771	0.933
FAM200B	5	-0.3375825	6.55E-12	1.20E-07	0.151	0.433
TMEM98	5	-0.3376017	1.12E-12	2.05E-08	0.083	0.356
RPL29	5	-0.3381607	4.55E-29	8.30E-25	1	1
RPS4X	5	-0.341086	5.31E-35	9.69E-31	1	1
AC005152.3	5	-0.3411459	4.08E-12	7.45E-08	0.039	0.282
TSPAN3	5	-0.3412074	7.34E-09	0.000134	0.366	0.581
FAM46A	5	-0.3417937	1.63E-08	0.00029823	0.376	0.634
PNRC1	5	-0.3435024	7.82E-14	1.43E-09	0.927	0.996
HSPB1	5	-0.3437027	6.13E-11	1.12E-06	0.576	0.82
RP11-983P16.4	5	-0.3453783	1.71E-11	3.11E-07	0.195	0.482
RPS29	5	-0.3460422	2.06E-32	3.75E-28	1	1
RPS3	5	-0.3471793	2.26E-36	4.13E-32	1	1
RPL5	5	-0.3508602	2.79E-32	5.10E-28	1	1
ABHD14A	5	-0.3512916	2.01E-15	3.66E-11	0.068	0.384
RPL11	5	-0.3521252	2.05E-41	3.74E-37	1	1
TCEAL8	5	-0.3528629	2.49E-11	4.55E-07	0.62	0.803
PNISR	5	-0.3554564	1.48E-10	2.70E-06	0.502	0.722
RNASET2	5	-0.3568416	2.63E-12	4.80E-08	0.156	0.444
PGAM1	5	-0.3573306	1.99E-12	3.64E-08	0.624	0.866
PLD3	5	-0.3578225	3.80E-11	6.93E-07	0.395	0.655
ST13	5	-0.3581603	2.12E-16	3.86E-12	0.966	0.975
C7orf73	5	-0.3607795	2.95E-11	5.38E-07	0.6	0.835
SH3BGRL	5	-0.3620503	2.43E-11	4.44E-07	0.322	0.606

C19orf43	5	-0.3621904	1.91E-13	3.49E-09	0.702	0.912
ASPN	5	-0.362477	5.47E-07	0.00997816	0.024	0.165
ATRAID	5	-0.3627404	1.18E-12	2.14E-08	0.737	0.915
COMMD6	5	-0.3636748	6.48E-20	1.18E-15	0.995	1
RPL6	5	-0.363832	4.10E-31	7.49E-27	1	1
ITM2B	5	-0.3664074	1.56E-14	2.86E-10	0.971	1
HERPUD1	5	-0.3671829	8.24E-11	1.50E-06	0.278	0.546
AC003090.1	5	-0.3695058	2.74E-17	4.99E-13	0.034	0.359
CMB9-22P13.1	5	-0.3708527	1.11E-11	2.02E-07	0.263	0.542
SLC44A1	5	-0.3715213	2.46E-14	4.50E-10	0.107	0.419
RPLP1	5	-0.3719098	4.92E-42	8.98E-38	1	1
PLPP1	5	-0.373917	1.08E-10	1.96E-06	0.083	0.32
RPS18	5	-0.3747336	3.63E-38	6.62E-34	1	1
NUCB2	5	-0.3788206	1.39E-09	2.54E-05	0.307	0.553
GNAS	5	-0.3798147	5.05E-12	9.21E-08	0.498	0.715
CRIP2	5	-0.3817817	8.71E-11	1.59E-06	0.254	0.518
RPS7	5	-0.3821086	2.37E-31	4.33E-27	1	1
EFNA1	5	-0.3829313	3.42E-12	6.24E-08	0.171	0.468
COL5A2	5	-0.3829313	1.55E-11	2.83E-07	0.151	0.43
ARHGAP29	5	-0.3831195	1.63E-11	2.98E-07	0.151	0.433
RPL22	5	-0.3834098	9.55E-33	1.74E-28	1	1
BHLHE41	5	-0.3842774	2.32E-12	4.23E-08	0.102	0.38
BRK1	5	-0.3847787	4.33E-16	7.89E-12	0.722	0.905
ANGPTL5	5	-0.3875226	1.53E-18	2.79E-14	0.059	0.415
GLTSCR2	5	-0.3885665	1.73E-16	3.15E-12	0.834	0.961
ATRNL1	5	-0.3893568	1.06E-16	1.93E-12	0.078	0.412
HDLBP	5	-0.3896558	2.69E-11	4.91E-07	0.283	0.553
ZBTB20	5	-0.3907134	3.78E-16	6.89E-12	0.102	0.444
VCAN	5	-0.3912805	8.60E-09	0.00015695	0.268	0.496
CTSK	5	-0.3913334	1.38E-17	2.52E-13	0.073	0.426
CCNG1	5	-0.3930687	9.99E-13	1.82E-08	0.307	0.599
RPL15	5	-0.3931861	2.31E-50	4.21E-46	1	1
RPS2	5	-0.393987	7.84E-43	1.43E-38	1	1
TCEA3	5	-0.3941276	4.81E-13	8.77E-09	0.146	0.437
RPS5	5	-0.3964929	1.42E-36	2.58E-32	1	1
CPE	5	-0.3996182	3.23E-11	5.90E-07	0.605	0.789
RPL13	5	-0.4001116	6.56E-52	1.20E-47	1	1
CALR	5	-0.4003478	2.97E-09	5.43E-05	0.546	0.732
NDUFA11	5	-0.4004639	5.51E-15	1.00E-10	0.8	0.951

TPD52L1	5	-0.4049232	5.61E-10	1.02E-05	0.283	0.539
ILF3-AS1	5	-0.4100029	1.21E-16	2.21E-12	0.141	0.496
HLA-C	5	-0.4119923	1.31E-12	2.39E-08	0.888	0.965
RCN2	5	-0.4133958	1.91E-13	3.48E-09	0.459	0.715
RPL3	5	-0.4137119	1.16E-38	2.11E-34	1	1
SRSF5	5	-0.4161095	1.76E-11	3.22E-07	0.449	0.683
PCBP2	5	-0.4182381	1.45E-18	2.64E-14	0.824	0.958
ENPP1	5	-0.4193687	4.76E-17	8.69E-13	0.078	0.419
RPSA	5	-0.4195375	2.27E-30	4.15E-26	1	1
DBP	5	-0.4198822	1.45E-15	2.65E-11	0.107	0.433
RPL13A	5	-0.420591	1.08E-53	1.97E-49	1	1
MYL6	5	-0.4216428	3.53E-18	6.44E-14	0.863	0.972
SSR2	5	-0.4220722	7.02E-16	1.28E-11	0.815	0.93
PTRF	5	-0.4222427	3.65E-14	6.66E-10	0.239	0.553
COL9A2	5	-0.4224456	3.76E-12	6.86E-08	0.195	0.489
SPON2	5	-0.4231115	1.52E-13	2.78E-09	0.078	0.363
PIK3R1	5	-0.4251056	6.74E-12	1.23E-07	0.059	0.306
ARL6IP5	5	-0.4252782	8.37E-14	1.53E-09	0.385	0.683
MFGE8	5	-0.4278859	1.17E-10	2.13E-06	0.824	0.947
PIM3	5	-0.4287651	3.79E-11	6.91E-07	0.322	0.588
TUBB	5	-0.4292913	6.28E-14	1.15E-09	0.698	0.898
RPL10	5	-0.4314032	4.67E-55	8.52E-51	1	1
TOMM7	5	-0.4315069	1.28E-41	2.33E-37	1	1
RPS27A	5	-0.43199	4.03E-51	7.35E-47	1	1
GRN	5	-0.4335974	1.76E-17	3.20E-13	0.098	0.458
SPINT2	5	-0.434976	2.31E-11	4.21E-07	0.268	0.539
RPL8	5	-0.4353674	4.19E-48	7.64E-44	1	1
FOS	5	-0.4385399	5.69E-12	1.04E-07	0.317	0.623
PPIA	5	-0.4387494	3.41E-19	6.21E-15	0.888	0.982
BAMBI	5	-0.4406854	6.09E-10	1.11E-05	0.312	0.574
RPS6	5	-0.4407955	8.30E-53	1.51E-48	1	1
PRELP	5	-0.4417649	3.12E-13	5.69E-09	0.917	0.979
CXXC5	5	-0.443059	2.66E-16	4.86E-12	0.068	0.391
EEF1B2	5	-0.4438472	1.00E-22	1.83E-18	0.985	1
SEC11A	5	-0.4447412	8.99E-20	1.64E-15	0.766	0.954
PDE4DIP	5	-0.4455148	1.01E-13	1.85E-09	0.259	0.56
CTGF	5	-0.4472256	7.83E-12	1.43E-07	0.127	0.419
RPS23	5	-0.4477147	1.21E-45	2.22E-41	1	1
FCGRT	5	-0.4496895	1.05E-12	1.92E-08	0.454	0.669

MAGED1	5	-0.4534041	3.76E-15	6.87E-11	0.024	0.306
RNF130	5	-0.4552902	6.97E-15	1.27E-10	0.356	0.655
RPS13	5	-0.4570191	6.26E-46	1.14E-41	1	1
RPN2	5	-0.4571794	1.39E-19	2.53E-15	0.063	0.44
RPL19	5	-0.4575742	4.78E-55	8.73E-51	1	1
CUTA	5	-0.4584743	4.58E-16	8.35E-12	0.702	0.859
RPL23	5	-0.4598066	1.12E-32	2.04E-28	0.995	1
MIA	5	-0.4619872	3.62E-15	6.60E-11	0.961	0.996
RPL34	5	-0.4628553	3.46E-50	6.31E-46	1	1
PPP1R3C	5	-0.4638098	1.92E-12	3.50E-08	0.263	0.539
GSN	5	-0.4644613	5.92E-14	1.08E-09	0.376	0.676
SEPP1	5	-0.4645571	1.25E-17	2.28E-13	0.205	0.592
PTMS	5	-0.4652031	4.27E-18	7.79E-14	0.395	0.764
PPIC	5	-0.4652651	2.97E-13	5.41E-09	0.234	0.518
RPL9	5	-0.4670324	5.11E-50	9.33E-46	1	1
HTRA1	5	-0.4671719	9.99E-18	1.82E-13	0.551	0.891
PSAP	5	-0.4681088	7.90E-20	1.44E-15	0.844	0.972
DNM3OS	5	-0.4683681	1.16E-19	2.11E-15	0.044	0.405
MKX	5	-0.4691041	1.42E-20	2.59E-16	0.093	0.496
ATPIF1	5	-0.4707462	5.33E-18	9.73E-14	0.444	0.764
PRUNE2	5	-0.4719408	7.40E-16	1.35E-11	0.161	0.511
HEXB	5	-0.4728812	5.58E-15	1.02E-10	0.307	0.627
PPT1	5	-0.4729566	7.24E-19	1.32E-14	0.146	0.525
AP2M1	5	-0.4735633	5.45E-19	9.95E-15	0.317	0.687
DCN	5	-0.4747403	1.32E-25	2.41E-21	1	0.996
LRPAP1	5	-0.4754356	1.20E-14	2.18E-10	0.41	0.711
RPLP2	5	-0.4759606	1.32E-50	2.40E-46	1	1
RPS17	5	-0.4762367	4.34E-43	7.91E-39	1	1
HMGN2	5	-0.4815716	2.98E-19	5.43E-15	0.795	0.965
YBX3	5	-0.4828381	3.96E-19	7.23E-15	0.717	0.933
CTSD	5	-0.4842036	2.69E-14	4.92E-10	0.776	0.944
RPL30	5	-0.4846686	3.45E-45	6.30E-41	1	1
DUSP1	5	-0.4867978	3.14E-13	5.73E-09	0.283	0.585
PKM	5	-0.4899077	3.61E-18	6.59E-14	0.815	0.951
SERPINA5	5	-0.4901724	1.69E-12	3.08E-08	0.332	0.602
CYR61	5	-0.490243	4.12E-10	7.51E-06	0.112	0.356
FTL	5	-0.4912289	8.71E-38	1.59E-33	1	1
HLA-A	5	-0.4968351	2.08E-17	3.80E-13	0.839	0.982
NFIA	5	-0.5001586	5.87E-22	1.07E-17	0.098	0.5

DDR2	5	-0.5029845	3.44E-16	6.27E-12	0.244	0.592
EID1	5	-0.5056838	3.49E-26	6.37E-22	0.834	0.993
RPL23A	5	-0.5089438	2.27E-54	4.14E-50	1	1
NAP1L1	5	-0.5152248	1.19E-28	2.16E-24	0.927	0.986
RPS16	5	-0.5161266	1.12E-54	2.04E-50	1	1
TTC3	5	-0.5176639	2.93E-16	5.34E-12	0.42	0.725
EIF4B	5	-0.5253384	1.67E-20	3.05E-16	0.702	0.93
RPS3A	5	-0.5263663	1.42E-58	2.59E-54	1	1
TSPO	5	-0.5297529	2.01E-22	3.67E-18	0.663	0.908
TCF4	5	-0.5313525	1.73E-21	3.15E-17	0.098	0.496
CST3	5	-0.5319909	1.41E-22	2.58E-18	0.985	1
SDC2	5	-0.5325419	3.32E-20	6.05E-16	0.683	0.933
PRDX4	5	-0.539263	5.62E-21	1.03E-16	0.595	0.894
CHID1	5	-0.5419246	2.00E-23	3.64E-19	0.346	0.764
RPL7	5	-0.54304	9.68E-47	1.77E-42	1	1
MDFI	5	-0.5441136	9.39E-19	1.71E-14	0.239	0.616
SSPN	5	-0.5472083	3.25E-25	5.94E-21	0.044	0.486
CCDC102B	5	-0.5474699	9.79E-27	1.79E-22	0	0.43
SOD3	5	-0.5475278	5.96E-13	1.09E-08	0.912	0.965
RPL18	5	-0.5477756	2.18E-55	3.98E-51	1	1
FMOD	5	-0.5515267	6.65E-25	1.21E-20	0.873	0.965
PLXDC2	5	-0.5559938	4.16E-22	7.59E-18	0.346	0.739
APOD	5	-0.5560694	4.94E-11	9.02E-07	0.244	0.511
JUNB	5	-0.5592359	2.19E-16	4.00E-12	0.405	0.746
SLC14A1	5	-0.5687846	7.64E-21	1.39E-16	0.166	0.563
ACTG1	5	-0.5755842	2.20E-24	4.01E-20	0.937	0.982
MT-CO3	5	-0.5783249	2.34E-06	0.0427071	0.8	0.778
BSG	5	-0.5801958	2.23E-26	4.07E-22	0.502	0.863
PDIA3	5	-0.5820383	1.72E-19	3.15E-15	0.376	0.725
XAGE2	5	-0.5874237	2.80E-10	5.10E-06	0.005	0.187
MALAT1	5	-0.589497	5.06E-23	9.24E-19	1	1
SLC40A1	5	-0.5903265	2.86E-24	5.21E-20	0.034	0.454
CCNI	5	-0.6047323	7.32E-37	1.34E-32	1	1
COL11A1	5	-0.6104001	1.59E-22	2.90E-18	0.556	0.866
SDF4	5	-0.6225502	2.06E-24	3.76E-20	0.454	0.799
HIST1H4C	5	-0.6247632	2.20E-21	4.02E-17	0.224	0.63
TPT1	5	-0.6278653	5.71E-57	1.04E-52	1	1
H3F3A	5	-0.6339503	6.47E-38	1.18E-33	0.893	1
RPLP0	5	-0.6359304	1.65E-58	3.01E-54	1	1

VIM	5	-0.6389763	1.64E-35	3.00E-31	1	1
ITM2A	5	-0.6439699	3.94E-22	7.18E-18	0.327	0.729
MT-ND1	5	-0.6495879	3.02E-18	5.51E-14	0.185	0.574
FXYD6	5	-0.6518338	1.86E-30	3.39E-26	0.576	0.912
MT-CYB	5	-0.6520834	6.35E-12	1.16E-07	0.483	0.683
GPX3	5	-0.6544743	4.57E-25	8.33E-21	0.844	0.986
ID4	5	-0.6563476	1.75E-22	3.19E-18	0.293	0.708
RBP4	5	-0.6633845	8.41E-22	1.54E-17	0.585	0.905
CTSC	5	-0.6637169	6.13E-14	1.12E-09	0.137	0.437
EIF3L	5	-0.6663124	1.56E-35	2.85E-31	0.761	0.972
MT-ND3	5	-0.6666068	1.09E-13	1.99E-09	0.376	0.637
PABPC1	5	-0.6667525	7.67E-34	1.40E-29	0.722	0.961
PEBP1	5	-0.6670344	3.24E-32	5.92E-28	0.6	0.923
RPL10A	5	-0.6673183	1.52E-58	2.77E-54	1	1
INHBA	5	-0.6681102	9.69E-25	1.77E-20	0.073	0.511
MT-ATP6	5	-0.6692349	3.04E-08	0.00055386	0.644	0.718
RPS8	5	-0.6712991	1.31E-65	2.40E-61	1	1
CRTAP	5	-0.6895814	5.29E-33	9.65E-29	0.517	0.88
SSR3	5	-0.6923373	1.60E-33	2.93E-29	0.81	0.982
NPC2	5	-0.6929965	2.06E-46	3.77E-42	0.966	1
RPS20	5	-0.6930997	1.32E-58	2.41E-54	1	1
RPS11	5	-0.6970069	6.23E-55	1.14E-50	0.985	1
NNMT	5	-0.7079383	6.69E-26	1.22E-21	0.805	0.972
GAS6	5	-0.7084861	8.50E-31	1.55E-26	0.127	0.637
FTH1	5	-0.7114272	6.94E-47	1.27E-42	1	1
ITM2C	5	-0.7168256	2.92E-26	5.33E-22	0.229	0.669
MGP	5	-0.7239868	1.34E-23	2.45E-19	0.995	1
CFH	5	-0.7305365	7.10E-15	1.29E-10	0.156	0.489
CEBPD	5	-0.7324729	4.09E-26	7.46E-22	0.668	0.93
CP	5	-0.7364002	7.96E-26	1.45E-21	0.556	0.852
NGFRAP1	5	-0.7369299	4.10E-40	7.47E-36	0.659	0.972
HNRNPA1	5	-0.7434003	5.38E-45	9.83E-41	0.976	1
ZFP36L2	5	-0.7510089	9.62E-22	1.75E-17	0.063	0.451
MT-CO2	5	-0.7533633	1.17E-08	0.00021417	0.859	0.838
CITED2	5	-0.7618759	2.41E-27	4.40E-23	0.21	0.694
RPL4	5	-0.7646504	6.18E-62	1.13E-57	0.995	1
CD9	5	-0.7649683	3.82E-28	6.97E-24	0.327	0.764
MT-ND2	5	-0.772648	8.08E-16	1.47E-11	0.307	0.627
ISLR	5	-0.7970921	1.12E-33	2.04E-29	0.083	0.623

SNAI2	5	-0.8045918	6.53E-28	1.19E-23	0.18	0.669
LECT1	5	-0.807707	3.08E-24	5.62E-20	0.722	0.951
PDIA6	5	-0.8090157	6.02E-38	1.10E-33	0.42	0.884
ID2	5	-0.8095915	1.57E-20	2.86E-16	0.293	0.669
SPARC	5	-0.8315831	3.72E-32	6.78E-28	0.288	0.785
EIF3H	5	-0.8320643	7.36E-56	1.34E-51	0.961	0.996
S100B	5	-0.8366854	6.67E-38	1.22E-33	0.663	0.951
C2orf82	5	-0.8420913	2.44E-36	4.46E-32	0.976	1
EEF2	5	-0.8777646	2.07E-47	3.77E-43	0.985	1
CD99	5	-0.8787647	5.13E-48	9.36E-44	0.81	0.989
ACAN	5	-0.8804383	1.03E-45	1.89E-41	0.917	0.993
FBXO2	5	-0.8804645	3.24E-44	5.91E-40	0.478	0.919
RHOBTB3	5	-0.9253643	4.39E-42	8.01E-38	0.278	0.856
NEAT1	5	-0.9324608	2.86E-47	5.22E-43	0.985	1
TSC22D1	5	-0.9440376	1.36E-27	2.49E-23	0.532	0.877
EEF1A1	5	-0.9496478	1.04E-73	1.90E-69	1	1
GREM1	5	-1.0223586	4.60E-28	8.39E-24	0.01	0.465
B2M	5	-1.0365504	2.06E-60	3.76E-56	0.976	1
COL2A1	5	-1.0391091	3.72E-45	6.79E-41	0.317	0.901
P4HB	5	-1.0465292	2.56E-49	4.68E-45	0.605	0.975
PAX1	5	-1.0492277	1.52E-49	2.77E-45	0.366	0.901
CHRDL2	5	-1.0593174	6.80E-31	1.24E-26	0.151	0.655
ADIRF	5	-1.0753092	3.46E-32	6.31E-28	0.337	0.817
CRISPLD1	5	-1.1009625	1.04E-42	1.89E-38	0.024	0.651
COL9A3	5	-1.1264335	4.43E-34	8.08E-30	0.488	0.884
CAPS	5	-1.1620519	2.32E-41	4.23E-37	0.61	0.958
SLPI	5	-1.1949452	3.85E-22	7.03E-18	0.976	1
PLAC9	5	-1.2044109	2.02E-52	3.69E-48	0.488	0.954
ID1	5	-1.3071634	4.47E-36	8.15E-32	0.22	0.757
ID3	5	-1.3939822	1.84E-47	3.35E-43	0.146	0.817
OGN	5	-1.4089503	6.10E-48	1.11E-43	0.722	0.975
CLEC3A	5	-1.9489993	1.70E-65	3.10E-61	0.215	0.947
FRZB	5	-1.9857645	7.34E-65	1.34E-60	0.629	0.993
SPTSSB	5	-2.0222614	7.11E-69	1.30E-64	0.024	0.884
**gene**	**cluster**	**avg_logFC**	**p_val**	**p_val_adj**	**pct.1**	**pct.2**
SPP1	6	2.54369566	5.05E-40	9.22E-36	0.827	0.162
MT2A	6	2.07931677	3.58E-63	6.54E-59	1	0.994
PRG4	6	1.76716136	7.62E-33	1.39E-28	0.632	0.06
CTSL	6	1.50849939	5.95E-38	1.09E-33	0.906	0.527

UPP1	6	1.43079229	7.34E-53	1.34E-48	0.858	0.132
IL11	6	1.4018953	3.49E-22	6.36E-18	0.421	0
IGFBP3	6	1.37822556	4.21E-30	7.68E-26	0.723	0.18
PTGS2	6	1.26029874	1.79E-40	3.26E-36	0.714	0.06
CXCL8	6	1.24623442	2.26E-07	0.00413108	0.17	0.012
MRPS6	6	1.24195197	1.20E-42	2.20E-38	0.987	0.91
S100A2	6	1.22139207	1.73E-21	3.15E-17	0.808	0.437
COMP	6	1.19568839	2.14E-22	3.91E-18	0.937	0.713
MT1X	6	1.1922278	4.89E-28	8.93E-24	1	0.976
SOD2	6	1.19129115	6.13E-33	1.12E-28	0.994	0.79
BMP2	6	1.17314856	2.20E-37	4.02E-33	0.717	0.09
STK17A	6	1.14842944	2.26E-42	4.13E-38	0.846	0.24
TGFBI	6	1.14544711	8.98E-23	1.64E-18	0.648	0.228
VEGFA	6	1.13484374	3.04E-41	5.54E-37	0.909	0.401
C11orf96	6	1.09415686	1.41E-35	2.57E-31	0.676	0.072
TM4SF1	6	1.07359393	4.56E-30	8.32E-26	0.915	0.593
SMOX	6	1.04098752	2.46E-28	4.49E-24	0.701	0.198
HMGA1	6	1.03386026	4.16E-33	7.59E-29	0.89	0.437
CD55	6	1.02492856	1.61E-36	2.94E-32	0.925	0.497
PHLDA1	6	0.96349177	1.11E-24	2.02E-20	0.579	0.096
TNFRSF12A	6	0.95851472	5.40E-38	9.85E-34	0.94	0.467
CTHRC1	6	0.93967199	6.51E-25	1.19E-20	0.604	0.126
PMP22	6	0.8800645	4.78E-33	8.73E-29	0.94	0.647
SERPINE1	6	0.86313025	9.73E-17	1.77E-12	0.56	0.186
BAALC	6	0.8446037	2.57E-23	4.69E-19	0.774	0.377
SLC25A37	6	0.83850094	2.45E-32	4.47E-28	0.928	0.587
CCDC85B	6	0.83404434	4.00E-23	7.30E-19	0.918	0.713
IER3	6	0.83208505	7.48E-13	1.37E-08	0.553	0.246
WTAP	6	0.7968952	8.25E-27	1.50E-22	0.865	0.455
NRP2	6	0.78540896	3.55E-21	6.48E-17	0.588	0.162
ERO1A	6	0.77472225	1.46E-26	2.67E-22	0.796	0.419
SPRY2	6	0.76802091	1.93E-27	3.53E-23	0.629	0.114
NDRG1	6	0.7614762	8.59E-23	1.57E-18	0.796	0.437
GSTO1	6	0.75403402	2.62E-28	4.78E-24	0.868	0.533
MIF	6	0.75180624	2.43E-48	4.44E-44	1	1
SLC5A3	6	0.75004323	3.61E-20	6.59E-16	0.792	0.455
EIF4A2	6	0.73938824	9.23E-36	1.68E-31	0.994	0.916
SERPINE2	6	0.71205744	1.01E-18	1.85E-14	1	1
BHLHE40	6	0.70368812	1.03E-14	1.87E-10	0.604	0.299

SNHG12	6	0.68379879	2.28E-22	4.15E-18	0.626	0.186
LINC00152	6	0.66798583	3.18E-18	5.79E-14	0.733	0.401
MT1E	6	0.66060962	5.68E-11	1.04E-06	0.994	0.982
BTG1	6	0.66047084	1.06E-27	1.94E-23	0.987	0.844
RABGGTB	6	0.64472825	6.58E-25	1.20E-20	0.862	0.491
PPP1R15A	6	0.63890365	1.68E-20	3.06E-16	0.626	0.204
CCND1	6	0.63769294	4.33E-19	7.89E-15	0.73	0.329
FOSL1	6	0.63687628	4.78E-16	8.71E-12	0.472	0.102
BZW1	6	0.63059682	3.71E-22	6.77E-18	0.915	0.623
ISG20	6	0.62743643	3.22E-19	5.88E-15	0.752	0.407
FN1	6	0.62611903	9.65E-21	1.76E-16	1	0.988
TREM1	6	0.61963447	3.76E-23	6.86E-19	0.447	0.006
PLAUR	6	0.61357751	6.72E-19	1.23E-14	0.648	0.24
HLA-DRB1	6	0.60587254	1.07E-20	1.96E-16	0.585	0.156
EIF1	6	0.60032156	1.07E-32	1.95E-28	1	1
S100A16	6	0.59899134	2.52E-17	4.60E-13	0.698	0.341
GBE1	6	0.59355359	1.89E-13	3.44E-09	0.723	0.461
HMGA2	6	0.59270506	2.87E-27	5.23E-23	0.525	0.024
NRN1	6	0.58755518	2.97E-11	5.42E-07	0.607	0.299
VAPA	6	0.5809334	5.67E-25	1.04E-20	0.978	0.784
ADSS	6	0.57328007	1.56E-16	2.85E-12	0.726	0.443
PPP3CA	6	0.5706976	6.80E-13	1.24E-08	0.899	0.635
SLC2A1	6	0.56605022	9.42E-15	1.72E-10	0.487	0.144
MT1G	6	0.56507432	2.46E-12	4.50E-08	0.959	0.76
ENO2	6	0.56198777	3.54E-18	6.46E-14	0.692	0.323
PPP1CB	6	0.55696972	1.03E-10	1.88E-06	0.821	0.629
CA9	6	0.55654417	1.29E-16	2.35E-12	0.525	0.156
FAM162A	6	0.54822896	4.09E-19	7.46E-15	0.934	0.743
TIMP3	6	0.54699428	1.49E-06	0.02726048	0.682	0.509
SLC20A1	6	0.54636227	1.58E-15	2.89E-11	0.55	0.192
RELB	6	0.53626827	2.34E-18	4.27E-14	0.484	0.096
RNF114	6	0.53428845	1.18E-16	2.15E-12	0.692	0.371
GDF15	6	0.53242209	3.73E-07	0.00680914	0.223	0.048
GJA1	6	0.52118602	1.22E-17	2.22E-13	0.447	0.072
DDIT3	6	0.51414682	1.03E-12	1.87E-08	0.692	0.359
DDIT4L	6	0.51229306	2.84E-16	5.17E-12	0.824	0.467
ZNF593	6	0.51209567	3.14E-20	5.72E-16	0.739	0.365
ANGPTL4	6	0.50281311	5.98E-12	1.09E-07	0.412	0.114
TFPI	6	0.50083547	1.08E-10	1.97E-06	0.377	0.108

CHI3L2	6	0.48892236	8.23E-07	0.01501012	0.597	0.407
TMSB10	6	0.4886482	7.68E-15	1.40E-10	1	0.994
RND3	6	0.48501393	4.42E-12	8.07E-08	0.374	0.084
FABP5	6	0.47747836	1.23E-06	0.02251441	0.667	0.491
AKAP12	6	0.47653363	3.24E-16	5.91E-12	0.406	0.054
BNIP3	6	0.47346334	6.62E-18	1.21E-13	0.997	0.976
PDLIM4	6	0.47088961	2.33E-14	4.26E-10	0.934	0.79
RP11-367F23.2	6	0.47039663	1.81E-06	0.03301734	0.126	0
CD44	6	0.46852143	1.58E-15	2.88E-11	0.921	0.659
EVI2A	6	0.46736368	5.81E-17	1.06E-12	0.447	0.078
PTGES	6	0.46232175	1.28E-15	2.33E-11	0.349	0.024
SGIP1	6	0.45643619	1.50E-14	2.73E-10	0.374	0.054
GPRC5A	6	0.45102115	2.06E-14	3.75E-10	0.437	0.096
TNFSF11	6	0.45060214	8.09E-15	1.48E-10	0.308	0.006
BCL2A1	6	0.45055471	7.77E-12	1.42E-07	0.239	0
EIF4EBP1	6	0.45031563	5.32E-10	9.71E-06	0.789	0.635
ARF6	6	0.44491609	2.62E-13	4.77E-09	0.72	0.431
ENO1	6	0.44108238	3.69E-07	0.00673442	0.987	0.982
CSGALNACT2	6	0.43905053	1.08E-13	1.97E-09	0.481	0.156
PPFIBP1	6	0.43849508	5.09E-12	9.29E-08	0.689	0.401
PDE4B	6	0.43704114	2.03E-11	3.70E-07	0.418	0.132
VDAC1	6	0.43290885	3.50E-13	6.39E-09	0.887	0.784
TMEM38B	6	0.43187801	1.59E-13	2.90E-09	0.594	0.269
MT1M	6	0.43017516	3.30E-07	0.00601889	0.638	0.449
LINC00493	6	0.42624713	4.01E-15	7.33E-11	0.921	0.719
GRB10	6	0.4228689	2.41E-16	4.40E-12	0.362	0.024
RNF126	6	0.41921535	6.00E-14	1.09E-09	0.475	0.144
TFPI2	6	0.41612734	2.48E-07	0.00452281	0.157	0.006
LTBP1	6	0.41571488	2.37E-14	4.33E-10	0.362	0.048
EPS8	6	0.41428082	4.20E-12	7.67E-08	0.566	0.257
PTGES3	6	0.41065277	2.07E-13	3.77E-09	0.94	0.916
RNF145	6	0.41059159	2.80E-08	0.00051031	0.487	0.287
FHL2	6	0.40554714	3.82E-10	6.97E-06	0.553	0.287
COX17	6	0.39724737	1.21E-11	2.20E-07	0.855	0.623
COPS2	6	0.39582943	8.70E-10	1.59E-05	0.638	0.419
FAP	6	0.3911851	7.89E-12	1.44E-07	0.415	0.12
ESD	6	0.39116214	1.83E-08	0.00033308	0.808	0.659
MSANTD3	6	0.38851824	1.34E-11	2.45E-07	0.443	0.15
EIF1B	6	0.3880452	2.43E-12	4.44E-08	0.78	0.527

SMS	6	0.38673212	3.66E-09	6.68E-05	0.711	0.497
MIR4435-2HG	6	0.38271329	1.01E-08	0.00018441	0.5	0.251
SAT2	6	0.38266749	4.21E-10	7.69E-06	0.799	0.569
NBPF14	6	0.38044675	1.56E-11	2.85E-07	0.355	0.078
FNIP2	6	0.37740409	5.05E-09	9.21E-05	0.469	0.228
SPHK1	6	0.37345469	1.40E-10	2.56E-06	0.365	0.102
EPB41L4A-AS1	6	0.37221008	8.15E-11	1.49E-06	0.887	0.754
CAMK2D	6	0.37218372	3.67E-10	6.70E-06	0.497	0.222
METAP2	6	0.37165463	3.89E-10	7.11E-06	0.799	0.569
ARPP19	6	0.37139691	9.07E-09	0.00016547	0.635	0.407
TBCA	6	0.37139454	3.31E-13	6.04E-09	0.969	0.868
SQSTM1	6	0.37048061	2.95E-08	0.00053821	0.928	0.844
ZNF385D	6	0.36948752	1.17E-08	0.00021395	0.72	0.497
PTP4A1	6	0.36754334	2.31E-07	0.00422062	0.582	0.371
WSB1	6	0.36705708	4.99E-08	0.00090968	0.761	0.611
SH3BP5	6	0.36423208	9.45E-10	1.73E-05	0.522	0.263
CLDND1	6	0.36227026	1.35E-10	2.46E-06	0.629	0.389
KDM7A	6	0.36052577	4.95E-12	9.04E-08	0.377	0.09
ABL2	6	0.35641868	2.08E-12	3.79E-08	0.437	0.132
TMEM45A	6	0.35396408	1.30E-07	0.00237381	0.887	0.749
PGK1	6	0.34831893	3.32E-07	0.00606107	0.918	0.916
SLC3A2	6	0.34778674	5.44E-07	0.00993352	0.481	0.281
SYF2	6	0.34704927	1.14E-09	2.09E-05	0.808	0.611
SH3GLB1	6	0.34476716	1.83E-10	3.35E-06	0.783	0.527
KLF3	6	0.34235309	5.31E-07	0.00968986	0.368	0.162
TNC	6	0.341846	2.58E-10	4.71E-06	0.261	0.03
ITGA5	6	0.34122603	9.32E-12	1.70E-07	0.428	0.12
ATF4	6	0.33586383	1.17E-10	2.14E-06	0.95	0.82
WDR45B	6	0.33376235	8.27E-09	0.00015087	0.459	0.21
PFKP	6	0.3326649	1.01E-07	0.00185177	0.544	0.311
SLC7A2	6	0.3312315	7.88E-10	1.44E-05	0.299	0.06
PLIN2	6	0.33089507	9.97E-09	0.0001819	0.377	0.138
RIPK2	6	0.32751848	7.75E-09	0.0001415	0.362	0.126
SFT2D1	6	0.32457853	1.64E-09	3.00E-05	0.843	0.677
EMP3	6	0.32340435	1.86E-09	3.40E-05	0.987	0.958
CHMP4B	6	0.3219566	2.87E-08	0.00052372	0.73	0.533
KCTD9	6	0.32154648	4.15E-10	7.58E-06	0.336	0.084
NPR3	6	0.32102333	5.87E-11	1.07E-06	0.233	0.006
EIF5	6	0.32089565	1.65E-07	0.00301246	0.792	0.605

UBE2B	6	0.3204999	7.35E-10	1.34E-05	0.836	0.635
CYGB	6	0.31915939	1.05E-09	1.91E-05	0.305	0.066
MEDAG	6	0.31865272	5.44E-08	0.00099309	0.28	0.072
CLMP	6	0.31664292	8.25E-10	1.51E-05	0.459	0.192
C11orf31	6	0.31659527	1.89E-07	0.00344917	0.821	0.695
PPP1R14C	6	0.31462094	3.77E-08	0.00068861	0.365	0.138
BTG3	6	0.31400257	4.82E-08	0.00087947	0.459	0.222
LRRFIP1	6	0.31340292	3.50E-08	0.00063881	0.654	0.425
HPCAL1	6	0.3133751	1.62E-06	0.02955109	0.371	0.18
AK2	6	0.30795124	6.17E-07	0.01125103	0.761	0.629
NDUFB4	6	0.30530103	7.72E-08	0.00140787	0.903	0.79
CCT8	6	0.30358369	3.80E-07	0.00693613	0.711	0.563
CLK1	6	0.30242956	1.57E-09	2.86E-05	0.619	0.377
CAPZA2	6	0.29869557	9.10E-07	0.01660698	0.78	0.623
AKIRIN2	6	0.29704833	9.17E-09	0.00016726	0.409	0.162
ATP6V1C1	6	0.29508562	1.77E-10	3.22E-06	0.443	0.156
FERMT2	6	0.29459007	9.00E-07	0.01642681	0.5	0.293
ARRDC3	6	0.29388388	2.81E-07	0.00512021	0.531	0.293
SMIM3	6	0.29351056	3.43E-09	6.26E-05	0.34	0.096
TMEM18	6	0.29338199	5.43E-08	0.00099049	0.635	0.401
CIRBP	6	0.29253843	1.32E-10	2.42E-06	0.987	0.964
NOTCH2	6	0.29175958	3.98E-08	0.00072633	0.487	0.257
RHOC	6	0.29115216	1.36E-07	0.0024774	0.909	0.796
MRPL51	6	0.28529935	7.43E-08	0.00135524	0.874	0.76
ERRFI1	6	0.28519231	4.81E-08	0.00087748	0.491	0.246
C16orf13	6	0.2842973	4.12E-07	0.00752625	0.836	0.671
TNS1	6	0.2839292	1.99E-09	3.62E-05	0.365	0.114
CD58	6	0.28248966	1.73E-07	0.00315987	0.365	0.15
C2orf49	6	0.27740113	1.37E-08	0.00025019	0.39	0.15
MRPL54	6	0.27659047	2.96E-08	0.0005399	0.535	0.281
RORA	6	0.27417303	5.20E-07	0.00948692	0.421	0.21
PPP1R2	6	0.27403334	5.35E-08	0.00097703	0.522	0.281
RHOD	6	0.27223316	5.50E-07	0.01004034	0.538	0.329
ZNF581	6	0.27223316	4.19E-09	7.65E-05	0.522	0.246
ZFYVE16	6	0.26894909	2.45E-09	4.47E-05	0.333	0.09
PRR34-AS1	6	0.26782787	1.41E-06	0.02569854	0.267	0.09
SAP18	6	0.26558862	2.65E-07	0.00484196	0.899	0.737
IFRD1	6	0.26509311	1.19E-07	0.00217489	0.572	0.323
SLC25A36	6	0.2648591	1.57E-06	0.02866147	0.538	0.323

AC090498.1	6	0.26392025	5.41E-13	9.88E-09	1	1
GNPNAT1	6	0.26386491	8.79E-08	0.00160414	0.362	0.138
TRMT112	6	0.26308397	6.21E-08	0.00113399	0.947	0.874
SQRDL	6	0.26305411	7.60E-07	0.01387251	0.374	0.174
LSM12	6	0.25934255	3.70E-08	0.00067566	0.412	0.168
ARL4C	6	0.25214979	1.78E-09	3.25E-05	0.236	0.024
CNIH1	6	0.25122188	5.87E-07	0.01071694	0.843	0.623
CDKN2B	6	0.25111624	6.91E-07	0.01261198	0.343	0.138
ERLEC1	6	-0.2502266	8.65E-07	0.01579033	0.365	0.575
IFT20	6	-0.2502431	3.01E-08	0.00054956	0.179	0.401
AFF3	6	-0.2509003	5.52E-15	1.01E-10	0.016	0.228
CHP1	6	-0.251015	7.07E-07	0.01289363	0.22	0.419
ANGPTL5	6	-0.2512242	2.96E-09	5.40E-05	0.101	0.311
ZCRB1	6	-0.2540011	6.02E-08	0.00109828	0.393	0.629
PRNP	6	-0.2544755	6.07E-09	0.0001107	0.214	0.461
ABHD14A	6	-0.2547337	6.26E-12	1.14E-07	0.063	0.287
SCP2	6	-0.2558343	1.45E-08	0.00026468	0.333	0.587
PRDX1	6	-0.2560911	3.60E-07	0.00656585	0.836	0.922
RPL31	6	-0.2582605	5.82E-13	1.06E-08	1	1
TMEM219	6	-0.258486	1.02E-06	0.01867156	0.61	0.737
MATN4	6	-0.2589125	4.48E-08	0.00081727	0.053	0.216
TSC22D3	6	-0.259481	5.14E-08	0.000937	0.17	0.383
CCDC88A	6	-0.2605111	2.09E-11	3.82E-07	0.116	0.371
CCNG1	6	-0.2612179	3.94E-07	0.00719168	0.289	0.503
FRMD4B	6	-0.2612563	1.40E-10	2.55E-06	0.088	0.311
MAGED1	6	-0.2618493	1.19E-10	2.17E-06	0.066	0.275
EFHD1	6	-0.2618493	2.17E-10	3.96E-06	0.069	0.275
CUTA	6	-0.2620038	2.71E-07	0.00494694	0.695	0.856
RPS25	6	-0.2627162	1.28E-16	2.34E-12	1	1
SNRPN	6	-0.2628151	1.77E-06	0.03235746	0.503	0.701
RP11-71N10.1	6	-0.2631336	9.46E-15	1.73E-10	0.013	0.216
GPAA1	6	-0.2634076	4.39E-07	0.00801226	0.217	0.419
CD63	6	-0.263458	1.76E-06	0.0321417	1	1
NDUFB11	6	-0.2643509	2.68E-08	0.00048975	0.657	0.838
C10orf54	6	-0.2645679	2.68E-12	4.89E-08	0.069	0.305
CTSC	6	-0.2649792	2.07E-06	0.0376895	0.123	0.293
BBX	6	-0.2651595	6.71E-10	1.22E-05	0.201	0.455
CREG1	6	-0.2655371	2.54E-07	0.00463399	0.362	0.581
CSGALNACT1	6	-0.2666504	1.66E-08	0.00030309	0.135	0.347

FKBP11	6	-0.2673419	8.74E-10	1.59E-05	0.119	0.347
TCEB2	6	-0.2674227	1.92E-09	3.50E-05	0.947	0.982
HMGN3	6	-0.2685141	1.84E-09	3.36E-05	0.236	0.503
PRUNE2	6	-0.2686084	7.92E-08	0.00144497	0.123	0.317
AC003090.1	6	-0.2690826	7.82E-20	1.43E-15	0.016	0.287
CYBRD1	6	-0.2691979	4.33E-08	0.00079027	0.481	0.677
ATP5A1	6	-0.2695427	1.34E-07	0.00244938	0.437	0.635
BTF3	6	-0.2703636	1.79E-10	3.27E-06	0.991	1
FAAP20	6	-0.2705801	1.45E-08	0.00026416	0.443	0.701
RPL27A	6	-0.2715655	4.29E-22	7.83E-18	1	1
RPL36A	6	-0.2724612	5.44E-07	0.00993309	0.962	0.988
CALM3	6	-0.2725744	4.52E-08	0.00082535	0.519	0.731
RPS24	6	-0.272723	2.38E-15	4.34E-11	1	1
TRPS1	6	-0.2744753	4.27E-07	0.0078003	0.368	0.587
RPL15	6	-0.275137	1.32E-23	2.40E-19	1	1
HINT2	6	-0.2755234	3.69E-08	0.00067314	0.242	0.467
MTDH	6	-0.2758061	1.40E-06	0.02557388	0.566	0.713
TSPAN3	6	-0.2765652	2.60E-07	0.00475286	0.346	0.563
COX6C	6	-0.2769274	1.57E-07	0.00286881	0.83	0.934
RP5-1097P24.1	6	-0.2779234	3.94E-10	7.19E-06	0.088	0.305
OST4	6	-0.2783467	8.69E-13	1.59E-08	0.994	1
EBPL	6	-0.2785252	1.82E-09	3.32E-05	0.23	0.479
RPS4Y1	6	-0.2785978	1.85E-10	3.37E-06	0.991	0.988
TCEAL3	6	-0.2794959	2.05E-07	0.00374929	0.371	0.599
RPL13	6	-0.2800549	2.11E-22	3.84E-18	1	1
RHOB	6	-0.2809655	7.68E-13	1.40E-08	0.053	0.281
IDI1	6	-0.2813174	4.62E-11	8.42E-07	0.151	0.419
MPP6	6	-0.2818012	6.36E-08	0.00116079	0.201	0.407
S100A13	6	-0.282059	1.93E-11	3.53E-07	0.991	0.994
ZIC1	6	-0.2827907	1.79E-10	3.27E-06	0.072	0.281
TCEA2	6	-0.2834757	2.26E-12	4.12E-08	0.201	0.515
CMB9-22P13.1	6	-0.284367	1.62E-08	0.00029508	0.261	0.497
SLC26A2	6	-0.2845296	7.82E-11	1.43E-06	0.085	0.311
CTSD	6	-0.2855925	6.06E-07	0.01106618	0.783	0.916
RPL7A	6	-0.2863525	3.94E-18	7.19E-14	1	1
PKM	6	-0.2875936	4.76E-07	0.00867792	0.871	0.976
RNASET2	6	-0.2896239	1.20E-10	2.18E-06	0.17	0.431
SLC38A1	6	-0.2897476	7.81E-11	1.43E-06	0.119	0.359
DUT	6	-0.2899304	6.66E-08	0.00121438	0.462	0.677

TCEAL8	6	-0.291837	2.39E-07	0.00436721	0.613	0.766
SEC11C	6	-0.2927844	9.53E-09	0.00017382	0.242	0.479
WWP2	6	-0.2933625	1.45E-08	0.00026535	0.318	0.581
CALM2	6	-0.2933891	1.78E-07	0.00324644	0.962	0.982
ARPC1A	6	-0.2934785	7.33E-09	0.00013373	0.447	0.671
RPL5	6	-0.2939056	1.31E-16	2.39E-12	1	1
OTOS	6	-0.294521	5.15E-16	9.39E-12	0.057	0.335
CD81	6	-0.2957509	1.36E-07	0.00247481	0.22	0.437
GTF3A	6	-0.2963113	1.13E-09	2.06E-05	0.311	0.569
TIMP4	6	-0.2964432	4.18E-12	7.63E-08	0.119	0.383
COPZ2	6	-0.2969141	1.71E-11	3.12E-07	0.116	0.365
PPA1	6	-0.2981339	1.09E-06	0.01981513	0.387	0.575
C1orf122	6	-0.2985559	3.20E-09	5.83E-05	0.302	0.557
SOCS3	6	-0.2988991	1.37E-06	0.02502371	0.135	0.311
SSR3	6	-0.2989434	2.52E-07	0.00459659	0.78	0.91
CKAP4	6	-0.3008791	2.46E-10	4.48E-06	0.204	0.455
SOD1	6	-0.3009289	1.47E-10	2.67E-06	0.925	0.988
RPS21	6	-0.3011128	9.49E-17	1.73E-12	1	1
HMGB1	6	-0.3014466	5.68E-09	0.00010361	0.708	0.874
RPL21	6	-0.3025989	4.34E-22	7.92E-18	1	1
RPL22	6	-0.3030177	1.89E-15	3.45E-11	1	1
PLPP1	6	-0.3030425	3.84E-07	0.00700163	0.11	0.281
UQCRB	6	-0.3037318	3.12E-07	0.00568561	0.956	0.964
SLC44A1	6	-0.3043191	1.09E-10	1.99E-06	0.142	0.389
TMEM14A	6	-0.3047511	5.57E-12	1.02E-07	0.258	0.569
C9orf16	6	-0.3055466	2.81E-07	0.00513225	0.358	0.581
UGP2	6	-0.3057786	1.45E-08	0.00026424	0.465	0.701
NME4	6	-0.30579	4.21E-09	7.69E-05	0.296	0.539
NFIC	6	-0.3061858	1.28E-06	0.02335513	0.283	0.467
TOMM7	6	-0.3082629	1.01E-19	1.84E-15	1	1
ATRNL1	6	-0.3093433	2.11E-14	3.85E-10	0.072	0.335
UBA52	6	-0.3098061	4.17E-22	7.62E-18	1	1
SRM	6	-0.30982	3.60E-09	6.58E-05	0.327	0.581
C1orf21	6	-0.3107549	6.92E-09	0.00012634	0.286	0.533
COMMD6	6	-0.3109014	9.47E-16	1.73E-11	0.991	1
MYL12B	6	-0.3112316	8.67E-08	0.00158212	0.591	0.743
DMKN	6	-0.3116495	7.51E-11	1.37E-06	0.305	0.587
SSR2	6	-0.3120949	4.27E-10	7.79E-06	0.796	0.934
ERP29	6	-0.3122043	3.70E-09	6.76E-05	0.616	0.778

VIM	6	-0.312259	4.41E-07	0.00805038	0.994	1
LUC7L3	6	-0.3122802	1.79E-10	3.26E-06	0.261	0.539
RPL29	6	-0.3128127	1.13E-24	2.05E-20	1	1
GRN	6	-0.312963	1.05E-12	1.92E-08	0.16	0.455
RPS3	6	-0.3142583	7.47E-26	1.36E-21	1	1
CFL1	6	-0.3151436	1.36E-07	0.00248187	0.833	0.88
AK1	6	-0.3153319	1.75E-07	0.00319934	0.739	0.898
BHLHE41	6	-0.3158413	2.11E-10	3.86E-06	0.151	0.407
RRBP1	6	-0.3169972	6.46E-09	0.00011779	0.236	0.485
TMEM14C	6	-0.3178165	1.38E-09	2.51E-05	0.516	0.76
SSPN	6	-0.3190577	2.27E-12	4.15E-08	0.119	0.389
ARHGAP29	6	-0.3213844	5.24E-10	9.56E-06	0.308	0.581
FADS1	6	-0.3222699	9.62E-17	1.75E-12	0.057	0.341
RPL6	6	-0.3234601	1.56E-20	2.84E-16	1	1
LSP1	6	-0.3247042	3.60E-07	0.00657132	0.214	0.413
PPIA	6	-0.3260131	3.39E-11	6.18E-07	0.943	1
CP	6	-0.3270712	3.77E-07	0.0068735	0.522	0.707
C9orf3	6	-0.3271051	6.46E-10	1.18E-05	0.211	0.467
ERH	6	-0.3274164	5.49E-10	1.00E-05	0.594	0.808
C1QTNF8	6	-0.3278603	2.42E-14	4.41E-10	0.044	0.281
INHBA	6	-0.3281401	2.20E-12	4.02E-08	0.179	0.479
ENPP1	6	-0.3283625	1.71E-12	3.12E-08	0.11	0.377
RPS9	6	-0.32931	1.49E-25	2.72E-21	1	1
PPP1R3C	6	-0.3297446	3.34E-07	0.00609624	0.327	0.545
NREP	6	-0.3304	1.67E-13	3.05E-09	0.116	0.401
CDK2AP2	6	-0.3305221	2.45E-07	0.00447414	0.255	0.461
EIF3K	6	-0.331037	1.43E-12	2.61E-08	0.802	0.934
RNF130	6	-0.331613	1.20E-08	0.00021826	0.374	0.587
CETN2	6	-0.3317697	3.12E-11	5.70E-07	0.189	0.455
DSTN	6	-0.3327439	2.22E-13	4.05E-09	1	1
HINT1	6	-0.3339557	8.54E-15	1.56E-10	0.972	1
MYEOV2	6	-0.3344176	2.75E-09	5.02E-05	0.632	0.808
NDUFA3	6	-0.3345937	1.46E-09	2.67E-05	0.421	0.659
RPS10	6	-0.3349614	1.72E-14	3.15E-10	0.981	1
RPS7	6	-0.3349996	4.13E-23	7.54E-19	1	1
BRK1	6	-0.3356984	1.13E-10	2.07E-06	0.805	0.97
SLC14A1	6	-0.3360146	1.53E-10	2.79E-06	0.248	0.527
JUN	6	-0.3377782	7.37E-08	0.00134394	0.182	0.389
DST	6	-0.338393	1.62E-10	2.95E-06	0.396	0.653

RPL35	6	-0.3386632	7.90E-26	1.44E-21	1	1
TMED2	6	-0.3392691	3.61E-12	6.58E-08	0.843	0.946
XAGE2	6	-0.3395684	3.84E-09	7.00E-05	0.006	0.126
CRELD2	6	-0.3397166	2.52E-15	4.60E-11	0.079	0.359
RCN3	6	-0.3401289	2.09E-10	3.82E-06	0.195	0.449
CALM1	6	-0.340722	2.49E-10	4.55E-06	0.465	0.725
RCN1	6	-0.3408713	1.21E-10	2.21E-06	0.336	0.617
MOXD1	6	-0.3415024	2.21E-09	4.03E-05	0.261	0.503
SERTAD4-AS1	6	-0.3419452	8.85E-18	1.62E-13	0.088	0.413
CCPG1	6	-0.3436535	8.53E-09	0.00015559	0.371	0.611
MALL	6	-0.343953	2.29E-09	4.17E-05	0.204	0.449
GAS6	6	-0.3443375	7.82E-14	1.43E-09	0.179	0.491
SPTLC3	6	-0.3443661	6.15E-10	1.12E-05	0.123	0.347
ARL2BP	6	-0.3447673	2.96E-10	5.40E-06	0.403	0.665
ITM2B	6	-0.3452022	1.69E-08	0.00030874	0.94	0.988
FCGRT	6	-0.3465037	2.08E-09	3.80E-05	0.412	0.635
DDOST	6	-0.346806	2.28E-11	4.15E-07	0.261	0.545
P4HA1	6	-0.3484881	1.19E-06	0.02178682	0.572	0.701
TSPAN4	6	-0.3496596	1.19E-08	0.00021666	0.418	0.617
CHID1	6	-0.3507098	3.75E-10	6.84E-06	0.374	0.623
TMED9	6	-0.3513546	2.21E-09	4.04E-05	0.522	0.743
VCAN	6	-0.351388	1.25E-11	2.29E-07	0.176	0.461
RPL37A	6	-0.3521774	3.14E-29	5.73E-25	1	1
ZBTB20	6	-0.3527058	1.55E-15	2.82E-11	0.126	0.443
DBI	6	-0.352879	1.47E-08	0.00026872	0.66	0.832
TMED3	6	-0.3529473	3.46E-09	6.31E-05	0.406	0.611
RPS4X	6	-0.353448	1.39E-31	2.54E-27	1	1
RPL18A	6	-0.3541053	6.07E-28	1.11E-23	1	1
RPL8	6	-0.3544231	3.13E-28	5.71E-24	1	1
CHPT1	6	-0.3545523	1.89E-09	3.45E-05	0.465	0.701
PNKD	6	-0.3546646	4.54E-14	8.29E-10	0.189	0.503
ATPIF1	6	-0.3548335	6.87E-10	1.25E-05	0.566	0.778
UQCRH	6	-0.3552711	2.46E-15	4.48E-11	0.921	0.976
HERPUD1	6	-0.3563755	1.84E-09	3.36E-05	0.374	0.611
RPS5	6	-0.3565861	4.61E-26	8.42E-22	1	1
EIF4B	6	-0.3582154	2.17E-10	3.96E-06	0.714	0.874
CMPK1	6	-0.3587368	7.96E-13	1.45E-08	0.654	0.868
PRDX5	6	-0.3587856	5.76E-12	1.05E-07	0.836	0.952
LMAN2	6	-0.3615819	2.38E-10	4.34E-06	0.318	0.581

ENAH	6	-0.3618251	8.34E-13	1.52E-08	0.129	0.401
PRKCDBP	6	-0.3619465	9.17E-10	1.67E-05	0.437	0.671
RPL10	6	-0.3638291	2.92E-32	5.33E-28	0.997	1
SLC1A5	6	-0.3647437	1.84E-14	3.36E-10	0.113	0.401
RPS27A	6	-0.3651281	5.63E-27	1.03E-22	1	1
NDUFA4L2	6	-0.3658861	2.44E-18	4.45E-14	1	0.994
LITAF	6	-0.3667383	4.13E-10	7.53E-06	0.403	0.659
TMEM59	6	-0.367482	2.04E-12	3.73E-08	0.956	0.994
AP2M1	6	-0.369089	1.05E-12	1.92E-08	0.387	0.677
PDIA4	6	-0.3704079	6.98E-12	1.27E-07	0.211	0.497
RCN2	6	-0.3708535	2.84E-09	5.18E-05	0.566	0.76
CTSK	6	-0.3709212	3.28E-16	5.98E-12	0.132	0.449
RPS13	6	-0.3711518	4.36E-24	7.96E-20	1	1
TMEM230	6	-0.3745029	1.04E-11	1.89E-07	0.607	0.832
TIMP2	6	-0.3760413	8.13E-12	1.48E-07	0.538	0.784
RPL18	6	-0.3762154	7.31E-27	1.33E-22	1	1
PFN1	6	-0.3772934	4.00E-12	7.31E-08	0.896	0.964
HLA-C	6	-0.3780281	1.23E-10	2.24E-06	0.802	0.904
LUM	6	-0.3785033	3.32E-16	6.07E-12	0.997	0.994
JTB	6	-0.3795784	4.47E-12	8.16E-08	0.629	0.838
RPS29	6	-0.380701	5.40E-26	9.86E-22	1	1
NAA38	6	-0.3816933	8.23E-12	1.50E-07	0.456	0.713
BEX2	6	-0.3827264	7.41E-09	0.00013527	0.176	0.395
SEC61B	6	-0.3857392	2.06E-12	3.75E-08	0.789	0.94
MEG3	6	-0.386969	2.15E-09	3.92E-05	0.192	0.425
SEPP1	6	-0.3895277	1.77E-17	3.23E-13	0.11	0.437
NINJ1	6	-0.389778	2.13E-13	3.89E-09	0.431	0.749
KDELR3	6	-0.3900368	3.40E-16	6.21E-12	0.126	0.455
MLEC	6	-0.3913849	1.66E-15	3.03E-11	0.264	0.611
LINC01503	6	-0.391985	1.30E-10	2.38E-06	0.157	0.401
PSAP	6	-0.3928949	3.20E-13	5.84E-09	0.777	0.94
RPL23A	6	-0.3932649	8.93E-31	1.63E-26	1	1
OAF	6	-0.3938331	7.48E-15	1.36E-10	0.176	0.497
NENF	6	-0.3940481	7.29E-17	1.33E-12	0.893	0.988
RPS23	6	-0.3990425	1.73E-32	3.16E-28	1	1
RPL37	6	-0.3999302	5.60E-31	1.02E-26	1	1
PPT1	6	-0.4006088	6.23E-17	1.14E-12	0.201	0.557
SPINT2	6	-0.4009315	2.10E-12	3.84E-08	0.242	0.539
RPL19	6	-0.401271	2.47E-36	4.51E-32	1	1

CITED2	6	-0.4015897	5.97E-10	1.09E-05	0.214	0.455
PCBP2	6	-0.4051014	3.29E-20	5.99E-16	0.877	0.976
SRP14	6	-0.4051657	4.63E-14	8.45E-10	0.953	1
MYDGF	6	-0.4063859	1.34E-10	2.44E-06	0.758	0.886
PRSS23	6	-0.4064312	6.34E-07	0.01157096	0.399	0.605
SSR1	6	-0.4065842	1.20E-10	2.19E-06	0.418	0.647
RPS19	6	-0.4075061	5.68E-25	1.04E-20	1	1
CRISPLD1	6	-0.4082956	1.97E-23	3.59E-19	0.003	0.293
RPS6	6	-0.4083023	1.04E-35	1.89E-31	1	1
SCRG1	6	-0.4108354	2.31E-09	4.22E-05	0.953	0.994
IER2	6	-0.4121548	4.03E-08	0.00073478	0.267	0.485
RPS18	6	-0.4135725	9.47E-35	1.73E-30	1	1
CNPY2	6	-0.4140725	6.76E-14	1.23E-09	0.456	0.754
FTL	6	-0.4147216	1.28E-27	2.34E-23	1	1
HMGN2	6	-0.4158597	1.65E-13	3.01E-09	0.723	0.88
SH3BGRL	6	-0.4170001	2.96E-15	5.41E-11	0.286	0.635
CCDC102B	6	-0.4170604	9.40E-25	1.72E-20	0.022	0.359
C14orf1	6	-0.4202453	1.88E-17	3.44E-13	0.151	0.497
RPL9	6	-0.4204674	2.17E-32	3.95E-28	1	1
FKBP10	6	-0.4220075	4.24E-11	7.74E-07	0.327	0.587
NFIA	6	-0.4230364	6.02E-21	1.10E-16	0.094	0.461
C7orf73	6	-0.4233689	3.05E-15	5.56E-11	0.607	0.898
PDE4DIP	6	-0.4264679	6.46E-15	1.18E-10	0.22	0.539
HDLBP	6	-0.4282236	2.43E-16	4.43E-12	0.296	0.635
RPL27	6	-0.4294897	1.36E-27	2.48E-23	1	1
PLXDC2	6	-0.4295401	2.15E-16	3.92E-12	0.208	0.551
COL5A2	6	-0.4300711	7.95E-14	1.45E-09	0.252	0.587
SMIM14	6	-0.4303704	1.85E-15	3.38E-11	0.252	0.587
GSTP1	6	-0.4307476	1.96E-15	3.57E-11	0.943	0.988
SEC11A	6	-0.4309213	3.02E-17	5.50E-13	0.792	0.958
ASPN	6	-0.4320459	1.68E-09	3.07E-05	0.035	0.198
MORF4L2	6	-0.432952	1.09E-13	1.99E-09	0.475	0.749
RPS16	6	-0.4338372	1.45E-31	2.64E-27	1	1
ID4	6	-0.4342752	5.97E-10	1.09E-05	0.368	0.623
KDELR2	6	-0.4414337	2.76E-16	5.04E-12	0.877	0.952
GSN	6	-0.4416256	1.06E-14	1.93E-10	0.283	0.617
PPIB	6	-0.4480406	4.44E-12	8.10E-08	0.865	0.94
LRPAP1	6	-0.4491005	1.29E-16	2.36E-12	0.314	0.647
PEBP1	6	-0.4497074	2.89E-16	5.28E-12	0.67	0.88

RPL3	6	-0.4512254	2.93E-33	5.35E-29	1	1
RPL13A	6	-0.4514293	1.69E-41	3.09E-37	1	1
AHNAK	6	-0.4524748	1.14E-14	2.07E-10	0.377	0.689
GOLIM4	6	-0.4536697	2.45E-13	4.47E-09	0.252	0.563
RPS2	6	-0.4539384	7.96E-37	1.45E-32	1	1
TPM2	6	-0.4539749	1.07E-15	1.96E-11	0.478	0.79
VKORC1	6	-0.4566165	1.07E-13	1.96E-09	0.947	0.982
MSMO1	6	-0.457074	1.98E-12	3.62E-08	0.186	0.461
RPL23	6	-0.4610786	2.69E-30	4.91E-26	1	1
SNAI2	6	-0.4613521	1.28E-12	2.34E-08	0.374	0.671
MALAT1	6	-0.4617399	3.75E-08	0.00068499	1	1
NDUFA11	6	-0.4647749	4.06E-17	7.41E-13	0.824	0.946
H3F3A	6	-0.4681417	1.97E-23	3.60E-19	0.943	0.994
CCNI	6	-0.4700262	5.58E-22	1.02E-17	0.987	1
MT-ND5	6	-0.4796174	5.29E-11	9.66E-07	0.421	0.641
RPL34	6	-0.484525	1.24E-35	2.26E-31	1	1
GNAS	6	-0.484564	8.59E-19	1.57E-14	0.506	0.808
RPL30	6	-0.4863418	6.13E-35	1.12E-30	1	1
TPD52L1	6	-0.4865512	1.02E-18	1.86E-14	0.204	0.581
RPN2	6	-0.4871634	3.55E-24	6.47E-20	0.126	0.545
PABPC1	6	-0.4935277	8.24E-19	1.50E-14	0.742	0.94
TCF4	6	-0.4939053	1.79E-15	3.27E-11	0.223	0.557
ERGIC3	6	-0.4942177	5.08E-21	9.28E-17	0.698	0.946
FTH1	6	-0.496065	3.67E-32	6.70E-28	1	1
NOVA1	6	-0.4972637	1.58E-09	2.88E-05	0.777	0.856
COL11A2	6	-0.4974541	2.42E-17	4.41E-13	0.299	0.641
MYL6	6	-0.4976589	6.21E-20	1.13E-15	0.921	0.988
SDF4	6	-0.4999086	6.01E-15	1.10E-10	0.465	0.707
DDR2	6	-0.5040443	8.12E-17	1.48E-12	0.208	0.551
AHNAK2	6	-0.5047683	3.88E-13	7.07E-09	0.236	0.533
BSG	6	-0.5062936	2.11E-16	3.86E-12	0.513	0.778
RPS12	6	-0.5123986	4.09E-37	7.47E-33	1	1
SERPINA5	6	-0.5136405	1.73E-17	3.16E-13	0.321	0.671
RPLP2	6	-0.5156029	1.20E-47	2.20E-43	1	1
FHL1	6	-0.5162242	5.13E-20	9.36E-16	0.201	0.587
CD9	6	-0.5165308	1.21E-13	2.21E-09	0.462	0.731
CRYAB	6	-0.5181123	1.32E-14	2.41E-10	0.619	0.874
MT-ND4	6	-0.5181911	2.20E-17	4.02E-13	0.953	0.976
FAM46A	6	-0.5207594	1.16E-07	0.0021185	0.374	0.545

RHOBTB3	6	-0.5226244	3.23E-18	5.88E-14	0.296	0.659
RPS3A	6	-0.5231907	1.05E-44	1.91E-40	1	1
EID1	6	-0.5234296	1.62E-20	2.96E-16	0.824	0.964
RPLP1	6	-0.5265939	1.34E-47	2.45E-43	1	1
EEF1B2	6	-0.5287061	3.37E-27	6.15E-23	0.959	0.994
ARL6IP5	6	-0.5300481	1.65E-17	3.02E-13	0.425	0.737
CCDC80	6	-0.533381	4.73E-11	8.63E-07	0.698	0.88
ATRAID	6	-0.5346512	4.70E-20	8.58E-16	0.66	0.904
JUNB	6	-0.536462	2.14E-11	3.90E-07	0.481	0.713
ARPC1B	6	-0.5405169	3.97E-17	7.24E-13	0.428	0.76
NPC2	6	-0.5409892	1.07E-23	1.95E-19	0.943	0.994
NUPR1	6	-0.544155	1.32E-14	2.41E-10	0.947	0.994
PCOLCE2	6	-0.5445393	1.44E-12	2.64E-08	0.792	0.934
SDC2	6	-0.5450975	1.96E-22	3.58E-18	0.635	0.934
RPS17	6	-0.5452898	3.64E-43	6.65E-39	1	1
PDIA3	6	-0.5461647	2.28E-13	4.16E-09	0.409	0.665
CXXC5	6	-0.5474307	1.81E-21	3.30E-17	0.164	0.557
MYLK	6	-0.5562269	4.91E-23	8.95E-19	0.047	0.389
SLC40A1	6	-0.5567647	9.55E-27	1.74E-22	0.016	0.365
S100A1	6	-0.5619224	2.21E-23	4.02E-19	0.991	1
NUCB2	6	-0.5654366	1.02E-21	1.86E-17	0.217	0.617
TTC3	6	-0.566528	1.05E-20	1.91E-16	0.472	0.814
RABAC1	6	-0.5711819	4.34E-27	7.92E-23	0.72	0.946
CALR	6	-0.5731058	1.74E-10	3.17E-06	0.575	0.766
ZFP36L2	6	-0.5745108	1.03E-12	1.87E-08	0.16	0.431
MFGE8	6	-0.574761	3.14E-19	5.72E-15	0.698	0.946
RPL10A	6	-0.5775313	2.28E-48	4.15E-44	1	1
CTGF	6	-0.5874539	1.33E-07	0.00242328	0.324	0.545
SPON2	6	-0.5906324	8.55E-24	1.56E-19	0.063	0.437
PRDX4	6	-0.5918718	7.60E-18	1.39E-13	0.597	0.874
CEBPD	6	-0.5945884	1.48E-12	2.70E-08	0.487	0.754
CRIP2	6	-0.5949406	2.87E-24	5.24E-20	0.314	0.743
SOD3	6	-0.5968134	1.89E-13	3.44E-09	0.94	0.994
HNRNPA1	6	-0.5970515	4.60E-24	8.39E-20	0.959	0.994
HSP90B1	6	-0.5991826	6.18E-19	1.13E-14	0.594	0.904
TPT1	6	-0.6020619	9.23E-46	1.68E-41	1	1
POLR2L	6	-0.6034016	2.66E-19	4.86E-15	0.814	0.952
RPL7	6	-0.6133927	3.41E-42	6.21E-38	1	1
FGFBP2	6	-0.6156054	3.46E-12	6.32E-08	0.855	0.94

RPS8	6	-0.6179191	3.97E-51	7.24E-47	1	1
RPSA	6	-0.6203072	7.29E-35	1.33E-30	0.994	1
FXYD6	6	-0.6313903	3.37E-28	6.15E-24	0.651	0.94
FMOD	6	-0.6321591	3.29E-28	6.00E-24	0.726	0.982
SERF2	6	-0.6343204	7.43E-36	1.36E-31	1	1
SSR4	6	-0.6375429	1.01E-29	1.84E-25	0.928	0.994
FOS	6	-0.6380635	5.88E-17	1.07E-12	0.336	0.683
TMED10	6	-0.6476016	8.29E-30	1.51E-25	0.758	0.988
MDFI	6	-0.6486979	1.78E-25	3.25E-21	0.286	0.695
CRTAP	6	-0.6536638	9.91E-28	1.81E-23	0.453	0.814
TPM1	6	-0.6580438	3.13E-13	5.72E-09	0.623	0.826
DNM3OS	6	-0.6690242	4.32E-33	7.89E-29	0.113	0.605
HTRA1	6	-0.6774972	4.53E-28	8.27E-24	0.588	0.946
APOD	6	-0.678459	6.77E-24	1.24E-19	0.148	0.575
PTRF	6	-0.6794595	4.08E-25	7.45E-21	0.299	0.707
ITM2A	6	-0.6797757	6.37E-24	1.16E-19	0.27	0.683
RPLP0	6	-0.6798361	5.94E-55	1.08E-50	1	1
HSPB1	6	-0.6809621	8.30E-22	1.52E-17	0.553	0.856
EIF3L	6	-0.6813112	1.35E-32	2.47E-28	0.654	0.916
EIF3H	6	-0.6819145	7.18E-37	1.31E-32	0.934	0.994
THY1	6	-0.6826549	2.02E-23	3.68E-19	0.236	0.659
YBX1	6	-0.6835783	2.65E-37	4.83E-33	0.962	0.994
ID2	6	-0.69517	4.30E-17	7.85E-13	0.39	0.707
TSPO	6	-0.7030088	2.57E-32	4.69E-28	0.686	0.946
YBX3	6	-0.7064585	3.70E-29	6.75E-25	0.642	0.94
RPS11	6	-0.7079202	7.94E-50	1.45E-45	0.991	1
RPS20	6	-0.7136477	4.11E-51	7.49E-47	1	1
LECT1	6	-0.7225529	1.87E-21	3.41E-17	0.519	0.898
PTMS	6	-0.7231762	6.64E-32	1.21E-27	0.469	0.886
ITM2C	6	-0.7257356	2.68E-27	4.90E-23	0.286	0.731
S100A4	6	-0.7275174	8.02E-11	1.46E-06	0.792	0.904
ID1	6	-0.7318846	1.58E-16	2.88E-12	0.415	0.76
PRELP	6	-0.7335212	5.19E-27	9.46E-23	0.777	0.964
RPL4	6	-0.7376979	3.68E-47	6.72E-43	0.997	1
COL9A3	6	-0.7434771	5.06E-19	9.24E-15	0.403	0.737
MIA	6	-0.7500958	5.14E-31	9.39E-27	0.95	1
GPX3	6	-0.7565635	1.17E-28	2.14E-24	0.858	0.994
EEF2	6	-0.7614578	3.42E-33	6.24E-29	0.969	1
C2orf40	6	-0.7665712	4.86E-22	8.87E-18	0.965	1

MT-CO3	6	-0.7787997	3.29E-30	6.00E-26	0.862	0.976
MT-CO1	6	-0.7804137	7.29E-25	1.33E-20	0.969	0.988
PDIA6	6	-0.7871584	2.00E-34	3.66E-30	0.472	0.898
MT-ND1	6	-0.8140102	4.50E-31	8.21E-27	0.434	0.856
DCN	6	-0.8178152	2.63E-35	4.80E-31	1	1
MT-ND3	6	-0.8210244	6.66E-30	1.21E-25	0.557	0.892
S100B	6	-0.8214566	1.43E-29	2.61E-25	0.506	0.856
COL11A1	6	-0.8476201	4.16E-31	7.60E-27	0.437	0.91
P4HB	6	-0.849702	1.54E-33	2.82E-29	0.645	0.946
PAX1	6	-0.8565322	2.79E-33	5.09E-29	0.365	0.826
IGFBP7	6	-0.8570414	1.84E-27	3.36E-23	0.701	0.976
EEF1A1	6	-0.879861	7.98E-63	1.46E-58	1	1
NGFRAP1	6	-0.8828965	1.54E-40	2.81E-36	0.648	0.94
CD99	6	-0.8832873	3.00E-42	5.48E-38	0.789	0.994
FBXO2	6	-0.8835375	8.48E-39	1.55E-34	0.418	0.88
MT-ND2	6	-0.8972099	4.75E-32	8.66E-28	0.428	0.85
ACAN	6	-0.8975202	1.34E-34	2.45E-30	0.77	0.976
CST3	6	-0.9076478	1.96E-41	3.57E-37	0.969	1
RBP4	6	-0.9156288	2.01E-30	3.67E-26	0.497	0.892
CYR61	6	-0.9167716	3.15E-28	5.75E-24	0.198	0.677
MT-CYB	6	-0.9307922	1.22E-33	2.23E-29	0.566	0.904
PPIC	6	-0.9428715	1.47E-39	2.68E-35	0.186	0.743
MT-CO2	6	-0.9605992	1.35E-33	2.46E-29	0.934	0.982
ISLR	6	-0.9701938	5.78E-44	1.06E-39	0.167	0.754
MT-ATP6	6	-0.9770459	7.87E-35	1.44E-30	0.698	0.964
B2M	6	-0.9807483	8.96E-52	1.63E-47	0.972	1
ID3	6	-1.0768148	4.55E-33	8.30E-29	0.226	0.743
TSC22D1	6	-1.0982949	3.35E-22	6.11E-18	0.66	0.904
NEAT1	6	-1.100139	4.10E-36	7.48E-32	0.969	0.994
IGFBP6	6	-1.1012989	1.57E-33	2.86E-29	0.484	0.916
ADIRF	6	-1.2351996	5.93E-43	1.08E-38	0.333	0.892
C2orf82	6	-1.2365579	3.23E-36	5.90E-32	0.921	0.988
MSMP	6	-1.2632661	8.91E-08	0.0016256	0.425	0.659
SPARC	6	-1.2687518	4.86E-49	8.88E-45	0.321	0.94
MGP	6	-1.3277503	7.14E-29	1.30E-24	0.997	1
OGN	6	-1.3552551	2.24E-38	4.09E-34	0.777	0.97
CAPS	6	-1.3568132	6.18E-47	1.13E-42	0.447	0.934
SPTSSB	6	-1.5439648	1.58E-59	2.89E-55	0.006	0.671
SLPI	6	-1.5776213	1.79E-39	3.27E-35	0.783	0.982

FRZB	6	-1.5949723	4.90E-36	8.95E-32	0.123	0.629
PLAC9	6	-1.7172205	3.11E-66	5.68E-62	0.456	0.994
CLEC3A	6	-1.8823065	1.35E-68	2.46E-64	0.075	0.838
COL2A1	6	-1.9807193	2.87E-67	5.24E-63	0.248	0.964
**gene**	**cluster**	**avg_logFC**	**p_val**	**p_val_adj**	**pct.1**	**pct.2**
MT2A	7	1.72269245	1.84E-43	3.36E-39	1	0.967
COL1A1	7	1.70124395	4.12E-34	7.52E-30	0.72	0.203
TGFBI	7	1.49108451	5.01E-54	9.15E-50	0.952	0.439
PRG4	7	1.32761838	6.14E-18	1.12E-13	0.57	0.211
IL11	7	1.23826226	3.34E-20	6.10E-16	0.357	0.028
SPP1	7	1.22279844	5.24E-35	9.56E-31	0.599	0.065
CTHRC1	7	1.21537295	4.32E-35	7.88E-31	0.831	0.423
SOD2	7	1.1819939	3.12E-20	5.69E-16	0.763	0.374
SERPINE2	7	1.04633559	3.53E-31	6.44E-27	1	1
IGFBP3	7	1.0429048	5.87E-22	1.07E-17	0.821	0.459
COL3A1	7	0.95662565	1.90E-15	3.46E-11	0.816	0.614
PGF	7	0.90818064	7.57E-19	1.38E-14	0.502	0.138
ENPP1	7	0.84033014	1.14E-11	2.08E-07	0.686	0.516
POSTN	7	0.83590696	3.94E-13	7.19E-09	0.319	0.061
IGFBP5	7	0.72075262	5.71E-07	0.01041161	0.57	0.382
SOX4	7	0.71827593	1.68E-17	3.06E-13	0.531	0.179
GJA1	7	0.71266022	6.95E-30	1.27E-25	0.729	0.24
CTSL	7	0.6892608	4.41E-08	0.00080553	0.614	0.431
COMP	7	0.68411957	3.12E-09	5.69E-05	0.729	0.524
PMEPA1	7	0.68116951	1.20E-23	2.20E-19	0.744	0.321
TM4SF1	7	0.67343828	2.44E-08	0.00044482	0.754	0.581
TNC	7	0.66610145	2.47E-22	4.51E-18	0.758	0.394
LTBP1	7	0.65465435	8.24E-36	1.50E-31	0.676	0.11
VMP1	7	0.65354822	7.74E-18	1.41E-13	0.821	0.549
H2AFZ	7	0.64937415	2.12E-08	0.00038691	0.826	0.805
MT1X	7	0.63824637	2.87E-09	5.23E-05	1	0.955
VEGFA	7	0.6092285	2.33E-11	4.25E-07	0.725	0.488
PHLDA1	7	0.60322953	9.39E-12	1.71E-07	0.401	0.138
UPP1	7	0.60137341	2.70E-08	0.00049248	0.391	0.191
PTGES	7	0.59622717	6.39E-25	1.17E-20	0.531	0.102
ADAMTS6	7	0.58688848	4.10E-19	7.48E-15	0.599	0.224
RND3	7	0.57237333	3.39E-09	6.19E-05	0.377	0.154
NRP2	7	0.56982471	2.77E-15	5.05E-11	0.676	0.341
TMSB4X	7	0.56206612	4.00E-13	7.29E-09	0.995	0.988

COL1A2	7	0.5511175	2.46E-08	0.00044947	0.884	0.854
TNFSF11	7	0.49870426	6.50E-20	1.19E-15	0.324	0.012
PTGS2	7	0.4842591	1.26E-12	2.30E-08	0.343	0.085
TMSB10	7	0.47577387	8.55E-21	1.56E-16	1	1
CCND1	7	0.47392107	4.59E-13	8.38E-09	0.671	0.411
FN1	7	0.47143666	8.83E-16	1.61E-11	1	0.996
HNRNPH1	7	0.46907964	1.17E-06	0.02132686	0.671	0.488
TNFRSF12A	7	0.46789941	2.23E-14	4.08E-10	0.865	0.577
STK17A	7	0.46605852	2.15E-06	0.03917543	0.459	0.297
CRLF1	7	0.4521976	2.61E-13	4.76E-09	0.705	0.423
PLAUR	7	0.44679719	1.30E-13	2.37E-09	0.56	0.248
GNG11	7	0.4464057	4.78E-10	8.72E-06	0.71	0.537
SPOCK1	7	0.4357752	1.91E-17	3.48E-13	0.502	0.146
AC090498.1	7	0.42983561	4.07E-26	7.44E-22	1	0.996
MMP14	7	0.42684688	1.23E-09	2.25E-05	0.565	0.341
BMP2	7	0.42565503	1.65E-13	3.01E-09	0.275	0.033
MT1G	7	0.42058218	2.22E-06	0.04050794	0.729	0.577
IVNS1ABP	7	0.41499854	1.31E-08	0.00023828	0.729	0.569
MT1E	7	0.41285635	1.11E-07	0.00202414	0.976	0.939
JAG1	7	0.41008612	3.78E-14	6.90E-10	0.478	0.171
PPP1R14B	7	0.4000285	4.82E-07	0.00879369	0.778	0.683
TREM1	7	0.39897691	1.77E-15	3.23E-11	0.251	0.008
MIF	7	0.39276676	3.59E-20	6.55E-16	1	1
MIR4435-2HG	7	0.38779146	2.11E-09	3.85E-05	0.758	0.569
FHL2	7	0.38642497	6.02E-08	0.0010982	0.469	0.26
ERO1A	7	0.38144675	1.77E-06	0.03238585	0.565	0.39
LINC00152	7	0.37722164	3.83E-07	0.00699285	0.778	0.687
ACTB	7	0.37479117	3.36E-09	6.14E-05	0.99	0.931
UBE2S	7	0.3744789	9.94E-08	0.00181458	0.411	0.207
ARL4C	7	0.37158593	9.43E-13	1.72E-08	0.338	0.077
APCDD1L	7	0.35927809	2.53E-15	4.62E-11	0.386	0.081
WTAP	7	0.34960969	2.09E-06	0.03805353	0.604	0.455
CYGB	7	0.34653593	1.20E-09	2.19E-05	0.343	0.114
BCL2A1	7	0.3455193	1.06E-07	0.00193496	0.14	0.012
SGIP1	7	0.33568502	7.97E-12	1.45E-07	0.353	0.098
STMN1	7	0.33438735	3.82E-08	0.00069794	0.44	0.207
TFPI2	7	0.33354311	2.60E-07	0.00474146	0.159	0.024
FRMD6	7	0.32950691	1.41E-09	2.57E-05	0.488	0.236
PDE4B	7	0.32729435	6.35E-14	1.16E-09	0.372	0.085

MMP2	7	0.32327302	3.46E-10	6.32E-06	0.15	0
MARCKS	7	0.32093929	1.14E-06	0.02076214	0.512	0.301
TFPI	7	0.31748641	1.65E-07	0.00301291	0.232	0.065
SNRPB	7	0.31150225	5.71E-08	0.00104277	0.58	0.382
PSMA7	7	0.30562099	3.05E-08	0.00055737	0.894	0.776
SLC20A1	7	0.28509073	2.32E-06	0.04234735	0.57	0.362
ANPEP	7	0.28478454	2.38E-11	4.34E-07	0.367	0.106
SMIM3	7	0.2829608	3.65E-10	6.67E-06	0.444	0.183
RPL41	7	0.28167743	6.16E-20	1.12E-15	1	1
CXCL8	7	0.27478092	1.31E-06	0.02385433	0.14	0.02
PCSK1	7	0.26664169	1.25E-06	0.02285813	0.319	0.142
FBLN1	7	0.26620618	4.29E-11	7.82E-07	0.304	0.069
EVI2A	7	0.26422606	2.68E-07	0.00488754	0.271	0.093
FGF1	7	0.26096191	4.16E-16	7.59E-12	0.271	0.012
MEDAG	7	0.26058152	1.37E-08	0.00025034	0.246	0.061
CENPW	7	0.2557055	5.20E-07	0.00948287	0.28	0.102
GADD45GIP1	7	0.25351938	4.88E-07	0.00890525	0.932	0.866
HMGA2	7	0.25236513	5.33E-11	9.72E-07	0.285	0.057
YIPF2	7	-0.2504603	3.15E-07	0.00574062	0.396	0.622
PRPSAP1	7	-0.2505279	8.91E-09	0.00016265	0.155	0.394
C9orf16	7	-0.2510017	2.84E-07	0.00518986	0.512	0.715
SMAD9	7	-0.2515984	8.11E-10	1.48E-05	0.097	0.337
SHISA4	7	-0.2518792	1.91E-06	0.03491293	0.343	0.545
RPS4Y1	7	-0.2525799	3.08E-07	0.00561436	0.87	0.955
NDUFA4	7	-0.2529595	4.49E-08	0.00081857	0.932	0.967
BTF3	7	-0.2531206	1.18E-10	2.16E-06	0.986	0.992
CAPN2	7	-0.2539058	2.78E-08	0.00050676	0.304	0.549
COPZ2	7	-0.2549353	2.23E-07	0.0040601	0.575	0.785
RPL9	7	-0.2551385	1.57E-15	2.86E-11	1	1
PPDPF	7	-0.256933	3.79E-12	6.92E-08	1	1
ARL6IP5	7	-0.2572438	1.79E-09	3.26E-05	0.652	0.841
GPRC5C	7	-0.2572438	2.97E-08	0.00054258	0.256	0.5
SYNGR1	7	-0.2575771	1.36E-08	0.00024846	0.213	0.451
HEBP2	7	-0.2577717	1.63E-06	0.02970901	0.676	0.841
NPC2	7	-0.2593418	1.65E-06	0.03003266	0.87	0.967
SLC25A3	7	-0.2611584	6.69E-08	0.0012207	0.874	0.923
EDF1	7	-0.2614257	1.95E-09	3.55E-05	0.928	0.98
MRAP2	7	-0.2629895	1.50E-13	2.74E-09	0	0.232
RPL7A	7	-0.2641674	2.31E-14	4.21E-10	1	1

TCEAL5	7	-0.2653515	6.27E-12	1.14E-07	0.063	0.325
MT-ND4	7	-0.2664648	9.95E-10	1.82E-05	0.99	0.984
RBM3	7	-0.2665813	2.99E-07	0.00545423	0.768	0.87
DBP	7	-0.2667539	2.76E-08	0.0005034	0.111	0.325
YBX3	7	-0.2677818	1.56E-06	0.0284081	0.715	0.854
VGLL4	7	-0.2683097	3.00E-07	0.00548048	0.396	0.61
SND1	7	-0.2694958	3.08E-10	5.62E-06	0.222	0.508
KMT2E	7	-0.2698576	8.95E-09	0.00016325	0.411	0.679
LINC01420	7	-0.2699298	6.17E-08	0.0011264	0.667	0.87
ANXA1	7	-0.2701238	6.30E-08	0.00114992	0.618	0.817
CST3	7	-0.2713186	9.32E-07	0.01699879	0.961	0.976
RPL31	7	-0.2726177	4.15E-15	7.58E-11	1	1
CRTAP	7	-0.2726983	1.44E-06	0.02631075	0.667	0.78
RPS9	7	-0.2729092	2.11E-17	3.86E-13	1	1
HOOK2	7	-0.2744463	1.36E-08	0.00024747	0.237	0.472
RPL5	7	-0.2745539	4.44E-12	8.10E-08	0.995	1
SNRPN	7	-0.2764355	3.70E-09	6.75E-05	0.609	0.837
SERF2	7	-0.2765072	1.66E-10	3.04E-06	1	1
CCSER2	7	-0.2768527	6.33E-09	0.00011551	0.319	0.565
MAPK10	7	-0.2769124	3.96E-14	7.22E-10	0.072	0.374
CP	7	-0.2769772	2.07E-07	0.00377403	0.145	0.358
HOXA10	7	-0.277636	3.24E-08	0.00059097	0.232	0.467
AFF3	7	-0.2778038	1.36E-08	0.00024893	0.111	0.329
ST13	7	-0.2784629	5.30E-08	0.00096757	0.696	0.85
PFN2	7	-0.2800422	1.37E-06	0.02502055	0.599	0.756
PIM3	7	-0.2801491	1.45E-07	0.00264355	0.13	0.333
N4BP2L2	7	-0.28077	1.39E-08	0.00025438	0.643	0.837
OTOS	7	-0.2810017	4.51E-11	8.22E-07	0.058	0.301
GSN	7	-0.2811304	1.07E-06	0.01958698	0.386	0.573
NR3C1	7	-0.2827795	3.25E-07	0.00592281	0.333	0.545
TMEM230	7	-0.2837686	6.63E-09	0.0001209	0.85	0.935
DHRS3	7	-0.2845244	3.94E-09	7.18E-05	0.227	0.476
KHDRBS3	7	-0.2852204	5.78E-09	0.00010547	0.048	0.244
PLXDC2	7	-0.2870856	2.98E-07	0.00543049	0.391	0.598
QARS	7	-0.2873037	1.21E-10	2.21E-06	0.266	0.561
TALDO1	7	-0.2880596	1.31E-06	0.02384118	0.406	0.593
RAMP1	7	-0.2880681	7.70E-10	1.41E-05	0.077	0.309
RPL13A	7	-0.2893776	4.70E-20	8.58E-16	1	1
HOXC10	7	-0.2904426	6.26E-08	0.00114279	0.469	0.687

PGM1	7	-0.2908106	1.78E-09	3.25E-05	0.415	0.675
SRSF8	7	-0.2914003	2.16E-12	3.94E-08	0.329	0.646
CDC42EP5	7	-0.2914003	1.21E-10	2.21E-06	0.324	0.63
LDHB	7	-0.293099	8.76E-08	0.00159788	0.671	0.793
NKX3-2	7	-0.2935626	6.02E-10	1.10E-05	0.097	0.337
AP2M1	7	-0.2939682	1.77E-08	0.00032252	0.715	0.846
ATP5A1	7	-0.2945779	1.25E-08	0.00022879	0.507	0.72
UGP2	7	-0.2946918	5.97E-08	0.00109015	0.502	0.74
MPC2	7	-0.2947628	1.18E-08	0.00021451	0.589	0.785
ERP29	7	-0.2950947	5.60E-08	0.00102139	0.671	0.809
CKAP4	7	-0.296691	8.29E-08	0.00151327	0.691	0.841
TIMP4	7	-0.2973909	4.10E-10	7.47E-06	0.135	0.386
GARS	7	-0.2978487	8.53E-07	0.01556621	0.28	0.484
MKX	7	-0.2978753	7.06E-08	0.00128767	0.213	0.439
PERP	7	-0.299823	1.11E-08	0.00020309	0.372	0.614
PRR7	7	-0.3032659	1.10E-07	0.0020138	0.353	0.565
EIF3F	7	-0.3035532	1.69E-09	3.08E-05	0.778	0.927
TENM3	7	-0.3036784	6.90E-15	1.26E-10	0.053	0.354
HNRNPA1	7	-0.3039854	2.99E-07	0.00545151	0.986	0.98
C1orf21	7	-0.3042065	4.83E-08	0.0008814	0.478	0.679
SSR2	7	-0.304229	2.01E-11	3.68E-07	0.957	0.963
AGR2	7	-0.3047836	2.19E-07	0.0039988	0.039	0.199
SCIN	7	-0.3052649	8.80E-09	0.00016051	0.198	0.435
RPS4X	7	-0.306072	2.15E-17	3.93E-13	1	1
BEX4	7	-0.3061945	3.10E-09	5.66E-05	0.338	0.622
APLN	7	-0.3069603	3.19E-07	0.00581679	0.058	0.228
PEBP1	7	-0.3107197	4.17E-10	7.61E-06	0.725	0.886
NENF	7	-0.3113656	2.49E-13	4.55E-09	0.899	0.967
RHOA	7	-0.3114581	7.45E-11	1.36E-06	0.903	0.963
CYBRD1	7	-0.314131	4.11E-09	7.50E-05	0.396	0.634
SPON2	7	-0.3146287	4.53E-09	8.27E-05	0.155	0.411
TCEAL8	7	-0.3153111	1.32E-08	0.00024104	0.691	0.882
RPL23	7	-0.3156811	1.05E-19	1.92E-15	0.995	1
FOS	7	-0.3160513	2.07E-07	0.00378006	0.213	0.447
TMEM258	7	-0.3173263	1.36E-14	2.49E-10	0.981	0.996
RPS8	7	-0.3174334	4.11E-25	7.51E-21	1	1
MAP7D3	7	-0.3176756	1.04E-06	0.01902012	0.377	0.569
TPM2	7	-0.320282	1.81E-06	0.0330377	0.87	0.923
PAPSS2	7	-0.3209596	3.81E-08	0.000696	0.353	0.585

CTSD	7	-0.3212498	4.16E-08	0.00075957	0.758	0.874
PDLIM2	7	-0.3213729	7.10E-10	1.30E-05	0.778	0.878
CKB	7	-0.3216836	2.56E-09	4.67E-05	0.135	0.378
OSBPL9	7	-0.3217999	3.46E-07	0.00630641	0.217	0.419
LPP	7	-0.3221679	7.56E-10	1.38E-05	0.324	0.585
ARHGAP29	7	-0.3232364	1.17E-07	0.00213041	0.338	0.561
LPIN1	7	-0.3262264	8.36E-13	1.53E-08	0.13	0.427
EIF4B	7	-0.3272151	2.11E-10	3.84E-06	0.551	0.776
COMMD6	7	-0.3285546	4.69E-17	8.56E-13	0.971	1
RPS3A	7	-0.329202	3.15E-24	5.74E-20	1	1
CLU	7	-0.3295852	3.27E-11	5.96E-07	0.87	0.992
RCN1	7	-0.3308464	6.35E-09	0.00011583	0.449	0.65
RPLP0	7	-0.3311445	1.84E-27	3.36E-23	1	1
MMP24-AS1	7	-0.3314246	3.99E-10	7.27E-06	0.667	0.866
RPL10A	7	-0.3330355	9.03E-21	1.65E-16	1	1
TNS2	7	-0.3338932	2.53E-14	4.61E-10	0.111	0.431
HDLBP	7	-0.3340549	6.67E-11	1.22E-06	0.725	0.902
DAP	7	-0.3373429	2.40E-11	4.38E-07	0.652	0.886
NFIC	7	-0.3377455	3.02E-10	5.51E-06	0.589	0.833
HSP90AB1	7	-0.33937	6.93E-10	1.27E-05	0.807	0.955
TSC22D4	7	-0.3396988	7.39E-13	1.35E-08	0.261	0.581
SH3BGRL	7	-0.3406669	6.23E-10	1.14E-05	0.599	0.805
TCF4	7	-0.3414596	6.09E-09	0.00011109	0.517	0.736
PLCD3	7	-0.3426672	8.36E-17	1.53E-12	0.082	0.427
RPL30	7	-0.3440253	3.49E-24	6.37E-20	1	1
TSPAN4	7	-0.3443953	8.38E-10	1.53E-05	0.527	0.76
ERGIC3	7	-0.3451654	4.19E-10	7.65E-06	0.754	0.866
GPX3	7	-0.3467417	2.82E-07	0.00515306	0.831	0.955
ATRNL1	7	-0.3467966	3.45E-14	6.29E-10	0.106	0.423
DDR2	7	-0.3474991	3.96E-10	7.23E-06	0.469	0.699
TUBB2B	7	-0.3490489	5.18E-08	0.00094443	0.169	0.398
CMB9-22P13.1	7	-0.3491914	6.84E-09	0.00012476	0.386	0.614
RPL18	7	-0.349294	1.34E-25	2.44E-21	1	1
NDUFA4L2	7	-0.3528543	8.29E-13	1.51E-08	0.986	1
C19orf60	7	-0.3531015	2.71E-12	4.94E-08	0.517	0.789
SRP14	7	-0.3545046	4.37E-15	7.97E-11	0.976	0.996
SMIM14	7	-0.3553165	8.17E-11	1.49E-06	0.536	0.764
MALL	7	-0.3574948	3.87E-07	0.00706531	0.585	0.76
CSDE1	7	-0.3577257	1.47E-13	2.68E-09	0.638	0.878

ACTG1	7	-0.3597965	1.31E-07	0.00239612	0.932	0.98
TTC3	7	-0.3623488	9.12E-12	1.66E-07	0.845	0.976
FXYD6	7	-0.3655973	4.51E-12	8.22E-08	0.816	0.959
LITAF	7	-0.3656119	1.16E-12	2.11E-08	0.425	0.72
SERPINA5	7	-0.3658963	2.15E-07	0.00393096	0.415	0.614
VCAN	7	-0.3670333	1.70E-07	0.00310434	0.681	0.837
TMED10	7	-0.3752727	4.05E-13	7.39E-09	0.797	0.959
GNB2L1	7	-0.375962	1.33E-15	2.43E-11	0.99	1
SLC14A1	7	-0.3779203	8.52E-15	1.56E-10	0.121	0.451
AHNAK	7	-0.3784598	1.61E-12	2.93E-08	0.691	0.898
FTH1	7	-0.3801504	1.16E-26	2.12E-22	1	1
COL5A2	7	-0.3805859	2.91E-08	0.00053049	0.865	0.972
HSPB6	7	-0.3807725	5.44E-12	9.92E-08	0.242	0.545
PPP1R3C	7	-0.381698	9.14E-08	0.00166814	0.353	0.589
CRISPLD1	7	-0.3817947	7.01E-07	0.01278367	0.063	0.224
DDIT4	7	-0.3831506	1.67E-08	0.0003054	0.415	0.679
VAMP5	7	-0.3873819	7.58E-13	1.38E-08	0.556	0.793
KAZALD1	7	-0.3920721	4.54E-15	8.28E-11	0.193	0.537
ITGA11	7	-0.3934982	1.48E-11	2.70E-07	0.304	0.581
AOC2	7	-0.3947651	3.08E-13	5.62E-09	0.063	0.337
MEF2C	7	-0.395892	9.46E-11	1.73E-06	0.193	0.459
DNAJC22	7	-0.3959717	2.14E-09	3.90E-05	0.068	0.28
SIX1	7	-0.3982449	1.24E-11	2.26E-07	0.246	0.549
PKM	7	-0.3995558	4.64E-12	8.47E-08	0.947	0.988
HSPA8	7	-0.4025915	4.04E-10	7.37E-06	0.517	0.74
MAGED1	7	-0.403518	2.63E-10	4.79E-06	0.425	0.687
TSC22D3	7	-0.4057589	1.71E-11	3.12E-07	0.174	0.451
NFIA	7	-0.4064211	1.27E-13	2.31E-09	0.372	0.663
RPL3	7	-0.4072275	1.39E-24	2.53E-20	1	1
ID3	7	-0.4091264	1.11E-06	0.02025896	0.739	0.911
PNISR	7	-0.4104167	1.23E-15	2.24E-11	0.623	0.866
TKT	7	-0.4107948	1.50E-09	2.73E-05	0.444	0.703
ID4	7	-0.410802	1.36E-07	0.00248145	0.512	0.728
C1QTNF8	7	-0.4142197	1.41E-18	2.57E-14	0.116	0.496
GPC6	7	-0.4165239	4.08E-16	7.45E-12	0.415	0.756
TBL1XR1	7	-0.4178442	1.16E-07	0.00212336	0.522	0.699
CRYAB	7	-0.4191916	1.08E-12	1.97E-08	0.787	0.923
CD68	7	-0.422896	5.75E-13	1.05E-08	0.488	0.772
RSPO2	7	-0.4276024	2.86E-11	5.22E-07	0.101	0.362

FAM46A	7	-0.4304626	5.27E-09	9.62E-05	0.599	0.785
P4HB	7	-0.4305927	2.60E-11	4.75E-07	0.928	0.984
CCDC102B	7	-0.4376468	9.37E-20	1.71E-15	0.058	0.431
B3GNT7	7	-0.4380134	5.16E-07	0.0094088	0.275	0.484
RPL4	7	-0.4382423	1.52E-28	2.77E-24	0.99	1
CXXC5	7	-0.4418349	7.59E-14	1.39E-09	0.58	0.829
LINC01503	7	-0.4455714	6.73E-11	1.23E-06	0.271	0.533
DMKN	7	-0.4464265	4.07E-18	7.43E-14	0.155	0.541
DNM3OS	7	-0.4482739	5.32E-11	9.70E-07	0.58	0.768
DSTN	7	-0.4498884	4.77E-22	8.71E-18	1	1
TPT1	7	-0.4512573	1.50E-33	2.73E-29	1	1
NPDC1	7	-0.4567924	6.34E-19	1.16E-14	0.169	0.565
ENAH	7	-0.4572854	2.90E-18	5.28E-14	0.406	0.752
SMIM5	7	-0.4574505	4.61E-18	8.41E-14	0.092	0.459
SLC38A1	7	-0.4580771	4.45E-21	8.13E-17	0.213	0.638
HRCT1	7	-0.4613715	2.11E-10	3.85E-06	0.116	0.366
ZBTB20	7	-0.4640442	4.81E-20	8.78E-16	0.246	0.638
GAPDH	7	-0.4722883	3.61E-21	6.60E-17	1	1
S100A4	7	-0.475819	6.59E-12	1.20E-07	0.971	0.988
RPL7	7	-0.4786017	2.37E-32	4.32E-28	1	1
S100A6	7	-0.4797065	9.97E-17	1.82E-12	1	1
CRIP2	7	-0.4844384	5.34E-15	9.75E-11	0.575	0.841
MOXD1	7	-0.4856877	2.07E-21	3.79E-17	0.053	0.447
HIST1H1C	7	-0.486776	2.51E-12	4.58E-08	0.15	0.439
PTRF	7	-0.4882736	1.69E-17	3.09E-13	0.744	0.919
AHNAK2	7	-0.4882736	5.71E-16	1.04E-11	0.184	0.533
METRN	7	-0.4899866	4.85E-17	8.85E-13	0.44	0.78
S100A13	7	-0.4912187	4.76E-23	8.68E-19	0.986	1
MT-CO1	7	-0.4925959	6.19E-21	1.13E-16	1	0.984
NINJ1	7	-0.4929465	1.66E-17	3.04E-13	0.242	0.638
MT-CO3	7	-0.494933	8.91E-24	1.63E-19	0.976	0.98
SEPP1	7	-0.4976501	1.31E-20	2.38E-16	0.053	0.439
MT-ATP6	7	-0.5010322	3.49E-21	6.36E-17	0.952	0.984
LECT1	7	-0.5024155	4.30E-10	7.85E-06	0.348	0.626
OAF	7	-0.505476	1.24E-19	2.26E-15	0.488	0.821
MT-ND1	7	-0.5055865	2.96E-17	5.40E-13	0.87	0.963
EIF3L	7	-0.5080861	1.74E-19	3.17E-15	0.705	0.886
LOXL1	7	-0.5082212	3.16E-13	5.77E-09	0.309	0.606
LIMCH1	7	-0.5105243	1.42E-14	2.59E-10	0.188	0.504

PNRC1	7	-0.5111093	3.91E-16	7.13E-12	0.662	0.894
SDC2	7	-0.5155291	1.17E-18	2.13E-14	0.816	0.967
C6orf48	7	-0.517098	3.69E-13	6.73E-09	0.758	0.923
SCRG1	7	-0.5176672	6.20E-08	0.00113188	0.942	0.967
COL14A1	7	-0.5183676	2.01E-13	3.67E-09	0.097	0.39
FRZB	7	-0.5183954	4.55E-10	8.30E-06	0.077	0.305
FXYD1	7	-0.5184832	1.66E-16	3.03E-12	0.155	0.508
CCDC80	7	-0.5205344	1.07E-07	0.00195769	0.831	0.931
RHOBTB3	7	-0.5263824	2.18E-16	3.98E-12	0.469	0.764
PRSS23	7	-0.5279037	1.37E-06	0.02508817	0.768	0.833
RPS20	7	-0.5298829	2.22E-38	4.05E-34	1	1
B2M	7	-0.5333179	5.52E-27	1.01E-22	0.99	1
DCN	7	-0.5339575	6.41E-17	1.17E-12	0.942	0.996
TPM1	7	-0.5371942	1.43E-12	2.60E-08	0.865	0.972
HCFC1R1	7	-0.5392048	4.90E-19	8.93E-15	0.841	0.976
SNAI2	7	-0.5413324	6.34E-11	1.16E-06	0.715	0.85
DAB2	7	-0.5455174	2.26E-18	4.12E-14	0.425	0.776
ZFP36L2	7	-0.5462593	8.28E-08	0.00151024	0.319	0.533
PDE4DIP	7	-0.5480339	1.87E-23	3.42E-19	0.242	0.683
SPTLC3	7	-0.5485475	2.57E-16	4.70E-12	0.203	0.557
RPS11	7	-0.5566394	3.76E-46	6.86E-42	1	1
COL11A1	7	-0.5596318	3.06E-12	5.59E-08	0.836	0.959
MIA	7	-0.5603439	3.31E-16	6.04E-12	0.739	0.927
PLPP1	7	-0.5646298	1.76E-17	3.21E-13	0.159	0.524
ISM1	7	-0.565385	6.39E-23	1.17E-18	0.053	0.463
SOX9	7	-0.5690934	3.24E-18	5.91E-14	0.498	0.833
ITM2C	7	-0.5699679	4.35E-18	7.95E-14	0.599	0.846
SPTSSB	7	-0.5700145	1.03E-15	1.88E-11	0	0.268
CHI3L1	7	-0.5714045	9.09E-10	1.66E-05	0.454	0.736
MT-ND3	7	-0.5722557	8.39E-30	1.53E-25	0.894	0.972
MT-ND2	7	-0.5735589	3.52E-24	6.43E-20	0.845	0.967
MFGE8	7	-0.5758752	8.57E-17	1.56E-12	0.517	0.797
THY1	7	-0.580819	2.25E-14	4.11E-10	0.85	0.976
TPD52L1	7	-0.5885129	6.19E-25	1.13E-20	0.237	0.691
NGFRAP1	7	-0.5905725	6.97E-25	1.27E-20	0.85	0.976
ISLR	7	-0.6034372	2.98E-22	5.43E-18	0.512	0.821
MT-CYB	7	-0.6035037	7.14E-25	1.30E-20	0.894	0.98
COL11A2	7	-0.6059886	1.33E-17	2.44E-13	0.126	0.488
WWP2	7	-0.6278268	2.11E-19	3.86E-15	0.43	0.776

MYLK	7	-0.6326812	3.14E-19	5.73E-15	0.193	0.573
SOD3	7	-0.6337505	1.41E-17	2.58E-13	0.541	0.878
MALAT1	7	-0.6357287	1.99E-33	3.63E-29	1	1
PAX1	7	-0.6390045	1.19E-19	2.18E-15	0.643	0.894
CYR61	7	-0.639161	1.78E-07	0.003256	0.614	0.752
COL9A3	7	-0.6425377	1.19E-10	2.18E-06	0.275	0.512
S100A10	7	-0.6434171	8.80E-27	1.61E-22	1	1
GREM1	7	-0.6619461	4.06E-09	7.41E-05	0.063	0.268
MEG3	7	-0.7039969	8.02E-15	1.46E-10	0.44	0.683
FTL	7	-0.7086002	1.10E-41	2.01E-37	1	1
NUPR1	7	-0.7225764	5.51E-16	1.01E-11	0.749	0.935
EIF3H	7	-0.7366416	9.07E-31	1.65E-26	0.749	0.947
HAPLN1	7	-0.7562568	5.17E-15	9.44E-11	0.522	0.789
NOVA1	7	-0.760258	1.29E-11	2.35E-07	0.377	0.614
IGFBP6	7	-0.7665718	1.38E-29	2.52E-25	0.647	0.919
EEF1A1	7	-0.8043419	4.62E-50	8.42E-46	1	1
CITED2	7	-0.8176871	1.24E-20	2.26E-16	0.266	0.646
ID2	7	-0.8497162	2.80E-19	5.12E-15	0.594	0.841
ID1	7	-0.856571	2.13E-18	3.89E-14	0.763	0.955
FGFBP2	7	-0.8583893	1.05E-15	1.92E-11	0.43	0.699
PLAC9	7	-0.8615236	4.12E-35	7.52E-31	0.734	0.976
FHL1	7	-0.8649119	3.75E-29	6.84E-25	0.261	0.732
TSC22D1	7	-0.8784208	5.86E-22	1.07E-17	0.56	0.882
OGN	7	-0.9052004	4.13E-20	7.54E-16	0.473	0.825
EEF2	7	-0.9283671	1.17E-37	2.13E-33	0.894	0.976
FBXO2	7	-0.9306345	1.05E-31	1.91E-27	0.309	0.772
S100B	7	-0.9400103	5.27E-28	9.62E-24	0.386	0.825
VIM	7	-0.9400161	1.09E-25	1.99E-21	1	1
NEAT1	7	-0.9490675	1.17E-40	2.13E-36	1	1
CLEC3A	7	-0.9802986	6.51E-23	1.19E-18	0.068	0.484
C2orf40	7	-0.9924035	1.53E-20	2.78E-16	0.618	0.85
CTGF	7	-1.1409723	7.65E-10	1.40E-05	0.604	0.797
ACAN	7	-1.2210077	2.25E-40	4.10E-36	0.705	0.967
S100A1	7	-1.3273355	1.00E-47	1.83E-43	0.787	0.976
ADIRF	7	-1.3657137	8.57E-38	1.56E-33	0.372	0.862
MGP	7	-1.3806844	5.32E-35	9.71E-31	0.923	1
CAPS	7	-1.4188485	1.27E-49	2.32E-45	0.353	0.923
C2orf82	7	-1.5195248	4.50E-31	8.21E-27	0.662	0.919
SLPI	7	-1.6363984	1.78E-41	3.25E-37	0.444	0.931

COL2A1	7	-1.7319334	1.95E-37	3.56E-33	0.425	0.907
MSMP	7	-1.9292835	2.11E-29	3.86E-25	0.169	0.683
**gene**	**cluster**	**avg_logFC**	**p_val**	**p_val_adj**	**pct.1**	**pct.2**
MT2A	8	2.14090465	5.30E-33	9.68E-29	1	0.97
SPP1	8	2.0048262	1.72E-46	3.13E-42	0.805	0.045
FN1	8	1.75653579	9.66E-37	1.76E-32	1	0.985
TGFBI	8	1.42944845	4.99E-33	9.11E-29	0.805	0.174
SOD2	8	1.20016016	2.74E-21	5.00E-17	0.963	0.617
CTHRC1	8	1.13897545	4.66E-27	8.50E-23	0.659	0.117
S100A2	8	1.10532605	3.96E-08	0.00072319	0.573	0.299
COL1A2	8	1.08145424	3.32E-11	6.05E-07	0.659	0.386
MT1G	8	1.04792749	5.32E-08	0.00097124	0.927	0.826
PRG4	8	1.04630302	3.45E-33	6.29E-29	0.537	0.015
IGFBP3	8	1.00615757	8.50E-15	1.55E-10	0.488	0.106
SERPINE2	8	0.96935943	1.09E-17	1.99E-13	1	0.996
ADAMTS6	8	0.95038739	9.72E-25	1.77E-20	0.585	0.091
AEBP1	8	0.94339451	3.09E-17	5.64E-13	0.805	0.428
VEGFA	8	0.85604002	9.19E-14	1.68E-09	0.793	0.42
MT1X	8	0.83608541	3.74E-10	6.82E-06	0.988	0.985
SLC25A37	8	0.82203366	6.08E-18	1.11E-13	0.878	0.504
MT1E	8	0.803218	1.28E-08	0.00023377	0.988	0.939
IL11	8	0.77128358	4.20E-13	7.66E-09	0.207	0.004
TMSB10	8	0.76186661	4.01E-17	7.31E-13	1	0.977
HMGA1	8	0.75446236	1.59E-07	0.0029014	0.402	0.159
TNFRSF12A	8	0.73097492	6.12E-09	0.00011176	0.537	0.269
BMP2	8	0.72781007	5.62E-26	1.02E-21	0.451	0.019
PTGS2	8	0.71116876	1.97E-21	3.60E-17	0.5	0.064
CTSL	8	0.70590246	1.59E-06	0.02902062	0.585	0.383
LTBP1	8	0.70220685	2.74E-31	4.99E-27	0.512	0.015
PPP3CA	8	0.70013905	2.65E-12	4.84E-08	0.915	0.625
ABI3BP	8	0.69626619	1.21E-10	2.21E-06	0.707	0.364
BAALC	8	0.69302145	3.06E-14	5.58E-10	0.707	0.318
CRLF1	8	0.69230578	2.02E-17	3.69E-13	0.61	0.163
TMSB4X	8	0.6751539	1.96E-08	0.00035708	0.915	0.777
MT1M	8	0.61879509	5.44E-09	9.93E-05	0.732	0.5
CTNNB1	8	0.61085708	4.76E-09	8.69E-05	0.61	0.311
BTG1	8	0.60639086	1.70E-11	3.11E-07	0.988	0.879
NRP2	8	0.59424229	6.62E-19	1.21E-14	0.537	0.098
WTAP	8	0.59074558	2.88E-09	5.26E-05	0.695	0.405

CCND1	8	0.58551652	3.94E-11	7.19E-07	0.402	0.102
SLC5A3	8	0.56582896	2.00E-09	3.65E-05	0.695	0.367
SLC20A1	8	0.56474831	3.20E-10	5.84E-06	0.402	0.114
TNC	8	0.56064006	3.49E-25	6.36E-21	0.476	0.03
S100A16	8	0.5554677	3.50E-11	6.39E-07	0.598	0.239
UPP1	8	0.5439601	6.49E-09	0.00011844	0.39	0.121
STK17A	8	0.54104057	2.64E-11	4.81E-07	0.415	0.106
TM4SF1	8	0.53719066	4.01E-07	0.00732175	0.549	0.292
MIF	8	0.53097094	1.66E-17	3.02E-13	1	1
CD44	8	0.52224472	6.61E-11	1.21E-06	0.854	0.47
COMP	8	0.50268642	5.23E-07	0.00953972	0.902	0.689
BZW1	8	0.50116951	4.17E-08	0.00076132	0.707	0.443
PHLDA1	8	0.49806158	1.87E-17	3.42E-13	0.305	0.011
LOX	8	0.49616943	4.22E-13	7.70E-09	0.634	0.231
SLC39A14	8	0.48788919	9.46E-11	1.73E-06	0.732	0.436
TGM2	8	0.4659303	9.53E-12	1.74E-07	0.415	0.098
C11orf96	8	0.45898824	8.49E-11	1.55E-06	0.341	0.068
WWTR1	8	0.44433398	2.29E-10	4.19E-06	0.671	0.311
FAP	8	0.43935067	1.72E-16	3.15E-12	0.561	0.133
TREM1	8	0.42956266	4.02E-18	7.34E-14	0.268	0
COL5A1	8	0.41545805	4.26E-07	0.00776462	0.573	0.314
DNER	8	0.41134513	3.22E-08	0.00058841	0.488	0.205
ERO1A	8	0.41069992	1.73E-06	0.03165783	0.622	0.386
LARP6	8	0.40646984	2.64E-06	0.04817188	0.707	0.447
SH3BGRL3	8	0.40402936	3.60E-08	0.00065775	1	0.992
NT5E	8	0.39270107	2.99E-07	0.00545699	0.524	0.254
SGIP1	8	0.38975459	1.11E-17	2.03E-13	0.293	0.008
DUSP6	8	0.38215086	9.14E-07	0.01668176	0.293	0.087
CCDC109B	8	0.38139535	2.02E-11	3.69E-07	0.549	0.182
PHLDA2	8	0.37870851	4.54E-08	0.00082783	0.329	0.091
PLAUR	8	0.37566393	2.26E-07	0.0041236	0.415	0.167
NGF	8	0.37482894	4.66E-12	8.50E-08	0.256	0.023
LINC00152	8	0.37389173	1.18E-06	0.02153161	0.512	0.254
RNF145	8	0.3735038	1.07E-07	0.00195561	0.598	0.303
SMOX	8	0.36921953	2.10E-10	3.83E-06	0.415	0.114
IER3	8	0.36664883	4.54E-07	0.008281	0.439	0.174
ITGB1	8	0.36545305	2.73E-06	0.04974186	0.817	0.614
EPS8	8	0.36485704	1.16E-10	2.11E-06	0.451	0.133
MMP3	8	0.36207323	2.51E-08	0.0004589	0.707	0.367

SMOC1	8	0.34687094	2.62E-06	0.0477682	0.463	0.212
C4orf3	8	0.33978961	6.71E-10	1.22E-05	0.988	0.97
TYMP	8	0.33531012	3.41E-11	6.23E-07	0.427	0.106
SEMA3A	8	0.32795248	1.80E-15	3.29E-11	0.366	0.042
STK38L	8	0.32644159	1.38E-09	2.52E-05	0.378	0.102
PDE4B	8	0.31153329	5.65E-11	1.03E-06	0.39	0.091
FNIP2	8	0.30963447	3.25E-08	0.00059335	0.378	0.117
KITLG	8	0.30738628	5.02E-11	9.16E-07	0.305	0.049
PODNL1	8	0.29486548	1.75E-06	0.03199105	0.573	0.288
F13A1	8	0.27217049	3.53E-07	0.00644744	0.378	0.129
FZD8	8	0.27069585	3.35E-08	0.00061157	0.415	0.136
RUNX1	8	0.2651344	1.46E-06	0.02667984	0.451	0.197
AC090498.1	8	0.26472129	1.36E-08	0.00024735	1	1
CLMP	8	0.26298946	8.09E-07	0.01475793	0.329	0.106
CDKN2B	8	0.2603741	3.00E-11	5.48E-07	0.22	0.015
PRRX2	8	0.25826301	2.04E-08	0.0003731	0.268	0.053
PPP1R14C	8	0.25826301	8.27E-08	0.00150821	0.256	0.053
GJA1	8	0.25459338	1.22E-06	0.02220494	0.366	0.133
KDM7A	8	0.25293912	3.82E-11	6.97E-07	0.293	0.042
CRYL1	8	-0.2513536	2.58E-06	0.04716086	0.049	0.303
RPL18A	8	-0.2520051	2.35E-13	4.29E-09	1	1
RPS5	8	-0.2563959	1.15E-10	2.10E-06	1	1
RPL23	8	-0.2565689	1.34E-08	0.00024479	1	0.996
SRP14	8	-0.2621492	5.77E-07	0.01052312	0.988	1
RPS19	8	-0.2639272	1.55E-12	2.83E-08	1	1
BTF3	8	-0.2641901	4.79E-08	0.00087343	1	1
TPT1	8	-0.2688804	2.94E-11	5.37E-07	1	1
TOMM7	8	-0.2702371	1.54E-09	2.81E-05	1	1
RPS9	8	-0.2742005	2.09E-13	3.82E-09	1	1
RPL14	8	-0.2764811	9.12E-11	1.66E-06	1	1
RPS12	8	-0.2770668	2.14E-13	3.90E-09	1	1
RPS23	8	-0.2807657	1.25E-14	2.28E-10	1	1
RARRES3	8	-0.2876821	3.40E-07	0.00620503	0	0.258
GPX4	8	-0.2902154	6.38E-07	0.01163985	0.988	0.992
RPL8	8	-0.2918459	3.06E-16	5.59E-12	1	1
RPS7	8	-0.2924122	1.16E-12	2.11E-08	1	1
RPL37	8	-0.2926984	4.85E-16	8.86E-12	1	1
COX7A2L	8	-0.3013899	3.50E-07	0.00638916	0.841	0.939
COMMD6	8	-0.3030852	3.41E-09	6.23E-05	0.988	1

RPL13	8	-0.3062595	2.16E-19	3.94E-15	1	1
RPL22	8	-0.3124198	1.39E-12	2.54E-08	1	1
UQCRB	8	-0.3149251	6.66E-07	0.01215624	0.976	0.989
RPS25	8	-0.3158783	6.65E-17	1.21E-12	1	1
RPS6	8	-0.3159356	2.26E-19	4.12E-15	1	1
PRUNE2	8	-0.3174125	1.92E-06	0.0350824	0.085	0.36
RPL19	8	-0.3183727	6.89E-21	1.26E-16	1	1
RPL21	8	-0.3247526	2.22E-18	4.04E-14	1	1
COX7C	8	-0.3258862	4.55E-11	8.31E-07	1	1
RPL13A	8	-0.3287674	2.25E-21	4.10E-17	1	1
CCDC102B	8	-0.3324292	2.45E-06	0.04463219	0.11	0.383
NDUFA11	8	-0.3326665	8.66E-08	0.00158009	0.89	0.97
PRDX6	8	-0.3369617	5.80E-08	0.0010576	0.78	0.909
RPS27	8	-0.3377268	1.67E-18	3.05E-14	1	1
CRTAP	8	-0.3399314	2.17E-07	0.00396802	0.793	0.909
RPS17	8	-0.3400225	3.90E-17	7.11E-13	1	1
CALM2	8	-0.3412828	4.47E-07	0.00815719	0.963	0.973
ST13	8	-0.3419501	1.74E-07	0.00317092	0.866	0.951
RPL23A	8	-0.3420657	3.85E-18	7.03E-14	1	1
HSP90AB1	8	-0.3457068	4.93E-08	0.00089957	0.951	0.981
TMEM98	8	-0.3487739	2.08E-07	0.00380021	0.232	0.545
RPL34	8	-0.3500223	3.94E-18	7.19E-14	1	1
UBC	8	-0.3513768	5.04E-08	0.00091979	0.817	0.936
TSPO	8	-0.3523611	6.07E-08	0.00110786	0.841	0.962
CCNI	8	-0.3558094	1.96E-08	0.00035685	1	0.992
C2orf40	8	-0.3590105	1.38E-06	0.02518553	0.963	1
RPL10A	8	-0.3611715	6.45E-16	1.18E-11	1	1
RPS13	8	-0.3644324	3.67E-17	6.70E-13	1	1
CLU	8	-0.365099	5.70E-07	0.01040087	0.988	1
SOD1	8	-0.369236	1.73E-08	0.00031504	0.866	0.973
SPCS2	8	-0.3700624	5.98E-08	0.0010918	0.61	0.83
RPL18	8	-0.3721546	3.39E-18	6.18E-14	1	1
TXNIP	8	-0.3729349	3.80E-07	0.00693464	0.012	0.277
PLXDC2	8	-0.373763	6.14E-07	0.01120598	0.549	0.739
KHDRBS3	8	-0.373763	3.67E-07	0.00669536	0.024	0.295
EDF1	8	-0.3781936	3.17E-11	5.78E-07	0.902	0.981
TMED9	8	-0.3813676	1.09E-06	0.01982271	0.561	0.795
BEX2	8	-0.3813676	1.45E-06	0.02639802	0.146	0.443
SNHG8	8	-0.3861408	7.94E-07	0.01449487	0.732	0.905

RPS3A	8	-0.3904186	5.26E-24	9.59E-20	1	1
LDHB	8	-0.3917627	1.02E-07	0.00185679	0.707	0.864
PSMA7	8	-0.392006	1.25E-07	0.00227913	0.829	0.894
SSR3	8	-0.3940904	9.80E-10	1.79E-05	0.902	0.973
CTSD	8	-0.3951654	1.71E-08	0.00031275	0.78	0.936
EID1	8	-0.39688	2.80E-09	5.10E-05	0.854	0.985
CRIP2	8	-0.3970359	5.06E-07	0.00923399	0.488	0.716
TUBB	8	-0.3983439	2.64E-06	0.04809499	0.622	0.856
SEC11A	8	-0.3986943	3.10E-09	5.65E-05	0.841	0.955
RPL9	8	-0.4006914	3.21E-22	5.85E-18	1	1
RCN1	8	-0.4030291	1.62E-06	0.02947349	0.512	0.705
RPS16	8	-0.4047896	5.64E-22	1.03E-17	1	1
ARL2BP	8	-0.4052283	2.34E-07	0.00427677	0.415	0.659
ATRNL1	8	-0.4066619	5.20E-07	0.00949542	0.171	0.466
MORF4L2	8	-0.4066733	1.14E-07	0.00207427	0.695	0.852
PSAP	8	-0.4086656	3.45E-08	0.00062949	0.793	0.924
RPS27A	8	-0.4090684	4.12E-25	7.51E-21	1	1
MT-ND3	8	-0.4095658	3.84E-07	0.00700016	0.793	0.962
LAPTM4A	8	-0.4100274	3.45E-08	0.00062964	0.89	0.977
RPS8	8	-0.4109458	7.09E-25	1.29E-20	1	1
ANXA5	8	-0.4118299	5.73E-07	0.01044738	0.634	0.837
CST3	8	-0.4221846	3.35E-10	6.11E-06	0.988	1
SLC14A1	8	-0.4230811	2.03E-07	0.00369654	0.268	0.557
ATRAID	8	-0.4238142	2.58E-09	4.70E-05	0.78	0.947
GAS6	8	-0.4277598	6.53E-08	0.00119143	0.146	0.473
NPC2	8	-0.4407042	2.60E-11	4.75E-07	0.976	0.989
COL11A2	8	-0.4446427	3.03E-09	5.52E-05	0.634	0.864
RPL30	8	-0.4495213	1.10E-23	2.02E-19	1	1
CREG1	8	-0.4516181	4.00E-09	7.30E-05	0.354	0.686
GSTP1	8	-0.4539257	6.70E-10	1.22E-05	0.915	0.992
PEBP1	8	-0.4569975	1.07E-09	1.95E-05	0.683	0.898
PRDX4	8	-0.4581592	2.74E-10	5.00E-06	0.817	0.966
C6orf48	8	-0.4602512	4.23E-09	7.71E-05	0.902	0.97
HSP90AA1	8	-0.46233	7.38E-08	0.00134741	0.5	0.769
TMED10	8	-0.4635036	6.50E-11	1.19E-06	0.841	0.966
APOD	8	-0.4635885	1.47E-07	0.00269026	0.098	0.409
FCGRT	8	-0.464342	3.15E-09	5.75E-05	0.427	0.739
CHID1	8	-0.4724937	5.82E-09	0.00010621	0.415	0.705
EEF2	8	-0.474226	3.49E-08	0.00063767	0.988	1

RPL7	8	-0.4777712	1.50E-22	2.74E-18	1	1
MSMO1	8	-0.4780783	2.01E-07	0.00366161	0.244	0.58
COL9A2	8	-0.47828	7.29E-07	0.0132935	0.488	0.697
EIF3L	8	-0.4785737	5.03E-12	9.18E-08	0.744	0.951
RPS11	8	-0.4811673	1.11E-19	2.02E-15	1	1
RHOBTB3	8	-0.4825104	8.80E-08	0.00160653	0.524	0.765
ITM2B	8	-0.4886814	1.85E-11	3.38E-07	0.988	0.992
PLAC9	8	-0.4900426	2.34E-11	4.27E-07	0.878	1
SPON2	8	-0.4919094	2.76E-09	5.03E-05	0.122	0.492
CCPG1	8	-0.4940185	2.29E-07	0.00418741	0.402	0.648
COL2A1	8	-0.4957399	2.95E-09	5.38E-05	0.951	1
ISLR	8	-0.4963219	3.10E-09	5.65E-05	0.415	0.739
NUCB2	8	-0.503153	2.31E-08	0.00042157	0.549	0.792
RPL4	8	-0.505718	8.69E-20	1.59E-15	1	1
NAP1L1	8	-0.5112255	3.03E-14	5.52E-10	0.854	0.962
DDR2	8	-0.5222453	6.92E-10	1.26E-05	0.402	0.727
MT-ND2	8	-0.5230603	7.88E-07	0.01438469	0.756	0.886
CP	8	-0.5235897	2.33E-08	0.00042503	0.378	0.693
ACAN	8	-0.5323327	4.93E-16	8.99E-12	0.976	1
INSIG1	8	-0.5329541	2.10E-07	0.00382882	0.354	0.674
MIA	8	-0.5351212	5.41E-14	9.87E-10	0.988	0.996
PDIA6	8	-0.5425503	2.90E-10	5.29E-06	0.756	0.902
MYLK	8	-0.5589916	4.48E-07	0.00817326	0.268	0.545
RPLP0	8	-0.5607529	2.00E-29	3.65E-25	1	1
ITM2A	8	-0.5653712	5.93E-09	0.00010816	0.439	0.727
CPE	8	-0.5681034	5.25E-09	9.58E-05	0.366	0.697
HLA-C	8	-0.5687684	4.44E-12	8.11E-08	0.829	0.962
CITED2	8	-0.5773653	3.25E-09	5.93E-05	0.317	0.648
NGFRAP1	8	-0.5818972	1.53E-17	2.78E-13	0.841	0.985
IGFBP7	8	-0.5938295	2.05E-11	3.74E-07	0.854	0.955
EEF1A1	8	-0.5966622	2.05E-28	3.74E-24	1	1
ID3	8	-0.5974656	1.29E-08	0.00023619	0.622	0.879
ID4	8	-0.6059729	9.84E-10	1.79E-05	0.598	0.841
FTL	8	-0.6080659	1.18E-23	2.15E-19	1	1
FHL1	8	-0.6107944	1.65E-11	3.00E-07	0.207	0.606
PLPP1	8	-0.6190147	2.32E-11	4.24E-07	0.159	0.557
HLA-B	8	-0.6396585	3.25E-13	5.92E-09	0.561	0.852
DDIT4	8	-0.6428531	2.08E-06	0.03789463	0.329	0.606
RPS20	8	-0.6432799	2.20E-29	4.02E-25	1	1

NUPR1	8	-0.6445331	1.83E-11	3.34E-07	0.963	0.996
MFGE8	8	-0.6489629	7.33E-12	1.34E-07	0.732	0.913
ID2	8	-0.6588007	5.03E-09	9.18E-05	0.561	0.792
PAX1	8	-0.6734777	3.13E-13	5.72E-09	0.561	0.871
ZFAS1	8	-0.6773151	9.31E-19	1.70E-14	0.939	0.996
PKM	8	-0.681905	7.03E-15	1.28E-10	0.915	0.958
HLA-A	8	-0.6951222	6.65E-14	1.21E-09	0.78	0.924
B2M	8	-0.6972409	4.01E-20	7.32E-16	0.963	1
ID1	8	-0.7098716	3.66E-07	0.00667705	0.732	0.864
CHRDL2	8	-0.7142684	2.31E-10	4.22E-06	0.037	0.413
EIF3H	8	-0.7257871	6.84E-24	1.25E-19	0.951	0.992
TMEM59	8	-0.7307678	4.56E-19	8.32E-15	0.915	0.985
SDC2	8	-0.7390773	2.03E-19	3.70E-15	0.817	0.981
SNAI2	8	-0.7859906	6.72E-10	1.23E-05	0.488	0.765
CD9	8	-0.805106	3.89E-13	7.10E-09	0.293	0.678
DCN	8	-0.8082969	7.00E-23	1.28E-18	1	1
FBXO2	8	-0.8707371	1.77E-23	3.23E-19	0.646	0.985
IGFBP6	8	-0.8788818	1.68E-15	3.07E-11	0.695	0.928
MGP	8	-0.8842117	1.34E-15	2.45E-11	0.988	1
ADIRF	8	-0.9371631	9.25E-17	1.69E-12	0.354	0.807
FTH1	8	-0.9698113	5.67E-31	1.04E-26	1	1
CAPS	8	-0.9931827	5.09E-21	9.29E-17	0.683	0.977
COL9A3	8	-1.0212242	1.68E-20	3.07E-16	0.634	0.951
C2orf82	8	-1.1068023	8.87E-26	1.62E-21	0.927	1
LECT1	8	-1.1132151	3.15E-24	5.75E-20	0.841	0.985
OGN	8	-1.1484734	1.12E-23	2.03E-19	0.805	0.996
CLEC3A	8	-1.2313356	2.21E-23	4.04E-19	0.537	0.943
CRISPLD1	8	-1.3869398	3.71E-24	6.78E-20	0.061	0.742
FRZB	8	-1.4765016	7.95E-20	1.45E-15	0.207	0.784
SLPI	8	-1.600589	4.60E-18	8.39E-14	0.854	1
SPTSSB	8	-1.7198785	4.34E-27	7.93E-23	0.049	0.784
**gene**	**cluster**	**avg_logFC**	**p_val**	**p_val_adj**	**pct.1**	**pct.2**
COL1A1	9	1.98487474	6.94E-18	1.27E-13	0.682	0.108
TGFBI	9	1.95399427	2.34E-29	4.28E-25	1	0.541
MT2A	9	1.79699428	2.82E-18	5.14E-14	1	0.955
PRG4	9	1.56820125	5.22E-14	9.53E-10	0.761	0.297
CTHRC1	9	1.52363759	4.37E-24	7.98E-20	0.886	0.306
SPP1	9	1.4508406	1.31E-16	2.38E-12	0.557	0.036
IL11	9	1.12001244	1.07E-09	1.96E-05	0.352	0.027

POSTN	9	1.1094336	5.65E-10	1.03E-05	0.386	0.036
TNC	9	1.00244296	1.26E-19	2.30E-15	0.739	0.135
CYTL1	9	0.90379872	1.30E-13	2.38E-09	0.784	0.279
CTSL	9	0.84837953	2.95E-08	0.00053886	0.886	0.757
MT1X	9	0.83641406	7.49E-08	0.00136754	0.989	0.982
IGFBP3	9	0.8211677	6.45E-09	0.00011766	0.773	0.396
LTBP1	9	0.76517182	1.37E-19	2.51E-15	0.716	0.108
COL1A2	9	0.76466887	2.54E-06	0.04628643	0.864	0.775
SERPINE2	9	0.74880671	5.71E-12	1.04E-07	1	1
PHLDA1	9	0.70699319	1.71E-08	0.00031261	0.455	0.117
ENPP1	9	0.65075503	1.40E-06	0.02555158	0.693	0.387
FAP	9	0.60964546	6.07E-12	1.11E-07	0.83	0.378
VMP1	9	0.59955865	1.48E-09	2.69E-05	0.83	0.541
TFPI2	9	0.59545814	8.30E-07	0.01514951	0.318	0.054
SMOC1	9	0.55913373	3.45E-08	0.00062947	0.841	0.613
LINC00152	9	0.55100638	3.56E-07	0.00650377	0.818	0.595
GNG11	9	0.54862487	1.32E-06	0.02401254	0.693	0.414
PLAUR	9	0.54002003	2.63E-08	0.00048074	0.705	0.342
GJA1	9	0.53651478	1.37E-07	0.00250173	0.591	0.243
MIF	9	0.5060195	9.97E-17	1.82E-12	1	1
CLEC11A	9	0.49634496	5.53E-07	0.01009447	0.909	0.784
TMSB4X	9	0.47317181	2.28E-07	0.0041528	0.977	0.874
PTGES	9	0.47214406	2.68E-11	4.89E-07	0.443	0.045
FBLN1	9	0.46318012	5.83E-10	1.06E-05	0.523	0.126
COL6A3	9	0.46147193	1.22E-06	0.02231186	0.864	0.577
FN1	9	0.45419081	1.52E-09	2.78E-05	1	0.991
TMSB10	9	0.44676533	2.26E-08	0.00041292	1	0.991
SOX4	9	0.44511538	2.38E-08	0.00043496	0.364	0.054
MIR4435-2HG	9	0.43852982	2.55E-06	0.04661852	0.761	0.468
CD44	9	0.42771646	3.98E-07	0.00726156	0.92	0.685
BMP2	9	0.4263494	1.43E-06	0.02612129	0.216	0.009
SPOCK1	9	0.42000946	1.69E-10	3.08E-06	0.477	0.081
ADAMTS6	9	0.41585813	9.76E-09	0.00017809	0.466	0.117
CRLF1	9	0.39924747	1.41E-06	0.02581144	0.659	0.351
TMEM59L	9	0.39727314	2.02E-08	0.00036885	0.352	0.045
TREM1	9	0.37350692	9.26E-10	1.69E-05	0.318	0.009
MMP14	9	0.36744743	1.82E-06	0.03324805	0.568	0.261
EVI2A	9	0.36140512	3.66E-07	0.00666977	0.295	0.036
JAG1	9	0.35355424	1.85E-06	0.03384495	0.386	0.108

SLC38A5	9	0.34905773	3.93E-07	0.00717758	0.386	0.09
TNFSF11	9	0.34200825	2.65E-08	0.00048312	0.273	0.009
SGIP1	9	0.3162765	4.09E-08	0.00074677	0.307	0.027
SRPX2	9	0.29673191	2.02E-06	0.03686423	0.568	0.243
LAMA4	9	0.28020261	2.06E-06	0.03757081	0.341	0.072
PLCD3	9	-0.2584295	2.39E-06	0.04363635	0.023	0.27
RPL37	9	-0.2606483	7.51E-07	0.01369636	1	1
RPLP2	9	-0.2627266	6.05E-07	0.01104672	1	1
RPL13A	9	-0.2646556	8.36E-07	0.01524681	1	1
KHDRBS3	9	-0.2719432	1.38E-06	0.02521356	0.023	0.279
CSTB	9	-0.291566	2.38E-06	0.04338322	0.989	0.991
RPL10A	9	-0.2956243	1.85E-06	0.03375941	1	1
RPS17	9	-0.2982527	2.42E-07	0.0044179	1	1
RP11-71N10.1	9	-0.3030508	1.05E-06	0.01918506	0.011	0.261
CD8B	9	-0.3108015	1.96E-06	0.03580328	0.159	0.477
ARFGAP3	9	-0.3119979	2.54E-06	0.04633493	0.227	0.559
ITGB5	9	-0.317853	1.74E-06	0.03183668	0.159	0.486
RPS25	9	-0.319942	7.42E-07	0.01354178	1	1
TENM3	9	-0.3211919	1.70E-07	0.00310755	0.045	0.351
KAZALD1	9	-0.3219127	1.44E-06	0.02633302	0.17	0.486
RPL23A	9	-0.3224779	1.42E-07	0.00259511	1	1
TMED10	9	-0.3271028	7.22E-07	0.01318037	0.955	0.982
SEC11C	9	-0.3344696	1.14E-06	0.02079805	0.114	0.432
RHOA	9	-0.3400223	8.73E-07	0.01593633	0.943	0.991
TMEM59	9	-0.3451962	5.60E-07	0.0102225	0.989	1
HLA-A	9	-0.3460962	2.54E-06	0.04629875	0.989	0.991
SRSF8	9	-0.3486865	1.53E-06	0.02787112	0.216	0.55
TXNIP	9	-0.3494013	1.35E-06	0.0246549	0.023	0.279
RPLP1	9	-0.3555031	1.30E-10	2.38E-06	1	1
ISM1	9	-0.35675	2.66E-06	0.04855941	0.08	0.36
RPS27	9	-0.3627618	1.18E-07	0.00215901	1	1
RPL4	9	-0.3645747	1.38E-07	0.00251496	1	1
C1QTNF8	9	-0.3711838	1.88E-06	0.03431337	0.114	0.414
RPL7	9	-0.3865184	6.93E-09	0.00012646	1	1
RPL9	9	-0.3895603	1.97E-10	3.60E-06	1	1
AFF3	9	-0.3908442	2.76E-07	0.00502903	0.08	0.396
SMIM5	9	-0.3919609	3.95E-09	7.21E-05	0.023	0.369
COMMD6	9	-0.3929389	1.53E-07	0.00279435	0.943	0.973
A2M	9	-0.3971641	3.45E-07	0.00630375	0.011	0.279

ARL2BP	9	-0.4018061	1.07E-06	0.01958525	0.511	0.784
JTB	9	-0.4059949	1.98E-07	0.00360472	0.648	0.883
AHNAK2	9	-0.4101815	8.58E-07	0.01566399	0.125	0.441
OSBPL9	9	-0.4144338	1.55E-06	0.02821037	0.307	0.604
LPP	9	-0.4210372	1.32E-06	0.02416763	0.25	0.55
HLA-C	9	-0.4214887	1.13E-06	0.02066762	0.966	0.991
ATRAID	9	-0.4244756	4.30E-08	0.00078409	0.943	0.955
RPS8	9	-0.4295999	6.97E-13	1.27E-08	1	1
COL14A1	9	-0.433998	2.32E-07	0.00423061	0.068	0.378
TIMP4	9	-0.434567	8.56E-09	0.00015626	0.091	0.459
ATRNL1	9	-0.4399004	7.67E-09	0.00014003	0.091	0.459
RPS11	9	-0.4413837	8.02E-12	1.46E-07	0.989	1
SCIN	9	-0.4417224	1.51E-06	0.02762172	0.182	0.486
ZBTB20	9	-0.4511976	1.27E-07	0.00231697	0.148	0.486
SLC14A1	9	-0.4516514	1.23E-07	0.00223909	0.17	0.523
SLC40A1	9	-0.4609538	2.60E-06	0.04752438	0.125	0.414
CXXC5	9	-0.4644442	2.26E-07	0.00412902	0.477	0.73
RPLP0	9	-0.4684165	6.97E-14	1.27E-09	1	1
TBL1XR1	9	-0.4746526	1.16E-06	0.02120354	0.455	0.721
RPL30	9	-0.4766668	6.31E-13	1.15E-08	1	1
DNM3OS	9	-0.4775931	2.91E-07	0.00530431	0.466	0.73
GPC1	9	-0.4786534	6.52E-11	1.19E-06	0.227	0.685
C1S	9	-0.4809323	5.53E-07	0.01008899	0.636	0.892
ARL6IP5	9	-0.4869293	2.04E-09	3.73E-05	0.727	0.901
CCDC102B	9	-0.4874805	4.16E-10	7.59E-06	0.045	0.441
SPINT2	9	-0.4892385	4.82E-07	0.00879466	0.295	0.613
MT-ND3	9	-0.4901956	4.79E-08	0.00087492	0.83	0.91
PNRC1	9	-0.4908068	8.13E-07	0.0148393	0.648	0.919
TSPAN4	9	-0.5019618	4.28E-09	7.80E-05	0.807	0.901
B2M	9	-0.5113682	1.72E-11	3.13E-07	1	1
TPT1	9	-0.5128256	1.09E-11	1.98E-07	1	1
PLXDC2	9	-0.5132533	1.00E-07	0.00183137	0.489	0.739
HERPUD1	9	-0.5285119	3.45E-07	0.00629234	0.636	0.829
CHI3L1	9	-0.5449192	1.11E-07	0.00201996	0.511	0.892
EEF2	9	-0.5514678	8.08E-10	1.47E-05	0.955	0.982
MYLK	9	-0.5649977	4.09E-08	0.00074667	0.239	0.604
SLC38A1	9	-0.5677838	4.43E-12	8.08E-08	0.148	0.622
C2orf40	9	-0.5740731	1.72E-07	0.00314119	0.83	0.955
COL9A2	9	-0.581033	1.21E-06	0.02215533	0.352	0.676

CRIP2	9	-0.5810981	6.09E-08	0.00111204	0.398	0.73
SDC2	9	-0.5840865	1.58E-11	2.89E-07	0.852	0.991
FXYD6	9	-0.5873249	2.71E-12	4.95E-08	0.773	0.937
MALAT1	9	-0.5911339	2.04E-08	0.00037187	1	1
NOV	9	-0.5964993	8.34E-07	0.01521147	0.057	0.342
FMOD	9	-0.6008292	1.24E-08	0.00022628	0.864	0.946
FTL	9	-0.6008527	3.47E-13	6.34E-09	1	1
NINJ1	9	-0.6065938	3.24E-12	5.91E-08	0.216	0.712
SERPINA5	9	-0.6066343	1.65E-06	0.03007842	0.693	0.865
PDE4DIP	9	-0.617463	2.44E-11	4.45E-07	0.33	0.73
RPS20	9	-0.6190717	1.03E-15	1.88E-11	0.989	1
SPTSSB	9	-0.6215045	1.04E-07	0.00189409	0.011	0.297
NGFRAP1	9	-0.6231205	1.43E-15	2.61E-11	0.886	0.991
CST3	9	-0.6316183	1.29E-14	2.36E-10	0.977	1
MATN4	9	-0.6414422	4.54E-07	0.0082768	0.023	0.297
EEF1A1	9	-0.6423597	3.10E-17	5.66E-13	1	1
ITM2A	9	-0.6473322	4.16E-09	7.60E-05	0.545	0.838
FTH1	9	-0.648118	2.81E-16	5.13E-12	1	1
ISLR	9	-0.6482782	5.54E-12	1.01E-07	0.705	0.946
PSAP	9	-0.6660036	2.03E-12	3.71E-08	0.818	0.982
PAX1	9	-0.6874068	2.83E-11	5.16E-07	0.625	0.883
MT-ND2	9	-0.6923278	2.69E-09	4.91E-05	0.784	0.901
CP	9	-0.7117489	7.74E-07	0.01411899	0.398	0.73
CLU	9	-0.7130899	9.54E-09	0.000174	0.955	0.991
ZFAS1	9	-0.72305	1.19E-08	0.00021765	0.795	0.91
APOD	9	-0.7271568	2.74E-09	5.00E-05	0.136	0.514
CRISPLD1	9	-0.7523056	7.86E-07	0.01433362	0.148	0.45
ID4	9	-0.7707362	7.96E-08	0.00145282	0.511	0.757
CPE	9	-0.7712832	2.52E-12	4.60E-08	0.523	0.865
MIA	9	-0.78026	1.75E-11	3.20E-07	0.83	0.973
MOXD1	9	-0.7874757	9.61E-14	1.75E-09	0.136	0.64
SOD3	9	-0.804061	8.19E-11	1.49E-06	0.739	0.982
ID2	9	-0.8098861	2.53E-07	0.004614	0.557	0.775
DCN	9	-0.8182076	4.44E-14	8.09E-10	1	1
EIF3H	9	-0.8508544	1.01E-16	1.84E-12	0.83	0.982
NUPR1	9	-0.8707667	1.64E-09	2.99E-05	0.807	0.937
ADIRF	9	-0.8943092	2.06E-11	3.76E-07	0.307	0.766
CRYAB	9	-0.9240317	5.17E-12	9.43E-08	0.852	0.955
MFGE8	9	-0.9260696	3.43E-15	6.25E-11	0.807	0.964

LECT1	9	-0.9404783	4.24E-10	7.73E-06	0.625	0.892
FGFBP2	9	-0.9589062	4.35E-09	7.94E-05	0.58	0.874
PLPP1	9	-0.9649237	1.70E-14	3.10E-10	0.511	0.865
COL9A3	9	-1.0138506	1.32E-10	2.40E-06	0.42	0.802
CITED2	9	-1.0291615	5.12E-08	0.00093345	0.443	0.694
COL11A2	9	-1.029599	7.99E-14	1.46E-09	0.261	0.721
IGFBP6	9	-1.0487405	4.31E-19	7.87E-15	0.636	0.973
S100A1	9	-1.0596004	1.86E-16	3.39E-12	0.83	0.964
MGP	9	-1.0901704	2.33E-13	4.26E-09	0.977	1
PLAC9	9	-1.0911871	2.27E-18	4.13E-14	0.75	0.964
FRZB	9	-1.160896	1.97E-10	3.60E-06	0.159	0.577
TSC22D1	9	-1.1928155	4.93E-12	8.99E-08	0.432	0.784
OGN	9	-1.2046262	1.20E-12	2.18E-08	0.636	0.937
ACAN	9	-1.2279623	6.33E-17	1.15E-12	0.83	0.964
FBXO2	9	-1.2482882	1.08E-22	1.96E-18	0.432	0.937
CLEC3A	9	-1.2867305	7.81E-13	1.42E-08	0.102	0.586
FHL1	9	-1.3438816	3.60E-20	6.57E-16	0.307	0.856
COL2A1	9	-1.4173024	1.96E-16	3.58E-12	0.511	0.946
C2orf82	9	-1.429542	1.65E-16	3.01E-12	0.739	0.928
CAPS	9	-1.6254376	3.77E-24	6.89E-20	0.443	0.946
SLPI	9	-1.6937446	4.22E-18	7.69E-14	0.648	0.964
**gene**	**cluster**	**avg_logFC**	**p_val**	**p_val_adj**	**pct.1**	**pct.2**
SPP1	10	2.74826017	2.77E-20	5.06E-16	0.876	0.125
PRG4	10	2.11408356	3.16E-15	5.76E-11	0.703	0.036
MT2A	10	2.11088645	8.26E-23	1.51E-18	1	0.982
SOD2	10	1.49006915	3.82E-18	6.97E-14	1	0.804
HMGA1	10	1.44781108	3.80E-19	6.94E-15	0.862	0.196
UPP1	10	1.41526068	5.56E-21	1.01E-16	0.883	0.161
CTSL	10	1.38441171	1.28E-15	2.33E-11	0.841	0.232
IGFBP3	10	1.36720338	5.74E-12	1.05E-07	0.641	0.107
COMP	10	1.35891967	1.65E-16	3.01E-12	0.945	0.643
PTGS2	10	1.29262374	1.05E-17	1.91E-13	0.759	0.036
CD55	10	1.19586118	5.01E-19	9.14E-15	0.966	0.446
C11orf96	10	1.18385085	4.63E-13	8.44E-09	0.655	0.071
VEGFA	10	1.12260989	2.42E-14	4.41E-10	0.862	0.339
MT1X	10	1.11771814	5.78E-08	0.00105503	1	1
WTAP	10	1.11620945	2.89E-14	5.27E-10	0.972	0.804
NRP2	10	1.07799899	1.71E-13	3.12E-09	0.697	0.089
S100A2	10	1.07033531	4.75E-12	8.67E-08	0.779	0.286

SERPINE2	10	1.05752477	2.82E-10	5.14E-06	1	1
CTNNB1	10	1.01853309	3.39E-15	6.18E-11	0.959	0.75
MRPS6	10	0.98963213	7.57E-11	1.38E-06	0.966	0.893
STK17A	10	0.96554056	1.02E-14	1.86E-10	0.745	0.107
DDIT4L	10	0.91919838	9.01E-10	1.64E-05	0.862	0.482
PPP3CA	10	0.90874631	2.90E-15	5.28E-11	0.993	0.929
ERO1A	10	0.89753583	1.16E-13	2.12E-09	0.848	0.304
VAPA	10	0.88454864	2.23E-18	4.07E-14	0.993	0.875
TIMP3	10	0.88420564	3.28E-08	0.00059817	0.738	0.411
TNFRSF12A	10	0.87043748	3.98E-10	7.27E-06	0.634	0.143
CCDC85B	10	0.86489703	7.28E-10	1.33E-05	0.931	0.732
TM4SF1	10	0.85893083	1.07E-09	1.96E-05	0.731	0.268
FN1	10	0.85790189	3.39E-12	6.19E-08	1	1
DNAJB9	10	0.85461253	7.64E-13	1.39E-08	0.869	0.429
BZW1	10	0.83682502	1.14E-12	2.09E-08	0.89	0.393
SMOX	10	0.83560414	4.09E-09	7.47E-05	0.641	0.214
SNHG12	10	0.82963618	2.23E-13	4.06E-09	0.8	0.25
ARF6	10	0.82639118	7.15E-14	1.30E-09	0.89	0.446
MIF	10	0.82424635	1.09E-21	1.99E-17	1	1
RABGGTB	10	0.81220654	8.96E-16	1.63E-11	0.924	0.571
GOLT1B	10	0.81131328	3.77E-11	6.88E-07	0.641	0.143
SELK	10	0.80818429	5.05E-14	9.21E-10	0.972	0.661
PPP1CB	10	0.78986756	1.33E-19	2.42E-15	1	0.964
ISG20	10	0.78791385	4.44E-12	8.10E-08	0.759	0.286
NDRG1	10	0.78675633	3.25E-10	5.93E-06	0.745	0.357
CSGALNACT2	10	0.77138455	2.90E-10	5.29E-06	0.559	0.071
GSTO1	10	0.76380092	1.76E-13	3.22E-09	0.924	0.536
DNER	10	0.76016108	1.40E-09	2.55E-05	0.717	0.268
BHLHE40	10	0.75758546	9.96E-08	0.00181657	0.731	0.393
BMP2	10	0.75176987	2.06E-09	3.75E-05	0.476	0.018
BAALC	10	0.74700335	5.86E-07	0.01069239	0.662	0.339
CTHRC1	10	0.74357803	1.96E-09	3.58E-05	0.455	0
PMP22	10	0.72971185	3.98E-13	7.26E-09	0.959	0.75
EIF4EBP1	10	0.72411761	3.45E-08	0.00062974	0.69	0.304
PPP1R15A	10	0.7177751	1.79E-07	0.00325924	0.655	0.268
RELB	10	0.71449717	1.40E-09	2.55E-05	0.559	0.089
ARPP19	10	0.68725502	8.03E-10	1.46E-05	0.738	0.304
DDIT3	10	0.68496331	1.87E-09	3.42E-05	0.752	0.25
TMSB10	10	0.67896593	3.42E-10	6.24E-06	0.993	1

RNF145	10	0.67436057	1.06E-07	0.00194256	0.6	0.214
ENO2	10	0.66880865	2.51E-09	4.58E-05	0.69	0.286
SLC25A37	10	0.66292205	1.36E-10	2.47E-06	0.966	0.661
CCND1	10	0.66108158	8.20E-09	0.00014963	0.586	0.143
SLC2A1	10	0.65178625	6.84E-08	0.00124776	0.538	0.143
EPS8	10	0.65136687	1.74E-10	3.18E-06	0.572	0.071
PPFIBP1	10	0.64481063	9.82E-08	0.00179109	0.655	0.321
EIF1	10	0.64420876	3.31E-19	6.04E-15	1	1
SLC7A2	10	0.64096143	1.39E-08	0.00025401	0.483	0.054
XBP1	10	0.63807739	1.09E-07	0.00198454	0.745	0.446
HLA-DRB1	10	0.62101459	1.51E-08	0.00027639	0.572	0.143
GRB10	10	0.61869479	1.43E-08	0.0002614	0.441	0.018
DNAJB1	10	0.6137556	1.18E-06	0.02154589	0.428	0.071
RNF114	10	0.60553783	1.19E-08	0.00021663	0.745	0.375
ZNF593	10	0.60342589	3.97E-09	7.25E-05	0.697	0.25
HMGA2	10	0.60299608	2.83E-11	5.16E-07	0.524	0
COX17	10	0.60248601	5.11E-10	9.32E-06	0.89	0.554
SLC3A2	10	0.60074852	6.67E-08	0.00121645	0.607	0.196
SGIP1	10	0.59764673	3.44E-08	0.00062759	0.448	0.036
ENO1	10	0.59435961	2.11E-07	0.00385023	0.993	0.964
PHLDA1	10	0.59281534	5.23E-07	0.00954034	0.372	0.018
ZNF385D	10	0.59259282	7.01E-09	0.00012782	0.834	0.482
AK2	10	0.59122515	3.20E-08	0.00058302	0.807	0.446
TGM2	10	0.57726661	2.95E-08	0.00053837	0.593	0.179
EIF1B	10	0.5767425	1.47E-09	2.68E-05	0.89	0.554
ATP6V1G1	10	0.57397274	6.08E-11	1.11E-06	1	0.946
PPP1R14C	10	0.56412404	2.23E-08	0.00040736	0.455	0.036
PTGES3	10	0.56390443	1.41E-09	2.57E-05	0.979	0.964
VDAC1	10	0.56120603	6.56E-08	0.00119702	0.952	0.661
TREM1	10	0.55852627	3.02E-08	0.00055085	0.428	0.018
NPR3	10	0.55269535	5.80E-07	0.01057445	0.345	0
FAM162A	10	0.55112917	2.05E-07	0.00373794	0.938	0.661
VIMP	10	0.54979259	4.08E-08	0.00074374	0.903	0.589
ANXA5	10	0.54700196	7.57E-09	0.00013821	0.938	0.679
TFPI	10	0.54683023	4.35E-08	0.00079441	0.421	0.018
WDR45B	10	0.54065737	3.99E-07	0.00728572	0.524	0.143
PLAUR	10	0.54027283	8.80E-07	0.01606336	0.462	0.089
NBPF14	10	0.53825466	9.21E-08	0.00167996	0.503	0.107
SVIP	10	0.53796754	5.78E-07	0.0105547	0.641	0.268

GADD45GIP1	10	0.53699644	1.64E-08	0.00029927	0.862	0.5
CISD2	10	0.53451303	1.10E-08	0.00020009	0.676	0.268
TMEM38B	10	0.53335238	3.89E-08	0.00071026	0.745	0.393
TRMT112	10	0.52261789	3.03E-08	0.00055213	0.966	0.804
LRRFIP1	10	0.5191044	1.53E-06	0.02789329	0.593	0.25
EIF4A2	10	0.51478886	1.88E-12	3.43E-08	1	1
TVP23B	10	0.51035202	1.80E-07	0.00327954	0.572	0.179
CDC37L1	10	0.50852413	2.16E-06	0.03934515	0.586	0.25
EVI2A	10	0.50273491	6.30E-08	0.0011499	0.414	0.018
CD44	10	0.49862812	1.68E-06	0.0306428	0.862	0.607
PFKP	10	0.49463154	7.28E-08	0.0013281	0.538	0.107
PPP1R2	10	0.48972055	1.47E-07	0.00268371	0.621	0.232
NDUFB8	10	0.48210055	1.47E-06	0.02687554	0.897	0.661
GPRC5A	10	0.46824873	4.79E-07	0.00874318	0.421	0.054
BTG3	10	0.46794284	5.08E-07	0.00927559	0.552	0.179
EIF5	10	0.46770213	8.73E-08	0.00159304	0.814	0.482
DDX21	10	0.46770213	5.50E-08	0.00100296	0.483	0.071
GPX1	10	0.46606748	1.08E-06	0.01974146	0.917	0.75
FAP	10	0.46463849	1.46E-07	0.00266703	0.421	0.036
SAP18	10	0.46454411	2.50E-07	0.00456198	0.917	0.643
BNIP3	10	0.45989874	5.99E-08	0.00109341	0.986	1
SH3BGRL3	10	0.45368801	4.71E-08	0.0008598	0.986	0.964
SNRPG	10	0.44652684	2.00E-06	0.03653731	0.848	0.571
NXT1	10	0.44638471	2.14E-06	0.03896792	0.497	0.143
RNF126	10	0.44335118	7.33E-07	0.01338391	0.414	0.054
SQSTM1	10	0.44242711	8.31E-07	0.0151688	0.972	0.893
SPHK1	10	0.43928868	1.44E-06	0.02628959	0.352	0.018
COX7A2	10	0.43287772	1.81E-09	3.31E-05	0.979	0.964
CCDC59	10	0.42148929	2.57E-06	0.04692614	0.621	0.268
ATP6V0E1	10	0.41945605	1.57E-06	0.02867889	0.952	0.786
CSNK2A2	10	0.41001476	2.50E-06	0.04556257	0.434	0.089
TBCA	10	0.40165509	4.76E-08	0.00086936	1	1
OAZ1	10	0.37306765	8.87E-08	0.0016184	1	0.982
AC090498.1	10	0.36360328	2.94E-12	5.36E-08	1	1
ZFHX4	10	-0.2515008	6.85E-07	0.01250387	0.028	0.25
RPL39	10	-0.2534405	3.75E-09	6.85E-05	1	1
UBA52	10	-0.2543924	1.70E-07	0.00310928	1	1
MKX	10	-0.2582349	9.94E-07	0.01814199	0.069	0.339
RPL18A	10	-0.2598134	1.84E-09	3.36E-05	1	1

SPTLC3	10	-0.2646658	2.41E-07	0.0043922	0.069	0.357
RP11-71N10.1	10	-0.2651078	1.01E-08	0.00018464	0	0.214
RP11-798M19.6	10	-0.2700693	2.66E-06	0.04855226	0.11	0.411
RPL7A	10	-0.2755897	4.54E-08	0.00082795	1	1
RPS7	10	-0.2772108	9.06E-08	0.00165403	1	1
CLDN11	10	-0.2784376	2.78E-08	0.00050775	0.014	0.25
HOXB8	10	-0.2784376	1.25E-07	0.00228638	0.014	0.232
RPSA	10	-0.2830149	6.55E-07	0.01194323	1	1
RPL11	10	-0.286462	4.34E-11	7.92E-07	1	1
DPYSL3	10	-0.2950488	8.31E-08	0.00151648	0.076	0.393
HACD4	10	-0.2964561	1.93E-08	0.0003522	0.062	0.375
RPL30	10	-0.2988511	2.93E-09	5.35E-05	1	1
RPL13	10	-0.3016178	3.24E-13	5.91E-09	1	1
RPS3	10	-0.3021244	1.54E-12	2.82E-08	1	1
RPLP1	10	-0.3076872	1.58E-14	2.89E-10	1	1
CA11	10	-0.3161288	7.89E-09	0.00014394	0.041	0.339
NKX3-2	10	-0.3184537	4.98E-10	9.10E-06	0	0.25
RPS17	10	-0.322106	5.45E-11	9.95E-07	1	1
MAGEF1	10	-0.32354	3.25E-07	0.00592825	0.097	0.411
AFF3	10	-0.3244843	1.89E-08	0.00034539	0.007	0.232
RPL6	10	-0.3248356	4.59E-10	8.37E-06	1	1
PPP1R16A	10	-0.3251291	1.23E-07	0.00225272	0.083	0.393
SNHG19	10	-0.3251291	4.95E-07	0.00903493	0.083	0.375
SNHG18	10	-0.3256762	4.89E-07	0.00892733	0.159	0.5
RPS18	10	-0.3268084	8.25E-13	1.51E-08	1	1
BTG2	10	-0.3285514	1.70E-09	3.10E-05	0.041	0.357
RPL23	10	-0.3287487	1.23E-08	0.00022409	1	1
RPS29	10	-0.3302084	3.48E-11	6.35E-07	1	1
ENAH	10	-0.3305555	7.51E-08	0.00136985	0.09	0.411
RP11-553L6.5	10	-0.3331979	1.31E-06	0.02397962	0.138	0.446
RPL14	10	-0.3338939	2.29E-12	4.18E-08	1	1
RP5-1097P24.1	10	-0.3342208	3.42E-07	0.00624692	0.048	0.304
NPM1	10	-0.3388793	3.41E-07	0.00622947	1	1
RPS13	10	-0.3425035	1.27E-09	2.32E-05	1	1
AC005152.3	10	-0.3440962	2.32E-11	4.23E-07	0	0.286
RPL8	10	-0.3446407	6.43E-14	1.17E-09	1	1
RP11-983P16.4	10	-0.3462036	1.80E-06	0.03284803	0.166	0.482
RPL9	10	-0.3465573	2.23E-10	4.06E-06	1	1
RPL5	10	-0.3480441	3.18E-11	5.80E-07	1	1

EIF3K	10	-0.3482105	1.62E-06	0.0295586	0.766	0.982
RPL15	10	-0.349786	1.26E-17	2.30E-13	1	1
CPQ	10	-0.3513253	5.19E-07	0.00946726	0.117	0.429
RPS5	10	-0.3538077	8.74E-14	1.59E-09	1	1
GSTA4	10	-0.3577645	1.47E-06	0.02688702	0.159	0.464
CCDC8	10	-0.3595874	9.46E-09	0.00017264	0.028	0.304
KDELR3	10	-0.3598147	1.04E-07	0.00189408	0.083	0.393
H1FX	10	-0.3598147	1.12E-06	0.0204899	0.083	0.357
RPS4X	10	-0.3599019	2.82E-13	5.15E-09	1	1
CCDC102B	10	-0.360889	3.63E-10	6.63E-06	0.021	0.321
RPS9	10	-0.3613791	1.09E-14	1.99E-10	1	1
ANGPTL5	10	-0.3648446	1.73E-07	0.00315325	0.09	0.393
RPS6	10	-0.3663867	1.46E-16	2.66E-12	1	1
RPS23	10	-0.3673468	2.53E-14	4.61E-10	1	1
LRRC2	10	-0.3687767	7.27E-07	0.01327062	0.062	0.321
C17orf89	10	-0.3689004	1.91E-06	0.03490555	0.234	0.571
RPS16	10	-0.3711294	8.39E-12	1.53E-07	1	1
PDGFRA	10	-0.3724731	4.64E-08	0.00084726	0.069	0.375
PYCARD	10	-0.3733042	3.27E-07	0.00596477	0.103	0.411
TOMM7	10	-0.3742764	5.25E-12	9.58E-08	1	1
SOCS3	10	-0.3746452	2.06E-06	0.03760608	0.097	0.375
RPL13A	10	-0.3747202	3.94E-17	7.19E-13	1	1
ERGIC3	10	-0.3750568	6.25E-07	0.01140902	0.69	0.946
TMEM98	10	-0.3753602	1.67E-06	0.03050888	0.145	0.446
RPL10	10	-0.3764008	3.81E-19	6.94E-15	1	1
RPS4Y1	10	-0.3782781	4.82E-09	8.79E-05	0.993	1
ABHD14A	10	-0.383398	6.60E-09	0.00012044	0.034	0.321
FGF7	10	-0.3837726	5.63E-07	0.01027357	0.069	0.339
RBM3	10	-0.3894271	1.29E-06	0.0234706	0.352	0.714
PIGP	10	-0.3907241	1.15E-06	0.02092395	0.441	0.768
RPLP2	10	-0.3914107	2.86E-16	5.22E-12	1	1
DNM3OS	10	-0.3948104	2.82E-07	0.00513902	0.103	0.411
01-Sep	10	-0.3965933	2.36E-10	4.30E-06	0.062	0.429
EIF2A	10	-0.3973064	1.19E-06	0.02167373	0.614	0.857
SPON2	10	-0.3982809	1.33E-09	2.44E-05	0.048	0.375
ILF3-AS1	10	-0.4002051	7.13E-07	0.01301493	0.193	0.518
RPL18	10	-0.4021146	1.02E-11	1.87E-07	1	1
SEPP1	10	-0.4021291	1.68E-07	0.00306037	0.138	0.464
RPS2	10	-0.4023099	1.16E-15	2.11E-11	1	1

ANKRD35	10	-0.4048383	1.23E-11	2.25E-07	0.048	0.429
DMKN	10	-0.4048383	3.93E-07	0.00717687	0.159	0.482
ST13	10	-0.4068293	3.38E-09	6.17E-05	0.945	1
AC003090.1	10	-0.4085171	3.03E-10	5.52E-06	0.021	0.321
EIF3E	10	-0.410195	6.50E-09	0.00011858	1	1
NAP1L1	10	-0.4102928	1.58E-08	0.00028806	0.945	0.982
DBP	10	-0.4105756	8.43E-11	1.54E-06	0.083	0.482
PLPP1	10	-0.4123856	6.18E-08	0.00112739	0.138	0.482
RPS27A	10	-0.4151757	4.90E-18	8.94E-14	1	1
CTGF	10	-0.4162391	7.55E-08	0.00137784	0.207	0.607
MIR99AHG	10	-0.4207538	5.52E-09	0.00010065	0.048	0.357
PRKCZ	10	-0.4257139	1.74E-07	0.00316752	0.2	0.554
RPL19	10	-0.4268144	7.17E-20	1.31E-15	1	1
RABAC1	10	-0.4302471	2.43E-07	0.00442584	0.662	0.946
SPINT2	10	-0.4315066	1.44E-06	0.02635542	0.221	0.536
BTF3	10	-0.4356476	1.55E-12	2.84E-08	0.993	1
TSPO	10	-0.4395043	8.05E-08	0.00146832	0.614	0.929
BGN	10	-0.4405564	1.99E-07	0.00362294	0.966	1
PNKD	10	-0.4405564	1.31E-08	0.00023917	0.172	0.554
ATPIF1	10	-0.4496297	1.81E-07	0.00330474	0.455	0.804
RPL3	10	-0.450594	4.24E-17	7.73E-13	1	1
NDUFA11	10	-0.4537269	1.01E-07	0.00184319	0.71	0.929
COL11A2	10	-0.4598722	3.62E-08	0.00066044	0.193	0.589
MYLK	10	-0.460101	3.12E-13	5.69E-09	0.028	0.429
RPL23A	10	-0.4604567	1.06E-19	1.94E-15	1	1
SSR4	10	-0.4616237	7.50E-08	0.00136883	0.89	0.982
EEF1B2	10	-0.4632048	4.17E-12	7.61E-08	0.986	1
PRDX4	10	-0.4658742	2.17E-07	0.00396226	0.545	0.821
CHID1	10	-0.4683587	4.86E-07	0.0088682	0.276	0.607
PPP1R3C	10	-0.4690579	8.89E-08	0.00162257	0.248	0.625
MPP6	10	-0.4754437	1.44E-08	0.0002635	0.145	0.5
FTL	10	-0.4758201	1.59E-11	2.90E-07	1	1
HDLBP	10	-0.4804023	8.18E-09	0.00014934	0.31	0.696
PRCP	10	-0.4879791	1.66E-13	3.03E-09	0.034	0.446
SDF4	10	-0.4882513	9.11E-07	0.01663111	0.386	0.714
VKORC1	10	-0.4905109	1.13E-10	2.06E-06	0.855	0.982
HIST1H4C	10	-0.4932732	1.12E-08	0.00020444	0.338	0.75
RPL34	10	-0.4940854	4.30E-20	7.85E-16	1	1
FTH1	10	-0.4961562	2.23E-11	4.07E-07	1	1

ATRNL1	10	-0.5003064	6.08E-11	1.11E-06	0.076	0.464
YBX1	10	-0.5027177	1.20E-11	2.20E-07	0.972	0.982
PCOLCE2	10	-0.5047987	7.80E-09	0.00014231	0.71	1
FCGRT	10	-0.5047987	3.05E-08	0.0005565	0.331	0.696
PLXDC2	10	-0.506071	1.45E-08	0.00026478	0.276	0.643
IGFBP6	10	-0.5065418	2.26E-06	0.04131048	0.428	0.75
RPS3A	10	-0.5086969	2.30E-19	4.20E-15	1	1
PCBP2	10	-0.5097956	6.33E-11	1.15E-06	0.855	1
AHNAK	10	-0.5114308	3.50E-09	6.38E-05	0.186	0.571
TPT1	10	-0.5153335	4.32E-17	7.89E-13	1	1
H3F3A	10	-0.5169788	1.21E-09	2.21E-05	0.924	1
ZBTB20	10	-0.5243445	2.23E-08	0.00040717	0.131	0.464
TCEA3	10	-0.5254783	2.93E-11	5.35E-07	0.159	0.607
ARHGAP29	10	-0.5304128	1.27E-07	0.00232231	0.241	0.589
ITM2C	10	-0.5378197	1.78E-08	0.00032538	0.2	0.589
ENPP1	10	-0.5459169	2.12E-13	3.87E-09	0.076	0.536
TRPS1	10	-0.5459169	2.52E-09	4.60E-05	0.234	0.625
SSPN	10	-0.5490371	2.11E-12	3.85E-08	0.09	0.536
CTSC	10	-0.5554864	7.60E-08	0.00138633	0.062	0.357
XAGE2	10	-0.5596158	1.02E-08	0.00018618	0	0.214
SSR3	10	-0.5659845	1.69E-09	3.08E-05	0.828	0.982
ITM2A	10	-0.5663258	2.84E-08	0.00051847	0.248	0.607
PDIA6	10	-0.5701739	5.30E-08	0.00096712	0.338	0.714
BRK1	10	-0.5714502	4.84E-11	8.83E-07	0.662	0.982
NFIA	10	-0.5728244	1.32E-12	2.40E-08	0.124	0.589
PTRF	10	-0.573873	3.42E-09	6.25E-05	0.248	0.661
SUMF2	10	-0.5740878	1.05E-06	0.01924953	0.441	0.732
VIM	10	-0.5849747	5.45E-12	9.95E-08	1	1
MDFI	10	-0.5878079	6.25E-09	0.00011407	0.241	0.607
HMGN2	10	-0.5887806	1.01E-09	1.84E-05	0.766	0.946
CXXC5	10	-0.5892674	9.58E-12	1.75E-07	0.09	0.5
SLC44A1	10	-0.5913793	3.05E-12	5.56E-08	0.159	0.625
HLA-A	10	-0.5958616	4.00E-08	0.00073019	0.738	0.964
EID1	10	-0.5959818	1.36E-13	2.48E-09	0.862	0.982
RPL7	10	-0.5963637	1.44E-19	2.63E-15	1	1
RPLP0	10	-0.5978305	2.80E-23	5.11E-19	1	1
S100A4	10	-0.6080487	1.04E-07	0.00190045	0.497	0.875
IGFBP7	10	-0.6083308	1.22E-06	0.02217088	0.676	0.893
BHLHE41	10	-0.611222	1.23E-10	2.24E-06	0.124	0.518

TXNIP	10	-0.6121663	9.53E-10	1.74E-05	0.007	0.268
S100A13	10	-0.6142713	5.97E-15	1.09E-10	0.979	1
EIF3L	10	-0.6182939	2.94E-11	5.37E-07	0.766	0.982
SLC14A1	10	-0.618372	4.66E-12	8.51E-08	0.172	0.625
PTMS	10	-0.6340242	2.99E-09	5.45E-05	0.372	0.732
MIA	10	-0.636731	3.30E-11	6.02E-07	0.931	1
CUTA	10	-0.638242	3.50E-14	6.39E-10	0.634	0.946
THY1	10	-0.6412271	3.80E-13	6.94E-09	0.131	0.607
CCDC80	10	-0.6439782	3.14E-09	5.72E-05	0.676	0.946
S100A1	10	-0.6450824	2.19E-12	3.99E-08	1	1
INHBA	10	-0.6460004	5.35E-11	9.76E-07	0.131	0.554
APOD	10	-0.6484325	1.72E-10	3.13E-06	0.145	0.571
PEBP1	10	-0.6511212	1.07E-11	1.96E-07	0.634	0.929
HSP90AB1	10	-0.6524816	2.81E-13	5.12E-09	0.986	1
RPS20	10	-0.6530461	1.42E-21	2.60E-17	1	1
CCNI	10	-0.6544454	3.70E-17	6.76E-13	1	1
GAS6	10	-0.6637	1.02E-14	1.86E-10	0.131	0.643
HTRA1	10	-0.6656474	1.08E-11	1.97E-07	0.524	0.929
RPS8	10	-0.6664253	1.95E-23	3.57E-19	1	1
VCAN	10	-0.6872305	1.53E-12	2.79E-08	0.11	0.571
NPC2	10	-0.6915745	2.53E-17	4.62E-13	0.938	1
RPL10A	10	-0.6996296	1.82E-23	3.32E-19	1	1
RPL4	10	-0.7147178	2.41E-21	4.40E-17	0.993	1
RPS11	10	-0.7183748	8.06E-21	1.47E-16	0.979	1
ID4	10	-0.7216667	1.81E-11	3.31E-07	0.31	0.786
FBXO2	10	-0.7219236	1.66E-13	3.02E-09	0.559	0.964
SERPINA5	10	-0.7225405	8.11E-13	1.48E-08	0.172	0.643
SPARC	10	-0.737808	4.96E-14	9.05E-10	0.186	0.714
CRTAP	10	-0.7413678	6.45E-16	1.18E-11	0.421	0.893
NNMT	10	-0.7433815	5.69E-10	1.04E-05	0.717	0.982
PABPC1	10	-0.7495827	5.91E-14	1.08E-09	0.731	0.982
MT-ND4	10	-0.7512228	6.74E-11	1.23E-06	0.91	1
COL9A3	10	-0.7532682	1.09E-13	1.99E-09	0.241	0.786
HNRNPA1	10	-0.7549758	4.85E-20	8.85E-16	0.979	1
JUNB	10	-0.7666479	2.21E-11	4.03E-07	0.352	0.821
YBX3	10	-0.7734706	1.10E-15	2.01E-11	0.655	0.964
GOLIM4	10	-0.7754914	7.19E-15	1.31E-10	0.717	0.964
CRISPLD1	10	-0.7868327	2.22E-21	4.06E-17	0	0.536
ID2	10	-0.7956798	2.02E-10	3.69E-06	0.303	0.714

MALAT1	10	-0.7959471	1.62E-15	2.96E-11	1	1
SNAI2	10	-0.7964336	1.75E-11	3.20E-07	0.276	0.732
S100B	10	-0.803408	4.15E-15	7.57E-11	0.462	0.946
SLC40A1	10	-0.8072482	9.68E-17	1.77E-12	0.028	0.5
TCF4	10	-0.8082812	3.02E-14	5.52E-10	0.221	0.696
CD99	10	-0.8171325	2.83E-14	5.17E-10	0.786	0.982
DDR2	10	-0.8280588	4.28E-13	7.82E-09	0.214	0.679
P4HB	10	-0.8305772	3.69E-14	6.74E-10	0.51	0.929
RHOBTB3	10	-0.8421828	4.46E-15	8.14E-11	0.276	0.786
COL11A1	10	-0.8478414	6.73E-14	1.23E-09	0.372	0.875
EEF1A1	10	-0.8546167	1.13E-26	2.06E-22	1	1
EIF3H	10	-0.8549422	3.85E-21	7.03E-17	0.952	1
RBP4	10	-0.855154	1.41E-12	2.58E-08	0.338	0.804
FOS	10	-0.8575633	6.79E-16	1.24E-11	0.262	0.804
FXYD6	10	-0.8591181	3.67E-18	6.69E-14	0.572	0.982
ISLR	10	-0.8680004	8.80E-21	1.60E-16	0.034	0.607
CST3	10	-0.8786628	3.34E-16	6.10E-12	0.945	1
CP	10	-0.8844114	6.62E-18	1.21E-13	0.393	0.929
DCN	10	-0.9088061	4.31E-20	7.87E-16	1	1
EEF2	10	-0.9230931	7.79E-19	1.42E-14	0.986	1
CYR61	10	-0.940146	4.46E-12	8.14E-08	0.103	0.536
C2orf40	10	-0.9652786	4.98E-16	9.09E-12	0.945	1
B2M	10	-0.9662976	3.51E-19	6.40E-15	0.959	1
NGFRAP1	10	-0.9749919	6.26E-19	1.14E-14	0.545	0.982
FMOD	10	-0.9757405	8.14E-19	1.49E-14	0.621	0.982
PRELP	10	-0.9808961	1.08E-17	1.97E-13	0.676	0.982
MT-ND3	10	-0.9995314	4.45E-22	8.11E-18	0.255	0.964
MT-CO3	10	-1.0409942	3.82E-17	6.97E-13	0.814	1
ID1	10	-1.0571717	1.29E-14	2.36E-10	0.269	0.804
MT-ND1	10	-1.0647107	2.83E-18	5.16E-14	0.331	0.893
MT-CO1	10	-1.0649909	3.31E-16	6.04E-12	0.924	1
MT-ND2	10	-1.1389217	2.47E-19	4.50E-15	0.331	0.929
ADIRF	10	-1.1399732	3.82E-17	6.98E-13	0.269	0.857
ACAN	10	-1.140909	8.42E-19	1.54E-14	0.752	0.982
ZFP36L2	10	-1.1593826	6.62E-15	1.21E-10	0.159	0.661
PAX1	10	-1.1678684	2.68E-23	4.89E-19	0.31	0.946
LECT1	10	-1.1886513	3.00E-21	5.47E-17	0.31	0.964
GPX3	10	-1.190399	1.17E-21	2.13E-17	0.766	1
C2orf82	10	-1.2403605	2.30E-17	4.19E-13	0.945	0.982

MT-ATP6	10	-1.2486336	1.58E-19	2.89E-15	0.641	0.982
MT-CYB	10	-1.2676516	1.29E-20	2.36E-16	0.421	0.964
NEAT1	10	-1.2696433	2.15E-23	3.92E-19	0.979	1
MT-CO2	10	-1.281732	1.69E-18	3.08E-14	0.855	1
CAPS	10	-1.2840878	7.16E-19	1.31E-14	0.462	0.946
TSC22D1	10	-1.3033185	3.69E-17	6.73E-13	0.421	0.893
SLPI	10	-1.3284582	1.01E-18	1.84E-14	0.71	1
PLAC9	10	-1.3313139	3.82E-24	6.98E-20	0.345	0.982
MGP	10	-1.4137439	1.65E-18	3.01E-14	0.993	1
CITED2	10	-1.4213857	2.89E-14	5.28E-10	0.152	0.643
SPTSSB	10	-1.4256868	2.28E-29	4.16E-25	0	0.714
ID3	10	-1.592039	2.50E-31	4.56E-27	0.11	0.946
COL2A1	10	-1.5998291	2.21E-26	4.03E-22	0.159	0.893
FRZB	10	-1.7994913	3.23E-25	5.90E-21	0.117	0.839
CLEC3A	10	-2.1071528	1.17E-37	2.13E-33	0.041	0.946
OGN	10	-2.2179229	1.24E-27	2.25E-23	0.559	1

gene	cluster	avg_logFC	p_val	p_val_adj	pct.1	pct.2
SPP1	11	2.32285066	4.43E-20	8.09E-16	0.808	0.128
SOD2	11	1.55849003	5.45E-20	9.95E-16	0.986	0.743
COMP	11	1.33567376	4.94E-11	9.02E-07	0.822	0.56
CXCL8	11	1.2508041	2.14E-06	0.03899892	0.192	0
PRG4	11	1.12070387	3.36E-14	6.14E-10	0.479	0.018
DNAJB9	11	1.08346949	1.75E-15	3.19E-11	0.932	0.679
MT2A	11	1.08336918	6.09E-10	1.11E-05	1	1
UPP1	11	1.005002	2.55E-15	4.65E-11	0.726	0.193
WTAP	11	0.99125575	5.49E-16	1.00E-11	0.945	0.532
CD55	11	0.97453408	9.92E-17	1.81E-12	0.89	0.468
FN1	11	0.91785533	1.31E-09	2.39E-05	1	0.991
SLC3A2	11	0.90893242	2.96E-12	5.40E-08	0.849	0.569
ARF4	11	0.88639626	6.15E-17	1.12E-12	0.973	0.725
VEGFA	11	0.81862364	9.67E-11	1.76E-06	0.616	0.202
SMOX	11	0.79060519	3.29E-11	6.00E-07	0.589	0.156
RABGGTB	11	0.78775623	1.35E-13	2.46E-09	0.849	0.532
MIF	11	0.78197311	1.53E-13	2.79E-09	1	1
HMGA1	11	0.77726059	1.07E-08	0.00019605	0.658	0.312
CTSL	11	0.77474821	8.42E-08	0.00153715	0.836	0.468
SNHG12	11	0.76959265	3.99E-11	7.28E-07	0.712	0.275
VIMP	11	0.74919514	2.08E-11	3.79E-07	0.973	0.807
NRP2	11	0.74181503	5.91E-07	0.01078858	0.452	0.147

ARF6	11	0.74056581	7.34E-13	1.34E-08	0.808	0.45
TRMT112	11	0.69922781	1.15E-17	2.09E-13	1	0.917
SELK	11	0.68784771	2.14E-08	0.00038998	0.945	0.826
XBP1	11	0.68699198	4.64E-10	8.46E-06	0.932	0.798
GOLT1B	11	0.68527562	1.11E-07	0.00203007	0.63	0.303
TAF1D	11	0.68156278	3.50E-09	6.39E-05	0.753	0.44
PTGS2	11	0.66325271	3.26E-10	5.95E-06	0.342	0.009
BHLHE40	11	0.65070024	2.49E-06	0.04542386	0.548	0.257
DDIT3	11	0.64656111	1.47E-08	0.00026791	0.945	0.807
STK17A	11	0.64043803	4.99E-08	0.00091025	0.548	0.202
NDRG1	11	0.61620927	2.74E-10	5.00E-06	0.712	0.312
MGST1	11	0.61044315	6.33E-07	0.01155814	0.589	0.239
SAR1A	11	0.60964325	2.38E-07	0.00435188	0.699	0.413
VAPA	11	0.60538964	4.65E-13	8.48E-09	0.986	0.927
ERO1A	11	0.59255586	1.48E-09	2.69E-05	0.63	0.22
ZNF593	11	0.58629166	1.15E-09	2.11E-05	0.726	0.367
GDF15	11	0.58321	1.42E-06	0.02599629	0.603	0.275
WDR33	11	0.56073714	9.23E-10	1.68E-05	0.699	0.312
GSTO1	11	0.5496337	7.29E-07	0.01330883	0.808	0.606
RND3	11	0.54787063	1.53E-09	2.79E-05	0.411	0.046
ADSS	11	0.54749192	4.10E-07	0.00747311	0.767	0.477
RNF114	11	0.54320466	7.96E-10	1.45E-05	0.753	0.394
CCND1	11	0.54097723	8.35E-07	0.01523366	0.438	0.128
PMP22	11	0.53045943	1.64E-06	0.02993205	0.849	0.651
NXT1	11	0.52730237	1.48E-07	0.00270147	0.589	0.257
ENO2	11	0.52518616	1.08E-06	0.01979109	0.507	0.202
GBE1	11	0.51680754	6.68E-07	0.01219579	0.726	0.459
COPS2	11	0.51545501	3.78E-07	0.00689854	0.726	0.394
C11orf96	11	0.51391183	1.91E-08	0.00034885	0.384	0.055
OSER1	11	0.50962164	1.16E-07	0.00211094	0.63	0.294
SPRY2	11	0.50952228	5.95E-07	0.01084881	0.411	0.11
RELB	11	0.49852691	4.30E-09	7.84E-05	0.425	0.073
TRAM1	11	0.49648356	6.33E-09	0.00011543	0.986	0.771
BZW1	11	0.49572254	1.59E-07	0.00290008	0.753	0.422
NOL7	11	0.48830038	2.52E-06	0.04598008	0.822	0.624
CTHRC1	11	0.48358016	5.60E-09	0.00010223	0.397	0.055
SYF2	11	0.47645805	1.43E-09	2.62E-05	0.986	0.752
PLAUR	11	0.47611186	2.08E-06	0.03792303	0.411	0.11
TGFBI	11	0.46542696	2.18E-07	0.00398074	0.397	0.092

TREM1	11	0.46313075	3.16E-09	5.77E-05	0.288	0
PTGES3	11	0.46057892	2.47E-09	4.51E-05	0.986	0.954
EIF4A2	11	0.46031186	1.09E-07	0.00199227	0.973	0.936
NAMPT	11	0.45972894	9.05E-07	0.01651527	0.685	0.33
AK2	11	0.45688271	7.55E-07	0.01378247	0.767	0.523
ZFAS1	11	0.44227366	8.73E-10	1.59E-05	1	0.991
ZNF581	11	0.43597976	3.82E-08	0.00069646	0.616	0.239
CCDC174	11	0.43052024	3.22E-08	0.00058724	0.562	0.183
EIF1	11	0.43038762	2.48E-11	4.52E-07	1	1
CLDND1	11	0.42136697	1.73E-06	0.03159824	0.603	0.284
SLC7A2	11	0.41843275	1.70E-07	0.00309342	0.315	0.037
ZFAND2A	11	0.41592632	2.55E-07	0.00465322	0.479	0.138
CD44	11	0.40088844	1.96E-06	0.03580629	0.849	0.615
AC090498.1	11	0.36375276	1.19E-10	2.17E-06	1	1
HMGA2	11	0.35436843	2.93E-07	0.00534245	0.247	0.009
C2orf49	11	0.35363556	4.67E-07	0.00851826	0.507	0.165
FAP	11	0.32942948	1.49E-07	0.00271078	0.37	0.064
EMC9	11	0.30953866	2.04E-06	0.03713938	0.452	0.138
NPR3	11	0.27388875	8.68E-07	0.01584719	0.205	0
RPL11	11	-0.2546833	1.71E-07	0.00312917	1	1
RPS29	11	-0.2563402	9.95E-09	0.00018148	1	1
RPL15	11	-0.2616234	6.64E-10	1.21E-05	1	1
RPS27A	11	-0.2669315	1.69E-09	3.09E-05	1	1
RPL37	11	-0.2722409	1.61E-08	0.00029359	1	1
RPLP1	11	-0.2733152	4.32E-11	7.88E-07	1	1
RPS4X	11	-0.274206	6.07E-07	0.01108205	1	1
RPL27	11	-0.2765294	3.85E-07	0.00702141	1	1
RPS23	11	-0.2833168	1.96E-08	0.00035678	1	1
COA5	11	-0.2922587	1.62E-06	0.02962292	0.096	0.431
SERF2	11	-0.2984354	6.98E-07	0.01272849	1	1
RPL13	11	-0.3026666	1.41E-10	2.57E-06	1	1
HOXB8	11	-0.3120614	1.42E-06	0.02596714	0.027	0.321
AC003090.1	11	-0.3123263	1.03E-06	0.01877188	0.014	0.303
RPS6	11	-0.3129144	8.26E-12	1.51E-07	1	1
RPS5	11	-0.3261603	3.02E-09	5.52E-05	0.986	1
RPS12	11	-0.3271915	5.08E-10	9.28E-06	1	1
RPS7	11	-0.3352707	2.69E-10	4.92E-06	1	1
RPL29	11	-0.3359102	6.64E-12	1.21E-07	1	1
RPL30	11	-0.3381383	3.43E-11	6.26E-07	1	1

RPS16	11	-0.3436451	2.06E-11	3.75E-07	1	1
RPL18	11	-0.3453238	2.25E-11	4.10E-07	1	1
RPL23A	11	-0.3472131	4.83E-11	8.82E-07	1	1
RPL5	11	-0.3484272	6.69E-09	0.00012209	1	1
RPL34	11	-0.3534426	2.50E-11	4.55E-07	1	1
RPS18	11	-0.3580359	3.54E-14	6.46E-10	1	1
ZSCAN16-AS1	11	-0.360467	7.82E-07	0.01426039	0.151	0.505
TOMM7	11	-0.3631428	4.16E-11	7.59E-07	1	1
RPL37A	11	-0.3631938	1.30E-13	2.37E-09	1	1
SLC40A1	11	-0.3639507	1.01E-06	0.01842724	0.014	0.303
RNASET2	11	-0.3645794	2.25E-06	0.04096707	0.096	0.413
RPLP2	11	-0.3778194	2.17E-15	3.96E-11	1	1
C17orf89	11	-0.3788268	6.78E-07	0.01236406	0.26	0.606
SSPN	11	-0.380366	9.90E-07	0.01806173	0.041	0.349
ERGIC3	11	-0.3858413	3.84E-07	0.00700051	0.671	0.917
EEF1B2	11	-0.3890902	6.49E-08	0.00118444	0.973	0.991
TMEM98	11	-0.3898972	8.32E-08	0.00151882	0.068	0.431
RPL9	11	-0.3911161	2.17E-13	3.96E-09	1	1
RPL6	11	-0.3918776	2.43E-12	4.43E-08	1	1
PPIA	11	-0.3993845	2.15E-06	0.03929766	0.932	0.972
HMGN3	11	-0.4010616	2.65E-06	0.04828189	0.178	0.505
DBP	11	-0.4031594	1.13E-07	0.00206191	0.11	0.477
LRP10	11	-0.4055874	1.40E-06	0.02562998	0.342	0.67
HNRNPM	11	-0.4076749	9.27E-07	0.01691997	0.247	0.587
RPS19	11	-0.4087665	5.23E-14	9.54E-10	1	1
ANAPC16	11	-0.4094776	3.57E-07	0.00652061	0.753	0.899
RPL3	11	-0.4143481	1.20E-12	2.19E-08	1	1
TUBB	11	-0.4145161	1.58E-06	0.02890436	0.795	0.927
PRDX4	11	-0.4148611	5.76E-07	0.01051125	0.575	0.835
FTL	11	-0.4160959	2.62E-09	4.77E-05	1	1
PCBP2	11	-0.4187186	7.09E-08	0.00129438	0.74	0.936
DYNLT1	11	-0.4188914	8.43E-08	0.00153783	0.26	0.679
GRN	11	-0.4200921	1.16E-07	0.00211288	0.11	0.505
TSPAN4	11	-0.4217928	4.44E-07	0.00809805	0.205	0.578
RPL19	11	-0.4231321	1.07E-17	1.95E-13	1	1
ARL6IP5	11	-0.423287	1.37E-06	0.02498362	0.411	0.743
IER2	11	-0.4257901	1.95E-08	0.00035553	0.123	0.541
AHNAK	11	-0.4278042	2.29E-06	0.04173772	0.247	0.578
IFI27L2	11	-0.4285038	1.86E-06	0.03395798	0.589	0.844

NUPR1	11	-0.4309224	8.47E-07	0.01546223	1	0.991
S100A1	11	-0.4311535	8.99E-07	0.01640308	0.986	0.991
RPS3A	11	-0.4314642	1.54E-15	2.80E-11	1	1
ZBTB20	11	-0.4464094	3.16E-08	0.00057695	0.137	0.532
MYL6	11	-0.4464094	6.41E-07	0.01169947	0.822	0.954
PRKCDBP	11	-0.4478228	1.25E-06	0.02288459	0.329	0.661
CRISPLD1	11	-0.4501618	1.57E-06	0.02867612	0.041	0.339
SH3BGRL	11	-0.45116	2.06E-07	0.00374984	0.192	0.569
PRDX5	11	-0.4532481	1.66E-08	0.00030266	0.795	0.972
RPSA	11	-0.4538032	9.10E-12	1.66E-07	0.986	1
RPS17	11	-0.4667259	2.04E-16	3.72E-12	1	1
VKORC1	11	-0.4679671	5.38E-07	0.0098117	0.63	0.917
ARHGAP29	11	-0.4681494	5.47E-07	0.00998174	0.178	0.55
RPS2	11	-0.4713958	3.06E-16	5.58E-12	1	1
GNAS	11	-0.4732567	1.17E-08	0.00021265	0.466	0.826
PCOLCE2	11	-0.4793432	1.31E-07	0.00239831	0.699	0.936
NDUFA11	11	-0.4813257	3.98E-08	0.00072668	0.753	0.927
RPL23	11	-0.4887758	1.78E-12	3.25E-08	1	1
NAP1L1	11	-0.4911729	5.85E-09	0.00010671	0.945	0.982
EIF3L	11	-0.4922508	5.59E-10	1.02E-05	0.795	0.936
ATPIF1	11	-0.5055175	5.90E-09	0.00010767	0.479	0.789
B2M	11	-0.508631	3.17E-10	5.78E-06	0.986	1
ARPC1A	11	-0.5090665	5.07E-08	0.00092517	0.26	0.633
SPON2	11	-0.5127321	2.07E-06	0.03785205	0.027	0.312
TSPO	11	-0.5224363	5.37E-09	9.81E-05	0.658	0.917
RPL7	11	-0.5264141	6.82E-18	1.24E-13	1	1
GAS6	11	-0.5273785	1.63E-08	0.00029716	0.11	0.514
CP	11	-0.5327271	3.29E-08	0.00059988	0.247	0.661
PPT1	11	-0.5334208	2.48E-10	4.53E-06	0.178	0.642
LITAF	11	-0.5346842	1.15E-08	0.00021004	0.397	0.752
RPN2	11	-0.5389328	2.25E-08	0.00041038	0.137	0.532
VCAN	11	-0.5406137	9.62E-09	0.00017561	0.164	0.596
YBX3	11	-0.5432712	6.82E-10	1.24E-05	0.726	0.936
EIF3H	11	-0.5519502	1.91E-09	3.49E-05	0.904	0.991
RPLP0	11	-0.5525946	1.68E-19	3.07E-15	1	1
ITM2C	11	-0.5537384	1.13E-10	2.06E-06	0.178	0.642
EID1	11	-0.5549758	4.55E-11	8.30E-07	0.753	1
TPT1	11	-0.5568566	2.72E-18	4.95E-14	1	1
SCRG1	11	-0.5636321	1.28E-08	0.00023271	0.904	0.945

MALAT1	11	-0.5713539	8.92E-09	0.00016274	1	1
MDFI	11	-0.5734767	1.15E-07	0.00210454	0.247	0.606
CYR61	11	-0.5783161	2.67E-06	0.04872412	0.055	0.349
JUNB	11	-0.5789862	2.67E-09	4.87E-05	0.342	0.771
H3F3A	11	-0.583965	2.87E-13	5.23E-09	0.918	0.991
PEBP1	11	-0.5856065	2.27E-10	4.14E-06	0.644	0.936
C7orf73	11	-0.5871462	3.83E-08	0.00069953	0.438	0.752
HMGN2	11	-0.5915637	3.59E-10	6.55E-06	0.877	0.982
PTRF	11	-0.5935836	8.62E-09	0.00015726	0.192	0.606
PABPC1	11	-0.6000084	1.35E-11	2.46E-07	0.795	0.972
TTC3	11	-0.6012636	2.05E-09	3.74E-05	0.452	0.807
THY1	11	-0.6061567	7.92E-08	0.00144586	0.096	0.45
CITED2	11	-0.609713	2.04E-06	0.03720078	0.205	0.532
VIM	11	-0.6157448	2.50E-09	4.56E-05	0.986	1
ITM2A	11	-0.6160458	1.12E-10	2.04E-06	0.137	0.606
PDIA6	11	-0.6229826	5.84E-09	0.00010649	0.616	0.881
S100A13	11	-0.6266279	4.87E-14	8.88E-10	0.932	1
YBX1	11	-0.6320797	7.76E-16	1.42E-11	0.973	1
BRK1	11	-0.63542	8.53E-14	1.56E-09	0.589	0.945
PKM	11	-0.6363068	4.76E-08	0.00086797	0.849	0.982
ZFP36L2	11	-0.6376199	1.58E-08	0.00028874	0.096	0.486
FBXO2	11	-0.6390751	1.67E-08	0.00030427	0.548	0.807
INHBA	11	-0.6412116	2.31E-07	0.00421518	0.178	0.541
RPS11	11	-0.6472691	2.44E-19	4.45E-15	1	1
DNM3OS	11	-0.6501987	2.16E-08	0.00039364	0.082	0.459
TPM1	11	-0.6536843	2.03E-08	0.00036966	0.493	0.835
PLXDC2	11	-0.6559842	6.96E-09	0.00012698	0.301	0.688
RPL10A	11	-0.6561249	8.70E-18	1.59E-13	0.986	1
ISLR	11	-0.6673527	2.29E-10	4.19E-06	0.068	0.514
RPS8	11	-0.6718561	1.84E-22	3.36E-18	1	1
SDC2	11	-0.67834	5.14E-12	9.37E-08	0.616	0.963
HNRNPA1	11	-0.7029071	2.80E-13	5.11E-09	0.959	0.991
RHOBTB3	11	-0.7044353	1.80E-14	3.29E-10	0.233	0.817
FXYD6	11	-0.707699	6.84E-13	1.25E-08	0.479	0.89
FOS	11	-0.7094199	4.76E-11	8.69E-07	0.137	0.606
CRTAP	11	-0.7159062	6.49E-14	1.18E-09	0.342	0.844
IGFBP6	11	-0.7242506	8.90E-09	0.0001624	0.384	0.743
NPC2	11	-0.7322717	4.77E-14	8.70E-10	0.877	0.982
RPS20	11	-0.7423327	8.20E-25	1.50E-20	1	1

IGFBP7	11	-0.7534658	2.72E-11	4.96E-07	0.507	0.881
P4HB	11	-0.7540125	2.62E-13	4.77E-09	0.849	0.982
SPARC	11	-0.7585791	6.48E-14	1.18E-09	0.123	0.697
PRELP	11	-0.7607368	7.68E-09	0.00014011	0.63	0.908
COL11A1	11	-0.7622624	1.04E-10	1.89E-06	0.205	0.679
NEAT1	11	-0.7701109	1.28E-12	2.33E-08	0.986	1
COL9A3	11	-0.7750155	3.44E-10	6.28E-06	0.288	0.734
EEF2	11	-0.792375	3.00E-15	5.48E-11	0.973	1
EEF1A1	11	-0.7966586	1.45E-17	2.65E-13	1	1
SNAI2	11	-0.8030844	1.01E-07	0.00184399	0.288	0.615
GPX3	11	-0.8065626	4.55E-14	8.31E-10	0.795	0.991
CD99	11	-0.8105431	2.12E-19	3.87E-15	0.753	0.991
CCNI	11	-0.8133589	1.36E-19	2.47E-15	0.986	1
FMOD	11	-0.8217019	1.19E-12	2.17E-08	0.548	0.881
MIA	11	-0.8239185	6.59E-13	1.20E-08	0.781	0.954
RPL4	11	-0.8299918	7.25E-25	1.32E-20	0.986	1
DCN	11	-0.8471372	1.57E-12	2.87E-08	1	1
PTMS	11	-0.8482048	6.98E-17	1.27E-12	0.37	0.89
MYLK	11	-0.8565474	4.51E-10	8.23E-06	0.301	0.734
LECT1	11	-0.8628036	9.04E-10	1.65E-05	0.26	0.706
CST3	11	-0.8699219	6.96E-16	1.27E-11	0.918	0.991
ACAN	11	-0.8743474	2.29E-12	4.18E-08	0.521	0.908
S100B	11	-0.8795261	1.08E-11	1.97E-07	0.315	0.734
HTRA1	11	-0.8968525	1.68E-15	3.06E-11	0.37	0.881
NGFRAP1	11	-0.9026	6.39E-17	1.17E-12	0.562	0.945
ID2	11	-0.9446947	1.35E-11	2.46E-07	0.164	0.651
ADIRF	11	-0.9478505	4.90E-14	8.94E-10	0.438	0.936
RBP4	11	-1.0198921	1.11E-16	2.03E-12	0.288	0.872
C2orf40	11	-1.0361414	5.70E-13	1.04E-08	0.781	0.972
CAPS	11	-1.037924	8.81E-14	1.61E-09	0.534	0.899
MGP	11	-1.0613715	1.62E-11	2.95E-07	0.986	1
C2orf82	11	-1.0777961	6.66E-13	1.21E-08	0.836	0.991
PAX1	11	-1.0975232	1.33E-21	2.43E-17	0.356	0.972
COL2A1	11	-1.1932151	3.96E-18	7.22E-14	0.205	0.835
ID3	11	-1.2943448	1.26E-14	2.31E-10	0.178	0.734
PLAC9	11	-1.3355209	5.11E-22	9.32E-18	0.438	0.963
ID1	11	-1.3405007	1.19E-12	2.16E-08	0.164	0.67
CLEC3A	11	-1.5309864	1.38E-20	2.52E-16	0.068	0.807
FRZB	11	-1.5599474	4.96E-14	9.05E-10	0.137	0.725

SPTSSB	11	-1.6249253	6.53E-19	1.19E-14	0.055	0.743
OGN	11	-1.6508851	1.94E-20	3.55E-16	0.274	0.954
SLPI	11	-2.3110037	4.43E-20	8.08E-16	0.685	1
**gene**	**cluster**	**avg_logFC**	**p_val**	**p_val_adj**	**pct.1**	**pct.2**
SPP1	12	3.88156734	6.84E-21	1.25E-16	0.964	0.022
PRG4	12	2.62280181	1.88E-15	3.44E-11	0.786	0.022
COMP	12	1.928703	6.30E-20	1.15E-15	1	0.8
MT2A	12	1.57115537	1.70E-13	3.10E-09	1	1
PTGS2	12	1.53991806	1.81E-17	3.30E-13	0.848	0.022
UPP1	12	1.5252796	2.98E-18	5.44E-14	0.893	0.089
IGFBP3	12	1.51089302	8.39E-12	1.53E-07	0.67	0.044
FN1	12	1.37764383	9.04E-19	1.65E-14	1	1
CHI3L2	12	1.25403767	1.81E-06	0.0330434	0.786	0.556
VEGFA	12	1.20842715	4.52E-15	8.24E-11	0.911	0.244
BHLHE40	12	1.20484933	2.26E-13	4.13E-09	0.804	0.156
BZW1	12	1.17318657	9.07E-15	1.65E-10	0.911	0.378
SOD2	12	1.17306641	2.53E-11	4.62E-07	1	0.867
TIMP1	12	1.16316147	9.83E-12	1.79E-07	1	1
TM4SF1	12	1.1518568	1.30E-07	0.00236362	0.83	0.444
ABI3BP	12	1.14801803	1.63E-12	2.98E-08	0.795	0.222
ERO1A	12	1.13426874	7.67E-13	1.40E-08	0.821	0.267
DDIT4L	12	1.11974914	2.96E-13	5.40E-09	0.964	0.333
TIMP3	12	1.11602578	5.95E-09	0.00010854	0.83	0.422
SLC3A2	12	1.07346617	1.32E-14	2.41E-10	0.884	0.311
NRP2	12	1.06989849	2.52E-11	4.61E-07	0.652	0.044
CTSL	12	1.06224464	1.02E-08	0.00018574	0.884	0.511
WTAP	12	1.0611757	1.42E-13	2.59E-09	0.938	0.444
S100A2	12	1.03407377	6.77E-08	0.00123501	0.75	0.356
PTP4A1	12	1.01268258	1.72E-13	3.13E-09	0.812	0.156
CD55	12	1.01089618	4.48E-13	8.18E-09	0.982	0.556
SLC2A1	12	1.00692278	8.49E-12	1.55E-07	0.688	0.067
CD44	12	0.98410644	5.11E-15	9.32E-11	0.991	0.422
ENO2	12	0.98215371	1.90E-14	3.47E-10	0.777	0.067
STK17A	12	0.96747898	3.13E-09	5.71E-05	0.634	0.133
TMEM45A	12	0.96423019	1.27E-10	2.33E-06	0.938	0.578
SMOX	12	0.96303799	1.58E-07	0.00288781	0.545	0.111
MMP3	12	0.9446856	3.82E-08	0.00069743	1	0.956
NDRG1	12	0.92222547	5.68E-11	1.04E-06	0.723	0.111
RGCC	12	0.91167179	2.53E-07	0.00462338	0.973	0.756

C11orf96	12	0.88361995	5.62E-08	0.00102479	0.464	0
DNER	12	0.86893397	7.25E-10	1.32E-05	0.759	0.244
PPP1CB	12	0.86311597	1.66E-08	0.00030322	0.893	0.511
PPP3CA	12	0.85237314	4.12E-10	7.53E-06	0.929	0.467
PPFIBP1	12	0.85121298	1.18E-08	0.00021505	0.634	0.156
PLCG2	12	0.83547133	1.83E-07	0.00334232	0.464	0.022
RABGGTB	12	0.80545893	1.20E-11	2.19E-07	0.884	0.356
DDIT3	12	0.80167089	1.81E-07	0.00330899	0.679	0.289
VAPA	12	0.79417354	6.95E-14	1.27E-09	0.982	0.867
MIF	12	0.78134526	1.23E-16	2.25E-12	1	1
ATP6V1G1	12	0.77764424	2.55E-13	4.65E-09	0.991	0.978
HMGA1	12	0.77217023	6.53E-08	0.00119084	0.696	0.222
IFRD1	12	0.7717095	6.10E-08	0.00111229	0.723	0.333
TGFBI	12	0.76100304	2.20E-06	0.04019776	0.562	0.178
RNF114	12	0.7592951	3.12E-07	0.0056993	0.696	0.356
GBE1	12	0.75658669	8.02E-09	0.00014628	0.848	0.422
RNF145	12	0.74248086	1.56E-07	0.00284985	0.661	0.2
AK2	12	0.74061892	3.10E-10	5.66E-06	0.884	0.556
TREM1	12	0.7282385	8.30E-08	0.00151383	0.455	0
WSB1	12	0.72639643	3.54E-09	6.45E-05	0.902	0.467
SGIP1	12	0.71958044	6.59E-09	0.00012017	0.509	0
CCDC85B	12	0.71509469	6.76E-07	0.01233031	0.92	0.578
FAP	12	0.71447957	1.97E-09	3.59E-05	0.58	0.044
PMP22	12	0.71114576	7.32E-08	0.00133495	0.902	0.533
PPP1R15A	12	0.70590033	2.19E-08	0.00039953	0.554	0.067
TNFRSF12A	12	0.70498164	8.90E-08	0.0016244	0.598	0.111
CSGALNACT2	12	0.70203613	2.68E-07	0.00489153	0.429	0
AIMP1	12	0.69760153	3.08E-09	5.62E-05	0.795	0.267
EIF5	12	0.68642823	6.40E-08	0.00116773	0.786	0.356
PGK1	12	0.67577413	1.72E-10	3.14E-06	0.973	0.956
NPR3	12	0.67562262	8.41E-07	0.01534631	0.429	0.022
ENO1	12	0.67270956	4.66E-11	8.50E-07	1	0.978
SBDS	12	0.67036838	4.83E-08	0.00088151	0.75	0.311
SNRNP27	12	0.6598612	1.21E-07	0.00221363	0.607	0.156
CRLF1	12	0.65260416	1.67E-06	0.03056338	0.491	0.089
MED21	12	0.65108152	2.78E-07	0.00506522	0.732	0.333
WDR45B	12	0.64841129	1.17E-08	0.00021273	0.607	0.111
ARPP19	12	0.63834583	4.05E-08	0.00073955	0.795	0.422
CCND1	12	0.63480063	8.89E-07	0.01621995	0.429	0.022

RBM39	12	0.63480063	7.05E-07	0.01285639	0.714	0.333
CRTAC1	12	0.63306301	7.81E-07	0.01425528	0.482	0.067
GOLT1B	12	0.62463251	1.63E-06	0.02967142	0.571	0.178
WDR33	12	0.62273665	1.12E-07	0.00204269	0.696	0.289
CCT8	12	0.61835216	5.97E-07	0.01089779	0.804	0.444
SQSTM1	12	0.61702104	7.12E-09	0.00012995	1	0.956
GPX1	12	0.61531541	1.05E-08	0.00019232	0.964	0.667
PDE4B	12	0.61512531	1.25E-06	0.02282425	0.473	0.067
DNAJB9	12	0.61039033	3.32E-07	0.00604994	0.884	0.533
XBP1	12	0.60834043	1.15E-07	0.00209559	0.884	0.422
SMS	12	0.60745043	7.61E-07	0.01389317	0.625	0.244
CTTN	12	0.59807194	2.31E-06	0.04214898	0.598	0.2
RELB	12	0.59450602	1.17E-07	0.0021286	0.518	0.044
SLC25A37	12	0.59224102	8.97E-08	0.00163632	0.902	0.533
RNMT	12	0.58840224	1.02E-06	0.01857144	0.634	0.222
CD68	12	0.58427285	6.37E-07	0.01162968	0.607	0.178
NBPF14	12	0.58035027	4.15E-07	0.00756878	0.473	0.044
EIF4A2	12	0.57997977	3.62E-12	6.61E-08	1	1
METAP2	12	0.57717076	1.57E-07	0.00286644	0.92	0.533
RAB1A	12	0.56831643	2.00E-06	0.03654263	0.857	0.556
COX7A2	12	0.56484204	2.12E-11	3.87E-07	1	0.978
ARF6	12	0.55690165	2.70E-06	0.04924246	0.679	0.289
CCDC174	12	0.55450069	5.52E-07	0.01006561	0.491	0.067
BTG3	12	0.5530203	1.48E-06	0.02698829	0.554	0.156
HMGA2	12	0.5285252	1.64E-06	0.02984975	0.384	0
SYF2	12	0.51908226	6.62E-07	0.01207499	0.955	0.667
NOL7	12	0.50077529	2.19E-06	0.03994288	0.786	0.378
FAM162A	12	0.49964823	1.12E-06	0.02043217	0.955	0.8
SPRY2	12	0.49643689	2.34E-06	0.04269209	0.375	0
GAPDH	12	0.4208271	2.57E-07	0.00469282	1	1
OAZ1	12	0.36599548	1.93E-06	0.03527826	1	0.978
AGT	12	-0.2537805	2.86E-07	0.00521978	0	0.222
MALL	12	-0.2612488	9.18E-07	0.01674849	0.027	0.289
RPS4X	12	-0.2722518	1.18E-07	0.00216012	1	1
RPL5	12	-0.2788938	5.30E-07	0.0096783	1	1
RPLP1	12	-0.2859181	1.45E-09	2.65E-05	1	1
RPL27A	12	-0.2867147	1.16E-08	0.00021155	1	1
KHDRBS3	12	-0.2876821	1.50E-08	0.00027329	0	0.267
RPL7A	12	-0.2884525	8.03E-07	0.01464503	1	1

RPL37	12	-0.2926375	8.65E-09	0.00015777	1	1
RPL22	12	-0.295628	3.04E-07	0.00555435	1	1
TXNIP	12	-0.3115829	6.30E-09	0.0001149	0.009	0.311
RPL37A	12	-0.3117796	1.45E-10	2.64E-06	1	1
RPL15	12	-0.3154444	4.09E-11	7.46E-07	1	1
ABHD14A	12	-0.3204719	3.37E-09	6.15E-05	0	0.289
RPS3	12	-0.3219582	1.68E-10	3.06E-06	1	1
RPL35A	12	-0.327089	1.79E-10	3.26E-06	1	1
RPL35	12	-0.3277368	2.73E-09	4.97E-05	1	1
RPL18A	12	-0.3312909	1.08E-09	1.97E-05	1	1
RPL29	12	-0.3343871	4.18E-08	0.00076292	1	1
RPL3	12	-0.3347264	1.16E-09	2.11E-05	1	1
RPL11	12	-0.3357421	2.68E-13	4.90E-09	1	1
RPL6	12	-0.3427255	4.70E-10	8.57E-06	1	1
CTSA	12	-0.3479009	4.44E-08	0.00081059	0.036	0.356
SMIM14	12	-0.3481473	1.61E-06	0.02937895	0.08	0.4
RPS23	12	-0.3518826	2.51E-10	4.57E-06	1	1
AC003090.1	12	-0.3522206	1.61E-10	2.95E-06	0	0.333
HOXB8	12	-0.3522206	7.49E-10	1.37E-05	0	0.311
RPN2	12	-0.3543551	1.86E-07	0.00339374	0.045	0.356
HTATIP2	12	-0.3629388	7.61E-10	1.39E-05	0.027	0.4
RPS27	12	-0.3654042	1.09E-14	1.99E-10	1	1
CXXC5	12	-0.3677248	3.40E-09	6.21E-05	0	0.289
RPL30	12	-0.370516	3.00E-10	5.47E-06	1	1
TOMM7	12	-0.3749991	5.15E-08	0.00093979	1	1
RPL13A	12	-0.3780493	8.42E-14	1.54E-09	1	1
RPS6	12	-0.3836302	7.37E-14	1.34E-09	1	1
NFIA	12	-0.3870245	1.05E-06	0.01916721	0.071	0.378
RPS18	12	-0.3896058	4.55E-13	8.31E-09	1	1
ENPP1	12	-0.3953928	2.33E-07	0.00424863	0.045	0.356
PTRF	12	-0.3960232	1.07E-06	0.01950224	0.143	0.511
RPS25	12	-0.3996719	1.66E-12	3.02E-08	1	1
C14orf1	12	-0.4065053	2.12E-06	0.03864507	0.08	0.378
RPL23	12	-0.4080038	1.98E-08	0.00036103	0.991	1
RPS3A	12	-0.4130777	6.97E-14	1.27E-09	1	1
RPL9	12	-0.4136332	3.07E-12	5.60E-08	1	1
PIGT	12	-0.4263286	2.95E-09	5.38E-05	0.036	0.4
RPL19	12	-0.4265985	1.04E-16	1.90E-12	1	1
RPL10	12	-0.4274643	7.61E-16	1.39E-11	1	1

RPL14	12	-0.4283128	7.56E-14	1.38E-09	1	1
RPS2	12	-0.4344979	1.51E-12	2.75E-08	1	1
RPS9	12	-0.4374649	1.17E-12	2.14E-08	1	1
RPS17	12	-0.439622	6.91E-11	1.26E-06	1	1
S100A13	12	-0.4397472	5.18E-08	0.00094498	0.973	1
EFNA1	12	-0.4403279	2.16E-06	0.03935588	0.143	0.489
GREM1	12	-0.4418328	6.66E-08	0.00121575	0	0.244
RPL13	12	-0.4428113	6.32E-16	1.15E-11	1	1
TMEM98	12	-0.4464731	4.06E-09	7.40E-05	0.107	0.556
RPS29	12	-0.4476599	1.27E-14	2.31E-10	1	1
PCBP2	12	-0.4478029	3.47E-07	0.0063398	0.804	0.978
COMMD6	12	-0.4481575	9.45E-08	0.00172373	1	1
RPL18	12	-0.4494705	3.14E-13	5.73E-09	1	1
ANXA2	12	-0.4505919	2.68E-06	0.04883063	0.804	0.978
RPS7	12	-0.4519411	1.13E-10	2.05E-06	1	1
SSR2	12	-0.4540033	1.33E-06	0.02434791	0.723	0.956
RPS5	12	-0.459356	1.59E-13	2.90E-09	1	1
RPS27A	12	-0.4599586	1.03E-16	1.88E-12	1	1
PNKD	12	-0.4641385	4.68E-08	0.00085308	0.161	0.578
FMOD	12	-0.4655493	5.74E-07	0.01046935	0.848	1
DYNLT1	12	-0.4674559	6.45E-08	0.00117756	0.116	0.511
RPS16	12	-0.4693864	9.54E-14	1.74E-09	1	1
SSPN	12	-0.4718851	9.83E-09	0.00017929	0.045	0.4
PRUNE2	12	-0.4735815	3.05E-07	0.00556709	0.08	0.422
RPL8	12	-0.479576	9.30E-15	1.70E-10	1	1
TCEB2	12	-0.4803343	4.42E-08	0.00080669	0.92	0.978
RPL34	12	-0.4847324	4.05E-16	7.38E-12	1	1
CST3	12	-0.4866468	4.16E-07	0.00759178	0.982	1
PRCP	12	-0.4894217	5.94E-08	0.00108429	0.062	0.4
GNAS	12	-0.4914679	5.55E-07	0.01012825	0.321	0.711
HMGN2	12	-0.4926411	5.81E-07	0.01061037	0.777	0.956
HNRNPA1	12	-0.4976376	1.44E-09	2.63E-05	0.991	1
ATRNL1	12	-0.4978606	3.41E-08	0.00062271	0.045	0.378
PIM3	12	-0.4997267	2.60E-07	0.00473654	0.214	0.622
IER2	12	-0.5022645	1.25E-07	0.00228733	0.08	0.422
SLC14A1	12	-0.5063713	4.82E-08	0.00087861	0.08	0.444
RPLP2	12	-0.5073379	4.37E-16	7.97E-12	1	1
FTL	12	-0.5086868	1.39E-12	2.53E-08	1	1
MT-ND4	12	-0.5209701	2.56E-06	0.04663177	0.946	0.978

PRELP	12	-0.5225321	2.52E-06	0.04602773	0.982	1
RPL7	12	-0.5239016	6.29E-16	1.15E-11	1	1
RABAC1	12	-0.5240709	1.98E-07	0.00360603	0.67	0.889
TPT1	12	-0.5248262	1.12E-14	2.05E-10	1	1
PRDX5	12	-0.5249686	3.01E-09	5.50E-05	0.795	0.978
DCN	12	-0.5273567	1.60E-10	2.93E-06	1	1
EEF1B2	12	-0.5290709	2.98E-09	5.44E-05	0.946	1
EEF2	12	-0.5293278	2.08E-06	0.03787782	0.991	1
RPL23A	12	-0.5309558	3.33E-19	6.08E-15	1	1
RPS13	12	-0.5349926	4.19E-16	7.65E-12	1	1
SEC11A	12	-0.5359915	1.29E-08	0.00023525	0.795	0.933
SARAF	12	-0.5382119	7.00E-07	0.01278123	0.339	0.689
C7orf73	12	-0.5389965	3.46E-07	0.00630633	0.384	0.8
FBXO2	12	-0.5406603	7.58E-09	0.00013828	0.598	0.933
INHBA	12	-0.5498564	3.38E-07	0.00616627	0.089	0.444
PEBP1	12	-0.5768612	2.52E-07	0.00459431	0.634	0.911
IFITM2	12	-0.5787578	1.57E-06	0.02862687	0.759	0.978
GAS6	12	-0.5851522	3.18E-10	5.80E-06	0.08	0.511
CTSD	12	-0.5910139	3.91E-07	0.00713005	0.732	0.956
TMED2	12	-0.5933827	7.36E-10	1.34E-05	0.616	0.933
RPS11	12	-0.5992023	2.15E-12	3.92E-08	1	1
SSR3	12	-0.6013891	2.44E-09	4.45E-05	0.804	0.956
MDFI	12	-0.6031009	3.28E-08	0.00059842	0.089	0.467
NEAT1	12	-0.6077766	3.26E-09	5.96E-05	0.991	1
CCNI	12	-0.6078214	1.84E-08	0.00033555	0.982	1
FCGRT	12	-0.608529	2.41E-09	4.39E-05	0.312	0.778
BSG	12	-0.6131392	4.28E-09	7.82E-05	0.473	0.867
TSPO	12	-0.613497	6.41E-10	1.17E-05	0.589	1
ID4	12	-0.6151045	1.71E-07	0.00312232	0.17	0.556
PRSS23	12	-0.6158555	3.03E-11	5.52E-07	0.071	0.533
PPIA	12	-0.6233268	2.01E-10	3.66E-06	0.893	1
ITM2C	12	-0.6241543	3.68E-08	0.00067204	0.179	0.578
LRPAP1	12	-0.6262752	6.95E-09	0.00012687	0.286	0.711
BRK1	12	-0.6322515	4.01E-12	7.32E-08	0.661	1
PABPC1	12	-0.6343585	5.62E-09	0.0001025	0.652	1
RNF130	12	-0.6344531	1.48E-09	2.71E-05	0.304	0.756
YBX1	12	-0.6396242	2.96E-13	5.41E-09	0.938	1
SNAI2	12	-0.6523252	1.04E-07	0.00190486	0.17	0.556
RPS8	12	-0.65537	1.54E-17	2.81E-13	1	1

KDELR2	12	-0.6562162	1.23E-09	2.25E-05	0.625	0.978
SLC40A1	12	-0.6617854	8.56E-14	1.56E-09	0.009	0.467
RPL4	12	-0.6639659	2.17E-13	3.96E-09	0.991	1
HLA-C	12	-0.6691265	3.02E-10	5.50E-06	0.938	1
NGFRAP1	12	-0.6706743	1.10E-10	2.00E-06	0.589	1
RPL10A	12	-0.671537	1.34E-16	2.44E-12	1	1
ATPIF1	12	-0.6726067	4.30E-08	0.00078444	0.438	0.756
NDUFA11	12	-0.6772204	9.31E-12	1.70E-07	0.705	0.978
DUSP1	12	-0.6820483	5.28E-08	0.0009637	0.223	0.622
PTMS	12	-0.6828277	1.64E-11	2.98E-07	0.295	0.844
SPON2	12	-0.6842582	2.20E-12	4.02E-08	0.009	0.422
RPS20	12	-0.6875184	1.82E-15	3.32E-11	1	1
RPLP0	12	-0.6877145	1.23E-18	2.24E-14	1	1
CCNG1	12	-0.6905994	2.76E-09	5.04E-05	0.17	0.578
HLA-B	12	-0.7019381	3.87E-10	7.07E-06	0.786	0.933
COL2A1	12	-0.7073047	1.60E-15	2.92E-11	0.268	0.933
POLR2L	12	-0.7083724	2.81E-12	5.13E-08	0.652	1
NPC2	12	-0.7119869	1.01E-13	1.84E-09	0.955	1
FXYD6	12	-0.7159619	1.09E-12	2.00E-08	0.482	0.956
EIF3H	12	-0.7174029	1.08E-12	1.97E-08	0.964	1
ITM2A	12	-0.7275899	5.06E-09	9.23E-05	0.491	0.822
EEF1A1	12	-0.7307085	1.12E-18	2.05E-14	1	1
ISLR	12	-0.7440429	5.68E-12	1.04E-07	0.098	0.578
C2orf40	12	-0.7462366	4.68E-11	8.55E-07	1	1
GRN	12	-0.7576857	3.53E-15	6.44E-11	0.045	0.6
MT-ND1	12	-0.7703368	1.96E-14	3.57E-10	0.179	0.8
S100B	12	-0.7777446	8.71E-12	1.59E-07	0.598	0.978
H3F3A	12	-0.7926114	1.99E-15	3.62E-11	0.866	1
HLA-A	12	-0.7950548	2.55E-11	4.66E-07	0.902	1
CRISPLD1	12	-0.7995691	1.66E-14	3.02E-10	0.009	0.489
CRTAP	12	-0.8064759	1.64E-11	2.99E-07	0.357	0.844
PDIA6	12	-0.8086521	9.41E-13	1.72E-08	0.277	0.8
COL11A1	12	-0.8104839	1.78E-14	3.25E-10	0.268	0.911
LECT1	12	-0.8200815	6.51E-10	1.19E-05	0.732	1
CTSC	12	-0.8216853	1.37E-09	2.51E-05	0.036	0.4
CITED2	12	-0.8412188	9.13E-14	1.67E-09	0.116	0.667
IGFBP6	12	-0.8434726	1.73E-07	0.00315268	0.482	0.844
RHOBTB3	12	-0.8438857	2.03E-13	3.70E-09	0.188	0.733
FOS	12	-0.849961	1.33E-09	2.42E-05	0.214	0.644

PNRC1	12	-0.8692768	2.49E-14	4.54E-10	0.866	1
P4HB	12	-0.8731842	1.47E-11	2.68E-07	0.482	0.933
RBP4	12	-0.8740961	3.10E-11	5.67E-07	0.527	0.911
ID2	12	-0.8972376	3.81E-11	6.96E-07	0.179	0.667
YBX3	12	-0.8982922	2.00E-12	3.64E-08	0.518	0.956
SPARC	12	-0.918854	4.74E-15	8.65E-11	0.152	0.778
ZFP36L2	12	-0.9353669	1.71E-13	3.12E-09	0.071	0.6
JUNB	12	-0.9728309	1.80E-12	3.28E-08	0.223	0.756
CD99	12	-0.9864472	5.62E-15	1.03E-10	0.768	0.978
COL9A3	12	-0.9942074	3.81E-13	6.95E-09	0.384	0.911
MT-ND2	12	-1.0109273	7.29E-17	1.33E-12	0.17	0.844
HIST1H1C	12	-1.0159766	2.93E-10	5.35E-06	0.232	0.711
CP	12	-1.0177287	9.17E-14	1.67E-09	0.366	0.867
CEBPD	12	-1.0243912	1.73E-14	3.16E-10	0.616	0.978
FTH1	12	-1.0265911	1.88E-19	3.42E-15	1	1
ID1	12	-1.030318	1.55E-16	2.83E-12	0.161	0.822
C2orf82	12	-1.0331468	1.18E-12	2.15E-08	0.929	1
EID1	12	-1.0389369	1.89E-17	3.44E-13	0.661	1
B2M	12	-1.0575482	4.73E-19	8.63E-15	0.991	1
MT-CO3	12	-1.0579002	1.85E-13	3.37E-09	0.777	0.978
MT-ATP6	12	-1.0605473	1.83E-14	3.35E-10	0.554	0.978
MT-ND3	12	-1.0659871	1.51E-20	2.76E-16	0.205	0.956
TSC22D1	12	-1.0659871	2.83E-13	5.16E-09	0.357	0.889
MT-CO1	12	-1.0919625	1.84E-12	3.36E-08	0.929	0.978
MT-CYB	12	-1.1046392	6.74E-15	1.23E-10	0.312	0.911
NNMT	12	-1.1119626	2.12E-17	3.87E-13	0.777	1
PAX1	12	-1.175127	1.81E-19	3.30E-15	0.33	0.956
SLPI	12	-1.1826446	5.05E-10	9.21E-06	0.92	1
ACAN	12	-1.1882824	4.89E-16	8.93E-12	0.75	1
CD9	12	-1.189262	8.38E-18	1.53E-13	0.241	0.867
ID3	12	-1.2623857	3.70E-22	6.75E-18	0.107	0.889
MGP	12	-1.2845456	6.35E-14	1.16E-09	1	1
CAPS	12	-1.3279968	1.65E-14	3.01E-10	0.473	0.978
GPX3	12	-1.3729246	8.49E-19	1.55E-14	0.821	1
ADIRF	12	-1.4650827	1.75E-15	3.19E-11	0.348	0.889
PLAC9	12	-1.5788724	1.40E-21	2.55E-17	0.357	1
MT-CO2	12	-1.5907806	4.13E-19	7.53E-15	0.812	1
CLEC3A	12	-1.6562769	9.46E-25	1.73E-20	0.107	0.956
OGN	12	-1.7588296	2.89E-18	5.27E-14	0.759	1

SPTSSB	12	-1.7791599	2.08E-28	3.80E-24	0.009	0.867
FRZB	12	-1.9401718	9.41E-19	1.72E-14	0.223	0.911
**gene**	**cluster**	**avg_logFC**	**p_val**	**p_val_adj**	**pct.1**	**pct.2**
TGFBI	13	1.22776807	2.91E-16	5.31E-12	0.897	0.216
SPP1	13	1.12172313	3.26E-10	5.96E-06	0.598	0.039
MT1X	13	1.08531551	3.86E-10	7.04E-06	1	0.922
MT2A	13	0.95630946	5.75E-12	1.05E-07	1	1
HMOX1	13	0.92440695	9.35E-08	0.00170629	0.619	0.176
NRP2	13	0.69645203	2.76E-08	0.00050343	0.938	0.608
FN1	13	0.63094089	1.58E-09	2.89E-05	1	1
GJA1	13	0.54369572	1.65E-06	0.03016796	0.515	0.118
SERPINE2	13	0.49209459	1.63E-06	0.02974397	1	1
PTGES	13	0.46188081	1.10E-06	0.02015457	0.433	0.039
MIF	13	0.30844324	1.70E-06	0.03104937	1	1
NPDC1	13	-0.3335641	2.43E-06	0.04434323	0.113	0.451
RPLP0	13	-0.3599503	1.14E-08	0.00020777	1	1
SUMO2	13	-0.3632613	2.88E-08	0.00052538	1	1
TPT1	13	-0.3843114	4.17E-08	0.0007601	1	1
CKAP4	13	-0.4059756	2.18E-06	0.03980482	0.639	1
ENAH	13	-0.4135753	2.19E-06	0.03994926	0.216	0.588
MT-ND4	13	-0.4153203	1.62E-06	0.0294743	0.959	0.941
B2M	13	-0.4184269	9.01E-07	0.01643954	1	1
RPL4	13	-0.4258208	7.01E-08	0.00127991	0.99	1
GPAA1	13	-0.4312362	2.67E-06	0.04873465	0.567	0.863
CSDE1	13	-0.4324808	2.36E-07	0.00431122	0.711	0.941
HNRNPA2B1	13	-0.4363168	1.98E-06	0.03608211	0.856	1
MOXD1	13	-0.4466054	2.65E-08	0.00048288	0.041	0.392
YBX1	13	-0.4544382	6.83E-08	0.00124552	1	1
S100A13	13	-0.4577692	3.81E-07	0.00694846	0.969	1
ITGB1BP1	13	-0.4813979	1.56E-06	0.02842178	0.742	0.902
SRP9	13	-0.4827999	2.41E-08	0.00043991	0.897	1
BCL7C	13	-0.484415	4.49E-07	0.00819876	0.691	0.922
AHNAK	13	-0.4857935	6.75E-07	0.01231405	0.67	0.961
RPS20	13	-0.4907098	5.97E-10	1.09E-05	1	1
CALM3	13	-0.4991538	5.15E-09	9.41E-05	0.887	0.98
AES	13	-0.5013858	7.95E-09	0.00014514	0.423	0.824
TPM3	13	-0.5033559	4.12E-07	0.00751944	0.67	0.941
RPS11	13	-0.5039599	7.98E-11	1.46E-06	0.979	1
MYL6	13	-0.5270122	8.35E-08	0.00152319	1	1

NGFRAP1	13	-0.5490192	8.36E-08	0.00152504	0.856	0.961
PABPC1	13	-0.5497949	1.35E-08	0.00024675	0.866	0.98
TMEM106C	13	-0.5886225	4.66E-09	8.49E-05	0.784	0.961
LMNA	13	-0.5903339	1.88E-07	0.00342412	0.794	0.961
CAV1	13	-0.617352	2.16E-06	0.03932262	0.845	0.98
HSPB1	13	-0.6241348	3.32E-08	0.00060493	0.876	1
EEF1A1	13	-0.6370148	4.86E-08	0.00088624	1	1
NFIC	13	-0.637636	4.94E-10	9.01E-06	0.608	0.882
ARPC1B	13	-0.6403885	3.55E-08	0.00064695	0.794	0.961
MT-ND3	13	-0.6428853	7.08E-08	0.00129197	0.691	0.882
MT-CO1	13	-0.6484255	1.86E-09	3.39E-05	0.938	0.922
PTMS	13	-0.6571917	5.69E-08	0.00103853	0.845	1
DYNLL1	13	-0.6647309	1.24E-08	0.00022592	0.979	0.98
SH3BGRL3	13	-0.6680482	8.74E-07	0.0159429	0.99	1
S100A10	13	-0.6694827	1.44E-10	2.63E-06	1	1
PRKCDBP	13	-0.6754808	3.69E-07	0.0067343	0.784	0.98
MT-ATP6	13	-0.6822384	3.25E-08	0.000593	0.907	0.941
MT-CO3	13	-0.6854108	1.05E-08	0.00019174	0.918	0.941
THY1	13	-0.686656	8.12E-08	0.00148179	0.68	0.961
S100A6	13	-0.7066888	2.91E-10	5.32E-06	1	1
PAX1	13	-0.7135029	9.14E-09	0.00016682	0.495	0.824
S100A1	13	-0.7572957	1.26E-06	0.02308039	0.804	0.941
MALL	13	-0.7595222	3.03E-09	5.53E-05	0.485	0.882
MT-ND1	13	-0.7786869	2.97E-10	5.42E-06	0.711	0.922
MT-CYB	13	-0.7920485	4.22E-09	7.69E-05	0.763	0.922
CAPS	13	-0.7952923	1.84E-06	0.03355371	0.34	0.706
FTH1	13	-0.7955116	2.08E-15	3.79E-11	1	1
PTRF	13	-0.7963952	7.31E-12	1.33E-07	0.732	0.961
IGFBP6	13	-0.805925	6.74E-08	0.00122998	0.526	0.824
MT-ND2	13	-0.8338153	2.85E-09	5.21E-05	0.763	0.922
SLC9A3R2	13	-0.8377251	3.88E-07	0.00708539	0.526	0.765
PLAC9	13	-0.8428371	1.03E-08	0.00018739	0.526	0.941
LOXL1	13	-0.8812964	2.61E-11	4.77E-07	0.258	0.745
CITED2	13	-0.8858315	1.27E-06	0.02314792	0.196	0.549
PRSS23	13	-1.1640738	2.55E-09	4.66E-05	0.701	0.902
ADIRF	13	-1.3739637	5.73E-16	1.05E-11	0.433	0.941
MSMP	13	-1.8072689	6.26E-10	1.14E-05	0.134	0.608
