# Supplementary material for: Single-Cell RNA-Seq Analysis of Cells from Degenerating and Non-Degenerating Intervertebral Discs from the Same Individual Reveals New Biomarkers for Intervertebral Disc Degeneration
Source: Int J Mol Sci. 2022 Apr 3;23(7):3993. doi: 10.3390/ijms23073993 (PMC8999935; doi:10.3390/ijms23073993)
Supplement: Supplementary file 1 [file ijms-23-03993-s001.zip › ijms-1655681-SI/Table S2.pdf]

Table S2. Cluster markers.

gene	cluster	avg_logFC	p_val	p_val_adj	pct.1	pct.2
SOD2	0	0.41950552	1.0047E-108	1.8332E-104	0.974	0.877
FRZB	0	0.40009756	2.05252E-29	3.74523E-25	0.578	0.499
CHI3L1	0	0.39754308	1.35121E-65	2.46556E-61	0.955	0.912
NPM1	0	0.32286647	3.8811E-205	7.0818E-201	1	0.995
DDIT4L	0	0.31757729	5.91416E-55	1.07916E-50	0.732	0.604
ZFAS1	0	0.30343857	8.9199E-202	1.6276E-197	0.999	0.983
EIF4A2	0	0.30287229	9.1187E-157	1.6639E-152	0.998	0.965
CIRBP	0	0.30091505	2.6584E-189	4.8508E-185	0.998	0.978
EPB41L4A-AS1	0	0.29815642	5.225E-134	9.534E-130	0.923	0.793
GADD45A	0	0.29716921	1.75641E-72	3.20492E-68	0.501	0.33
RSL24D1	0	0.29600174	3.9587E-163	7.2234E-159	0.989	0.93
SNHG12	0	0.28992616	3.50952E-81	6.40383E-77	0.544	0.366
EIF1	0	0.28367582	3.9354E-182	7.181E-178	1	1
ANP32B	0	0.27400809	5.9054E-118	1.0776E-113	0.949	0.86
RGS2	0	0.27175231	1.75784E-22	3.20754E-18	0.464	0.377
UBXN1	0	0.26530636	1.1859E-104	2.1639E-100	0.828	0.7
SYF2	0	0.26423483	7.1837E-105	1.3108E-100	0.882	0.751
EIF3E	0	0.2509564	9.1058E-117	1.6615E-112	0.999	0.988
**gene**	**cluster**	**avg_logFC**	**p_val**	**p_val_adj**	**pct.1**	**pct.2**
FGFBP2	1	0.79747726	0	0	0.995	0.911
CLEC3A	1	0.76001412	2.3064E-154	4.2084E-150	0.729	0.512
OGN	1	0.75482998	3.29E-237	6.0033E-233	0.951	0.838
C2orf40	1	0.65879817	3.5161E-219	6.4159E-215	0.997	0.96
SLPI	1	0.60405173	7.29848E-91	1.33175E-86	0.972	0.92
C2orf82	1	0.59792332	2.2595E-191	4.1229E-187	0.991	0.945
PRELP	1	0.58609273	1.8571E-241	3.3886E-237	0.986	0.914
LECT1	1	0.52414331	1.6686E-198	3.0446E-194	0.922	0.748
APOD	1	0.48160145	2.18869E-73	3.99371E-69	0.501	0.333
IGFBP6	1	0.46993258	7.38664E-97	1.34784E-92	0.78	0.62
RBP4	1	0.4622583	5.4443E-126	9.9343E-122	0.868	0.712
TSC22D1	1	0.43258866	7.79953E-67	1.42318E-62	0.796	0.707
MIA	1	0.42535463	4.707E-205	8.5889E-201	0.996	0.954
CST3	1	0.39576885	2.9146E-192	5.3183E-188	0.998	0.986
ITM2A	1	0.38799962	5.1138E-120	9.3311E-116	0.702	0.489

MGP	1	0.36442408	1.3731E-82	2.5055E-78	0.998	0.995
IVNS1ABP	1	0.33443427	2.39923E-94	4.37788E-90	0.843	0.708
FMOD	1	0.33121741	3.9432E-131	7.1951E-127	0.965	0.873
S100B	1	0.30855962	1.0303E-85	1.87999E-81	0.86	0.727
ISLR	1	0.30793285	3.06309E-62	5.58922E-58	0.601	0.451
CRISPLD1	1	0.30323379	4.83979E-38	8.83117E-34	0.36	0.242
SERPINA5	1	0.30207559	2.29492E-89	4.18753E-85	0.686	0.497
SLC40A1	1	0.29988706	1.43507E-57	2.61858E-53	0.415	0.261
TMEM59	1	0.29506021	8.765E-129	1.5994E-124	0.997	0.966
DCN	1	0.29038518	2.1265E-117	3.8803E-113	1	0.997
ITM2C	1	0.28591766	2.93626E-70	5.35779E-66	0.682	0.529
SLC14A1	1	0.28122229	4.75561E-60	8.67757E-56	0.505	0.353
PAX1	1	0.26870844	6.65891E-45	1.21505E-40	0.737	0.651
SSR4	1	0.26715138	1.5519E-108	2.8317E-104	0.984	0.954
CAPS	1	0.2667153	1.23976E-58	2.26218E-54	0.847	0.723
XAGE2	1	0.25727565	1.50845E-17	2.75247E-13	0.149	0.092
PRDX4	1	0.25448151	2.95974E-85	5.40065E-81	0.867	0.742
SCRG1	1	0.25248237	1.2433E-99	2.2686E-95	0.998	0.971
HLA-C	1	0.25221995	2.70276E-66	4.93173E-62	0.926	0.863
CCDC80	1	0.2517931	1.83052E-42	3.34015E-38	0.954	0.895
**gene**	**cluster**	**avg_logFC**	**p_val**	**p_val_adj**	**pct.1**	**pct.2**
MT1G	2	1.69951687	0	0	0.999	0.892
MT1H	2	1.52952008	0	0	0.955	0.589
MT1F	2	1.27147422	0	0	0.989	0.852
MT1X	2	1.22879532	0	0	1	0.992
MT1E	2	1.05954523	0	0	1	0.961
MT2A	2	0.83331624	1.2184E-255	2.2232E-251	1	0.995
MT1M	2	0.59045262	2.3735E-127	4.331E-123	0.734	0.487
**gene**	**cluster**	**avg_logFC**	**p_val**	**p_val_adj**	**pct.1**	**pct.2**
CXCL8	3	1.4626932	7.32048E-74	1.33577E-69	0.201	0.065
G0S2	3	1.46055786	5.4926E-122	1.0022E-117	0.482	0.23
C15orf48	3	1.24588459	0	0	0.415	0.078
SOD2	3	1.23316654	0	0	0.998	0.885
CCL20	3	1.04476805	7.51E-91	1.37E-86	0.147	0.03
MT1H	3	1.0244411	1.2022E-165	2.1937E-161	0.855	0.612
MMP3	3	1.00119489	1.6321E-178	2.9781E-174	0.914	0.73
MT1G	3	0.97929123	7.7395E-180	1.4122E-175	0.983	0.897
MT2A	3	0.96230984	3.5627E-257	6.5008E-253	1	0.996
WTAP	3	0.9343821	7.91E-197	1.44E-192	0.898	0.669

NFKBIA	3	0.82866962	7.623E-138	1.391E-133	0.549	0.265
LCN2	3	0.79270775	1.93611E-57	3.53283E-53	0.611	0.423
MT1X	3	0.77746521	4.8047E-160	8.7671E-156	1	0.993
IER3	3	0.70657222	3.46E-118	6.31E-114	0.505	0.24
SERPINE2	3	0.69686501	1.4749E-130	2.6912E-126	0.998	0.995
MT1E	3	0.69244666	6.3724E-148	1.1628E-143	0.994	0.963
SLC7A2	3	0.69077725	2.3744E-286	4.3325E-282	0.474	0.114
MT1M	3	0.67955764	1.9433E-122	3.546E-118	0.743	0.495
SPP1	3	0.64450646	7.21225E-31	1.31602E-26	0.543	0.409
CHI3L2	3	0.63357387	2.02325E-81	3.69183E-77	0.77	0.567
MT1F	3	0.6068355	5.1513E-99	9.39959E-95	0.952	0.861
RGS16	3	0.55349928	1.35363E-56	2.46997E-52	0.356	0.185
FTH1	3	0.54950742	1.55E-82	2.82E-78	1	1
CHI3L1	3	0.54245071	1.18E-69	2.15E-65	0.944	0.918
SLC39A14	3	0.53895805	1.96E-177	3.58E-173	0.827	0.529
ORM1	3	0.47440949	2.23E-23	4.07E-19	0.334	0.218
CXCL2	3	0.46416551	1.94E-94	3.53E-90	0.123	0.02
BMP2	3	0.46091826	4.58E-78	8.37E-74	0.433	0.213
HMGA1	3	0.45185235	3.65E-71	6.66E-67	0.603	0.368
CD44	3	0.43933918	4.16E-115	7.59E-111	0.928	0.76
PTX3	3	0.43586925	1.71E-165	3.12E-161	0.249	0.049
IL32	3	0.43193498	8.73E-190	1.59E-185	0.374	0.097
CTSL	3	0.42257873	8.63E-39	1.57E-34	0.7	0.562
PRG4	3	0.3978813	1.02E-11	1.87E-07	0.393	0.31
SLC25A37	3	0.39759585	6.71E-91	1.22E-86	0.906	0.739
HILPDA	3	0.39545925	4.09E-33	7.46E-29	0.577	0.446
C11orf96	3	0.38912957	3.36E-57	6.14E-53	0.391	0.203
RGS2	3	0.38676098	7.31E-37	1.33E-32	0.546	0.378
SOD3	3	0.38648673	2.45E-59	4.48E-55	0.959	0.916
TNFRSF11B	3	0.37551323	1.05E-10	1.91E-06	0.642	0.596
TFPI2	3	0.37079515	3.09E-30	5.64E-26	0.124	0.049
CLU	3	0.36224558	3.75E-55	6.85E-51	0.998	0.994
PDE4B	3	0.35158228	7.78E-92	1.42E-87	0.492	0.241
TNFAIP6	3	0.35152827	4.13E-47	7.54E-43	0.18	0.069
ZFP36L1	3	0.34683146	3.55E-45	6.47E-41	0.534	0.354
AKR1C2	3	0.34584111	1.04E-75	1.89E-71	0.343	0.149
TYMP	3	0.34487515	1.71E-84	3.12E-80	0.534	0.29
RELB	3	0.34198775	2.00E-105	3.65E-101	0.491	0.222

SERPINA1	3	0.33905264	3.68E-41	6.72E-37	0.961	0.923
NRP2	3	0.33668498	2.28E-44	4.16E-40	0.471	0.293
PNRC1	3	0.33148668	2.41E-65	4.40E-61	0.98	0.943
FN1	3	0.32953252	1.30E-58	2.37E-54	0.998	0.997
NAMPT	3	0.32723697	2.56E-55	4.68E-51	0.656	0.47
TNFAIP8	3	0.30755512	6.69E-144	1.22E-139	0.254	0.058
ASS1	3	0.30416213	2.65E-64	4.83E-60	0.565	0.354
DEFB1	3	0.30188342	2.73E-56	4.99E-52	0.308	0.143
GSTO1	3	0.29863321	4.82E-53	8.79E-49	0.791	0.653
GADD45A	3	0.28593966	1.29E-18	2.36E-14	0.456	0.353
UPP1	3	0.28410682	1.91E-35	3.48E-31	0.531	0.367
PDPN	3	0.27829784	2.41E-53	4.40E-49	0.673	0.485
BTG1	3	0.27695744	1.01E-39	1.84E-35	0.98	0.948
CEBPD	3	0.27525052	1.91E-33	3.48E-29	0.854	0.761
ICAM1	3	0.26725478	8.39E-53	1.53E-48	0.363	0.191
FNIP2	3	0.26664276	4.83E-56	8.82E-52	0.442	0.249
TM4SF1	3	0.26515629	1.93E-29	3.52E-25	0.644	0.502
SQRDL	3	0.26304701	3.99E-92	7.28E-88	0.409	0.177
MGST1	3	0.25876149	3.81E-30	6.96E-26	0.418	0.28
AKR1C1	3	0.25809841	6.58E-70	1.20E-65	0.29	0.117
RAB13	3	0.25583832	7.25E-49	1.32E-44	0.904	0.821
BIRC3	3	0.25574767	8.23E-138	1.50E-133	0.213	0.042
ORM2	3	0.25541268	1.72E-15	3.14E-11	0.166	0.097
ISG20	3	0.25488592	1.18E-41	2.15E-37	0.602	0.426
TREM1	3	0.25435389	6.05E-40	1.10E-35	0.337	0.186
LPP	3	0.25049184	1.04E-38	1.91E-34	0.595	0.426
**gene**	**cluster**	**avg_logFC**	**p_val**	**p_val_adj**	**pct.1**	**pct.2**
CTGF	4	1.58128898	0	0	0.917	0.419
ID1	4	0.87020271	8.8104E-178	1.6076E-173	0.891	0.581
CYR61	4	0.87000174	4.3551E-246	7.9467E-242	0.824	0.388
COL2A1	4	0.79178812	2.943E-142	5.3702E-138	0.902	0.662
COL11A1	4	0.75061128	1.5774E-171	2.8782E-167	0.936	0.704
THY1	4	0.69764592	1.382E-193	2.5218E-189	0.798	0.399
ID3	4	0.62530988	1.8725E-116	3.4168E-112	0.848	0.55
COL5A2	4	0.62173156	6.8388E-170	1.2479E-165	0.774	0.382
TPM1	4	0.60806303	9.147E-109	1.6691E-104	0.823	0.599
PLPP1	4	0.60324151	1.1455E-171	2.0902E-167	0.615	0.251
SCRG1	4	0.59422495	2.3368E-128	4.264E-124	0.998	0.974
CRYAB	4	0.58439778	7.749E-104	1.414E-99	0.91	0.746

FHL1	4	0.58275177	4.518E-119	8.2441E-115	0.71	0.413
DNM3OS	4	0.56608575	7.5568E-158	1.3789E-153	0.702	0.339
TUBB2B	4	0.54877542	5.74018E-70	1.04741E-65	0.629	0.402
TBL1XR1	4	0.54337376	9.0458E-139	1.6506E-134	0.669	0.35
POLR2L	4	0.52513865	1.2409E-174	2.2643E-170	0.985	0.847
LINC01503	4	0.51740382	8.464E-111	1.5444E-106	0.599	0.303
INHBA	4	0.508776	1.9655E-130	3.5864E-126	0.707	0.362
LOX	4	0.49449279	1.5816E-151	2.8859E-147	0.578	0.229
LINC00152	4	0.48638695	3.81994E-79	6.97025E-75	0.603	0.336
SERF2	4	0.47928412	2.1463E-181	3.9164E-177	1	1
ASPN	4	0.47473956	2.64506E-40	4.82644E-36	0.26	0.121
PLAC9	4	0.47377092	9.64172E-97	1.75932E-92	0.945	0.79
OGN	4	0.47361018	7.77557E-94	1.41881E-89	0.987	0.846
CAPS	4	0.47039227	4.15373E-52	7.57931E-48	0.867	0.734
MT-CO3	4	0.46905232	8.1843E-109	1.4934E-104	0.971	0.881
MT-CYB	4	0.45569602	1.51825E-85	2.77035E-81	0.912	0.707
MT-CO1	4	0.44635357	2.17652E-89	3.9715E-85	0.977	0.942
NEAT1	4	0.44504986	1.48351E-84	2.70697E-80	0.998	0.99
MT-CO2	4	0.44388738	2.76366E-85	5.04285E-81	0.982	0.926
ZFP36L1	4	0.44117437	9.91382E-60	1.80898E-55	0.576	0.353
GPX3	4	0.43869069	2.43363E-96	4.44064E-92	0.99	0.924
LSP1	4	0.43536376	5.3464E-100	9.75564E-96	0.624	0.319
PPIC	4	0.43375475	3.6238E-100	6.61228E-96	0.754	0.453
ID4	4	0.43115588	1.81688E-76	3.31526E-72	0.769	0.528
OSBPL9	4	0.42843828	1.04E-121	1.8977E-117	0.525	0.23
VCAN	4	0.41767743	4.32478E-67	7.89142E-63	0.667	0.419
COL3A1	4	0.41652804	9.2355E-126	1.6852E-121	0.526	0.209
MT-ATP6	4	0.40987296	6.78817E-78	1.23864E-73	0.947	0.806
DOK1	4	0.40875533	4.1789E-100	7.62532E-96	0.435	0.177
SLC40A1	4	0.4068712	3.53443E-42	6.44927E-38	0.462	0.273
SAA1	4	0.40073287	1.39086E-28	2.5379E-24	0.145	0.059
MT-ND3	4	0.39695377	1.08439E-73	1.97869E-69	0.899	0.682
CITED2	4	0.38489483	2.64889E-54	4.83343E-50	0.639	0.414
GOLIM4	4	0.38339131	2.51006E-36	4.5801E-32	0.599	0.434
S100A4	4	0.38116771	3.60042E-64	6.56968E-60	0.899	0.764
NNMT	4	0.37320527	1.13037E-81	2.06258E-77	0.959	0.882
CYP1B1	4	0.37254787	7.32361E-37	1.33634E-32	0.28	0.14
TCF4	4	0.35807884	9.6418E-69	1.75934E-64	0.683	0.431
HCFC1R1	4	0.35326198	9.56338E-74	1.74503E-69	0.857	0.657

COL11A2	4	0.35101594	3.24699E-55	5.92478E-51	0.686	0.487
SPTLC3	4	0.34890987	2.49256E-67	4.54818E-63	0.413	0.194
GAS1	4	0.34698853	2.78302E-84	5.07818E-80	0.438	0.191
MT-ND1	4	0.34194764	3.20393E-63	5.84622E-59	0.845	0.633
CXXC5	4	0.33942892	4.72782E-82	8.62685E-78	0.605	0.332
MAGED1	4	0.33794923	1.73136E-55	3.15921E-51	0.459	0.241
VKORC1	4	0.33414303	3.91996E-77	7.15274E-73	0.987	0.947
NREP	4	0.33301544	5.0379E-102	9.19267E-98	0.528	0.235
DSEL	4	0.33019583	6.05068E-65	1.10407E-60	0.377	0.173
MDFI	4	0.33017686	1.15366E-61	2.10508E-57	0.729	0.489
ENAH	4	0.3292482	2.11771E-75	3.86419E-71	0.562	0.309
SNAI2	4	0.32367929	1.51069E-45	2.75656E-41	0.695	0.493
AEBP1	4	0.32285777	6.70008E-47	1.22256E-42	0.768	0.589
MT-ND4	4	0.31806521	4.9716E-59	9.07167E-55	0.984	0.942
FSTL1	4	0.31534361	1.20422E-64	2.19735E-60	0.577	0.333
TRPS1	4	0.31519533	5.24778E-60	9.57562E-56	0.668	0.44
C7orf73	4	0.31227847	1.60691E-59	2.93214E-55	0.893	0.735
MT-ND2	4	0.31101644	3.29294E-51	6.00862E-47	0.853	0.658
BGN	4	0.31026426	5.57685E-71	1.01761E-66	1	0.983
SDC2	4	0.3093131	1.74083E-49	3.17648E-45	0.925	0.821
IFITM3	4	0.30844146	5.59386E-58	1.02071E-53	0.943	0.865
ACAN	4	0.30827545	8.4116E-52	1.53486E-47	0.978	0.914
SLC38A1	4	0.30598791	8.67708E-79	1.58331E-74	0.547	0.283
ID2	4	0.29953359	1.80947E-30	3.30173E-26	0.713	0.559
TMEM230	4	0.29877164	4.71992E-59	8.61244E-55	0.86	0.7
NGFRAP1	4	0.29780861	1.79975E-57	3.284E-53	0.923	0.837
THBS1	4	0.29681217	1.96773E-74	3.59051E-70	0.268	0.092
SPARC	4	0.29364709	1.01818E-76	1.85787E-72	0.846	0.573
FBXO2	4	0.29326136	8.28897E-38	1.51249E-33	0.851	0.732
RPL3	4	0.29200474	7.307E-134	1.3333E-129	1	1
FXYD6	4	0.29129162	3.76807E-58	6.8756E-54	0.923	0.801
C1orf122	4	0.29112989	7.66101E-62	1.39791E-57	0.621	0.388
ECM2	4	0.29110865	2.09765E-84	3.82759E-80	0.461	0.211
EPYC	4	0.29061004	1.66914E-39	3.04567E-35	0.314	0.159
IFITM2	4	0.28751395	2.69908E-50	4.92501E-46	0.954	0.86
NME4	4	0.28669776	2.82864E-61	5.16142E-57	0.673	0.432
IFITM10	4	0.28511216	1.16125E-73	2.11892E-69	0.44	0.205
RGCC	4	0.28197323	6.67839E-12	1.21861E-07	0.802	0.761
NDUFA4L2	4	0.28187944	3.96994E-96	7.24395E-92	1	0.999

CRIP1	4	0.28102723	1.07248E-29	1.95696E-25	0.481	0.328
HIF1A-AS2	4	0.28101174	6.07208E-46	1.10797E-41	0.398	0.22
TTC3	4	0.28063306	1.50416E-52	2.74465E-48	0.858	0.687
PDGFRA	4	0.27516628	2.12494E-72	3.87738E-68	0.505	0.252
AFF3	4	0.27359432	2.36601E-86	4.31726E-82	0.389	0.155
CP	4	0.27335953	9.83288E-28	1.79421E-23	0.8	0.674
MIR4435-2HG	4	0.27093079	2.63831E-47	4.81412E-43	0.411	0.22
FMOD	4	0.27092594	1.17976E-55	2.15271E-51	0.973	0.881
CCDC102B	4	0.27022565	2.10404E-52	3.83925E-48	0.414	0.215
ZIC1	4	0.26858098	5.68915E-46	1.0381E-41	0.391	0.211
P3H2	4	0.26781108	5.04169E-47	9.19957E-43	0.439	0.25
MEG3	4	0.26732739	1.57999E-41	2.88301E-37	0.468	0.284
GTF2H5	4	0.26377577	6.27413E-45	1.14484E-40	0.767	0.601
NFIA	4	0.26360739	1.18508E-45	2.16242E-41	0.548	0.35
ISM1	4	0.26018333	1.79575E-75	3.27671E-71	0.361	0.148
OST4	4	0.25958021	8.15363E-84	1.48779E-79	1	0.999
COL1A2	4	0.25765358	3.126E-55	5.70402E-51	0.488	0.265
COPZ2	4	0.25758213	5.87281E-64	1.07161E-59	0.51	0.269
NDUFA11	4	0.255039	3.14921E-51	5.74636E-47	0.951	0.874
PTRF	4	0.25190765	7.0037E-51	1.27797E-46	0.709	0.484
SOX9	4	0.25177593	5.00679E-33	9.13589E-29	0.807	0.655
ATRNL1	4	0.25027477	2.70221E-47	4.93073E-43	0.454	0.257
**gene**	**cluster**	**avg_logFC**	**p_val**	**p_val_adj**	**pct.1**	**pct.2**
FRZB	5	1.56279282	2.8104E-238	5.1281E-234	0.858	0.488
PLA2G2A	5	1.3097467	5.8056E-155	1.0593E-150	0.707	0.38
SLPI	5	0.83595956	1.1088E-103	2.0233E-99	0.994	0.923
CHRDL2	5	0.69490397	6.3249E-133	1.1541E-128	0.506	0.195
SPTSSB	5	0.60214803	2.66089E-46	4.85533E-42	0.508	0.318
GREM1	5	0.54642056	8.3913E-68	1.53116E-63	0.303	0.116
CFH	5	0.50259621	2.58536E-37	4.71751E-33	0.355	0.202
CLEC3A	5	0.46235042	1.92511E-41	3.51274E-37	0.723	0.535
C2orf82	5	0.44634631	6.77795E-66	1.23677E-61	0.99	0.95
COL9A3	5	0.40388391	3.54558E-49	6.46962E-45	0.8	0.618
SCRG1	5	0.39569469	3.422E-56	6.24412E-52	0.996	0.974
NOVA1	5	0.37963473	2.31629E-39	4.22653E-35	0.904	0.81
ACAN	5	0.35851945	6.31899E-64	1.15303E-59	0.979	0.914
ORM1	5	0.34750103	1.01735E-16	1.85636E-12	0.332	0.221
SERPINA1	5	0.33912073	1.8478E-59	3.37168E-55	0.992	0.921
CRISPLD1	5	0.33078318	1.58359E-36	2.88958E-32	0.427	0.25

S100B	5	0.30060849	5.22282E-40	9.53007E-36	0.88	0.74
FGFBP2	5	0.29218344	1.37427E-34	2.50762E-30	0.975	0.922
PCOLCE2	5	0.28708951	6.27813E-52	1.14557E-47	0.97	0.9
HAPLN1	5	0.28123126	3.78943E-25	6.91457E-21	0.745	0.621
CCDC80	5	0.27942848	1.02515E-33	1.8706E-29	0.97	0.9
BGN	5	0.27577888	1.63212E-50	2.97813E-46	0.999	0.983
RGS2	5	0.27530539	9.57721E-10	1.74755E-05	0.478	0.387
CHI3L2	5	0.26156108	6.68202E-21	1.21927E-16	0.699	0.577
FABP3	5	0.26023926	4.49488E-31	8.2018E-27	0.412	0.258
**gene**	**cluster**	**avg_logFC**	**p_val**	**p_val_adj**	**pct.1**	**pct.2**
MSMP	6	1.74404499	2.28257E-41	4.16501E-37	0.443	0.269
S100A2	6	1.37933196	3.22313E-88	5.88124E-84	0.705	0.458
TNFRSF11B	6	0.97692481	5.1169E-106	9.3368E-102	0.819	0.586
FABP5	6	0.96444394	3.01579E-80	5.50291E-76	0.606	0.35
TNFRSF12A	6	0.93137415	1.4511E-189	2.6478E-185	0.798	0.392
SERPINE2	6	0.78731604	3.1995E-117	5.8381E-113	1	0.995
IL11	6	0.77765637	3.72576E-97	6.7984E-93	0.281	0.075
CTSL	6	0.76978959	6.13271E-69	1.11904E-64	0.763	0.563
IGFBP3	6	0.75002368	1.05075E-62	1.91731E-58	0.545	0.299
PHLDA1	6	0.71450323	2.17192E-77	3.9631E-73	0.462	0.207
TM4SF1	6	0.71307171	1.49467E-86	2.72732E-82	0.762	0.5
HMGA1	6	0.6835974	3.2904E-102	6.00397E-98	0.682	0.371
MRPS6	6	0.6835815	3.51383E-74	6.41168E-70	0.963	0.909
BMP2	6	0.6833891	4.244E-103	7.744E-99	0.518	0.215
MT2A	6	0.64304393	5.13708E-66	9.37364E-62	0.999	0.996
FOSL1	6	0.63869997	4.61705E-55	8.42474E-51	0.386	0.179
STK17A	6	0.63849981	2.1231E-105	3.874E-101	0.653	0.335
SPP1	6	0.59212667	8.35669E-28	1.52485E-23	0.586	0.411
SERPINE1	6	0.57759464	4.2478E-115	7.751E-111	0.437	0.147
S100A6	6	0.55603441	6.1436E-143	1.121E-138	1	1
SLC5A3	6	0.54830395	3.68966E-65	6.73252E-61	0.701	0.48
PMP22	6	0.51601792	4.3156E-62	7.87468E-58	0.857	0.72
SMOX	6	0.50733444	9.24281E-64	1.68654E-59	0.554	0.307
RGCC	6	0.50587159	7.58671E-53	1.38435E-48	0.888	0.756
CCDC85B	6	0.49461925	4.13831E-45	7.55117E-41	0.878	0.792
CCND1	6	0.47824632	6.99079E-92	1.27561E-87	0.567	0.263
SPRY2	6	0.47812607	2.20291E-93	4.01965E-89	0.482	0.202
NT5E	6	0.47253627	1.7269E-107	3.1511E-103	0.678	0.346
UPP1	6	0.46555771	2.10401E-56	3.83918E-52	0.606	0.368

EMP3	6	0.44585284	3.93525E-96	7.18065E-92	0.975	0.869
C11orf96	6	0.43828435	1.22492E-51	2.23512E-47	0.423	0.207
SH3BGRL3	6	0.43774365	2.2793E-110	4.1591E-106	1	0.977
BZW1	6	0.42402774	2.08022E-61	3.79577E-57	0.81	0.611
VEGFA	6	0.42332293	1.60931E-45	2.93651E-41	0.755	0.575
TGFBI	6	0.42317306	1.78413E-20	3.25551E-16	0.541	0.408
KCNMA1	6	0.41452592	3.41248E-25	6.22675E-21	0.675	0.545
BAALC	6	0.40893467	2.16133E-43	3.94377E-39	0.625	0.434
CAV1	6	0.39726862	1.24136E-36	2.26511E-32	0.842	0.722
ISG20	6	0.3967095	1.0359E-59	1.8902E-55	0.664	0.428
LINC00152	6	0.39452935	7.58151E-64	1.3834E-59	0.603	0.341
S100A4	6	0.39443562	5.10676E-21	9.3183E-17	0.836	0.771
NDRG1	6	0.39115474	5.62447E-43	1.0263E-38	0.696	0.51
S100A16	6	0.38694388	6.50491E-53	1.18695E-48	0.567	0.345
ANXA2	6	0.38196224	2.93021E-69	5.34676E-65	0.941	0.85
IER3	6	0.37928053	5.12549E-27	9.35248E-23	0.417	0.255
TMSB10	6	0.37185209	9.19939E-74	1.67861E-69	0.998	0.988
ERRFI1	6	0.36479589	3.51898E-34	6.42109E-30	0.444	0.268
PLAUR	6	0.36238074	2.53151E-48	4.61925E-44	0.525	0.314
EIF4EBP1	6	0.35937931	1.62044E-43	2.95682E-39	0.723	0.529
CTHRC1	6	0.35123167	1.31849E-35	2.40584E-31	0.459	0.269
S100A10	6	0.33892706	4.80637E-60	8.77018E-56	0.99	0.98
SLC20A1	6	0.33424497	5.18941E-54	9.46912E-50	0.461	0.234
S100A11	6	0.31582563	9.95422E-54	1.81635E-49	0.977	0.915
ANXA5	6	0.31523081	5.46451E-47	9.9711E-43	0.917	0.818
GEM	6	0.3099186	8.91905E-31	1.62746E-26	0.427	0.261
AXL	6	0.30825086	3.08531E-34	5.62977E-30	0.518	0.332
ADSS	6	0.29793022	7.97348E-36	1.45492E-31	0.638	0.463
ANGPTL4	6	0.29681461	4.18433E-18	7.63514E-14	0.33	0.215
ARRDC3	6	0.29666147	1.59509E-21	2.91057E-17	0.52	0.382
FHL2	6	0.29622419	8.14414E-60	1.48606E-55	0.409	0.184
CD55	6	0.29584185	3.09013E-25	5.63856E-21	0.717	0.574
RHOC	6	0.29552676	2.80332E-41	5.11523E-37	0.872	0.779
RTN4	6	0.29522486	3.44315E-40	6.28272E-36	0.8	0.63
EPS8	6	0.29135562	3.45312E-44	6.30092E-40	0.493	0.286
PTGS2	6	0.28237306	2.33679E-27	4.26395E-23	0.509	0.326
ERO1A	6	0.27810001	3.51345E-28	6.41099E-24	0.675	0.528
TIMP3	6	0.27525133	7.09216E-11	1.29411E-06	0.59	0.51
BNIP3	6	0.2749844	1.7187E-32	3.13611E-28	0.993	0.992

PPFIBP1	6	0.27391615	6.2043E-32	1.1321E-27	0.625	0.445
TNFSF11	6	0.27369928	1.65673E-50	3.02304E-46	0.224	0.077
MGLL	6	0.26602211	5.51483E-52	1.00629E-47	0.343	0.15
SLC16A3	6	0.26355867	3.85555E-21	7.03523E-17	0.595	0.467
HMGA2	6	0.26207508	5.19006E-66	9.4703E-62	0.352	0.135
C10orf10	6	0.26176018	9.39549E-10	1.71439E-05	0.563	0.484
TMSB4X	6	0.26159748	1.35061E-10	2.46445E-06	0.828	0.768
NRN1	6	0.25691095	1.11872E-07	0.002041323	0.542	0.457
ENO2	6	0.25493247	2.78692E-26	5.08528E-22	0.6	0.44
PDLIM4	6	0.25309299	7.41992E-30	1.35391E-25	0.885	0.779
INSIG2	6	0.25217498	5.80728E-20	1.05965E-15	0.629	0.511
SDC4	6	0.25147989	5.09667E-12	9.29989E-08	0.451	0.351
MIR4435-2HG	6	0.25135575	6.66795E-41	1.2167E-36	0.418	0.223
**gene**	**cluster**	**avg_logFC**	**p_val**	**p_val_adj**	**pct.1**	**pct.2**
TMSB4X	7	1.71692251	0.00E+00	0.00E+00	0.992	0.759
COL1A2	7	1.62948271	0.00E+00	0.00E+00	0.872	0.248
CYTL1	7	1.56125071	4.38E-168	8.00E-164	0.559	0.181
LGALS1	7	1.37045979	0.00E+00	0.00E+00	0.999	0.972
S100A4	7	1.32958057	6.06E-234	1.11E-229	0.972	0.763
TMSB10	7	1.30728631	7.02E-302	1.28E-297	1	0.988
COL5A2	7	1.27044713	0.00E+00	0.00E+00	0.933	0.382
MSMP	7	1.25822043	2.03E-42	3.70E-38	0.46	0.269
COL1A1	7	1.23734039	0.00E+00	0.00E+00	0.392	0.04
THY1	7	1.21056595	7.28E-281	1.33E-276	0.917	0.401
COL3A1	7	1.18938001	4.94E-308	9.01E-304	0.752	0.204
CTGF	7	1.14900802	1.93E-94	3.53E-90	0.735	0.441
ARPC1B	7	1.11938994	2.82E-265	5.15E-261	0.898	0.455
SERF2	7	1.10153954	0.00E+00	0.00E+00	1	1
PRSS23	7	1.04516338	1.85E-217	3.38E-213	0.785	0.323
COL6A3	7	1.04000462	0.00E+00	0.00E+00	0.887	0.32
SH3BGRL3	7	1.02465919	3.89E-233	7.10E-229	0.997	0.977
LSP1	7	1.00977371	0.00E+00	0.00E+00	0.883	0.312
POLR2L	7	0.99462212	2.27E-231	4.15E-227	0.981	0.85
VIM	7	0.97771895	9.09E-90	1.66E-85	1	0.999
COL6A1	7	0.95995546	7.85E-286	1.43E-281	0.931	0.452
CRIP1	7	0.95243343	4.27E-222	7.78E-218	0.786	0.314
SPARC	7	0.95232965	8.37E-238	1.53E-233	0.954	0.573
TPM1	7	0.94611225	6.40E-164	1.17E-159	0.9	0.599
S100A10	7	0.91090984	1.42E-210	2.58E-206	0.996	0.979

COL2A1	7	0.892317	2.51E-45	4.57E-41	0.779	0.675
MT-CYB	7	0.89114049	1.52E-161	2.77E-157	0.935	0.71
IGFBP5	7	0.87814464	7.70E-66	1.41E-61	0.413	0.176
PTMS	7	0.87797836	1.28E-171	2.34E-167	0.929	0.655
C12orf75	7	0.87616936	0.00E+00	0.00E+00	0.661	0.146
ID1	7	0.86170303	4.62E-108	8.43E-104	0.876	0.589
ITGB1	7	0.85955912	1.75E-254	3.20E-250	0.934	0.499
MYDGF	7	0.844447	5.19E-205	9.48E-201	0.966	0.767
ID3	7	0.84001015	2.58E-112	4.71E-108	0.856	0.557
MT-CO3	7	0.835577	4.86E-177	8.88E-173	0.974	0.883
C1orf122	7	0.83225806	9.78E-245	1.79E-240	0.851	0.38
PPP1R14B	7	0.82060996	1.85E-279	3.37E-275	0.682	0.182
CFL1	7	0.81730627	2.04E-172	3.73E-168	0.952	0.763
CYR61	7	0.81071065	4.49E-98	8.19E-94	0.72	0.404
COL5A1	7	0.80011052	0.00E+00	0.00E+00	0.775	0.15
MT-CO1	7	0.78179259	4.02E-154	7.33E-150	0.985	0.942
CTHRC1	7	0.781521	2.57E-102	4.69E-98	0.599	0.262
CKAP4	7	0.77713109	6.95E-234	1.27E-229	0.762	0.292
PRKCDBP	7	0.77434464	3.69E-180	6.74E-176	0.847	0.466
PFN1	7	0.77378835	5.66E-167	1.03E-162	0.987	0.873
CYP1B1	7	0.74883037	6.39E-68	1.17E-63	0.359	0.139
HCFC1R1	7	0.74415488	1.49E-150	2.72E-146	0.927	0.657
S100A11	7	0.73842814	4.95E-202	9.03E-198	0.995	0.914
FKBP1A	7	0.73749276	1.29E-218	2.36E-214	0.899	0.472
MT-ND1	7	0.73584098	5.51E-141	1.00E-136	0.918	0.633
MT-ATP6	7	0.7350504	2.28E-145	4.16E-141	0.966	0.808
PPIC	7	0.72805363	1.30E-185	2.37E-181	0.879	0.453
TPM4	7	0.72229633	4.37E-249	7.98E-245	0.77	0.263
COL11A1	7	0.71983295	5.13E-108	9.35E-104	0.925	0.71
NREP	7	0.71543571	6.59E-229	1.20E-224	0.712	0.232
C9orf3	7	0.70488454	3.85E-174	7.02E-170	0.799	0.382
KCNMA1	7	0.6976959	2.60E-29	4.74E-25	0.696	0.544
IGFBP3	7	0.69646059	3.38E-69	6.16E-65	0.592	0.298
CALR	7	0.69566472	2.73E-137	4.98E-133	0.921	0.657
C4orf48	7	0.69264091	2.95E-190	5.38E-186	0.798	0.344
FSTL1	7	0.68434431	3.12E-223	5.69E-219	0.812	0.325
MALL	7	0.67508667	9.38E-182	1.71E-177	0.684	0.253
TNC	7	0.67408957	0.00E+00	0.00E+00	0.517	0.069
SEC61B	7	0.67123014	1.12E-147	2.04E-143	0.95	0.809

SRM	7	0.6634503	2.18E-158	3.97E-154	0.756	0.351
LOX	7	0.66134922	3.11E-232	5.68E-228	0.738	0.228
OAZ1	7	0.65731567	4.50E-216	8.21E-212	1	0.997
EIF5A	7	0.65346457	2.39E-126	4.37E-122	0.847	0.55
CALU	7	0.64493038	1.51E-165	2.75E-161	0.913	0.566
AXL	7	0.64471068	1.65E-128	3.01E-124	0.7	0.323
C7orf73	7	0.63472702	1.56E-160	2.84E-156	0.95	0.736
RRBP1	7	0.63205208	1.09E-167	1.98E-163	0.809	0.397
GUK1	7	0.63005442	1.32E-161	2.41E-157	0.989	0.88
OSTC	7	0.62969983	8.08E-171	1.47E-166	0.98	0.8
METRN	7	0.62714618	7.43E-228	1.36E-223	0.624	0.175
MEG3	7	0.6234493	8.02E-99	1.46E-94	0.59	0.281
MT-CO2	7	0.62268439	8.14E-112	1.48E-107	0.983	0.927
SCD	7	0.61512662	1.16E-190	2.11E-186	0.528	0.142
PDGFRA	7	0.61386216	2.15E-205	3.93E-201	0.716	0.246
OAF	7	0.61385302	5.45E-172	9.95E-168	0.674	0.252
DOK1	7	0.61358442	5.10E-130	9.30E-126	0.515	0.178
MYL6	7	0.61290549	1.95E-138	3.56E-134	0.988	0.927
CLEC11A	7	0.61148395	1.23E-155	2.24E-151	0.829	0.432
OST4	7	0.60987523	2.61E-212	4.77E-208	0.997	0.999
TGFBI	7	0.60502439	4.65E-54	8.48E-50	0.65	0.402
TTC3	7	0.60362079	2.57E-130	4.69E-126	0.921	0.687
CD151	7	0.59955377	9.82E-129	1.79E-124	0.813	0.471
PART1	7	0.5975284	1.77E-230	3.24E-226	0.499	0.107
SERPINH1	7	0.5931522	2.76E-261	5.03E-257	0.587	0.134
DSTN	7	0.59256689	8.36E-174	1.53E-169	1	0.997
YBX1	7	0.59236192	7.69E-154	1.40E-149	0.989	0.981
TPM2	7	0.59014026	3.02E-123	5.51E-119	0.878	0.555
DYNLL1	7	0.58695517	1.05E-113	1.91E-109	0.961	0.842
HMGN1	7	0.58482522	9.38E-125	1.71E-120	0.965	0.827
COPZ2	7	0.57934811	1.95E-186	3.56E-182	0.704	0.263
LOXL1	7	0.5722075	0.00E+00	0.00E+00	0.462	0.051
TMED9	7	0.55575415	3.17E-110	5.79E-106	0.878	0.61
MT-ND2	7	0.55522063	9.17E-93	1.67E-88	0.904	0.66
SNAI2	7	0.55111015	5.98E-88	1.09E-83	0.803	0.491
ENPP1	7	0.55070907	2.54E-69	4.63E-65	0.584	0.303
INSIG1	7	0.54846399	3.02E-74	5.52E-70	0.692	0.406
WWTR1	7	0.54780577	2.13E-147	3.88E-143	0.716	0.324
PPIB	7	0.54774567	1.20E-97	2.19E-93	0.974	0.892

EFEMP2	7	0.54737537	3.17E-153	5.78E-149	0.821	0.403
VCAN	7	0.54731232	1.89E-96	3.46E-92	0.767	0.419
MT-ND4	7	0.54397127	1.15E-115	2.09E-111	0.983	0.943
MYEOV2	7	0.54109535	2.14E-132	3.91E-128	0.937	0.706
MDFI	7	0.53856564	1.87E-103	3.41E-99	0.818	0.49
ATP5E	7	0.53267948	1.57E-178	2.86E-174	0.999	0.996
TMED3	7	0.53130378	1.74E-123	3.17E-119	0.833	0.481
PTRF	7	0.5302236	1.47E-115	2.69E-111	0.832	0.482
NT5E	7	0.5287153	3.46E-122	6.32E-118	0.721	0.346
CNPY2	7	0.52712229	5.21E-103	9.50E-99	0.869	0.632
CALD1	7	0.52425519	1.34E-113	2.44E-109	0.879	0.546
UQCRQ	7	0.52359693	1.57E-116	2.87E-112	0.98	0.918
ATP5J2	7	0.52299036	3.96E-121	7.22E-117	0.86	0.524
DNM3OS	7	0.52033966	2.93E-119	5.35E-115	0.731	0.345
LMAN1	7	0.51927522	1.69E-152	3.09E-148	0.735	0.325
DBI	7	0.51392711	2.97E-98	5.43E-94	0.917	0.728
RBX1	7	0.51235943	3.19E-115	5.82E-111	0.865	0.559
COL6A2	7	0.51125008	1.96E-89	3.58E-85	0.884	0.604
PHLDA2	7	0.50777156	1.26E-87	2.30E-83	0.42	0.155
PGF	7	0.50625085	1.43E-125	2.61E-121	0.245	0.043
S100A16	7	0.50502209	9.05E-95	1.65E-90	0.669	0.34
IFITM10	7	0.50341781	6.72E-168	1.23E-163	0.611	0.2
LY6E	7	0.50250371	5.38E-101	9.82E-97	0.783	0.456
IGFBP4	7	0.50064185	2.34E-136	4.27E-132	0.437	0.123
FBN1	7	0.4980677	5.39E-215	9.84E-211	0.528	0.125
NME4	7	0.49783307	2.13E-109	3.90E-105	0.771	0.432
SEP15	7	0.49772403	1.71E-142	3.11E-138	0.813	0.419
PRRX1	7	0.4972137	1.95E-139	3.55E-135	0.556	0.192
AHNAK	7	0.49537996	2.86E-105	5.23E-101	0.793	0.461
LINC00152	7	0.49433113	1.76E-93	3.21E-89	0.67	0.338
MIR4435-2HG	7	0.49426897	4.98E-139	9.09E-135	0.59	0.214
TUBA1B	7	0.49421581	5.73E-38	1.04E-33	0.542	0.342
PDLIM2	7	0.49331139	1.30E-107	2.37E-103	0.82	0.495
CXXC5	7	0.49189226	4.34E-117	7.92E-113	0.711	0.332
TMED2	7	0.49026147	5.43E-109	9.92E-105	0.966	0.818
CD99	7	0.48422441	9.52E-121	1.74E-116	0.989	0.922
CRIP2	7	0.48136719	3.40E-82	6.20E-78	0.711	0.412
MT-ND3	7	0.47931787	1.46E-83	2.67E-79	0.938	0.685
LMNA	7	0.47905963	1.11E-132	2.02E-128	0.694	0.302

FOXP1	7	0.47820414	5.34E-114	9.74E-110	0.595	0.252
BCL7C	7	0.47593426	3.02E-132	5.51E-128	0.639	0.269
KDELR3	7	0.472774	1.16E-147	2.12E-143	0.672	0.26
TMEM167A	7	0.4723614	1.23E-99	2.25E-95	0.907	0.695
DRAP1	7	0.47228638	7.17E-90	1.31E-85	0.849	0.576
CRELD2	7	0.47204106	2.77E-137	5.05E-133	0.579	0.211
SAT1	7	0.46893941	4.01E-20	7.32E-16	0.964	0.943
VKORC1	7	0.46580019	3.47E-100	6.34E-96	0.997	0.947
ATP5G1	7	0.4657166	1.67E-106	3.05E-102	0.75	0.413
ATP1B1	7	0.46396027	3.36E-112	6.13E-108	0.451	0.149
NME1	7	0.46162774	4.07E-104	7.42E-100	0.585	0.259
FKBP10	7	0.4611951	1.74E-98	3.18E-94	0.798	0.461
HDLBP	7	0.45984282	4.79E-108	8.74E-104	0.82	0.48
FAM114A1	7	0.45960667	2.04E-122	3.72E-118	0.684	0.32
AP2S1	7	0.45950323	4.91E-87	8.96E-83	0.81	0.524
ITGA11	7	0.4575459	1.42E-179	2.58E-175	0.462	0.112
HMGN3	7	0.45658819	3.25E-100	5.93E-96	0.736	0.398
HEBP2	7	0.45540649	1.11E-81	2.02E-77	0.767	0.497
DAP	7	0.45216953	1.86E-104	3.39E-100	0.793	0.467
ANXA2	7	0.45154618	9.00E-88	1.64E-83	0.957	0.849
NEDD8	7	0.44824117	8.95E-95	1.63E-90	0.904	0.71
RP11-14N7.2	7	0.44792188	1.96E-182	3.58E-178	0.557	0.158
SOX4	7	0.44786703	3.15E-104	5.74E-100	0.306	0.077
ZFP36L1	7	0.44688624	2.40E-52	4.38E-48	0.603	0.357
EIF4EBP1	7	0.44594144	5.05E-72	9.21E-68	0.801	0.526
LINC01420	7	0.44315744	4.04E-92	7.37E-88	0.809	0.539
CD59	7	0.44242086	2.24E-95	4.09E-91	0.987	0.938
GNAS	7	0.44171764	1.04E-94	1.89E-90	0.876	0.611
NEAT1	7	0.43910684	2.26E-46	4.12E-42	1	0.99
POSTN	7	0.43902464	1.54E-68	2.82E-64	0.143	0.026
KDELR2	7	0.4384529	1.25E-92	2.28E-88	0.966	0.853
LOXL2	7	0.43630668	6.30E-290	1.15E-285	0.507	0.085
TBL1XR1	7	0.43597544	1.24E-71	2.27E-67	0.649	0.358
NDUFA4L2	7	0.43476963	7.52E-103	1.37E-98	0.996	0.999
CRNDE	7	0.43357928	9.16E-158	1.67E-153	0.596	0.199
TXN	7	0.43317428	2.52E-81	4.60E-77	0.969	0.84
H3F3A	7	0.43135476	3.75E-113	6.85E-109	0.997	0.957
SRSF2	7	0.43112441	1.23E-96	2.24E-92	0.739	0.403
PYCR1	7	0.42646733	3.95E-185	7.21E-181	0.52	0.135

KIAA0101	7	0.42610245	1.03E-125	1.87E-121	0.264	0.049
NOP10	7	0.4259789	1.49E-93	2.71E-89	0.952	0.753
CAPNS1	7	0.42536214	5.23E-138	9.55E-134	0.6	0.224
TIMM8B	7	0.42394038	9.31E-86	1.70E-81	0.759	0.465
PTTG1IP	7	0.42393659	5.92E-82	1.08E-77	0.77	0.458
ERLEC1	7	0.42346873	4.40E-84	8.03E-80	0.734	0.432
GTF3A	7	0.4234111	6.42E-86	1.17E-81	0.682	0.387
WBP5	7	0.42303519	1.24E-82	2.26E-78	0.925	0.737
NT5DC2	7	0.42147129	5.84E-190	1.07E-185	0.474	0.111
IDI1	7	0.42084635	1.32E-83	2.41E-79	0.585	0.281
GPC6	7	0.41937593	3.60E-112	6.56E-108	0.629	0.276
RBM3	7	0.41934594	7.64E-79	1.39E-74	0.795	0.524
SSR1	7	0.41863834	7.30E-89	1.33E-84	0.806	0.504
ZNHIT1	7	0.41805718	5.67E-89	1.03E-84	0.833	0.549
MARCKS	7	0.41771925	6.15E-99	1.12E-94	0.37	0.114
NFIC	7	0.41629344	1.01E-83	1.85E-79	0.723	0.416
NAV1	7	0.41477446	4.42E-186	8.06E-182	0.495	0.123
MAP1B	7	0.41427401	2.13E-143	3.89E-139	0.41	0.105
P4HA1	7	0.41413627	5.99E-69	1.09E-64	0.872	0.652
ENG	7	0.41406214	9.37E-285	1.71E-280	0.49	0.081
ADIRF	7	0.41213701	8.49E-13	1.55E-08	0.661	0.576
LMO4	7	0.41207885	4.45E-91	8.13E-87	0.599	0.277
LOXL3	7	0.40963682	6.58E-164	1.20E-159	0.491	0.131
ARPC5	7	0.40773152	6.67E-83	1.22E-78	0.824	0.54
GNG11	7	0.40693443	5.63E-83	1.03E-78	0.556	0.26
RTN4	7	0.40633733	3.21E-83	5.85E-79	0.899	0.626
S100A6	7	0.4035507	7.36E-69	1.34E-64	1	1
SMIM14	7	0.40217162	3.88E-96	7.07E-92	0.677	0.337
GANAB	7	0.39955285	1.92E-123	3.50E-119	0.645	0.268
MGLL	7	0.39764864	1.04E-111	1.89E-107	0.448	0.145
COX20	7	0.39747396	1.19E-70	2.17E-66	0.853	0.626
SCX	7	0.39624733	2.34E-133	4.28E-129	0.277	0.053
SLC9A3R2	7	0.3943982	1.21E-53	2.21E-49	0.529	0.292
GNAI2	7	0.39418002	1.77E-107	3.23E-103	0.634	0.285
DAB2	7	0.39361681	4.04E-58	7.38E-54	0.639	0.384
KRT10	7	0.39323652	7.96E-73	1.45E-68	0.931	0.825
FSCN1	7	0.39308189	4.65E-176	8.48E-172	0.526	0.142
CD81	7	0.39302105	2.45E-99	4.47E-95	0.642	0.303
CCND1	7	0.39125025	6.74E-41	1.23E-36	0.486	0.27

HMGB1	7	0.3908907	4.13E-50	7.54E-46	0.894	0.759
DYNLT1	7	0.38988598	4.56E-81	8.32E-77	0.766	0.475
B4GALT1	7	0.38966968	3.30E-81	6.03E-77	0.33	0.106
P3H4	7	0.38859413	1.11E-144	2.03E-140	0.526	0.165
EIF4G2	7	0.38677606	2.83E-78	5.16E-74	0.833	0.565
PTN	7	0.38648463	0.00E+00	0.00E+00	0.363	0.034
MMP24-AS1	7	0.3853893	4.26E-65	7.78E-61	0.756	0.522
NDUFB2	7	0.38533939	2.94E-68	5.36E-64	0.906	0.764
NFIX	7	0.3843497	1.45E-109	2.64E-105	0.557	0.222
SEP	7	0.38357585	1.82E-148	3.32E-144	0.49	0.142
ID2	7	0.38331468	1.54E-37	2.82E-33	0.763	0.559
SHC1	7	0.38327875	1.91E-144	3.49E-140	0.49	0.147
GREM1	7	0.38264543	2.38E-13	4.35E-09	0.213	0.124
LIMCH1	7	0.38225983	2.02E-111	3.69E-107	0.4	0.122
TMA7	7	0.38084849	1.99E-103	3.63E-99	0.997	0.988
TCEB2	7	0.38038316	2.10E-105	3.83E-101	0.995	0.967
TNFRSF12A	7	0.38001477	1.13E-54	2.07E-50	0.68	0.401
ADAMTS6	7	0.37991935	3.47E-68	6.33E-64	0.382	0.15
LINC00998	7	0.37970189	2.86E-79	5.21E-75	0.618	0.322
HMGN2	7	0.3794301	7.19E-36	1.31E-31	0.929	0.895
ATP5G3	7	0.37791665	4.21E-60	7.68E-56	0.872	0.68
ITM2C	7	0.37740577	1.96E-46	3.58E-42	0.767	0.542
GOLIM4	7	0.37415561	3.42E-37	6.24E-33	0.653	0.435
FAM127A	7	0.37361678	5.97E-109	1.09E-104	0.592	0.249
TRAPPC1	7	0.37249875	6.18E-67	1.13E-62	0.708	0.441
MLEC	7	0.37209937	2.72E-72	4.96E-68	0.672	0.389
HMOX1	7	0.37196614	7.07E-35	1.29E-30	0.355	0.184
GPX8	7	0.37159805	2.32E-91	4.23E-87	0.604	0.281
KCNQ1OT1	7	0.37158611	1.08E-105	1.98E-101	0.443	0.149
DKK3	7	0.37157962	9.30E-130	1.70E-125	0.495	0.156
MORF4L2	7	0.37153232	3.39E-72	6.19E-68	0.865	0.641
TMEM230	7	0.3715228	7.47E-73	1.36E-68	0.908	0.7
H2AFV	7	0.37132707	6.50E-52	1.19E-47	0.732	0.499
NAA38	7	0.37046146	3.51E-62	6.40E-58	0.853	0.64
SQLE	7	0.36934571	1.44E-93	2.64E-89	0.495	0.195
SIX1	7	0.36858491	3.03E-91	5.53E-87	0.419	0.147
COL14A1	7	0.36753251	1.57E-97	2.86E-93	0.283	0.07
LSM7	7	0.36586242	1.02E-72	1.87E-68	0.771	0.496
HSPB6	7	0.36469223	1.36E-95	2.48E-91	0.435	0.154

PLOD1	7	0.36449517	2.11E-85	3.84E-81	0.694	0.376
RCN2	7	0.36435539	1.55E-67	2.82E-63	0.871	0.636
CRLF1	7	0.36380338	8.16E-69	1.49E-64	0.538	0.261
COL8A1	7	0.3622733	7.43E-117	1.36E-112	0.273	0.057
C11orf24	7	0.36078256	3.23E-160	5.89E-156	0.45	0.114
YIF1A	7	0.36028598	3.71E-65	6.77E-61	0.688	0.422
P3H2	7	0.35747198	3.86E-60	7.04E-56	0.498	0.251
SNF8	7	0.35704296	5.22E-67	9.53E-63	0.653	0.374
SPATS2L	7	0.35679621	3.10E-72	5.66E-68	0.721	0.428
ARPC2	7	0.35577835	1.09E-44	1.99E-40	0.882	0.79
BEND6	7	0.3556982	4.48E-217	8.18E-213	0.45	0.087
MTDH	7	0.35564096	4.66E-58	8.51E-54	0.849	0.642
CDK2AP2	7	0.35552265	5.71E-75	1.04E-70	0.567	0.275
HAPLN1	7	0.35547594	2.43E-19	4.43E-15	0.725	0.624
PRR7	7	0.3552025	1.53E-96	2.79E-92	0.478	0.182
EI24	7	0.35408592	2.38E-69	4.35E-65	0.661	0.381
SSR2	7	0.35375106	1.08E-63	1.97E-59	0.948	0.844
MRPL52	7	0.35352385	8.86E-69	1.62E-64	0.774	0.496
RPSA	7	0.35314171	1.22E-78	2.23E-74	1	0.999
ENAH	7	0.35306273	3.68E-75	6.72E-71	0.616	0.312
THBS1	7	0.35166917	2.99E-66	5.46E-62	0.289	0.094
FAAP20	7	0.35098296	6.06E-68	1.11E-63	0.777	0.498
COL27A1	7	0.35059459	2.05E-102	3.75E-98	0.47	0.168
CNN3	7	0.35028166	1.64E-75	2.99E-71	0.61	0.307
OAZ2	7	0.35002787	1.84E-87	3.35E-83	0.649	0.327
SDF2L1	7	0.34972436	1.15E-78	2.10E-74	0.54	0.245
ATOX1	7	0.34951261	1.57E-60	2.86E-56	0.894	0.765
EMILIN1	7	0.34907683	5.72E-134	1.04E-129	0.478	0.144
GLRX	7	0.34882354	1.84E-73	3.35E-69	0.635	0.321
JOSD2	7	0.34815194	2.40E-74	4.38E-70	0.744	0.457
TIMM13	7	0.34693047	2.28E-70	4.16E-66	0.712	0.433
TAX1BP3	7	0.34675414	1.94E-88	3.55E-84	0.529	0.225
ANKH	7	0.34581684	3.10E-61	5.66E-57	0.791	0.53
COX7A1	7	0.34577109	5.23E-45	9.55E-41	0.913	0.815
ARPC1A	7	0.34541186	7.52E-66	1.37E-61	0.785	0.512
CTTN	7	0.34442189	1.97E-50	3.60E-46	0.639	0.399
UBL5	7	0.3441833	9.91E-68	1.81E-63	0.983	0.948
SMOC1	7	0.34358098	7.35E-77	1.34E-72	0.538	0.247
PGAM1	7	0.3433253	1.61E-52	2.93E-48	0.919	0.779

YWHAB	7	0.34270149	3.00E-74	5.48E-70	0.68	0.377
AEBP1	7	0.34198431	3.05E-50	5.56E-46	0.832	0.589
IL11	7	0.34111418	3.00E-08	5.48E-04	0.141	0.084
CERCAM	7	0.34094196	3.01E-173	5.50E-169	0.41	0.09
CYCS	7	0.34041716	1.49E-47	2.73E-43	0.696	0.483
GJA1	7	0.34014199	5.52E-62	1.01E-57	0.431	0.19
RCAN1	7	0.33977632	1.23E-71	2.24E-67	0.287	0.089
MRC2	7	0.33961049	1.02E-88	1.86E-84	0.564	0.248
TFDP1	7	0.33804909	1.16E-158	2.12E-154	0.456	0.116
IMPAD1	7	0.33797866	1.51E-125	2.76E-121	0.498	0.162
COTL1	7	0.33785625	4.38E-157	7.99E-153	0.373	0.081
GTF3C6	7	0.33744548	3.60E-66	6.57E-62	0.624	0.351
SNRPG	7	0.33684774	9.50E-49	1.73E-44	0.894	0.72
MRPL23	7	0.3357167	5.14E-72	9.38E-68	0.693	0.394
SUMO1	7	0.33518137	8.44E-70	1.54E-65	0.896	0.678
SPTLC3	7	0.33439843	7.01E-50	1.28E-45	0.42	0.199
COX8A	7	0.33406991	3.45E-53	6.29E-49	0.948	0.873
LTBP2	7	0.33347197	1.70E-151	3.10E-147	0.417	0.102
DDIT4	7	0.33282563	1.11E-13	2.03E-09	0.583	0.466
CBX5	7	0.33191095	1.39E-66	2.53E-62	0.639	0.352
CDC42	7	0.33081004	2.83E-28	5.16E-24	0.779	0.649
SRSF9	7	0.33071268	3.90E-57	7.11E-53	0.787	0.532
LSM1	7	0.33069265	2.26E-83	4.12E-79	0.58	0.275
CLMP	7	0.3305993	6.10E-88	1.11E-83	0.479	0.187
ATP5I	7	0.32926778	7.77E-74	1.42E-69	0.989	0.955
MARVELD1	7	0.32907372	1.13E-163	2.07E-159	0.428	0.101
MMP14	7	0.32860036	2.91E-76	5.31E-72	0.439	0.175
C6orf62	7	0.32856553	3.81E-75	6.96E-71	0.62	0.322
P4HB	7	0.32837233	2.56E-54	4.67E-50	0.964	0.808
FLNA	7	0.32769368	1.36E-115	2.48E-111	0.44	0.137
FKBP2	7	0.32747736	4.76E-52	8.68E-48	0.857	0.655
LTBP1	7	0.32716732	1.49E-64	2.71E-60	0.351	0.135
P2RY6	7	0.32711201	3.21E-253	5.87E-249	0.332	0.04
FDPS	7	0.32674006	4.98E-71	9.09E-67	0.489	0.219
TSPO	7	0.32665431	1.10E-57	2.01E-53	0.95	0.813
SWI5	7	0.32629499	6.74E-67	1.23E-62	0.693	0.415
TXNL4A	7	0.32601313	4.99E-60	9.11E-56	0.697	0.437
MLF2	7	0.32564533	2.46E-67	4.49E-63	0.719	0.44
BANF1	7	0.32532369	1.14E-58	2.07E-54	0.766	0.509

SEC61G	7	0.32469856	5.54E-68	1.01E-63	0.999	0.995
PMEPA1	7	0.32370185	2.70E-43	4.92E-39	0.443	0.237
ZNF580	7	0.32364858	2.95E-91	5.39E-87	0.577	0.254
VAMP5	7	0.3226026	1.04E-58	1.90E-54	0.707	0.445
PRMT2	7	0.3224115	8.37E-59	1.53E-54	0.767	0.505
MYL9	7	0.32211422	8.65E-69	1.58E-64	0.471	0.21
CTSZ	7	0.32210156	1.56E-100	2.85E-96	0.511	0.194
AP3S1	7	0.32201727	1.67E-63	3.04E-59	0.945	0.769
TPST1	7	0.32168673	5.24E-90	9.55E-86	0.46	0.173
LRP1	7	0.32160299	2.57E-57	4.69E-53	0.743	0.486
PTP4A2	7	0.31920833	1.61E-61	2.94E-57	0.74	0.466
GALNT1	7	0.31912144	9.30E-76	1.70E-71	0.537	0.25
SHFM1	7	0.31889473	4.76E-60	8.68E-56	0.945	0.855
GAS1	7	0.31868463	1.99E-57	3.63E-53	0.435	0.197
YWHAZ	7	0.31704263	2.56E-55	4.68E-51	0.801	0.563
MLLT11	7	0.31693856	7.65E-109	1.40E-104	0.358	0.099
GMDS	7	0.31592472	1.18E-60	2.15E-56	0.548	0.284
MXRA5	7	0.31504702	8.16E-220	1.49E-215	0.339	0.048
DUSP14	7	0.31496352	9.65E-84	1.76E-79	0.46	0.181
UQCC2	7	0.31482168	1.91E-46	3.48E-42	0.696	0.468
TMEM263	7	0.313997	3.00E-66	5.47E-62	0.622	0.339
JTB	7	0.31398425	5.49E-51	1.00E-46	0.892	0.728
TMEM165	7	0.31366981	1.41E-66	2.56E-62	0.635	0.343
ACTB	7	0.3130224	5.60E-20	1.02E-15	0.956	0.945
HIGD2A	7	0.31207778	1.51E-65	2.75E-61	0.623	0.345
TADA3	7	0.31179792	3.35E-72	6.12E-68	0.631	0.331
MYLK	7	0.31103572	3.58E-37	6.53E-33	0.467	0.266
ACTN4	7	0.31008871	1.15E-76	2.10E-72	0.498	0.217
SH3BGRL	7	0.30976803	1.49E-52	2.72E-48	0.748	0.515
KAZALD1	7	0.30909219	4.73E-85	8.64E-81	0.432	0.161
ITGAE	7	0.30879379	2.85E-104	5.20E-100	0.511	0.189
TMEM106C	7	0.3084494	1.25E-44	2.28E-40	0.565	0.345
ATPIF1	7	0.30840171	7.86E-47	1.43E-42	0.863	0.674
ITGB1BP1	7	0.30755041	4.22E-59	7.70E-55	0.707	0.439
CHCHD2	7	0.30746929	1.13E-64	2.05E-60	0.991	0.964
C14orf1	7	0.30734277	8.47E-55	1.55E-50	0.544	0.293
ADAMTS2	7	0.30715725	2.40E-129	4.38E-125	0.443	0.127
TRPS1	7	0.30677537	1.23E-53	2.24E-49	0.715	0.443
FHL1	7	0.30664205	3.33E-13	6.08E-09	0.546	0.429

GPAA1	7	0.30656221	4.59E-66	8.37E-62	0.638	0.348
MRPL14	7	0.30549071	2.13E-53	3.89E-49	0.794	0.556
GTF2H5	7	0.3053176	1.91E-43	3.48E-39	0.793	0.604
CTDNEP1	7	0.30511085	2.50E-74	4.57E-70	0.549	0.262
PLEC	7	0.30464714	4.37E-124	7.97E-120	0.396	0.108
ISLR	7	0.30428618	3.84E-44	7.01E-40	0.715	0.463
CBX6	7	0.3039908	2.53E-52	4.62E-48	0.482	0.249
MPC2	7	0.30319969	1.80E-57	3.28E-53	0.707	0.441
HMGCS1	7	0.30274993	6.43E-70	1.17E-65	0.334	0.117
TGFB1	7	0.30261025	9.71E-96	1.77E-91	0.394	0.129
COX7B	7	0.30230479	1.39E-44	2.54E-40	0.813	0.621
UQCR11	7	0.30201524	1.59E-66	2.90E-62	0.987	0.961
TLN1	7	0.30107048	1.78E-83	3.25E-79	0.485	0.198
AES	7	0.30095063	7.11E-71	1.30E-66	0.553	0.27
BOLA3	7	0.3008222	4.03E-60	7.34E-56	0.536	0.276
MXD4	7	0.30078383	4.12E-129	7.52E-125	0.369	0.092
RHOA	7	0.3003863	8.22E-47	1.50E-42	0.926	0.835
PNKD	7	0.29984842	2.73E-63	4.98E-59	0.643	0.36
PPIA	7	0.29968406	2.55E-56	4.65E-52	0.989	0.959
SURF4	7	0.2996305	5.21E-74	9.50E-70	0.536	0.25
RPL22L1	7	0.29955342	7.83E-32	1.43E-27	0.773	0.618
METTL9	7	0.29908422	5.46E-78	9.96E-74	0.591	0.284
C17orf89	7	0.29901315	1.19E-57	2.18E-53	0.654	0.393
NQO1	7	0.29844041	3.87E-126	7.07E-122	0.357	0.087
PTGER2	7	0.2982768	2.80E-126	5.11E-122	0.338	0.079
ERH	7	0.29801889	5.31E-44	9.70E-40	0.884	0.71
TPI1	7	0.29687385	1.61E-46	2.94E-42	0.993	0.989
NDUFS6	7	0.29666587	3.84E-45	7.01E-41	0.624	0.411
AP2M1	7	0.296326	7.26E-47	1.32E-42	0.789	0.543
SEC61A1	7	0.29620202	2.12E-98	3.86E-94	0.486	0.178
UQCR10	7	0.29604656	1.30E-52	2.38E-48	0.938	0.798
NDUFA11	7	0.29548741	2.48E-52	4.52E-48	0.956	0.875
YIF1B	7	0.29544314	1.15E-88	2.10E-84	0.476	0.186
NHP2	7	0.29492855	6.05E-48	1.10E-43	0.709	0.479
IER3IP1	7	0.29483498	3.07E-48	5.60E-44	0.834	0.62
HSPB1	7	0.2945994	1.18E-33	2.16E-29	0.864	0.7
TSPAN4	7	0.29452957	1.28E-44	2.33E-40	0.696	0.466
NGFRAP1	7	0.29230824	2.27E-33	4.14E-29	0.938	0.838
MAP7D3	7	0.2915499	5.48E-48	9.99E-44	0.491	0.259

PXDN	7	0.29097699	5.05E-131	9.21E-127	0.386	0.098
HRCT1	7	0.29092151	1.71E-76	3.12E-72	0.252	0.067
CDK2AP1	7	0.28979791	1.98E-92	3.61E-88	0.458	0.168
SLC20A1	7	0.28977729	1.18E-36	2.15E-32	0.432	0.237
SRA1	7	0.28902951	1.62E-63	2.96E-59	0.502	0.243
LINC00116	7	0.28896528	7.75E-46	1.41E-41	0.759	0.541
BRI3	7	0.28881987	1.43E-42	2.61E-38	0.937	0.83
SULF1	7	0.28855779	6.10E-72	1.11E-67	0.316	0.104
MAGED1	7	0.28801541	5.03E-89	9.18E-85	0.577	0.239
DNAJC22	7	0.28795223	1.00E-137	1.83E-133	0.221	0.032
ORAI2	7	0.28786783	6.18E-104	1.13E-99	0.405	0.127
SLC38A2	7	0.28766881	3.70E-51	6.75E-47	0.627	0.371
COX6C	7	0.28753148	2.08E-47	3.79E-43	0.931	0.835
ANAPC11	7	0.2868404	1.42E-40	2.59E-36	0.89	0.756
TMEM14A	7	0.28671861	1.28E-53	2.33E-49	0.63	0.375
DGCR6L	7	0.28577249	2.49E-51	4.55E-47	0.63	0.388
CETN2	7	0.28531849	8.12E-61	1.48E-56	0.584	0.308
ST6GALNAC6	7	0.2851133	2.16E-81	3.95E-77	0.495	0.208
SDHC	7	0.28497755	3.59E-60	6.55E-56	0.553	0.291
ROMO1	7	0.28456062	1.60E-39	2.91E-35	0.913	0.821
SNHG18	7	0.28415562	1.19E-63	2.17E-59	0.556	0.278
HSPE1	7	0.28414991	6.85E-31	1.25E-26	0.781	0.628
AP2B1	7	0.28411154	1.89E-50	3.44E-46	0.513	0.278
PRELID1	7	0.28382602	3.84E-39	7.00E-35	0.875	0.727
SHISA4	7	0.28372104	1.45E-62	2.64E-58	0.482	0.227
SLC35B2	7	0.28353584	2.14E-73	3.90E-69	0.486	0.208
IKBIP	7	0.28348141	1.73E-63	3.16E-59	0.53	0.26
SEPT2	7	0.2833341	2.03E-60	3.70E-56	0.592	0.326
SMIM5	7	0.28319927	1.51E-74	2.75E-70	0.331	0.11
CYB5R3	7	0.28303528	2.63E-44	4.80E-40	0.849	0.646
TBPL1	7	0.28273564	9.79E-41	1.79E-36	0.53	0.316
RSPO2	7	0.28248356	2.58E-84	4.72E-80	0.268	0.07
SLC1A5	7	0.28230415	1.21E-45	2.21E-41	0.475	0.255
FKBP11	7	0.28218148	1.41E-64	2.56E-60	0.448	0.195
RABAC1	7	0.28145649	1.09E-44	1.99E-40	0.946	0.819
ADAM12	7	0.28106138	3.24E-219	5.92E-215	0.303	0.038
SDC2	7	0.28052795	4.99E-29	9.10E-25	0.914	0.824
ZNF428	7	0.28046504	9.22E-66	1.68E-61	0.563	0.283
CAPZA1	7	0.28022249	2.60E-47	4.75E-43	0.604	0.363

ARL5A	7	0.27967895	1.63E-51	2.98E-47	0.665	0.414
ISOC2	7	0.27676495	3.74E-65	6.82E-61	0.495	0.232
CALM1	7	0.27651767	4.44E-36	8.10E-32	0.754	0.537
TMEM14B	7	0.27597885	8.11E-48	1.48E-43	0.738	0.501
CDC42EP1	7	0.27590701	1.64E-109	2.99E-105	0.398	0.118
NPM3	7	0.275287	8.51E-86	1.55E-81	0.421	0.153
B4GALT7	7	0.27457266	6.83E-79	1.25E-74	0.464	0.189
GAPDH	7	0.27443849	5.43E-35	9.91E-31	1	1
PHPT1	7	0.27420733	5.58E-44	1.02E-39	0.913	0.751
SKA2	7	0.27329592	3.19E-102	5.83E-98	0.357	0.102
MAZ	7	0.27276654	1.49E-78	2.72E-74	0.459	0.185
UBE2J1	7	0.27265258	4.53E-57	8.26E-53	0.498	0.248
TPGS2	7	0.27230296	3.35E-59	6.11E-55	0.522	0.263
MYL12A	7	0.2716498	4.76E-33	8.68E-29	0.728	0.55
SERP1	7	0.2716265	3.42E-34	6.24E-30	0.899	0.829
GOLM1	7	0.271507	1.20E-61	2.19E-57	0.472	0.22
BIRC2	7	0.27135908	4.28E-44	7.81E-40	0.653	0.414
LAMTOR1	7	0.27113905	9.92E-44	1.81E-39	0.786	0.575
MANF	7	0.27039014	3.71E-47	6.77E-43	0.567	0.322
B4GALT2	7	0.27021393	4.38E-158	7.99E-154	0.323	0.06
PLOD2	7	0.27018021	9.69E-41	1.77E-36	0.892	0.741
PABPN1	7	0.26988459	2.56E-74	4.67E-70	0.467	0.195
SGCB	7	0.26977438	4.69E-44	8.56E-40	0.651	0.407
NDUFA13	7	0.26929896	1.13E-33	2.07E-29	0.791	0.622
EHBP1	7	0.2685783	2.01E-91	3.66E-87	0.361	0.112
FIBP	7	0.26834694	2.27E-45	4.14E-41	0.639	0.406
EHD2	7	0.26770273	1.80E-72	3.29E-68	0.472	0.202
NTAN1	7	0.26695261	7.95E-63	1.45E-58	0.466	0.213
C4orf3	7	0.26692946	2.88E-46	5.25E-42	0.991	0.978
PRRX2	7	0.26655455	2.54E-79	4.63E-75	0.346	0.114
FAM46A	7	0.26611752	4.03E-19	7.35E-15	0.705	0.56
DNAJC3	7	0.26600732	3.38E-38	6.16E-34	0.58	0.366
SCN1B	7	0.26511383	1.11E-89	2.03E-85	0.37	0.119
MPG	7	0.26493858	2.65E-40	4.83E-36	0.69	0.474
MAP3K13	7	0.26479325	4.30E-62	7.84E-58	0.581	0.303
FKBP9	7	0.26477593	8.39E-61	1.53E-56	0.431	0.19
SYNGR1	7	0.26423367	1.54E-113	2.82E-109	0.363	0.097
DHCR24	7	0.26419919	1.02E-150	1.86E-146	0.304	0.056
IFT20	7	0.26414047	1.07E-57	1.95E-53	0.569	0.302

RARG	7	0.26407581	8.50E-77	1.55E-72	0.369	0.13
SELT	7	0.2631249	3.77E-40	6.89E-36	0.755	0.551
POLR2J3	7	0.26304652	5.27E-68	9.61E-64	0.486	0.217
FXYD6	7	0.26204232	4.93E-30	8.99E-26	0.907	0.805
MSMO1	7	0.2619842	1.30E-45	2.38E-41	0.502	0.271
POLR3H	7	0.26174766	2.62E-88	4.79E-84	0.386	0.13
TMEM54	7	0.26090655	1.21E-83	2.20E-79	0.396	0.139
RSRP1	7	0.26090534	4.02E-48	7.34E-44	0.525	0.289
CBR1	7	0.26052939	1.32E-47	2.41E-43	0.514	0.278
PKM	7	0.26046092	7.54E-35	1.38E-30	0.964	0.924
SMCO4	7	0.25951641	8.09E-66	1.48E-61	0.476	0.214
ERGIC1	7	0.25945499	1.13E-54	2.07E-50	0.502	0.254
ITGBL1	7	0.25898052	6.22E-70	1.13E-65	0.342	0.12
GTF2I	7	0.25856043	2.31E-71	4.21E-67	0.462	0.195
LAMTOR2	7	0.25790106	3.80E-44	6.94E-40	0.686	0.458
FAM89B	7	0.25710733	2.55E-67	4.65E-63	0.358	0.134
MXRA8	7	0.25694904	6.52E-56	1.19E-51	0.49	0.236
CCDC167	7	0.25692376	4.65E-69	8.48E-65	0.431	0.179
BRK1	7	0.25670802	7.85E-42	1.43E-37	0.945	0.846
SUB1	7	0.25663587	6.47E-34	1.18E-29	0.929	0.833
FTH1	7	0.25651392	1.20E-21	2.18E-17	1	1
GGH	7	0.25629657	5.19E-86	9.46E-82	0.369	0.119
SLC25A39	7	0.25624673	1.90E-52	3.48E-48	0.479	0.243
C19orf24	7	0.25529499	3.29E-47	6.00E-43	0.686	0.431
SRP14	7	0.25486548	1.37E-41	2.50E-37	0.989	0.982
NBL1	7	0.254737	2.53E-76	4.62E-72	0.244	0.064
CLIC3	7	0.25458902	1.53E-52	2.78E-48	0.365	0.154
DDX5	7	0.2545159	4.53E-37	8.27E-33	0.775	0.577
RP11-783K16.5	7	0.25433065	3.06E-289	5.58E-285	0.266	0.02
LMAN2	7	0.25397478	2.86E-42	5.21E-38	0.674	0.432
PDAP1	7	0.25371419	5.45E-41	9.94E-37	0.68	0.45
MZT2B	7	0.25369948	7.51E-44	1.37E-39	0.98	0.956
CDK16	7	0.25361715	1.35E-76	2.47E-72	0.386	0.141
CTNNB1	7	0.25307415	7.05E-11	1.29E-06	0.51	0.397
C5orf24	7	0.25281698	1.54E-54	2.80E-50	0.533	0.275
TUSC3	7	0.25245796	4.12E-49	7.51E-45	0.448	0.224
UROS	7	0.2523399	4.09E-101	7.47E-97	0.334	0.092
PRKCA	7	0.25199655	2.57E-84	4.69E-80	0.406	0.143
MINOS1	7	0.25157868	5.28E-31	9.63E-27	0.795	0.632

SKIL	7	0.25135358	1.02E-41	1.87E-37	0.459	0.25
DEXI	7	0.2511762	5.35E-74	9.76E-70	0.4	0.152
CD63	7	0.25109663	1.46E-30	2.67E-26	0.999	0.999
CCDC28B	7	0.25002377	8.81E-97	1.61E-92	0.346	0.1
**gene**	**cluster**	**avg_logFC**	**p_val**	**p_val_adj**	**pct.1**	**pct.2**
COL2A1	8	1.48140121	3.2664E-303	5.9602E-299	0.994	0.663
COL11A1	8	1.13204728	2.6986E-257	4.924E-253	0.989	0.707
SLPI	8	0.98701574	2.31205E-93	4.21881E-89	0.983	0.925
COL9A3	8	0.90520274	1.76E-150	3.219E-146	0.915	0.615
SPARC	8	0.87894218	1.3187E-186	2.4062E-182	0.948	0.575
C2orf82	8	0.819749	6.6143E-107	1.2069E-102	0.981	0.951
CRISPLD1	8	0.73254663	2.6585E-102	4.85094E-98	0.585	0.245
LECT1	8	0.69438676	7.3898E-116	1.3484E-111	0.973	0.767
PLAC9	8	0.69330274	5.713E-139	1.0425E-134	0.98	0.792
CLEC3A	8	0.68942105	2.84521E-81	5.19166E-77	0.84	0.533
PPIC	8	0.68265289	1.50E-155	2.731E-151	0.867	0.455
COL5A2	8	0.67541151	7.2656E-148	1.3258E-143	0.834	0.389
ACAN	8	0.65547774	2.4028E-138	4.3844E-134	0.997	0.914
HAPLN1	8	0.63029011	5.28826E-95	9.64949E-91	0.884	0.616
COL11A2	8	0.62504366	1.4587E-144	2.6618E-140	0.867	0.483
CAPS	8	0.59451507	8.50864E-70	1.55257E-65	0.894	0.736
COL9A2	8	0.57836408	1.5367E-116	2.804E-112	0.738	0.357
SNAI2	8	0.57395498	2.07E-69	3.76981E-65	0.748	0.495
RBP4	8	0.55988431	3.57789E-72	6.52858E-68	0.918	0.729
MDFI	8	0.54605439	8.1522E-104	1.4875E-99	0.825	0.491
MATN4	8	0.52812826	9.60617E-98	1.75284E-93	0.474	0.172
ID4	8	0.51942016	1.02391E-73	1.86833E-69	0.804	0.533
ID3	8	0.49695661	1.47051E-60	2.68325E-56	0.832	0.559
SPTSSB	8	0.48787972	2.46141E-31	4.49134E-27	0.513	0.322
PPIB	8	0.48282568	2.48E-95	4.52681E-91	0.971	0.892
ID1	8	0.47446958	9.8052E-64	1.78915E-59	0.858	0.591
C6orf48	8	0.46869068	1.25314E-77	2.28661E-73	0.94	0.855
FBXO2	8	0.46624319	1.03144E-72	1.88206E-68	0.911	0.732
LSP1	8	0.45139491	2.55491E-77	4.66195E-73	0.648	0.326
P4HB	8	0.44495429	2.45388E-69	4.47759E-65	0.963	0.808
SSR4	8	0.43616931	2.2489E-100	4.1035E-96	0.994	0.957
SERPINA5	8	0.43615068	1.41E-77	2.56726E-73	0.818	0.514
IGFBP6	8	0.43424554	6.16158E-46	1.1243E-41	0.831	0.637
C7orf73	8	0.42941238	4.19358E-78	7.65203E-74	0.918	0.738

SCRG1	8	0.41896094	3.1562E-63	5.75911E-59	1	0.974
ID2	8	0.41167304	2.44741E-42	4.46578E-38	0.766	0.56
FMOD	8	0.4064008	9.97381E-86	1.81992E-81	0.99	0.883
MYDGF	8	0.40605269	4.07597E-70	7.43743E-66	0.914	0.77
DNM3OS	8	0.39642035	2.15E-75	3.91791E-71	0.678	0.35
OSTC	8	0.39251119	8.89826E-78	1.62367E-73	0.951	0.802
NUCB2	8	0.39056307	4.16848E-52	7.60623E-48	0.695	0.44
MIA	8	0.38860917	6.58195E-67	1.20101E-62	0.996	0.96
PDIA6	8	0.38695471	6.9187E-61	1.26246E-56	0.87	0.659
IGF2	8	0.38603605	1.20429E-17	2.19747E-13	0.317	0.194
VKORC1	8	0.38319228	7.01829E-78	1.28063E-73	0.989	0.948
MYLK	8	0.37984135	4.83E-48	8.81825E-44	0.5	0.265
MT-CO2	8	0.37605511	3.54623E-55	6.47081E-51	0.979	0.927
SLC26A2	8	0.37594889	3.74367E-79	6.83107E-75	0.499	0.211
HSP90B1	8	0.37047515	1.218E-54	2.22248E-50	0.914	0.779
MSMO1	8	0.36117819	2.43638E-38	4.44565E-34	0.473	0.273
TSC22D1	8	0.35684225	7.39891E-36	1.35008E-31	0.867	0.714
TMED2	8	0.35523652	5.21032E-67	9.50727E-63	0.947	0.82
SOX9	8	0.35511791	4.77E-43	8.70849E-39	0.837	0.658
P4HA1	8	0.35330511	2.39347E-51	4.36736E-47	0.837	0.654
CD99	8	0.35110465	6.00867E-68	1.0964E-63	0.991	0.922
INSIG1	8	0.34899561	1.43635E-26	2.62092E-22	0.577	0.413
KDELR2	8	0.34879604	3.68324E-55	6.7208E-51	0.941	0.855
CNPY2	8	0.34490924	2.72112E-51	4.96523E-47	0.822	0.636
RCN2	8	0.34358315	1.15707E-58	2.1113E-54	0.86	0.637
ASPN	8	0.34082362	4.03E-13	7.35353E-09	0.218	0.126
EPYC	8	0.33945121	2.9218E-34	5.3314E-30	0.334	0.162
MT-ND3	8	0.33926099	1.39664E-49	2.54844E-45	0.924	0.687
IDI1	8	0.33402372	2.06502E-48	3.76805E-44	0.526	0.286
FXYD6	8	0.33382743	1.5197E-52	2.77299E-48	0.946	0.803
OAF	8	0.33179176	7.66759E-67	1.3991E-62	0.547	0.26
PRDX4	8	0.33105584	3.53551E-45	6.45125E-41	0.893	0.757
FXYD1	8	0.33032698	1.78E-39	3.2532E-35	0.519	0.306
TTC3	8	0.32753744	4.83476E-51	8.82198E-47	0.874	0.691
SDC2	8	0.32601922	1.62254E-42	2.96064E-38	0.938	0.823
SEC61B	8	0.322399	1.11261E-51	2.03018E-47	0.917	0.811
SERF2	8	0.32181282	2.30104E-91	4.19871E-87	1	1
TMED10	8	0.3215117	6.78101E-50	1.23733E-45	0.941	0.867
ISLR	8	0.31500576	4.7441E-39	8.65656E-35	0.699	0.464

S100A4	8	0.31397472	3.50E-42	6.38313E-38	0.895	0.768
S100B	8	0.31365267	7.77488E-32	1.41868E-27	0.874	0.743
ERGIC3	8	0.31232001	2.13918E-49	3.90335E-45	0.903	0.802
NGFRAP1	8	0.31192602	8.78888E-49	1.60371E-44	0.954	0.838
TMED3	8	0.31041642	4.64871E-42	8.48249E-38	0.701	0.489
TMEM230	8	0.30962661	1.43011E-45	2.60953E-41	0.86	0.704
TMEM258	8	0.30558546	6.71829E-71	1.22589E-66	0.996	0.996
SRM	8	0.30187232	2.57E-42	4.68693E-38	0.599	0.361
MAGED1	8	0.29844935	4.18862E-33	7.64298E-29	0.443	0.247
FKBP10	8	0.29459717	1.8401E-34	3.35763E-30	0.675	0.469
OGN	8	0.29230866	5.55849E-36	1.01426E-31	0.966	0.851
GREM1	8	0.29166194	7.25136E-23	1.32316E-18	0.248	0.122
PAPSS2	8	0.28923815	5.41164E-38	9.87463E-34	0.703	0.48
MT-CO3	8	0.28889409	4.6595E-40	8.50219E-36	0.974	0.883
DDIT4	8	0.28670993	3.67E-07	0.006696432	0.542	0.469
KDELR3	8	0.28643314	2.62313E-36	4.78642E-32	0.477	0.272
FKBP11	8	0.2848732	4.43319E-64	8.08925E-60	0.456	0.196
CHRDL2	8	0.28482394	1.8673E-21	3.40725E-17	0.357	0.209
KRT10	8	0.28382625	7.65569E-38	1.39693E-33	0.93	0.825
MT-ATP6	8	0.28338971	3.63683E-36	6.63612E-32	0.954	0.809
CXXC5	8	0.28311293	4.31908E-38	7.88102E-34	0.569	0.341
H3F3A	8	0.28185855	4.11E-54	7.49992E-50	0.993	0.958
BGN	8	0.28174934	3.11779E-44	5.68903E-40	1	0.984
OTOS	8	0.28014893	4.08215E-46	7.4487E-42	0.338	0.145
ITM2C	8	0.27956219	2.19205E-30	3.99983E-26	0.746	0.544
WWP2	8	0.27944177	2.23864E-27	4.08485E-23	0.745	0.578
TRPS1	8	0.27935094	1.21734E-34	2.22129E-30	0.659	0.447
GOLIM4	8	0.27746296	8.10664E-21	1.47922E-16	0.6	0.439
S100A13	8	0.2771758	1.52E-57	2.77818E-53	0.999	0.994
SSR1	8	0.27649181	2.47525E-35	4.5166E-31	0.708	0.51
CRIP2	8	0.27577937	1.61199E-31	2.94139E-27	0.61	0.419
C1orf122	8	0.27527734	2.67573E-43	4.8824E-39	0.638	0.393
MORF4L2	8	0.27469938	1.13245E-38	2.06637E-34	0.841	0.643
HERPUD1	8	0.27383678	1.60839E-20	2.93483E-16	0.63	0.484
MT-CO1	8	0.27282167	1.69106E-35	3.08568E-31	0.987	0.942
MT-ND1	8	0.26858204	1.90E-30	3.47466E-26	0.845	0.638
OST4	8	0.26697724	1.9394E-67	3.53882E-63	1	0.999
TSPO	8	0.26657237	1.55762E-36	2.84219E-32	0.911	0.816
STK26	8	0.26648573	2.17944E-43	3.97683E-39	0.477	0.251

ITM2A	8	0.26555598	4.49041E-23	8.19365E-19	0.685	0.517
NDUFA11	8	0.26409884	1.84072E-37	3.35876E-33	0.951	0.876
CRELD2	8	0.26172443	3.95824E-48	7.22261E-44	0.453	0.219
IFT20	8	0.26041752	2.09E-41	3.80936E-37	0.532	0.304
RBM3	8	0.25973722	3.17528E-34	5.79393E-30	0.728	0.529
RABAC1	8	0.25914191	2.22441E-38	4.05889E-34	0.928	0.821
MT-CYB	8	0.25911523	5.96775E-34	1.08894E-29	0.914	0.712
CRTAP	8	0.25518837	1.09051E-31	1.98985E-27	0.841	0.683
EDIL3	8	0.25395233	2.38537E-19	4.35258E-15	0.563	0.426
SERP1	8	0.25310065	8.59089E-35	1.56758E-30	0.918	0.828
MTHFD2	8	0.25159566	8.06E-18	1.47123E-13	0.678	0.556
PDIA4	8	0.25079744	1.44953E-33	2.64496E-29	0.56	0.356
**gene**	**cluster**	**avg_logFC**	**p_val**	**p_val_adj**	**pct.1**	**pct.2**
CTGF	9	2.36353243	2.19E-144	3.99E-140	0.903	0.444
CYR61	9	1.69705981	1.82E-126	3.31E-122	0.849	0.409
COL1A2	9	1.64081417	3.98E-167	7.27E-163	0.804	0.267
PCOLCE	9	1.36083535	5.88E-254	1.07E-249	0.883	0.251
EPYC	9	1.34793631	1.00E-85	1.83E-81	0.512	0.161
COL3A1	9	1.33567321	3.06E-202	5.58E-198	0.807	0.217
ANXA2	9	1.32221841	3.28E-180	5.99E-176	0.995	0.851
TGFBI	9	1.31296082	9.77E-74	1.78E-69	0.755	0.406
THY1	9	1.30216542	1.79E-166	3.27E-162	0.932	0.415
COL1A1	9	1.29634639	3.84E-84	7.00E-80	0.285	0.053
SPARC	9	1.26061618	9.52E-135	1.74E-130	0.948	0.584
LUM	9	1.25335098	5.85E-129	1.07E-124	1	0.999
PPIB	9	1.21271422	2.01E-153	3.67E-149	1	0.893
CLEC11A	9	1.15810144	4.65E-114	8.49E-110	0.82	0.443
PKM	9	1.14451035	4.49E-155	8.19E-151	1	0.924
HSPA5	9	1.1431935	2.27E-124	4.15E-120	0.883	0.458
CA9	9	1.11409194	4.37E-145	7.97E-141	0.846	0.347
PDIA3	9	1.11255273	3.06E-161	5.58E-157	0.953	0.572
TPM1	9	1.10897314	1.17E-89	2.14E-85	0.901	0.608
RPN2	9	1.09232413	9.38E-217	1.71E-212	0.883	0.285
CALR	9	1.07308851	3.81E-124	6.96E-120	0.948	0.663
COL2A1	9	1.06243528	3.21E-42	5.86E-38	0.82	0.676
PRSS23	9	1.06021124	4.37E-86	7.97E-82	0.728	0.337
ENO1	9	1.05781217	7.29E-159	1.33E-154	1	0.983
LECT1	9	1.05159795	2.56E-42	4.67E-38	0.846	0.776
IGFBP7	9	1.05032798	1.24E-134	2.27E-130	0.99	0.848

HSP90B1	9	1.04860852	2.55E-121	4.65E-117	0.979	0.78
S100A4	9	1.03934905	2.82E-89	5.14E-85	0.961	0.769
LOX	9	1.0161591	5.35E-180	9.77E-176	0.817	0.239
ITGB1	9	1.01228031	4.31E-130	7.87E-126	0.914	0.512
PGK1	9	1.00477294	6.00E-145	1.09E-140	0.992	0.908
LGALS1	9	1.003773	8.21E-98	1.50E-93	1	0.972
C1R	9	1.00023272	4.82E-169	8.80E-165	0.778	0.246
PLPP1	9	0.97651117	5.51E-119	1.01E-114	0.728	0.266
RCN3	9	0.97166418	1.53E-155	2.79E-151	0.872	0.364
COL6A2	9	0.95816482	2.44E-115	4.45E-111	0.948	0.61
PDIA6	9	0.9496385	1.51E-132	2.75E-128	0.969	0.661
PPIC	9	0.94323671	2.72E-129	4.96E-125	0.911	0.463
SCRG1	9	0.9412399	2.48E-79	4.52E-75	0.987	0.975
EFEMP2	9	0.94095148	6.76E-168	1.23E-163	0.922	0.411
PRDX4	9	0.93109269	4.33E-138	7.89E-134	0.977	0.757
P4HB	9	0.92382557	6.28E-120	1.15E-115	0.99	0.811
GRN	9	0.92198316	4.05E-167	7.40E-163	0.885	0.342
COL5A2	9	0.91863754	3.04E-127	5.55E-123	0.88	0.398
TUBA1B	9	0.91334153	4.35E-73	7.94E-69	0.7	0.343
P4HA2	9	0.89072144	1.25E-139	2.28E-135	0.862	0.388
SERPINA5	9	0.88761355	2.16E-79	3.95E-75	0.838	0.521
CYP1B1	9	0.8875924	1.75E-51	3.18E-47	0.405	0.144
ANGPTL4	9	0.88486816	8.20E-72	1.50E-67	0.564	0.212
CYTL1	9	0.87930223	9.59E-57	1.75E-52	0.499	0.193
ACTB	9	0.87442494	2.95E-70	5.38E-66	0.987	0.944
SERPINH1	9	0.86290134	3.48E-177	6.36E-173	0.645	0.144
HAPLN1	9	0.85776999	2.33E-76	4.26E-72	0.901	0.622
CTHRC1	9	0.85281808	1.46E-54	2.66E-50	0.582	0.271
TIMP1	9	0.85127395	1.39E-79	2.54E-75	0.997	0.997
PRDX1	9	0.84924221	1.55E-119	2.83E-115	0.979	0.827
COL6A1	9	0.84329881	1.51E-101	2.76E-97	0.869	0.466
CD63	9	0.84262554	3.41E-145	6.23E-141	1	0.999
ATP6AP2	9	0.83927228	8.12E-158	1.48E-153	0.862	0.338
MAGED1	9	0.83704951	4.45E-94	8.13E-90	0.668	0.245
CTSD	9	0.83083019	7.99E-69	1.46E-64	0.974	0.892
HTRA1	9	0.82799442	2.30E-112	4.19E-108	0.958	0.788
AEBP1	9	0.82048501	3.96E-78	7.22E-74	0.89	0.594
PLOD2	9	0.81525916	4.72E-111	8.61E-107	0.961	0.743
SERPINE2	9	0.80076998	3.89E-53	7.09E-49	1	0.995

LOXL3	9	0.80017938	2.21E-257	4.03E-253	0.734	0.133
CNN3	9	0.79482717	1.54E-137	2.81E-133	0.815	0.309
LMNA	9	0.79334994	1.58E-110	2.89E-106	0.77	0.311
CRYAB	9	0.7882128	8.52E-49	1.55E-44	0.93	0.754
COL9A3	9	0.783399	1.09E-27	1.99E-23	0.749	0.627
MAGED2	9	0.7797176	6.37E-107	1.16E-102	0.869	0.465
SMOC1	9	0.77920663	1.15E-111	2.11E-107	0.695	0.251
COMP	9	0.77438647	1.54E-39	2.81E-35	0.903	0.806
ID1	9	0.76815385	4.84E-50	8.84E-46	0.867	0.597
SAA1	9	0.76109357	1.88E-14	3.42E-10	0.159	0.063
FHL1	9	0.76043707	1.82E-39	3.32E-35	0.666	0.429
CALU	9	0.7592452	1.51E-94	2.76E-90	0.914	0.576
NUCB2	9	0.75807496	1.24E-82	2.26E-78	0.812	0.442
COL11A1	9	0.756791	9.32E-73	1.70E-68	0.95	0.715
TUBB2A	9	0.74975842	1.16E-97	2.11E-93	0.616	0.208
LAPTM4A	9	0.74327808	1.54E-122	2.80E-118	1	0.947
VKORC1	9	0.7426738	2.90E-111	5.29E-107	0.997	0.949
ITM2B	9	0.74217333	8.05E-117	1.47E-112	1	0.972
PDIA4	9	0.73744378	1.25E-125	2.29E-121	0.825	0.353
TPM2	9	0.73725097	3.04E-84	5.55E-80	0.903	0.563
ID3	9	0.71782399	4.12E-49	7.51E-45	0.843	0.565
FKBP10	9	0.7137458	7.39E-99	1.35E-94	0.864	0.469
S100A10	9	0.71363135	5.84E-86	1.06E-81	1	0.98
MYDGF	9	0.71250114	2.11E-86	3.84E-82	0.961	0.772
S100A11	9	0.70338198	2.29E-99	4.17E-95	0.997	0.917
ECM1	9	0.70016153	2.17E-187	3.96E-183	0.634	0.135
PDGFRL	9	0.69870045	1.88E-188	3.43E-184	0.637	0.132
TMED9	9	0.69738023	1.68E-95	3.06E-91	0.94	0.615
CFL1	9	0.69272678	3.72E-66	6.80E-62	0.948	0.768
ANXA1	9	0.69025999	4.81E-85	8.78E-81	0.961	0.733
ITM2C	9	0.68653707	6.61E-71	1.21E-66	0.88	0.545
TPI1	9	0.68238132	9.54E-122	1.74E-117	1	0.989
CD151	9	0.67835646	8.85E-78	1.62E-73	0.828	0.48
SDC2	9	0.67526548	1.16E-72	2.11E-68	0.956	0.825
FSTL1	9	0.67508792	1.17E-116	2.14E-112	0.807	0.338
RCN2	9	0.66454623	2.89E-87	5.27E-83	0.922	0.641
LMAN2	9	0.65810673	1.28E-102	2.34E-98	0.859	0.433
GGH	9	0.65437227	2.06E-178	3.75E-174	0.593	0.119
CTSA	9	0.64268201	1.29E-124	2.36E-120	0.684	0.222

C1S	9	0.64203276	2.40E-57	4.39E-53	0.851	0.613
SRGN	9	0.63305985	4.18E-63	7.63E-59	0.585	0.253
LMAN1	9	0.63305877	7.82E-89	1.43E-84	0.749	0.336
BSG	9	0.63287823	4.70E-93	8.57E-89	0.95	0.684
TMSB4X	9	0.62762121	1.63E-41	2.97E-37	0.922	0.767
DDOST	9	0.62713928	3.88E-98	7.08E-94	0.817	0.394
MATN4	9	0.62616489	5.71E-09	1.04E-04	0.292	0.184
ISLR	9	0.62539538	1.17E-70	2.14E-66	0.843	0.466
SLC16A3	9	0.6240932	1.03E-80	1.88E-76	0.843	0.464
RTN4	9	0.62331263	8.47E-69	1.54E-64	0.909	0.633
FDPS	9	0.62271192	8.72E-66	1.59E-61	0.559	0.225
COL9A2	9	0.62205571	3.29E-44	6.01E-40	0.653	0.368
TMBIM6	9	0.61902825	1.61E-73	2.94E-69	0.922	0.693
HLA-A	9	0.61826427	1.09E-84	1.99E-80	0.995	0.885
CD59	9	0.61462107	7.25E-77	1.32E-72	0.984	0.939
MSMO1	9	0.61148298	6.23E-50	1.14E-45	0.58	0.275
ITM2A	9	0.60719034	3.05E-46	5.56E-42	0.794	0.517
LAMP1	9	0.60417048	2.89E-86	5.27E-82	0.82	0.431
TSPAN4	9	0.60358318	3.28E-81	5.98E-77	0.851	0.468
P3H1	9	0.60246779	2.33E-207	4.25E-203	0.606	0.107
MYL6	9	0.60011366	1.51E-74	2.76E-70	0.984	0.929
NNMT	9	0.59981818	2.23E-45	4.07E-41	0.966	0.885
P4HA1	9	0.59967065	2.90E-72	5.29E-68	0.914	0.657
VIM	9	0.59118928	9.45E-44	1.73E-39	1	0.999
INSIG1	9	0.58949783	3.34E-31	6.10E-27	0.653	0.415
GSN	9	0.58799482	1.77E-68	3.22E-64	0.856	0.501
DDIT4	9	0.58624296	9.58E-11	1.75E-06	0.582	0.469
POLR2L	9	0.58180459	4.25E-76	7.76E-72	0.99	0.854
IGFBP3	9	0.57994581	2.25E-18	4.11E-14	0.499	0.309
FKBP11	9	0.57874575	5.04E-99	9.19E-95	0.603	0.198
CKAP4	9	0.57106346	7.61E-71	1.39E-66	0.676	0.307
THBS1	9	0.57005255	6.61E-89	1.21E-84	0.402	0.096
HLA-C	9	0.57003828	8.41E-71	1.54E-66	0.984	0.87
OSTC	9	0.5687268	5.51E-72	1.01E-67	0.956	0.805
PAPSS1	9	0.56841941	6.19E-86	1.13E-81	0.663	0.268
POSTN	9	0.56694883	2.07E-44	3.77E-40	0.157	0.029
MYL12B	9	0.56628009	8.15E-66	1.49E-61	0.872	0.603
LAMP2	9	0.56611734	1.50E-78	2.75E-74	0.825	0.451
CALD1	9	0.5646103	8.50E-51	1.55E-46	0.838	0.556

PFN1	9	0.56390716	2.65E-50	4.84E-46	0.966	0.877
LOXL2	9	0.56326071	4.93E-173	8.99E-169	0.533	0.096
LSP1	9	0.56289758	8.16E-80	1.49E-75	0.749	0.331
PRKCDBP	9	0.56012959	2.23E-65	4.08E-61	0.812	0.477
PLD3	9	0.55541664	2.52E-62	4.60E-58	0.836	0.52
COL8A1	9	0.552282	3.25E-100	5.93E-96	0.334	0.061
SNAPC1	9	0.55184592	1.02E-49	1.86E-45	0.768	0.466
PGAM1	9	0.55101854	2.95E-68	5.39E-64	0.953	0.782
PRELP	9	0.54953702	2.72E-34	4.97E-30	0.963	0.925
CRIP1	9	0.5440258	4.25E-48	7.76E-44	0.642	0.331
TUBB2B	9	0.5435861	1.63E-10	2.98E-06	0.522	0.417
ARF4	9	0.542646	1.53E-64	2.79E-60	0.922	0.723
OAT	9	0.54124662	2.12E-93	3.86E-89	0.742	0.304
ERP44	9	0.53845576	2.88E-100	5.25E-96	0.71	0.269
LRPAP1	9	0.53780886	1.47E-66	2.69E-62	0.833	0.514
PCOLCE2	9	0.53534259	4.87E-57	8.89E-53	0.982	0.903
SIL1	9	0.53332484	6.47E-83	1.18E-78	0.697	0.292
C5orf15	9	0.53325624	7.16E-87	1.31E-82	0.794	0.375
MORF4L2	9	0.53052558	4.87E-57	8.88E-53	0.885	0.646
KDELR3	9	0.52977894	8.93E-82	1.63E-77	0.679	0.271
CITED2	9	0.52751108	3.87E-18	7.06E-14	0.614	0.426
APLP2	9	0.5255246	4.33E-68	7.90E-64	0.71	0.353
HLA-B	9	0.52181109	2.22E-46	4.04E-42	0.916	0.774
SERF2	9	0.52103338	1.09E-78	1.98E-74	1	1
DOK1	9	0.52047417	1.86E-68	3.39E-64	0.525	0.187
MFGE8	9	0.5188582	1.66E-29	3.03E-25	0.919	0.822
ATP5J2	9	0.51823849	7.27E-60	1.33E-55	0.822	0.534
PSMA4	9	0.51542795	6.54E-61	1.19E-56	0.721	0.399
RCAN1	9	0.51449302	1.62E-62	2.96E-58	0.345	0.092
PPIA	9	0.51431267	4.44E-69	8.10E-65	0.997	0.959
SCARB2	9	0.51389104	4.94E-66	9.01E-62	0.789	0.45
COL6A3	9	0.51262715	7.91E-46	1.44E-41	0.653	0.342
IGFBP6	9	0.51184978	8.57E-34	1.56E-29	0.849	0.641
PLOD1	9	0.51167862	1.27E-64	2.31E-60	0.734	0.384
RCN1	9	0.51140628	2.05E-58	3.75E-54	0.765	0.44
HERPUD1	9	0.50900801	1.48E-42	2.70E-38	0.755	0.484
CD99	9	0.50826292	4.99E-68	9.10E-64	0.992	0.924
MDH1	9	0.50715739	7.68E-85	1.40E-80	0.692	0.287
P3H2	9	0.50661283	3.81E-64	6.94E-60	0.603	0.255

CRELD2	9	0.50619694	6.41E-88	1.17E-83	0.627	0.22
SSR4	9	0.50454308	5.73E-73	1.04E-68	0.997	0.958
SQLE	9	0.50389408	8.50E-61	1.55E-56	0.522	0.203
SNAI2	9	0.50238488	6.75E-38	1.23E-33	0.781	0.5
PRDX2	9	0.50055244	4.48E-53	8.18E-49	0.911	0.702
TMED10	9	0.49981646	5.59E-68	1.02E-63	0.969	0.868
TMSB10	9	0.49758232	4.38E-36	7.99E-32	0.995	0.988
NREP	9	0.4958283	4.40E-71	8.03E-67	0.627	0.247
CCPG1	9	0.49526845	5.31E-49	9.68E-45	0.773	0.477
CAPG	9	0.49431729	8.41E-44	1.53E-39	0.661	0.377
YIF1A	9	0.49398626	2.42E-55	4.41E-51	0.755	0.428
SGCB	9	0.4931985	2.19E-57	3.99E-53	0.739	0.411
KDELR2	9	0.49113048	1.21E-52	2.21E-48	0.969	0.856
CD164	9	0.49075605	1.66E-64	3.02E-60	0.916	0.671
CYBA	9	0.49044221	1.67E-44	3.04E-40	0.794	0.558
DNAJB11	9	0.48999234	5.52E-63	1.01E-58	0.569	0.239
GPX8	9	0.48842541	1.27E-62	2.31E-58	0.634	0.289
ATP5B	9	0.48637181	5.12E-58	9.34E-54	0.755	0.419
LINC00152	9	0.48539753	1.94E-38	3.53E-34	0.642	0.348
TMEM59	9	0.48502788	4.12E-70	7.51E-66	0.997	0.97
P3H4	9	0.47989847	3.64E-97	6.64E-93	0.572	0.173
AP2M1	9	0.47851845	1.96E-51	3.58E-47	0.849	0.548
HEXB	9	0.47687707	1.70E-58	3.10E-54	0.796	0.451
RNH1	9	0.47540391	1.36E-44	2.49E-40	0.807	0.532
RABAC1	9	0.47527432	1.23E-58	2.24E-54	0.937	0.823
PSMB3	9	0.47518375	5.23E-71	9.54E-67	0.744	0.356
COL11A2	9	0.47293189	1.16E-20	2.11E-16	0.642	0.498
NME1	9	0.47051643	9.36E-60	1.71E-55	0.608	0.268
RBX1	9	0.46882354	6.25E-53	1.14E-48	0.838	0.568
MYL12A	9	0.46580936	4.38E-46	7.99E-42	0.815	0.552
TNC	9	0.46489668	3.20E-69	5.84E-65	0.345	0.086
ZFP36L1	9	0.46124797	3.13E-32	5.71E-28	0.627	0.363
TMED3	9	0.46078392	2.42E-55	4.42E-51	0.828	0.49
FAP	9	0.45674469	7.36E-61	1.34E-56	0.572	0.232
ATP1B3	9	0.45531632	7.11E-59	1.30E-54	0.655	0.308
PLOD3	9	0.45504894	7.23E-94	1.32E-89	0.564	0.174
FSCN1	9	0.45427129	8.38E-93	1.53E-88	0.522	0.152
VCAN	9	0.44923025	1.69E-32	3.08E-28	0.7	0.43
TMED2	9	0.44780665	1.47E-56	2.67E-52	0.961	0.823

LOXL1	9	0.44519959	1.07E-98	1.94E-94	0.347	0.065
MGST3	9	0.44330415	4.17E-53	7.61E-49	0.903	0.654
PIGT	9	0.44304402	1.09E-77	1.99E-73	0.653	0.254
ITGBL1	9	0.44286771	1.46E-88	2.66E-84	0.46	0.123
PTTG1IP	9	0.44242547	2.32E-39	4.22E-35	0.734	0.467
NOV	9	0.44013254	1.41E-31	2.58E-27	0.201	0.058
COL5A1	9	0.43862326	6.63E-86	1.21E-81	0.556	0.173
EMILIN1	9	0.43829483	9.70E-94	1.77E-89	0.527	0.151
IGFBP4	9	0.43479411	2.48E-42	4.52E-38	0.373	0.133
CPQ	9	0.43350077	2.05E-50	3.74E-46	0.611	0.308
IFITM3	9	0.43134791	9.07E-42	1.66E-37	0.956	0.868
ARPC1B	9	0.43059038	4.42E-38	8.07E-34	0.744	0.471
PDIA5	9	0.42977762	1.13E-134	2.06E-130	0.415	0.073
RRBP1	9	0.42922589	6.18E-49	1.13E-44	0.739	0.41
TPM4	9	0.42896612	6.62E-45	1.21E-40	0.574	0.282
ANGPTL5	9	0.4279155	1.53E-39	2.79E-35	0.538	0.269
DAD1	9	0.42785804	6.04E-49	1.10E-44	0.903	0.709
ATRAID	9	0.42722763	6.63E-47	1.21E-42	0.961	0.833
TSPO	9	0.42406755	4.49E-45	8.20E-41	0.956	0.817
CTSK	9	0.42359838	3.82E-51	6.97E-47	0.611	0.29
DKK3	9	0.42337301	6.93E-68	1.26E-63	0.493	0.165
SMOC2	9	0.42288935	3.07E-52	5.60E-48	0.366	0.116
CPE	9	0.42227266	2.24E-20	4.08E-16	0.778	0.657
HCFC1R1	9	0.42155892	8.00E-39	1.46E-34	0.909	0.665
EIF5A	9	0.42090615	8.30E-42	1.51E-37	0.812	0.559
DSTN	9	0.42054087	1.40E-48	2.55E-44	1	0.997
MANF	9	0.41931345	7.27E-42	1.33E-37	0.619	0.328
DPT	9	0.41896294	5.81E-97	1.06E-92	0.326	0.06
FAM114A1	9	0.41709311	9.01E-42	1.64E-37	0.611	0.332
SEC13	9	0.4167692	9.86E-81	1.80E-76	0.548	0.182
ENG	9	0.41482187	2.13E-105	3.89E-101	0.431	0.094
RBP4	9	0.41460502	5.34E-24	9.74E-20	0.883	0.735
CNPY2	9	0.41100421	1.68E-41	3.07E-37	0.883	0.638
CFD	9	0.41055426	1.24E-13	2.26E-09	0.256	0.131
PIGK	9	0.41005854	4.17E-75	7.61E-71	0.574	0.206
ATPIF1	9	0.40917136	6.34E-38	1.16E-33	0.862	0.68
TRAPPC1	9	0.40847615	1.82E-52	3.31E-48	0.796	0.445
NQO1	9	0.4080931	5.34E-46	9.74E-42	0.311	0.096
C2orf40	9	0.40735259	1.31E-12	2.39E-08	0.937	0.967

TALDO1	9	0.40728328	5.69E-43	1.04E-38	0.778	0.477
TUBB	9	0.40535831	6.23E-17	1.14E-12	0.896	0.798
DCN	9	0.40534937	5.64E-21	1.03E-16	1	0.998
HIF1A	9	0.40489401	1.20E-27	2.18E-23	0.919	0.807
MXRA8	9	0.40450733	1.49E-55	2.72E-51	0.564	0.241
PPP1R3C	9	0.40337785	3.30E-23	6.03E-19	0.629	0.426
IDI1	9	0.40180246	8.28E-27	1.51E-22	0.514	0.292
CYB5R3	9	0.4008989	1.92E-41	3.50E-37	0.88	0.651
FBXO2	9	0.40082805	5.51E-17	1.01E-12	0.807	0.74
TUBA1A	9	0.39784535	6.72E-37	1.23E-32	0.426	0.184
MARCKS	9	0.39583767	2.44E-53	4.45E-49	0.376	0.12
NDUFA13	9	0.39516984	8.91E-42	1.63E-37	0.849	0.625
ARL6IP5	9	0.39437411	8.20E-40	1.50E-35	0.836	0.596
FKBP7	9	0.39419466	2.92E-56	5.33E-52	0.538	0.22
EIF4A1	9	0.39408774	3.16E-33	5.76E-29	0.614	0.366
PTRF	9	0.39337967	3.99E-34	7.28E-30	0.76	0.494
GPAA1	9	0.39289076	8.25E-47	1.51E-42	0.658	0.356
HNRNPA2B1	9	0.39285958	1.94E-37	3.55E-33	0.82	0.567
TECR	9	0.39238851	1.17E-42	2.14E-38	0.676	0.389
NUCB1	9	0.39150462	6.97E-41	1.27E-36	0.833	0.566
IFI6	9	0.39127921	3.31E-31	6.03E-27	0.58	0.318
HSPA8	9	0.39071329	9.71E-24	1.77E-19	0.666	0.454
NDN	9	0.3900415	3.65E-54	6.66E-50	0.634	0.299
IFITM10	9	0.38853488	2.66E-58	4.86E-54	0.543	0.214
CD9	9	0.38824879	4.37E-27	7.98E-23	0.755	0.513
CALM2	9	0.38821424	1.62E-42	2.96E-38	0.995	0.967
SCPEP1	9	0.38794889	2.08E-58	3.79E-54	0.58	0.245
AXL	9	0.38784822	3.33E-23	6.08E-19	0.559	0.337
GAPDH	9	0.38759285	1.12E-57	2.04E-53	1	1
HNRNPK	9	0.38688885	4.40E-43	8.04E-39	0.924	0.722
ACADVL	9	0.38584844	1.35E-50	2.46E-46	0.603	0.283
TM9SF2	9	0.38375236	5.17E-51	9.43E-47	0.582	0.274
PSMB1	9	0.38337917	8.25E-44	1.50E-39	0.901	0.69
FIBIN	9	0.3827324	9.98E-69	1.82E-64	0.339	0.084
MALL	9	0.380998	7.91E-35	1.44E-30	0.538	0.268
MIR4435-2HG	9	0.3806803	1.07E-45	1.95E-41	0.525	0.226
DNM3OS	9	0.38059614	8.29E-38	1.51E-33	0.655	0.358
PLAC9	9	0.37947509	6.73E-21	1.23E-16	0.922	0.798
C12orf75	9	0.37947429	1.39E-45	2.53E-41	0.439	0.166

YWHAB	9	0.379088	1.80E-36	3.28E-32	0.65	0.386
WBP5	9	0.37778846	2.72E-36	4.96E-32	0.922	0.742
PTN	9	0.3760107	1.74E-127	3.17E-123	0.319	0.044
SPPL2A	9	0.37582982	2.58E-55	4.71E-51	0.645	0.3
HSP90AA1	9	0.37338235	1.53E-27	2.78E-23	0.875	0.744
LGMN	9	0.37226837	4.68E-72	8.54E-68	0.493	0.16
CHID1	9	0.37189078	2.38E-39	4.35E-35	0.809	0.538
PSMB6	9	0.37035921	2.72E-37	4.96E-33	0.755	0.485
SRSF2	9	0.37028741	1.66E-34	3.03E-30	0.671	0.414
UGP2	9	0.36962073	1.82E-28	3.32E-24	0.807	0.619
PHGDH	9	0.36905504	1.07E-43	1.94E-39	0.352	0.124
TUSC3	9	0.36721736	1.52E-42	2.77E-38	0.501	0.229
IKBIP	9	0.3665808	6.25E-50	1.14E-45	0.585	0.265
STMN1	9	0.36487299	1.89E-22	3.45E-18	0.206	0.074
COPE	9	0.36483046	1.23E-36	2.25E-32	0.809	0.557
SORBS2	9	0.36363631	8.85E-17	1.62E-12	0.24	0.11
ATP5G1	9	0.36202117	9.27E-39	1.69E-34	0.7	0.424
MYL9	9	0.36193872	2.93E-48	5.35E-44	0.512	0.216
MLEC	9	0.36152602	3.18E-39	5.80E-35	0.681	0.396
VCP	9	0.36111739	2.87E-49	5.24E-45	0.501	0.209
SSR1	9	0.36024625	1.06E-32	1.93E-28	0.77	0.513
DUSP14	9	0.3597761	9.65E-49	1.76E-44	0.473	0.188
SRM	9	0.35976937	1.71E-31	3.11E-27	0.608	0.367
PPP1CA	9	0.35916782	3.75E-44	6.84E-40	0.608	0.305
FDFT1	9	0.35910699	1.79E-30	3.26E-26	0.499	0.263
FBLN1	9	0.35908748	9.15E-146	1.67E-141	0.308	0.037
SERPING1	9	0.35842374	5.52E-18	1.01E-13	0.684	0.53
RPN1	9	0.35790335	2.38E-57	4.34E-53	0.52	0.202
EXT2	9	0.3577441	1.70E-66	3.09E-62	0.473	0.156
BCAP31	9	0.35753419	4.51E-41	8.23E-37	0.715	0.422
MRPL13	9	0.35714268	4.95E-93	9.03E-89	0.47	0.122
PDGFRA	9	0.35617773	2.84E-43	5.17E-39	0.567	0.263
P3H3	9	0.35602223	6.14E-56	1.12E-51	0.493	0.189
QSOX1	9	0.35588519	6.03E-45	1.10E-40	0.642	0.321
COPB2	9	0.35575077	1.52E-57	2.77E-53	0.54	0.216
NDUFB2	9	0.35347138	2.30E-37	4.20E-33	0.922	0.768
ARPC3	9	0.3528322	3.45E-37	6.30E-33	0.909	0.727
SMPD1	9	0.35278825	2.33E-66	4.26E-62	0.488	0.164
MTDH	9	0.35222064	4.83E-32	8.82E-28	0.849	0.648

SRPX2	9	0.35221496	7.87E-80	1.44E-75	0.392	0.098
FCGRT	9	0.35148569	2.18E-29	3.97E-25	0.796	0.568
CBR1	9	0.35144687	1.72E-38	3.15E-34	0.556	0.284
DBI	9	0.35133347	1.46E-23	2.66E-19	0.862	0.735
MT-CYB	9	0.35130459	9.83E-22	1.79E-17	0.903	0.717
UXS1	9	0.34911785	6.32E-60	1.15E-55	0.514	0.191
RRAGA	9	0.34834357	3.34E-43	6.09E-39	0.653	0.344
ANXA5	9	0.3480214	2.27E-33	4.14E-29	0.945	0.821
FUCA2	9	0.34781199	2.83E-47	5.16E-43	0.493	0.208
HMGB1	9	0.34726868	9.24E-22	1.69E-17	0.89	0.763
INHBA	9	0.34624777	3.72E-20	6.79E-16	0.595	0.383
GSTP1	9	0.34464055	6.91E-36	1.26E-31	0.984	0.954
TMEM30A	9	0.34442	2.13E-38	3.89E-34	0.689	0.405
TXNDC15	9	0.34430545	2.82E-41	5.15E-37	0.627	0.326
TBL1XR1	9	0.34289371	1.04E-28	1.90E-24	0.614	0.367
HNRNPH2	9	0.34289031	1.86E-52	3.39E-48	0.517	0.212
ATP6AP1	9	0.34266516	6.37E-52	1.16E-47	0.548	0.232
ID4	9	0.34265848	8.37E-13	1.53E-08	0.695	0.542
TUBB4B	9	0.34202958	3.21E-13	5.85E-09	0.59	0.44
HEXA	9	0.34182597	1.94E-48	3.53E-44	0.621	0.299
FMOD	9	0.34036346	3.89E-19	7.09E-15	0.94	0.887
GNG11	9	0.34029302	1.05E-26	1.93E-22	0.499	0.269
TXN	9	0.33977583	5.19E-23	9.48E-19	0.927	0.845
44446	9	0.33740019	2.31E-31	4.22E-27	0.789	0.537
PPT1	9	0.33736547	1.25E-37	2.29E-33	0.687	0.397
HMOX1	9	0.33724881	8.77E-15	1.60E-10	0.337	0.189
LY6E	9	0.33712489	2.56E-30	4.68E-26	0.723	0.467
C4orf48	9	0.33694669	1.79E-29	3.27E-25	0.616	0.361
FHL2	9	0.33613836	1.07E-28	1.95E-24	0.41	0.191
RDX	9	0.33577574	3.64E-27	6.64E-23	0.708	0.485
SEPT2	9	0.33559135	1.27E-38	2.31E-34	0.614	0.333
NT5E	9	0.33552108	1.31E-29	2.38E-25	0.624	0.359
SCD	9	0.33549006	3.90E-27	7.12E-23	0.355	0.157
CETN2	9	0.3354317	1.92E-41	3.50E-37	0.608	0.315
TMEM45A	9	0.33436357	5.23E-29	9.55E-25	0.943	0.811
EMC7	9	0.33297303	2.76E-36	5.04E-32	0.671	0.394
DNASE2	9	0.3321542	9.79E-55	1.79E-50	0.491	0.187
ALDOA	9	0.33206334	2.40E-40	4.38E-36	0.997	0.995
CLIC3	9	0.33148016	3.33E-33	6.07E-29	0.381	0.16

PSAT1	9	0.33062256	3.98E-50	7.25E-46	0.225	0.051
CAPZB	9	0.3303814	1.02E-33	1.86E-29	0.692	0.427
FKBP1A	9	0.32976455	5.26E-33	9.60E-29	0.744	0.488
PODNL1	9	0.32965903	8.68E-43	1.58E-38	0.587	0.283
XRCC6	9	0.32945223	4.26E-35	7.78E-31	0.642	0.365
MAP1B	9	0.32868291	1.94E-47	3.54E-43	0.355	0.115
WISP3	9	0.32819824	3.78E-57	6.90E-53	0.517	0.197
SIX1	9	0.32814411	4.20E-36	7.67E-32	0.386	0.155
C1GALT1C1	9	0.32752793	4.65E-117	8.49E-113	0.371	0.065
MT-CO3	9	0.32654392	3.56E-22	6.49E-18	0.969	0.885
GLT8D1	9	0.32646014	2.33E-56	4.25E-52	0.483	0.179
CFH	9	0.32627637	2.91E-25	5.32E-21	0.418	0.207
EDARADD	9	0.32608146	2.64E-74	4.82E-70	0.462	0.136
TNFRSF1A	9	0.32589436	1.48E-26	2.70E-22	0.64	0.399
HHIPL2	9	0.32557073	3.11E-60	5.68E-56	0.413	0.129
PSMB5	9	0.32387592	1.53E-27	2.79E-23	0.791	0.588
PRG4	9	0.32354949	1.88E-18	3.43E-14	0.514	0.312
PYCR1	9	0.32312486	1.19E-54	2.17E-50	0.431	0.148
GADD45B	9	0.32299554	3.30E-12	6.02E-08	0.418	0.266
ATP5G3	9	0.32176084	1.49E-27	2.72E-23	0.843	0.686
LDHA	9	0.32075031	1.24E-27	2.26E-23	1	0.998
DDX5	9	0.32014982	6.69E-23	1.22E-18	0.755	0.583
AP2S1	9	0.31931424	7.53E-24	1.37E-19	0.734	0.533
EHD2	9	0.31930032	5.33E-50	9.73E-46	0.514	0.208
DRAP1	9	0.31798049	3.92E-23	7.15E-19	0.781	0.586
BGN	9	0.31753736	4.18E-31	7.63E-27	0.995	0.984
HACD3	9	0.3162883	2.00E-48	3.65E-44	0.499	0.203
ILK	9	0.31625234	1.29E-36	2.36E-32	0.564	0.295
LTBP1	9	0.31588043	7.82E-41	1.43E-36	0.379	0.14
ECM2	9	0.31540461	1.81E-38	3.29E-34	0.491	0.222
EIF6	9	0.31478028	2.36E-33	4.30E-29	0.58	0.315
SLC35B2	9	0.3145023	3.76E-49	6.86E-45	0.517	0.215
TMEM165	9	0.31413783	8.26E-38	1.51E-33	0.645	0.351
YIPF3	9	0.31402184	9.81E-34	1.79E-29	0.728	0.456
OSBPL9	9	0.31353628	5.10E-30	9.30E-26	0.486	0.246
GPC6	9	0.31342777	1.20E-34	2.18E-30	0.554	0.288
SLC39A7	9	0.31329508	2.47E-44	4.50E-40	0.488	0.208
ENPP1	9	0.31271074	9.38E-20	1.71E-15	0.517	0.312
FKBP2	9	0.31200881	1.31E-31	2.39E-27	0.893	0.659

HSPB1	9	0.31094254	3.63E-16	6.63E-12	0.809	0.706
CALM1	9	0.31013178	2.08E-17	3.79E-13	0.721	0.544
C1orf122	9	0.30860272	2.54E-31	4.63E-27	0.666	0.398
MESDC2	9	0.30849282	1.92E-32	3.51E-28	0.637	0.37
RHOA	9	0.30828979	1.45E-29	2.65E-25	0.924	0.838
IGFBP5	9	0.3082245	1.05E-14	1.91E-10	0.339	0.184
GTF2H5	9	0.30726863	5.58E-24	1.02E-19	0.789	0.609
SDHC	9	0.30665028	4.54E-40	8.28E-36	0.585	0.297
OS9	9	0.30638511	2.66E-25	4.86E-21	0.684	0.452
DEGS1	9	0.30453952	2.73E-47	4.98E-43	0.446	0.173
CD320	9	0.30347891	2.46E-31	4.49E-27	0.731	0.462
YWHAQ	9	0.30328323	2.64E-24	4.81E-20	0.885	0.699
PSAP	9	0.30300043	6.07E-17	1.11E-12	0.95	0.894
JKAMP	9	0.30238153	1.00E-36	1.83E-32	0.569	0.29
CERCAM	9	0.30107342	7.21E-72	1.32E-67	0.381	0.099
OMD	9	0.30101258	2.70E-19	4.93E-15	0.303	0.144
PRCP	9	0.30046916	3.47E-45	6.34E-41	0.567	0.251
SLC1A5	9	0.30045764	1.25E-23	2.29E-19	0.462	0.261
CD47	9	0.30020358	1.90E-27	3.47E-23	0.697	0.454
MGLL	9	0.30006033	2.22E-26	4.05E-22	0.352	0.156
SEPT11	9	0.29945954	1.50E-46	2.74E-42	0.418	0.154
YIF1B	9	0.2989705	3.29E-38	6.01E-34	0.449	0.195
COMT	9	0.29881912	2.51E-28	4.58E-24	0.728	0.485
EID1	9	0.29800625	1.31E-30	2.40E-26	0.982	0.92
SSR2	9	0.29747812	3.12E-17	5.69E-13	0.927	0.848
PSMD8	9	0.29743848	5.20E-25	9.49E-21	0.742	0.529
PAM	9	0.2973174	3.33E-23	6.08E-19	0.77	0.572
MDFI	9	0.29703947	2.93E-17	5.35E-13	0.681	0.503
PLIN3	9	0.29611586	3.76E-35	6.87E-31	0.486	0.232
SURF4	9	0.29359143	8.47E-35	1.55E-30	0.52	0.258
SEPT15	9	0.29295767	5.91E-26	1.08E-21	0.663	0.434
SDF4	9	0.29248177	1.86E-28	3.40E-24	0.877	0.65
C7orf73	9	0.29201283	4.06E-29	7.40E-25	0.919	0.742
ANXA6	9	0.29199911	2.66E-57	4.86E-53	0.42	0.137
ITFG1	9	0.29161554	6.05E-39	1.10E-34	0.551	0.263
CXXC5	9	0.28998518	1.13E-23	2.06E-19	0.585	0.346
SERPINE1	9	0.28963327	4.51E-07	8.23E-03	0.258	0.162
ASPH	9	0.28910605	2.23E-28	4.07E-24	0.601	0.346
TMX1	9	0.28871502	5.11E-29	9.32E-25	0.705	0.449

SEC23A	9	0.2864053	1.37E-28	2.49E-24	0.619	0.363
SH3BGRL3	9	0.28614179	1.69E-10	3.09E-06	0.995	0.978
DPP7	9	0.28586714	4.84E-20	8.84E-16	0.762	0.589
RANBP1	9	0.28437423	1.38E-21	2.52E-17	0.752	0.541
MLF2	9	0.28426608	1.05E-24	1.91E-20	0.676	0.448
PSMB2	9	0.283976	3.07E-28	5.60E-24	0.653	0.402
HSPA9	9	0.28356729	7.67E-33	1.40E-28	0.499	0.242
GPC1	9	0.28343148	1.65E-33	3.00E-29	0.426	0.19
ARPC5	9	0.28305863	1.08E-23	1.97E-19	0.749	0.55
FBN1	9	0.28121867	6.59E-32	1.20E-27	0.347	0.141
MT-ND3	9	0.28115822	1.15E-15	2.10E-11	0.88	0.694
COPZ2	9	0.28085695	8.08E-28	1.47E-23	0.52	0.28
TCTN1	9	0.28078019	6.99E-36	1.28E-31	0.509	0.244
TRIP6	9	0.28036252	6.71E-33	1.22E-28	0.525	0.266
C9orf3	9	0.28020793	4.22E-26	7.70E-22	0.648	0.398
CNPY4	9	0.27958656	4.89E-79	8.92E-75	0.394	0.097
UBC	9	0.27943531	5.70E-20	1.04E-15	0.966	0.933
TNFRSF12A	9	0.2792071	1.05E-13	1.92E-09	0.601	0.411
SYNGR1	9	0.27895284	2.56E-58	4.67E-54	0.363	0.104
ACAT2	9	0.27875003	3.69E-20	6.73E-16	0.373	0.195
APP	9	0.27855419	1.51E-24	2.75E-20	0.601	0.37
NT5DC2	9	0.27769392	1.25E-46	2.28E-42	0.371	0.124
PHLDA2	9	0.27579133	2.89E-19	5.27E-15	0.332	0.165
ERP29	9	0.27543914	2.23E-20	4.08E-16	0.862	0.72
ERGIC3	9	0.27525566	1.36E-22	2.48E-18	0.927	0.804
NDUFA4L2	9	0.27499964	3.94E-30	7.20E-26	1	0.999
ERLEC1	9	0.2744046	2.59E-26	4.72E-22	0.689	0.442
NDUFA12	9	0.27405241	1.36E-28	2.49E-24	0.577	0.328
FBLN2	9	0.27399446	3.47E-71	6.34E-67	0.235	0.042
TMED7	9	0.27362338	1.96E-26	3.58E-22	0.65	0.403
GNL3	9	0.27184854	5.03E-39	9.18E-35	0.514	0.235
SEC61B	9	0.27158747	6.72E-24	1.23E-19	0.922	0.813
PRKCSH	9	0.27157033	8.64E-24	1.58E-19	0.624	0.393
ECH1	9	0.27091918	2.16E-28	3.93E-24	0.624	0.365
MFSD10	9	0.27089966	8.35E-33	1.52E-28	0.533	0.269
PLAU	9	0.26990421	1.08E-58	1.96E-54	0.117	0.013
TMEM230	9	0.26957469	7.99E-23	1.46E-18	0.869	0.707
GOLM1	9	0.26869277	2.55E-37	4.66E-33	0.496	0.227
GAS6	9	0.26861537	1.10E-14	2.01E-10	0.574	0.393

RAB2A	9	0.26849391	5.25E-24	9.59E-20	0.88	0.689
CKB	9	0.26830216	1.92E-14	3.50E-10	0.253	0.126
ARPC2	9	0.26823446	2.12E-17	3.86E-13	0.898	0.792
ERH	9	0.26791221	4.76E-22	8.69E-18	0.877	0.715
ATP5C1	9	0.26771624	3.16E-23	5.76E-19	0.705	0.485
NDUFA11	9	0.26739653	2.55E-25	4.66E-21	0.974	0.877
VWA1	9	0.26730688	4.63E-47	8.44E-43	0.379	0.128
FBLN7	9	0.265566	2.30E-35	4.20E-31	0.467	0.209
TPST1	9	0.26546415	1.64E-34	3.00E-30	0.418	0.182
HMGCS1	9	0.26521798	6.05E-22	1.10E-17	0.285	0.124
MXRA5	9	0.2647519	6.05E-76	1.10E-71	0.29	0.058
DYNLL1	9	0.26472344	7.66E-19	1.40E-14	0.898	0.847
SCP2	9	0.26470579	4.50E-26	8.21E-22	0.674	0.433
CHMP1B	9	0.26457867	8.18E-12	1.49E-07	0.371	0.232
GLT8D2	9	0.2626978	1.70E-63	3.10E-59	0.379	0.106
FKBP9	9	0.26230511	3.04E-29	5.56E-25	0.42	0.197
ITGA11	9	0.26170939	1.49E-41	2.71E-37	0.358	0.124
PDLIM2	9	0.26037071	6.11E-18	1.12E-13	0.684	0.508
GLB1	9	0.26002841	5.42E-82	9.89E-78	0.321	0.066
ID2	9	0.25960356	3.46E-08	6.31E-04	0.695	0.567
ARSA	9	0.25937463	2.24E-54	4.08E-50	0.394	0.125
HSD17B12	9	0.25916075	4.63E-30	8.45E-26	0.522	0.268
PRDX3	9	0.25893327	1.09E-22	1.98E-18	0.585	0.367
AKR7A2	9	0.25878468	9.70E-25	1.77E-20	0.624	0.39
WDR61	9	0.2586289	5.35E-34	9.76E-30	0.509	0.245
RP11-14N7.2	9	0.25857708	1.34E-38	2.45E-34	0.426	0.172
DPM1	9	0.2583618	6.09E-28	1.11E-23	0.559	0.311
SFPQ	9	0.25731438	6.74E-26	1.23E-21	0.52	0.283
C14orf119	9	0.25695679	3.22E-38	5.87E-34	0.47	0.204
SRSF11	9	0.25694508	1.73E-24	3.15E-20	0.723	0.481
MEG3	9	0.25659453	1.33E-13	2.43E-09	0.457	0.293
MT-ATP6	9	0.255857	9.46E-16	1.73E-11	0.945	0.813
PSMC2	9	0.25574535	1.37E-33	2.49E-29	0.452	0.206
B2M	9	0.2555891	7.90E-24	1.44E-19	1	0.991
AKR1A1	9	0.25531634	5.20E-24	9.50E-20	0.574	0.35
RAMP2	9	0.25509742	2.72E-40	4.97E-36	0.337	0.116
PSMA3	9	0.25496628	2.14E-27	3.91E-23	0.527	0.285
CTSB	9	0.25432395	2.58E-15	4.71E-11	0.742	0.585
YIPF5	9	0.25394933	1.08E-24	1.97E-20	0.593	0.359

UQCRQ	9	0.25307164	4.24E-19	7.73E-15	0.974	0.92
S100A6	9	0.25267676	8.75E-18	1.60E-13	1	1
NDUFA3	9	0.25248621	2.76E-21	5.03E-17	0.762	0.556
MAGEH1	9	0.25219768	6.60E-30	1.20E-25	0.441	0.21
CD46	9	0.25205273	3.70E-39	6.76E-35	0.431	0.176
MAP7D3	9	0.25160921	2.88E-22	5.25E-18	0.48	0.266
ANXA4	9	0.25139925	2.53E-20	4.61E-16	0.595	0.384
GJA1	9	0.25132442	1.19E-19	2.17E-15	0.379	0.198
SYPL1	9	0.25071652	2.52E-19	4.60E-15	0.807	0.625
SPTLC3	9	0.2504571	4.67E-19	8.53E-15	0.394	0.205
ALDOC	9	0.25043529	4.32E-23	7.89E-19	0.567	0.336
**gene**	**cluster**	**avg_logFC**	**p_val**	**p_val_adj**	**pct.1**	**pct.2**
PPP3CA	10	1.16514419	2.26E-114	4.13E-110	0.977	0.755
CTNNB1	10	1.15696994	3.27E-151	5.97E-147	0.909	0.39
PPP1CB	10	1.03291707	1.26E-132	2.30E-128	0.983	0.758
HNRNPH1	10	0.87321914	1.18E-143	2.16E-139	0.969	0.497
WTAP	10	0.79456585	3.45E-56	6.29E-52	0.906	0.684
SET	10	0.71884121	3.38E-109	6.18E-105	0.971	0.743
B3GNT7	10	0.66949141	3.63E-76	6.63E-72	0.729	0.319
GOLIM4	10	0.65872269	1.91E-69	3.48E-65	0.829	0.437
CDC42	10	0.6065014	2.58E-83	4.71E-79	0.943	0.649
TSPYL1	10	0.57613144	5.77E-70	1.05E-65	0.791	0.422
PHKG1	10	0.56039384	2.83E-100	5.17E-96	0.586	0.168
C1orf56	10	0.52506846	1.81E-70	3.30E-66	0.574	0.205
TRA2A	10	0.48496542	3.64E-68	6.64E-64	0.654	0.271
MDM4	10	0.44630936	3.86E-71	7.05E-67	0.643	0.247
VEGFA	10	0.43804227	1.10E-16	2.01E-12	0.72	0.582
EIF2S3	10	0.43387662	3.83E-41	6.98E-37	0.826	0.543
ARF6	10	0.4128256	2.95E-24	5.39E-20	0.717	0.554
CDC42SE1	10	0.40472776	5.42E-43	9.89E-39	0.554	0.258
MRPS6	10	0.40358401	4.50E-14	8.20E-10	0.943	0.911
UPP1	10	0.40018315	5.19E-23	9.48E-19	0.617	0.376
SEC22B	10	0.39980596	9.58E-40	1.75E-35	0.697	0.413
C11orf96	10	0.3971298	5.42E-17	9.89E-13	0.394	0.216
YWHAE	10	0.3922121	1.40E-43	2.56E-39	0.974	0.886
KLF3	10	0.37426839	2.81E-16	5.12E-12	0.454	0.283
HMGA1	10	0.37059516	4.80E-15	8.75E-11	0.566	0.385
EIF4A2	10	0.36710121	3.55E-39	6.48E-35	1	0.971
THAP5	10	0.36494436	7.80E-43	1.42E-38	0.58	0.28

CCDC85B	10	0.36226544	1.42E-13	2.59E-09	0.886	0.795
PPP1R15A	10	0.35702423	6.98E-13	1.27E-08	0.514	0.34
PTGS2	10	0.35230701	2.23E-15	4.07E-11	0.526	0.332
SLC25A37	10	0.35131457	6.57E-24	1.20E-19	0.897	0.751
INSR	10	0.35017752	1.34E-51	2.44E-47	0.506	0.193
PTPRS	10	0.34698	8.91E-58	1.63E-53	0.431	0.136
SUMF2	10	0.33410345	2.10E-17	3.82E-13	0.614	0.45
ERO1A	10	0.33331936	1.08E-17	1.96E-13	0.689	0.533
PRG4	10	0.32489725	9.22E-10	1.68E-05	0.469	0.313
ARRDC3	10	0.32341595	7.88E-14	1.44E-09	0.551	0.386
PAQR7	10	0.32245793	5.24E-82	9.56E-78	0.406	0.095
NRN1	10	0.31577218	9.33E-10	1.70E-05	0.611	0.458
SH3BP5	10	0.31471812	4.71E-17	8.59E-13	0.534	0.353
TMED4	10	0.31452865	5.43E-26	9.91E-22	0.654	0.422
RABGGTB	10	0.31223364	5.46E-20	9.97E-16	0.786	0.626
SDC4	10	0.30899801	1.62E-20	2.96E-16	0.557	0.352
STK17A	10	0.29667041	1.16E-13	2.11E-09	0.523	0.35
CBX6	10	0.29390746	1.12E-34	2.05E-30	0.534	0.254
GRB10	10	0.29143681	2.53E-21	4.63E-17	0.28	0.116
CSGALNACT2	10	0.2890696	1.18E-12	2.15E-08	0.386	0.243
LRRC75A	10	0.28342999	1.65E-45	3.00E-41	0.34	0.104
SNHG12	10	0.27147373	1.90E-14	3.47E-10	0.566	0.396
NRP2	10	0.2644277	5.31E-10	9.69E-06	0.446	0.306
CD55	10	0.25288987	6.07E-09	0.000110693	0.703	0.579
LSM12	10	0.25210943	1.35E-18	2.46E-14	0.486	0.294
GIGYF1	10	0.25110194	2.38E-54	4.34E-50	0.357	0.1
PER2	10	0.25082422	5.73E-28	1.05E-23	0.303	0.117
PPFIBP1	10	0.25015251	4.97E-09	9.07E-05	0.563	0.453
**gene**	**cluster**	**avg_logFC**	**p_val**	**p_val_adj**	**pct.1**	**pct.2**
DDIT3	11	1.01636039	1.98829E-77	3.62803E-73	0.855	0.495
HSPA5	11	0.91244838	3.91406E-64	7.14199E-60	0.808	0.462
NUPR1	11	0.81865542	2.28731E-78	4.17365E-74	0.991	0.966
DNAJB9	11	0.8130223	1.52999E-36	2.79177E-32	0.805	0.614
SLC3A2	11	0.77190581	2.99164E-52	5.45884E-48	0.752	0.402
TCEA1	11	0.7216598	3.13005E-71	5.7114E-67	0.934	0.748
GDF15	11	0.69976465	3.9355E-116	7.1811E-112	0.421	0.073
SARS	11	0.67811653	5.64109E-70	1.02933E-65	0.855	0.561
PPP1R15A	11	0.67615385	1.81982E-36	3.32062E-32	0.635	0.338
MYLK	11	0.65840034	1.64116E-35	2.99462E-31	0.528	0.271

HERPUD1	11	0.64516059	4.15499E-33	7.58161E-29	0.739	0.485
XBP1	11	0.64374725	2.49601E-41	4.55448E-37	0.862	0.624
TRIB3	11	0.63383925	5.9572E-233	1.087E-228	0.371	0.028
FTL	11	0.62672792	3.97119E-43	7.24622E-39	1	1
RPS27L	11	0.56241273	1.08334E-48	1.97677E-44	0.991	0.953
MAP1LC3B	11	0.55658305	6.4877E-30	1.18381E-25	0.761	0.509
VIMP	11	0.55368449	2.41424E-33	4.40526E-29	0.868	0.694
SQSTM1	11	0.5386931	5.80024E-39	1.05837E-34	0.981	0.895
ARF4	11	0.52124572	2.62515E-16	4.79012E-12	0.84	0.726
HSPA9	11	0.51855897	1.06446E-60	1.94231E-56	0.594	0.241
RHEB	11	0.49004017	3.79267E-42	6.92049E-38	0.962	0.788
MANF	11	0.48268286	2.67609E-14	4.88306E-10	0.506	0.332
FTH1	11	0.47586166	2.24136E-28	4.08982E-24	1	1
PRDX1	11	0.46621681	5.48781E-36	1.00136E-31	0.95	0.828
CDKN1A	11	0.46567728	3.03516E-25	5.53826E-21	0.409	0.198
ISG15	11	0.46422136	5.60459E-07	0.010226689	0.186	0.102
SEC61B	11	0.45941699	5.27048E-36	9.61704E-32	0.928	0.814
MGST1	11	0.45858142	8.92906E-13	1.62929E-08	0.443	0.29
CYCS	11	0.45133861	1.45645E-22	2.65759E-18	0.701	0.49
BEX2	11	0.44812387	1.01105E-26	1.84486E-22	0.55	0.312
RNF187	11	0.4459392	4.52439E-35	8.25565E-31	0.764	0.507
CEBPB	11	0.4403046	9.16598E-29	1.67252E-24	0.805	0.58
ATP6V0B	11	0.42384025	1.31753E-21	2.4041E-17	0.733	0.533
CDK2AP2	11	0.42349519	1.49185E-18	2.72218E-14	0.494	0.286
UBC	11	0.4201087	2.46031E-21	4.48933E-17	0.981	0.933
PFDN2	11	0.41278706	1.00624E-22	1.83608E-18	0.77	0.57
CEBPG	11	0.4092593	1.2882E-53	2.35059E-49	0.528	0.196
GHITM	11	0.40762429	7.3981E-31	1.34993E-26	0.84	0.609
XPOT	11	0.39899758	2.62247E-57	4.78523E-53	0.557	0.206
NR1D1	11	0.39193081	1.28389E-08	0.000234272	0.469	0.321
SDF2L1	11	0.39003641	5.43535E-31	9.91789E-27	0.525	0.255
ODC1	11	0.38502917	1.3568E-29	2.47575E-25	0.594	0.333
HSP90AA1	11	0.38295605	4.99687E-15	9.11779E-11	0.868	0.745
SELK	11	0.38289867	2.50654E-12	4.57368E-08	0.874	0.814
ORM1	11	0.38172633	6.6673E-09	0.000121658	0.352	0.226
RNH1	11	0.36651659	5.30569E-27	9.6813E-23	0.752	0.535
SDCBP	11	0.36385489	5.26885E-07	0.009614064	0.619	0.541
SPCS2	11	0.36372765	1.21968E-21	2.22555E-17	0.909	0.793
MTHFD2	11	0.36360921	2.39885E-18	4.37718E-14	0.736	0.558

GARS	11	0.36238992	2.31777E-41	4.22923E-37	0.553	0.246
SEC11C	11	0.36067115	5.75304E-28	1.04976E-23	0.525	0.272
TIMM17A	11	0.36033497	7.9034E-18	1.44213E-13	0.459	0.26
TMED9	11	0.35968215	2.4458E-21	4.46284E-17	0.824	0.62
POLR2K	11	0.35675361	1.63234E-12	2.97853E-08	0.714	0.567
TXN	11	0.35294826	3.23084E-11	5.89532E-07	0.906	0.846
SERP1	11	0.35126925	9.39125E-12	1.71362E-07	0.881	0.831
ADRM1	11	0.34969405	2.04979E-31	3.74024E-27	0.591	0.319
IFRD1	11	0.34612734	4.66795E-17	8.51761E-13	0.682	0.505
HSP90B1	11	0.34487448	2.20882E-18	4.03043E-14	0.89	0.783
EIF2S2	11	0.34197982	8.27855E-28	1.51059E-23	0.893	0.776
MYDGF	11	0.33796891	1.76957E-14	3.22894E-10	0.862	0.775
CCPG1	11	0.33122608	1.31537E-17	2.40015E-13	0.67	0.481
TRMT112	11	0.33074562	9.72435E-24	1.7744E-19	0.962	0.899
BAX	11	0.32666292	1.54266E-23	2.81488E-19	0.61	0.366
NUDC	11	0.32584977	1.42521E-21	2.60058E-17	0.777	0.587
WARS	11	0.32550266	2.31553E-63	4.22514E-59	0.324	0.073
HNRNPH3	11	0.32539989	2.55654E-12	4.66492E-08	0.604	0.447
ERH	11	0.32349542	5.11855E-20	9.33981E-16	0.862	0.716
ISCU	11	0.32328154	1.58423E-18	2.89074E-14	0.597	0.413
DNAJB11	11	0.32182673	4.03698E-24	7.36627E-20	0.481	0.242
PSMB3	11	0.3216184	1.12299E-21	2.04912E-17	0.579	0.361
PTP4A1	11	0.32153336	6.60041E-17	1.20438E-12	0.695	0.479
HM13	11	0.32096612	2.79148E-18	5.09361E-14	0.56	0.351
PSMA7	11	0.31545006	3.19838E-25	5.83608E-21	0.953	0.862
SNU13	11	0.31277787	1.34046E-15	2.44594E-11	0.906	0.848
SNRPG	11	0.31248116	1.87826E-11	3.42726E-07	0.833	0.727
GFPT1	11	0.31227043	1.28984E-20	2.35358E-16	0.481	0.271
H3F3B	11	0.31199046	1.40597E-13	2.56547E-09	1	0.999
TKT	11	0.30870773	6.04911E-16	1.10378E-11	0.698	0.513
RNF181	11	0.30857247	2.14944E-17	3.92208E-13	0.761	0.584
CANX	11	0.30698977	3.19576E-19	5.8313E-15	0.846	0.712
RAD23A	11	0.30629126	8.70396E-20	1.58821E-15	0.796	0.635
ARF1	11	0.3058705	9.51101E-20	1.73547E-15	0.833	0.65
ZC3H15	11	0.30476442	2.69137E-18	4.91094E-14	0.695	0.484
CYC1	11	0.30352654	5.78431E-19	1.05546E-14	0.676	0.457
COPE	11	0.30332203	1.7233E-19	3.1445E-15	0.752	0.559
PSMB1	11	0.30309391	4.22588E-18	7.71096E-14	0.84	0.692
COX7B	11	0.30280978	2.40124E-11	4.38154E-07	0.748	0.628

PSMB6	11	0.30251987	1.21492E-18	2.21686E-14	0.673	0.488
UFM1	11	0.30165533	1.0762E-14	1.96373E-10	0.723	0.56
UQCC2	11	0.30131842	2.64516E-19	4.82663E-15	0.695	0.476
TERF2IP	11	0.29838187	6.82839E-12	1.24598E-07	0.597	0.437
TALDO1	11	0.29766105	4.712E-16	8.59799E-12	0.654	0.481
CAMTA2	11	0.29759747	1.10883E-22	2.02329E-18	0.318	0.135
WBSCR22	11	0.29702957	3.99816E-23	7.29544E-19	0.572	0.334
SF3B2	11	0.29169396	6.37472E-17	1.1632E-12	0.594	0.396
EIF1B	11	0.29164777	8.33776E-17	1.52139E-12	0.821	0.667
TCEB1	11	0.29102922	1.23937E-10	2.26148E-06	0.726	0.6
EIF5	11	0.29091154	1.01925E-13	1.85983E-09	0.808	0.647
MTCH1	11	0.29048603	2.65184E-17	4.8388E-13	0.698	0.499
ANXA5	11	0.28958193	2.28361E-13	4.16691E-09	0.909	0.822
SRP14	11	0.28897534	7.78987E-21	1.42142E-16	0.994	0.983
BUD31	11	0.28811199	1.03188E-16	1.88287E-12	0.651	0.453
DDX5	11	0.28730423	5.4956E-11	1.00278E-06	0.742	0.584
TMBIM6	11	0.28531224	5.07812E-20	9.26605E-16	0.858	0.696
MTDH	11	0.28431453	4.52193E-11	8.25117E-07	0.78	0.651
ATF5	11	0.28360783	1.91156E-19	3.48802E-15	0.236	0.093
COX6A1	11	0.28152528	7.807E-16	1.42454E-11	0.89	0.836
DDX24	11	0.2803736	1.05735E-09	1.92936E-05	0.585	0.445
DNAJA1	11	0.27968341	4.83E-17	8.8133E-13	0.428	0.235
NAPA	11	0.27925379	3.3548E-21	6.12151E-17	0.465	0.251
TRAM1	11	0.2791617	4.73736E-12	8.64426E-08	0.877	0.774
PHGDH	11	0.27893726	2.63551E-44	4.80902E-40	0.387	0.124
CMSS1	11	0.27879972	8.06644E-18	1.47188E-13	0.547	0.342
EIF1	11	0.27877083	3.17155E-27	5.78713E-23	1	1
PHLDA3	11	0.27830939	3.33693E-23	6.0889E-19	0.415	0.2
MORF4L2	11	0.27793397	1.42639E-06	0.026027287	0.742	0.651
DNAJC8	11	0.27773991	9.0053E-15	1.6432E-10	0.619	0.446
SAP18	11	0.27676133	3.52808E-17	6.43768E-13	0.899	0.814
SLIRP	11	0.27464993	6.43838E-15	1.17481E-10	0.855	0.704
PSMD8	11	0.27070791	1.80657E-15	3.29645E-11	0.701	0.531
ATF4	11	0.2704856	3.41641E-20	6.23393E-16	0.956	0.895
MKNK2	11	0.26861941	3.38832E-41	6.18267E-37	0.377	0.125
DNAJC3	11	0.2675472	3.79888E-08	0.000693182	0.513	0.374
FKBP2	11	0.26700036	7.79183E-11	1.42177E-06	0.752	0.664
DNAJB6	11	0.26463118	4.75551E-11	8.67739E-07	0.594	0.455
POMP	11	0.26331062	8.41211E-11	1.53496E-06	0.912	0.845

PDIA3	11	0.26305986	9.59548E-15	1.75089E-10	0.736	0.579
PTGES3	11	0.2628195	5.13917E-18	9.37745E-14	0.969	0.927
SAR1B	11	0.25937563	7.75882E-14	1.41575E-09	0.525	0.346
PSMD11	11	0.25909883	3.28668E-19	5.99721E-15	0.513	0.295
SSR3	11	0.25733173	7.20109E-16	1.31398E-11	0.95	0.877
PSMC1	11	0.25706328	2.76385E-16	5.0432E-12	0.547	0.352
TGIF1	11	0.25659859	3.52476E-11	6.43163E-07	0.572	0.424
AKR1C2	11	0.2550844	6.85694E-07	0.012511851	0.264	0.165
ATP6V1E1	11	0.25507052	8.10666E-20	1.47922E-15	0.531	0.306
SF3B5	11	0.25319813	2.94569E-11	5.375E-07	0.84	0.729
MDM2	11	0.25089938	4.01497E-16	7.32612E-12	0.292	0.137
**gene**	**cluster**	**avg_logFC**	**p_val**	**p_val_adj**	**pct.1**	**pct.2**
MMP3	12	1.5295724	7.27E-115	1.33E-110	0.983	0.742
COMP	12	1.14845643	2.95E-51	5.37E-47	0.917	0.806
SPP1	12	1.02146992	6.67E-15	1.22E-10	0.62	0.417
CHI3L2	12	0.99530207	2.91E-38	5.32E-34	0.809	0.581
SERPINA1	12	0.87320209	5.15E-67	9.39E-63	0.997	0.925
LUM	12	0.83699934	1.45E-79	2.65E-75	1	0.999
PRG4	12	0.7557971	2.07E-14	3.78E-10	0.482	0.313
TIMP1	12	0.74793424	5.74E-57	1.05E-52	1	0.997
LCN2	12	0.74678932	5.48E-30	9.99E-26	0.69	0.435
CLU	12	0.69091147	2.99E-58	5.46E-54	1	0.995
SLC3A2	12	0.6234273	1.21E-45	2.20E-41	0.719	0.403
ORM1	12	0.62121745	2.05E-10	3.73E-06	0.366	0.225
PTGS2	12	0.59540223	7.62E-26	1.39E-21	0.587	0.331
ADM	12	0.54391788	1.13E-14	2.06E-10	0.789	0.663
DCN	12	0.5349675	9.81E-53	1.79E-48	1	0.998
DDIT4L	12	0.51901737	2.92E-25	5.34E-21	0.802	0.624
MFGE8	12	0.47673454	4.79E-35	8.75E-31	0.954	0.822
BHLHE40	12	0.47541002	1.94E-19	3.55E-15	0.637	0.465
PGK1	12	0.46172278	9.96E-32	1.82E-27	0.977	0.909
SLC2A1	12	0.44991103	3.09E-25	5.63E-21	0.535	0.298
ERO1A	12	0.44724299	3.24E-13	5.91E-09	0.65	0.534
CD55	12	0.44686755	9.28E-29	1.69E-24	0.815	0.577
PRELP	12	0.44424925	1.12E-24	2.04E-20	0.987	0.925
ENO1	12	0.42646548	2.54E-37	4.63E-33	0.997	0.983
HLA-A	12	0.4185728	1.68E-20	3.06E-16	0.957	0.887
CHI3L1	12	0.41775613	5.31E-18	9.69E-14	0.983	0.919
SERPING1	12	0.41404591	2.33E-25	4.25E-21	0.743	0.53

ORM2	12	0.41091964	3.03E-14	5.53E-10	0.231	0.1
LAPTM4A	12	0.4105258	1.47E-37	2.68E-33	0.99	0.947
HLA-C	12	0.39622595	2.32E-28	4.24E-24	0.96	0.871
PTP4A1	12	0.38975148	4.00E-14	7.31E-10	0.63	0.481
TIMP3	12	0.38057005	1.48E-07	0.002702892	0.614	0.513
SOD2	12	0.37347122	8.88E-19	1.62E-14	0.974	0.894
HLA-B	12	0.36330775	3.43E-20	6.26E-16	0.881	0.776
TMEM45A	12	0.36188928	6.76E-12	1.23E-07	0.861	0.813
AK2	12	0.35873025	7.10E-22	1.30E-17	0.809	0.656
BZW1	12	0.35200984	2.00E-12	3.65E-08	0.739	0.621
ESD	12	0.34870849	5.30E-20	9.67E-16	0.865	0.772
NPR3	12	0.34745231	3.98E-22	7.26E-18	0.277	0.108
LAMP2	12	0.34451179	7.94E-22	1.45E-17	0.66	0.457
GBE1	12	0.33999426	1.44E-15	2.62E-11	0.726	0.577
ABI3BP	12	0.33857212	1.18E-09	2.15E-05	0.578	0.467
TMEM59	12	0.33399403	1.07E-31	1.94E-27	0.997	0.971
CCT8	12	0.33021005	2.50E-17	4.55E-13	0.743	0.606
VAPA	12	0.3252865	1.79E-21	3.27E-17	0.95	0.894
ATP6V1G1	12	0.3247944	1.15E-19	2.10E-15	0.983	0.96
SKP1	12	0.32368451	8.76E-36	1.60E-31	0.997	0.979
EMC2	12	0.32306611	1.97E-16	3.60E-12	0.604	0.441
SQSTM1	12	0.32120359	7.61E-23	1.39E-18	0.983	0.895
IFRD1	12	0.3204773	1.33E-13	2.42E-09	0.653	0.505
UBC	12	0.31885824	7.50E-17	1.37E-12	0.96	0.934
C1S	12	0.31798296	1.35E-12	2.47E-08	0.713	0.618
ENO2	12	0.31414191	3.57E-15	6.51E-11	0.627	0.445
PLCG2	12	0.31242539	1.05E-23	1.91E-19	0.297	0.115
ITM2B	12	0.31208149	7.38E-23	1.35E-18	0.993	0.972
ANXA1	12	0.3085938	7.30E-11	1.33E-06	0.818	0.737
HNRNPK	12	0.304986	7.61E-19	1.39E-14	0.832	0.725
MED21	12	0.30379951	1.08E-14	1.97E-10	0.597	0.429
PTGES3	12	0.30114309	1.09E-23	1.98E-19	0.98	0.927
LGALS3	12	0.30076629	6.75E-19	1.23E-14	0.977	0.923
UPP1	12	0.29660764	3.43E-09	6.27E-05	0.515	0.379
CRTAC1	12	0.29576862	1.83E-12	3.34E-08	0.386	0.234
DNER	12	0.28874571	2.26E-10	4.12E-06	0.587	0.448
VEGFA	12	0.28735735	1.85E-10	3.38E-06	0.716	0.583
CPE	12	0.27433677	5.59E-15	1.02E-10	0.776	0.658
RAB7A	12	0.27205722	2.57E-13	4.69E-09	0.719	0.631

TMEM38B	12	0.27200764	1.09E-14	1.98E-10	0.62	0.448
HIF1A	12	0.26919931	3.65E-09	6.65E-05	0.891	0.808
FN1	12	0.26828732	2.36E-11	4.30E-07	1	0.997
SDCBP	12	0.26662303	1.41E-12	2.57E-08	0.67	0.54
WDR33	12	0.26577445	1.20E-14	2.20E-10	0.591	0.414
PLA2G4A	12	0.2656	3.87E-09	7.07E-05	0.366	0.244
XBP1	12	0.26467845	9.49E-11	1.73E-06	0.733	0.628
SYF2	12	0.26280429	9.23E-16	1.68E-11	0.871	0.774
METAP2	12	0.26181412	9.20E-13	1.68E-08	0.805	0.719
CA9	12	0.25960976	1.00E-08	0.000183131	0.488	0.358
RAB1A	12	0.25857483	3.41E-11	6.23E-07	0.795	0.717
AIMP1	12	0.25654945	3.77E-12	6.87E-08	0.65	0.527
PCOLCE2	12	0.25549764	5.28E-17	9.64E-13	0.96	0.904
ARPP19	12	0.25229449	4.65E-12	8.48E-08	0.68	0.549
PID1	12	0.25166281	2.70E-09	4.93E-05	0.597	0.485
SOD3	12	0.25025768	4.50E-13	8.21E-09	0.974	0.919
**gene**	**cluster**	**avg_logFC**	**p_val**	**p_val_adj**	**pct.1**	**pct.2**
HIST1H4C	13	2.38875711	1.18755E-54	2.16692E-50	0.849	0.503
TUBA1B	13	2.27228344	2.3194E-167	4.2323E-163	1	0.343
TMSB4X	13	2.1540031	1.3078E-102	2.38626E-98	1	0.768
STMN1	13	2.13243582	0	0	0.98	0.064
LGALS1	13	1.88400448	1.4346E-115	2.6176E-111	1	0.973
H2AFZ	13	1.88296632	6.7158E-106	1.2254E-101	0.99	0.847
KIAA0101	13	1.88110534	0	0	0.899	0.048
HMGB2	13	1.80844934	6.7809E-119	1.2373E-114	0.91	0.372
UBE2S	13	1.63696645	0	0	0.864	0.09
PTTG1	13	1.54511046	0	0	0.884	0.06
CKS2	13	1.53771142	4.7499E-277	8.6671E-273	0.849	0.103
TOP2A	13	1.52132354	0	0	0.849	0.012
C12orf75	13	1.52124796	4.6584E-200	8.5001E-196	0.889	0.163
TMSB10	13	1.4844476	6.3058E-101	1.15062E-96	1	0.988
BIRC5	13	1.43149535	0	0	0.91	0.018
TUBB	13	1.41381418	3.01233E-96	5.4966E-92	0.985	0.798
ARL6IP1	13	1.40747321	1.16779E-69	2.13087E-65	0.864	0.48
UBE2C	13	1.39878865	0	0	0.839	0.015
CRIP1	13	1.37611961	3.80233E-98	6.93811E-94	0.874	0.331
HMGB1	13	1.36870713	4.1982E-100	7.6604E-96	0.99	0.763
HMGN2	13	1.35910155	3.15416E-68	5.7554E-64	0.965	0.896
PFN1	13	1.33143977	9.77467E-87	1.78358E-82	0.995	0.877

H2AFV	13	1.30069343	1.1292E-116	2.0604E-112	0.975	0.505
CFL1	13	1.29521015	3.76433E-91	6.86878E-87	0.99	0.77
CDKN3	13	1.28474054	0	0	0.834	0.045
CENPF	13	1.22968342	0	0	0.784	0.009
PTMS	13	1.21719608	5.77855E-63	1.05441E-58	0.92	0.666
RRM2	13	1.17090262	0	0	0.673	0.011
HN1	13	1.17017563	1.5604E-161	2.8472E-157	0.779	0.15
TYMS	13	1.16694607	0	0	0.905	0.063
AXL	13	1.1629493	2.14024E-68	3.90529E-64	0.799	0.337
RHEB	13	1.16011208	5.5255E-103	1.00823E-98	1	0.789
PPP1R14B	13	1.13879229	1.0979E-104	2.0033E-100	0.759	0.201
ACTB	13	1.13848137	1.16484E-78	2.12549E-74	0.995	0.944
CALM2	13	1.13131473	6.92587E-85	1.26376E-80	1	0.968
CDK1	13	1.12115387	0	0	0.688	0.012
TPM4	13	1.08869853	6.7385E-128	1.2296E-123	0.915	0.281
COL1A1	13	1.07331669	1.2339E-103	2.2515E-99	0.412	0.054
DYNLL1	13	1.06825523	3.14599E-61	5.7405E-57	0.985	0.846
NUCKS1	13	1.0615244	2.65231E-94	4.83968E-90	0.99	0.632
CENPW	13	1.06005573	0	0	0.814	0.029
MZT1	13	1.05703434	3.2919E-109	6.0067E-105	0.95	0.392
PRSS23	13	1.05531257	6.89853E-48	1.25878E-43	0.749	0.342
PBK	13	1.04416522	0	0	0.774	0.007
TM4SF1	13	1.04378116	7.75673E-43	1.41537E-38	0.879	0.51
COL6A3	13	1.03423615	2.31552E-87	4.22512E-83	0.864	0.343
COL1A2	13	1.03079836	3.74596E-57	6.83525E-53	0.704	0.275
CKS1B	13	1.02419935	1.1326E-102	2.06672E-98	0.94	0.451
NUSAP1	13	1.00770248	0	0	0.754	0.009
THY1	13	1.00350444	3.39701E-47	6.19852E-43	0.794	0.424
EIF5A	13	0.99185165	1.23215E-50	2.24831E-46	0.879	0.561
LMNA	13	0.98936078	1.11328E-85	2.0314E-81	0.854	0.316
TK1	13	0.97622001	0	0	0.688	0.03
VIM	13	0.97371625	4.40086E-42	8.03026E-38	0.995	0.999
CDKN2C	13	0.97278815	1.31447E-98	2.39851E-94	0.734	0.202
DRAP1	13	0.96753819	1.08205E-73	1.97441E-69	0.94	0.586
SMC4	13	0.96059679	0	0	0.794	0.056
SKA2	13	0.9513697	2.7408E-256	5.0011E-252	0.839	0.105
TUBA1C	13	0.95069401	1.01803E-82	1.8576E-78	0.849	0.347
CCNB1	13	0.94899451	4.1392E-224	7.5528E-220	0.523	0.042
DUT	13	0.94705487	1.98513E-62	3.62226E-58	0.95	0.627

PRC1	13	0.94342337	0	0	0.749	0.014
TMEM106C	13	0.94171101	1.07512E-81	1.96176E-77	0.849	0.35
ARPC1B	13	0.93555212	2.89894E-55	5.2897E-51	0.844	0.473
YWHAH	13	0.9323229	3.29249E-86	6.00781E-82	0.96	0.491
TUBB4B	13	0.93230014	1.57414E-58	2.87234E-54	0.849	0.438
DEK	13	0.92272074	2.97838E-77	5.43464E-73	0.96	0.634
POLR2L	13	0.9158435	4.92482E-52	8.98631E-48	0.98	0.856
ITGB1	13	0.91316253	1.83712E-75	3.35218E-71	0.94	0.517
PRKCDBP	13	0.90962425	5.29016E-50	9.65295E-46	0.839	0.482
MAD2L1	13	0.90951697	0	0	0.744	0.046
CKAP2	13	0.90901555	0	0	0.698	0.019
HMGN3	13	0.90793886	2.0432E-63	3.72823E-59	0.799	0.411
CDKN2D	13	0.90684254	6.3675E-145	1.1619E-140	0.673	0.112
CYTL1	13	0.89811113	3.78319E-39	6.90318E-35	0.543	0.196
RANBP1	13	0.88779011	9.47793E-54	1.72944E-49	0.864	0.543
DTYMK	13	0.88644851	8.5804E-169	1.5657E-164	0.759	0.132
MYL12A	13	0.87653913	2.89576E-63	5.2839E-59	0.935	0.554
SH3BGRL3	13	0.87312814	9.41451E-50	1.71787E-45	0.99	0.978
HMGN1	13	0.86830921	7.7954E-57	1.42243E-52	0.975	0.833
TNFRSF12A	13	0.84709864	1.39754E-45	2.55009E-41	0.809	0.41
CALU	13	0.84471423	2.09001E-70	3.81365E-66	0.95	0.58
RAN	13	0.83743966	1.38953E-72	2.53547E-68	0.98	0.742
DBI	13	0.83547374	2.68877E-62	4.90619E-58	0.935	0.736
MYL6	13	0.83132947	8.58849E-64	1.56714E-59	1	0.93
KPNA2	13	0.83063561	6.938E-233	1.266E-228	0.492	0.035
TPX2	13	0.82475212	0	0	0.688	0.006
MKI67	13	0.81536666	0	0	0.638	0.005
PHLDA2	13	0.81429125	3.21072E-67	5.8586E-63	0.593	0.163
LSM5	13	0.81032717	1.72155E-77	3.14131E-73	0.96	0.591
LSM4	13	0.80883996	3.91962E-89	7.15214E-85	0.839	0.313
CBX5	13	0.80841515	5.33746E-96	9.73927E-92	0.93	0.359
RPL39L	13	0.80321702	6.1913E-103	1.12973E-98	0.799	0.235
S100A10	13	0.80219905	8.0201E-47	1.46343E-42	1	0.98
CYCS	13	0.78848039	2.97059E-59	5.42043E-55	0.884	0.489
HSPA8	13	0.78621915	2.64371E-42	4.82397E-38	0.809	0.455
HNRNPA2B1	13	0.78161993	8.76758E-55	1.59982E-50	0.905	0.57
S100A11	13	0.77865885	4.12046E-50	7.51861E-46	0.99	0.918
CCDC34	13	0.77736287	1.2892E-139	2.3524E-135	0.724	0.137
UBE2T	13	0.7760133	0	0	0.653	0.02

S100A4	13	0.77190733	2.51894E-25	4.59632E-21	0.91	0.773
SRSF2	13	0.77161008	5.41841E-60	9.88697E-56	0.839	0.415
CALM1	13	0.76929113	3.92271E-61	7.15777E-57	0.94	0.543
HSP90AA1	13	0.76555178	5.31471E-37	9.69776E-33	0.955	0.744
ARPC5	13	0.76510667	2.26974E-58	4.14159E-54	0.91	0.55
COX8A	13	0.76328042	1.76899E-71	3.22788E-67	0.995	0.875
RTN4	13	0.76034241	1.12804E-57	2.05833E-53	0.95	0.636
MORF4L2	13	0.75551011	1.99789E-53	3.64555E-49	0.935	0.649
CCNA2	13	0.75406805	0	0	0.598	0.009
YWHAZ	13	0.7464054	2.10973E-67	3.84963E-63	0.955	0.57
CALM3	13	0.74427912	3.21117E-54	5.85942E-50	0.92	0.643
SNRPG	13	0.74317717	1.5595E-42	2.84561E-38	0.915	0.727
CLIC1	13	0.74148415	4.25305E-55	7.76053E-51	0.975	0.835
CDC20	13	0.73727942	0	0	0.503	0.006
CYR61	13	0.73552408	7.95037E-19	1.4507E-14	0.683	0.417
CDCA3	13	0.73015074	0	0	0.613	0.009
KLF6	13	0.73010214	2.70273E-48	4.93168E-44	0.648	0.245
SMS	13	0.72957691	2.18229E-48	3.98203E-44	0.869	0.528
CRNDE	13	0.72683218	8.65048E-82	1.57845E-77	0.719	0.213
CCNB2	13	0.72562485	0	0	0.578	0.012
BCL7C	13	0.72255821	3.75705E-73	6.85549E-69	0.764	0.282
AP2B1	13	0.71891836	1.82468E-71	3.32949E-67	0.779	0.284
CLEC11A	13	0.71837043	3.55568E-43	6.48804E-39	0.814	0.448
PLP2	13	0.71248215	2.36144E-52	4.30892E-48	0.975	0.774
KIF20B	13	0.71096057	0	0	0.568	0.024
ANXA2	13	0.7104787	2.09403E-36	3.82098E-32	0.98	0.853
ANP32E	13	0.70733716	1.38408E-60	2.52553E-56	0.734	0.294
COX20	13	0.70697576	3.77365E-59	6.88577E-55	0.93	0.634
CD151	13	0.70346144	6.6496E-46	1.21335E-41	0.844	0.485
CKAP4	13	0.70310666	9.68885E-62	1.76792E-57	0.774	0.31
ANLN	13	0.70133685	0	0	0.628	0.006
CENPA	13	0.69667547	0	0	0.442	0.004
TXN	13	0.68871649	7.57409E-54	1.38204E-49	0.98	0.845
H2AFX	13	0.68380517	1.8729E-126	3.4175E-122	0.583	0.094
GGH	13	0.68274565	3.0628E-101	5.5887E-97	0.618	0.125
CENPK	13	0.68177707	0	0	0.658	0.017
FSTL1	13	0.67830629	1.79757E-54	3.28003E-50	0.774	0.345
SERF2	13	0.67812228	9.28916E-54	1.69499E-49	1	1
TUBB6	13	0.67621941	1.4036E-107	2.5612E-103	0.739	0.176

NRP2	13	0.67296541	6.46964E-61	1.18051E-56	0.804	0.302
ENPP1	13	0.66799973	5.95523E-14	1.08665E-09	0.523	0.315
RACGAP1	13	0.66481771	0	0	0.618	0.023
MALL	13	0.66376132	5.36088E-40	9.782E-36	0.633	0.271
SRSF3	13	0.65782563	3.54059E-54	6.46052E-50	0.95	0.725
ARPC2	13	0.65691467	3.84885E-45	7.02299E-41	0.955	0.793
IGFBP3	13	0.65224478	1.38311E-27	2.52376E-23	0.638	0.31
HNRNPH1	13	0.65145699	1.17444E-15	2.14301E-11	0.744	0.506
FAM64A	13	0.64971947	0	0	0.588	0.005
BOLA3	13	0.64969459	3.51138E-69	6.40721E-65	0.779	0.282
RBX1	13	0.6482322	1.22245E-45	2.2306E-41	0.884	0.571
CKLF	13	0.64513201	9.98119E-44	1.82127E-39	0.794	0.413
S100A16	13	0.64459913	1.74748E-25	3.18863E-21	0.638	0.354
ATP5J2	13	0.64345953	2.27961E-41	4.1596E-37	0.864	0.537
MAP1B	13	0.64302698	8.54949E-63	1.56002E-58	0.492	0.116
COL6A1	13	0.64193646	7.36056E-42	1.34308E-37	0.844	0.472
BANF1	13	0.64128173	7.6378E-46	1.39367E-41	0.849	0.518
PPIA	13	0.64105663	6.84652E-56	1.24928E-51	1	0.96
ANAPC11	13	0.64090756	1.6666E-49	3.04105E-45	0.95	0.761
PTP4A2	13	0.63746143	6.67211E-53	1.21746E-48	0.874	0.475
GTSE1	13	0.63555442	0	0	0.558	0.011
APOLD1	13	0.63516341	3.7299E-221	6.8059E-217	0.558	0.048
ACTG1	13	0.63225568	6.12687E-29	1.11797E-24	0.995	0.956
ACTN4	13	0.63208493	3.60358E-87	6.57545E-83	0.769	0.224
HMOX1	13	0.63188963	6.76295E-26	1.23404E-21	0.467	0.189
AURKB	13	0.63090618	0	0	0.553	0.005
SAC3D1	13	0.6304874	1.0136E-185	1.8496E-181	0.603	0.068
CTNNAL1	13	0.62972833	9.50521E-85	1.73442E-80	0.673	0.178
CAPG	13	0.62874716	4.90239E-39	8.9454E-35	0.734	0.379
INSIG1	13	0.62708963	3.15905E-33	5.76432E-29	0.759	0.417
UQCC2	13	0.62681099	4.68465E-45	8.54808E-41	0.829	0.476
OAZ1	13	0.62620933	1.3497E-52	2.4628E-48	1	0.997
ERH	13	0.62603474	8.84909E-45	1.61469E-40	0.93	0.716
TPM3	13	0.62500945	1.55757E-57	2.8421E-53	0.779	0.333
AP2S1	13	0.62175257	9.8452E-45	1.79645E-40	0.864	0.534
ZWINT	13	0.6177711	0	0	0.598	0.033
YBX1	13	0.61732989	3.34231E-46	6.09871E-42	1	0.982
HMGB3	13	0.61545252	0	0	0.563	0.028
SPATS2L	13	0.61338518	2.02184E-44	3.68926E-40	0.799	0.439

PDGFRA	13	0.61219936	3.63562E-51	6.63392E-47	0.693	0.265
PGF	13	0.61204896	9.64511E-72	1.75994E-67	0.337	0.05
ASPM	13	0.61204859	0	0	0.487	0.003
RRBP1	13	0.61145917	1.89411E-48	3.45619E-44	0.819	0.413
SRM	13	0.60969367	9.08848E-31	1.65837E-26	0.683	0.369
TAGLN2	13	0.60822297	7.48801E-37	1.36634E-32	0.925	0.63
DEPDC1	13	0.60792875	0	0	0.503	0.004
PGP	13	0.60299396	3.5876E-160	6.5462E-156	0.573	0.071
RND3	13	0.6007073	5.15172E-16	9.40035E-12	0.392	0.178
KIF5B	13	0.6003433	1.33872E-59	2.44276E-55	0.714	0.262
PTTG1IP	13	0.59756591	1.95224E-42	3.56226E-38	0.844	0.469
FKBP1A	13	0.59618092	1.28186E-29	2.339E-25	0.759	0.491
VCAN	13	0.59517296	2.25258E-30	4.11029E-26	0.809	0.432
DIXDC1	13	0.59464346	1.62742E-90	2.96955E-86	0.623	0.142
SNRPB	13	0.59338467	8.23221E-43	1.50213E-38	0.709	0.337
EMP3	13	0.59247096	2.138E-33	3.90122E-29	0.99	0.874
RAD21	13	0.59020049	1.60119E-72	2.9217E-68	0.789	0.278
MGLL	13	0.58674693	4.93516E-67	9.00518E-63	0.588	0.155
HMMR	13	0.58603643	0	0	0.553	0.003
EIF4G2	13	0.58540455	1.42218E-36	2.59505E-32	0.864	0.575
C9orf3	13	0.58304313	5.23507E-34	9.55243E-30	0.734	0.4
LOXL1	13	0.58035098	1.66981E-77	3.0469E-73	0.407	0.068
PTMA	13	0.58031741	1.82207E-45	3.32473E-41	1	1
COL5A2	13	0.58007599	2.18091E-41	3.9795E-37	0.819	0.405
FHL2	13	0.57981251	7.38449E-55	1.34745E-50	0.608	0.191
CENPM	13	0.57930666	0	0	0.528	0.014
TNC	13	0.57922231	1.0045E-117	1.8328E-113	0.563	0.086
COL5A1	13	0.57403943	1.1222E-68	2.04768E-64	0.638	0.177
FOSL1	13	0.57399639	3.19118E-42	5.82294E-38	0.568	0.186
MZT2B	13	0.57144177	7.53271E-54	1.37449E-49	0.995	0.957
SPC25	13	0.57072844	0	0	0.508	0.002
PGAM1	13	0.57066831	8.37937E-41	1.52898E-36	0.95	0.784
SMC2	13	0.56955892	1.2915E-166	2.3565E-162	0.618	0.078
CEP55	13	0.56759683	0	0	0.543	0.003
MIR4435-2HG	13	0.56698413	1.88913E-61	3.4471E-57	0.693	0.228
HNRNPAB	13	0.56689223	1.63631E-71	2.98578E-67	0.558	0.139
CENPH	13	0.56050485	1.2403E-232	2.2632E-228	0.598	0.052
TPGS2	13	0.55868941	8.41308E-61	1.53514E-56	0.729	0.27
CENPN	13	0.55452651	0	0	0.628	0.032

HP1BP3	13	0.55443402	2.63225E-46	4.80307E-42	0.784	0.391
SUMO3	13	0.55349115	1.64875E-43	3.00848E-39	0.894	0.571
C7orf73	13	0.553452	7.02101E-27	1.28112E-22	0.889	0.745
CBX1	13	0.55222767	2.94263E-54	5.36943E-50	0.759	0.305
UQCRQ	13	0.55138375	7.74917E-36	1.41399E-31	0.975	0.92
SGOL2	13	0.55105668	0	0	0.513	0.027
NUDT1	13	0.55088933	7.57578E-94	1.38235E-89	0.698	0.168
EMC9	13	0.55051872	5.23176E-93	9.5464E-89	0.729	0.182
RCAN1	13	0.54912583	1.85249E-47	3.38024E-43	0.402	0.095
NT5DC2	13	0.54902537	1.27554E-75	2.32747E-71	0.543	0.125
HINT1	13	0.54868868	2.34335E-51	4.2759E-47	1	0.983
MYDGF	13	0.54861957	1.33109E-24	2.42883E-20	0.925	0.775
MYL12B	13	0.54786137	1.826E-31	3.3319E-27	0.874	0.607
POSTN	13	0.54785012	2.71629E-25	4.95642E-21	0.161	0.031
ATP5G3	13	0.54644083	1.53305E-35	2.79735E-31	0.92	0.687
COL3A1	13	0.54229822	8.62932E-37	1.57459E-32	0.593	0.228
CKB	13	0.54209393	8.7051E-35	1.58842E-30	0.407	0.126
MLF2	13	0.54060508	1.36914E-35	2.49827E-31	0.764	0.45
CTHRC1	13	0.53970212	1.42456E-22	2.59939E-18	0.573	0.276
MT-CYB	13	0.53892676	3.11641E-13	5.68651E-09	0.829	0.721
ITGB1BP1	13	0.53875739	7.16175E-37	1.3068E-32	0.804	0.448
SNRPD1	13	0.53520386	3.49956E-37	6.38564E-33	0.869	0.572
ATP1B1	13	0.53347061	4.97635E-42	9.08034E-38	0.503	0.161
SUMO2	13	0.53313846	4.55112E-49	8.30443E-45	1	0.982
TMPO	13	0.53264739	6.3285E-137	1.1548E-132	0.528	0.069
MYEOV2	13	0.53258264	3.8683E-27	7.05849E-23	0.894	0.716
SLC9A3R2	13	0.53151434	7.09137E-19	1.29396E-14	0.548	0.301
TFDP1	13	0.52912604	3.69799E-86	6.74773E-82	0.583	0.128
PTN	13	0.52905512	2.0703E-147	3.7778E-143	0.452	0.046
RPA3	13	0.52843383	1.45574E-31	2.65628E-27	0.824	0.528
MT-CO3	13	0.52733433	4.27974E-16	7.80924E-12	0.925	0.887
DNMT1	13	0.52716425	3.0775E-172	5.6156E-168	0.538	0.057
GNAI2	13	0.52567001	2.6943E-49	4.91629E-45	0.729	0.298
CCND1	13	0.52494666	6.22223E-20	1.13537E-15	0.543	0.278
YWHAB	13	0.52044891	4.14994E-42	7.57239E-38	0.779	0.387
H3F3A	13	0.52009707	6.78109E-32	1.23734E-27	0.995	0.959
MAP4K4	13	0.51930554	1.41936E-94	2.58991E-90	0.543	0.101
CDT1	13	0.51886569	5.816E-292	1.0612E-287	0.377	0.015
HCFC1R1	13	0.51874393	1.18949E-27	2.17047E-23	0.889	0.669

C11orf24	13	0.51854292	2.09895E-66	3.82996E-62	0.518	0.126
CDCA4	13	0.51827732	1.9076E-102	3.48074E-98	0.578	0.108
MSN	13	0.51569252	6.06182E-68	1.1061E-63	0.633	0.179
MT-CO2	13	0.51562291	1.60253E-16	2.92414E-12	0.92	0.93
STRA13	13	0.51496893	1.08806E-36	1.98538E-32	0.693	0.327
FUS	13	0.51411589	4.51448E-35	8.23758E-31	0.804	0.442
CARHSP1	13	0.51393725	1.30104E-32	2.374E-28	0.849	0.547
BZW1	13	0.51303287	1.85082E-22	3.37719E-18	0.879	0.619
GUK1	13	0.51291021	6.20375E-35	1.132E-30	0.975	0.885
TMED9	13	0.50946532	1.37713E-22	2.51286E-18	0.814	0.622
YWHAQ	13	0.5091055	1.13288E-28	2.06717E-24	0.915	0.701
PTRF	13	0.50850111	1.54951E-26	2.82738E-22	0.814	0.496
RHOA	13	0.50773134	1.1298E-39	2.06155E-35	0.975	0.838
IKBIP	13	0.50636037	6.98349E-58	1.27428E-53	0.734	0.267
C4orf48	13	0.50634854	4.57949E-32	8.35619E-28	0.719	0.363
CIB1	13	0.50494888	7.78318E-28	1.4202E-23	0.844	0.563
PART1	13	0.50385647	3.34393E-55	6.10167E-51	0.482	0.123
DYNLT1	13	0.50319452	2.94243E-27	5.36906E-23	0.749	0.487
DNAJC9	13	0.50306271	4.31992E-96	7.88256E-92	0.568	0.109
MAZ	13	0.50255044	1.14796E-68	2.09468E-64	0.658	0.193
AAMDC	13	0.50145014	6.26004E-55	1.14227E-50	0.704	0.257
BUB3	13	0.49640623	2.07682E-35	3.78958E-31	0.704	0.358
HJURP	13	0.49631645	0	0	0.452	0.003
ITGAE	13	0.49535034	2.30914E-60	4.21349E-56	0.643	0.2
PHPT1	13	0.49525788	3.06965E-32	5.60119E-28	0.945	0.757
LMO4	13	0.49510306	2.63302E-31	4.80446E-27	0.638	0.29
MINOS1	13	0.49338308	1.3034E-28	2.37831E-24	0.894	0.637
MT-CO1	13	0.49163129	7.75824E-16	1.41565E-11	0.945	0.944
MT-ATP6	13	0.49122576	4.79115E-15	8.74242E-11	0.905	0.815
NAA38	13	0.48979631	1.01709E-24	1.85589E-20	0.834	0.649
LSP1	13	0.4893634	4.21267E-22	7.68687E-18	0.633	0.338
DDX39A	13	0.48929198	2.18106E-70	3.97978E-66	0.538	0.125
ZNF367	13	0.48912745	0	0	0.387	0.009
GNG11	13	0.48873672	1.74803E-34	3.18962E-30	0.613	0.271
SPARC	13	0.48828728	1.57201E-21	2.86845E-17	0.829	0.591
MTDH	13	0.48780544	1.97549E-26	3.60468E-22	0.879	0.65
CAPNS1	13	0.48652521	6.30362E-48	1.15022E-43	0.648	0.238
SRP9	13	0.48494576	3.05645E-32	5.57711E-28	0.95	0.747
PCNA	13	0.48383963	2.17148E-44	3.96229E-40	0.432	0.117

LOX	13	0.48370883	4.35964E-39	7.95504E-35	0.648	0.25
SFPQ	13	0.48204592	5.22581E-40	9.53553E-36	0.663	0.285
DNAJB1	13	0.48094129	9.98828E-17	1.82256E-12	0.482	0.238
AURKAIP1	13	0.48072698	9.90838E-29	1.80798E-24	0.859	0.633
SLC16A3	13	0.47761623	2.98096E-30	5.43935E-26	0.809	0.47
HSPB1	13	0.4773925	1.01802E-21	1.85757E-17	0.925	0.705
NME1	13	0.47666196	6.52393E-34	1.19042E-29	0.618	0.272
NQO1	13	0.47382392	9.14993E-81	1.66959E-76	0.503	0.096
CMC2	13	0.4735506	8.3811E-101	1.52931E-96	0.573	0.106
NREP	13	0.47314814	1.48339E-29	2.70674E-25	0.573	0.253
ARL4A	13	0.47289478	1.6258E-109	2.9666E-105	0.447	0.06
DHFR	13	0.47274252	2.63E-109	4.7989E-105	0.477	0.069
RNASEH2A	13	0.47146171	2.0369E-103	3.7168E-99	0.503	0.079
ATP5G1	13	0.46956819	1.65601E-28	3.02172E-24	0.739	0.427
ARPC1A	13	0.46863749	1.20142E-24	2.19223E-20	0.789	0.522
CBX3	13	0.46847222	1.79107E-37	3.26817E-33	0.955	0.68
TGFBI	13	0.46812667	7.497E-18	1.36798E-13	0.673	0.412
ZDHHC12	13	0.46802288	2.10155E-44	3.83469E-40	0.573	0.2
SIVA1	13	0.46606336	5.90118E-26	1.07679E-21	0.915	0.81
EI24	13	0.46516056	8.92307E-34	1.62819E-29	0.744	0.391
ATP1B3	13	0.46307946	2.09747E-32	3.82724E-28	0.668	0.313
PIN1	13	0.46268547	1.12549E-39	2.05368E-35	0.673	0.306
CDC42	13	0.46239465	3.41008E-18	6.22238E-14	0.844	0.654
CRIP2	13	0.46186668	8.42288E-33	1.53692E-28	0.824	0.423
RHOC	13	0.46081579	5.70743E-21	1.04143E-16	0.915	0.782
MRPL52	13	0.46058638	3.53236E-28	6.4455E-24	0.824	0.507
GNG5	13	0.46054329	4.53072E-29	8.26721E-25	0.864	0.629
NEDD8	13	0.46028316	1.66082E-27	3.0305E-23	0.91	0.718
TEX30	13	0.45992811	1.70827E-68	3.11708E-64	0.603	0.157
PDXK	13	0.4580593	4.49799E-30	8.20748E-26	0.573	0.257
NT5E	13	0.45738586	5.21454E-27	9.51497E-23	0.693	0.361
RDX	13	0.45569261	2.36604E-35	4.31732E-31	0.839	0.486
MXD3	13	0.45525647	1.6055E-304	2.9295E-300	0.407	0.017
ACTN1	13	0.45390531	1.15853E-43	2.11397E-39	0.683	0.27
PMEPA1	13	0.45357157	4.32999E-23	7.90093E-19	0.513	0.244
ISOC2	13	0.45345237	6.51051E-41	1.18797E-36	0.603	0.241
BLOC1S1	13	0.45339631	3.91116E-30	7.13669E-26	0.814	0.513
PHF19	13	0.45149287	1.9952E-158	3.6407E-154	0.487	0.05
HRAS	13	0.45144349	2.87396E-48	5.24412E-44	0.563	0.185

LSM3	13	0.45110377	8.08533E-30	1.47533E-25	0.754	0.446
LY6E	13	0.44964734	4.5326E-24	8.27063E-20	0.789	0.469
ADAM12	13	0.44781731	4.3663E-139	7.9671E-135	0.442	0.047
NFIC	13	0.44670188	6.15164E-26	1.12249E-21	0.734	0.428
ARHGAP11A	13	0.44631119	0	0	0.452	0.009
SSNA1	13	0.44432971	9.64614E-36	1.76013E-31	0.704	0.345
CTSL	13	0.44332206	2.87516E-08	0.00052463	0.754	0.572
DIAPH3	13	0.44292516	0	0	0.417	0.007
ARHGDIA	13	0.44237044	7.33156E-26	1.33779E-21	0.739	0.428
FOXM1	13	0.44231703	0	0	0.442	0.01
HIGD1A	13	0.44216562	8.43723E-35	1.53954E-30	0.683	0.333
MT-ND1	13	0.44207063	9.61044E-13	1.75362E-08	0.779	0.647
H3F3B	13	0.44131902	9.62933E-40	1.75706E-35	1	0.999
AHNAK	13	0.44087167	5.16204E-24	9.41917E-20	0.764	0.475
SLC25A5	13	0.43979948	4.43934E-19	8.10047E-15	0.744	0.52
IQGAP3	13	0.43967633	1.1333E-271	2.0679E-267	0.362	0.015
SAP30	13	0.43935462	2.14419E-37	3.91251E-33	0.648	0.275
PXMP2	13	0.43897504	1.7545E-50	3.20144E-46	0.543	0.163
LRRC59	13	0.43829284	1.81682E-27	3.31515E-23	0.593	0.291
CALR	13	0.43770525	1.42564E-18	2.60136E-14	0.839	0.669
MARCKS	13	0.43688108	3.9097E-42	7.13403E-38	0.437	0.123
NUDCD2	13	0.43607	3.28768E-37	5.99902E-33	0.653	0.29
ENO1	13	0.43442101	5.67875E-10	1.0362E-05	1	0.984
44450	13	0.43220664	1.53626E-50	2.80322E-46	0.533	0.155
GTF3A	13	0.43112447	6.3465E-23	1.15805E-18	0.678	0.399
LINC00152	13	0.43088721	3.37184E-31	6.1526E-27	0.709	0.351
HDGF	13	0.43044304	1.3073E-41	2.38543E-37	0.603	0.228
RP11-14N7.2	13	0.42983953	2.43519E-40	4.4435E-36	0.523	0.174
SEPT15	13	0.4293668	3.99918E-16	7.2973E-12	0.759	0.541
B4GALT1	13	0.42935168	9.11516E-18	1.66324E-13	0.302	0.115
MYH9	13	0.42838944	2.6374E-99	4.81239E-95	0.497	0.08
PRADC1	13	0.42696413	2.93896E-68	5.36273E-64	0.518	0.119
NTAN1	13	0.4263452	7.56037E-44	1.37954E-39	0.603	0.221
ARPC5L	13	0.42568192	4.33759E-38	7.91479E-34	0.588	0.232
KDELR2	13	0.42560021	4.38448E-21	8.00036E-17	0.925	0.858
UBB	13	0.42550584	3.77224E-18	6.8832E-14	0.95	0.857
CAPZA1	13	0.42530855	1.35376E-25	2.4702E-21	0.668	0.372
TPM2	13	0.4250511	3.14449E-18	5.73774E-14	0.809	0.569
PRG4	13	0.42497425	2.33372E-09	4.25834E-05	0.508	0.314

CRELD2	13	0.42302272	3.72691E-32	6.8005E-28	0.563	0.226
EMP2	13	0.42106266	8.7428E-31	1.5953E-26	0.794	0.434
UBALD2	13	0.42027099	2.07045E-74	3.77795E-70	0.482	0.097
GLRX5	13	0.42019493	9.2272E-45	1.68369E-40	0.528	0.172
AP3S1	13	0.41963911	5.35332E-27	9.76821E-23	0.945	0.776
CORO1C	13	0.41961132	3.93749E-53	7.18475E-49	0.523	0.146
RBM8A	13	0.41959485	1.25422E-23	2.28857E-19	0.814	0.564
UBE2N	13	0.41841683	4.18848E-31	7.64271E-27	0.673	0.321
WDR34	13	0.41763309	1.46496E-42	2.67312E-38	0.568	0.201
HSPE1	13	0.41577068	1.5424E-17	2.81442E-13	0.829	0.633
KPNB1	13	0.41536402	7.18097E-39	1.31031E-34	0.688	0.299
TPI1	13	0.41432593	1.9908E-26	3.63261E-22	1	0.989
UAP1	13	0.41395401	8.96587E-30	1.636E-25	0.719	0.366
NUF2	13	0.41387711	0	0	0.422	0.003
SET	13	0.41371867	4.26434E-11	7.78115E-07	0.829	0.748
CAV1	13	0.41366154	3.20546E-13	5.84901E-09	0.889	0.727
HNRNPA3	13	0.4104754	3.30264E-26	6.02633E-22	0.819	0.525
HNRNPR	13	0.40971201	1.93349E-27	3.52804E-23	0.618	0.306
TCEB1	13	0.40926703	2.30676E-26	4.20914E-22	0.864	0.599
FSCN1	13	0.40863174	4.05345E-43	7.39632E-39	0.503	0.158
SMTN	13	0.40822028	2.46256E-98	4.49343E-94	0.482	0.076
MAD2L2	13	0.40813849	2.76137E-35	5.03867E-31	0.623	0.264
RALBP1	13	0.40764725	1.28458E-33	2.34397E-29	0.638	0.284
NUCB2	13	0.40697046	1.22833E-21	2.24133E-17	0.734	0.449
MRPL23	13	0.40605219	2.26628E-26	4.13528E-22	0.709	0.406
PDAP1	13	0.40554163	2.2947E-17	4.18714E-13	0.704	0.459
CD59	13	0.40472637	4.20112E-20	7.66578E-16	0.985	0.94
CCDC167	13	0.404718	8.24054E-51	1.50365E-46	0.588	0.187
BAX	13	0.4040103	2.07589E-25	3.78787E-21	0.668	0.367
TMEM14A	13	0.40371543	2.85246E-26	5.20488E-22	0.698	0.384
CTNNBIP1	13	0.40365899	9.05673E-37	1.65258E-32	0.548	0.206
SEPT15	13	0.4022413	8.13006E-33	1.48349E-28	0.714	0.335
PRMT2	13	0.40128287	7.06169E-26	1.28855E-21	0.824	0.515
WHSC1	13	0.40126447	5.1192E-243	9.341E-239	0.437	0.025
TMEM54	13	0.40124684	1.75772E-56	3.20731E-52	0.543	0.147
SMOC1	13	0.40109328	6.26614E-31	1.14338E-26	0.598	0.258
UBE2E3	13	0.40097964	4.14182E-25	7.55757E-21	0.809	0.53
LSM7	13	0.40094913	7.68526E-21	1.40233E-16	0.754	0.508
ARL6IP6	13	0.40062989	9.22934E-34	1.68408E-29	0.548	0.214

NFIX	13	0.40027785	3.41358E-37	6.22877E-33	0.603	0.235
CAV2	13	0.4002545	9.59812E-21	1.75137E-16	0.688	0.414
TCF19	13	0.39959313	1.0641E-235	1.9416E-231	0.372	0.018
C19orf48	13	0.39956633	1.08088E-37	1.97228E-33	0.518	0.182
CACYBP	13	0.39935877	3.79085E-27	6.91716E-23	0.673	0.355
RUVBL2	13	0.39891981	2.62999E-48	4.79894E-44	0.558	0.174
RASSF1	13	0.39852935	4.84035E-36	8.83219E-32	0.472	0.159
ARF6	13	0.39758496	5.37579E-27	9.8092E-23	0.889	0.554
HPCAL1	13	0.39734374	1.05091E-35	1.9176E-31	0.482	0.167
NDUFS6	13	0.39714465	5.58368E-21	1.01885E-16	0.693	0.419
MTPN	13	0.39609582	2.5984E-28	4.74131E-24	0.648	0.321
TROAP	13	0.39556363	0	0	0.402	0.005
TXNDC17	13	0.39554558	1.79312E-23	3.27191E-19	0.698	0.399
LMNB2	13	0.39547962	1.9417E-135	3.5429E-131	0.407	0.041
ANKRD28	13	0.39530691	5.83558E-28	1.06482E-23	0.603	0.281
KIFC1	13	0.39518282	0	0	0.387	0.008
MT-ND4	13	0.39393721	7.92078E-17	1.4453E-12	0.945	0.945
TOMM5	13	0.39352145	1.7121E-24	3.12408E-20	0.653	0.357
AURKA	13	0.39292635	0	0	0.312	0.004
KMT5A	13	0.39271612	1.58652E-38	2.89493E-34	0.422	0.125
C20orf24	13	0.39265205	1.23267E-28	2.24926E-24	0.558	0.254
FLNA	13	0.39217591	3.42151E-47	6.24323E-43	0.508	0.149
NAV2	13	0.3915127	5.82906E-83	1.06363E-78	0.397	0.061
PRELID1	13	0.39109513	1.90985E-11	3.4849E-07	0.814	0.734
TMEM107	13	0.39005174	1.06269E-27	1.9391E-23	0.503	0.212
SLC20A1	13	0.38940399	9.41462E-26	1.71789E-21	0.548	0.243
C5orf15	13	0.3891403	1.21932E-22	2.22489E-18	0.683	0.383
RCN2	13	0.38894842	1.6143E-18	2.9456E-14	0.854	0.646
C9orf142	13	0.38892932	3.29261E-32	6.00802E-28	0.598	0.258
SLBP	13	0.38879238	5.68843E-13	1.03797E-08	0.457	0.267
PSMC3IP	13	0.38871437	2.8889E-110	5.2714E-106	0.422	0.053
CDC25B	13	0.38870953	2.60503E-85	4.7534E-81	0.457	0.078
CNN3	13	0.38662	4.19189E-24	7.64894E-20	0.633	0.319
ARL2	13	0.38653383	2.64671E-24	4.82946E-20	0.764	0.487
FDPS	13	0.38605438	4.64235E-29	8.4709E-25	0.538	0.229
RP5-1198O20.4	13	0.38559947	1.8282E-226	3.3358E-222	0.412	0.024
LRR1	13	0.38465676	3.6676E-178	6.6923E-174	0.432	0.035
KIF23	13	0.38391728	0	0	0.352	0.003
GNB1	13	0.38379988	2.30107E-32	4.19875E-28	0.543	0.218

MANF	13	0.38333975	9.80087E-24	1.78836E-19	0.633	0.331
TRAPPC1	13	0.38194982	1.75833E-20	3.20843E-16	0.709	0.452
SRSF7	13	0.38091905	6.20012E-19	1.13134E-14	0.653	0.399
SURF4	13	0.38075916	1.64991E-35	3.01059E-31	0.628	0.26
PSMA4	13	0.38074791	2.91143E-13	5.31248E-09	0.613	0.405
CCDC85B	13	0.38039853	5.93151E-18	1.08232E-13	0.955	0.795
CENPE	13	0.38035117	0	0	0.342	0.003
SNRPF	13	0.38004021	2.84684E-22	5.19462E-18	0.854	0.59
C1orf122	13	0.37850342	1.80016E-16	3.28475E-12	0.633	0.402
ACADVL	13	0.37823335	2.00656E-26	3.66137E-22	0.593	0.288
PYURF	13	0.37665211	1.96442E-18	3.58447E-14	0.834	0.625
H2AFY	13	0.3766496	1.32786E-30	2.42294E-26	0.633	0.288
ATAD2	13	0.37641419	3.2399E-141	5.9119E-137	0.312	0.023
COMMD4	13	0.3756922	1.63306E-43	2.97985E-39	0.503	0.157
HELLS	13	0.37425296	3.5585E-177	6.4933E-173	0.342	0.021
CRLF1	13	0.37377822	6.95076E-21	1.2683E-16	0.548	0.272
PPP1CA	13	0.3723007	9.72846E-26	1.77515E-21	0.618	0.309
TRIM59	13	0.37209639	2.3988E-134	4.3771E-130	0.337	0.028
ID1	13	0.37199313	2.35359E-11	4.2946E-07	0.774	0.602
CBFB	13	0.37191413	3.0291E-39	5.5272E-35	0.543	0.191
TUBG1	13	0.37145242	4.98759E-85	9.10086E-81	0.422	0.067
PRRX1	13	0.3713366	1.18135E-31	2.15562E-27	0.533	0.207
CYGB	13	0.37087	1.65008E-18	3.01091E-14	0.337	0.131
SNF8	13	0.37050177	1.7021E-21	3.10582E-17	0.668	0.385
HSPB11	13	0.37036399	4.50947E-28	8.22842E-24	0.548	0.238
BIRC2	13	0.37035289	6.92886E-14	1.26431E-09	0.653	0.424
ANAPC15	13	0.37011645	4.4672E-46	8.15131E-42	0.467	0.131
OSTC	13	0.36941269	1.3808E-19	2.51955E-15	0.925	0.808
OAZ2	13	0.36932143	2.9406E-25	5.36572E-21	0.653	0.34
FBN1	13	0.36872878	6.42081E-42	1.17161E-37	0.477	0.142
MZT2A	13	0.36864915	8.55501E-26	1.56103E-21	0.94	0.783
IL11	13	0.36846256	2.26086E-18	4.1254E-14	0.261	0.085
MIS18BP1	13	0.36813679	5.00701E-98	9.13629E-94	0.442	0.064
CDK2AP2	13	0.36602715	1.11871E-26	2.0413E-22	0.618	0.286
NOP10	13	0.36595534	1.59817E-18	2.91619E-14	0.915	0.761
TMED2	13	0.36573751	5.88104E-18	1.07311E-13	0.92	0.825
PLEC	13	0.36536627	4.31071E-53	7.86575E-49	0.472	0.118
CDCA5	13	0.36508607	0	0	0.352	0.004
KNSTRN	13	0.36498828	5.3741E-255	9.8061E-251	0.352	0.015

GPAA1	13	0.36493664	3.95293E-25	7.21291E-21	0.683	0.359
PPIC	13	0.36443273	4.15236E-17	7.57681E-13	0.739	0.472
TIMM8B	13	0.36439254	4.60814E-19	8.40847E-15	0.714	0.477
COTL1	13	0.36370703	6.33902E-48	1.15668E-43	0.392	0.092
TAF9	13	0.36370013	4.31012E-25	7.86468E-21	0.578	0.282
GPX8	13	0.3636764	7.56914E-25	1.38114E-20	0.603	0.294
SMIM14	13	0.36287382	9.84118E-22	1.79572E-17	0.653	0.351
GANAB	13	0.36257231	1.2235E-22	2.23252E-18	0.568	0.284
PSIP1	13	0.3625262	2.59622E-29	4.73732E-25	0.538	0.221
COX7A2	13	0.36243735	9.42913E-29	1.72053E-24	1	0.979
HIGD2A	13	0.36237804	9.51987E-23	1.73709E-18	0.653	0.356
FAP	13	0.36215559	6.49554E-21	1.18524E-16	0.513	0.237
CMTM6	13	0.36212869	7.61964E-40	1.39036E-35	0.523	0.174
ATP5E	13	0.36171559	4.1322E-22	7.54003E-18	1	0.996
TAX1BP3	13	0.36105021	2.00893E-21	3.66569E-17	0.503	0.238
CDC42BPA	13	0.36102889	8.25895E-39	1.50701E-34	0.487	0.158
FAM127A	13	0.36047795	1.32213E-36	2.4125E-32	0.643	0.262
MTCH2	13	0.36010482	3.91715E-28	7.14762E-24	0.658	0.326
MRPL51	13	0.35954193	6.38158E-27	1.16445E-22	0.955	0.814
C8orf88	13	0.35952466	9.28985E-84	1.69512E-79	0.437	0.072
TGOLN2	13	0.3592458	4.3327E-21	7.90587E-17	0.658	0.373
LMAN1	13	0.35900055	2.71314E-24	4.95067E-20	0.663	0.343
PRDX1	13	0.35889683	2.73028E-07	0.004981938	0.91	0.83
CLSPN	13	0.35808435	0	0	0.307	0.007
MCUR1	13	0.35734276	2.92287E-40	5.33336E-36	0.508	0.166
ITGA5	13	0.3565193	8.55484E-32	1.561E-27	0.462	0.161
ECT2	13	0.35606054	0	0	0.362	0.007
CTSZ	13	0.35584124	1.33832E-34	2.44204E-30	0.548	0.207
HNRNPUL1	13	0.35579125	1.26493E-26	2.30812E-22	0.593	0.279
GSPT1	13	0.35549522	2.49116E-26	4.54562E-22	0.563	0.262
YIF1B	13	0.35478356	5.63186E-38	1.02765E-33	0.548	0.197
PRKDC	13	0.35440899	3.34504E-44	6.10369E-40	0.477	0.14
TGFB1	13	0.3531186	1.63141E-47	2.97684E-43	0.492	0.138
NUTF2	13	0.35311509	6.22966E-30	1.13673E-25	0.623	0.28
SGOL1	13	0.35308724	0	0	0.352	0.003
USP1	13	0.35283922	1.10768E-32	2.02118E-28	0.487	0.178
NDUFAF3	13	0.35255962	8.53803E-19	1.55793E-14	0.854	0.651
PSRC1	13	0.35254959	2.1187E-173	3.8659E-169	0.382	0.027
SQLE	13	0.35248673	1.74492E-25	3.18396E-21	0.497	0.208

C1QL1	13	0.35191293	4.39119E-55	8.0126E-51	0.322	0.057
MRPL18	13	0.35118553	3.79617E-23	6.92688E-19	0.608	0.324
SPC24	13	0.35117337	0	0	0.327	0.004
MKKS	13	0.35099949	7.77945E-25	1.41952E-20	0.613	0.315
SRSF9	13	0.35081217	5.48119E-15	1.00015E-10	0.724	0.543
NME4	13	0.3505448	5.82501E-18	1.06289E-13	0.714	0.447
ATL3	13	0.35023834	8.64772E-35	1.57795E-30	0.538	0.199
CNIH4	13	0.34961193	3.61437E-21	6.59514E-17	0.769	0.485
TLN1	13	0.34885369	1.51646E-26	2.76709E-22	0.497	0.209
ILF2	13	0.34883805	4.39193E-22	8.01395E-18	0.578	0.296
BCL2L12	13	0.34834016	1.17242E-70	2.13931E-66	0.467	0.093
NHP2	13	0.34804755	6.17442E-16	1.12665E-11	0.698	0.488
FOXP1	13	0.3480353	7.89651E-20	1.44088E-15	0.533	0.267
ANKRD11	13	0.34760992	9.74253E-41	1.77772E-36	0.487	0.151
RP11-844P9.2	13	0.34714221	1.9023E-252	3.4711E-248	0.312	0.011
CDK16	13	0.3463359	1.33094E-44	2.42856E-40	0.503	0.149
RRM1	13	0.34618186	1.56663E-38	2.85864E-34	0.407	0.116
NDUFB2	13	0.34591422	8.96992E-16	1.63674E-11	0.874	0.77
GNAS	13	0.34486287	5.05086E-19	9.2163E-15	0.869	0.622
SERTAD4-AS1	13	0.34443698	5.27667E-25	9.62834E-21	0.492	0.207
DLGAP5	13	0.3438686	0	0	0.317	0.001
SHFM1	13	0.34366312	2.18994E-17	3.99599E-13	0.935	0.859
RTN3	13	0.34331418	9.43945E-29	1.72242E-24	0.588	0.261
UCP2	13	0.34321241	7.7511E-155	1.4143E-150	0.362	0.028
TMEM165	13	0.34293864	7.63059E-25	1.39235E-20	0.678	0.354
TMBIM6	13	0.34277791	3.13277E-11	5.71636E-07	0.859	0.697
UBE2A	13	0.34272702	4.1606E-24	7.59185E-20	0.663	0.355
SPDL1	13	0.34266923	6.7513E-188	1.2319E-183	0.367	0.023
SEC61B	13	0.34211308	6.79015E-14	1.239E-09	0.889	0.815
PSMG3	13	0.34137367	5.76609E-30	1.05214E-25	0.568	0.238
GNB2	13	0.34099903	9.05926E-19	1.65304E-14	0.739	0.492
EIF4EBP1	13	0.33951052	2.58376E-15	4.71459E-11	0.754	0.537
PXDN	13	0.33926289	1.52272E-36	2.77851E-32	0.387	0.11
FIBP	13	0.33924674	1.10516E-20	2.01659E-16	0.698	0.414
DEF8	13	0.33922723	5.92893E-49	1.08185E-44	0.457	0.119
SNX7	13	0.33917761	7.79554E-35	1.42245E-30	0.603	0.237
GCLM	13	0.3387074	3.89287E-49	7.10332E-45	0.437	0.108
CD47	13	0.33809858	2.58585E-12	4.7184E-08	0.673	0.457
SUMO1	13	0.33750387	3.76929E-21	6.87782E-17	0.905	0.686

SLC25A39	13	0.33723767	1.72867E-24	3.1543E-20	0.543	0.252
DSTN	13	0.33713727	6.69146E-18	1.22099E-13	1	0.997
KDELC2	13	0.3370335	1.33778E-43	2.44106E-39	0.477	0.139
MRPS16	13	0.3369824	1.53335E-22	2.7979E-18	0.663	0.376
CXXC5	13	0.3368614	2.90943E-19	5.30884E-15	0.648	0.349
IER3IP1	13	0.33654565	4.07986E-19	7.44452E-15	0.854	0.629
MT2A	13	0.33646732	9.63299E-21	1.75773E-16	1	0.996
CCDC18	13	0.33606525	4.4261E-207	8.0763E-203	0.357	0.02
GALNT1	13	0.33597558	6.88426E-20	1.25617E-15	0.508	0.262
TMA7	13	0.33564793	1.1958E-17	2.18198E-13	0.985	0.989
COX6C	13	0.33495924	5.90577E-17	1.07763E-12	0.925	0.839
RBBP7	13	0.33476572	4.18156E-28	7.63009E-24	0.538	0.224
PARPBP	13	0.33476093	0	0	0.367	0.01
KIF14	13	0.33436739	0	0	0.322	0.003
SHCBP1	13	0.33407299	0	0	0.317	0.004
SDF2L1	13	0.33313091	7.51043E-21	1.37043E-16	0.533	0.257
MLLT11	13	0.33242095	2.02094E-40	3.68761E-36	0.402	0.108
PCSK1	13	0.33221748	3.04338E-93	5.55326E-89	0.327	0.037
SERPINH1	13	0.33145404	4.73732E-24	8.6442E-20	0.407	0.155
CALD1	13	0.33053464	8.09882E-17	1.47779E-12	0.794	0.56
CDCA8	13	0.33026158	0	0	0.291	0.004
PLIN3	13	0.32984054	1.78655E-23	3.25992E-19	0.518	0.235
POMP	13	0.32977039	2.66349E-17	4.86007E-13	0.94	0.846
RALY	13	0.32976355	2.52286E-24	4.60346E-20	0.583	0.28
ITGB3BP	13	0.32959246	3.39993E-21	6.20385E-17	0.623	0.333
VKORC1	13	0.32933544	2.68558E-16	4.90039E-12	1	0.949
RAD51AP1	13	0.32922788	0	0	0.312	0.008
VBP1	13	0.32879202	2.3474E-27	4.2833E-23	0.578	0.26
SON	13	0.32790165	9.65669E-17	1.76206E-12	0.789	0.54
DCTN3	13	0.32772772	1.19722E-17	2.18457E-13	0.759	0.512
CENPU	13	0.32607671	0	0	0.327	0.008
DBF4	13	0.32332034	2.96755E-73	5.41489E-69	0.322	0.045
CKAP2L	13	0.32184242	0	0	0.307	0.002
CDK2AP1	13	0.32143997	1.79748E-29	3.27986E-25	0.477	0.179
SLC35B2	13	0.32139365	3.39017E-33	6.18605E-29	0.568	0.218
EFEMP2	13	0.32120603	9.67139E-16	1.76474E-11	0.658	0.422
DR1	13	0.32109618	9.77207E-30	1.78311E-25	0.513	0.2
HACD3	13	0.32107902	7.30318E-18	1.33261E-13	0.442	0.208
LRRFIP1	13	0.32094176	4.13096E-18	7.53775E-14	0.633	0.363

SUB1	13	0.32085214	1.96433E-17	3.58431E-13	0.935	0.837
JAG1	13	0.32054874	8.48208E-43	1.54773E-38	0.382	0.094
DDAH2	13	0.32003325	4.16213E-16	7.59463E-12	0.533	0.301
SRSF10	13	0.31986702	2.09371E-20	3.8204E-16	0.643	0.36
COPZ2	13	0.31979037	1.076E-20	1.96338E-16	0.568	0.283
TMEM167A	13	0.31960355	1.12595E-17	2.05451E-13	0.869	0.704
BARD1	13	0.31882963	3.948E-103	7.2039E-99	0.337	0.036
COL6A2	13	0.31847855	3.63926E-11	6.64055E-07	0.794	0.616
MRPS11	13	0.3183305	2.76045E-32	5.037E-28	0.508	0.187
TMEM50A	13	0.31806609	2.69096E-15	4.91019E-11	0.759	0.507
KDELR3	13	0.31707174	1.38688E-18	2.53064E-14	0.543	0.279
EIF1AX	13	0.31686129	5.29316E-16	9.65843E-12	0.809	0.609
SDC1	13	0.31678276	2.8341E-172	5.1713E-168	0.327	0.02
GMNN	13	0.31641415	8.72745E-53	1.5925E-48	0.387	0.083
PRR34-AS1	13	0.31629545	9.22186E-26	1.68271E-21	0.492	0.2
HINT2	13	0.3162397	7.63265E-21	1.39273E-16	0.608	0.322
EIF4E	13	0.31582474	3.35769E-20	6.12677E-16	0.613	0.337
MCFD2	13	0.3156741	9.50219E-17	1.73387E-12	0.543	0.303
POLD2	13	0.31565957	1.00738E-18	1.83817E-14	0.633	0.366
SCD	13	0.31525608	4.87015E-28	8.88656E-24	0.442	0.159
NDUFS8	13	0.31452682	7.65618E-19	1.39702E-14	0.643	0.372
FAM114A1	13	0.31404239	1.76965E-21	3.22907E-17	0.633	0.335
BRI3	13	0.31313591	1.373E-13	2.50532E-09	0.93	0.834
PDCD5	13	0.31301668	4.28637E-14	7.82133E-10	0.784	0.556
METTL9	13	0.31265056	2.50994E-21	4.57988E-17	0.578	0.297
RNH1	13	0.31246286	7.98483E-07	0.014569911	0.658	0.538
PHLDA1	13	0.3124019	3.21129E-10	5.85964E-06	0.402	0.22
CLMP	13	0.31210734	6.5982E-18	1.20397E-13	0.432	0.2
NAV1	13	0.31201371	2.73117E-31	4.98357E-27	0.422	0.139
TUBA1A	13	0.31179043	5.94893E-14	1.0855E-09	0.387	0.187
RAB32	13	0.31143778	8.3069E-24	1.51576E-19	0.598	0.285
REEP4	13	0.31118095	1.5626E-169	2.8512E-165	0.317	0.019
DCK	13	0.31111096	2.76527E-23	5.04578E-19	0.432	0.174
NDC80	13	0.31094714	0	0	0.312	0.001
VDAC3	13	0.31065005	1.76533E-25	3.2212E-21	0.558	0.248
PRDX3	13	0.31041899	2.65064E-19	4.83662E-15	0.653	0.369
ALKBH5	13	0.31026667	1.77785E-13	3.24405E-09	0.613	0.384
RPSA	13	0.31025801	1.54392E-16	2.81718E-12	1	0.999
SOX4	13	0.30979776	9.41488E-34	1.71793E-29	0.332	0.086

RFC3	13	0.30977768	7.7561E-117	1.4152E-112	0.352	0.035
UBE2Q2	13	0.30917426	6.17898E-20	1.12748E-15	0.598	0.312
SLIRP	13	0.30821455	9.6815E-13	1.76658E-08	0.849	0.705
OIP5	13	0.30807393	0	0	0.317	0.008
PPP4C	13	0.30785413	3.69456E-18	6.74147E-14	0.568	0.309
C6orf62	13	0.30739853	5.85366E-18	1.06812E-13	0.593	0.335
CHSY1	13	0.30736042	1.85446E-29	3.38384E-25	0.457	0.165
GTF2A2	13	0.30676133	1.86065E-14	3.39514E-10	0.673	0.44
MRPL14	13	0.30658153	5.42804E-14	9.90454E-10	0.774	0.566
GTF3C6	13	0.30641595	4.25069E-17	7.75623E-13	0.618	0.362
NCAPG	13	0.30623183	0	0	0.317	0.003
NCL	13	0.30551962	1.43746E-12	2.62294E-08	0.799	0.619
YWHAG	13	0.30542932	2.94952E-22	5.38198E-18	0.518	0.235
EZH2	13	0.30514717	5.9542E-265	1.0865E-260	0.307	0.01
RNF26	13	0.30423056	4.77985E-53	8.72179E-49	0.377	0.079
ARHGAP11B	13	0.30411114	0	0	0.317	0.008
TGFBR1	13	0.3040739	1.93121E-37	3.52388E-33	0.387	0.107
FAAP20	13	0.3038621	1.74296E-15	3.18037E-11	0.739	0.51
LTBP2	13	0.30375829	1.61389E-38	2.94487E-34	0.412	0.115
SCX	13	0.30310352	4.70075E-17	8.57746E-13	0.206	0.063
LMNB1	13	0.30292166	0	0	0.312	0.006
RTF1	13	0.30253496	3.43919E-15	6.27549E-11	0.714	0.481
EIF4E2	13	0.30245887	3.04983E-19	5.56502E-15	0.553	0.288
SPATA33	13	0.30239608	2.94814E-44	5.37947E-40	0.382	0.093
EVA1A	13	0.30189328	3.72014E-65	6.78814E-61	0.281	0.039
ANPEP	13	0.30187923	1.92116E-80	3.50553E-76	0.236	0.023
EZR	13	0.30176339	9.60343E-30	1.75234E-25	0.417	0.14
LOXL2	13	0.30172913	6.40877E-43	1.16941E-38	0.407	0.104
SLC2A4RG	13	0.30159544	3.68118E-33	6.71706E-29	0.417	0.132
G2E3	13	0.30084427	4.49097E-57	8.19467E-53	0.377	0.074
FLOT1	13	0.30077964	4.93406E-14	9.00318E-10	0.698	0.479
TXNL4A	13	0.30000086	2.03435E-15	3.71209E-11	0.688	0.448
GINS2	13	0.29974424	3.36759E-84	6.14484E-80	0.317	0.038
TXNDC12	13	0.29909041	4.07627E-25	7.43797E-21	0.588	0.274
PCMT1	13	0.29907995	5.76455E-12	1.05186E-07	0.643	0.436
RAC1	13	0.29890413	2.627E-18	4.79348E-14	0.985	0.926
MELK	13	0.29889807	0	0	0.291	0.002
FBXO5	13	0.2979038	3.32045E-64	6.05882E-60	0.296	0.044
CLIC4	13	0.29740471	1.5749E-15	2.87371E-11	0.729	0.472

KIF11	13	0.2970576	0	0	0.302	0.004
LSM2	13	0.2969359	5.58075E-12	1.01832E-07	0.704	0.53
HMGXB4	13	0.29648134	9.86393E-30	1.79987E-25	0.437	0.153
MLEC	13	0.29640477	1.03322E-17	1.88532E-13	0.673	0.4
KIF20A	13	0.29591081	0	0	0.296	0.002
NCAM1	13	0.29583869	2.9117E-147	5.3129E-143	0.302	0.02
IQGAP1	13	0.29579286	8.71123E-16	1.58954E-11	0.663	0.394
EIF5	13	0.29573884	1.18163E-15	2.15612E-11	0.864	0.648
PIGX	13	0.294713	1.3416E-22	2.44801E-18	0.407	0.161
HOMER3	13	0.29433035	4.7964E-31	8.752E-27	0.412	0.135
TIMM10	13	0.29426836	1.70412E-21	3.10951E-17	0.593	0.303
SEPT4	13	0.29376466	3.28719E-15	5.99813E-11	0.673	0.437
ACTL6A	13	0.29292192	2.0414E-17	3.72494E-13	0.462	0.222
AES	13	0.29289729	6.22771E-20	1.13637E-15	0.563	0.281
UQCR10	13	0.29257898	4.04457E-14	7.38012E-10	0.899	0.804
NOP56	13	0.29246545	1.53591E-22	2.80257E-18	0.452	0.19
E2F1	13	0.29228817	3.7977E-263	6.9296E-259	0.271	0.008
SCOC	13	0.29114814	3.44782E-17	6.29123E-13	0.799	0.538
AK6	13	0.29090273	6.24101E-17	1.1388E-12	0.543	0.294
NEK2	13	0.28893645	0	0	0.271	0.001
GCSH	13	0.28878848	8.79649E-21	1.6051E-16	0.523	0.252
CCDC74A	13	0.28877071	1.72209E-41	3.1423E-37	0.432	0.119
LIMK2	13	0.28868649	4.20121E-83	7.66595E-79	0.337	0.044
ALYREF	13	0.28847326	9.60375E-78	1.7524E-73	0.347	0.049
IDI1	13	0.28845587	1.42798E-16	2.60563E-12	0.563	0.294
IMPAD1	13	0.28817406	3.93837E-29	7.18634E-25	0.477	0.176
ROMO1	13	0.28809863	1.09992E-11	2.00702E-07	0.884	0.826
SYNCRIP	13	0.28741168	3.31314E-30	6.04549E-26	0.573	0.229
TUSC3	13	0.28731718	3.53224E-23	6.44529E-19	0.528	0.232
RAD23A	13	0.28725286	2.05976E-15	3.75845E-11	0.874	0.635
ZWILCH	13	0.28667772	5.7536E-165	1.0499E-160	0.332	0.021
WSB2	13	0.28654936	2.26772E-38	4.13792E-34	0.422	0.12
FAM122B	13	0.28592843	3.92774E-57	7.16695E-53	0.382	0.075
HNRNPM	13	0.28549068	3.91742E-18	7.14812E-14	0.628	0.343
ASF1B	13	0.2850338	0	0	0.266	0.005
MCM7	13	0.28496031	1.51686E-29	2.76781E-25	0.281	0.073
DAZAP1	13	0.28487514	1.7049E-37	3.11093E-33	0.437	0.131
C2orf69	13	0.28478885	2.32169E-35	4.23638E-31	0.412	0.123
TP53I13	13	0.2840536	4.24594E-20	7.74757E-16	0.598	0.312

SYPL1	13	0.2837631	4.59445E-11	8.38349E-07	0.819	0.627
METRN	13	0.28313265	5.6926E-23	1.03873E-18	0.477	0.196
UBE2D2	13	0.28285097	3.73644E-14	6.81789E-10	0.814	0.597
RP11-783K16.5	13	0.28282207	2.1044E-107	3.8399E-103	0.312	0.029
MYADM	13	0.28260288	1.8014E-22	3.28702E-18	0.457	0.193
MGST3	13	0.28260189	3.3697E-12	6.1487E-08	0.839	0.658
PSMD14	13	0.28247362	1.85948E-27	3.39299E-23	0.472	0.18
HNRNPA0	13	0.28229742	6.83158E-12	1.24656E-07	0.879	0.681
APITD1	13	0.2820987	1.32071E-42	2.40991E-38	0.372	0.09
TRIOBP	13	0.28147123	8.05213E-45	1.46927E-40	0.442	0.116
PMM1	13	0.27984888	4.46571E-14	8.14857E-10	0.548	0.316
RECQL	13	0.27970195	7.20812E-43	1.31526E-38	0.432	0.115
TRA2B	13	0.27969001	2.89956E-14	5.29083E-10	0.633	0.385
PCOLCE	13	0.2796762	2.91404E-12	5.31725E-08	0.482	0.265
SNRNP25	13	0.27942488	3.01023E-17	5.49276E-13	0.497	0.257
PPP2CA	13	0.27940763	2.54893E-14	4.65104E-10	0.608	0.364
PPM1G	13	0.27938778	3.51523E-21	6.41424E-17	0.477	0.217
POP7	13	0.27916034	3.02755E-18	5.52438E-14	0.653	0.365
C4orf46	13	0.27893802	1.7606E-99	3.21258E-95	0.317	0.033
OST4	13	0.27877779	9.69411E-14	1.76888E-09	1	0.999
HK1	13	0.27865019	1.02676E-34	1.87353E-30	0.427	0.131
SNRPE	13	0.27863462	9.2557E-13	1.68889E-08	0.849	0.668
MYO1C	13	0.27863228	2.9092E-23	5.30842E-19	0.447	0.181
CAP1	13	0.2779876	6.57598E-16	1.19992E-11	0.503	0.263
RER1	13	0.27754007	1.19995E-12	2.18955E-08	0.779	0.576
FAT1	13	0.27746349	3.57732E-53	6.52754E-49	0.332	0.062
FOSL2	13	0.27700349	1.16048E-13	2.11753E-09	0.482	0.265
FAM83D	13	0.27648811	0	0	0.256	0.002
TXNDC9	13	0.27621925	1.19284E-21	2.17658E-17	0.422	0.172
CYB5R3	13	0.27605982	1.83965E-12	3.35681E-08	0.844	0.655
ARPP19	13	0.27568766	2.84489E-12	5.19107E-08	0.759	0.549
RB1	13	0.27539692	3.12685E-37	5.70556E-33	0.377	0.102
PSENEN	13	0.27495403	9.66024E-18	1.7627E-13	0.538	0.279
TMCO3	13	0.27480432	2.16696E-13	3.95406E-09	0.608	0.373
LSM1	13	0.27459323	3.30295E-21	6.0269E-17	0.583	0.288
SRRM2	13	0.27456111	3.74434E-15	6.8323E-11	0.688	0.427
TMEM19	13	0.27455203	7.64723E-52	1.39539E-47	0.307	0.055
KLF10	13	0.27429514	1.94325E-19	3.54585E-15	0.442	0.192
MND1	13	0.27413257	0	0	0.271	0.005

ARF5	13	0.27340713	5.68869E-16	1.03802E-11	0.709	0.437
EMC6	13	0.2733033	1.08508E-12	1.97994E-08	0.583	0.369
SHC1	13	0.27328185	5.41028E-26	9.87215E-22	0.437	0.162
USMG5	13	0.27303093	2.1319E-13	3.89008E-09	0.975	0.929
FAM96A	13	0.27234679	3.22414E-16	5.8831E-12	0.543	0.297
G3BP1	13	0.27196211	5.94863E-21	1.08545E-16	0.573	0.284
PTPN14	13	0.27188545	2.15231E-27	3.92733E-23	0.432	0.155
PLK1	13	0.27172097	0	0	0.261	0.003
PTS	13	0.27149041	1.24364E-15	2.26928E-11	0.543	0.308
ATP5B	13	0.27133929	7.26294E-10	1.32527E-05	0.628	0.426
DESI2	13	0.2712917	2.70003E-31	4.92674E-27	0.402	0.127
TPM1	13	0.27127784	4.56561E-07	0.008330867	0.749	0.614
ARHGDIB	13	0.27084706	1.32258E-26	2.41331E-22	0.291	0.082
ENG	13	0.27073591	2.14221E-36	3.9089E-32	0.372	0.099
POC1A	13	0.27053636	3.9399E-307	7.1892E-303	0.307	0.008
NRM	13	0.26961649	9.9452E-65	1.8147E-60	0.322	0.05
DLEU2	13	0.26957853	2.32288E-47	4.23857E-43	0.382	0.086
CCM2	13	0.26923876	2.42953E-35	4.43316E-31	0.347	0.091
NRAS	13	0.26882834	6.97104E-35	1.27201E-30	0.417	0.125
FGD5-AS1	13	0.26839078	1.43584E-23	2.61998E-19	0.462	0.189
HNRNPA1	13	0.2682049	2.26895E-11	4.14015E-07	0.995	0.989
CCDC109B	13	0.26780958	1.30098E-17	2.3739E-13	0.523	0.269
CNPY3	13	0.26778846	3.28667E-14	5.99719E-10	0.543	0.308
UBL5	13	0.26679808	8.99394E-14	1.64112E-09	0.98	0.949
GGCT	13	0.26671218	6.09683E-15	1.11249E-10	0.442	0.227
LMO7	13	0.2667053	2.38524E-46	4.35235E-42	0.332	0.069
WBP5	13	0.26657066	1.12367E-09	2.05037E-05	0.874	0.745
MMP14	13	0.26590684	2.97729E-25	5.43267E-21	0.477	0.185
PLAU	13	0.26560158	2.65807E-78	4.85018E-74	0.181	0.014
SFN	13	0.26515563	9.94454E-26	1.81458E-21	0.226	0.055
CSE1L	13	0.26504656	1.55785E-41	2.84262E-37	0.407	0.106
SAE1	13	0.26484677	4.17648E-36	7.62083E-32	0.417	0.122
SGCB	13	0.26460926	7.96227E-12	1.45288E-07	0.638	0.417
GOLM1	13	0.26447156	1.01275E-20	1.84797E-16	0.508	0.23
SAR1A	13	0.26403019	3.41681E-12	6.23465E-08	0.734	0.519
COQ2	13	0.2638018	2.16173E-78	3.94451E-74	0.317	0.041
BUD31	13	0.26374864	1.23553E-13	2.25448E-09	0.683	0.454
UQCRFS1	13	0.26372106	2.59062E-14	4.7271E-10	0.719	0.494
DPYSL2	13	0.26364587	2.62622E-19	4.79206E-15	0.543	0.263

EXOSC8	13	0.26343078	2.08474E-26	3.80403E-22	0.442	0.162
PDZRN3	13	0.26283795	2.35986E-64	4.30603E-60	0.261	0.034
ZEB1	13	0.26269574	6.49135E-19	1.18448E-14	0.407	0.174
PRRX2	13	0.26256535	1.39183E-21	2.53967E-17	0.342	0.123
C21orf58	13	0.26226793	9.1223E-181	1.6645E-176	0.271	0.013
MIF	13	0.26215841	1.4056E-17	2.5648E-13	1	1
XPO1	13	0.2620599	4.96004E-27	9.05059E-23	0.472	0.179
MFSD14B	13	0.26201936	2.26373E-42	4.13063E-38	0.377	0.092
DAP	13	0.26112023	1.74899E-12	3.19138E-08	0.709	0.482
EPN1	13	0.26108799	4.15806E-16	7.58721E-12	0.447	0.222
ACTR2	13	0.26097054	1.05224E-17	1.92003E-13	0.598	0.322
CD70	13	0.26095625	1.37548E-53	2.50983E-49	0.216	0.028
SPTSSA	13	0.26088635	7.01176E-13	1.27944E-08	0.628	0.408
TMEM14C	13	0.26085682	3.41458E-11	6.23059E-07	0.814	0.609
MBNL1	13	0.26079485	4.28587E-13	7.82042E-09	0.593	0.368
ENSA	13	0.26068993	3.61365E-17	6.59382E-13	0.508	0.258
HYLS1	13	0.26061053	7.8261E-101	1.42803E-96	0.281	0.026
MGAT2	13	0.2604525	1.69418E-26	3.09137E-22	0.422	0.151
GAS2L1	13	0.26006527	9.77195E-25	1.78309E-20	0.372	0.129
APLP2	13	0.26001925	3.71038E-14	6.77032E-10	0.608	0.36
ZFR	13	0.25987762	3.70704E-10	6.76423E-06	0.503	0.304
RNPS1	13	0.25984739	2.58788E-16	4.7221E-12	0.618	0.352
UBE2K	13	0.2596494	7.72226E-16	1.40908E-11	0.553	0.307
SUZ12	13	0.25942403	2.45709E-34	4.48346E-30	0.402	0.118
DDX5	13	0.25937457	1.50939E-11	2.75419E-07	0.799	0.585
UHRF2	13	0.25936266	1.91145E-31	3.48782E-27	0.357	0.103
PHF20	13	0.25934689	1.48549E-26	2.71057E-22	0.432	0.158
FAM111A	13	0.25930999	1.68787E-44	3.07986E-40	0.266	0.048
CRAT	13	0.25909785	1.3946E-38	2.54473E-34	0.347	0.085
ICMT	13	0.25878389	6.02667E-58	1.09969E-53	0.317	0.053
PABPN1	13	0.2581715	1.34584E-23	2.45576E-19	0.492	0.206
UBE2M	13	0.25810274	1.54305E-21	2.81561E-17	0.427	0.175
VMP1	13	0.25806377	4.86819E-09	8.88298E-05	0.714	0.539
ORC6	13	0.25804667	5.5285E-163	1.0088E-158	0.281	0.015
MYBL1	13	0.25796566	2.2625E-227	4.1283E-223	0.271	0.009
UHRF1	13	0.25789249	7.8307E-190	1.4289E-185	0.231	0.008
CAST	13	0.257609	7.82627E-14	1.42806E-09	0.623	0.393
HN1L	13	0.25636085	3.71379E-35	6.77655E-31	0.372	0.102
COX6A1	13	0.25616858	2.59212E-14	4.72984E-10	0.95	0.836

HNRNPD	13	0.25614323	7.79509E-14	1.42237E-09	0.658	0.414
GNPNAT1	13	0.25609792	8.81767E-26	1.60896E-21	0.457	0.172
KRTCAP2	13	0.25608257	1.89502E-10	3.45784E-06	0.879	0.777
VCL	13	0.25563071	5.11571E-33	9.33464E-29	0.377	0.109
NPM3	13	0.25529278	1.58491E-22	2.89199E-18	0.417	0.163
DDX21	13	0.2551815	2.26621E-12	4.13515E-08	0.508	0.29
PNKD	13	0.25485896	5.36037E-14	9.78106E-10	0.618	0.372
MAPRE1	13	0.25482667	4.1346E-21	7.54441E-17	0.462	0.196
DHCR24	13	0.25458814	3.04085E-42	5.54864E-38	0.312	0.066
ZNHIT1	13	0.25420972	2.25164E-11	4.10857E-07	0.734	0.562
PDGFC	13	0.2538918	1.76716E-42	3.22454E-38	0.357	0.083
NIPA2	13	0.25380861	4.44651E-29	8.11354E-25	0.387	0.124
CERCAM	13	0.25360354	1.6843E-34	3.07334E-30	0.372	0.103
C7orf49	13	0.25322277	1.68804E-25	3.08016E-21	0.367	0.125
DCAF7	13	0.25322026	2.96634E-31	5.41269E-27	0.367	0.108
CCP110	13	0.25317589	7.01198E-31	1.27948E-26	0.312	0.083
PKMYT1	13	0.25304174	0	0	0.221	0.003
CASC5	13	0.2527471	0	0	0.256	0.003
PKM	13	0.25259208	1.19359E-09	2.17793E-05	0.995	0.926
SPTBN1	13	0.25209613	6.40551E-17	1.16881E-12	0.432	0.203
WASF2	13	0.25188884	3.82811E-16	6.98515E-12	0.698	0.421
PHTF1	13	0.25181565	5.51129E-51	1.00565E-46	0.307	0.055
VCP	13	0.25140902	1.36726E-17	2.49483E-13	0.452	0.213
POLR2E	13	0.25132671	2.03903E-15	3.72062E-11	0.533	0.294
MBNL1-AS1	13	0.25121543	5.34621E-22	9.75523E-18	0.261	0.078
GNB4	13	0.2510872	9.57621E-82	1.74737E-77	0.281	0.031
MT-ND2	13	0.25043663	4.38722E-07	0.008005354	0.814	0.671
