# Supplementary material for: Single-Cell RNA-Seq Analysis of Cells from Degenerating and Non-Degenerating Intervertebral Discs from the Same Individual Reveals New Biomarkers for Intervertebral Disc Degeneration
Source: Int J Mol Sci. 2022 Apr 3;23(7):3993. doi: 10.3390/ijms23073993 (PMC8999935; doi:10.3390/ijms23073993)
Supplement: Supplementary file 1 [file ijms-23-03993-s001.zip › ijms-1655681-SI/Table S25.pdf]

Table S25. Classification of selected genes according to their expression pattern.

	nD vs D			scRNA-seq/ qPCR results Correlation	
	Genes	NP	AF	NP	AF
Group 1	UPP1			-	-
	PRG4			-	-
	MT1G			-	-
	MT2A			+	-
	FRZB			-	-
	ADIRF			-	+
	Vimentin			+	+
	MT-ND2			-	-
	MT-CYB			-	-
	CAPS			-	-
	SYF2 (p29)			+	+
	MGST1			-	+
	TAF1D			+	+
	MT1F			+	+
	TNFRSF11B			-	-
	S100A2			+	-
	MSMP			-	-
	SPTSSB			-	-
	HMGA1			-	-
	FGFBP2			-	-
Group 2	SOD2			-	+
	IGFBP3			-	-
	SLPI			-	-
	C2orf40			-	-
	S100B			-	-
	Col2A1			-	-
	FBXO2			-	-
	S100A1			+	-
	CLEC3A			-	-

**CHI3L2**			-	-
**PLA2G2A**			-	-
**CTGF**			-	-
**EPYC**			-	-

Legend

Homogenous Expression in 14 Clusters
Opposite expression within 14 clusters

(+): scRNA-seq result **validated** by qPCR
(-): scRNA-seq result **not validated** by qPCR

Group 1: Similar pattern of expression between NP and AF
Group 2: Opposite pattern of expression between NP and AF
