# Supplementary material for: Single-Cell RNA-Seq Analysis of Cells from Degenerating and Non-Degenerating Intervertebral Discs from the Same Individual Reveals New Biomarkers for Intervertebral Disc Degeneration
Source: Int J Mol Sci. 2022 Apr 3;23(7):3993. doi: 10.3390/ijms23073993 (PMC8999935; doi:10.3390/ijms23073993)
Supplement: Supplementary file 1 [file ijms-23-03993-s001.zip › ijms-1655681-SI/Table S26.pdf]

Table S26. Donors demographics.

Donor ID	Age	Sex	Cause of death	Disc level	Degeneration	Cell type	Assays
1	28	M	Suicide	S1-L5	Degenerate	NP & AF	Sc-RNA<br>qPCR<br>IHC<br>WB
				L5-L4	Non-degenerate		
				L4-L1			
2	53	F	Cardiac arrest	S1-L4	Non-degenerate	NP & AF	qPCR<br>IHC<br>WB
3	67	M	Cardiac arrest	L5-L4	Degenerate	NP & AF	qPCR
4	69	M	Cerebral hemorrhage	S1-L5	Degenerate	NP & AF	qPCR<br>IHC<br>WB
				L5-L4	Non-Degenerate		
5	20	F	Intoxication CVA	S1-L1	Non-degenerate	NP & AF	qPCR
					Degenerate		

6	53	M	Cerebral aneurysm rupture	S1-L4	Degenerate	NP & AF	qPCR IHC WB
				L4-L1	Non-degenerate		
7	76	F	Anoxia	S1-L2	Degenerate	AF	qPCR
8	72	M	Intracerebral hemorrhage	L2-L4	Degenerate	NP	qPCR
9	67	M	Hemorrhagic stroke	L2-L5	Degenerate	NP & AF	IHC WB
10	68	F	Cerebral hemorrhage	L2-L5	Degenerate	NP & AF	IHC WB
11	39	M	Gunshot	S1-L3	Non-degenerate	NP & AF	IHC WB
