# Supplementary material for: Single-Cell RNA-Seq Analysis of Cells from Degenerating and Non-Degenerating Intervertebral Discs from the Same Individual Reveals New Biomarkers for Intervertebral Disc Degeneration
Source: Int J Mol Sci. 2022 Apr 3;23(7):3993. doi: 10.3390/ijms23073993 (PMC8999935; doi:10.3390/ijms23073993)
Supplement: Supplementary file 1 [file ijms-23-03993-s001.zip › ijms-1655681-SI/Table S3-6.pdf]

Table S3. Variation in cell proportions between iAFnD and iAFD.

Clusters	iAFnD	iAFD	% Variation
**0**	18.56	17.92	-3.44827586
**1**	20.79	14.17	-31.8422318
**2**	11.31	14.29	20.8537439
**3**	8.18	9.93	17.6233635
**4**	9.45	7.12	-24.6560847
**5**	8.18	7.05	-13.8141809
**6**	3.16	7.86	59.7964377
**7**	5.53	3.78	-31.6455696
**8**	6.32	4.98	-21.2025316
**9**	2.65	3.27	18.9602446
**10**	1.63	3.17	48.5804416
**11**	2.11	2.26	6.63716814
**12**	1.47	3.23	54.4891641
**13**	0.67	0.97	30.9278351

Table S4. Variation in cell proportions between NPnD NPD and NPD.

Clusters	NPnD	NPD	% Variation
**0**	18.75	20.68	9.33268859
**1**	18.7	14.15	-24.3315508
**2**	11.09	12.95	14.3629344
**3**	7.42	11.55	35.7575758
**4**	9.57	4.28	-55.276907
**5**	7.34	5.62	-23.4332425
**6**	4.32	8.72	50.4587156
**7**	6.36	5.68	-10.6918239
**8**	6.83	2.25	-67.057101
**9**	2.87	2.41	-16.0278746
**10**	1.45	3.98	63.5678392
**11**	2.82	2	-29.0780142
**12**	1.16	3.07	62.2149837
**13**	1.32	2.66	50.3759398

Tables S5. Variation in cell proportions between iAFnD and NPnD.

Clusters	iAFnD	NPnD	% Variation
**0**	18.56	18.75	1.01333333
**1**	20.79	18.7	-10.0529101
**2**	11.31	11.09	-1.94518126
**3**	8.18	7.42	-9.29095355
**4**	9.45	9.57	1.2539185
**5**	8.18	7.34	-10.2689487
**6**	3.16	4.32	26.8518519
**7**	5.53	6.36	13.0503145
**8**	6.32	6.83	7.4670571
**9**	2.65	2.87	7.66550523
**10**	1.63	1.45	-11.0429448
**11**	2.11	2.82	25.177305
**12**	1.47	1.16	-21.0884354
**13**	0.67	1.32	49.2424242

Tables S6. Variation in cell proportions between iAFnD and NPD.

Clusters	iAFD	NPD	% Variation
**0**	17.92	20.68	15.401786
**1**	14.17	14.15	-0.14114326
**2**	14.29	12.95	-9.37718684
**3**	9.93	11.55	16.314199
**4**	7.12	4.28	-39.8876404
**5**	7.05	5.62	-20.2836879
**6**	7.86	8.72	10.941476
**7**	3.78	5.68	50.26455
**8**	4.98	2.25	-54.8192771
**9**	3.27	2.41	-26.2996942
**10**	3.17	3.98	25.55205
**11**	2.26	2	-11.5044248
**12**	3.23	3.07	-4.95356037
**13**	0.97	2.66	174.2268
