# Supplementary material for: Single-Cell RNA-Seq Analysis of Cells from Degenerating and Non-Degenerating Intervertebral Discs from the Same Individual Reveals New Biomarkers for Intervertebral Disc Degeneration
Source: Int J Mol Sci. 2022 Apr 3;23(7):3993. doi: 10.3390/ijms23073993 (PMC8999935; doi:10.3390/ijms23073993)
Supplement: Supplementary file 1 [file ijms-23-03993-s001.zip › ijms-1655681-SI/Table S8.pdf]

Table S8. NPnD vs iAFnD in total cell analysis.

Gene	logFC.AF	Pval.AF	Fdr.AF	logFC.NP	Pval.NP	Fdr.NP
**A4GALT**	0.259909053	1.84E-120	3.35E-116	0.162967	4.15E-89	7.57E-85
**ABHD14A**	-0.1259594	1.38E-23	2.52E-19	-0.27304	3.30E-154	6.03E-150
**ABI3BP**	0.117949862	4.67E-07	8.51E-03	4.46E-01	5.97E-81	1.09E-76
**AC003090.1**	-0.29652262	5.26E-128	9.60E-124	-3.60E-01	9.58E-276	1.75E-271
**AC005152.3**	-0.237572752	2.37E-94	4.33E-90	-2.85E-01	4.48E-175	8.17E-171
**AC090498.1**	-0.045082659	5.13E-07	9.37E-03	3.07E-01	1.39E-259	2.53E-255
**ACAN**	-0.242208226	3.61E-86	6.59E-82	-1.06304	0	0
**ACTG1**	-0.738803033	0.00E+00	0.00E+00	-0.42731	2.52E-189	4.60E-185
**ADAMTS6**	0.358049185	1.80E-102	3.29E-98	0.234479	3.79E-54	6.92E-50
**ADIRF**	-1.159908787	0.00E+00	0.00E+00	-1.13737	0	0
**ADM**	0.634712081	1.49E-124	2.72E-120	0.278125	1.22E-19	2.22E-15
**ADSS**	0.244699737	2.31E-40	4.22E-36	0.429081	6.01E-139	1.10E-134
**AEBP1**	0.389886874	1.90E-83	3.47E-79	0.190889	5.25E-13	9.57E-09
**AFF3**	-0.287261958	9.58E-133	1.75E-128	-0.28413	1.98E-196	3.62E-192
**AHNAK**	-0.209314149	3.79E-43	6.91E-39	-0.35713	1.54E-144	2.81E-140
**AHNAK2**	-0.266978893	2.72E-62	4.96E-58	-0.28105	4.24E-94	7.74E-90
**AIMP1**	0.064144662	7.18E-03	1.00E+00	0.293913	2.97E-71	5.41E-67
**AK2**	0.454154678	1.39E-112	2.53E-108	0.46028	3.68E-148	6.72E-144
**AKAP12**	0	NA	NA	0.290599	7.05E-122	1.29E-117
**ANGPTL4**	0.290228624	2.20E-50	4.01E-46	0.166131	1.88E-22	3.42E-18
**ANGPTL5**	-0.179909676	2.44E-38	4.45E-34	-0.25481	1.05E-118	1.91E-114
**ANXA5**	0.039381562	1.22E-01	1.00E+00	0.2854	1.58E-92	2.89E-88
**AP2M1**	-0.313852914	5.77E-93	1.05E-88	-0.39382	2.66E-180	4.85E-176
**APOD**	-0.842829333	1.41E-222	2.57E-218	-0.70612	1.10E-231	2.00E-227
**ARF4**	0.305211542	8.63E-66	1.57E-61	0.26348	3.97E-66	7.25E-62
**ARF6**	0.55047121	2.24E-176	4.09E-172	0.525656	4.75E-251	8.67E-247
**ARHGAP29**	-0.462284385	6.40E-142	1.17E-137	-4.77E-01	1.51E-209	2.76E-205
**ARL2BP**	-0.30685546	8.76E-89	1.60E-84	-0.33779	3.71E-126	6.78E-122
**ARL6IP4**	-0.211094794	7.14E-62	1.30E-57	-0.2694	1.17E-126	2.13E-122
**ARL6IP5**	-0.260517764	1.97E-66	3.59E-62	-0.37882	1.65E-168	3.00E-164
**ARPC1A**	-0.270898981	9.64E-79	1.76E-74	-0.30391	4.51E-127	8.24E-123
**ARPC1B**	-0.305487112	1.54E-50	2.80E-46	-0.22583	2.90E-41	5.29E-37
**ARPP19**	0.36008382	1.09E-100	2.00E-96	0.456979	8.80E-179	1.61E-174
**ARRDC3**	0.514501097	8.80E-90	1.61E-85	0.201655	1.77E-24	3.22E-20
**ASPN**	-0.137186971	2.29E-18	4.17E-14	-0.39804	2.47E-99	4.51E-95
**ATF4**	0.059800302	3.52E-05	6.43E-01	0.255941	7.75E-79	1.41E-74

**ATP5A1**	-0.304315905	3.39E-90	6.18E-86	-0.2845	2.08E-100	3.80E-96
**ATP5D**	-0.277876275	3.78E-99	6.90E-95	-0.24584	7.47E-94	1.36E-89
**ATP5G2**	-0.337399853	2.74E-159	4.99E-155	-0.11939	1.43E-24	2.61E-20
**ATP5L**	-0.286929692	5.83E-119	1.06E-114	-0.13226	1.03E-37	1.88E-33
**ATP5O**	-0.354268263	1.71E-140	3.12E-136	-0.20492	4.65E-62	8.48E-58
**ATP6V0B**	0.158154502	4.44E-20	8.11E-16	0.250298	2.31E-60	4.21E-56
**ATP6V0E1**	0.253021236	1.43E-69	2.60E-65	2.02E-01	6.61E-56	1.21E-51
**ATP6V1G1**	0.356440025	2.06E-143	3.75E-139	4.37E-01	1.73E-191	3.15E-187
**ATPIF1**	-0.172431368	2.86E-36	5.22E-32	-0.38303	4.18E-179	7.63E-175
**ATRAID**	-0.060894672	1.84E-05	3.37E-01	-0.43285	4.95E-200	9.03E-196
**ATRNL1**	-0.445437016	1.42E-206	2.59E-202	-0.42987	5.22E-260	9.53E-256
**B2M**	-0.677733435	0.00E+00	0.00E+00	-0.86962	0	0
**BAALC**	0.386405941	5.07E-95	9.25E-91	0.536672	3.75E-131	6.84E-127
**BAMBI**	0.001226699	1.95E-02	1.00E+00	-0.44419	7.16E-116	1.3E-111
**BEX2**	-0.276439393	2.55E-83	4.66E-79	-0.28003	4.03E-98	7.36E-94
**BEX4**	-0.147808338	7.64E-28	1.39E-23	-0.30838	1.63E-117	2.98E-113
**BGN**	-0.262315957	2.37E-93	4.32E-89	-0.28996	8.45E-127	1.54E-122
**BHLHE40**	0.545672004	2.06E-106	3.76E-102	0.70973	1.11E-226	2.03E-222
**BHLHE41**	-0.085668066	8.65E-10	1.58E-05	-0.36344	1.46E-162	2.67E-158
**BMP2**	0.804177334	1.47E-268	2.68E-264	0.828155	0	0
**BNIP3**	0.261735351	1.63E-53	2.98E-49	0.199448	1.39E-48	2.53E-44
**BRK1**	-0.277301277	3.90E-110	7.11E-106	-0.42734	1.86E-264	3.39E-260
**BSG**	-0.250477711	9.83E-73	1.79E-68	-0.38986	5.37E-177	9.80E-173
**BTF3**	-0.395372955	1.29E-277	2.35E-273	-0.33176	1.98E-246	3.61E-242
**BTG1**	0.537765777	3.57E-202	6.51E-198	0.343597	8.21E-116	1.50E-111
**BTG3**	0.190606173	4.93E-33	9.00E-29	0.324586	8.45E-117	1.54E-112
**BZW1**	0.550532917	1.33E-185	2.43E-181	0.750769	0	0
**C10orf10**	0.302770071	1.28E-34	2.34E-30	-0.09743	5.718E-07	0.010434
**C11orf31**	0.281188806	9.14E-80	1.67E-75	0.309476	3.07E-114	5.60E-110
**C11orf96**	0.37649005	1.96E-99	3.58E-95	0.915627	0	0
**C12orf57**	-0.254024498	3.05E-99	5.56E-95	-0.23663	7.86E-101	1.43E-96
**C14orf1**	-0.259998899	1.58E-76	2.89E-72	-0.27384	7.85E-112	1.43E-107
**C15orf48**	0.272177486	6.89E-47	1.26E-42	0.565112	2.99E-153	5.45E-149
**C19orf43**	-0.279473316	1.50E-98	2.74E-94	-0.23828	2.28E-87	4.17E-83
**C1orf21**	-0.16354735	1.69E-31	3.08E-27	-0.2972	7.55E-109	1.38E-104
**C1S**	0.0050643	3.64E-01	1.00E+00	-0.27177	4.952E-83	9.04E-79
**C2orf40**	0.209268514	1.59E-06	2.90E-02	-0.80392	0	0
**C2orf49**	0.138206019	3.26E-27	5.96E-23	0.25093	3.56E-92	6.49E-88
**C2orf82**	-0.637108273	3.32E-218	6.06E-214	-1.22501	0	0

**C4orf48**	0.333726798	6.31E-102	1.15E-97	0.181019	1.49E-46	2.72E-42
**C6orf48**	-0.253295849	1.58E-48	2.89E-44	-0.16477	2.04E-25	3.72E-21
**C7orf73**	0.021609485	4.74E-06	8.64E-02	-0.27222	5.41E-126	9.88E-122
**CA9**	0.573847564	3.30E-167	6.03E-163	0.267099	1.76E-66	3.22E-62
**CALM3**	-0.215594521	6.60E-47	1.20E-42	-0.26422	1.48E-79	2.71E-75
**CALR**	-0.183506038	9.31E-17	1.70E-12	-0.39138	1.14E-119	2.09E-115
**CANX**	-0.095414107	1.31E-11	2.39E-07	-0.28461	1.65E-98	3.01E-94
**CAPS**	-1.010981402	0.00E+00	0	-1.32832	0	0
**CAPZA2**	0.257756899	1.26E-58	2.29E-54	0.283186	5.81E-82	1.06E-77
**CCDC102B**	-0.552779497	0.00E+00	0.00E+00	-0.43519	0	0
**CCDC8**	-0.273445129	8.49E-112	1.55E-107	-0.23551	1.17E-134	2.14E-130
**CCDC80**	0.33012088	1.06E-29	1.94E-25	-0.46874	3.66E-170	6.67E-166
**CCDC85B**	0.90509136	1.33E-280	2.42E-276	0.684452	8.54E-255	1.56E-250
**CCDC88A**	-0.157652237	2.56E-30	4.66E-26	-0.27069	7.20E-121	1.31E-116
**CCL20**	0	NA	NA	0.505346	3.67E-83	6.70E-79
**CCND1**	0.264639775	4.74E-84	8.65E-80	0.549957	3.07E-258	5.61E-254
**CCNG1**	-0.220716717	1.84E-52	3.36E-48	-0.39061	1.40E-187	2.55E-183
**CCNG2**	0.269956889	3.20E-44	5.84E-40	0.087943	1.53E-05	0.279731
**CCNI**	-0.62124171	0.00E+00	0.00E+00	-0.5978	0	0
**CCT8**	0.110936367	2.22E-09	4.04E-05	0.292136	4.68E-76	8.53E-72
**CD320**	-0.049616363	2.47E-04	1	-0.30361	5.78E-114	1.05E-109
**CD44**	0.432968968	1.70E-154	3.11E-150	0.4654	4.22E-233	7.70E-229
**CD55**	0.34000849	6.76E-79	1.23E-74	0.993607	0	0
**CD63**	-0.190533957	1.61E-47	2.94E-43	-0.2634	4.21E-94	7.68E-90
**CD9**	-0.576632467	1.62E-209	2.96E-205	-0.61648	2.18E-283	3.98E-279
**CD99**	-0.321060618	1.07E-141	1.96E-137	-0.72987	0	0
**CDC37L1**	0.220069752	2.04E-49	3.71E-45	0.280352	5.84E-80	1.07E-75
**CDK5RAP2**	0.312299946	7.72E-37	1.41E-32	0.092342	7.87E-05	1
**CEBPD**	-0.312331417	4.62E-49	8.44E-45	-0.6641	3.07E-250	5.60E-246
**CFD**	0.468305237	2.31E-113	4.22E-109	0	NA	NA
**CFH**	-0.28708416	4.10E-40	7.48E-36	-0.3546	3.00E-64	5.47E-60
**CHD2**	0.139974956	1.31E-14	2.39E-10	0.250606	1.48E-38	2.71E-34
**CHI3L1**	0.416970778	5.73E-58	1.04E-53	-0.49678	1.63E-82	2.97E-78
**CHI3L2**	1.242716698	0	0	0.518394	1.35E-81	2.46E-77
**CHID1**	-0.2771385	1.31E-73	2.39E-69	-0.42356	1.26E-198	2.30E-194
**CHMP4B**	0.152752216	1.11E-19	2.02E-15	0.27108	5.39E-74	9.84E-70
**CHPT1**	-0.178986308	1.04E-34	1.90E-30	-0.25872	6.08E-76	1.11E-71
**CHRDL2**	-0.478539659	1.80E-106	3.28E-102	-0.42287	7.28E-146	1.33E-141
**CITED2**	-0.61830128	1.28E-194	2.33E-190	-0.74605	0	0

**CKAP4**	-0.269859021	1.37E-65	2.51E-61	-0.26501	6.54E-92	1.19E-87
**CLDND1**	0.304492273	4.29E-99	7.84E-95	0.311427	3.73E-113	6.81E-109
**CLEC2B**	0.238602568	3.98E-26	7.27E-22	0.406686	2.77E-70	5.06E-66
**CLEC3A**	-1.101842308	0	0	-1.80123	0	0
**CLK1**	0.361879422	5.03E-103	9.18E-99	0.381247	9.97E-129	1.82E-124
**CMB9-22P13.1**	-0.22372045	5.47E-47	9.98E-43	-0.33944	2.66E-134	4.85E-130
**CMPK1**	-0.074179299	5.68E-09	0.000104	-0.27267	5.39E-103	9.84E-99
**CNIH1**	0.191873189	4.39E-38	8.01E-34	0.295286	6.35E-95	1.16E-90
**COL11A1**	-0.078043876	7.36E-25	1.34E-20	-0.63755	0	0
**COL11A2**	-0.062861466	4.47E-07	0.008156	-0.49439	6.24E-205	1.14E-200
**COL1A1**	0	NA	NA	0.550273	4.91E-38	8.96E-34
**COL2A1**	-0.812915384	9.33E-187	1.70E-182	-1.36708	0	0
**COL5A2**	-0.274405993	2.59E-33	4.73E-29	-0.32683	7.34E-92	1.34E-87
**COL9A2**	-0.065094935	1.38E-09	2.51E-05	-0.38549	4.85E-154	8.84E-150
**COL9A3**	-0.339422645	4.13E-73	7.54E-69	-0.9621	0	0
**COMMD6**	-0.311252765	1.55E-189	2.83E-185	-0.31465	9.32E-232	1.70E-227
**COMP**	1.198002554	0	0	1.23287	0	0
**COX17**	0.346679387	4.24E-111	7.74E-107	0.41746	6.41E-177	1.17E-172
**COX7A2**	0.142240428	8.74E-40	1.60E-35	0.325129	2.35E-202	4.29E-198
**COX7C**	-0.266719328	4.12E-152	7.52E-148	-0.14659	2.35E-64	4.28E-60
**CP**	-0.370041985	5.01E-138	9.14E-134	-0.76308	0	0
**CPE**	-0.30556084	3.50E-80	6.39E-76	-0.32012	4.56E-96	8.32E-92
**CREG1**	-0.205893105	1.25E-40	2.27E-36	-0.34075	3.61E-136	6.59E-132
**CRIP2**	-0.484322769	1.12E-166	2.04E-162	-0.40384	3.84E-160	7.01E-156
**CRISPLD1**	-0.905225098	9.46E-275	1.73E-270	-0.79239	0	0
**CRLF1**	0.282360545	1.76E-86	3.21E-82	0.373145	1.82E-140	3.32E-136
**CRTAC1**	0.364271752	6.34E-119	1.16E-114	0.3038	2.46E-106	4.49E-102
**CRTAP**	-0.737970474	0	0	-0.67345	0	0
**CRYAB**	-0.353058867	1.22E-47	2.23E-43	-0.33924	1.32E-75	2.41E-71
**CSGALNACT2**	0.280733673	2.22E-66	4.04E-62	0.450878	1.07E-208	1.95E-204
**CST3**	-0.223397466	7.27E-54	1.33E-49	-0.71614	0	0
**CTGF**	-0.866074275	7.15E-134	1.30E-129	-0.7487	5.81E-104	1.06E-99
**CTHRC1**	0.843182592	0	0	0.7076	4.49E-247	8.19E-243
**CTNNB1**	0.386522095	5.54E-48	1.01E-43	0.515906	2.11E-89	3.85E-85
**CTSC**	-0.321659155	3.81E-39	6.96E-35	-0.50056	1.78E-143	3.25E-139
**CTSD**	0.798865369	3.20E-129	5.84E-125	-0.42878	1.37E-195	2.50E-191
**CTSK**	-0.213986806	1.64E-58	2.99E-54	-0.32788	4.41E-186	8.05E-182
**CTSL**	0.757536233	7.88E-102	1.44E-97	1.251541	0	0

**CUTA**	-0.2567633	6.16E-74	1.12E-69	-0.34505	3.99E-155	7.28E-151
**CXCL8**	0	NA	NA	0.889855	1.03E-151	1.89E-147
**CXXC5**	-0.363800126	4.75E-110	8.66E-106	-0.50595	9.91E-284	1.81E-279
**CYBRD1**	-0.308054	9.48E-95	1.73E-90	-0.34063	6.85E-140	1.25E-135
**CYP1B1**	0.378808849	1.31E-21	2.39E-17	0.078351	0.412547	1
**CYR61**	-0.617464094	8.29E-123	1.51E-118	-0.61439	5.76E-185	1.05E-180
**CYSTM1**	-0.279761208	4.34E-73	7.91E-69	-0.11654	1.28E-18	2.33E-14
**CYTL1**	0.349312952	2.27E-61	4.14E-57	0.057582	2.64E-06	0.048214
**DBP**	-0.302270841	6.84E-86	1.25E-81	-0.34735	4.65E-176	8.48E-172
**DCN**	-0.227907345	4.10E-63	7.49E-59	-0.75614	0	0
**DDIT3**	0.483737221	2.19E-114	4.00E-110	0.435109	1.15E-123	2.10E-119
**DDIT4**	0.13068525	7.77E-17	1.42E-12	-0.31586	4.79E-65	8.75E-61
**DDIT4L**	0.794520392	2.05E-266	3.73E-262	0.840644	3.44E-282	6.27E-278
**DDOST**	-0.153807269	9.44E-22	1.72E-17	-0.26382	8.64E-81	1.58E-76
**DDR2**	-0.287904719	2.61E-64	4.76E-60	-0.53247	9.04E-283	1.65E-278
**DMKN**	-0.303592155	1.32E-100	2.40E-96	-0.33869	1.37E-154	2.50E-150
**DNAJB1**	0.150315439	7.67E-09	0.00014	0.380069	1.50E-60	2.73E-56
**DNAJB9**	0.404172612	5.68E-88	1.04E-83	0.488258	6.00E-145	1.10E-140
**DNER**	0.342563303	3.47E-70	6.33E-66	0.494635	8.02E-176	1.46E-171
**DNM3OS**	-0.297344125	2.94E-78	5.37E-74	-0.49211	2.74E-234	5.00E-230
**DSEL**	-0.302288723	8.07E-79	1.47E-74	-0.22789	1.40E-92	2.56E-88
**DUSP1**	0.004863469	0.004482	1	-0.3847	3.12E-124	5.69E-120
**EBPL**	-0.208066468	1.22E-51	2.22E-47	-0.28165	1.22E-120	2.22E-116
**EDF1**	-0.273561081	1.33E-127	2.44E-123	-0.18484	1.61E-78	2.94E-74
**EEF1A1**	-0.665422984	0	0	-0.86024	0	0
**EEF1B2**	-0.503276276	0	0	-0.437	0	0
**EEF2**	-0.631231384	0	0	-0.81748	0	0
**EFNA1**	-0.163478827	1.60E-32	2.92E-28	-0.32363	4.61E-133	8.42E-129
**EGLN3**	0.483817658	7.66E-139	1.40E-134	0.1368	1.97E-16	3.60E-12
**EGR1**	0.383555074	7.02E-54	1.28E-49	-0.01361	0.0123146	1
**EID1**	-0.336486583	2.04E-166	3.72E-162	-0.58167	0	0
**EIF1**	-0.023088268	0.047079	1	0.488611	0	0
**EIF1B**	0.198592273	2.20E-36	4.02E-32	0.387931	1.40E-154	2.55E-150
**EIF2A**	-0.360150702	6.31E-113	1.15E-108	-0.28251	1.92E-79	3.50E-75
**EIF2S3**	-0.252045148	5.22E-59	9.52E-55	-0.28277	6.09E-75	1.11E-70
**EIF3D**	-0.32443765	6.96E-96	1.27E-91	-0.27212	5.63E-83	1.03E-78
**EIF3E**	-0.242266542	1.26E-81	2.31E-77	-0.26691	3.28E-98	5.99E-94
**EIF3F**	-0.291056917	2.18E-98	3.99E-94	-0.17517	1.00E-44	1.83E-40
**EIF3H**	-0.831105398	0	0	-0.7591	0	0

**EIF3K**	-0.351774765	1.96E-145	3.58E-141	-0.2129	2.32E-77	4.24E-73
**EIF3L**	-0.540784852	1.26E-306	2.30E-302	-0.59001	0	0
**EIF3M**	-0.252648717	7.12E-65	1.30E-60	-0.09439	5.68E-13	1.04E-08
**EIF4A2**	0.270111092	2.79E-69	5.08E-65	0.427465	7.98E-207	1.46E-202
**EIF4B**	-0.530532656	2.35E-229	4.28E-225	-0.48024	9.40E-238	1.72E-233
**EIF4EBP1**	0.24911843	1.34E-35	2.45E-31	0.371279	9.68E-89	1.77E-84
**EIF5**	0.046800612	0.008275	1	0.292287	4.33E-90	7.90E-86
**EMP3**	0.339230826	5.37E-97	9.80E-93	0.399299	8.28E-147	1.51E-142
**ENAH**	-0.174032587	1.91E-38	3.49E-34	-0.38046	3.64E-205	6.64E-201
**ENO1**	0.260547029	1.05E-24	1.91E-20	0.348543	3.41E-84	6.23E-80
**ENO2**	0.559899514	1.41E-181	2.57E-177	0.52463	4.74E-248	8.64E-244
**ENPP1**	-0.276526369	2.44E-92	4.46E-88	-0.13934	3.11E-128	5.67E-124
**EPS8**	0.302613612	3.03E-83	5.53E-79	0.389791	8.03E-176	1.47E-171
**EPYC**	0.805758755	1.78E-174	3.25E-170	-0.05459	7.04E-09	0.000128
**ERGIC3**	-0.346893494	2.14E-128	3.90E-124	-0.41541	3.65E-211	6.66E-207
**ERO1A**	0.717084589	1.07E-230	1.95E-226	0.794649	0	0
**ERP29**	-0.206806241	5.32E-47	9.70E-43	-0.25923	4.38E-88	8.00E-84
**ERRFI1**	0.388005596	1.09E-72	1.99E-68	0.318599	1.66E-59	3.03E-55
**ESD**	0.060474646	0.584716	1	0.256515	2.38E-38	4.35E-34
**EVI2A**	0.306739564	1.23E-124	2.25E-120	0.363436	1.10E-196	2.01E-192
**FABP5**	0.514626255	6.73E-17	1.23E-12	0.396332	9.24E-26	1.69E-21
**FAM162A**	0.493578667	1.32E-185	2.41E-181	0.350376	5.58E-124	1.02E-119
**FAM46A**	-0.109896446	1.47E-05	0.26827	-0.44166	1.33E-125	2.43E-121
**FAP**	0.165360643	1.72E-35	3.14E-31	0.423061	8.90E-211	1.62E-206
**FBXO2**	-0.412630374	6.62E-144	1.21E-139	-0.81174	0	0
**FCGRT**	-0.240092081	6.75E-55	1.23E-50	-0.44205	1.02E-199	1.86E-195
**FGFBP2**	0.065406456	1.25E-06	0.022786	-0.37073	2.00E-61	3.65E-57
**FHL1**	-0.550626366	1.39E-149	2.54E-145	-0.50738	2.55E-187	4.65E-183
**FHL2**	0.013595601	0.021966	1	0.281161	1.67E-99	3.05E-95
**FIS1**	-0.165946965	1.40E-30	2.56E-26	-0.27279	8.93E-97	1.63E-92
**FKBP10**	-0.148985161	1.95E-20	3.55E-16	-0.30353	4.69E-93	8.56E-89
**FMOD**	-0.262846734	2.45E-80	4.47E-76	-0.64325	0	0
**FN1**	0.701163854	0	0	0.864219	0	0
**FNIP2**	0.045987076	7.24E-05	1	0.343229	2.22E-112	4.05E-108
**FOS**	-0.039445246	3.81E-09	6.95E-05	-0.58727	1.35E-274	2.47E-270
**FOSL1**	0.679148411	1.08E-205	1.98E-201	0.447072	4.21E-208	7.68E-204
**FRZB**	-1.838581404	0	0	-1.73719	0	0
**FTH1**	-0.700432726	0	0	-0.64642	0	0
**FTL**	-0.259786279	4.53E-142	8.26E-138	-0.52324	0	0

**FXYD1**	0.020384998	3.86E-05	0.705024	-0.29932	6.93E-74	1.27E-69
**FXYD6**	-0.259850564	8.03E-90	1.47E-85	-0.66714	0	0
**G0S2**	0.912798042	7.39E-55	1.35E-50	0.650508	3.29E-30	6.00E-26
**GADD45B**	-0.232659873	1.25E-32	2.29E-28	-0.27765	1.34E-79	2.45E-75
**GADD45GIP1**	0.103133193	1.41E-11	2.58E-07	0.284097	3.56E-101	6.49E-97
**GAPDH**	0.372905986	3.47E-64	6.33E-60	0.056261	1.61E-06	0.029302
**GAS6**	-0.43085833	4.12E-143	7.52E-139	-0.50619	3.06E-274	5.59E-270
**GBE1**	0.423336832	5.79E-83	1.06E-78	0.478605	5.33E-139	9.73E-135
**GDF15**	0.182630224	2.34E-31	4.27E-27	0.275085	2.22E-72	4.04E-68
**GJA1**	0.151401637	7.56E-40	1.38E-35	0.350957	5.51E-144	1.01E-139
**GLTSCR2**	-0.300356046	7.23E-106	1.32E-101	-0.29294	4.65E-118	8.48E-114
**GNAS**	-0.414733817	1.08E-161	1.96E-157	-0.39978	4.37E-188	7.98E-184
**GOLIM4**	-0.079304106	3.70E-17	6.75E-13	-0.37367	6.36E-99	1.16E-94
**GOLT1B**	0.295756127	4.97E-77	9.06E-73	0.437263	2.62E-175	4.78E-171
**GPAA1**	-0.172034437	9.80E-33	1.79E-28	-0.25191	2.42E-100	4.42E-96
**GPRC5A**	0.256538167	1.08E-81	1.97E-77	0.323618	2.18E-156	3.98E-152
**GPX1**	0.305472938	2.09E-79	3.81E-75	0.369747	3.28E-138	5.99E-134
**GPX3**	-0.552780703	5.68E-225	1.04E-220	-0.81213	0	0
**GRB10**	0.242653477	5.66E-89	1.03E-84	0.379731	2.56E-232	4.68E-228
**GREM1**	-0.52166636	1.43E-103	2.61E-99	-0.446	1.33E-157	2.44E-153
**GRN**	-0.126044847	4.41E-34	8.04E-30	-0.3564	2.47E-185	4.51E-181
**GSN**	-0.163429737	1.64E-24	2.99E-20	-0.45228	1.10E-190	2.01E-186
**GSTA4**	-0.142989052	9.09E-31	1.66E-26	-0.28407	1.90E-133	3.47E-129
**GSTO1**	0.135302748	1.69E-11	3.08E-07	0.601756	4.20E-290	7.67E-286
**GSTP1**	-0.300744354	3.12E-120	5.69E-116	-0.27934	1.36E-111	2.48E-107
**H1FX**	-0.22970855	5.18E-61	9.45E-57	-0.28598	2.48E-110	4.52E-106
**H3F3A**	-0.475573049	1.80E-289	3.29E-285	-0.50736	0	0
**HADHA**	-0.238829042	1.37E-61	2.50E-57	-0.25901	1.50E-100	2.73E-96
**HDLBP**	-0.416852472	3.47E-139	6.33E-135	-0.39985	9.52E-175	1.74E-170
**HERPUD1**	-0.307291011	8.63E-79	1.57E-74	-0.29942	1.28E-103	2.34E-99
**HEXB**	-0.193757668	4.74E-31	8.66E-27	-0.3337	1.24E-115	2.26E-111
**HIF1A**	0.251163061	2.92E-39	5.32E-35	-0.0147	2.13E-05	0.389002
**HILPDA**	0.620157536	1.07E-161	1.95E-157	0.325549	4.78E-40	8.72E-36
**HINT1**	-0.460551505	5.37E-308	9.79E-304	-0.2434	1.51E-134	2.75E-130
**HIST1H1C**	-0.608653279	8.25E-140	1.51E-135	-0.33284	3.81E-60	6.95E-56
**HIST1H4C**	-0.379684761	3.55E-129	6.49E-125	-0.13015	7.89E-155	1.44E-150
**HLA-A**	-0.421367417	6.84E-140	1.25E-135	-0.4498	4.85E-175	8.84E-171
**HLA-B**	-0.433264205	2.56E-143	4.67E-139	-0.32255	1.75E-85	3.20E-81
**HLA-C**	-0.473123052	9.16E-176	1.67E-171	-0.46337	1.09E-179	1.99E-175

**HLA-DRB1**	0.463897887	3.15E-143	5.75E-139	0.500159	1.56E-239	2.85E-235
**HMGA1**	0.388423289	1.61E-53	2.94E-49	1.129443	0	0
**HMGA2**	0.280445332	1.12E-149	2.04E-145	0.441018	0	0
**HMGN2**	-0.253702472	2.43E-89	4.43E-85	-0.37878	1.69E-243	3.09E-239
**HMGN3**	-0.132278861	1.30E-25	2.38E-21	-0.28122	1.21E-136	2.21E-132
**HNRNPA1**	-0.452287252	2.26E-253	4.13E-249	-0.5943	0	0
**HOXB6**	-0.256334854	6.78E-69	1.24E-64	-0.21712	3.76E-60	6.86E-56
**HOXB8**	-0.238580197	2.63E-99	4.80E-95	-0.27475	6.17E-186	1.13E-181
**HOXC10**	-0.380329177	1.46E-123	2.66E-119	-0.24942	8.71E-64	1.59E-59
**HSP90AB1**	-0.316062883	9.46E-95	1.73E-90	-0.32803	3.66E-147	6.68E-143
**HSP90B1**	-0.114985285	1.27E-06	0.023208	-0.34244	8.13E-93	1.48E-88
**HSPA8**	-0.221835645	4.70E-41	8.57E-37	-0.2662	2.32E-65	4.24E-61
**HSPB1**	-0.301633343	4.75E-74	8.66E-70	-0.36405	2.74E-134	5.01E-130
**HTRA1**	-0.083361814	2.55E-14	4.65E-10	-0.45086	7.02E-253	1.28E-248
**ID1**	-0.428335671	1.99E-55	3.62E-51	-0.95704	0	0
**ID2**	-0.340493964	1.68E-40	3.07E-36	-0.76025	9.76E-292	1.78E-287
**ID3**	-0.324858919	4.90E-90	8.93E-86	-1.009	0	0
**ID4**	-0.283779068	1.47E-57	2.69E-53	-0.64784	5.08E-289	9.27E-285
**IER2**	-0.041602381	1.48E-11	2.71E-07	-0.30538	8.18E-92	1.49E-87
**IER3**	0.261771782	1.12E-39	2.05E-35	0.536152	6.13E-139	1.12E-134
**IFI27L2**	-0.227645139	5.58E-64	1.02E-59	-0.26469	2.75E-98	5.02E-94
**IFITM2**	-0.091965467	5.23E-09	9.55E-05	-0.26793	2.07E-77	3.77E-73
**IFRD1**	0.384678343	1.01E-95	1.84E-91	0.345254	6.79E-83	1.24E-78
**IGBP1**	-0.259591499	1.00E-58	1.82E-54	-0.15853	2.04E-26	3.72E-22
**IGFBP3**	0.726318985	7.53E-184	1.37E-179	1.185738	0	0
**IGFBP5**	0.527778205	7.46E-115	1.36E-110	0.282887	1.39E-21	2.54E-17
**IGFBP6**	-0.268792833	1.63E-44	2.97E-40	-0.74637	1.01E-237	1.85E-233
**IGFBP7**	-0.239182436	6.63E-29	1.21E-24	-0.45524	3.46E-155	6.31E-151
**IL11**	0.374415286	5.45E-104	9.94E-100	0.64615	1.18E-171	2.15E-167
**ILF3-AS1**	-0.255671861	3.68E-77	6.71E-73	-0.3325	6.09E-158	1.11E-153
**INHBA**	-0.344140632	1.05E-90	1.91E-86	-0.47236	2.95E-219	5.38E-215
**INSIG1**	-0.318837875	5.94E-39	1.08E-34	-0.26384	1.83E-40	3.34E-36
**INSIG2**	0.365054518	4.90E-66	8.95E-62	0.116902	3.62E-06	0.066035
**ISG20**	0.43537636	2.79E-110	5.09E-106	0.575614	1.33E-239	2.43E-235
**ISLR**	-0.643902092	4.44E-246	8.11E-242	-0.81498	0	0
**ISM1**	-0.24300764	4.75E-84	8.67E-80	-0.25457	1.61E-155	2.93E-151
**ITM2A**	-0.404370438	1.12E-108	2.03E-104	-0.57602	1.23E-254	2.24E-250
**ITM2B**	0.076385364	0.211905	1	-0.32983	2.73E-123	4.98E-119
**ITM2C**	-0.306435229	1.59E-67	2.89E-63	-0.71269	0	0

**JUNB**	0.039095944	1.43E-06	0.026066	-0.55778	6.09E-224	1.11E-219
**KDELR2**	-0.279442088	3.67E-76	6.69E-72	-0.32364	3.57E-140	6.52E-136
**KDELR3**	-0.193870739	2.63E-50	4.80E-46	-0.29861	7.66E-130	1.40E-125
**KDM7A**	0.165116696	3.52E-53	6.42E-49	0.253656	1.09E-126	1.98E-122
**KHDRBS3**	-0.30551581	2.35E-161	4.29E-157	-0.21711	9.90E-116	1.81E-111
**KLF3**	0.23603232	7.49E-33	1.37E-28	0.379022	1.86E-90	3.40E-86
**KMT2E-AS1**	0.135697875	2.92E-41	5.33E-37	0.265346	3.07E-87	5.61E-83
**KRT8**	-0.264177346	4.26E-67	7.77E-63	-0.21267	1.75E-59	3.19E-55
**LDHB**	-0.34888117	5.70E-128	1.04E-123	-0.34823	3.75E-151	6.84E-147
**LECT1**	-0.336815455	1.46E-64	2.66E-60	-1.01953	0	0
**LGALS1**	-0.335409178	2.88E-53	5.26E-49	0.120207	0.9493502	1
**LINC00152**	0.05949318	0.030501	1	0.414252	2.55E-83	4.66E-79
**LINC00493**	0.082543339	3.72E-08	0.000679	0.289634	9.95E-105	1.82E-100
**LINC01503**	-0.324963244	6.06E-54	1.11E-49	-0.34435	5.78E-103	1.05E-98
**LITAF**	-0.285594293	9.93E-83	1.81E-78	-0.34747	3.79E-145	6.92E-141
**LRPAP1**	-0.070674363	2.39E-07	0.004361	-0.35162	8.91E-126	1.63E-121
**LRRC2**	-0.306582053	3.24E-112	5.92E-108	-0.28383	6.22E-147	1.13E-142
**LTBP1**	0.272063035	1.78E-138	3.26E-134	0.334075	9.42E-203	1.72E-198
**LUC7L3**	-0.17876329	4.82E-37	8.79E-33	-0.266	5.75E-102	1.05E-97
**MAGED1**	-0.453063413	1.15E-79	2.10E-75	-0.48345	4.98E-158	9.09E-154
**MALAT1**	-0.345618927	1.67E-109	3.05E-105	-0.61874	0	0
**MDFI**	-0.212544412	1.29E-43	2.35E-39	-0.51144	8.91E-249	1.63E-244
**MED21**	0.301386268	4.01E-78	7.32E-74	0.370046	5.14E-108	9.38E-104
**MEG3**	-0.279160677	4.57E-41	8.34E-37	-0.32886	1.51E-83	2.76E-79
**METAP2**	0.236448491	1.10E-54	2.01E-50	0.291276	2.25E-98	4.10E-94
**MFGE8**	-0.176097089	9.60E-24	1.75E-19	-0.41084	6.72E-137	1.23E-132
**MGP**	-0.431110363	1.55E-115	2.82E-111	-1.02934	0	0
**MGST1**	0.262566951	7.04E-60	1.28E-55	0.242936	7.31E-44	1.33E-39
**MIA**	-0.291775154	5.43E-54	9.91E-50	-0.5882	2.49E-248	4.54E-244
**MIF**	0.297741047	1.53E-154	2.80E-150	0.731694	0	0
**MIR99AHG**	-0.187881633	7.93E-62	1.45E-57	-0.25621	6.37E-158	1.16E-153
**MKX**	-0.217517028	4.60E-54	8.40E-50	-0.32263	2.65E-166	4.84E-162
**MMP3**	0.392312277	5.71E-63	1.04E-58	0.679387	3.41E-109	6.23E-105
**MORF4L2**	-0.12978875	2.14E-20	3.91E-16	-0.34713	3.09E-133	5.64E-129
**MOXD1**	-0.395904977	1.71E-138	3.12E-134	-0.37415	2.89E-155	5.27E-151
**MPP6**	-0.291827045	5.39E-116	9.83E-112	-0.26567	7.33E-118	1.34E-113
**MRPS6**	0.772335405	1.83E-126	3.34E-122	0.79218	3.19E-213	5.82E-209
**MSANTD3**	0.195122215	3.29E-54	6.00E-50	0.291504	1.11E-115	2.03E-111
**MSMO1**	-0.226384949	8.53E-27	1.56E-22	-0.25512	5.81E-55	1.06E-50

**MSMP**	-0.722180457	1.56E-73	2.84E-69	-0.79703	1.67E-82	3.05E-78
**MT-ATP6**	-0.839727465	3.61E-278	6.59E-274	-0.87972	0	0
**MT-CO1**	-0.856561051	1.04E-275	1.90E-271	-0.73119	1.61E-273	2.93E-269
**MT-CO2**	-0.738865014	1.15E-238	2.09E-234	-0.75754	0	0
**MT-CO3**	-0.820046739	3.31E-260	6.05E-256	-0.73769	4.47E-303	8.15E-299
**MT-CYB**	-0.991686987	0	0	-0.89712	0	0
**MT-ND1**	-0.758908781	1.46E-255	2.67E-251	-0.78626	0	0
**MT-ND2**	-0.952916953	7.72E-303	1.41E-298	-0.93057	0	0
**MT-ND3**	-1.040498693	0	0	-0.84782	0	0
**MT-ND4**	-0.594189094	1.27E-189	2.31E-185	-0.49082	8.63E-171	1.57E-166
**MT-ND5**	-0.32805583	3.32E-57	6.05E-53	-0.37088	2.43E-99	4.43E-95
**MT1E**	0.175536194	1.46E-07	0.002663	0.430335	2.31E-28	4.22E-24
**MT1F**	0.388752241	2.64E-47	4.81E-43	-0.0527	0.0027746	1
**MT1G**	0.870051473	4.56E-149	8.32E-145	0.636863	2.91E-78	5.31E-74
**MT1H**	0.640489767	3.26E-68	5.94E-64	0.522455	3.31E-43	6.05E-39
**MT1M**	0.264765417	5.32E-26	9.71E-22	0.405756	1.24E-54	2.26E-50
**MT1X**	0.94011814	6.13E-221	1.12E-216	0.97663	5.90E-238	1.08E-233
**MT2A**	0.979258309	3.70E-217	6.75E-213	1.856842	0	0
**MTDH**	-0.28366825	3.82E-79	6.96E-75	-0.2803	1.23E-87	2.24E-83
**MYL12B**	-0.299047751	3.55E-85	6.48E-81	-0.19951	6.60E-50	1.20E-45
**MYL6**	-0.416169768	1.36E-184	2.48E-180	-0.3447	2.09E-162	3.82E-158
**MYLK**	-0.371129229	3.57E-105	6.52E-101	-0.46966	1.46E-220	2.66E-216
**MZT2B**	-0.276078954	1.24E-123	2.26E-119	-0.14807	1.65E-52	3.01E-48
**NACA**	-0.268202496	3.41E-178	6.23E-174	-0.21487	7.55E-146	1.38E-141
**NAMPT**	0.415128479	2.89E-127	5.26E-123	0.250267	1.10E-60	2.01E-56
**NAP1L1**	-0.40967988	1.08E-199	1.97E-195	-0.39303	2.54E-217	4.64E-213
**NBEAL1**	-0.260849408	6.98E-112	1.27E-107	0.043653	0.0034794	1
**NBPF14**	0.198524562	4.68E-60	8.55E-56	0.386182	1.45E-196	2.65E-192
**NDRG1**	0.457039683	5.69E-117	1.04E-112	0.678279	0	0
**NDUFA11**	-0.400336014	4.54E-189	8.28E-185	-0.40808	1.98E-229	3.61E-225
**NDUFB8**	0.066192547	0.000683	1	0.260752	5.31E-60	9.68E-56
**NEAT1**	-0.796160042	0	0	-0.94106	0	0
**NENF**	-0.136956847	2.69E-30	4.91E-26	-0.27225	7.39E-124	1.35E-119
**NFIA**	-0.390475603	3.65E-156	6.66E-152	-0.48899	0	0
**NFIC**	-0.293819013	1.53E-79	2.79E-75	-0.29248	1.02E-100	1.86E-96
**NFKBIA**	0.102219684	2.25E-12	4.10E-08	0.270574	7.51E-32	1.37E-27
**NGFRAP1**	-0.396595902	7.36E-163	1.34E-158	-0.81782	0	0
**NINJ1**	-0.297232679	1.15E-89	2.09E-85	-0.24215	3.31E-95	6.05E-91
**NKX3-2**	-0.31906555	8.88E-112	1.62E-107	-0.27554	9.55E-153	1.74E-148

**NME4**	-0.092680447	2.97E-12	5.42E-08	-0.27343	1.02E-94	1.86E-90
**NMRK1**	-0.270085709	3.95E-80	7.21E-76	-0.28336	1.19E-118	2.17E-114
**NNMT**	-0.379006291	1.79E-109	3.27E-105	-0.56703	3.31E-220	6.04E-216
**NOL7**	0.200199227	8.88E-30	1.62E-25	0.317616	5.24E-96	9.56E-92
**NOVA1**	0.186444544	4.55E-09	8.30E-05	-0.33522	3.08E-60	5.61E-56
**NPC2**	-0.547508189	0	0	-0.57538	0	0
**NPM1**	-0.340699168	1.02E-117	1.85E-113	-0.22266	4.97E-66	9.07E-62
**NPR3**	0.28304238	3.14E-148	5.74E-144	0.370328	5.47E-232	9.99E-228
**NRN1**	0.372515651	2.33E-35	4.25E-31	0.239571	1.62E-34	2.95E-30
**NRP2**	0.389938487	1.92E-112	3.51E-108	0.896849	0	0
**NT5C3A**	0.379480961	8.52E-98	1.56E-93	0.177167	7.04E-42	1.29E-37
**NT5E**	0.066154529	1.44E-07	0.00262	0.31035	1.29E-98	2.36E-94
**NTMT1**	0.256162582	1.24E-64	2.25E-60	0.352127	2.19E-137	4.00E-133
**NUCB1**	-0.024461981	0.161962	1	-0.27534	8.87E-87	1.62E-82
**NUCB2**	-0.326035717	4.88E-69	8.90E-65	-0.40914	1.44E-154	2.63E-150
**NUPR1**	-0.244007185	1.52E-43	2.77E-39	-0.36249	2.35E-137	4.30E-133
**OAF**	-0.280432546	1.03E-73	1.89E-69	-0.32375	5.66E-136	1.03E-131
**OGN**	-1.078087937	0	0	-1.55808	0	0
**ORM1**	0.474674845	3.29E-46	6.00E-42	0.43969	3.22E-30	5.87E-26
**ORM2**	0.145793632	1.58E-11	2.89E-07	0.273606	4.58E-26	8.35E-22
**OS9**	-0.127449476	1.41E-19	2.58E-15	-0.26038	2.67E-87	4.86E-83
**OSBPL9**	-0.250663295	8.26E-54	1.51E-49	-0.21343	1.82E-65	3.33E-61
**OXA1L**	-0.356925375	4.10E-115	7.49E-111	-0.24631	3.17E-67	5.79E-63
**P4HB**	-0.570607535	1.49E-243	2.73E-239	-0.78829	0	0
**PABPC1**	-0.460574462	1.03E-231	1.89E-227	-0.56531	0	0
**PAM**	0.196305144	1.41E-28	2.57E-24	-0.25098	6.86E-80	1.25E-75
**PAX1**	-0.812142646	0	0	-1.00815	0	0
**PCBP2**	-0.40973104	1.81E-218	3.30E-214	-0.43098	1.61E-276	2.94E-272
**PCOLCE2**	-0.036591022	0.025366	1	-0.42227	8.06E-169	1.47E-164
**PDE4B**	0.435873712	1.64E-183	2.99E-179	0.397643	2.49E-182	4.54E-178
**PDE4DIP**	-0.35474254	2.33E-121	4.25E-117	-0.42978	9.22E-221	1.68E-216
**PDIA3**	-0.233138353	1.48E-41	2.70E-37	-0.44664	1.39E-176	2.54E-172
**PDIA6**	-0.40303167	4.19E-126	7.65E-122	-0.65016	0	0
**PEBP1**	-0.425279972	1.12E-205	2.04E-201	-0.55665	0	0
**PFDN2**	0.183872715	2.27E-28	4.13E-24	0.369926	1.98E-144	3.61E-140
**PGAM1**	-0.259094944	1.44E-69	2.64E-65	-0.25301	1.47E-90	2.68E-86
**PGK1**	0.380349906	1.05E-97	1.91E-93	0.418274	1.59E-146	2.90E-142
**PHLDA1**	0.869958322	1.57E-244	2.86E-240	0.6237	2.81E-255	5.12E-251
**PID1**	0.203109864	2.51E-24	4.57E-20	0.303355	4.46E-61	8.14E-57

**PIK3R1**	-0.164332366	9.35E-22	1.71E-17	-0.25189	3.81E-89	6.95E-85
**PIM3**	-0.134714195	8.77E-13	1.60E-08	-0.32985	1.23E-102	2.24E-98
**PKM**	-0.217721915	4.40E-32	8.03E-28	-0.37436	8.30E-143	1.51E-138
**PLA2G16**	-0.314801484	5.16E-99	9.42E-95	-0.2594	2.37E-96	4.32E-92
**PLA2G2A**	1.370606499	0	0	0.126266	0.0084414	1
**PLA2G4A**	0.327739529	2.30E-116	4.19E-112	0.211714	1.00E-73	1.83E-69
**PLAC9**	-0.423987588	2.82E-133	5.14E-129	-1.28233	0	0
**PLAUR**	0.343950481	3.49E-93	6.38E-89	0.520776	5.92E-245	1.08E-240
**PLCG2**	0.133604367	4.34E-24	7.91E-20	0.470338	2.15E-104	3.92E-100
**PLD3**	-0.043599125	1.16E-08	0.000211	-0.29713	2.33E-116	4.26E-112
**PLP2**	-0.250071972	1.43E-68	2.61E-64	-0.06329	1.54E-08	0.000281
**PLPP1**	-0.486484433	2.13E-131	3.89E-127	-0.50353	1.85E-206	3.38E-202
**PLXDC2**	-0.370895384	1.39E-108	2.53E-104	-0.46294	4.12E-226	7.51E-222
**PMP22**	0.51733219	3.28E-137	5.98E-133	0.74764	0	0
**PNISR**	-0.228026145	2.87E-52	5.23E-48	-0.33643	1.73E-133	3.16E-129
**PNKD**	-0.093776692	9.71E-15	1.77E-10	-0.27103	6.91E-123	1.26E-118
**PNRC1**	0.059033604	0.829217	1	-0.26635	7.21E-116	1.32E-111
**POLR2L**	-0.140835779	7.73E-16	1.41E-11	-0.34664	3.52E-126	6.42E-122
**PPFIBP1**	0.394367815	2.25E-83	4.10E-79	0.432862	9.27E-138	1.69E-133
**PPIA**	-0.340137456	4.58E-171	8.36E-167	-0.26948	1.14E-139	2.08E-135
**PPIB**	-0.199311708	4.35E-37	7.94E-33	-0.3102	2.03E-103	3.71E-99
**PPIC**	-0.351470832	2.07E-80	3.77E-76	-0.5053	1.80E-213	3.28E-209
**PPP1CB**	0.546811979	2.70E-116	4.93E-112	0.545931	7.70E-161	1.40E-156
**PPP1R14C**	0.196454822	8.05E-74	1.47E-69	0.321433	7.08E-178	1.29E-173
**PPP1R15A**	0.42355969	4.56E-125	8.32E-121	0.565643	9.42E-244	1.72E-239
**PPP1R3C**	0.07031931	0.000616	1	-0.40155	3.65E-138	6.67E-134
**PPP3CA**	0.895530043	0	0	0.621868	1.04E-216	1.90E-212
**PPT1**	-0.282680136	8.64E-82	1.58E-77	-0.38217	9.55E-184	1.74E-179
**PRCP**	-0.259463286	7.75E-86	1.41E-81	-0.30823	2.01E-171	3.68E-167
**PRDX4**	-0.183932027	4.44E-34	8.11E-30	-0.43837	3.02E-174	5.52E-170
**PRDX5**	-0.270865933	8.73E-102	1.59E-97	-0.32704	2.05E-163	3.75E-159
**PRELP**	0.074196163	0.069956	1	-0.59135	5.83E-262	1.06E-257
**PRG4**	1.357437599	0	0	1.997413	0	0
**PRSS23**	-0.286324437	1.02E-15	1.86E-11	-0.27661	2.56E-26	4.68E-22
**PRUNE2**	-0.44340507	1.24E-135	2.26E-131	-0.41492	4.55E-201	8.30E-197
**PSAP**	-0.282556266	9.04E-87	1.65E-82	-0.42044	1.21E-203	2.20E-199
**PSMA7**	-0.346093411	8.46E-123	1.54E-118	-0.01005	0.3378195	1
**PTGES**	0.214775527	6.90E-95	1.26E-90	0.27336	6.56E-156	1.20E-151
**PTGES3**	0.324783234	4.73E-112	8.62E-108	0.379671	2.89E-173	5.28E-169

**PTGS2**	1.11478136	0	0	1.189169	0	0
**PTMS**	-0.376605247	3.15E-135	5.75E-131	-0.47495	3.63E-282	6.62E-278
**PTP4A1**	0.536434095	1.58E-126	2.89E-122	0.44409	2.72E-137	4.96E-133
**PTPRD-AS1**	-0.2254385	1.40E-56	2.56E-52	-0.3002	1.89E-129	3.44E-125
**PTRF**	-0.495446008	2.16E-182	3.95E-178	-0.53925	1.12E-269	2.04E-265
**RAB13**	0.268377603	1.23E-52	2.24E-48	0.231329	1.31E-51	2.40E-47
**RAB1A**	0.27671385	2.26E-65	4.13E-61	0.163201	3.25E-26	5.93E-22
**RABAC1**	-0.363523813	4.53E-153	8.27E-149	-0.36007	7.45E-184	1.36E-179
**RABGGTB**	0.399869235	6.65E-132	1.21E-127	0.638346	0	0
**RARRES3**	-0.262226806	5.00E-111	9.12E-107	-0.26378	1.13E-176	2.06E-172
**RBM3**	-0.248216709	2.30E-53	4.19E-49	-0.2644	6.52E-79	1.19E-74
**RBP4**	-0.084257377	1.90E-27	3.46E-23	-0.7101	0	0
**RCN1**	-0.163560822	1.42E-24	2.59E-20	-0.28617	2.39E-91	4.35E-87
**RELB**	0.284116965	2.56E-83	4.68E-79	0.530461	4.34E-287	7.91E-283
**RGCC**	-0.122823966	0.125307	1	0.434918	3.71E-121	6.76E-117
**RGS10**	-0.314814699	6.40E-91	1.17E-86	-0.0722	1.36E-11	2.49E-07
**RGS16**	0.239005653	1.58E-15	2.89E-11	0.404056	3.35E-51	6.10E-47
**RGS2**	0.14626965	2.56E-29	4.68E-25	0.376537	8.12E-54	1.48E-49
**RHOBTB3**	-0.530301361	1.55E-197	2.82E-193	-0.77459	0	0
**RIPK2**	0.135405785	4.27E-25	7.79E-21	0.299301	1.26E-127	2.30E-123
**RNASET2**	-0.11005092	2.10E-16	3.83E-12	-0.25175	7.19E-106	1.31E-101
**RND3**	0.207229247	1.06E-48	1.93E-44	0.477802	1.83E-167	3.35E-163
**RNF114**	0.177533484	2.45E-19	4.47E-15	0.466091	7.06E-170	1.29E-165
**RNF126**	0.250328024	3.24E-68	5.91E-64	0.270064	2.92E-95	5.33E-91
**RNF130**	-0.203023328	2.08E-45	3.80E-41	-0.36818	1.52E-150	2.77E-146
**RNF145**	0.441947215	5.02E-132	9.15E-128	0.528494	1.55E-199	2.84E-195
**RNMT**	0.328772294	5.03E-82	9.18E-78	0.259755	7.16E-58	1.31E-53
**RP11-553L6.5**	-0.190659703	4.99E-45	9.10E-41	-0.26309	3.19E-127	5.82E-123
**RP11-71N10.1**	-0.259891993	3.73E-121	6.80E-117	-0.23785	8.11E-163	1.48E-158
**RP11-983P16.4**	-0.203744686	5.79E-46	1.06E-41	-0.32001	3.43E-150	6.25E-146
**RP5-1097P24.1**	-0.215130415	2.24E-63	4.09E-59	-0.34165	7.55E-181	1.38E-176
**RPA3**	-0.25951939	1.29E-73	2.35E-69	-0.16398	4.28E-37	7.82E-33
**RPL10**	-0.399052674	0	0	-0.33251	0	0
**RPL10A**	-0.600013881	0	0	-0.58749	0	0
**RPL11**	-0.291537797	5.09E-291	9.29E-287	-0.24152	1.94E-265	3.55E-261
**RPL13**	-0.335580039	0	0	-0.29511	0	0
**RPL13A**	-0.4240303	0	0	-0.35161	0	0
**RPL14**	-0.415023066	0	0	-0.28396	1.34E-278	2.45E-274
**RPL15**	-0.278245026	0	0	-0.28651	0	0

**RPL18**	-0.560658654	0	0	-0.39718	0	0
**RPL18A**	-0.361013653	0	0	-0.23731	4.38E-250	7.99E-246
**RPL19**	-0.377446596	0	0	-0.36404	0	0
**RPL21**	-0.367858429	0	0	-0.21813	1.97E-220	3.59E-216
**RPL22**	-0.311641152	5.29E-237	9.65E-233	-0.26272	2.86E-208	5.22E-204
**RPL23**	-0.502072008	0	0	-0.37561	0	0
**RPL23A**	-0.491563689	0	0	-0.41789	0	0
**RPL27**	-0.362528094	1.02E-303	1.86E-299	-0.21471	2.93E-151	5.35E-147
**RPL27A**	-0.295372093	6.47E-281	1.18E-276	-0.1821	4.70E-162	8.57E-158
**RPL28**	-0.271941912	2.35E-252	4.29E-248	-0.16079	6.24E-134	1.14E-129
**RPL29**	-0.366443184	0	0	-0.23241	2.11E-223	3.85E-219
**RPL3**	-0.368806953	0	0	-0.43298	0	0
**RPL30**	-0.46893798	0	0	-0.38014	0	0
**RPL31**	-0.388772276	0	0	-0.18681	2.48E-136	4.52E-132
**RPL34**	-0.501580745	0	0	-0.43144	0	0
**RPL35**	-0.324073533	0	0	-0.19545	1.91E-172	3.48E-168
**RPL35A**	-0.328356236	0	0	-0.1883	5.84E-151	1.07E-146
**RPL36AL**	-0.257852895	1.75E-112	3.20E-108	-0.13732	1.85E-40	3.38E-36
**RPL37**	-0.430016787	0	0	-0.27035	1.59E-290	2.90E-286
**RPL37A**	-0.344437927	0	0	-0.2519	0	0
**RPL39**	-0.30553987	1.73E-253	3.16E-249	-0.1597	1.69E-100	3.09E-96
**RPL4**	-0.603032179	0	0	-0.67059	0	0
**RPL5**	-0.250473411	3.62E-150	6.60E-146	-0.31593	2.08E-277	3.79E-273
**RPL6**	-0.224920895	7.03E-146	1.28E-141	-0.28609	8.89E-281	1.62E-276
**RPL7**	-0.569407136	0	0	-0.52936	0	0
**RPL7A**	-0.29003522	5.24E-249	9.55E-245	-0.27743	3.18E-289	5.81E-285
**RPL8**	-0.387690008	0	0	-0.31745	0	0
**RPL9**	-0.419501355	0	0	-0.37862	0	0
**RPLP0**	-0.735199464	0	0	-0.57104	0	0
**RPLP1**	-0.373397583	0	0	-0.29514	0	0
**RPLP2**	-0.52177402	0	0	-0.38167	0	0
**RPN2**	-0.211585734	4.37E-52	7.98E-48	-0.33094	8.68E-156	1.58E-151
**RPS11**	-0.996321936	0	0	-0.67142	0	0
**RPS12**	-0.321608688	1.06E-291	1.94E-287	-0.27011	1.22E-272	2.22E-268
**RPS13**	-0.430611059	0	0	-0.32814	0	0
**RPS14**	-0.256522044	2.05E-260	3.74E-256	-0.18167	1.96E-182	3.57E-178
**RPS15A**	-0.267163197	4.69E-237	8.55E-233	-0.07521	5.31E-34	9.69E-30
**RPS16**	-0.540914761	0	0	-0.40861	0	0
**RPS17**	-0.531098443	0	0	-0.36662	0	0

**RPS18**	-0.390598893	0	0	-0.29434	0	0
**RPS19**	-0.268172186	1.58E-228	2.89E-224	-0.21834	1.54E-185	2.81E-181
**RPS2**	-0.411142258	0	0	-0.35477	0	0
**RPS20**	-0.989254592	0	0	-0.66435	0	0
**RPS21**	-0.288315569	4.97E-220	9.07E-216	-0.11658	8.21E-55	1.50E-50
**RPS23**	-0.386972626	0	0	-0.31687	0	0
**RPS24**	-0.284117856	3.01E-219	5.49E-215	-0.18546	6.91E-132	1.26E-127
**RPS25**	-0.290318986	6.45E-243	1.18E-238	-0.2403	1.31E-202	2.40E-198
**RPS27**	-0.32760625	9.94E-293	1.81E-288	-0.17803	4.15E-120	7.57E-116
**RPS27A**	-0.431504219	0	0	-0.34714	0	0
**RPS29**	-0.467745767	0	0	-0.28703	0	0
**RPS3**	-0.283168784	5.21E-285	9.51E-281	-0.26529	0	0
**RPS3A**	-0.511330521	0	0	-0.45149	0	0
**RPS4X**	-0.263612511	2.54E-239	4.64E-235	-0.30781	0	0
**RPS4Y1**	-0.23473409	4.59E-100	8.37E-96	-0.28762	2.89E-162	5.28E-158
**RPS5**	-0.367780398	0	0	-0.32009	0	0
**RPS6**	-0.313071371	0	0	-0.32879	0	0
**RPS7**	-0.374114298	0	0	-0.31652	0	0
**RPS8**	-0.637925867	0	0	-0.61223	0	0
**RPS9**	-0.299823187	7.46E-278	1.36E-273	-0.29039	0	0
**RPSA**	-0.453682315	0	0	-0.34579	5.49E-275	1.00E-270
**S100A1**	-0.296795191	1.12E-114	2.05E-110	-0.49437	0	0
**S100A13**	-0.237534744	4.10E-107	7.48E-103	-0.38199	9.21E-284	1.68E-279
**S100A16**	0.099340413	1.55E-06	0.028353	0.346662	1.81E-89	3.30E-85
**S100A2**	0.985003003	2.41E-174	4.41E-170	1.126137	0	0
**S100A4**	-0.345899812	4.90E-38	8.93E-34	-0.48364	3.19E-106	5.83E-102
**S100B**	-0.051730395	4.12E-15	7.51E-11	-0.78257	0	0
**SAR1A**	0.283855111	6.41E-67	1.17E-62	0.305322	2.93E-90	5.35E-86
**SARAF**	-0.095110849	8.92E-12	1.63E-07	-0.25676	2.62E-84	4.79E-80
**SAT1**	0.253304341	5.14E-53	9.38E-49	0.017158	0.3346245	1
**SCRG1**	0.082860694	0.013475	1	-0.30437	1.877E-79	3.43E-75
**SDC2**	-0.332100401	1.48E-87	2.71E-83	-0.53649	3.41E-274	6.22E-270
**SDF4**	-0.283235366	1.57E-85	2.87E-81	-0.46365	8.07E-232	1.47E-227
**SEC11A**	-0.475524619	4.62E-259	8.44E-255	-0.41467	3.13E-257	5.72E-253
**SELK**	0.285640658	4.87E-73	8.89E-69	0.378225	9.62E-141	1.76E-136
**SEPP1**	-0.192352728	3.83E-49	6.99E-45	-0.396	6.57E-196	1.20E-191
**SERF2**	-0.056523476	1.53E-06	0.027863	-0.30604	1.00E-163	1.83E-159
**SERPINA5**	-0.460785663	1.06E-145	1.94E-141	-0.53898	5.85E-227	1.07E-222
**SERPINE1**	0.291447657	5.46E-88	9.97E-84	0.482349	1.56E-182	2.84E-178

**SERPINE2**	0.686987933	3.68E-186	6.71E-182	0.775334	1.42E-241	2.59E-237
**SFT2D1**	0.203116495	8.37E-39	1.53E-34	0.255385	1.73E-78	3.16E-74
**SGIP1**	0.372898096	2.57E-173	4.70E-169	0.480338	3.88E-289	7.08E-285
**SH3BGRL**	-0.220492437	1.53E-56	2.79E-52	-0.40485	7.96E-196	1.45E-191
**SH3BP5**	0.281808366	1.19E-42	2.17E-38	0.356151	1.98E-102	3.62E-98
**SH3GLB1**	0.005662482	0.911977	1	0.255229	8.98E-71	1.64E-66
**SLC14A1**	-0.559160275	5.60E-223	1.02E-218	-0.53924	1.28E-300	2.34E-296
**SLC1A5**	-0.128711153	1.71E-22	3.11E-18	-0.27594	1.63E-130	2.98E-126
**SLC20A1**	0.271628319	7.55E-68	1.38E-63	0.41148	6.07E-169	1.11E-164
**SLC25A3**	-0.407225694	1.57E-195	2.86E-191	-0.26565	5.20E-114	9.48E-110
**SLC25A36**	0.296803911	9.64E-77	1.76E-72	0.26363	6.94E-68	1.27E-63
**SLC25A37**	0.585658652	2.34E-218	4.27E-214	0.614924	0	0
**SLC29A1**	-0.260938801	1.56E-78	2.84E-74	-0.18663	3.18E-59	5.81E-55
**SLC2A1**	0.546497282	1.10E-155	2.00E-151	0.617201	2.13E-284	3.89E-280
**SLC38A1**	-0.253385768	3.53E-78	6.44E-74	-0.31405	8.57E-156	1.56E-151
**SLC39A8**	0.351690559	7.96E-107	1.45E-102	0.053007	0.0003806	1
**SLC3A2**	0.515566623	1.47E-97	2.68E-93	0.607648	1.44E-203	2.64E-199
**SLC40A1**	-0.786406252	1.77E-287	3.24E-283	-0.6631	0	0
**SLC44A1**	-0.34242341	5.20E-111	9.49E-107	-0.31791	1.15E-134	2.10E-130
**SLC5A3**	0.351336296	2.68E-45	4.89E-41	0.371444	3.84E-63	7.01E-59
**SLC7A2**	0.19064044	2.40E-53	4.37E-49	0.440427	1.42E-192	2.59E-188
**SLPI**	-0.118154094	7.37E-25	1.35E-20	-1.53355	0	0
**SMIM14**	-0.131479983	1.24E-22	2.26E-18	-0.25416	1.19E-87	2.18E-83
**SMIM3**	0.297005171	1.23E-125	2.25E-121	0.196389	1.79E-78	3.27E-74
**SMOX**	0.316539717	8.83E-54	1.61E-49	0.760869	8.93E-293	1.63E-288
**SMS**	0.165691709	1.27E-17	2.32E-13	0.314466	8.30E-74	1.51E-69
**SNAI2**	-0.222412539	1.20E-36	2.19E-32	-0.65739	3.94E-247	7.19E-243
**SNHG12**	0.242920247	4.41E-42	8.05E-38	0.643826	2.70E-288	4.93E-284
**SNRNP27**	0.26091916	2.05E-71	3.74E-67	0.275948	2.01E-79	3.68E-75
**SOCS3**	-0.187395917	2.05E-34	3.73E-30	-0.26309	4.12E-88	7.53E-84
**SOD1**	-0.165076722	5.38E-45	9.82E-41	-0.26493	8.84E-109	1.61E-104
**SOD2**	0.554512124	4.82E-160	8.80E-156	1.278455	0	0
**SOD3**	-0.142208546	4.03E-08	0.000735	-0.54831	3.15E-169	5.76E-165
**SOX9**	0.105780382	8.31E-08	0.001517	-0.26217	1.36E-65	2.48E-61
**SPARC**	-0.794579106	7.12E-253	1.30E-248	-0.63089	0	0
**SPINT2**	-0.388831955	1.63E-131	2.97E-127	-0.39017	4.13E-170	7.54E-166
**SPON2**	-0.216316552	1.01E-34	1.84E-30	-0.498	3.50E-272	6.39E-268
**SPP1**	1.967115816	0	0	2.760795	0	0
**SPRY2**	0.420645313	9.16E-154	1.67E-149	0.532124	1.79E-295	3.26E-291

**SPTLC3**	-0.334604693	3.73E-74	6.80E-70	-0.27506	3.35E-85	6.11E-81
**SPTSSB**	-1.439238522	0	0	-1.46735	0	0
**SQSTM1**	0.081289696	1.59E-06	0.029064	0.36613	2.71E-99	4.94E-95
**SRGN**	0.276315096	1.76E-55	3.21E-51	0.03765	0.569085	1
**SRSF5**	-0.125072284	5.95E-19	1.09E-14	-0.2817	2.20E-94	4.02E-90
**SSPN**	-0.373958685	2.21E-135	4.03E-131	-0.43388	5.28E-251	9.63E-247
**SSR2**	-0.458879589	6.71E-232	1.23E-227	-0.39392	8.66E-207	1.58E-202
**SSR3**	-0.503695353	5.38E-214	9.82E-210	-0.47655	3.19E-256	5.81E-252
**SSR4**	-0.304516646	3.09E-97	5.64E-93	-0.39563	7.70E-190	1.41E-185
**ST13**	-0.272378054	7.20E-98	1.31E-93	-0.3048	9.54E-140	1.74E-135
**STK17A**	0.67305458	9.52E-180	1.74E-175	0.893491	0	0
**SVIP**	0.053532502	0.007189	1	0.275301	2.39E-65	4.37E-61
**SYF2**	0.063708267	0.000741	1	0.305108	2.00E-92	3.65E-88
**TBCA**	0.143695969	1.57E-28	2.86E-24	0.253669	1.25E-103	2.28E-99
**TCEA3**	-0.185657665	1.63E-42	2.98E-38	-0.3059	4.61E-132	8.42E-128
**TCEAL8**	-0.221173872	1.88E-59	3.43E-55	-0.35364	1.91E-157	3.48E-153
**TCF4**	-0.555353189	8.84E-206	1.61E-201	-0.61326	0	0
**TFPI**	0.253117224	5.46E-87	9.96E-83	0.409543	4.11E-175	7.50E-171
**TFPI2**	0	NA	NA	0.341522	1.78E-102	3.24E-98
**TGFBI**	1.115436889	0	0	0.943265	6.97E-308	1.27E-303
**TGM2**	0.098732655	4.02E-11	7.34E-07	0.439775	2.00E-202	3.65E-198
**THY1**	-0.723040339	5.32E-165	9.71E-161	-0.63183	3.93E-214	7.17E-210
**TIMP1**	0.643646611	1.82E-75	3.32E-71	0.78624	1.04E-83	1.90E-79
**TIMP2**	-0.260358731	3.65E-66	6.66E-62	-0.1057	1.14E-19	2.08E-15
**TIMP3**	0.098902165	0.012343	1	0.759801	2.68E-181	4.89E-177
**TM4SF1**	0.326338148	1.72E-20	3.14E-16	1.00172	0	0
**TMED10**	-0.240675012	3.23E-60	5.89E-56	-0.44035	4.03E-210	7.36E-206
**TMED2**	-0.046658795	0.000226	1	-0.29723	1.91E-123	3.48E-119
**TMEM219**	-0.19856927	1.84E-50	3.36E-46	-0.29544	1.35E-116	2.46E-112
**TMEM230**	-0.08148184	2.48E-11	4.52E-07	-0.33432	2.03E-131	3.71E-127
**TMEM38B**	0.304631861	5.04E-67	9.19E-63	0.413674	4.04E-153	7.37E-149
**TMEM45A**	0.540571774	4.31E-159	7.86E-155	0.387245	7.51E-90	1.37E-85
**TMEM59**	-0.043239496	8.25E-06	0.150486	-0.32401	2.84E-119	5.18E-115
**TMEM98**	-0.163830055	1.02E-37	1.86E-33	-0.29725	2.35E-145	4.29E-141
**TMSB10**	0.187980108	1.99E-35	3.62E-31	0.528432	3.46E-212	6.31E-208
**TMSB4X**	0.104848561	1.38E-14	2.52E-10	0.425685	1.39E-50	2.53E-46
**TNC**	0	NA	NA	0.260979	2.78E-87	5.07E-83
**TNFAIP6**	0.308085839	3.58E-96	6.54E-92	0.204247	2.61E-64	4.77E-60
**TNFRSF11B**	0.226396661	2.57E-26	4.70E-22	0.279503	6.90E-21	1.26E-16

**TNFRSF12A**	0.622308688	1.81E-112	3.31E-108	0.800967	0	0
**TNFRSF1A**	-0.194998369	9.43E-40	1.72E-35	-0.26836	9.07E-86	1.65E-81
**TNFSF11**	0.272281382	5.72E-138	1.04E-133	0.216884	2.38E-121	4.35E-117
**TNS1**	0.20195313	8.51E-64	1.55E-59	0.276067	1.35E-131	2.46E-127
**TOMM7**	-0.370034314	0	0	-0.31597	6.82E-307	1.24E-302
**TPD52**	0.309333344	2.67E-76	4.88E-72	0.040003	0.1263158	1
**TPD52L1**	-0.165266308	3.26E-27	5.94E-23	-0.37745	4.82E-153	8.80E-149
**TPM1**	-0.60815029	9.76E-116	1.78E-111	-0.41675	5.44E-77	9.92E-73
**TPM2**	-0.222271274	5.84E-37	1.07E-32	-0.3334	3.12E-127	5.70E-123
**TPT1**	-0.55679477	0	0	-0.55298	0	0
**TREM1**	0.54984536	9.81E-299	1.79E-294	0.629349	0	0
**TRMT112**	0.227436813	5.79E-61	1.06E-56	0.381174	8.79E-173	1.60E-168
**TRPS1**	-0.236692745	6.64E-52	1.21E-47	-0.32428	2.86E-126	5.22E-122
**TSC22D1**	-0.632081354	1.76E-144	3.20E-140	-1.0115	0	0
**TSHZ2**	0.256886588	5.17E-123	9.43E-119	0.161631	1.37E-82	2.51E-78
**TSPAN3**	-0.159744055	4.21E-33	7.68E-29	-0.34476	3.67E-148	6.70E-144
**TSPAN4**	-0.255235332	3.80E-50	6.94E-46	-0.38808	1.47E-159	2.68E-155
**TSPO**	-0.374581658	4.28E-153	7.82E-149	-0.48163	1.99E-281	3.62E-277
**TTC3**	-0.341866508	8.81E-115	1.61E-110	-0.49969	1.54E-251	2.81E-247
**TUBB**	-0.441982163	1.67E-185	3.05E-181	-0.27873	2.33E-109	4.25E-105
**TUBB2B**	-0.427682451	3.72E-76	6.79E-72	-0.31117	2.72E-50	4.96E-46
**TVP23B**	0.207261806	2.53E-47	4.61E-43	0.278767	1.78E-86	3.24E-82
**TXNIP**	-0.299283363	1.19E-140	2.17E-136	-0.29632	4.92E-203	8.98E-199
**TYMP**	0.095233346	2.23E-12	4.07E-08	0.297859	2.30E-83	4.19E-79
**UBA52**	-0.375740064	0	0	-0.22431	5.52E-222	1.01E-217
**UBE2B**	0.105531061	4.76E-12	8.69E-08	0.26761	4.92E-90	8.97E-86
**UGP2**	-0.031055494	0.006753	1	-0.25854	3.32E-84	6.05E-80
**UMAD1**	-0.25094252	2.74E-54	5.00E-50	-0.28387	1.59E-109	2.90E-105
**UPP1**	0.779818858	1.26E-256	2.31E-252	1.272814	0	0
**UQCRB**	-0.479797226	1.80E-279	3.28E-275	-0.25177	2.60E-100	4.74E-96
**UQCRH**	-0.37673204	1.09E-211	1.98E-207	-0.26468	1.49E-145	2.72E-141
**USMG5**	0.008052511	0.419184	1	0.265916	1.01E-116	1.84E-112
**VAMP5**	-0.109590341	4.19E-15	7.64E-11	-0.27354	4.13E-93	7.54E-89
**VAPA**	0.413583535	1.27E-150	2.31E-146	0.577676	0	0
**VCAN**	-0.215304819	8.36E-35	1.53E-30	-0.44699	4.91E-162	8.97E-158
**VDAC1**	0.257269171	4.09E-44	7.46E-40	0.336878	9.71E-115	1.77E-110
**VEGFA**	1.135044052	0	0	1.027389	0	0
**VIM**	-0.606059102	2.69E-271	4.91E-267	-0.56591	0	0
**VIMP**	0.113215159	1.39E-11	2.54E-07	0.337029	2.86E-118	5.22E-114

**VKORC1**	-0.112391436	2.10E-15	3.83E-11	-0.38707	5.24E-179	9.57E-175
**VMP1**	0.223207265	2.40E-27	4.39E-23	0.416965	4.82E-92	8.80E-88
**WDR33**	0.281001819	1.40E-53	2.56E-49	0.352184	3.88E-100	7.08E-96
**WDR45B**	0.37695991	3.96E-116	7.23E-112	0.352001	1.61E-135	2.94E-131
**WSB1**	0.285670978	7.79E-75	1.42E-70	0.30563	3.44E-69	6.28E-65
**WTAP**	0.782914133	2.67E-213	4.87E-209	1.010374	0	0
**WWP2**	0.307214884	7.9E-46	1.44E-41	-0.26672	2.142E-59	3.91E-55
**XAGE2**	-0.673552506	2.32E-144	4.23E-140	-0.51369	1.60E-139	2.91E-135
**XBP1**	0.283093056	1.42E-48	2.59E-44	0.292176	1.99E-60	3.64E-56
**YBX1**	-0.399728116	1.53E-233	2.80E-229	-0.44095	0	0
**YBX3**	-0.613452969	0	0	-0.62471	0	0
**ZBTB20**	-0.38546287	1.01E-147	1.84E-143	-0.40969	1.39E-238	2.54E-234
**ZFAND5**	0.158420143	2.23E-19	4.06E-15	0.304339	4.53E-74	8.27E-70
**ZFAS1**	-0.291533576	3.06E-94	5.58E-90	0.069741	2.16E-09	3.94E-05
**ZFP36L2**	-0.962161339	0	0	-0.84876	0	0
**ZIC1**	-0.268645915	2.80E-63	5.12E-59	-0.23816	6.31E-96	1.15E-91
**ZNF385D**	0.292309231	3.86E-60	7.04E-56	0.434037	9.24E-153	1.69E-148
**ZNF593**	0.209237054	1.16E-43	2.11E-39	0.452242	9.99E-217	1.82E-212
**ZNF90**	-0.272972744	1.12E-77	2.04E-73	-0.00679	0.1956773	1
