# Supplementary material for: Single-Cell RNA-Seq Analysis of Cells from Degenerating and Non-Degenerating Intervertebral Discs from the Same Individual Reveals New Biomarkers for Intervertebral Disc Degeneration
Source: Int J Mol Sci. 2022 Apr 3;23(7):3993. doi: 10.3390/ijms23073993 (PMC8999935; doi:10.3390/ijms23073993)
Supplement: Supplementary file 1 [file ijms-23-03993-s001.zip › ijms-1655681-SI/Table S9.pdf]

Table S9: Opposite gene expression in NPnD and AFnD in total cell analysis.

Gene	logFC.AF	Pval.AF	Fdr.AF		logFC.NP	Pval.NP	Fdr.NP
**BAMBI**	0.001226699	1.95E-02	1.00E+00		-0.44419	7.16E-116	1.3E-111
**C10orf10**	0.302770071	1.28E-34	2.34E-30		-0.09743	5.718E-07	0.010434
**C1S**	0.0050643	3.64E-01	1.00E+00		-0.27177	4.952E-83	9.04E-79
**C2orf40**	0.209268514	1.59E-06	2.90E-02		-0.80392	0	0
**C7orf73**	0.021609485	4.74E-06	8.64E-02		-0.27222	5.41E-126	9.88E-122
**CCDC80**	0.33012088	1.06E-29	1.94E-25		-0.46874	3.66E-170	6.67E-166
**CHI3L1**	0.416970778	5.73E-58	1.04E-53		-0.49678	1.63E-82	2.97E-78
**CTSD**	0.798865369	3.20E-129	5.84E-125		-0.42878	1.37E-195	2.50E-191
**DUSP1**	0.004863469	0.004482	1		-0.3847	3.12E-124	5.69E-120
**EGR1**	0.383555074	7.02E-54	1.28E-49		-0.01361	0.0123146	1
**EPYC**	0.805758755	1.78E-174	3.25E-170		-0.05459	7.04E-09	0.000128
**FGFBP2**	0.065406456	1.25E-06	0.022786		-0.37073	2.00E-61	3.65E-57
**FXYD1**	0.020384998	3.86E-05	0.705024		-0.29932	6.93E-74	1.27E-69
**HIF-1a**	0.251163061	2.92E-39	5.32E-35		-0.0147	2.13E-05	0.389002
**ITM2B**	0.076385364	0.211905	1		-0.32983	2.73E-123	4.98E-119
**JUNB**	0.039095944	1.43E-06	0.026066		-0.55778	6.09E-224	1.11E-219
**MT1F**	0.388752241	2.64E-47	4.81E-43		-0.0527	0.0027746	1
**PNRC1**	0.059033604	0.829217	1		-0.26635	7.21E-116	1.32E-111
**PRELP**	0.074196163	0.069956	1		-0.59135	5.83E-262	1.06E-257
**SCRG1**	0.082860694	0.013475	1		-0.30437	1.877E-79	3.43E-75
**SOX9**	0.105780382	8.31E-08	0.001517		-0.26217	1.36E-65	2.48E-61
**EIF1**	-0.023088268	0.047079	1		0.488611	0	0
**LGALS1**	-0.335409178	2.88E-53	5.26E-49		0.120207	0.9493502	1
**RGCC**	-0.122823966	0.125307	1		0.434918	3.71E-121	6.76E-117

**Avg_logFC**
Decrease
Increase
